# Local Effect of Enhancer of Zeste-Like Reveals Cooperation of Epigenetic and *cis*-Acting Determinants for Zygotic Genome Rearrangements

**DOI:** 10.1371/journal.pgen.1004665

**Published:** 2014-09-25

**Authors:** Maoussi Lhuillier-Akakpo, Andrea Frapporti, Cyril Denby Wilkes, Mélody Matelot, Michel Vervoort, Linda Sperling, Sandra Duharcourt

**Affiliations:** 1 Institut Jacques Monod, CNRS, UMR 7592, Université Paris Diderot, Sorbonne Paris Cité, Paris, France; 2 Sorbonne Universités, UPMC Univ., IFD, Paris, France; 3 CNRS UPR3404 Centre de Génétique Moléculaire, Gif-sur-Yvette, France; 4 Département de Biologie, Université Paris-Sud, Orsay, France; 5 Institut Universitaire de France, Paris, France; Fred Hutchinson Cancer Research Center, United States of America

## Abstract

In the ciliate *Paramecium tetraurelia*, differentiation of the somatic nucleus from the zygotic nucleus is characterized by massive and reproducible deletion of transposable elements and of 45,000 short, dispersed, single-copy sequences. A specific class of small RNAs produced by the germline during meiosis, the scnRNAs, are involved in the epigenetic regulation of DNA deletion but the underlying mechanisms are poorly understood. Here, we show that trimethylation of histone H3 (H3K27me3 and H3K9me3) displays a dynamic nuclear localization that is altered when the endonuclease required for DNA elimination is depleted. We identified the putative histone methyltransferase Ezl1 necessary for H3K27me3 and H3K9me3 establishment and show that it is required for correct genome rearrangements. Genome-wide analyses show that scnRNA-mediated H3 trimethylation is necessary for the elimination of long, repeated germline DNA, while single copy sequences display differential sensitivity to depletion of proteins involved in the scnRNA pathway, Ezl1- a putative histone methyltransferase and Dcl5- a protein required for iesRNA biogenesis. Our study reveals *cis*-acting determinants, such as DNA length, also contribute to the definition of germline sequences to delete. We further show that precise excision of single copy DNA elements, as short as 26 bp, requires Ezl1, suggesting that development specific H3K27me3 and H3K9me3 ensure specific demarcation of very short germline sequences from the adjacent somatic sequences.

## Author Summary

The unicellular eukaryote *Paramecium tetraurelia* provides an extraordinary model for studying the mechanisms involved in zygotic genome rearrangements. At each sexual cycle, differentiation of the somatic nucleus from the zygotic nucleus is characterized by extensive remodeling of the entire somatic genome, which includes the precise excision of 45,000 short noncoding germline DNA segments to reconstitute functional open reading frames. Exploiting the unique properties of the *Paramecium* genome, we show that the enhancer of zeste like protein Ezl1 is necessary for histone H3 trimethylation on lysines 27 and 9 and is required for the precise excision of 31,000 of these single copy, dispersed germline DNA segments that can be as short as 26 bp in length. This implies that histone marks usually associated with heterochromatin may contribute to the precise demarcation of segments that are even shorter than the length of DNA wrapped around a single nucleosome. A quantitative analysis of high throughput sequencing datasets further shows that the underlying genetic properties of the germline DNA segments might act in concert with epigenetic signals to define germline specific sequences.

## Introduction

Ciliates provide extraordinary model organisms with which to gain understanding into the organization of eukaryotic genomes. The differentiation of the somatic nucleus from the zygotic nucleus is characterized by massive and reproducible rearrangements at the DNA level [1]. In *Paramecium tetraurelia*, as in all ciliates, germline and somatic functions are separated between two distinct nuclei that coexist in the same cytoplasm. During vegetative growth, the diploid germline micronucleus (MIC) remains transcriptionally silent, while the highly polyploid somatic macronucleus (MAC) supports gene expression. During sexual events, the MAC is fragmented and eventually lost, whereas the MIC undergoes meiosis and transmits the germline genome to the new MIC and MAC of the next generation. During the differentiation of the zygotic MAC, germline-specific regions up to several kbp in length, often containing repetitive sequences, are imprecisely eliminated leading to germline chromosome fragmentation or intra-chromosomal deletions. Moreover, and this is a critical point for the present study, 45,000 single-copy, short and non-coding Internal Eliminated Sequences (IES) are excised precisely from intergenic and genic regions. These « DNA introns » are found throughout the germline genome, appear to be remnants of ancestral insertions of transposable elements (TEs) [2], and are invariably flanked by two 5′- TA -3′ dinucleotide repeats, one of which is left after excision. All DNA elimination events rely on the domesticated piggyBac transposase, PiggyMac (Pgm), which is essential to introduce DNA cleavages at each IES boundary [2], [3].

Precision of IES excision is critical for the assembly of functional genes in the somatic genome and the survival of sexual progeny. Yet the weak consensus found at IES ends is not sufficient to determine the excision pattern across the whole genome [2], [4]. The molecular mechanisms underlying the specific recognition of such a large number of different germline sequences remain poorly understood. A class of small RNAs that resemble the metazoan piRNAs, called the scnRNAs, is produced by the meiosis-specific Dicer-like proteins Dcl2 and Dcl3 [5], [6]. scnRNAs are required to promote IES excision [7]. In the current “genome scanning” model, scnRNAs are produced from most of the germline genome during MIC meiosis and are then filtered by pairing interactions with nascent transcripts in the maternal MAC, resulting in the selective inactivation of those able to find a perfect match, and thus in the selection of MIC-specific scnRNAs [8]. Once selected by this “scanning” procedure, MIC-specific scnRNAs would be exported to the developing zygotic MAC to target homologous sequences, thereby recruiting the excision machinery [9]. This RNA-mediated genomic subtraction can explain the epigenetic inheritance of alternative rearrangement patterns, such as retention of a given IES in the MAC [10], [11], deletion of a given gene [12] or mating type determination [13] across sexual generations. The scnRNA pathway is conserved in the distantly related ciliate *Tetrahymena thermophila*, where scnRNA-mediated tri-methylation of histone H3 on lysine 9 and lysine 27 (H3K9me3 and H3K27me3) [14]–[17] is thought to guide the recruitment of an endonuclease [18], [19] initiating the deletion of germline sequences. As observed in small RNA-guided heterochromatin formation in other organisms [20], [21], the data obtained so far support the idea that heterochromatin formation occurs downstream of the scnRNA pathway and leads to the imprecise elimination of long, repetitive germline sequences, which are nearly all found in intergenic regions in *Tetrahymena* [22]. Recently, a class of 26-30 nt long, IES-specific *Paramecium* sRNAs, called iesRNAs, was reported [6]. iesRNAs accumulate during late MAC development and require the Dicer-like protein Dcl5 for their biogenesis. Dcl5 depletion leads to partial impairment of excision of a small fraction of IESs. The precise role of iesRNAs in IES excision remains to be elucidated.

The chromatin modifications that may guide the Pgm endonuclease to specific germline sequences are not yet characterized in *Paramecium*. The vast majority of IESs are shorter than 150 bp and some are as short as 26 bp; they are thus not even as long as the DNA wrapped around a single nucleosome. Excision of these 45,000 DNA segments must require a marking mechanism of considerable precision, allowing the demarcation of these very short, numerous, interspersed germline sequences from adjacent retained somatic sequences. The present study was designed to test the involvement of histone H3 methylation in the DNA elimination process, with a special interest for its role on IES excision. We show here that the putative histone methyltransferase (HMT) Ezl1 is required for the accumulation of H3K27me3 and H3K9me3 in the developing somatic macronucleus. Re-sequencing the genome following Ezl1 depletion showed that EZL1 is required for correct genome rearrangements. We found that scnRNA-mediated H3K27me3 and H3K9me3 is necessary for the elimination of a fraction of germline DNA, including transposable elements and long IESs. Strikingly, the putative HMT Ezl1 is also required for the precise excision of about 70% of the 45,000 short, unique copy IESs, providing evidence that it may contribute to the precise demarcation of short germline sequences. Our genome wide study shows that IESs display differential sensitivity to depletion of the scnRNA pathway, Dcl5 or Ezl1 proteins and identifies *cis* acting determinants, such as DNA length that might act in concert with epigenetic signals to define germline specific sequences.

## Results

### Dynamic localization of histone H3 tri-methylation on K27 and on K9 in the developing MAC

**Figure 1:**
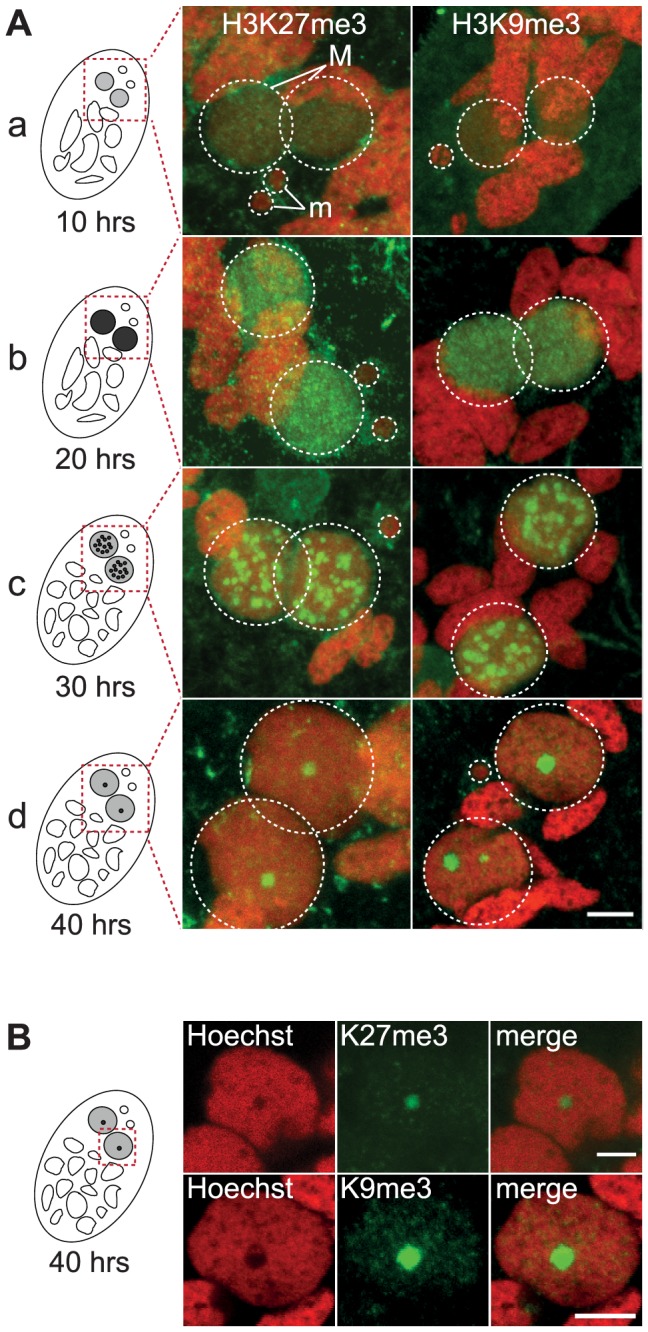
Dynamic localization of H3K27me3 and H3K9me3 in the developing somatic macronuclei (MAC). **A**. Immunofluorescence of cells at different stages of nuclear differentiation are shown, aligned with their schematic representations on the left: (a) 10 hrs; (b) 20 hrs; (c) 30 hrs; (d) 40 hrs. The time points refer to hours after T =  0 hr that is defined as the time when cells begin fragmentation of the maternal MAC, as evaluated by cytological observation. See Figure S4 for details on progression of autogamy and quantification of the number of stained cells at each time point. Overlay of Z-projections of magnified views of H3K27me3- or H3K9me3- specific antibodies (in green), and Hoechst (in red) on selected stacks are presented. Dashed white circles indicate the two developing MACs (M) and the MICs (m) when visible. The other Hoechst-stained nuclei are fragments from the old vegetative MAC. The grey to black color represents the intensity of H3K27me3 or H3K9me3 staining. The scale bar is 5 µm. See Figures S2 and S3 for the entire images and a description of the staining throughout the life cycle. **B**. Selected stack of the images shown above (A panel d). Scale bar is 5 µm.

Indirect immunostaining experiments were performed to determine the *in situ* localization of H3K27me3 and H3K9me3 during various stages of the *Paramecium* life cycle (Fig. S1-S2-S3). No H3K27me3 (Fig. S2) and H3K9me3 (Fig. S3) could be detected in the transcriptionally active MAC or in the transcriptionally inactive MIC during vegetative growth. The sexual process of autogamy (self-fertilization), which is induced by starvation, starts with meiosis of the MIC and proceeds through the development of new zygotic MACs. H3K27me3 was transiently found in the MIC during the first meiotic division and detected in the fragments of the maternal MAC by the end of meiosis (Fig. S2), whereas no H3K9me3 signal was observed at these stages (Fig. S3). After karyogamy, the diploid zygotic nucleus divides twice and two of the products differentiate into new MICs and the other two into new MACs (Fig. S4). H3K27me3 and H3K9me3 were detected at early stages of MAC development and the signals persisted throughout the course of MAC development (Fig. 1A, Fig. S2-S3-S4). The enrichment of H3K27me3 in the developing MAC compared to vegetative MAC was confirmed by Western blot analysis on purified nuclei (Fig. S2B). A Pgm-GFP fusion protein was detected together with H3K27me3 and H3K9me3 in the developing MAC, indicating that both histone marks are present when genome rearrangements occur (Fig. S2C and S3B).

The staining in the developing MAC, initially diffuse and evenly distributed (Fig. 1A a-b, Fig. S2-S3), gradually condensed into a punctuate pattern (Fig. 1A c-d, Fig. S2-S3). This is reminiscent of the heterochromatin bodies detected in *Tetrahymena*, which comprise H3K9me3, H3K27me3 [15], [16] and the chromodomain protein Pdd1p [23]. Yet the H3K27me3 and H3K9me3 foci we observed are located inside the nucleus and are not preferentially found at the periphery of the developing MAC as observed in *Tetrahymena*. As development proceeds, the number of these intensely labeled foci diminishes and the single remaining spot found in a DNA-poor region of the macronucleus eventually disappeared (Fig. 1B, Fig. S2-S3-S4).

### The domesticated transposase PiggyMac is required for H3K27me3 and H3K9me3 foci formation

**Figure 2:**
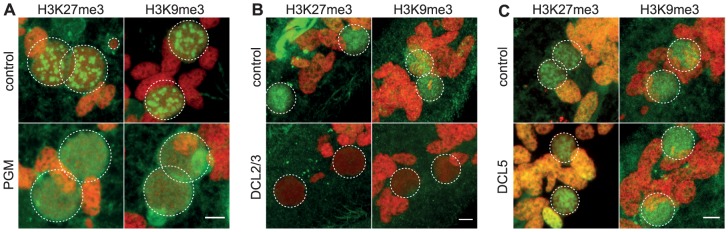
Depletion of the Pgm endonuclease and of the Dicer-Like 2 and 3 proteins alter H3K27me3 and H3K9 me3 localization. Immunolabeling with H3K27me3- or H3K9me3- antibodies (in green) and staining with Hoechst (in red) (A) in ND7 (control) or PGM KD cells at 30 hrs, (B) in ND7 (control) or DCL2/3 KD cells at 6 hrs, (C) in ND7 (control) or DCL5 KD cells at 6 hrs. See Figure S4 for progression of autogamy. Dashed white circles indicate the two developing MACs and the MICs when visible. Note that the H3K27me3 antibodies decorate the oral apparatus (“control” panel in Fig. 2B). Scale bar is 5 µm.

To obtain further insight into the possible role of H3K27me3 and H3K9me3 in genome rearrangements, we knocked-down by RNA interference (RNAi) the domesticated transposase Pgm that is required for the introduction of DNA-double strand breaks at the boundaries of germline-limited segments [2], [3]. Immunofluorescence experiments revealed that the H3K27me3 and H3K9me3 signals progressively increased in the developing MACs and are detected in all Pgm-depleted and control cells (Fig. 2A and Fig. S4). Western blot analysis showed that the amount of H3K27me3 is not altered in Pgm-depleted cells as compared to control cells (Fig. S2B). Since depletion of Pgm does not affect the biogenesis and accumulation of H3K27me3 and H3K9me3 in the developing MACs, it suggests that the endonuclease Pgm must act downstream of H3K27me3 and H3K9me3, in agreement with the scanning model.

We noticed that the H3K27me3 and H3K9me3 signals remained diffuse as development proceeds in Pgm-depleted cells and no foci could be detected (Fig. 2A and Fig. S4). The endonuclease Pgm is thus required for H3K27me3 and H3K9me3 foci formation, even though it is not yet clear whether these foci are a prerequisite for or the consequence of DNA double strand break formation.

### The scnRNA pathway, but not iesRNAs, is required for H3K27me3 and H3K9me3 accumulation in the early developing MAC

The scanning model posits that MIC-specific scnRNAs guide the loading of histone marks specifically on DNA segments that are eliminated in the developing MAC. We therefore expected that the co-silencing of the two Dicer-like genes, DCL2 and DCL3, that results in failure to generate scnRNAs [5], [6], would also abolish the establishment of H3K27me3 and H3K9me3 chromatin in the developing MAC. We examined the effects of DCL2/3 co-silencing on H3K27me3 and H3K9me3 by immunofluorescence staining. No detectable H3K27me3 or H3K9me3 signal was observed in Dcl2/3 depleted cells at an early stage when the developing MAC of control cells stained intensely (Fig. 2B, Fig. S4). As development proceeds, H3K27me3 and H3K9me3 signals in developing MACs start to be observed in Dcl2/3-depleted cells (Fig. S4) but Western blot analysis showed that the total amount of H3K27me3 is greatly reduced in DCL2/3-knockdowns (KD) relative to control (Fig. S2B).

We then investigated the effects of silencing DCL5, a gene required for iesRNA biogenesis, on H3K27me3 and H3K9me3. In contrast to what is observed in DCL2/3 KD, H3K27me3 and H3K9me3 signals were not altered in DCL5 KD, as assessed by immunofluorescence staining (Fig. 2C) and this was further confirmed by Western blot analysis for H3K27me3 (Fig. S2B).

We conclude that the generation of scnRNAs, but not iesRNAs, is required for establishment and accumulation of these chromatin modifications in the developing MAC. These results suggest that scnRNAs and K9 and K27 methylation participate in the same pathway leading to genome rearrangements. To support this hypothesis, it is necessary to demonstrate that K27 and K9 methylation is required for DNA elimination.

### Identification of SET-domain containing proteins and functional analysis of 5 EZL genes reveal that EZL1 is essential

**Figure 3:**
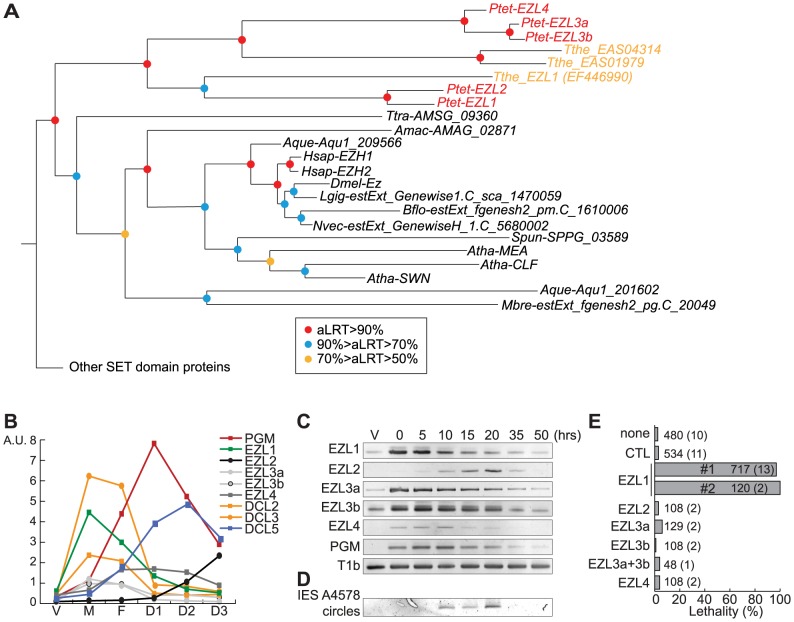
Identification and functional analysis of EZL genes. **A**. Phylogenetic analysis of the EZH/EZL SET domain proteins. The part of the Maximum-likelihood (ML) tree shown in Figure S5 and which includes the EZH/EZL proteins, is depicted. Statistical supports (aLRT values) are indicated on the nodes by colored circles (color code is indicated in the figure). Species abbreviations: *Amac* =  *Allomyces macrogynus* (Fungi); *Atha* =  *Arabidopsis thaliana* (Virdiplantae); *Aque* =  *Amphimedon queenslandica* (Metazoa); *Bflo* =  *Branchiostoma floridae* (Metazoa); *Dmel* =  *Drosophila melanogaster* (Metazoa); *Hsap* =  *Homo sapiens* (Metazoa); *Mbre* =  *Monosiga brevicolis* (choanoflagellata); *Nvec* =  *Nematostella* (Metazoa); *Ptet* =  *Paramecium tetraurelia* (Ciliata); *Spun* =  *Spizellomyces punctatus* (Fungi); *Tthe* =  *Tetrahymena thermophila* (Ciliata); *Ttra* =  *Thecamonas trahens* (Apusozoa). **B**. Expression patterns of EZL genes during the life cycle. EZL, DCL2, DCL3, DCL5 and PGM gene expression levels, as determined by microarray expression data during autogamy time-course experiments [26]. The vegetative time point (V) consists of 4 samples from mass cultures containing only log-phase cells showing no sign of meiosis. The meiosis time point (M) consists of 4 samples containing 20-39% of cells undergoing meiosis, and little or no fragmentation of the maternal MAC. The fragmentation (F) time point consists of 4 samples that contained a similar proportion of meiotic cells (20-29%) as the M time point, but also contained 37-43% of cells with a fragmented maternal MAC. The D1 time point groups 3 samples with 35-56% of cells with fragmented maternal MACs and 35-51% of cells that already contained clearly visible new MACs. D2 consists of 3 samples with 73-98% of cells with visible new MACs, and the D3 samples were taken ∼10 hours after the D2 samples. **C**. Detection of EZL and PGM mRNA during autogamy by RT-PCR. Total RNAs were extracted at each time point (see Fig. S7), were reverse transcribed and cDNAs were amplified by PCR with gene specific primers and, as a loading control, with primers for the T1b gene, which encodes a component of the secretory granules [55]. **D**. PCR detection of IES 51A4578 circles with divergent primers on genomic DNA at each time point shown in Figure S7 after ICL7 (control) or EZL1 silencing. **E**. Lethality of post-autogamous progeny following EZL gene silencing. The gene targeted in each silencing experiment is indicated. Two non-overlapping fragments (#1 and #2) of EZL1 gene were used independently. The ND7 or ICL7 genes were used as control (CTL) RNAi targets, since their silencing has no effect on sexual processes [27]. Autogamy was also performed in standard *K. pneumoniae* medium (none). Cells were starved in each medium to induce autogamy and, following 3-4 days of starvation, autogamous cells were transferred individually to *K. pneumoniae* medium to monitor growth of sexual progeny. The total number of autogamous cells analyzed for each RNAi and the number of independent experiments (in parenthesis) are indicated. Death in progeny after EZL1 silencing was observed after less than three cell divisions. The absence of lethality observed after EZL2, EZL3a, EZL3b, EZL4 KDs should be taken with caution as the level of KDs was not measured.

To eliminate K9 and K27 methylation, we sought to identify the gene(s) responsible for these modifications [24]. We searched for SET domain containing proteins encoded in the *P. tetraurelia* MAC genome [25]. Among 34 putative HMTs (Fig. S5, Table S1), we identified putative H3K27-specific HMTs of the Enhancer of zeste family, named EZL1 to EZL4 (Fig. 3A) but no member of the H3K9-specific HMTs of the Suvar39/EHMT/SETDB8/SETMAR group could be identified in ciliate genomes. Alignment of the predicted Ezl proteins revealed conservation of key residues implicated in binding the methyl donor, the target lysine, and catalysis (Fig. S6).

The expression patterns of EZL genes during the life cycle were examined using microarray data [26] and confirmed by RT–PCR analysis (Fig. 3B-C). Little or no expression is observed during vegetative growth but the genes are specifically expressed during the sexual phase of the life cycle, although they show markedly different patterns. EZL2 and EZL4 are silent during vegetative growth but EZL4 is specifically expressed after meiosis, whereas EZL2 becomes expressed at the onset of MAC development. EZL1 is turned on to high levels immediately upon meiosis, and this is true also, to a lesser extent, for EZL3a and EZL3b. Expression of the EZL1 gene is very transient, preceding PGM and DCL5 expression, and detection of IES excision products (Fig. 3D). This expression pattern is similar to that seen for the Dicer-like genes DCL2 and DCL3 [5](Fig. 3B).

To test the function of EZL genes, we knocked down their expression by RNAi during autogamy. After EZL1 KD, 97% of post-autogamous progeny were unable to resume vegetative growth, whereas no lethality was observed after KD of any other EZL gene (Fig. 3E). The transcription of EZL1 is induced during meiosis, largely before programmed genome rearrangements take place in developing new MACs. This pattern led us to consider the possibility that this protein may be involved in a meiotic function. We checked the progression of meiosis by Hoechst staining during autogamy of EZL1 KD cells. We observed that meiotic divisions I and II occur normally, since there were cells with 4 then 8 haploid nuclei in the population (Fig. S4). There was no arrest until new MACs differentiate from mitotic copies of the zygotic nucleus. To control for possible off-target silencing artifacts, two non-overlapping fragments of EZL1 were used independently to induce RNAi, and similar results were obtained with both constructs (Fig. 3E). For one construct, the efficiency of EZL1 KD was checked by semi-quantitative RT-PCR of total RNA extracted throughout autogamy from control and EZL1 KD cells (Fig. S7): a significant decrease of EZL1 mRNA accumulation was observed at early time points in EZL1 KD cells, without affecting the onset of induction of other EZL genes. Therefore, EZL1 gene expression is essential during development for the production of viable sexual progeny.

In an EZL1 KD, the transcription of the PGM gene and of all EZL genes is switched on normally during autogamy, indicating that these genes are not induced in response to EZL1 induction but more likely as part of a general transcription program during MAC development. In contrast to control cells, the levels of these mRNAs do not decrease at later time-points in an EZL1 silencing experiment (Fig. S7), suggesting that the completion of MAC development is a signal for transcriptional switch-off. Alternatively, EZL1 histone methylation could be required for silencing transcription of these genes.

### The Ezl1 protein is required for H3K27me3 and H3K9me3 in the developing MAC

**Figure 4:**
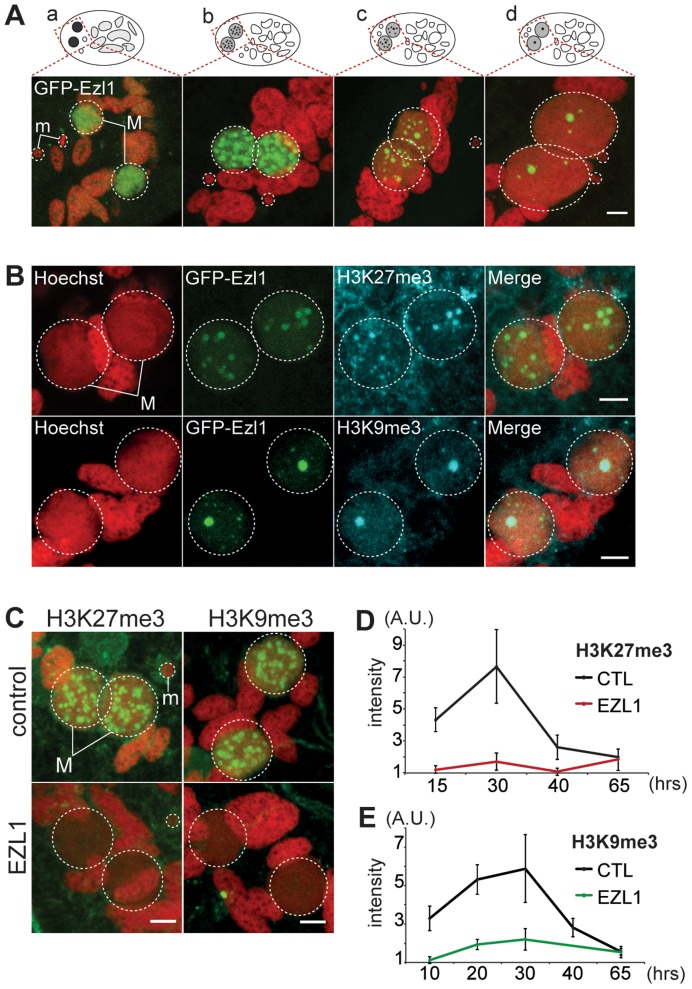
EZL1 is required for H3K27me3 and H3K9me3 in the developing somatic MAC. **A.** GFP-EZL1 localization in the developing new somatic MAC. Overlay of Z-projections of magnified views of GFP-EZL1 (in green) and Hoechst (in red) on selected stacks at different stages of development of the somatic MAC (a-d) are presented. See Figure S8 for the entire images and a description of the staining throughout the life cycle. Dashed white circles indicate the two developing MACs (M) and the MICs (m) when visible. The other Hoechst-stained nuclei are fragments from the old vegetative MAC. The grey to black color represents the GFP-EZL1 intensity. Scale bar is 5 µm. **B**. Co-localization of GFP-EZL1 fusion protein and H3K27me3 or H3K9me3. GFP-EZL1 transformed cells were immunolabeled with H3K27me3- or H3K9me3- antibodies and stained with Hoechst at 40 hrs during autogamy. Overlay of Z-projections of magnified views of GFP-EZL1 (in green), H3K27me3- or H3K9me3- specific antibodies (in blue), and Hoechst (in red) on selected stacks are presented. Dashed white circles indicate the two developing MACs (M). Scale bar is 5 µm. **C**. Immunolabeling with H3K27me3- or H3K9me3- antibodies (in green) and staining with Hoechst (in red) in ND7 (control) or EZL1 knockdown cells at 30 hrs. See Figure S4 for progression of autogamy and quantification of the number of stained cells at each time point. Dashed white circles indicate the two developing MACs (M) and the MICs (m) when visible. Scale bar is 5 µm. **D**. Quantification of H3K27me3 or H3K9me3 fluorescence intensities in the developing MACs. At each time point, the mean of fluorescence intensities was calculated for ten individual cells after control or EZL1 silencing (see Materials and Methods). Errors bars represent the standard deviation.

To gain further insight into the role of EZL1, we examined the subcellular localization of Ezl1. A GFP fusion was constructed by inserting the GFP coding sequence into the EZL1 gene, downstream of the start codon. Expression of the fusion gene was under the control of the natural EZL1 up- and downstream sequences. After microinjection of the construct into the MAC, no fluorescence could be detected during vegetative growth of transformed clones (Fig. S8A). When autogamy was induced, GFP fluorescence first appeared transiently in the MIC during meiosis I and in the MAC before it became fragmented. When fragmentation of the maternal MAC was complete, GFP fluorescence started to decrease and progressively relocalized to the new MACs as they developed (Fig. 4A and Fig. S8). Eventually all of the fusion protein was concentrated to the new MACs. The localization pattern of GFP-Ezl1 fusion is very similar to that observed for H3K27me3 and H3K9me3 (Fig. 1 and Fig. S2-S3). Hence, the GFP-Ezl1 fusion colocalized with H3K27me3 and H3K9me3 foci in the new developing MACs (Fig. 4B). Moreover, although the GFP-Ezl1 fusion protein properly localized in the new developing MACs in PGM and DCL2/3 KD cells, foci formation was prevented in the former and strongly reduced in the latter (Fig. S8B).

We therefore investigated the effects of EZL1 KD on H3K27me3 and H3K9me3. Immunofluorescence staining with H3K27me3- and H3K9me3- specific antibodies showed little or no signal in Ezl1-depleted cells, whereas in control cells H3K27me3 and H3K9me3 increased as development proceeded and completely disappeared at the latest time point (Fig. 4C and Fig. S4). For more accuracy, we quantified the fluorescence intensities throughout the volume of the developing new MACs in control and EZL1 KD cells at different developmental time points (see Materials and Methods). The quantification indicated that H3K27 and H3K9 methylation was nearly abolished in the developing new MACs of EZL1 KD cells (Fig. 4D-E). This was further confirmed for H3K27me3 by Western blot analysis (Fig. S2B). Together these data show that EZL1 encodes a development specific putative HMT necessary for H3K27me3 and H3K9me3 in the developing zygotic MAC.

### The Ezl1 protein is required for imprecise deletion of MIC-specific sequences

EZL1 KD led to phenotypes consistent with an essential function for Ezl1 during MAC development since: (1) no viable sexual progeny were isolated from Ezl1-depleted cells, a phenotype described in KDs defective in DNA elimination [3], [5], [27]–[29]; (2) no H3K27me3 and H3K9me3 were detected in developing new MACs.

**Figure 5:**
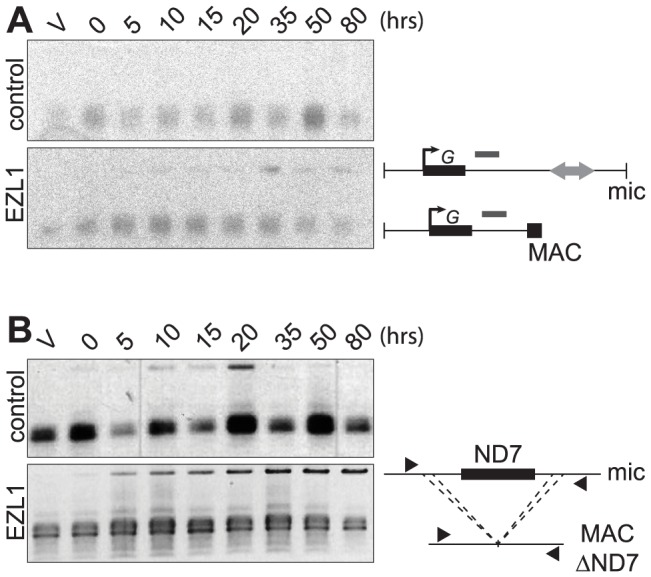
EZL1 is required for imprecise DNA elimination. **A**. Fragmentation of germline DNA downstream of the G51 gene is analyzed by Southern blot hybridization of PstI-digested total genomic DNA run on an 0.8% agarose gel after ICL7 (control) or EZL1 KD. ICL7 is a non essential gene that encodes an infraciliary lattice centrin [56]. The subtelomeric tel51G probe [3] is shown as a grey rectangle above the line. The black square represents telomeric repeats of the MAC chromosome. **B**. Maternal inheritance of macronuclear deletion is analyzed after ICL7 (control) or EZL1 RNAi, using a cell line reproducibly deleting the ND7 gene from the MAC genome at each sexual generation. PCR analysis was performed on the same DNA samples as in (A) with primers (black arrows) located upstream and downstream of the ND7 open reading frame.

Different assays were used to monitor genome rearrangements in autogamy time course experiments, after EZL1 KD. We first tested the role of the EZL1 gene in the imprecise DNA elimination mechanism that is responsible for the deletion of MIC transposable elements during MAC development. We analyzed by Southern blot hybridization the germline region located downstream of the surface antigen G gene, which contains a Sardine transposon that is eliminated imprecisely during MAC development, leading to chromosome fragmentation [2]. At this locus, in control RNAi experiments, only the rearranged forms originating from both the maternal and new MACs could be detected (Fig. 5A). In contrast, after EZL1 KD, non-rearranged forms accumulated throughout autogamy in the new MACs, relative to the rearranged forms present in the fragments of the maternal MAC (Fig. 5A). EZL1 KD thus led to retention of the MIC sequences and impaired germline chromosome fragmentation.

We further tested the role of Ezl1p in the imprecise elimination mechanism also responsible for maternally inherited deletions of non-essential cellular genes, which can be induced experimentally [12]. The variant cell line 51ΔND7 has a wild type MIC but carries a maternally inherited MAC deletion of the ND7 gene. We therefore used this strain to monitor the effect of EZL1 KD on maternal inheritance of MAC deletions. Phenotypic testing was used to assess reversion of the ND7 MAC deletion in post-autogamous cell populations. We observed the occurrence of trichocyst discharge indicating that the ND7 gene was at least partially maintained in the new MAC after EZL1 KD but not in the controls. ND7 was transiently amplified before deletion from the new developing MACs and, at later time points, only the rearranged forms, originating from both the old and new MACs, could be detected in control silencing (Fig. 5B). In contrast, full-length ND7 gene product accumulated at late time points after EZL1 KD. Thus, like imprecise deletion of MIC specific regions, maternally inherited elimination of the ND7 gene is blocked in EZL1 KD cells, and the non-rearranged germline locus is retained in the developing new MACs.

To expand these results genome wide, we sequenced DNA isolated from newly developed MACs following EZL1 silencing. DNA isolated from newly developed MACs at the same developmental stage from a cell culture grown without RNAi was also sequenced as a control (Table S2). We compared the sequence complexity of different datasets by mapping the reads in each dataset to contigs previously assembled from new MACs after Pgm depletion [2], which is currently the best representation of the un-rearranged germline genome. As shown in Table S3, EZL1 reads have the same sequence complexity as the PGM reads, while the control dataset has about 13 Mb less sequence complexity. Of note, the total sequence complexity in the MIC is expected to be larger than the 89 Mb that we analyzed since our analysis only used PGM contigs larger than 1 kb.

If we compare the complexity of regions not covered by the control sample, which correspond to the part of the MIC genome that is not collinear with MAC chromosomes, PGM and EZL1 datasets again show a similar complexity. We also performed a qualitative evaluation of Sardine retention by mapping reads from each dataset to the known cloned copies of this transposable element [2](Fig. S9). Consistent with our Southern blot analysis (Fig. 5A), we found that all characterized Sardine copies are retained after EZL1 silencing. This global analysis supports the conclusions made at the molecular level for two individual loci: EZL1, like PGM, is required for the imprecise elimination of germline-limited sequences.

### The Ezl1 protein is required for the excision of a subset of IESs

We then investigated the role of the EZL1 gene in IES excision. Excision was first analyzed by PCR on genomic DNA, extracted after EZL1 or control silencing at a time when IES excision is normally finished. In control RNAi experiments, the 10 IESs analyzed were completely excised from the new developing MACs as expected (Fig. S10). In contrast, IES-retaining forms accumulated in the new MACs of PGM or EZL1 KD cells. We observed that EZL1 KD impaired the excision of 7 out of 10 tested IESs, whereas PGM KD impaired the excision of all IESs (Fig. S10). Consistent with the lack of excision for affected IESs, we could not detect the formation of excised IES circles by PCR upon EZL1 KD (Fig. S7)[30]. Altogether these data indicate that the EZL1 gene is required for IES excision and, most likely, EZL1p acts upstream of the introduction of DNA double-strand breaks.

**Figure 6:**
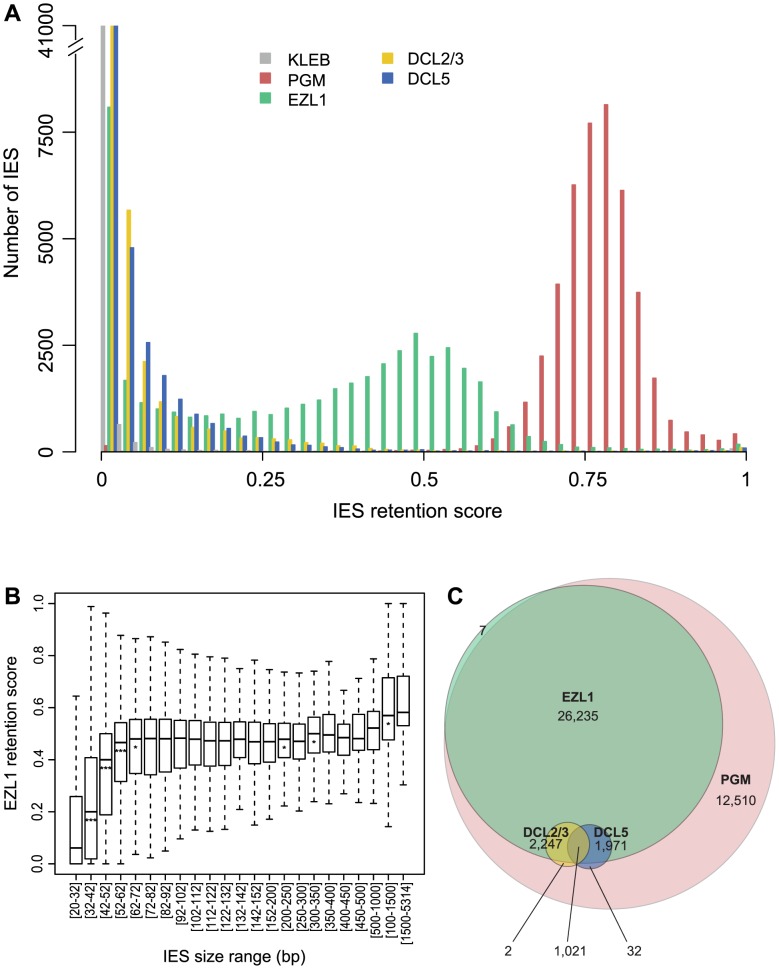
Genome-wide effects of EZL1, DCL2/3 or DCL5 KD on IES excision. **A**. IES retention scores after PGM, EZL1, DCL2/3, or DCL5 KD. Histograms of IES retention scores, as defined in Materials and Methods, for control (grey), PGM (red), EZL1 (green) and DCL5 (blue) silencing and DCL2/3 co-silencing (yellow) datasets. **B**. EZL1 retention scores display a strong bias for IES size. IESs were grouped by size, each group corresponding to a peak in the genome-wide IES size distribution [2]. Each box summarizes the EZL1 retention score distribution for the group. The median EZL1 retention score (horizontal line inside the box) and the first (top of box) and third (bottom of box) quantiles are shown. Stars beneath the median value of a group indicate that the retention score distribution of that group is significantly different from the retention score distribution of the previous group. One star, p<0.05, two stars, p<1e-10, three stars, p<2.2e-16. The groups contain: 15,857; 485; 6,354; 3,514; 3,108; 3,642; 2,459; 1,934; 1,532; 1,111; 795; 594; 410; 1,176; 780; 398; 198; 89; 64; 47; 183; 63; 135 IESs, respectively. **C**. Venn diagram of significantly retained IESs after PGM, EZL1, DCL2/3 or DCL5 silencing.

Based on our PCR analyses, not all IESs are affected following EZL1 KD. To observe the effects of Ezl1p depletion genome-wide, we used the EZL1 DNA-seq dataset. A retention score (RS) was calculated for each IES in the reference set [2]: reads that map to IES ends were classified as IES-containing or as MAC junction-containing reads, representing retained and excised IESs respectively. The RS is the ratio of IES-containing to total classified reads, and RS values vary from 0 for no IES retention to 1 for complete IES retention. As expected (Fig. 6A), the RS distribution of the control dataset is close to 0 (mean 0.008), whereas a Gaussian distribution was observed for the PGM dataset [2] with a mean RS of 0.77. Even if Pgm is responsible for complete excision of all IESs [2], [3], the mean RS never reaches 1 owing to the presence of rearranged DNA in the PGM sample coming from the fragments of the maternal MAC still present in the cytoplasm. Consistent with previous work [2], excision of all IESs appears to be affected in a similar manner following PGM KD. In the EZL1 dataset however, the mean RS is 0.32 and the distribution is bimodal with 8,085 IESs that have an RS close to 0, the rest of the IESs displaying a wide distribution of retention scores (mean 0.39) with a mode of 0.5 (Fig. 6A).

We used a statistical test (see Materials and Methods) to compare the retention scores in the EZL1 or PGM datasets to the retention scores in the control dataset in order to identify significantly retained IESs. In the PGM dataset, 44,028 IESs (97.9%) are significantly retained compared to control and in the EZL1 dataset, 31,481 IESs (70.1%) are significantly retained with a mean RS of 0.42. A biological replicate from an independent EZL1 silencing experiment showed good correlation of retention scores (Spearman correlation coefficient: 0.887, p< 2.2 10^−16^). Based on these data, we can define two classes of IESs: those that are significantly retained after EZL1 KD and those that are not. Importantly, our PCR analyses are completely coherent with our genome-wide analysis (Table S4).

We then wondered what distinguishes the two classes of IESs. Our PCR analyses indicated that long IESs were retained in the new developing MAC following EZL1 KD (Fig. S10 and Table S4). To confirm this observation genome-wide, IESs were grouped according to their size [2] and the distribution of retention scores for each group represented in a box plot (Fig. 6B). The IES were grouped as previously described to follow the periodic distribution of IES size with peaks every ten base pairs [2]. The first 5 groups (26-72 bp) have retention score distributions that are significantly different from each other: the larger the IES size, the higher the retention score. Starting with the 5^th^ group (>72 bp), the median does not change much, which indicates that IESs of these sizes are similarly affected by Ezl1p depletion. For the largest IESs (>1 kb), the retention score distribution is significantly shifted to higher values. Among those, there is one group composed of 28 IESs, which have been shown to derive from Tc1/mariner TEs named Anchois [2]. All of them are retained after EZL1 inactivation (Table S5). Roughly 50% of IESs are over 52 bp in length and among them, 89.9% are significantly retained after EZL1 KD, while only 40% of the IESs shorter than 52 bp in length are significantly retained. This robust correlation between IES size and retention score is not observed for the PGM dataset (Fig. S11), indicating that it is a property specific to Ezl1p depletion.

We searched for features other than size that could be associated with EZL1 retained IESs. We compared IESs of the same size (26-32 bp) and did not find any meaningful correlation for a large number of criteria, including the consensus present at IES ends and the scnRNA density on IESs. We did observe that EZL1-retained IESs have: (i) a slightly higher GC content, (ii) a more frequent location within gene coding sequences (Fig. S12). It is intriguing that these two properties can also be important determinants in nucleosome positioning [31]–[33].

### The Dcl2 and Dcl3 proteins are required for the excision of a subset of IESs that are highly sensitive to Ezl1 depletion

Since DCL2 and DCL3 genes, like EZL1, are required for establishing H3K27me3 and H3K9me3 in the developing MAC (Fig. 2 and 4), we hypothesized that depletion of Dcl2 and Dcl3 proteins would impair DNA elimination in a similar manner to that of Ezl1 depletion. To address this question, we sequenced DNA isolated from newly developed MACs following DCL2/3 co-silencing. When compared to the PGM and EZL1 datasets, the total sequence complexity was similar in the DCL2/3 dataset (Table S3) and analysis of Sardine retention showed that all characterized Sardine copies are retained following DCL2/3 co-silencing (Fig. S9). This global analysis confirmed that the Dcl2 and Dcl3 proteins are required for the imprecise elimination of germline-limited sequences [5].

Analysis of the effects of DCL2/3 KD on IES excision led to a surprising finding. Compared to PGM or EZL1 silencing, most IESs are weakly or not at all retained after DCL2/3 co-silencing (Fig. 6A). Only 3,272 IESs (7.3%) are significantly retained in the DCL2/3 dataset with a mean RS of 0.24. The small number of significantly retained IESs and their low RS might be explained in part by incomplete silencing. Yet, the possibility that there are still low amounts of Dcl2 and Dcl3 proteins that would provide sufficient scnRNAs for IES excision is unlikely because very little if any scnRNAs can be detected in typical DCL2/3 KDs [5], [6]. Moreover, we found 3,160 IESs significantly retained for a biological replicate [6] and a good correlation of RS was observed for the two biological replicates (Spearman correlation coefficient 0.616, p< 2.2 10^−16^) despite use of different silencing constructs. Furthermore, the RS measured for the DCL2/3 dataset are in agreement with our PCR analyses (Fig. S10 and Table S4). Based on our PCR analyses, we noticed that all mcIESs are significantly retained in the DCL2/3 dataset and that all IESs retained after DCL2/3 KD are retained in the EZL1 dataset. The latter was confirmed genome-wide: almost all significantly retained IESs in the DCL2/3 dataset are significantly retained in the EZL1 dataset (3,269/3,272) (Fig. 6C). Furthermore, IESs retained upon DCL2/3 KD are among the most retained IESs in the EZL1 dataset (Fig. S13). Only the largest IESs are retained in the DCL2/3 dataset; 50% of the IESs larger than 1 kb are significantly retained (Fig. S11). Among those, 19/28 Anchois IESs are significantly retained after DCL2/3 KD (Table S5). Altogether our data indicate that IESs retained upon DCL2/3 KD correspond to a small subset of EZL1 retained IESs (Fig. 6C).

### The Dcl5 protein does not play a major role in the imprecise deletion of MIC-specific sequences and affects the excision of only a subset of IESs that are sensitive to Ezl1p depletion

The Dicer-like protein Dcl5 was reported to be required for efficient excision of at least a fraction of IESs [6]. We therefore compared the effects of Dcl5 depletion on DNA elimination, using the previously published DCL5 dataset [6] (Table S2), to those observed after Ezl1 depletion. Compared to the PGM and EZL1 datasets, the total sequence complexity was much lower in the DCL5 dataset (Table S3) and analysis of Sardine transposon retention showed that none of the characterized Sardine copies are retained following DCL5 silencing (Fig. S9). This global analysis indicates that, in contrast to Pgm, Ezl1 or Dcl2/3 proteins, the Dcl5 protein is unlikely to play a major role in the imprecise elimination of germline-limited sequences.

We then measured the retention score for each IES using our criteria for statistical significance (see Materials and Methods) and, consistent with previous work [6], most IESs are weakly or not at all retained after DCL5 silencing (Fig. 6A). Only 3,024 IESs (6.7%) are significantly retained in the DCL5 dataset with a mean RS of 0.21. Almost all significantly retained IESs in the DCL5 dataset are strongly retained in the EZL1 dataset (Fig. 6C). IESs retained upon DCL5 KD correspond to a small subset of EZL1 retained IESs, which is furthermore different than the subset of IESs retained upon DCL2/3 KD.

## Discussion

H3K27 and H3K9 tri-methylation and the histone methyltransferase Ezl1 appear to participate in the regulatory pathway that controls developmental genome rearrangements in *Paramecium*. Indeed, H3K27me3 and H3K9me3 appear transiently in the developing somatic macronucleus when genome rearrangements occur, and, as in *Tetrahymena*, the EZL1 gene is required for correct genome rearrangements and encodes a development specific putative HMT necessary for H3K27me3 and H3K9me3 in the developing new MAC.

### scnRNA-mediated H3K27 and H3K9 methylation is required for deletion of transposable elements

**Figure 7:**
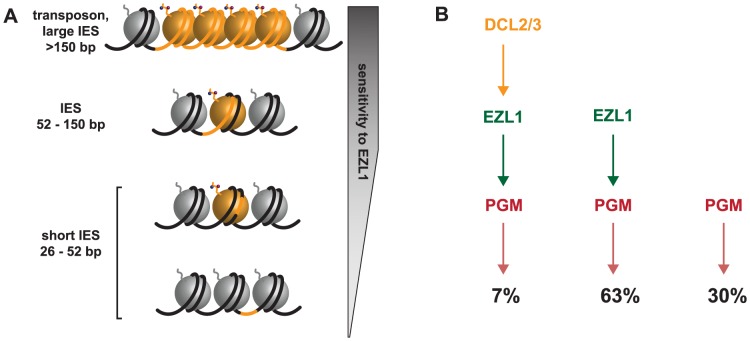
Model for the action of the histone methyltransferase Ezl1 in programmed genome rearrangements and schematic representation of partially overlapping pathways involved in IES excision. **A.** To take into account the length bias of DNA sequences retained after Ezl1 depletion, we propose that efficient excision of germline-limited DNA segments (orange) is regulated by the position of methylated nucleosomes. For long DNA segments (>150 bp in length) that are covered by at least one nucleosome, the putative HMT Ezl1 would be targeted to the eliminated sequences and would catalyze the trimethylation of H3 K27 and K9. These histone marks would attract, or activate, the excision machinery, thereby the excision of marked DNA segments. DNA segments whose size is comprised between 52 bp and 150 bp would be partly or entirely included within one nucleosome and histone H3 methylation would be essential for their recognition and excision. The smallest DNA segments (26-52 bp in length), however, would be either wrapped around a nucleosome and histone methylation might be needed for efficient excision, or located within the linker DNA and histone methylation might be dispensable for their excision. **B.** Schematic representation of partially overlapping pathways involved in IES excision. While all IESs are ultimately excised by the Pgm endonuclease, there appears to be different classes of IESs. Some IESs (30%) require neither EZL1 nor DCL2/3 for complete excision; others (7%) require EZL1 and DCL2/3; while the majority require only EZL1 (66%).

The work presented here demonstrates that the putative HMT Ezl1 is required for the elimination of transposable elements, of their more recent relics in the form of long IESs and of germline DNA regions that encompass several kb in length, which might altogether represent at least 25% of the germline genome. We have shown that H3K27me3 and H3K9me3 signals are abolished after Ezl1 depletion and that scnRNAs are necessary for the deposition of these histone marks in the developing somatic MAC. We also provide evidence that the Dcl5 protein necessary for iesRNA biogenesis does not play a major role in the elimination of transposable elements. Therefore, our results support the idea that scnRNAs guide the putative HMT Ezl1 to specific germline sequences in the developing somatic macronucleus. Consistent with the idea that the Ezl1 protein acts downstream of scnRNAs, analysis of small RNA sequencing datasets showed that scnRNA biogenesis is not affected upon Ezl1 depletion as compared to control RNAi or wild type (A. de Vanssay and O. Arnaiz, personal communication). Deposition of H3K27me3 and H3K9me3 would allow the recruitment, or activation of the excision machinery, followed by elimination of marked DNA segments (Fig. 7A), consistent with our observations that the Ezl1 protein acts upstream of the Pgm endonuclease. Our study provides evidence that RNAi-mediated heterochromatin formation is necessary for elimination of germline DNA in *Paramecium*, as is the case in *Tetrahymena* [1]. Ciliates use a similar, sRNA-dependent mechanisms for heterochromatin formation as other eukaryotes [20], [21], except that it goes a step further with the physical elimination of the targeted sequences during development of the somatic nucleus. Very much like metazoan piRNAs, the scnRNA pathway controls the silencing of ‘genomic parasites’ such as TEs, thereby ensuring the integrity of the genome [34], [35].

### Short, dispersed, single copy germline sequences display differential sensitivity to depletion of the scnRNA pathway, Dcl5 or Ezl1 proteins

While all IESs are ultimately excised by the Pgm endonuclease [2], IESs appear to differ in their recognition mechanism. Only about a third of IESs (5 out of 13 tested), called mcIESs, are sensitive to the presence of homologous sequences in the maternal MAC [11]. Interestingly, genome-wide analyses of the effects of depletion of Dicer-like 2 and 3 proteins showed that they are both required for excision of Tc1/mariner TEs and of mcIES, but not of non-mcIESs ([6] and this study). The evidence obtained so far is consistent with the idea that Dcl2/3 retained IESs correspond to mcIESs, but unfortunately, it is not possible to experimentally determine the genome-wide set of mcIESs. Surprisingly however, only a small fraction of IESs (less than 10%) are retained after depletion of the Dicer-like 2 and 3 proteins. Even though it remains possible that we underestimate the total number of IESs retained in DCL2/3 KD either for technical reasons or because the ablation of the scnRNA pathway is compensated by another unidentified small RNA pathway, our data indicate that the fraction of mcIESs in the genome might be smaller than initially thought.

More importantly, our data indicate that most IESs are correctly excised in the absence of scnRNAs. IESs, even those that are not under maternal control, normally do produce scnRNAs during MIC meiosis [5], [6], [13] and, when introduced into the maternal MAC, give rise to non-coding transcripts like any other sequence [7], suggesting that the genome scanning process should inactivate their scnRNAs. Our conclusion is thus that excision of non-mcIESs simply does not depend on scnRNAs. None of the shortest (∼28 bp) IESs tested, which are also the oldest [2], was found to be a mcIES, raising the possibility that non-mcIES represent the endpoint of IES evolution. In support of this view, our data indicate that recognizable TEs and young (longer) IESs display higher retention scores after depletion of Dicer-like 2 and 3 proteins, indicating that they indeed depend on their own scnRNAs for recognition and elimination.

In addition to these two classes of IESs, genome wide analysis of the effects of Ezl1 depletion provided evidence for additional classes of IESs, showing differential sensitivity to the different factors studied here. In order to group IESs into functionally similar classes, we have quantitated the requirement of each of the 45,000 IESs for each of the factors analyzed (Fig. 6C). Our data show that a large fraction of IESs are retained after Ezl1 depletion. One surprising finding is that the set of EZL1 retained IESs is not the same as the set of IESs retained upon DCL2/3 KD. We also showed that IESs retained upon DCL5 KD correspond to a small subset of EZL1-retained IESs, which does not correspond to IESs retained upon DCL2/3 KD. Even though IESs retained after DCL2/3 KD or after DCL5 KD are all included in EZL1 retained IESs, our results argue that EZL1 is necessary for correct excision of most IESs, without the need of scnRNAs or iesRNAs. Because the excision of IESs, whether they are maternally controlled or not, EZL1 sensitive or not, is still dependent on the Ptiwi01/09 proteins [27], it remains possible that these proteins may be alternatively loaded with a different type of small RNA.

EZL1 encodes a putative histone methyltransferase necessary for H3K27me3 and H3K9me3 and for excision of about 70% of IESs, suggesting that H3K27me3 and H3K9me3 are required for their excision, as discussed below. DCL2/3 KD also leads to diminution of H3K27me3 and H3K9me3 signals at early stages of MAC development (Fig. 2) and yet, only approximately 10% of IESs are retained after DCL2/3 KD. The excision of IESs in DCL2/3 KD might be explained by H3K27me3 and H3K9me3 detected at late stages of MAC development in DCL2/3 KD, but not in EZL1 KD (Fig. S4). The low amount of H3K27me3 detected by Western blot appears to be compatible with the fact that IESs cover 3.2 Mb and thus represent about 3% of the sequence complexity of the MIC genome [2]. Yet we cannot formally exclude the possibility that the Ezl1 protein has an additional role in DNA elimination independently of histone H3 methylation. One can imagine, for instance, that the Ezl1 protein is also necessary for methylation of lysine residues within proteins involved in DNA elimination.

We now understand that IES excision involves partially overlapping pathways given our observations of different classes of IESs. This led us rethink the simple model according to which scnRNAs -produced by the Dcl2/3 proteins- lead to the loading of chromatin modifications- H3K27me3/HK9me3 through the action of the putative histone methyltransferase Ezl1- and recruitment of the Pgm endonuclease. Indeed, a small subset of IESs require DCL2/3 and EZL1 (7%), while the majority of IESs require only EZL1 (63%), and some IESs require neither EZL1 nor DCL2/3 for complete excision (30%) (Fig. 7B). The relative position of the Dcl5 protein in this process is not yet clear. Whether the existence of overlapping pathways reflects distinct protein complexes, complexes containing some different components, nucleosome positioning and/or unidentified determinants remains to be investigated. Future studies combining genetic and biochemical approaches will be necessary to first describe and then determine the functional significance of the amazing level of complexity that is beginning to emerge.

### Local effect of the putative histone methyltransferase Ezl1

In *Drosophila* and in mammals, Enhancer of zeste proteins are catalytic subunits of Polycomb complexes, which target H3K27me3 and maintain repression of numerous developmental genes. Domains enriched in H3K27me3 cover large regions of the genome, usually exceeding 10 kb [36]. One unexpected finding of our study is the putative HMT Ezl1p is required for the excision of very short DNA segments, as Ezl1 depletion leads to retention of 31,481 IESs (70,1%). Since the vast majority of IESs are shorter than 150 bp in length, the Ezl1 protein might not necessarily trigger the formation of heterochromatin on eliminated sequences. Instead, we imagine that Ezl1 acts locally and is responsible for trimethylation on lysines 27 and 9 on one or a few nucleosomes that overlap with the IES. The quantitative genome-wide analysis of IES retention showed that all IESs are not equal: IESs are retained to a different extent after Ezl1 depletion and we could not identify any features in the IES sequences that distinguish IESs that are significantly retained from those that are not. However, our analysis revealed a strong correlation between IES size and retention score, as 90% of IESs longer than 52 bp are retained after Ezl1 depletion. We propose that the excision process is regulated by the presence of methylated nucleosomes and depends on the relative positions of IES ends with respect to the methylated nucleosomes. As illustrated in Figure 7A, the positioning of nucleosomes might play a major role in IES excision. We reasoned that longer sequences have a higher probability to be associated with modified nucleosomes and would thus be more sensitive to Ezl1 depletion and loss of methylated H3. Any IES over 52 bp in length would be either entirely or partially covered by one nucleosome. This might reflect the length of the linker DNA in the developing somatic MAC, which is not known, but would be consistent with linker ranging from 20 to 90 bp in general [37]. Strikingly, small IESs between 26 and 52 bp in length have a wide range of retention scores. 34% of the smallest IESs (26-32 bp) required Ezl1 to be excised and we imagine those small IESs are either within, or partially covered by, one modified nucleosome. IESs that are not retained after Ezl1 depletion might be located in the linker region between nucleosome core particles or in nucleosome-free regions and alternative mechanisms would ensure their correct and precise excision. In *S. cerevisiae*, the chromatin remodeler SWR1 binds *in vitro* long nucleosome-free DNA and the adjoining nucleosome core particle, allowing discrimination of gene promoters over gene bodies. SWR1 binding is enhanced on acetylated nucleosomes, but recognition of nucleosome-free and nucleosomal DNA is dominant over interaction with acetylated histones [38]. Such hierarchical cooperation between DNA and posttranslational histone modifications might participate in guiding the excision machinery. Precise mapping of nucleosomes and of histone marks along the genome will be needed to explore this possibility. An exciting challenge for the future is to understand the mechanisms by which histone modifications position the excision machinery for precise DNA cleavage.

## Materials and Methods

### *Paramecium* strains, cultivation and autogamy

Unless otherwise stated, all experiments were carried out with the entirely homozygous strain 51 of *P. tetraurelia*. Cells were grown in wheat grass powder (WGP) (Pines International) infusion medium bacterized the day before use with *Klebsiella pneumoniae*, unless otherwise stated, and supplemented with 0.8 mg/mL β-sitosterol (Merck). Cultivation and autogamy were carried out at 27°C as described [39], [40].

### Phylogenetic analyses

SET domain proteins were retrieved using Pfam [41] and BLAST [42]. Multiple alignments were performed with MUSCLE 3.8 [43] and were subsequently manually improved. Maximum likelihood (ML) analyses were performed with PHYML [44] using the PHYML web server [45] hosted at the Montpellier bioinformatics platform (http://www.atgc-montpellier.fr/phyml/). PHYML analyses were performed using the Le and Gascuel (LG) amino-acid substitution model [46], using two rate categories (one constant and four γ rates). Statistical support for the different internal branches was assessed by approximate Likelihood-ratio test (aLRT; [47]).

### Gene silencing experiments

Plasmids used for T7Pol-driven dsRNA production in silencing experiments were obtained by cloning PCR products from each gene using plasmid L4440 and *Escherichia coli* strain HT115 DE3, as previously described [48]. Sequences used for silencing of EZL2, EZL3a, EZL3b, EZL4, DCL5 were segments 955-1519; 1402–1980; 1404–1982; 1398-1976 and 4-1998 of GSPATG00032888001; GSPATG00012695001; GSPATG00013305001; PTETG1700020001, GSPATG00003051001 [49], respectively. For EZL1 silencing, two non-overlapping gene fragments covering positions 991-1500 (EZL1-1) and 332-754 (EZL1-2) of GSPATG00037872001 were used. The fragments used for ND7 [12], ICL7a [27], DCL2, DCL3 [5] and PGM-1 [3] are those previously published. Preparation of silencing medium and RNAi during autogamy were performed as described in [3]. Lethality of post-autogamous cells after double silencing of DCL2 and DCL3 or silencing of PGM was 90-100% (30–60 cells were checked in each silencing experiment). As expected, Pgm depletion led to retention of all tested germline-limited elements in the developing MAC genome, while Dcl2/3 depletion led to retention of well-characterized IESs (Fig. S10) and Dcl5 depletion led to partial impairment of excision for IESs retained in the DCL5 dataset.

### Injection of GFP fusion transgenes

For the construction of in-frame GFP-EZL1 fusion, a GFP-coding fragment adapted to *Paramecium* codon usage [28] was added by PCR fusion to the 5′ end of the EZL1 gene. As a result, the GFP is fused to the N-terminus of EZL1 and the fusion protein is expressed under the control of the EZL1 transcription signals (promoter and 3′UTR). It contains the 830-bp genomic region upstream of the EZL1 open reading frame, the 304-bp genomic region downstream.

Plasmids carrying the GFP-EZL1 or PGM-GFP [3] fusion transgenes were linearized by XmnI or AflIII, respectively, and microinjected into the MAC of vegetative 51 cells. No lethality was observed in the post-autogamous progeny of injected cells, indicating that the GFP-EZL1 and PGM-GFP fusions did not interfere with normal progression of autogamy.

### DNA and RNA extraction, Southern blot, RT-PCR and PCR

DNA samples were typically extracted from 200-400-ml cultures of exponentially growing cells at <1,000 cells/ml or of autogamous cells at 2,000–4,000 cells/ml as previously described [40]. Small-scale DNA samples were prepared from ≤1,000 cells using the NucleoSpin Tissue kit (Macherey-Nagel). Electrophoresis and blotting were carried out according to standard procedures.

RNA samples were typically extracted from 200–400-ml cultures of exponentially growing cells at <1,000 cells/ml or of autogamous cells at 2,000–4,000 cells/ml as previously described [40]. RNA samples were reverse-transcribed with RevertAid H Minus Reverse Transcriptase (Thermo Scientific) using polydT primers (Thermo Scientific) according to the manufacturer's instructions. It was then followed by PCR amplifications in a final volume of 25 µL, with 10 pmol of each primer, 10 nmol of each dNTP and 2 U of DyNAzyme II DNA polymerase (Thermo Scientific).

PCR amplifications were performed in a final volume of 25 µL, with 10 pmol of each primer, 10 nmol of each dNTP and 1.9 U of Expand Long Template Enzyme mix (Expand Long Template PCR system, Roche). PCR products were analyzed on 0.8% agarose gels (Fig. 5). For PCR analysis of IES excision (Fig. 3, S7 and 10), PCR amplifications were performed with 1.9 U of Expand Long Template Enzyme mix (Expand Long Template PCR system, Roche). Oligonucleotides were purchased from Eurofins MWG Operon (see Table S6).

### Histone extraction and Western blot

Cell pellets were mechanically lysed in three volumes of lysis solution (0.25 M sucrose, 10 mM MgCl_2_, 10 mM Tris pH 6.8, 0.2% Nonidet P-40) with a Potter-Elvehjem homogenizer. Following the addition of 2.5 volumes of washing solution (0.25 M sucrose, 10 mM MgCl_2_, 10 mM Tris pH7.4), the nuclei-containing pellet was collected by centrifugation at 1000 g for 1 min and acid extraction of histones was performed as previously described [50]. 10 µg of histone extracts were used for Western blot. Electrophoresis and blotting were carried out according to standard procedures. The H3K27me3 (1∶500; Millipore, 07-449) and H3 (1∶10 000; Millipore, 07-690) primary antibodies were used. Secondary horseradish peroxidase-conjugated donkey anti-rabbit IgG antibody (Promega) was used at 1∶10 000 dilution followed by detection by ECL (SuperSignal West Pico Chemiluminescent Substrate, Thermo Scientific). For normalization, the membranes probed with H3K27me3 antibody were stripped in mild stripping buffer (glycine 200 mM, SDS 0.1%, Tween-20 1%, pH 2.2) and probed again with H3 antibody.

### Indirect immunofluorescence and fluorescence quantification

Cells were fixed for 30 minutes in solution I (10 mM EGTA, 25 mM HEPES, 2 mM MgCl2, 60m M PIPES pH 6.9 (PHEM 1X); paraformaldehyde 1%, Triton X-100 2.5%, Sucrose 4%) and for 10 minutes in solution II (PHEM 1X, paraformaldehyde 6.5%, Triton X-100 1.2%, Sucrose 4%). The primary antibodies used were rabbit polyclonal α-H3K27me3 (07-449, Millipore) and α-H3K9me3 (07-442, Millipore) at 1∶500. After incubation with the primary antibodies, cells were washed in 1X phosphate-buffered saline (PBS), incubated with the secondary antibodies (Alexa Fluor 568-conjugated goat anti-rabbit IgG, A-11036, Invitrogen) at 1∶500 for 1h, stained with 1 µg/mL Hoechst, washed in 1X PBS, centrifuged on microscope slides with the CytoSpin™ 4 Cytocentrifuge (Thermo Scientific) and finally mounted in Citifluor AF2 glycerol solution (Citifluor Ltd, London). Images were acquired using a Zeiss LSM 710 laser-scanning confocal microscope and a Plan-Apochromat 63x/1.40 oil DIC M27 objective. Z-series were performed with Z-steps of 0.5 µm.

To quantify the H3K27me3 and H3K9me3 signals, the Imaris 3D visualization software (Bitplane) was used. For each time point, the fluorescence intensities of H3K27me3/H3K9me3 in the developing MACs (signal) and in the corresponding volume of the cytoplasm (noise) were measured. The mean value and standard deviation of the signal to noise ratios were calculated using ten individual cells at each time point.

### DNA sequencing

DNA for deep-sequencing was isolated from post-autogamous cells as previously described [2] and sequenced by a paired-end strategy using Illumina GA-IIx and Hi-Seq next-generation sequencers (Table S2).

### Reference genomes

The following reference genomes [2] were used in the IES analyses and for read mapping.

MAC reference (strain 51):

http://paramecium.cgm.cnrs-gif.fr/download/fasta/ptetraurelia_mac_51.fa

MAC+IES reference (strain 51):

http://paramecium.cgm.cnrs-gif.fr/download/fasta/ptetraurelia_mac_51_with_ies.fa

PGM contigs:

http://paramecium.cgm.cnrs-gif.fr/download/fasta/assemblies/ptetraurelia_PGM_k51_ctg.fa

Macronuclear DNA reads for PiggyMac [2] and DCL5 depleted cells and for a biological replicate of the DCL2/3 co-silencing experiment [6] were obtained from the European Nucleotide Archive (Accession number ERA137420) (PGM) and the GenBank Sequence Read Archive (Accession numbers: SRX387766 (DCL2/3); SRX387766 (DCL5)).

### Genome-wide analysis of IES retention

After quality filtering and removal of adapters, Illumina reads were aligned to the reference genomes (*P. tetraurelia* MAC reference genome and MAC+IES reference genome) using BWA [51] with default parameters. Alignments were indexed with Samtools [52].

For each sample, IES retention scores were determined as follows, for each IES in the genome previously identified in [2]. The number of reads that contain the IES sequence (symbolised IES^+^) and the number of reads that contain only the macronuclear IES junction consisting of a TA dinucleotide (IES^−^) were determined. Only reads with unambiguous alignments were counted. Each read was counted only once to avoid over-counting owing to paralogous matches. Reads were only counted at IES ends, to avoid length biases resulting from IES length variation. The retention score (RS) of an IES is then given by the following equation:

RS  =  (IES^+^) / (IES^+^ + IES^−^)

Since RS are based on read counts, appropriate statistical tests allowed us to discriminate IES retention as a result of gene silencing from IES retention as a result of biases in Illumina sequencing or errors in the IES identification pipeline (estimated false positive rate ≤ 4%, [2]). First, we calculated the confidence interval (alpha  = 0.95) of the control retention score value, using the Pearson-Klopper exact method as implemented by the R binom package version 1.0–5 [53]. Then we tested for higher retention in the experiment, thanks to a frequency comparison test (based on a binomial law of probability) between the experimental retention score and the upper bound of the confidence interval in the control. Resulting p-values were adjusted for multiple testing using the Benjamini & Hochberg method [54]. IESs with adjusted p-value <0.05 are considered significantly retained in the sample.

### Data accessibility

The EZL1 KD, DCL2/3 KD and control DNA-seq datasets have been deposited in the European Nucleotide Archive (Accession number ERA309409). All IES retention scores may be obtained via ParameciumDB (http://paramecium.cgm.cnrs-gif.fr/).

## Supporting Information

Figure S1 Alignment of *Paramecium* and human histone H3 N terminal tails. Specificity of commercial H3K27me3 antibodies in *Paramecium*. A) N terminal tails (1-31) of human and *Paramecium* H3 are aligned. H3K9 epitope (in red) is present in *Paramecium* H3. Considering the fact that the N terminal tail of *Paramecium* histone H3 displays some amino acid differences around K27 with respect to mammalian histone H3 (H3K27 epitope in orange), we checked crossreactivity of the H3K27me3 antibodies and verified the specificity by dot blot and competition assays. B) Dot blot assay using H3 peptides. 100 pmol of each of the indicated peptides was spotted on the membrane, and probed with the H3K27me3 antibodies. Polyclonal antibodies raised against tri-methyl K27 showed specific reactivity with human (HsH3K27me3: SKAARK(Me3)SAP) or *Paramecium* tri-methyl K27 (PtH3K27me3: TKAARK(Me3)TAP) but not against no-methyl-, mono-methyl, di-methyl *Paramecium* K27, di-mehyl, tri-methyl *Paramecium* K9 (QTARK(Me3)STAGN). H3K27me3 antibodies are specific for H3K27me3 peptides (*Paramecium* and human) and do not cross react with *Paramecium* H3K9me3 peptide. C) Competition assay. 0 to 100 pmol of *Paramecium* H3K27me3 peptides were spotted and probed with the H3K27me3 antibodies alone or in presence of a 50-fold molar excess of the indicated peptides. Competition with the *Paramecium* (PtK27me3: TKAARK(Me3)TAP) or human (HsK27me3: SKAARK(Me3)SAP) tri-methyl K27 peptides completely eliminates the signal, while competition with the un-methylated peptide (PtK27me0: TKAARKTAP) does not.
(EPS)

Figure S2 Immunostaining and Western blot analysis with H3K27me3 antibodies during *Paramecium* life cycle, co-localization of Pgm-GFP fusion protein and H3K27me3. A) Immunostaining with H3K27me3 antibodies during *Paramecium* life cycle. Schematic representations of key nuclear events in *Paramecium* autogamy are depicted on the left: (a) vegetative growth, (b-c-d) meiosis I, (e) meiosis II, (f-i) MAC development. The time points refer to hours after T =  0 hr that is defined as the time when cells begin fragmentation of the maternal MAC, as evaluated by cytological observation. See Figure S4 for details on progression of autogamy and quantification of the number of stained cells at each time point. The grey to black color represents the intensity of H3K27me3 staining. Immunolabeling with H3K27me3 antibodies (in green) and staining with Hoechst (in red). Filled arrowheads indicate MICs, empty arrowheads indicate maternal MAC, dashed circles indicate the two developing MACs. Panels (f-i) are the entire images of the magnified views presented in Figure 1 (a-d). Note that H3K27me3 antibodies decorate the cilia and the oral apparatus. Scale bar is 10 µm. Magnified views of the MICs are presented in the right inside. Scale bar is 2 µm. B) Western blot with H3K27me3 and H3 antibodies. Acid extracts from (a) vegetative (Veg) or developing somatic MACs (Dev) of wild type cells at 25 hrs during autogamy, from nuclei of (b) control or Pgm-depleted cells, (c) control or Dcl2/3-depleted cells, (d) control or Dcl5-depleted cells control or (e) Ezl1-depleted cells at 25 hrs during autogamy were resolved on 15% SDS-PAGE, blotted, and probed with the indicated antibodies. (b) is a composite of two parts of the original image and a dotted line marks the cut/paste sites. C) Co-localization of Pgm-GFP fusion protein and H3K27me3. PGM-GFP transformed cells were immunolabeled with H3K27me3 antibodies and stained with Hoechst at 10 hrs during autogamy. Overlay of Z-projections of magnified views of Hoechst (in red), H3K27me3-specific antibodies (in blue) and PGM-GFP (in green) on selected stacks are presented. Dashed white circles indicate the two developing MACs. The other Hoechst-stained nuclei are fragments from the old vegetative MAC. Scale bar is 5 µm.
(TIFF)

Figure S3 Immunostaining with H3K9me3 antibodies during *Paramecium* life cycle, co-localization of Pgm-GFP fusion protein and H3K9me3. A) Immunostaining with H3K9me3 antibodies during *Paramecium* life cycle. Schematic representations of key nuclear events in *Paramecium* autogamy are depicted on the left: (a) vegetative growth, (b-c-d) meiosis I, (e) meiosis II, (f-i) MAC development. The time points refer to hours after T =  0 hr that is defined as the time when cells begin fragmentation of the maternal MAC, as evaluated by cytological observation. See Figure S4 for details on progression of autogamy and quantification of the number of stained cells at each time point. The grey to black color represents the intensity of H3K9me3 staining. Immunolabeling with H3K9me3 antibodies (in green) and staining with Hoechst (in red). Filled arrowheads indicate MICs, dashed circles indicate the two developing MACs. Panels (f-i) are the entire images of the magnified views presented in Figure 1 (a-d). Note that H3K9me3 antibodies decorate the cilia and the oral apparatus. Scale bar is 10 µm. Magnified views of the MICs are presented in the right inside. Scale bar is 2 µm. B) Co-localization of Pgm-GFP fusion protein and H3K9me3. PGM-GFP transformed cells were immunolabeled with H3K9me3 antibodies and stained with Hoechst at 10 hrs during autogamy. Overlay of Z-projections of magnified views of Hoechst (in red), H3K9me3-specific antibodies (in blue) and PGM-GFP (in green) on selected stacks are presented. Dashed white circles indicate the two developing MACs. The other Hoechst-stained nuclei are fragments from the old vegetative MAC. Scale bar is 5 µm.
(TIFF)

Figure S4 Progression of autogamy. A) Schematic representations of key nuclear events in *Paramecium* autogamy are depicted. B) and C) Progression of autogamy was followed by cytology with Hoechst staining in time course experiments after (B) ND7 (control), or PiggyMac (PGM) or EZL1 silencing and (C) ND7 (control) silencing or DCL2 and DCL3 co-silencing. Linear charts show quantifications of positive signals in the developing MAC after immunolabeling with H3K27me3- or H3K9me3- antibodies at each time point. The time-points refer to hours after T = 0 hr that is defined as the time when cells begin fragmentation of the maternal MAC, as evaluated by cytological observation. Because cells enter autogamy from a fixed point of the cell cycle [57], a minimum asynchrony of 5–6 h is observed between the first and the last cells to undergo meiosis. VEG: vegetative, MEI: meiosis, FRAG: fragmented maternal MAC, ANL: two visible developing MACs, KAR: karyonide. At least 100 cells were scored for each time point by fluorescence microscopy.
(EPS)

Figure S5 Phylogenetic analysis of SET domain proteins from *P. tetraurelia*, *T.thermophila* and *O. trifallax*. An unrooted Maximum-likelihood (ML) tree is shown. This tree has been constructed using the SET domains encoded by the genome of a large number of species. The full list of used sequences and studied species can be found in Table S1 and Text S1. We identified 13 monophyletic groups that include sequences from several different species. We named most of these groups by using the name(s) of the Human and/or *Drosophila* proteins that are included in the group. We tried to use as much as possible the nomenclatures used in previous phylogenetic analyses of SET domain proteins [58], [59]. In two cases, the monophyletic groups do not include animal proteins and we used the name(s) of the included *Arabidopsis* protein(s) to name these groups (ATXR3 and ATXR5/6). We listed in the figure the proteins from *Paramecium tetraurelia* (Ptet), *Oxytricha trifallax* (Otri), and *Tetrahymena thermophila* (Tthe), which belong to the different monophyletic groups. The robustness of the nodes that define the different monophyletic groups was assessed by evaluating their statistical support (aLRT values) in the ML analysis and by performing phylogenetic analyses (ML and Bayesian inference) using a smaller sampling of species (ciliates + Human + *Drosophila* + yeasts). This is represented on the tree by the presence close to the name of the group of *** (aLRT values>0,8 and groups similar in all analyses), ** (0,8>aLRT values>0,5 and groups similar in all analyses), or * (aLRT values<0,5 and/or groups significantly different in the analyses with a different sampling of species). We obtained strong support for the existence of ciliate members of the EZH, ASH/SET2/NSD and SET1 groups that also include proteins from several other species including animals and *Arabidopsis*. No member of the Suvar39/EHMT/SETDB8/SETMAR group could be identified in the ciliate genomes. Several ciliate SET domains cluster with either *Arabidopsis* ATXR3 or *Arabidopsis* ATXR5/6 in groups that only include sequences from a small number of species and none from Human or *Drosophila*. These groups may therefore correspond to ancestral SET domain proteins that have been lost in some lineages such as animals or to divergent members of some other groups. This latter possibility is supported by the fact that the two groups are associated to the ASH/SET2/NSD group in the phylogenetic analyses made on the ciliates + Human + *Drosophila* + yeasts dataset. The inclusion of *T. thermophila* and *O. trifallax* proteins in the SMYD and SETD7 groups, as well as the belonging of a large number of ciliate proteins to the SETD6 group, has to be taken with caution, given the poor support of these groups, and may correspond to the artefactual grouping of highly divergent sequences.
(EPS)

Figure S6 Alignment of SET domains from EZH-EZL proteins from *P. tetraurelia*, *T. thermophila*, *O. trifallax* and other organisms. Sequences were aligned using the Muscle v3.8 software. The white text on a black background denotes invariant residues; white text on a gray background indicates conserved residues. Highly conserved residues are highlighted with different colors according to [60], [61]: catalytic site (red), adenosylmethionine (AdoMet) binding pocket (green), lysine substrate binding pocket (blue). EZL3a and EZL3b are gene duplicates from the last whole genome duplication [25]. Note that Ptet_EZL3a, Ptet_EZL3b and Ptet_EZL4 do not show all conserved residues. Accession numbers are given in Table S1 and Text S1. Species name abbreviations: Amac  =  *Allomyces macrogynus* (Fungi); Atha  =  *Arabidopsis thaliana* (Viridiplanta); Aque  =  *Amphimedon queenslandica* (Metazoa); Dmel  =  *Drosophila melanogaster* (Metazoa); Hsap  =  *Homo sapiens* (Metazoa); Lgig  =  *Lottia gigantea* (Metazoa); Mbre  =  *Monosiga brevicolis* (Choanoflagellata); Nvec  =  *Nematostella vectensis* (Metazoa); Otri  =  *Oxytricha trifallax* Ptet  =  *Paramecium tetraurelia* (Ciliata); Spun  =  *Spizellomyces punctatus* (Fungi); *Tetrahymena thermophila* (Ciliata); Ttra  =  *Thecamonas trahens* (Apusozoa).
(DOCX)

Figure S7 Expression patterns of EZL genes after EZL1 knockdown. A) Progression of autogamy in time course experiments, in which ICL7 (control) or EZL1 genes have been knocked down by RNAi. See Figure S4 legend. B) Detection of EZL and PGM mRNA during autogamy by RT-PCR. Total RNAs were extracted at each time point shown in A, were reverse transcribed and cDNAs were amplified by PCR with gene specific primers and, as a loading control, with primers for the T1b gene, which encodes a component of the secretory granules [55]. C) PCR detection of IES 51A4578 circles with divergent primers on genomic DNA at each time point shown in (B) after ICL7 (control) or EZL1 silencing. Panels B and C for ICL7 silencing are reproduced from Figures 3C and 3D to facilitate comparison.
(EPS)

Figure S8 Localization of a GFP-EZL1 fusion protein. A) Localization of a GFP-EZL1 fusion protein during vegetative growth (a), meiosis I and II (b-e) and MAC development (f-j). Panels (g-j) are the entire images of the magnified views presented in Figure 4 (a-d). Filled arrowheads indicate MICs, empty arrowheads indicate maternal MAC, dashed circles indicate the two developing MACs. The grey to black color represents the GFP-EZL1 intensity. Scale bar is 10 µm. B) GFP-EZL1 fusion protein localization after PGM or DCL2/3 silencing at 40 hrs during autogamy. Scale bar is 5 µm.
(TIFF)

Figure S9 Sardine coverage. The bar plots represent read coverage of 8 individual copies of the Sardine transposon (GenBank Accession No. HE774468-HE774475). The coverage was determined by mapping reads using BWA with default parameters, for the control (KLEB) dataset (grey), PGM silencing (red), EZL1 silencing (green), DCL2/3 co-silencing (yellow), and DCL5 silencing (blue) datasets. The normalized units (RPKM) are reads per kilobase of the transposon sequence per million library read mapped against the MAC reference genome.
(EPS)

Figure S10 PCR analysis of IES retention after EZL1, DCL2/3 or PGM silencing. PCR analysis of IES retention with primers (black arrows, Table S6) located on either side of the IES in mass autogamies after RNAi-mediated silencing of the indicated genes. Total DNA samples were prepared from starved post-autogamous cells at approximately 72 hrs. Because the maternal MAC is still present at this stage, the excised version is amplified in all cases; the IES-retaining fragment can be detected only if it accumulates in the zygotic developing MACs. Control: unrelated negative control (ND7 or ICL7 RNAi); “+ “: positive PCR control on cloned MIC DNA, or DNA from mating-type E cells in the case of the IES mtA. Note that i) in the case of IES 51A6649, the IES-containing fragment is shorter in DCL/3 and EZL1 KDs than in PGM KD and MIC DNA, due to excision of a 29-bp internal IES [11], ii) in the case of IES 51A2591, the IES-containing fragment also lacks a 28-bp internal IES [11] in DCL2/3 KD.
(EPS)

Figure S11 IES retention score and size in control, EZL1, DCL2/3 or PGM datasets. IESs were grouped by size (cf. legend of Figure 6B), and boxplots were determined to show the distribution of A) PGM dataset retention scores, B) control (KLEB) dataset retention scores, C) DCL2/3 dataset retention scores and D) EZL1 silencing sample retention scores (reproduced from Figure 6B to facilitate comparison). E) The proportion of significantly retained IESs for PGM, DCL2/3 and EZL1 datasets are plotted as a function of IES size, using the same size groups as for parts A-D, corresponding to the peaks in the IES size distribution. This representation shows, for example, that 34% of the IESs in the smallest peak are significantly retained after EZL1 silencing. In the PGM dataset, essentially all IESs are significantly retained except in the last few groups of largest size, and that is because of the small number of IESs in those groups, introducing a lot of noise in the curve. In the EZL1 dataset, the increase in significantly retained IESs is dramatic over the first 3 groups in the size distribution and then flattens out. The variation for the largest size groups is very similar to the variation found for the PGM dataset, in support of the hypothesis that this variation is noise that arises from the small number of IESs in those groups. For the DCL2/3 dataset, few IESs are significantly retained in any of the groups. However, there does appear to be an increase in significantly retained IESs for the largest IES sizes. As already noted, the largest groups include very few IESs introducing a lot of noise, however for the DCL2/3 dataset, the variations are different than for the other 2 samples, in support of a real increase in significant retention of IESs larger than ∼ 500 bp. In A-D, the stars indicate that the retention score distribution of a group is significantly different from the distribution of the previous group.
(EPS)

Figure S12 IES properties. IESs are grouped according to sample: those with significant retention scores (EZL, DCL2/3), those that do not have significant retention scores (nEZL, nDCL/2/3) and ALL IESs. The left column (A,C) concerns only the IESs in the first peak of the size distribution (< 32 bp) and the right column (B,D) considers all IESs whatever their size. These IES groups were examined with respect to GC content (A,B) and with respect to the proportion of IESs that are within genes (C,D). The number of IESs in each group is indicated in boldface type inside the boxplots in A and B. Significant differences in GC content (A,B) and genomic distribution (C,D) for sensitive versus insensitive IESs are designated by asterisks (p<2.2 10 -16) and were determined using a Mann-Whitney test (A,B) or a Chi2 test (C,D).
(EPS)

Figure S13 EZL1 retention score for DCL2/3 and DCL5 retained IESs. Boxplots show the distribution of EZL1 retention scores for EZL1-retained IESs (green), DCL2/3-retained IESs (yellow) and DCL5-retained IESs (blue), showing that the significantly retained IESs in the DCL2/3 and the DCL5 datasets are among the most retained IESs in the EZL1 dataset. Significant differences are designated by asterisks (p<2.2 10-16) and were determined using a Mann-Whitney test.
(EPS)

Table S1 SET domain proteins identified in the somatic MAC genome of *P. tetraurelia.* Accession numbers (see ParameciumDB, http://paramecium.cgm.cnrs-gif.fr/), names and conserved domains are indicated.
(DOCX)

Table S2 Sequencing and mapping statistics. Read statistics are provided for the samples sequenced for this study, with the exception of the PGM and DCL5 silencing samples, which were previously published [2], [6]; European Nucleotide Archive Acc No. ERA137444 (PGM); GenBank Sequence Read Archive Acc No. SRX387766 (DCL5).
(DOCX)

Table S3 Sequence complexity of control, PGM, EZL1, DCL2/3 and DCL5 datasets. The previously published contigs assembled from a PGM dataset [2] were used as reference, representing the currently best available germline DNA assembly. However, only contigs larger than 1 kb were considered, representing 91 Mb of sequence complexity, of which 89 Mb are covered by read mapping above our cutoff (i.e. 2 reads per kb of contig per million mapped reads in the library). Reads from each sample were mapped to the PGM contigs using BWA with default parameters, to determine the complexity of the contigs covered by at least 2 reads per kilobase of contig per million reads in the library (RPKM), giving the first row of the table (“PGM” Reference). In addition, a set of PGM contigs were selected that had control (KLEB) coverage below the cutoff of 2 RPKM (“PGM not KLEB” Reference), representing pure germline DNA not collinear with MAC chromosomes. The coverage of these contigs by each sample was also determined (second row of table). Note that 76 Mb is the complexity of the MAC reference genome [25] and that the total germline complexity is at least 10 Mb greater than 91 Mb. Although the N50 of the PGM assembly that we have used as Reference is 28,076 bp (meaning that half of the assembly is contained in contigs larger than 28 kb), the analysis presented in the table uses the 7,310 contigs greater in size than 1 kb out of a total of 30,013 contigs, a choice dictated by the necessity of obtaining good paired-end read mapping to calculate coverage.
(DOCX)

Table S4 IES retention analyzed by PCR and deep-sequencing after control, DCL2/3, EZL1 and PGM silencing. Maternally (mcIES) or non-maternally controlled (non-mcIES) is indicated as “+” or “–“ respectively, based on previous studies [11]. Star(s) following a retention score indicate that the IES is significantly retained (cf. Materials and Methods and the legend to Figure 6B). NA: not analyzed. No retention score can be calculated for (i) the mtA IES since it is retained in the MAC reference genome [13] and for (ii) IES51A1835, IES51A4404, IES51A2591 and IES51A4578 because the control strain carries a maternally inherited MAC deletion of the A gene. ^1^: S. Duharcourt and E. Meyer, personal communication
(DOCX)

Table S5 Anchois retention score. The 28 IESs that were used to identify the Anchois Tc1/mariner transposon [2] are provided with their ParameciumDB Accession Numbers, size and retention scores in the different RNAi datasets under consideration. Retention scores followed by a star are statistically significant. The DCL23_r sample is a biological replicate previously published by [6] that was retrieved from the Genbank Short Read Archive (Accession number SRX387766).
(DOCX)

Table S6 Oligonucleotides used in this study.
(DOCX)

Text S1 Accession numbers and sequences of SET domain protein used in Figure 3 and Figures S5- S6 (94 pages).
(DOCX)
