# Supplementary figures and images for: Local Effect of Enhancer of Zeste-Like Reveals Cooperation of Epigenetic and cis-Acting Determinants for Zygotic Genome Rearrangements
Source: PLoS Genet. 2014 Sep 25;10(9):e1004665. doi: 10.1371/journal.pgen.1004665 (PMC4177680; doi:10.1371/journal.pgen.1004665)

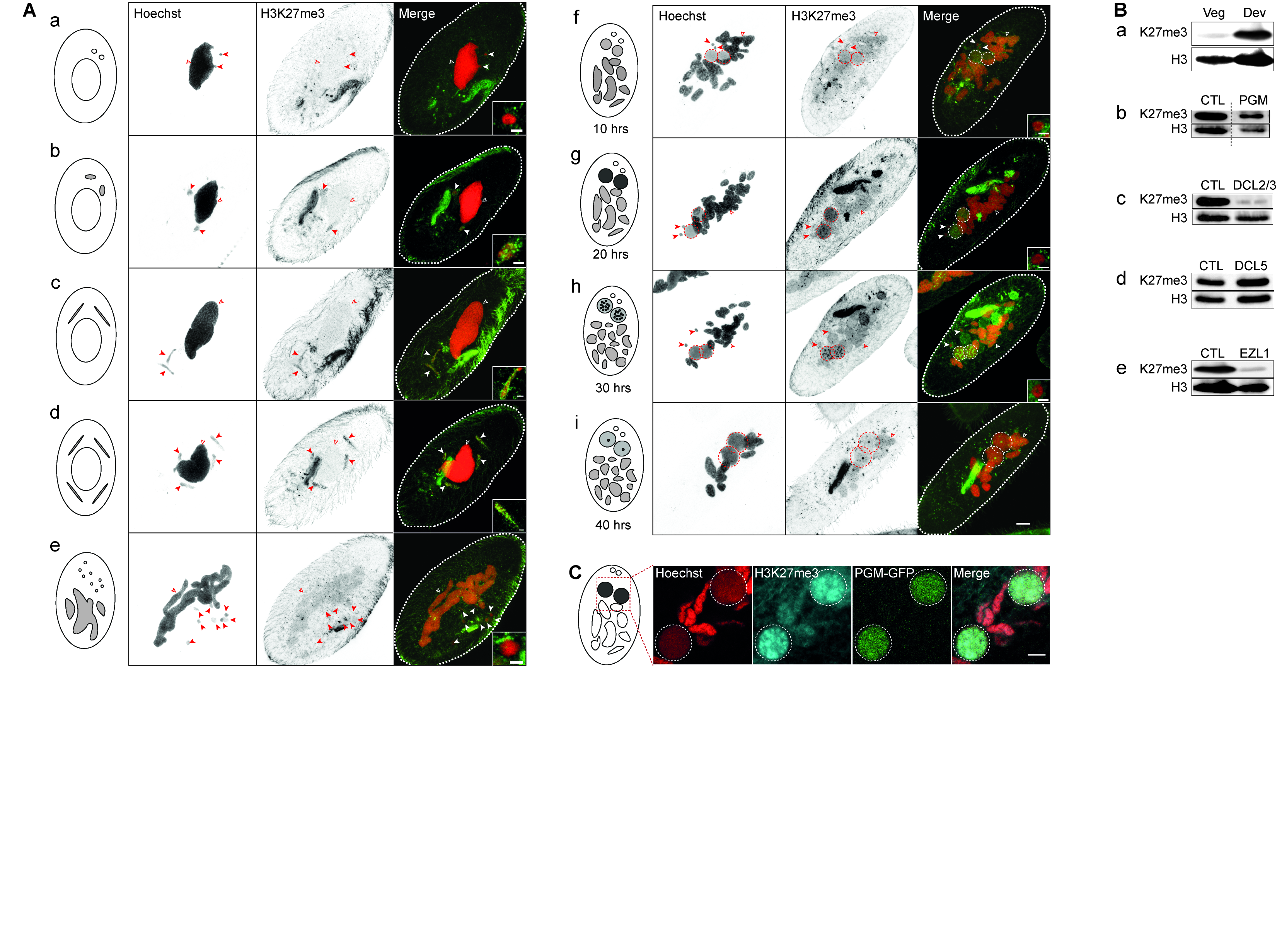

Supplement: Figure S2 — Immunostaining and Western blot analysis with H3K27me3 antibodies during Paramecium life cycle, co-localization of Pgm-GFP fusion protein and H3K27me3. A) Immunostaining with H3K27me3 antibodies during Paramecium life cycle. Schematic representations of key nuclear events in Paramecium autogamy are depicted on the left: (a) vegetative growth, (b-c-d) meiosis I, (e) meiosis II, (f-i) MAC development. The time points refer to hours after T = 0 hr that is defined as the time when cells begin fragmentation of the maternal MAC, as evaluated by cytological observation. See Figure S4 for details on progression of autogamy and quantification of the number of stained cells at each time point. The grey to black color represents the intensity of H3K27me3 staining. Immunolabeling with H3K27me3 antibodies (in green) and staining with Hoechst (in red). Filled arrowheads indicate MICs, empty arrowheads indicate maternal MAC, dashed circles indicate the two developing MACs. Panels (f-i) are the entire images of the magnified views presented in Figure 1 (a-d). Note that H3K27me3 antibodies decorate the cilia and the oral apparatus. Scale bar is 10 µm. Magnified views of the MICs are presented in the right inside. Scale bar is 2 µm. B) Western blot with H3K27me3 and H3 antibodies. Acid extracts from (a) vegetative (Veg) or developing somatic MACs (Dev) of wild type cells at 25 hrs during autogamy, from nuclei of (b) control or Pgm-depleted cells, (c) control or Dcl2/3-depleted cells, (d) control or Dcl5-depleted cells control or (e) Ezl1-depleted cells at 25 hrs during autogamy were resolved on 15% SDS-PAGE, blotted, and probed with the indicated antibodies. (b) is a composite of two parts of the original image and a dotted line marks the cut/paste sites. C) Co-localization of Pgm-GFP fusion protein and H3K27me3. PGM-GFP transformed cells were immunolabeled with H3K27me3 antibodies and stained with Hoechst at 10 hrs during autogamy. Overlay of Z-projections of magnified views of Hoec [file pgen.1004665.s002.tiff]

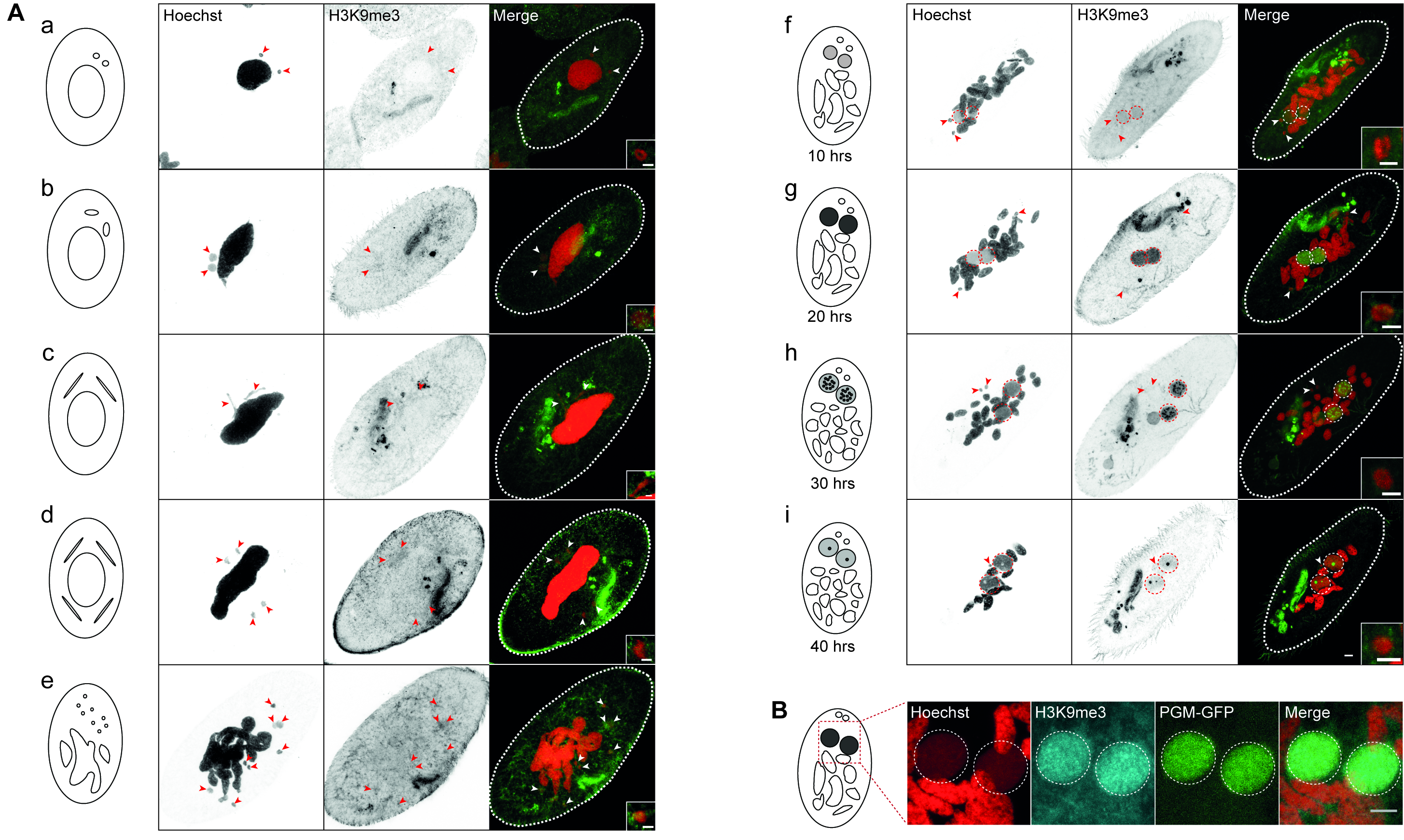

Supplement: Figure S3 — Immunostaining with H3K9me3 antibodies during Paramecium life cycle, co-localization of Pgm-GFP fusion protein and H3K9me3. A) Immunostaining with H3K9me3 antibodies during Paramecium life cycle. Schematic representations of key nuclear events in Paramecium autogamy are depicted on the left: (a) vegetative growth, (b-c-d) meiosis I, (e) meiosis II, (f-i) MAC development. The time points refer to hours after T = 0 hr that is defined as the time when cells begin fragmentation of the maternal MAC, as evaluated by cytological observation. See Figure S4 for details on progression of autogamy and quantification of the number of stained cells at each time point. The grey to black color represents the intensity of H3K9me3 staining. Immunolabeling with H3K9me3 antibodies (in green) and staining with Hoechst (in red). Filled arrowheads indicate MICs, dashed circles indicate the two developing MACs. Panels (f-i) are the entire images of the magnified views presented in Figure 1 (a-d). Note that H3K9me3 antibodies decorate the cilia and the oral apparatus. Scale bar is 10 µm. Magnified views of the MICs are presented in the right inside. Scale bar is 2 µm. B) Co-localization of Pgm-GFP fusion protein and H3K9me3. PGM-GFP transformed cells were immunolabeled with H3K9me3 antibodies and stained with Hoechst at 10 hrs during autogamy. Overlay of Z-projections of magnified views of Hoechst (in red), H3K9me3-specific antibodies (in blue) and PGM-GFP (in green) on selected stacks are presented. Dashed white circles indicate the two developing MACs. The other Hoechst-stained nuclei are fragments from the old vegetative MAC. Scale bar is 5 µm. (TIFF) [file pgen.1004665.s003.tiff]

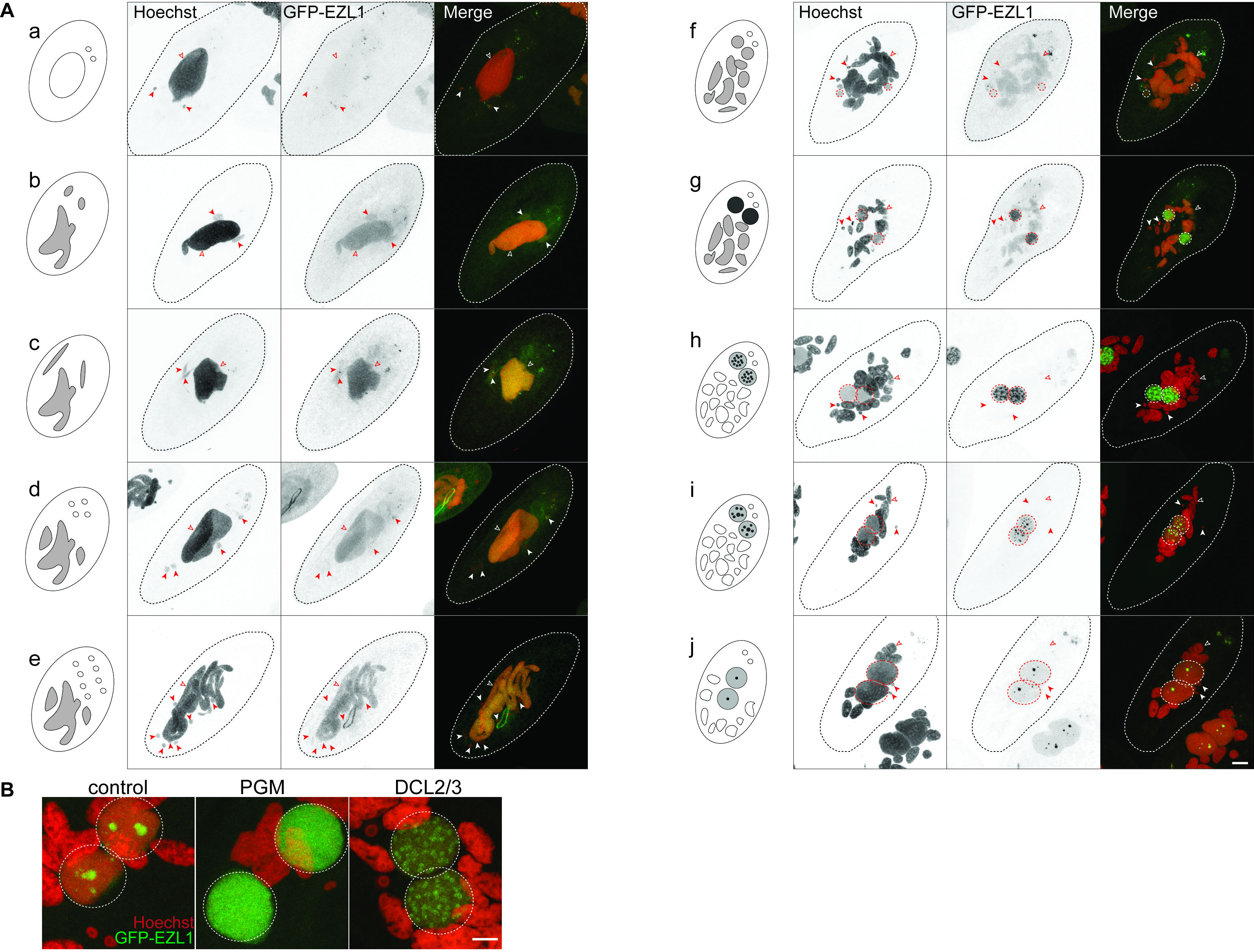

Supplement: Figure S8 — Localization of a GFP-EZL1 fusion protein. A) Localization of a GFP-EZL1 fusion protein during vegetative growth (a), meiosis I and II (b-e) and MAC development (f-j). Panels (g-j) are the entire images of the magnified views presented in Figure 4 (a-d). Filled arrowheads indicate MICs, empty arrowheads indicate maternal MAC, dashed circles indicate the two developing MACs. The grey to black color represents the GFP-EZL1 intensity. Scale bar is 10 µm. B) GFP-EZL1 fusion protein localization after PGM or DCL2/3 silencing at 40 hrs during autogamy. Scale bar is 5 µm. (TIFF) [file pgen.1004665.s008.tiff]
