# Supplementary material for: Local Effect of Enhancer of Zeste-Like Reveals Cooperation of Epigenetic and cis-Acting Determinants for Zygotic Genome Rearrangements
Source: PLoS Genet. 2014 Sep 25;10(9):e1004665. doi: 10.1371/journal.pgen.1004665 (PMC4177680; doi:10.1371/journal.pgen.1004665)
Supplement: Figure S6 — Alignment of SET domains from EZH-EZL proteins from P. tetraurelia, T. thermophila, O. trifallax and other organisms. Sequences were aligned using the Muscle v3.8 software. The white text on a black background denotes invariant residues; white text on a gray background indicates conserved residues. Highly conserved residues are highlighted with different colors according to [60], [61]: catalytic site (red), adenosylmethionine (AdoMet) binding pocket (green), lysine substrate binding pocket (blue). EZL3a and EZL3b are gene duplicates from the last whole genome duplication [25]. Note that Ptet_EZL3a, Ptet_EZL3b and Ptet_EZL4 do not show all conserved residues. Accession numbers are given in Table S1 and Text S1. Species name abbreviations: Amac = Allomyces macrogynus (Fungi); Atha = Arabidopsis thaliana (Viridiplanta); Aque = Amphimedon queenslandica (Metazoa); Dmel = Drosophila melanogaster (Metazoa); Hsap = Homo sapiens (Metazoa); Lgig = Lottia gigantea (Metazoa); Mbre = Monosiga brevicolis (Choanoflagellata); Nvec = Nematostella vectensis (Metazoa); Otri = Oxytricha trifallax Ptet = Paramecium tetraurelia (Ciliata); Spun = Spizellomyces punctatus (Fungi); Tetrahymena thermophila (Ciliata); Ttra = Thecamonas trahens (Apusozoa). (DOCX) [file pgen.1004665.s006.docx]

Tthe_EAS01979 1 -------------------------------------HAYVLREMQNVIQIYVNRV----
Tthe_EAS04314 1 --------------------------------KDRCKNNEYLYQKQEVIY----------
Ptet_EZL4 1 -----------------RGCNCK----DRCSFDSRCSCRKDNMECDPLVCKCCSIDSNF-
Ptet_EZL3a 1 -----------------KGCACK----DRCSIDSKCSCRRDNVECDPLVCKCCTLDSNL-
Ptet_EZL3b 1 ---------------KLQGCTCK----ERCSIDSKCSCRIDNVECDPLICKCCKVDSNF-
Ptet_EZL2 1 ---------KGKCEKKIKRCDCKNCGYDEKKRKFQCTCFNLGFECDPSICKCT-------
Ptet_EZL1 1 --------CKGQCQQKSKSCDCRVCGYDEKKQKHSCPCYISGYECDPQLCKCQ-------
Ttra_AMSG_09360T0 1 ------------------SDSSSSTSLNERPRKRRASRRKRVQRREPVTGTAPPAAAVQ-
Tthe_EZL1 1 ----------------------------------NCQCRSKLRECDPEVCRCFCNRNART
Otri_Contig14971_0_g38 1 -------------------------------GKL-SVNKVGGQMQQQSLAR---------
Amac_AMAG_02871T0 1 -----------------------------------CLCVQWMRECGPACACPCR------
Spun_SPPG_03589T0 1 PPAVPKKATWDQTENRHVKGHFK----LQANGKEFCRCVHEERECDPDVCRSCGADVECG
Atha_MEA 1 ---------------------AI----GQCTNRQ-CPCFAANRECDPDLCRSCPLSCGDG
Atha_CLF 1 ---------------------------------Q-CPCFAADRECDPDVCRNCWVIGGDG
Atha_SWN 1 -----------------------------CRSRQ-CPCFAAGRECDPDVCRNCWVSCGDG
Mbre_estExt_fgenesh2_pg.C_20049 1 ---------EYRGEVRWAACCML----CVCVCVCVCVCVCVCVCVCVCVCVCVCVC----
Dmel_Ez 1 -----FCNCSSDCQNRFPGCRCK----AQCNTKQ-CPCYLAVRECDPDLCQACGADQFKL
Lgig_estExt_Genewise1.C_sca_1470059 1 --------------------------KAQCNTKQ-CPCFLAVRECDPDLCQTCGSDQFDT
Nvec_estExt_GenewiseH_1.C_5680002 1 -------QCNSDCQNRFPGCRCK----AQCNTKQ-CPCFLAVRECDPDLCGTCGADNFDQ
Aque_Aqu1_209566 1 ------------------GCRCR----SSCSTKH-CPCFLAVRECDPDLCSTCGAGDNLE
Hsap_EZH1 1 ---EKFCQCNPDCQNRFPGCRCK----TQCNTKQ-CPCYLAVRECDPDLCLTCGASEHWD
Hsap_EZH2 1 -----FCQCSSECQNRFPGCRCK----AQCNTKQ-CPCYLAVRECDPDLCLTCGAADHWD


Tthe_EAS01979 20 ------------LNANLNVITIRSHLTKSKVVIANSEICGG-LGIYNVYPLQKGDLITIY
Tthe_EAS04314 19 ------------LNKNPNLFYFMLSK---RTVIGMSFVCQG-LGLYNVFPIQQHSLVLSY
Ptet_EZL4 39 ------------ICSNTQILIKNVK----PTLLARSTVCSG-LGLFSKHFIMKGELIILY
Ptet_EZL3a 39 ------------VCSNTQILINNVK----PTLLGRSGVCNG-IGVFARNYIMKDELIILY
Ptet_EZL3b 41 ------------VCSNIQILINNVQ----PTLLGRSGVCNG-LGVFARNYIMKDELIILY
Ptet_EZL2 45 ------------NCNNVNLTLGISK----QLILGNSLICNG-IGLFAAQHFKTYDFIGEY
Ptet_EZL1 46 ------------SCSNKNLLMQIRK----SLVLGKSLICNG-LGLFAAQNFKVCDFVGEY
Ttra_AMSG_09360T0 42 ------------PCRNLAHFNKVHN----KVVVGISTVAG--WGLFAGQPISRGQFIGEY
Tthe_EZL1 27 IQKFGLNID---YCTNSQALYNCKP----RVLLGKSLVCEG-LGLFAGQDFKKNQYIGCY
Otri_Contig14971_0_g38 20 ------------LCKNVPLTLNFPCK---RVFTAKSTLCDEIVGLFTMDRVKKDELIMEY
Amac_AMAG_02871T0 20 ------------ACKNREIGKGVDHDKKLRVDVSTIPKAG--WGLFAQCDFKADDFLGEY
Spun_SPPG_03589T0 57 AVGRKTSSL---TCHNQAMQRNMKK----HLCVGDSEVHG--RGAFLREPAEKGELLAEY
Atha_MEA 35 TLGETPVQI---QCKNMQFLLQTNK----KILIGKSDVHG--WGAFTWDSLKKNEYLGEY
Atha_CLF 27 SLGVPSQRGDNYECRNMKLLLKQQQ----RVLLGISDVSG--WGAFLKNSVSKHEYLGEY
Atha_SWN 31 SLGEAPRRGEG-QCGNMRLLLRQQQ----RILLGKSDVAG--WGAFLKNSVSKNEYLGEY
Mbre_estExt_fgenesh2_pg.C_20049 44 ------------VCVCVCVCVCVCV----CVCVCVCVCVC---------------VCVCV
Dmel_Ez 51 TKI---------TCKNVCVQRGLHK----HLLMAPSDIAG--WGIFLKEGAQKNEFISEY
Lgig_estExt_Genewise1.C_sca_1470059 34 IKI---------SCKNVSVQRSQGK----HLLLAPSDVAG--WGIFLKEPAEKNEFISEY
Nvec_estExt_GenewiseH_1.C_5680002 49 DSK---------TCKNVSLQRGQRK----HMLLAPSDVAG--WGIYIKQSVKKNEFISEY
Aque_Aqu1_209566 38 MKFT--------TCKNVSIQRGQKK----HLLMVLSDVAG--WGIFLKDGAEKNEFISEY
Hsap_EZH1 53 CKVV--------SCKNCSIQRGLKK----HLLLAPSDVAG--WGTFIKESVQKNEFISEY
Hsap_EZH2 51 SKNV--------SCKNCSIQRGSKK----HLLLAPSDVAG--WGIFIKDPVQKNEFISEY **AdoMet** **G XG catalytic Y**
Tthe_EAS01979 67 YGEVLQDLDIIIR---DSWKPSNLFYIFSLLG-DL--TVDSKYIGNKSRFMNHSKS---K
Tthe_EAS04314 63 LGEYISEDEQVIR---ESWLKSDIFYTFTKNDFEK--LVDSKYFGNKSRFMNHHISLE--
Ptet_EZL4 82 IGEAIIDDEDEIR---DQFDDAFSFYNYQLSDERY--SLDSRFCGNESRFINHNSL--NL
Ptet_EZL3a 82 IGEVLIDDEDEIR---DQFDDTFTFYNYSLNDDKY--SLESRFCGNESRFINHNSS--NL
Ptet_EZL3b 84 IGEVLIDDEDEIR---DQFDDTFTFYNYQLNEDKY--SLESRFCGNESRFINHNSQ--NL
Ptet_EZL2 88 RGNYLLLDEESYI-IEQCNMLTGKHYLFEVDD-KW--QVDGTYYSNYLRFINHATNQSET
Ptet_EZL1 89 TGNYILLDDESMA-IEQCDWITNNHYLFEVDD-KW--QVDGTYYSNCLRYINHATKKSDL
Ttra_AMSG_09360T0 84 VGEVVSSTEVERR--HSEYSSMRISYVFDYNR-DF--VIDAFRKGSGTKLINHSDE----
Tthe_EZL1 79 IGEIINEKQGTER--QEVQQPQGISYLFMLNK-ET--DVDSFRYGNKMRYVNHNCG--SM
Otri_Contig14971_0_g38 65 TGKVITQEGQKETVDQLVNDLRGRSYGFTLDR-LT--TLDAVYVGNLMRFANHSSD--KL
Amac_AMAG_02871T0 66 SGEVLSSDEADRR--GIIYDRKSLNYCFQLAT-DA--VVDAYRLGPVLRFCNHAAK----
Spun_SPPG_03589T0 108 TGGVISVAESNRR--DAINAASKLNYLFSLGD-KYAPLVDASRKGNKTRFINHGEY-PDK
Atha_MEA 86 TGELITHDEANER--GRIEDRIGSSYLFTLND-QL--EIDARRKGNEFKFLNHSAR----
Atha_CLF 81 TGELISHKEADKR--GKIYDRENCSFLFNLND-QF--VLDAYRKGDKLKFANHSPE----
Atha_SWN 84 TGELISHHEADKR--GKIYDRANSSFLFDLND-QY--VLDAQRKGDKLKFANHSAK----
Mbre_estExt_fgenesh2_pg.C_20049 73 CVCIISQEEADRR--GKVYDQLKCSFLFNLNQ-EY--VVDATRKGNKIRFANHAND----
Dmel_Ez 96 CGEIISQDEADRR--GKVYDKYMCSFLFNLNN-DF--VVDATRKGNKIRFANHSIN----
Lgig_estExt_Genewise1.C_sca_1470059 79 CGEIISQDEADRR--GKVYDKYMCSFLFNLNN-DF--VVDATRKGNKIRFANHSIN----
Nvec_estExt_GenewiseH_1.C_5680002 94 CGEVISQDEADRR--GKVYDKYMCSFLFNLNN-DF--VVDATRKGNKIRFANHSIS----
Aque_Aqu1_209566 84 CGEIISQDEADRR--GKVYDKYMCSFLFNLNN-DY--VVDATRKGNKIRFANHSVD----
Hsap_EZH1 99 CGELISQDEADRR--GKVYDKYMSSFLFNLNN-DF--VVDATRKGNKIRFANHSVN----
Hsap_EZH2 97 CGEIISQDEADRR--GKVYDKYMCSFLFNLNN-DF--VVDATRKGNKIRFANHSVN----
 **AdoMet** **RFXNHS**

Tthe_EAS01979 118 ENSYAKMVYAKGGYTIGLFSNEKIIPGTELLFDYD-GQGTLKLKYEWIMQKADS---KAK
Tthe_EAS04314 116 -NCFARSVYSRGDYHLGLYAKQDIPPGNELLFDYD-ADKTLAVEKEWITRNNYSN--YKI
Ptet_EZL4 135 NNCRTNQIFTCGQYQLAIFAIKNIDPEQEILLNYNEGESLNKEVHNWNEQQQ-----QFW
Ptet_EZL3a 135 MNCKTRQIFSSGKFQLAIYALKDIYPEQEILLNYNEGDQINRDLNNWVDINT-----QYW
Ptet_EZL3b 137 MNCKTRQIFSSGKFQLAIYALTEISPQQEILLNYNEGDQINKDLNNWVDMNS-----QYW
Ptet_EZL2 144 ANCQAIILFSEGRWKIGMLATREIEVGQELYFDYG-----DKFKTKWLQEFNKISE-RYF
Ptet_EZL1 145 ANCQAQILFSEGRWRIAMFTTKNISIGEELFFDYG-----DKFLTKWLTDFNKLCD-DYY
Ttra_AMSG_09360T0 135 PNVHPMTKVVNGVERVGLYASRDLRTGDELLFCYN----YDKEKREWYLDSDFQQILEAE
Tthe_EZL1 132 ANCKVDVIYNRGINIVRFSAKEDIQKGQEIYFDYN-----RNYQIEWMKYFNEYYD-KFE
Otri_Contig14971_0_g38 120 SNCKINMIFAQGMQRVCLVASRNIDQGEELFFDYG-----FSKEFEWLKDYDRNGCIDVI
Amac_AMAG_02871T0 117 SNVNARVAFVDGSQRIGLYAKKNINAGDELFLDYGRMYWGDESGTPKAAASTSSSRSSPS
Spun_SPPG_03589T0 164 ANCFPVVKMVSGELRVGIYAARRIEAEKELFFDYG----------------------EDF
Atha_MEA 137 PNCYAKLMIVRGDQRIGLFAERAIEEGEELFFDYC--YGPEHADWSRGREPRKTGASKRS
Atha_CLF 132 PNCYAKVIMVAGDHRVGIFAKERILAGEELFYDYR--YEPDRAP-AWAKKPEAPGSKKDE
Atha_SWN 135 PNCYAKVMFVAGDHRVGIFANERIEASEELFYDYR--YGPDQAP-VWARKPE--GS-KKD
Mbre_estExt_fgenesh2_pg.C_20049 124 PNCCARVMMVAGEHRIGIFAERDIPAGRELFFNYR--YGPTDAL-------------KYV
Dmel_Ez 147 PNCYAKVMMVTGDHRIGIFAKRAIQPGEELFFDYR--YGPTEQL-------------KFV
Lgig_estExt_Genewise1.C_sca_1470059 130 PNCYAKVMMVNGDHRIGIFAKRSIQSGEELFFDYR--YGPTEQL-------------RFV
Nvec_estExt_GenewiseH_1.C_5680002 145 PNCYAKVMMVNGDHRIGIFAKRDIEAGEELFFDYR--YSATDAL-------------KFV
Aque_Aqu1_209566 135 PNCYAKVMIVGGDHRIGIFAKHNIELEEELFFDYR-------------------------
Hsap_EZH1 150 PNCYAKVVMVNGDHRIGIFAKRAIQAGEELFFDYR--YSQADAL-------------KYV
Hsap_EZH2 148 PNCYAKVMMVNGDHRIGIFAKRAIQTGEELFFDYR--YSQADAL-------------KYV
 **lysine binding** **EELXFDY**

Tthe_EAS01979 174 QIQEDNIIVFEAHNSPAKNQNLIKQKKRK---
Tthe_EAS04314 172 RMDNIRITIPRNKDLTRFNSTLIIIDDEN---
Ptet_EZL4 190 NYIQNTDTKER---------------------
Ptet_EZL3a 190 NYRQTTLIGKK---------------------
Ptet_EZL3b 192 NYKQTTFIG-----------------------
Ptet_EZL2 198 QGK-----------------------------
Ptet_EZL1 199 KK------------------------------
Ttra_AMSG_09360T0 191 GNKEAPLVVE----------------------
Tthe_EZL1 186 QEEKKQQVQKKIISKFIKN-------------
Otri_Contig14971_0_g38 175 VIKQPDGTLVSSPFHLRFGKLKVLKSSDK---
Amac_AMAG_02871T0 177 SPAAASSRRKRARK------------------
Spun_SPPG_03589T0 202 TK------------------------------
Atha_MEA 195 KEARPAR-------------------------
Atha_CLF 189 NVTPSVGRPKKLA-------------------
Atha_SWN 189 DSAITHRRARKHQSH-----------------
Mbre_estExt_fgenesh2_pg.C_20049 169 SVERDQEEEPVDWLDIDWVQPLSDFTGVTMAV
Dmel_Ez 192 GIEREMEIV-----------------------
Lgig_estExt_Genewise1.C_sca_1470059 175 GIERDADVP-----------------------
Nvec_estExt_GenewiseH_1.C_5680002 190 GIERDVDFALR---------------------
Aque_Aqu1_209566 --------------------------------
Hsap_EZH1 195 GIERETDVL-----------------------
Hsap_EZH2 193 GIEREMEIP-----------------------
