# Supplementary material for: Local Effect of Enhancer of Zeste-Like Reveals Cooperation of Epigenetic and cis-Acting Determinants for Zygotic Genome Rearrangements
Source: PLoS Genet. 2014 Sep 25;10(9):e1004665. doi: 10.1371/journal.pgen.1004665 (PMC4177680; doi:10.1371/journal.pgen.1004665)
Supplement: Table S1 — SET domain proteins identified in the somatic MAC genome of P. tetraurelia. Accession numbers (see ParameciumDB, http://paramecium.cgm.cnrs-gif.fr/), names and conserved domains are indicated. (DOCX) [file pgen.1004665.s014.docx]

| **Paramecium SET domain proteins** | **Paramecium DB Accession numbers** | **Domains (CDD-search + SMART)** |
| --- | --- | --- |
| Ptet_EZL1 | GSPATG00037872001 | **SET**: 459-571 |
| Ptet_EZL2 | GSPATG00032888001 | **SET**: 466-575 |
| Ptet_EZL3a | GSPATG00012695001 | **Metallothio:** 467-509 **SET**: 555-661 |
| Ptet_EZL3b | GSPATG00013305001 | **Metallothio**: 460-509 **SET**: 555-661 |
| Ptet_EZL4 | PTETG1700020001 | **SET**: 553-659 |
| Ptet_SET1 | GSPATG00013040001 | **zf-HC5HC2H:** 347-417 **SET:** 704-821 |
| Ptet_ASHH1 | GSPATG00013040001 | **AWS**: 28-62  **SET**: 73-187 **PHD**: 223-271 |
| Ptet_ASHH2a | GSPATG00003275001 | **AWS**: 67-127  **SET**: 138-252 **PHD**: 288-336 |
| Ptet_ASHH2b | GSPATG00004957001 | **AWS**: 67-127  **SET**: 138-252 **PHD**: 288-336 |
| Ptet_SETa1 | GSPATG00014017001 | **SET**: 283-399 |
| Ptet_SETa2a | GSPATG00025368001 | **SET**: 120-242 |
| Ptet_SETa2b | GSPATG00031547001 | **SET**: 306-414 |
| Ptet_SETb1 | GSPATG00018768001 | **Bromodomain**: 133-183 **SET**: 422-544 |
| Ptet_SETb2 | GSPATG00035094001 | **Bromodomain:** 138-190  **SET**: 421-553 |
| Ptet_SETc1 | GSPATG00025951001 | **PHD**: 40-80 **SET**: 196-308 |
| Ptet_SETc2a | GSPATG00033097001 | **PHD**: 41-73 **SET**: 245-357 |
| Ptet_SETc2b | GSPATG00036078001 | **PHD**: 36-82 **SET**: 254-366 |
| Ptet_SETd | GSPATG00035182001 | **SET**: 296-402 |
| Ptet_SETe | GSPATG00021145001 | **SET**: 19-135 |
| Ptet_SETf | GSPATG00024000001 | **TPR**: 142-238 **SET**: 303-466 |
| Ptet_SETg | GSPATG00031631001 | **SET**: 156-230 |
| Ptet_SETh1 | GSPATG00003961001 | **SET**: 175-270 **Rubis-subs-bind:** 299-429 |
| Ptet_SETh2a | GSPATG00027281001 | **SET**: 182-270 **Rubis-subs-bind:** 299-429 |
| Ptet_SETh2b | GSPATG00020579001 | **SET**: 175-270 **Rubis-subs-bind:** 299-429 |
| Ptet_SETi1a | GSPATG00015535001 | **TPR**: 13-111 **SET**: 153-359 **Rubis-subs-bind:** 395-531 |
| Ptet_SETi1b | GSPATG00012103001 | **TPR**: 13-111 **SET**: 153-369 **Rubis-subs-bind:** 393-522 |
| Ptet_SETi2 | GSPATG00016863001 | **SET**: 342-388 **Rubis-subs-bind:** 423-545 |
| Ptet_SETj1a | GSPATG00037631001 | **TPR**: 161-194 **SET**: 251-383 |
| Ptet_SETj1b | GSPATG00010387001 | **SET**: 249-402 |
| Ptet_SETk1a | GSPATG00038239001 | **SET**: 34-253 |
| Ptet_SETk1b | GSPATG00007235001 | **SET**: 34-133 |
| Ptet_SETk2a | GSPATG00012599001 | **SET**: 34-527  **Rubis-subs-bind:** 610-692 |
| Ptet_SETk2b | GSPATG00013397001 | **SET**: 45-107 |
| Ptet_SETl | GSPATG00012308001 | **SET**: 34-450  **Rubis-subs-bind:** 526-609 |
