# Supplementary material for: Local Effect of Enhancer of Zeste-Like Reveals Cooperation of Epigenetic and cis-Acting Determinants for Zygotic Genome Rearrangements
Source: PLoS Genet. 2014 Sep 25;10(9):e1004665. doi: 10.1371/journal.pgen.1004665 (PMC4177680; doi:10.1371/journal.pgen.1004665)
Supplement: Table S2 — Sequencing and mapping statistics. Read statistics are provided for the samples sequenced for this study, with the exception of the PGM and DCL5 silencing samples, which were previously published [2], [6]; European Nucleotide Archive Acc No. ERA137444 (PGM); GenBank Sequence Read Archive Acc No. SRX387766 (DCL5). (DOCX) [file pgen.1004665.s015.docx]

| DNA | Insert size (bp) | Read length (bp) | Reads | Aligned reads (MAC) | Aligned | Genome coverage % | Average Coverage |
| --- | --- | --- | --- | --- | --- | --- | --- |
| PGM | 341 | 108 | 130,266,728 | 110,189,786 | 84.59% | 99 | 161x |
| EZL1#1 | 159 | 74 | 113,316,836 | 89,872,888 | 79.31% | 99 | 89x |
| EZL1#2 | 328 | 74 | 102,580,084 | 81,824,588 | 79.77% | 99 | 83x |
| control | 212 | 74 | 79,063,468 | 77,712,438 | 98.29% | 99 | 79x |
| DCL2/3  DCL5 | 318  214 | 101  100 | 99,744,888  90,105,744 | 92,247,446  87,728,998 | 92.48%  97.36% | 99  99 | 130x  122x |
|  |  |  |  |  |  |  |  |
