# Supplementary material for: Local Effect of Enhancer of Zeste-Like Reveals Cooperation of Epigenetic and cis-Acting Determinants for Zygotic Genome Rearrangements
Source: PLoS Genet. 2014 Sep 25;10(9):e1004665. doi: 10.1371/journal.pgen.1004665 (PMC4177680; doi:10.1371/journal.pgen.1004665)
Supplement: Table S3 — Sequence complexity of control, PGM, EZL1, DCL2/3 and DCL5 datasets. The previously published contigs assembled from a PGM dataset [2] were used as reference, representing the currently best available germline DNA assembly. However, only contigs larger than 1 kb were considered, representing 91 Mb of sequence complexity, of which 89 Mb are covered by read mapping above our cutoff (i.e. 2 reads per kb of contig per million mapped reads in the library). Reads from each sample were mapped to the PGM contigs using BWA with default parameters, to determine the complexity of the contigs covered by at least 2 reads per kilobase of contig per million reads in the library (RPKM), giving the first row of the table (“PGM” Reference). In addition, a set of PGM contigs were selected that had control (KLEB) coverage below the cutoff of 2 RPKM (“PGM not KLEB” Reference), representing pure germline DNA not collinear with MAC chromosomes. The coverage of these contigs by each sample was also determined (second row of table). Note that 76 Mb is the complexity of the MAC reference genome [25] and that the total germline complexity is at least 10 Mb greater than 91 Mb. Although the N50 of the PGM assembly that we have used as Reference is 28,076 bp (meaning that half of the assembly is contained in contigs larger than 28 kb), the analysis presented in the table uses the 7,310 contigs greater in size than 1 kb out of a total of 30,013 contigs, a choice dictated by the necessity of obtaining good paired-end read mapping to calculate coverage. (DOCX) [file pgen.1004665.s016.docx]

| Reference | PGM | | EZL1 | | DCL23 | |  | | DCL5 | KLEB | | |
| --- | --- | --- | --- | --- | --- | --- | --- | --- | --- | --- | --- | --- |
| PGM | 88,996,911 bp 100 % | | 88,959,057 bp 99.96 % | | 88,430,111 bp 99.36 % | |  | | 78,067,454 bp  87,72 % | 76,088,297 bp bp  85.50 % | | |
| PGM  not KLEB | | 12,908,614 bp 100 % | | 12,870,760 bp 99.71 % | 12,349,370 bp 95.67 % |  | | 2,025,155 bp  15,69 % | | | 0 bp  0 % |  |
