# Supplementary material for: Local Effect of Enhancer of Zeste-Like Reveals Cooperation of Epigenetic and cis-Acting Determinants for Zygotic Genome Rearrangements
Source: PLoS Genet. 2014 Sep 25;10(9):e1004665. doi: 10.1371/journal.pgen.1004665 (PMC4177680; doi:10.1371/journal.pgen.1004665)
Supplement: Table S4 — IES retention analyzed by PCR and deep-sequencing after control, DCL2/3, EZL1 and PGM silencing. Maternally (mcIES) or non-maternally controlled (non-mcIES) is indicated as “+” or “–“ respectively, based on previous studies [11]. Star(s) following a retention score indicate that the IES is significantly retained (cf. Materials and Methods and the legend to Figure 6B). NA: not analyzed. No retention score can be calculated for (i) the mtA IES since it is retained in the MAC reference genome [13] and for (ii) IES51A1835, IES51A4404, IES51A2591 and IES51A4578 because the control strain carries a maternally inherited MAC deletion of the A gene. 1: S. Duharcourt and E. Meyer, personal communication (DOCX) [file pgen.1004665.s017.docx]

|  | | | | **DCL2/3 RNAi** | | | **EZL1-RNAi** | | | **PGM-RNAi** | | | **control-RNAi** | | |
| --- | --- | --- | --- | --- | --- | --- | --- | --- | --- | --- | --- | --- | --- | --- | --- |
| **NAME** | **length (bp)** | **mcIES** | **PCR** | | **Ret. score** | **PCR** | | **Ret. score** | **PCR** | | **Ret. score** | **PCR** | | **Ret. score** |  |
| **IES51A1835** | 28 | - | - | | 0 | - | | 0.03636 | + | | 0.80478 | - | | NA |  |
| **IES51G1832** | 30 | - | - | | 0.02128 | - | | 0.05263 | + | | 0.7337 | - | | 0 |  |
| **IES51G-11** | 43 | +^1^ | - | | 0.0396 | + | | 0.48421 *** | + | | 0.79512 *** | - | | 0 |  |
| **IES51G1413** | 52 | - | - | | 0.00943 | + | | 0.24468 ** | + | | 0.78307 *** | - | | 0 |  |
| **IES51A4404** | 77 | - | - | | 0.01316 | - | | 0.05376 * | + | | 0.8287 *** | - | | NA |  |
| **mtA**  **IES51G2835** | 195  229 | +  + | +  + | | NA  0.14706 * | +  + | | NA  0.42222 *** | +  + | | NA  0.77193 *** | +  - | | NA  0.00538 |  |
| **IES51A2591** | 370 | + | + | | 0.43373 | + | | 0.52482 | + | | 0.9102 | - | | NA |  |
| **IES51A6649** | 370 | + | + | | 0.38017 | + | | 0.45113 * | + | | 0.81333 * | - | | 0 |  |
| **IES51A4578** | 883 | - | +/- | | 0.06329 | + | | 0.45882 | + | | 0.77844 | - | | NA |  |
