# Supplementary material for: Local Effect of Enhancer of Zeste-Like Reveals Cooperation of Epigenetic and cis-Acting Determinants for Zygotic Genome Rearrangements
Source: PLoS Genet. 2014 Sep 25;10(9):e1004665. doi: 10.1371/journal.pgen.1004665 (PMC4177680; doi:10.1371/journal.pgen.1004665)
Supplement: Table S5 — Anchois retention score. The 28 IESs that were used to identify the Anchois Tc1/mariner transposon [2] are provided with their ParameciumDB Accession Numbers, size and retention scores in the different RNAi datasets under consideration. Retention scores followed by a star are statistically significant. The DCL23_r sample is a biological replicate previously published by [6] that was retrieved from the Genbank Short Read Archive (Accession number SRX387766). (DOCX) [file pgen.1004665.s018.docx]

| **ANCHOIS ID** | **SIZE** | **PGM** | **EZL1** | **EZL1_r** | **DCL23** | **DCL23_r** | **KLEB** |
| --- | --- | --- | --- | --- | --- | --- | --- |
| \| IESPGM.PTET51.1.103.177611 \| \| --- \| \| IESPGM.PTET51.1.104.49056 \| \| IESPGM.PTET51.1.105.239361 \| \| IESPGM.PTET51.1.120.182371 \| \| IESPGM.PTET51.1.128.254421 \| \| IESPGM.PTET51.1.132.167159 \| \| IESPGM.PTET51.1.163.702 \| \| IESPGM.PTET51.1.169.56908 \| \| IESPGM.PTET51.1.173.70900 \| \| IESPGM.PTET51.1.174.130670 \| \| IESPGM.PTET51.1.181.1750 \| \| IESPGM.PTET51.1.214.11549 \| \| IESPGM.PTET51.1.24.100577 \| \| IESPGM.PTET51.1.28.457973 \| \| IESPGM.PTET51.1.29.290535 \| \| IESPGM.PTET51.1.35.111752 \| \| IESPGM.PTET51.1.42.397702 \| \| IESPGM.PTET51.1.42.72890 \| \| IESPGM.PTET51.1.47.408041 \| \| IESPGM.PTET51.1.50.348282 \| \| IESPGM.PTET51.1.51.131273 \| \| IESPGM.PTET51.1.57.48117 \| \| IESPGM.PTET51.1.76.220822 \| \| IESPGM.PTET51.1.77.209216 \| \| IESPGM.PTET51.1.77.311405 \| \| IESPGM.PTET51.1.80.84925 \| \| IESPGM.PTET51.1.85.45587 \| \| IESPGM.PTET51.1.98.309432 \| | \| 2462 \| \| --- \| \| 2238 \| \| 2483 \| \| 1769 \| \| 3392 \| \| 1500 \| \| 1956 \| \| 3272 \| \| 2714 \| \| 3001 \| \| 3001 \| \| 2317 \| \| 1340 \| \| 2468 \| \| 1722 \| \| 2820 \| \| 2473 \| \| 3389 \| \| 2125 \| \| 1251 \| \| 2219 \| \| 1257 \| \| 3048 \| \| 4154 \| \| 3470 \| \| 1513 \| \| 3479 \| \| 2003 \| | \| 0.82379 - * \| \| --- \| \| 0.82759 - * \| \| 0.84783 - * \| \| 0.83333 - * \| \| 0.88199 - * \| \| 0.75281 - * \| \| 0.82353 - * \| \| 0.86447 - * \| \| 0.93431 - * \| \| 0.83908 - * \| \| 0.57576 - * \| \| 0.78161 - * \| \| 0.81928 - * \| \| 0.84577 - * \| \| 0.74214 - * \| \| 0.72131 - * \| \| 0.84231 - * \| \| 0.84135 - * \| \| 0.83525 - * \| \| 0.85714 - * \| \| 0.85926 - * \| \| 0.78212 - * \| \| 0.83857 - * \| \| 0.87264 - * \| \| 0.83682 - * \| \| 0.79227 - * \| \| 0.84965 - * \| \| 0.88776 - * \| | \| 0.47959 - * \| \| --- \| \| 0.53153 - * \| \| 0.6 - * \| \| 0.5678 - * \| \| 0.68182 - * \| \| 0.55556 - * \| \| 0.59091 - * \| \| 0.55556 - * \| \| 0.95 - * \| \| 0.5098 - * \| \| 0.4875 - * \| \| 0.56962 - * \| \| 0.51064 - * \| \| 0.48718 - * \| \| 0.54321 - * \| \| 0.42424 - * \| \| 0.56 - * \| \| 0.58209 - * \| \| 0.61702 - * \| \| 0.61667 - * \| \| 0.54945 - * \| \| 0.36232 - * \| \| 0.65152 - * \| \| 0.56579 - * \| \| 0.47059 - * \| \| 0.53608 - * \| \| 0.61702 - * \| \| 0.62821 - * \| | \| 0.49682 - * \| \| --- \| \| 0.54918 - * \| \| 0.5 - * \| \| 0.42308 - * \| \| 0.73171 - * \| \| 0.5 - NS \| \| 0.5122 - * \| \| 0.46667 - * \| \| 0.88235 - * \| \| 0.43011 - * \| \| 0.39474 - * \| \| 0.56452 - * \| \| 0.40449 - * \| \| 0.6 - * \| \| 0.45545 - * \| \| 0.45455 - * \| \| 0.50667 - * \| \| 0.40541 - * \| \| 0.53846 - * \| \| 0.58333 - * \| \| 0.47059 - * \| \| 0.64 - * \| \| 0.5 - * \| \| 0.63158 - * \| \| 0.45833 - * \| \| 0.52778 - * \| \| 0.55844 - * \| \| 0.72619 - * \| | \| 0.04132 - NS \| \| --- \| \| 0.32353 - * \| \| 0.26126 - * \| \| 0.15306 - * \| \| 0.39604 - * \| \| 0.1413 - NS \| \| 0.19231 - * \| \| 0.01887 - NS \| \| 0.34783 - NS \| \| 0.39735 - * \| \| 0.05085 - NS \| \| 0.29496 - * \| \| 0.2314 - * \| \| 0.20213 - * \| \| 0.33588 - * \| \| 0.04511 - NS \| \| 0.04167 - NS \| \| 0.28986 - * \| \| 0.39167 - * \| \| 0.47761 - * \| \| 0.23256 - * \| \| 0.08333 - NS \| \| 0.38255 - * \| \| 0.33071 - * \| \| 0.32117 - NS \| \| 0.12712 - * \| \| 0.29167 - * \| \| 0.61667 - * \| | \| 0.07692 - NS \| \| --- \| \| 0.48352 - * \| \| 0.32927 - * \| \| 0.11111 - NS \| \| 0.75728 - * \| \| 0.32812 - NS \| \| 0.47761 - * \| \| 0.04396 - NS \| \| 0.67647 - * \| \| 0.76647 - * \| \| 0.03636 - NS \| \| 0.80303 - * \| \| 0.30263 - * \| \| 0.22951 - * \| \| 0.78102 - * \| \| 0.0396 - NS \| \| 0 - NS \| \| 0.66667 - * \| \| 0.67153 - * \| \| 0.72436 - * \| \| 0.34314 - * \| \| 0.16667 - NS \| \| 0.70526 - * \| \| 0.5873 - * \| \| 0.60204 - * \| \| 0.25773 - * \| \| 0.50485 - * \| \| 0.80405 - * \| | \| 0 \| \| --- \| \| 0 \| \| 0 \| \| 0.00685 \| \| 0 \| \| 0 \| \| 0 \| \| 0 \| \| 0 \| \| 0 \| \| 0 \| \| 0 \| \| 0.01053 \| \| 0 \| \| 0 \| \| 0 \| \| 0 \| \| 0 \| \| 0 \| \| 0 \| \| 0.01266 \| \| 0 \| \| 0 \| \| 0 \| \| 0.05128 \| \| 0 \| \| 0 \| \| 0.01136 \| |
