# Supplementary material for: Local Effect of Enhancer of Zeste-Like Reveals Cooperation of Epigenetic and cis-Acting Determinants for Zygotic Genome Rearrangements
Source: PLoS Genet. 2014 Sep 25;10(9):e1004665. doi: 10.1371/journal.pgen.1004665 (PMC4177680; doi:10.1371/journal.pgen.1004665)
Supplement: Table S6 — Oligonucleotides used in this study. (DOCX) [file pgen.1004665.s019.docx]

| **Name** | **Sequence (5’ to 3’)** | **Application** |
| --- | --- | --- |
| bigNd7_5' | GATATACGTAAAATGATTGAATTGGTTTAC | PCR around ND7 gene |
| bigNd7_3' | TTCATAAGTTAACTTCTTAACTGATAATGC | PCR around ND7 gene |
| tel51G-01up | GATGTTAGACATATTAAGAGTTTGTTAGAC | tel51G probe |
| tel51G-02down | CTTAAGAATAAAGCTATGCCAAAAGGAAGG | tel51G probe |
| EZL1_ATG_F | AAAAGCACCTAAATGACCAAAGAATAAAT | RT-PCR EZL1 |
| EZL1_R5 | CAATATCCTCTGTTAATTTGGTCGG | RT-PCR EZL1 |
| EZL2_U1_S | GGACTAGTATTACAGCATCCTGTCTATAAGATTTACTG | RT-PCR EZL2 |
| EZL2_R1_S | GGACTAGTCTCTATAATGTAGCTTTCTTCATCCAACAAC | RT-PCR EZL2 |
| EZL3a_U1 | GCTCTTGTGAATTATCCATTTAATAATGC | RT-PCR EZL3a |
| EZL3a_R1 | ATGAAAATATTTGCCTGGTTTTGCAATTC | RT-PCR EZL3a |
| EZL3b_U2 | TAAGTAGGTATCGATTAAATGATTCTG | RT-PCR EZL3b |
| EZL3b_R2 | TTAGAATATTGTTTGCTTTATATGAGAA | RT-PCR EZL3b |
| EZL4_U1_S | GGACTAGTCGCATTAATTAATAATCCATATAAATCTG | RT-PCR EZL4 |
| EZL4_R1_S | GGACTAGTCAAGTAAAAATTTGATTTGTTCTACAATTA | RT-PCR EZL4 |
| PGM_F2 | GAAAGAGGTACAGGAATGTGAATTC | RT-PCR PGM |
| PGM_R2 | GCAGTTTCGGATCATCAAACAAGGATC | RT-PCR PGM |
| T1b_5'(2)b | TCTAATTAAACCAAGAACACGCTGAATTC | RT-PCR T1b |
| T1b_3' | TTGAGTTGGGATTTGACATAATCGGTGAA | RT-PCR T1b |
| 51A4578-2 | TGGTTGTTAGTCTCAAAGAATTCTAAAGAC | PCR for IES circles |
| 514578-7 | AAGAAATTTTATTGTAAATATATTTTCAGC | PCR for IES circles |
| 51A-1835-5'(3) | GGTTGCGTAACACTTCCTCTTAAATGTGAG | PCR around IESA1835 |
| 51A-1835-3' | ATCCTAACATCCTTGAATAGTTACTGATCC | PCR around IESA1835 |
| 51G1832up | GCTATAACTCTTGAAGCTGCTTGTAATATG | PCR around IESG1832 |
| 51G1832do | TTGTCAATGAGCCATTAACAGTTGCTGGAT | PCR around IESG1832 |
| 51A-4404-5'(2) | TGGAATAGTGCTGCATCACCAGCTGCTTGC | PCR around IESA4404 |
| 51A-4404-3'(2) | CCAGTTATTGAACTGCAACTTACTGCAGTG | PCR around IESA4404 |
| 51G-11up | ATCATAAGATTGATATCTTCTCCCTTCTCC | PCR around IESG-11 |
| 51G-11do | ACTTGCTACTAAAGCAAGAAACATTGAGAG | PCR around IESG-11 |
| 51G1413up | GAAGCTGCTTGTGTTAAGAATTCTACTGG | PCR around IESG1413 |
| 51G1413do | GCATCCAGCACTAGTTGAATTTACTGTA | PCR around IESG1413 |
| mtAF6 | GGTGTTTATATCTTAATTGTTGACCCTCAC | PCR around IES mtA |
| mtAR7 | CCATCTATACTCCATTCTTTATCTTAATTCAT | PCR around IES mtA |
| 51G2832-6 | CAAATACTGGTGGTGCAACATCCTCAACTGC | PCR around IESG2835 |
| 51G2832-8 | GAACCATTTAATGCGCAATATCCTGCATTTCC | PCR around IESG2835 |
| 51A2591-18 | AAGTGCAACCTGTGCTGATGCTCCCGATGA | PCR around IESA2591 |
| 51A2591-20 | AGTTCCTTTGAAAGATGTGCAAGCTCCAGA | PCR around IESA2591 |
| 51A-6649-5' | AAATGGTACTGTTTGTGCTTGGGATAGTGC | PCR around IESA6649 |
| 51A-6649-3' | CAGCAGTACATCCAGCTCTCTAAGTTTAGC | PCR around IESA6649 |
| 51A4578-5' | CACTGCAGTAAGTTGCAGTTCAATAACTGG | PCR around IESA4578 |
| 51A4578-3' | GTAGTCTTAAAATCTTAGCATGTTGTACC | PCR around IESA4578 |
