# Supplementary material for: Local Effect of Enhancer of Zeste-Like Reveals Cooperation of Epigenetic and cis-Acting Determinants for Zygotic Genome Rearrangements
Source: PLoS Genet. 2014 Sep 25;10(9):e1004665. doi: 10.1371/journal.pgen.1004665 (PMC4177680; doi:10.1371/journal.pgen.1004665)
Supplement: Text S1 — Accession numbers and sequences of SET domain protein used in Figure 3 and Figures S5- S6 (94 pages). (DOCX) [file pgen.1004665.s020.docx]

***Allomyces macrogynus* (Fungi)**

>AMAG_02693T0 | AMAG_02693 | Allomyces macrogynus ATCC 38327 SET domain-containing protein (581 aa)

MSKSSAPVPARNGSRKYGGRWEYVPLDAPSPPTTRAEKIRHFFRYSPIPGVPSDGDDNDDDDDDDASNASHSSASSIPAWMNLRNWNPTVLSPAEVAMGRWLEQRHAAQLARSAVRKRVAKRKRLPVRPPPPPRRVSGNDSDSTLSTADDSGSDDDRSSHRTASRAGSASLLPVPDTWRKCIKLHTTADGVVWLHMRGTRANESLTDPDEPRPAHILDCGLFAESLPCLPVPPNLPERPGWRVVAVAKDHLLPLPLPLPGPYRWLFEQRDFVLPPALLAVGNHLKRMRKNKEKWTPITKNRFVSRPRIRRYEHHPCSCPKGSKCGPLCLNRCMYIECEPDTCPAGKSCTNQRFQKGEHVEGLEVRETENRGKGIFTTVPIAKDALILEYTGEIIDEKTCHARMDVTYAQARHFYFLCVGNGDIIDGTRYGSIARFINHSCAPNCRVEAWTVNGDVCMGIFADEDIAPGSELFYDYNFEDFNARHRQYCFCGAEECRGMLGERREPVEPRVVLRSVVDRRMHAGADLRSLLKSKKTLANVKESRLFLIRNLETVAAAARRSKPDMERPASARSDRAKLANA*

>AMAG_02871T0 | AMAG_02871 | Allomyces macrogynus ATCC 38327 conserved hypothetical protein (631 aa)

MGRARGTRSDATVERDATTSGAAQTNSLTHAPTSSPATWIFPVVTYSPPPTVWPRKPPQHVYTKILHGVYAVPDDLVVRWWPDPSATAEADADLGFRHYKENYAHHLTAHKDLVLVRSGMVDVVAHALIRAFPELVTHVATLDAARSTLLWTPTKSDPPIFQSLEKFMERQQPGLASHVWRWVSATTTNPGTRRRSRSASNVTHSGSAHGDLVPAPATNRHQPDAALSGSDLPCSATCCKASPLAPTPPAPALSADQIALIRDSLATGLTPCDVAALIPVPCRAIADQVAAAIVPGPAGDDAPGSASDSVPRRTSLTRRRASTPSRRASSPPFTSARSAHWVPVNRGTGGKKKTMTMSFADGEADPFLPRSRRFVEPAHLPLHRPCSHPGEPCTLATCSCLEAEHFCDTLCGCASECPNRWPGCDCVDFQDDTTCRDSSECLCVQWMRECGPACACPCRACKNREIGKGVDHDKKLRVDVSTIPKAGWGLFAQCDFKADDFLGEYSGEVLSSDEADRRGIIYDRKSLNYCFQLATDAVVDAYRLGPVLRFCNHAAKSNVNARVAFVDGSQRIGLYAKKNINAGDELFLDYGRMYWGDESGTPKAAASTSSSRSSPSSPAAASSRRKRARK*

>AMAG_03278T0 | AMAG_03278 | Allomyces macrogynus ATCC 38327 Suv39h1b protein (459 aa)

MTPHWTGLPEFVSLTTASKTTKTMSTDAQAMTRNLCRKTIRPVSLNLCRKTSKPISRNLGRKTCKPVSHNLCRKTSKPMPHNLGRKTSSKPVSNPHTTAATIRSEYGASGSRKRRKFDSACDDARRDGEDAHSDDKKLAKDPSGLISLDRASISPTTIFPPRSFSNTSTLLYSPHASAFLPPVTALPACSFARGTCASPTTPCSCTAAGDAYYAPHGTTMAYTPTGRLRDVLDLPHDALRTLHVLECTARCKCGPACANRVVQRGSTVPVEVVWDDAVGWGVRATRNVEKGEFVMQYLGRVVAEDECSGVEGRYEFALDLDEGRGEGWETRPMLVVDAEEVGNVGRFLNHACDPNLLVVAVRFDSLVDSGLYRIALFARRDIAAQEPLCFDYEGGGPDLPAGARTKKGDLSFRCRCGSANCRKIIFPLLVGGDEESDGVESDHDVDSNEDELSYLSGR*

>AMAG_04063T0 | AMAG_04063 | Allomyces macrogynus ATCC 38327 predicted protein (437 aa)

MDPQGVSIKWVSDAKERGVLADRDFAEGNVIFEEQPMVCDQYLYNKDFFPTCEYCLACLESPADMVKRLAGLPEPPHLWGAEAVPVLKNVPCEHICGELYCSTSCRDKAWAQYHSLLCLGPHPRDDHPLLEIRQEWKSFHFPPESATIGLLVKMLAMIAVTRDEALFDHFKADYRNETLHIVAKFMDEQFSDRLTKFLRLFRDLFAPHGLHVSQELFDKLLTIVSLNGQGIGTSSFEAYERILRNLSAAAEEKAGSPEALAVTEALDQVDALREAIEEHSDDFTHAEGTGLYRAHAMINHACVPNAEIQFVRNNATLRVVATRDIAKGAEVTISYMHFDGCESDGEEDEDEEESDHEHDHHGHDHHHDHDEHLVDVDRRREMLREYYLFECTCDQCLADSGAGKAALATAPGSRTVAGSSSSSSSAAGSSSSAARR*

>AMAG_04388T0 | AMAG_04388 | Allomyces macrogynus ATCC 38327 predicted protein (661 aa)

MSGTSAPSSTATPVLQRPWVLATLAVAAVGIGYAAFFDYKRRADPSFRRNLRKQRKRVEKKKTEEDSAKARDVAASGGAADGLEAEIEALLNEPLPTTPEAKEAFFMEQLSKGETLAKNGPEFFKLAAAFFYKALKVYPAPAELVMLYQKTLTEPVFNLVMGFISSEVKKRQEQYFVSFPDASMNVRVGEEPDPKRKAPDGTPLKRRTLIAAKDFEAGETIYEEEPIVGTLNPAFLQSGEHCAHCFKPTACDGKVESTRTGATYCSKACEDIAWETYESLLFADYARPGGSTTTTLVAECATDGVIVPLHMARFLAYMVHEDTKKAENPDADGFGVWDHIERLRYLELTPTAIDAKYITMLQTLLGEKVPGLEDFLTLERYLLLKGKFLYNQMAVATRQVEALVAAEAAAEAETTAEAKPEEPAAEEAEKPTEVIAAAADPAPELDVESLADDADDQQADDSPAALLEEEDTSSAIVVEVEKSEDDESAASGDAQDVAADAADAETPAAEEPAAAEETPDASTDAIPEATVEEAEEEEEVKSKEDEEKPKVAVLEPERSASAAAASTAAALYPVSAYLTHSCTPNVEATFPRKSRTLALVAKTPVRAGDVLMTAFVDLALPYADRKAALGAGWRYTCECGRCAAEAPKEEEAEEEAKVDE*

>AMAG_05234T0 | AMAG_05234 | Allomyces macrogynus ATCC 38327 predicted protein (389 aa)

MFAVRDIEPNEPIVQVPRNLLVSRTAVLSKWCPVLTKSPHAARLSEHCVLTAFLVLLKLQAPCVKLSVHEFWAPYLATVPAQFTNLFFDDAVHNCEFMPQDAKRRLDTQIRTVRTDFAAFTAVQKDLGDQHLLPSTFDVPAATFHWAWFAVNSRCVSLQMLPTAGSGRRSTPTKSADTMALAPFLDMINHSPTTTVTVVAPPNSPYYAMHTGQSWSRGTEVFIHYGDHAPWDLYVEYGFAMPASNHPVSVRNFVERSFPETDERFAWRWRALRNVGLLGGAHDDQLLVLPVSFVLDLVLRLMFLPDKMCRGGIASPGYRAWSRRYYEADEGEPVADEEPLVRKWVAAARRKALTQRETYLAAAAVESVTDTLRVILDDEVSVLRSNVE*

>AMAG_05437T0 | AMAG_05437 | Allomyces macrogynus ATCC 38327 predicted protein (546 aa)

MSSRIQNRRAEYLNAVENVLQKIATCGQLALPLGVKPDIEWQYFEELNDIQVELATIQADADKLLRSKNAVIKEPAPLSQRLDAFVDWLHRNGVSRDVPVQFVSGLNEGIGAIAKRDIAEGEELISIPRSVLLSLVTAYESKRMRAAMTSVPNLAKLPAHLLLVLHLVDEKNDPNSFWKPYIDVLPETFALPVFFDPEDMTELQSTTVFRETIEQLRSMLNSYVQCFRIVEASKLMPIHKFTWSTFRWGLGVLQTRQNMIPAYTVEEMEGSNGQRTPEVKALALIPGWDMLNHREGLPITSESNIQDGSQNCTSPAAYKAGTQVFIAYTTHRPNRHRFLHSGFLGDVPRSVAGDFVRIMFRFPALKDVDERKTLARTKFGLQEPFPLDLCVPIANNYDNVIGTLACAVGTPADLAWMQDKLEDLVDEATSREVYARNARTVSRAFKAAIGDATASQDAYVLECSVKIINYVKLQATMLQRRLKDTAASDTAALKKLVNVKSVRDQFHKNVLQMRAVERIIAQDLVADADKVLQKAQAAAGSKASS*

>AMAG_06039T0 | AMAG_06039 | Allomyces macrogynus ATCC 38327 predicted protein (344 aa)

MDETALITASPGGEEPLRAALLSQYPTMPAATQARIQRLFSGLLDHDRFGSAPPTLSDAQARGVVRFNTFAPFDHVAMTTGNDASIVPTTGLWLSPSFLNHACSHNAFWHTPTLDRMVVRTVVPVAKGTELTIAYRNAHDPFALRALALRERYAMTCMCPVCTADRRVSETDRAARSADMERLSALATNVFEWAAEVDMDAADDEAARGIAEHLDQWLVAHAGSETYRPHDVRYIQYLLGCVELYVDVDNAGGRSRAADRIVTALVPTDLRVQWFDEEVRGSAPDWLFDLIRCVRTAHAFSAQMPVRAQQALLEHAAKLHDVMFGGGEEGFRAAFGELAVVEE*

>AMAG_06329T0 | AMAG_06329 | Allomyces macrogynus ATCC 38327 ASHH4 (597 aa)

MSKSQVLVNGNRKYGPKWEYVPLDAPPPPSTRTEKIRHFFRYSPIPGMPSDGDDNDEDDTSNASHSSASSIPAWMDLKYWNPTILSPAEVAMGRWLEQRQAAQQARSAVRKRVAKRKRSPVRRVLPRRVVPGTDTDSTLSSADESDSEDDRSLHRSASRTGSVSLLPAPDTWRKCIKLQTTADGVVWLHMRGTRANESLTDQDEPRPAHILDCGLYAESLPCLPVPPNLPERPGWRVVAVAKDHLLPLPVPEWGVFEQRDFVLPPALLAVGNHLKRMRKNKEKWTTITKNRFVSRPRIRRYEHHPCSCPKGSKCGPLCLNRCMYMECESDTCPAGKSCTNQRFQKGEQVEGLEVREVRRSLPLTISGPLHITDDSLPSSSAQTETRGKGIFTTKPIAKDTLILEYTGEIIDEKTCHARMDVTYAQARHFYFLCVGNGDIIDGTRYGSIARFINHSCAPNCRVEAWTVNGDVSMGIFADEDIPIGSELFYDYNFEDFNARHRQYCFCGAEECRGMLGERREPVEPRVVLRSVVDRRQHAGADLRSLLKSKKTLANVKESRLFLIRNLETVAAAVKRSKPDVERPASARSDRTKSANA*

>AMAG_06505T0 | AMAG_06505 | Allomyces macrogynus ATCC 38327 Mll1 protein (1282 aa)

MPPAKTQGRVTLADLVDAGLLEAGDQVTFDNHIATVQANGELYVGEPGSIPVYLQPTYQSPSSWCTDVRRRGRESSATVSGWIKARLAADRSRTLDSVRRQYMETIHPQGVPGTDATPPPPGGAYHGRTRGAAPETDYAADVNAYLDEDEDDEDDDDVVRAPTSAIRRRHLVEVPPHDDDKDADFQAGEQRKKKGRSRPSKAGTAAGSPAVSAGAAGSAPPSKKAKVLDGPGSGKVRTLSAGAHRSTTPAASSAVAGGALGLVPAAGAGPSAAAATTAELERKVEEPMPVPAVAILPLTTASTDPDADIFRAATSCVDCSAVLHADEEYLTCASCTEAHHPSCTPLYICPPPATARDPWICIYCTFCSKCNKSTPRDQLAKCSGCQHVFHLDCVHVSPDLIGADGTIFCNNCAECFSCGTKPRGNVALAPPAWDRHPAKNAYQCQSCSASYHADNYCPVCLKAYPEDDFETPMVGCDGPCGKWVHKACDPLLSNIHYDELHATNHEYVCPRCRTPPASEADPDEVILFPWQRRARTPDPAAAAIADALATTAITDATAAPAAAAPPAAADHDMHDAHNDPINNEPSMAEIALRTYMSGRECAFCHQTAAVPSGIGRLLFVGGPDPLSLGLGQWVHTGCLHCTCSNLDCVHVSPDLIGADGTISATTAPECFSCGTKPRGNVALAPPAWDRHPAKNAYQCQSCSASYHADNYCPVCLKAYPEDDFETPMVGCDGPCGKWVHKACDPLLSNIHYDELHATNHEYVCPRCRTPPASEADPDEVMHVDDVPAVAAPAAAPAAQIVSQISRTVTPPPDMLNGDDGADKMAVDESIPATSPDMVMTADAVTATVTAMQPVTCALCKRGSANVRCQSASCKTAYHWACLVRALFTEVPAKAGYGTAADLKRAHKVHLDVFQKHFRCATHATPGTANTAFRPYLPRLADCERVCQMRFAIQQPNTVPLYVSAAPAPQPGQLLTTAPPYDPACLVAGGAIVTHIGRLPFADPNWLMDRTRMPPVLVPVGYTVVRRFWSYKRPGTVTSLVISRIVDGDDGDDGDEHALASARALEAASIDEGTVVQGESIPDRTIVRHHADEPSAAGNASTLVPPPASATKPAAPAGATARGDAVAPPAPTVWETTIPQQLQYQKLKASEHAHCTVKPSKIQGVGLFAERSFQAHDMIIEYLGQVVGQKVADARELVNDRRGIGTYFFAIEHDRILDATTCGNAARFINHSCEPNCYARIVAPVQGVRRVVIYALRAVHAGEELTYDYKFQPEEDVSKRIRC

>AMAG_06513T0 | AMAG_06513 | Allomyces macrogynus ATCC 38327 predicted protein (614 aa)

MPPSPRRHAPVRRIEHEHTLCLDFLATLRARTAVPPPRPRPVSPPPPVVVPAAPPAPVPVPPETASPPVPLPPATMPPPHSTSASKPARATTLIVIPSSDSEDELPVRPPPPPSAVNNVDVVQPAPAPTPVSPTGPSRRTAARILPQKRRHPSPSPLLPTPTPTPPQVPPASAPPVPILDDFDEPGDVVVLAPRAPRTAARLAKHARHEPAHLPPRAQFPTSKLPSRPASQPNARLPAPKPPAAAVQPLPPAVQPPTTPPQARARLPGSTATAAANGAKGVAAGENGTGANGTPAVSRMNRTPVKQAWRPFGDDHTERHTIDSFRAEIAKETATTIAIDLTGVTPECLPPKSWTYVTDLTFAPGADAPDPAFLAGCDCDGKCRLSFNGVVPCECVALLDELTDGKLRGARSLLAPYKASGQLRVLPKAAHEMVELIECNPNCMCDMATCPLRVVQRGPVSIQVGLKFMPRKGWGVYATAPIPPGTFVAQYIGEVLHVTAVRNSAYLFDLDYFTREGHQYMIDAAQKGNFARFLNHSCSPNLAQAIVLYDSHNLDFHRVAFFTRRDIARGEELTFDYTGGVPDQDAGAVANGAGDAPVFFKCECGAEGCRGMVN*

>AMAG_09124T0 | AMAG_09124 | Allomyces macrogynus ATCC 38327 predicted protein (559 aa)

MTNVTTTAPAAPPARTAPAPLFAKRKVNKGHLRKKTSDAATAPVNDVDQDKSAVQSPQAPGAAAVYSTAESLHKLTINDAATSSDAKQSQPATTPTAPPSDADAKGARIIDGPVSPEFVAYFRSCLPPSTKATDLTIELVPNKRRGLIAHRAYAVGDLLWREDPVVALPFAEEADSECAWCFATAAPNGKLLRCTACAAVMYCSRDCQRRDWSLHKHECPHFKTKKDYPIAVRFFARMMRLREAKATTPTGHDWWSLVEELHWAPLEHAEAENMARAIFAAAKLVSRDTLPSPSAAMQFMSILQTNGLTVHHTSDLARKADGLYVATAMANHACDPNAICVFSGRSVTVRCLRPIAEGDEVTINYVDVCQPEPDRQRDLKHFHFTCACALCVRERADAVGKGAKQKVEIRTLADHDRAVADPTIDPRLLQEPRRHLISQLMARPDTADYAAVARLATALMDTYAQYLGSIHPVTLFHVPPALKAQVHLLDERPSSLAEAHAQIQSAQALLAGFRQWQRDVKVWGYAGEDIRAMDVDTAVQALQQFVGMLEMGLRSRAF*

>AMAG_09338T0 | AMAG_09338 | Allomyces macrogynus ATCC 38327 predicted protein (345 aa)

MDETALITASPGGEEPLRAALLAQYPTMPAATQARIHRLFSGVPNHDRSGPAPHTLSNAHAQGTVRFNTFAPFDHVAMTTGSDASVVPTTGLWSSPSFLNHACNHNAFWHTPAPDRMVVRTVVPVAKGTELTIAYRNVHDPFALRTQALHDRYAMTCACPVCMADRRVSESDRAARSADMERLSALATNVFEWAAEVDMDAADDEAARGIAEHLDQWLVAHSGPETYRPHDVRYIQYLLGCVELYVDVDNAGGRSRAADRIVTALVPTDLRVQWFDEEARGSAPDWLFDLIRCVRTAHAFSAQMPVRAQQALLEHAAKLHDVLFGGGEEGFRSAFGELAVVEEE*

>AMAG_11220T0 | AMAG_11220 | Allomyces macrogynus ATCC 38327 conserved hypothetical protein (418 aa)

MLLGPSSRPGSSELHAPRPAIALGSSPTDGIPPSLRPLFPVRRRTPPPPDAHVRPDDEVYNMPLTTDARLPPPRTDAERAALATFELLTESVITRPALGRAEGAGDYPCMCRYNPDADPLATACGPHAQCINRQLFVECVPGVCPVGKKCQNRRILTRQSAAVEVVQTAQKGFGLRALEALPAGAFVLEYTGEVISRSMFLRRAKAYSALGHRHFYFMSLQKDEIIDAQRKGGLARFINHSCNPNCETQKLEETQDFLKQYTAYQKIQATTPQTPAYALNDSPVGKDLVMDAPRGRDQPVTWRADAMLNNTALCDLPRVTDGLQEDGIEYSTVFPGQLGRTTSTRRKTSASVDYAASTAYAVSAKRYRGAWDSPSSSAVRDPRSGFPPPPPPPAAVHDANTMSVALLRNPAASYRAS*

>AMAG_11310T0 | AMAG_11310 | Allomyces macrogynus ATCC 38327 conserved hypothetical protein (897 aa)

MDPSAARAPVPAPATPPSSASLPAHPPAATAGPTPPAAYAWPASAALNAPSAAAAAVVTASGAEPVVVLPTAAPAFPPPPSHHPHPHSLSQVLAYPYTAGPRHATVPPVMPSSSPSSSPAVPTARRPTAGGPAAARAPNGSKRPRTSTALSAGAADGHDDAPARKMPKLASSSPSLSSLASLTSPVPAGAASAPYLPGSGPSGPPGTGPTTATASPGGTTPVIGRSRNPSSSGVYSLPPPLPLHPDEEAGLEDTGEIRCICGYTDDDGYTIQCDRCLVWQHASCVGIHRDNEPEHYYCDRCQPRELDVRKAQLSQRRAMLARAREVAAHTGGPPVGGAPANGAGTMNGGGNAPLPPGVALPAFATVASDGVQSPVPGRRRAEGKSASGKRGGGNAGASASASASSGAGGNANGNGGITSNGTSGAEKKPASSRGRKRTVPATGGPSNGPGKSASTDPAASAILPPLPPSVARLKSHPSSLHADRSAHVPPFLATHSRGPSTSSVRIEPGDDTEVEENGSGTEADGYRSPAFARSSPTAPGVALPPPPVPPSAIAMPAALVQHTLPHLQGQRVNLDEHVHPAYSLLNQDYSIGLTANRISPVHAPYVQQLVAQAVPDPEVGLSVVVSAADDPFLHLHQQVTAPSLRTRSMSEATVCLIVLPANDPPPHLVPPPLPYAAPEHVAAVQAAVAASNAQSRSGRGGAGARSRQGSASQPDASATVPVQIASLCPPLEPNEGTNDAVALHINTTATPVPAGRAVLEYMGHLLPASQHPHMLSPFSLQVPGLPLIIDASRESNAARFCRRSCRGNLSPHTYVRRTPDPASPLAVALVANRDIEPNEALTLAWSPADLAWLASSAQCKPDPNHFLLFDAPATAQGPAHAAAAGRDRLRLGRCVG*

>AMAG_11375T0 | AMAG_11375 | Allomyces macrogynus ATCC 38327 predicted protein (676 aa)

MAGQPRPIAGADHGGMEQHHQQSHHGAGSVDRPEKRARLEPIDYDAEPAEADYRRLEKWILDNGGNLDNICLRKSETSEGFGVFAKSNIPAGADVAFVPNHLILSETDALASDLGRALLAYNATIPLDEYPAQHVCTKTILWLFIVHEFFNKGRASMWFPYLRALPRSYDTPLFWPERERAWLRGTNLHFVVAEKEAELQADFAAAKVVILDRFLEVWGDTSTLTFANFLWARTSCSSRAFPSDLRVVEQGRTKESKDDDVAGIGRDGVHAVIQPGPDGKYPPCKCFLALWPLFDMLNHRRGQSMMWDATRDNGIWFTTGVAMTKGEEVFNSYGPKGNDELLLSYGFCLDIPNNPDDYVPIKINFAQDPLRDRKESFLTHCNLLPFKSLFLLRYADLVPATLVACMRVLVANATELARIETGAVDVITTNVSHRNEMTVWDTLVSLLSHKCAVLEATSSDDDGDEIETALVPVEDAAAQGQFRARMALIYRSGQAQILAAALASAAQQLGASVEAASARLVRSADIADAAEFWDAAAPLIDHAAEAEWGPDEWLFAYLVWAWRAGKLPSDVDVSFAAERGNVQGRGDEDQDEDEDEALAPIFAVLAAAEPHDMGSVWNSVETDVRAVFEWMQDHSVTLMNVQTAASEDNDDDDDDEEEEEGCVNAEPLMGVVLEQ*

>AMAG_11412T0 | AMAG_11412 | Allomyces macrogynus ATCC 38327 predicted protein (733 aa)

MNGQQCDKGTPPSVGTGSSSPVPYFAEPPSDEASSTSTTPPASSSSSEQRATDRLMQLAEVALGLGIHMGAGDDGAMESTAQERTGLKQSTTSSSLSSVPTVESGPRKHAVDDVVMEDAHADSGTRQDKVPAAAGPDVAPMDVDTSPVASKANSVLSSVRSPVSSKASSVLTSVRSSPLSSVPDHLFNDAGLDSSFSSIDDSDEHDDPDHRPSSRRLPSIEVTRKDAVEIGAMTLVEFAKCTNMKLTITAYAMARCPEPSFRDLAAYDDVCTHILLDAMYLPFRVLKMDLADLAVANTTEATGKQPHHELPRQIVENPTEFPVPADDIAVVLRNVAEGKVSELDAVHMIIDQTAWLKKWLTGSVPIAQIAHFCRHMRRYVAMYTPAAGFDVVESRRYESAQPPAANKLRQAALVATRAYKPGEVLKLCSGHALALTPAEEDALQDAARDFSVLHSSRLNAFCLFLGPGRFANHDCEPNIDFVIRRRTLPPVYAAVIADVGDPPNTTTVPHASEIYMRATREIRAGEELTVSYGRHYFDRNNAACLCETCERTGRGQFAPERRSKAPDAASGDPAYASPAASSPLQSPPGSGGEEPAADASADSSVLRSPRMVRRAKVDARLHIRAEHKRDEPDMVGGLVVAPESDWDLDAPVQEGARLGSLRQFFHHVQDLGIGAKPRPAPRVPAVVIPPKCHKCLRHSLLFHVPWPQRTGATGESAPAFQPAHAPVLGPMC*

>AMAG_11461T0 | AMAG_11461 | Allomyces macrogynus ATCC 38327 predicted protein (433 aa)

MLVRSMRAGGFLTTTIASTDILPETLDTPVFWTDAELVHLAGTDVPKRTAKDESQSLFENVILPALREHVDAADLARFTPDAFFFAASLLMAYGFHVGPLHEPENPDDDESDVEEEDPLPVLVPLADTLNAVHPASAHLERDGDSVAMTLSRDVAGGAQIYNTYGDHSTNELLRRYGYVEWENEFDHVSIDGMAVVGHVMDALKKVGWAKGMSTNDKEKALAKRLEFLEEIEVFEDEFEVSVLRPVPADLVLTIKLLLLDPDQYKAAKKDPSLVFPLLSKFTDADPTQPIDRAVIYLTKTEAAVLHAAVAARLAEYPETPVAAEYLAAIEASTDLAGYLARVVPAMGNVRVAYAYVVRFREQRLLRQLLAALDAAEQIATWKDAKAHAKSAQETEGTRVVIVGQAYPLTPAKRAASGATAGSGKKEKKAKQK*

>AMAG_11915T0 | AMAG_11915 | Allomyces macrogynus ATCC 38327 predicted protein (289 aa)

MARAIFAAAKLVSRDTLPSPSAAMQFMSIVCDSLIQNNGLTIHHTSDLSRKVDGLYMATSMTNHACDPNAICVFSGRSVAVRCLSLIADGDEVMINHVDVCQPEPDRQRDLKHFRFTCECTLCVRERADAVGKTLKKKVEIRMLAANEDRDRAIDPRLVQESRRHLISQLMSRPDTADYAAVARLAAALMETYAQYLGPIHPVTHFHVPPALKAQVHLLDERPTSMAAAHAQIQSAQALLAGFRQWQRDVKVWGYSEEGVRVMDVDAAVQALQQFIWMLEMGLRSRAF*

>AMAG_13595T0 | AMAG_13595 | Allomyces macrogynus ATCC 38327 histone methyltransferase (1241 aa)

MVVHTPSSGAPFLPTPDPPHGSASAPSPPPWAAGTFARPPSSHSMRRDVRDDYYDARGRSPHGSGPRGPYPPEYRDRDWDRDRDWDRERDRDRDRAPLIDSYRSAPLPSRGPSAVDRYDPRDRDRDRGDWPPIRDRDRYPPPPPPGPRGAYPDDDWDALPPPSRMIDRYRSSDRDRDAPLPPPSRTRSYSGARPPLDEYPRSRSSSGILPEPVAGGSLLLSPTPGVDIDPLPAPTPMPVPTPVPEKYVPPPPAPFGEMWKALGDPAKQSKKEPPRRTNGVMVLASWVLRSSGSCWDDGADAIFRLSSPPRPSRGCSSRTGSITARYCARISSVTYLPTFVTVANLDPTLDPAGVRAMFAECGPIEYTRVELHPANGMSLGIARIKFHSTAATNDFGRDGYTRSPTALHERDGDSDLPTDVNRANVPHARPGSLLAPTTCTPRTCGPRAPPALPRATATRNAGKPANGTTRSPPLRRHERELYSNDALVPPYLRIANLPLHVAAHKLRALVSSLGPRRVAVVDGVWVITFADPDNVVRGYQTLDGKRHEGYVLRAHVVRGDDGAPNGPAGAVPPKSAWDRDADVAKTRLRIDARKAVWDRVMAEHLLDAIKRRVHARVAAAGGGAGDDLEDLALTDVRAPPRTATLDNGVTVTSDSHLPTPATTSDHPVPRARAATDASLDRAILALPSFKVRRSDARRRGRDLSDDESDTDDARRARRKRLRRGRSRSPRDDSDGDSLAAARPAARRRRAMSWSTSTSISESEAELLALGTTGGARRRSTTARKRRNIDFDSTSEEDGDEDDDEAAGDSDAVSAVPPAGDNEADDGVMPAAAVVPRAKPKSAKAAAAARRPSPGARLAEPQPFSALESQLEDTLASLGADLAHDGLASSWPNPDRITWVGHGARDEYKYMYVALQERYAALPASPEKPLAARDPVIAAPPPPLMLPPAVAVPDDPIVAPLDRLGLGPSRDAQGKPGSPTPWSAAADMAGAASMDPGAAKWSTCSRTMGVRKIPFDAKVHYLPRIPPLTPQERHQALLAAGTSTRAGAAVLGVLGALGHDESGGAAAAASGSVPDAGTSSSTWVVEGTMSVSTLKARAKRLKIAKSGIHNWGLYALEPIEAGDLVIEYVGEVVRQKVADTRELQYEREGLGGSSYLFRLDDDRVVDATKTGNYARFINHCCDPNCIARIIAVDGRKRIVIYAAKPIAVGEEITYDYKFDFEDDDAKIPCLCEAKACRGFLN*

>AMAG_13838T0 | AMAG_13838 | Allomyces macrogynus ATCC 38327 predicted protein (669 aa)

MAGQPRPISADHDGNMERHRQQSQHGAGAVDRPEKRARLEPIDHDAEPVEADYRRLEKWILDNGGNLENICLRKSETSEGFGVFAKTNIPAGADVAFVPSHLILSETDALASDLGRALLAHNATIPLDEYPAQHVCTKTILWLFIVHEFFNKGRASKWFPYLRALPRSYDTPLFWPEHERAWLRGTNLHFVVTEKEAELQADFAAAKAMILDRVPQVWGDVSTLTFANFLWARTSCSSRAFPSDLRVVEQGRTNESKDDDVAGIGRDGVHAVMQPGPDGKYPPCKCFLALWPLFDMLNHRRGQSMMWDATRDNGIWFTTGVSMTKGEEVFNSYGPKGNDELLLSYGFCLDIPNNPDDYVPIKINFAQDPLRDRKESFLTHCNLLPFKSLFLLRHADLIPATLVACMRVLVANATELAFIETGAVDVLTTHVSHRNEMTVWDTLVSLLSHKYAALESSSPDDDEADSGYVPVEVAAAQGQFRARMALIYRSGQAHILVAALANAAQQLGAAIEAASDRFVRSADIADAVEFWDAAAPLIENAAEAEWGPDEWLLVYLVWAQRAGKLPSDVAVGSSAAKRGDVEDGEDEDEDEDEALTPIFAVLAAAEPNMGSMWKGVEADVRAMFEWMQDHSVTLLNVQTAAAEDDDDGQDEEEGGMGADLLMGVVLEQ*

>AMAG_13870T0 | AMAG_13870 | Allomyces macrogynus ATCC 38327 predicted protein (768 aa)

MWLKKWLTSTVPIEQLAHFCRHMRRYVAMYTTAAGFDVVESRRYESAQPPAANKLRQAALVATRAYKPGEVLKLCSGHALALTPSEEDALQDAARDFSVLHSSRLNAFCLFLGPGRFANHYCEPNIDFVIRRRTIPPVIAAVIADVGDPPGTTTVPHASEIYMRATREIRAGEELTVSYGRHYFDRNNAACLCETCERTGRGQFAPERRAKAPAAAAATGGELAYASPAASSPAQSPPGSGGEVPAAAASADSSVPRSPRMVRRAKVDARLHIRAEHKRDEPDMVGGLVVAPESEWELDAPVQEGARLGSLRQFFYHVQDLGIGATQRPAPRVPAIVIPPKVTCYHCQAQTYTFELSRRGVVECHKCLRHSLLFHVPWPQRTGAAAESRRRPAFARTLLCWVELHLTGQTIVDKEAADRGRGSGKNARKGKDEGRAPTPPSPMLEPMDDASARRAAGKKGVGKAKSGKKRSAEEDIAQALAQIAALREIPPEWRHLPREIQDENGRTLNLNERNLLLCEDTGTWYPAVSLPVSDWVEEGFAPSPEHIAVRYLHARDLQYGFVDFQHVYSVDQVLFEAEGIYLVASIQSPSPSSSSSLASASSSASTPARGRDVRMLLSVNQDRALRCIAMPTRTKLLWRKWADEMHVLKQLSHPSMPYRPVANFDLANLTFPPVMTRSGAPSSVVSSGTSTPSASMRGKHNAPLERVRTYRASDVGPRSPSLRVRDGDRGSSPLTSLSPASDVAEPRYSSDLPTTRTTRASKRRKLE*

>AMAG_13919T0 | AMAG_13919 | Allomyces macrogynus ATCC 38327 predicted protein (538 aa)

MSTDNTSSSSSSSSSSSSTFDDLNQRFLTFLSSSGATISPDLAIKDYSTEFAGRGVAATRDLPAGEMLFTIPLDLCLSYESARVSEWVRDHPALFDNGWAPAILAVLYEFVNRDTSKWKAYFDILPETLNTPVFWTDAELTHLAGTDVLKRVAKDESQSLFENVILPALREHVSETDLARFTPDAFFFAASLLMAYGFHVGPLHEPEIPDDDESDVEEEDPLPVLVPLADTLNAVHPASAHLERDGDNVAMTLSRDVQGGAQIYNTYGDHSTNELLRRYGYVEWENEFDHVSIDGMAVVGHVMDALKKVAWSTRMSAKDNEKALAKRLEFLEEIEVFEDEFEVSVVRPIPADLVLTIKLLLLDPDQYKAAKKDPSLVFPSLSKFTDADPTQPIENAVIYLTKTEGAVLRAAVTARLAEYLETPIATEYLAAIEASTDLTAYLARVVPAMGNVRVAYAYVVRFREQKLLRQLLAAFDAAEELATWKDAKAHAKTVQETETTGARVAIVNQAYTLTPAKRAASGAASGSGKKEKKAKQK*

>AMAG_15442T0 | AMAG_15442 | Allomyces macrogynus ATCC 38327 predicted protein (530 aa)

MPPPNGSTASRPARTTTVIEIPPSDSEDELPVLPPRPPAAVNTVNAVHPAPVPTPVSPTGASRRTPARALPQKRRHPSPSPPPPTPTPTLPQVPPAPAAPHAPILDDDLDEPGDVVVLAPRAPRAAARPAKHARHKPVHSPPKARLPTSKLPSRPASPGSTAATFQQPTTPPQARARLPESGADANGAKGVAAGANGITTGANGTPAASRLNRTPVKQAWRPFGDDHTERHTIDSFRAEIAKETATSIEIDLTGVTPECLPPKSWTYVTDLTFAPGVDAPDPAFLAGCDCDGKCRLSYNGIVPCECVALLDELTDGKLRGARSLLAPYKANGQLRVLPKAAHEMVELIECNSNCMCDMATCPLRVVQRGPVSIQVGLKFMPHKGWGVYATAPIPRGTFVAQYVGEVLHVTTVRNSAYLFELDYFTREGQQYMIDTAQKGNLARFLNHSCSPNLVQAIVLYDSHNLDFHRVAFFTRCDIAMGEELTFDYTGGIPALPGQDHGAAVANGAADAPVFFKCECGAEGCREMVN*

>AMAG_15452T0 | AMAG_15452 | Allomyces macrogynus ATCC 38327 mixed-lineage leukemia protein (94 aa)

MIIEYLGQVVGQKVADARELVNDRRGIGTYFLRLSTTAFSTQRRGVRRVVIYALRAVQAGEELTYDYKFQPEEDVSKRIRCRCGASKCRGYMN*

***Amphimedon queenslandica* (Metazoa)**

>Aqu1_201094

HFMCARKKDCRFLSNKEVYCQQHSSLPVTSDTVKDEDMRVDRCIIIHTDNDRPGRKFTKSFPPQNIQLRIGSLLIRSAGSLSPLSDTISPSLLIPHSFSSSSLFWSLTTPTQRITYTLTVSADLPKEEPLPLTTPTPETPPTSPGDLRWPCVSTQVSSDPDNEGVWSAVSSKSQILSSPSLINNSVTDKSNPEIALRIFRIPKKTRSSSSSPPPTLAIGLTITAPLPVTSVHCPQSHREEETVRPVLPLDSPALNQWETGSPPHSVNSVHYELSNEEGQSWRGPDLKGSDTNNDNNDDNNNMYMYMLVLLFGRSY*

>Aqu1_201602

MWCRVLSDLSALASGLVAKIAHSAFSLVRYFQRLDRKLVRINQIDHAAPYYVVDATCKGNKIWFANHSVNPNCYAKVKIVEGDHRIGIFAKHSIEELFLITDLSPQLLQAFIYQLKMFKEQR*

>Aqu1_202766

MSRRSYLEQKPTSKNSFSLSLLSIDGADGREYLTYRIDVCIFCGNHYECFAPAYFHVKGRPNITSDDSNKVVITGNIDKDIHLLPTGEDIQLATEGMGVKVIVNRTGCARSEGYNGIRSESDMFSFLMSQWRPDPPKPDPEALDMAVIQAECQGNSNTHHQPQNDVPVAMHYRACQWMYRENVGVFGSHIHGLGLFCLQEIDSGDMVIEYAGTVIRSTLTDYQERFYESKGIGCYMFRIDSDEVVDPNCYSKVVAVDGQKIMIFALET

>Aqu1_204472

MNYEIIDQCVPGPGVQLKLDHVLYESCDCHVTCQKEECPCIKKCSYDEYGRLSQELMSETTCPIFECNSKCTCTLDCINRVTQRKGTVFDKVMVKETSNKGDGLFAKVDIPIGTFVGEYVGEIIRVLTAKERLQSLCPQDSCYIVQFKENSEGSDVIMTTCIDARFYGNYTRFINHSCSPNLVMVAVRRDSIVPSLCLFTSAAVGAGEELCFSYCEGSETDEVSLGQKPCFCGSTNCRKYLPLQSS*

>Aqu1_205506

MDKNLIGMEKKVSETFLTLKEQSHDECKEEKAECIEEKEDLKKDIQRLSKQATDLNLEYTKCDSERPAHAECKEDKRKCLQETRDLNDLNEQLKRDKAALDVKYTKCDTERVAFDQCKVDKRELKRINKNLEADLKALNIEAITLNITHSSENKELTICDRRLHVCKVNMLDYLGRYETCLKDKGNTKTESQKNDVICKVELAKCEANLRQAIDTSSQCQEKLASCRWC*

>Aqu1_205646

SSNYHFMCARKKDCRFLSNKEVYCQQHSSLPVTSDTVKDEDMRVDRCIIIHTDNDRPGRKFTKSFPPQNIQLRIGSLLIRSAGSLSPLSDTICPSLLIPHSFSSSSLFWSLTTPTQWITYTLTVSADLPKEEPLPLTTPTPETPPTSPGDLRWPCVSTQSNPEIALRIFRIPKKTRSSSSSPPPTLAIGLTITAPSPVTSVHCPQSHREEETIRPVLPLDSPALNQWETGLPPHSVNSVQYELSNEEGQSWKGPDLKVLWESVVSELQDARHKLDLPFVPFFPDNVYSQFGLSNHLVLQLLEQLPDHEKCSRHNFIYYQPEKVSNWQQKRVIVNRTGCARSEGYNGIVPGEELTYDYKFPIEEAKIPCKCGSARCRKTLN*

>Aqu1_206359

MEAIPALPRYSVWCPIQENFLVEHETVLHHIPYMGEEVLCKEDSFIEDLIKNYDGKIHDGATETGETVEDDVLQDLVEAMKKYTVLPKGTNRFPGCRCRSSCSTKHCPCFLAVRECDPDLCSTCGAERVHTKRSEEASADDSIRCCWRGIFLKDGAEKNEFISKYCGEIISQDEADRRGKVYDKYMCSFLFNLNNX

>Aqu1_207609

MKLAKELEKKENRRMKALKKKEHKGETKKGKENQSEPKEKHKVTFATVGSNSQFDTGETLSESDDDTICPKCGLAYSADHDSSNLWICCDKCDVWFHLKCTIVKSKWTIPDTYFCEECQNKRCQITSEKRI*

>Aqu1_207682

MCKVWSKVTTPSSKNSISASSGSEPVPLPVNHLSGIKSLTNLTTPPKVNIVSGWKRQGQAKTSTSSDLNVSGSGGGVAGGGVEGETDKEETLPDGWNGQELILFRMLRPIYCHNYCSMAEVIESKTCQEVYSYASTLPLDPILNQHPSRPPGTSQKRKNMRFGLARIQYHRKIKRVAGQATYQFNYQPCYHPGLPCDQECQCVSSNNFCEKYCNCNPDCELIIIMRASPTVLRL*

>Aqu1_208650

MEEEGIKIRKCNTRLRSNTKALPSTVSEALHQGLLNKENDRTDLCTVLDIEPGEKGVFAMVNIAAHTFLCKYVGDIVRGESNMRDIEDMYKRKGINKFYVFQIDECTFVDATERSDSIGRFFNHLPANKCNCIAAKENDEIGIVTLRDVKMGEQLTYRLVIAMVNKTQQISNSDNAGTRKRSRKFRDIQAQRYGIFDKSINS*

>Aqu1_208740

MREEGLDVKVSGVSKLIKKYMETGSITRRPGSGRPSKPNRYFLESRVLTASQLCSMPPKLKSKSGTTATGNKQTSTSKGKDSGSSGVSGAQSGSHSTVNCVCGSPIDSGHMVECESCSCWSHVQCIGLSPSCASTYPYICPFCVRLLVTNLSELRSEVAFLRNKIESIESSFSTTSTSSISSEIESINDQLRLLSSRVDSSPDTSQTQTNTSETPSRPVSNHTVNHDGSRRGNLVITGLPECPQGTTRVDRFLKDQETVASILSTVSPSVNSQSVKDLFRLGRYRPDQSRPILVKFFRSWDATVVLRNKSKLADSPNISIRPDLSPSQKTARSLLLRERRTLMTSGNNAGDIKIRGNKIFLRDSLYGEVNNNSFVRALAPGDSDTSNTTDSATVSSTLPPSRVSPPVTPSPPTVTPPSFQ*

>Aqu1_209566

TNRFPGCRCRSSCSTKHCPCFLAVRKYDPDLCSTCGAGDNLEMKFTMCKNVSIQRGQKKHLLMAPSDVAGWGIFLKDGAEKNEFISEYCGELADFGLAHSLASTSNEGSKGVGGDGVNDFNPAMTDYVATRWYRSPEILLGSRRYTRGVDMWSLGCILAEMIASRPLYPGSSTINQLNRIMSTLPPPSRQDVESIKSPYAKAILDQIIHKRHKYLTDILPQADPSKITHYTIDDSTNRFPGCRCRSSCSTKHCPCFLAVRECDPDLCSTCGAGDNLEMKFTTCKNVSIQRGQKKHLLMVLSDVAGWGIFLKDGAEKNEFISEYCGEIISQDEADRRGKVYDKYMCSFLFNLNNDYVVDATRKGNKIRFANHSVDPNCYAKVMIVGGDHRIGIFAKHNIELEEELFFDYR*

>Aqu1_210505

MYKLCFLGNIERESLTSVCKNLESQLASLSSEFLLASLRSLAKAVGLSSMHCCVAMHEYMMARPVRTVSSQATFKSVAEEDLRESKEPLDLVTVEHITANELGVFAKCNIEPKTFITEYKGELLSKEEAARKEDLYKLIKQDKFFLLDIRPNKTLDGMNAIGTIGRQMNHAPKPHCNCMGVIVHERVAIVSERDIAQGDQLTWDYGLRSKDLPWLKTYSSIARAKNEDILLRLHQKSRRRFTQCPLCLSMVESSQSSEHLHRQHHSLTDVDLKYYAAEMLSVKHITKDASQGRKRGGSTRGMEEYAVSEATPPMVKQYIESVVFILIVIKLPIVTIATHRYSAT*

>Aqu1_213191

MGLISGTSMDGIDVCIIKINISDSSRDDILDFSVVDYMTVPYPHGLKEDLLNIGINGRTRDVCLYHSLLGRLFSDAAKAMMEKLHIKSSDIYVIGSHGQTVLHLPNPVQLYGHSINGSLQLGDPSIIANHTGVTTVGDFRTADMALGGEGAPLVPYLDFILLKQHFMETKRVGVFLNVGGISNIFSGNPHEGMNDTDMECIGFDCGPGNVLIDSLMQIFYGREFDQDGTVASEGNINDSLLSWLMKDKFVLRSPPKSTGRELYNADYVHEIVKKCKDDSISNKDCISTVTAFTACAVHYNFKQYVQALLNDQAESDIDLFVSGGGYRNNALVKCLRDCFKPVKIVSTDELGIPCDAKEVSYYEKFELILVQAACFAVLAYQAVAGCKNHLPSPLSLASFHPPPQSRSVISSRSSSGSSSPSIPSHLAPKPLLIPALSSLPTSSLSTNHAFQPIDNGHSDSRSRFRRSPTPGPWGKMLSKNKNQKVKDDVDKEKEIDTWVITDVVESDPVTSPRIKLKRVSKTDSSSPSLSPPKIFIASPEKSKGEEIHIIEDEDGRLVTDEDFDGSLVCFTSQSGSSTPPLLVPFSPSPPPSPSSSSLSDGSSPLPSFSRFLYSPAIDSPCPSLPSLPSPCPLSIHDKDKPIVLDTYLNSQKNDKIKLPHPPPPILKYDTTTLNGTAASDCSSTSPPITRRATSRINSIHHTETAKDERASRSVTPTPVFSLESVNTIAPPNSYYYNILLLWQGSLNSARGSEAPIFIENLYDNEPPPVNFKYITSSIYSTNVPVPNITALVGCSCLNCSESVDCCPQLAGQKAAYTKDKRMKAARGTPIYECNFMCSCSSTCYNRVVQFGRQFPVCIFRTRNGRGWGVKTCSDLKRGTFVTEYVGEVITTEEAERRGVTYDREGSTYLFDLDFDEDHPEFTIDAGHCGNISHFFNHSCSPNLQVFSVWINTLDTRLPQLALFAKKDIVAGEELTFDYQMSHNLAGHTRGKGRVPCLCGSTDGLYGEIYGDDSGLKMAAFDDKKMLLAHIRHSFVTSDDTGMCELIMIKEDVEQEIENDHSYQKKSLRMQQRYKQAEKRSTNNMAASYDIQASPTLSRRPPLKNTSDMFQIMEKKKASRVRTVQWEITPLELVDRSLFAPQPIRPHPTGPSPLKQLLAEQDKKKSNPFYKYSKFNGENHGPASSNKFLMFLSMAKDPLYPVEVTVLHTATIQDLVGVTFWKYYEEGRKPFYLKGIESYAVMIADDDGEIDTDLPELQGDDYISKFGFKYLGIVERSNPDDVKIEDVPAKYKIVKVYYENGGFSTIAISEDDVEMSEILARVIRKRRLQIHGYELEKKDSPGEFINLRSSLSSQGTLDFMLVRNTRQGHTPSSSVEDDVDSPDVGTEVTSHLYTSFSVTMLHRMRSNTRVQLGIDPQRERVEIQPIESLKGFLAKPLKPMKYDINEISSVDILEEKPPNAVFRIVRLTKEGSVKNIDFEAPHNITKKIVAKLKLIISDSPSSIKSKQK*

>Aqu1_213265

MPKLKVPGENQKPRLHSKKLSGAKKIALKTRLNNKRETTRDDKGKKSSLLGKRKNGTLRLSFSKTFGASDDEDCDWEGFTIEDIENAEHKKEEGAPLENGLLMETDSLDSLPFISPSSSKRVHALSHTPNDDPALSDDHSPVRLLKRRRRCLECKACLRENDCGKCTNCLDKPKFGGPGSRKQACIHRKCLNLLREAAVKSSQSNKRVQSSDSHAPPSKRPLREDGGGGPMPSSLRPRRILPSSQSKTKDNQPVIQLYWPPADSIEYGYPVYTTSLSPSLPPSLPLCTRCGSLGSESFLYCRICCQPYHWFCANVPQTTPTFTCQQCITCRICGKPNKCVRCCECNEWYHEDCLLGHTSRPRTDNKRIDDSGRSLLSSLLATPTQWSDGYCYCSDCIVLKAKGNSCPVCGECYLDNDFDSKMVQCSQCDNWVHSHCENMTDEEYEILSDLPDSVEYVCRLCIAYNRTVKEDKKRRREGEGEEEEEVEWRAAVNGFKHEAYEKMISSIKTALGPIWVNDINVLTPLLSVNEFANRISEQLSKCFSGIKTGSHKHRLLFSSQQSYNKELEKLFPWFNMEMSDAVKNEAPPTDTPIADDINDIENWLSSVSHDHCYVSHDKKGTIKQEEGKRKEEEEEERGTVGKDVPADNRFCLLCTLKGDQSSTDAGRLLPCGGMDEWVHVNCALWSAEVYEDTNGYLCSVHAAIGRGLRLKCDLCHLHGATVGCCHSSCSSNYHFMCARKKDCRFLSNKEVYCQQHSSLPVTSDTVKDEDMRVDRCIIIHTDNDKPGRKFTKSFPPQNIQLRIGSLLIRSTGSLSPLSDTICPSLLVPHSFSSSSLFWSLTTPTQRITYTLTVSADLPKEEPLPLTTPTPETPPTSPGDLRWPYVSTQVNDSDNEGVWSAVSSKSQILSSPSLINNNVTDKSNPEIALRIFRIPKKTRSSSSSPPPTLAIGLTITAPSPVTSVHCPQSHKEEETVRPVLPLDSPALNQWEIGSPPHSVNSVQYELSNEEGQSWRGPDLKVLWESVISELQDARHKLDLPFVPFFPDNVYNQFGLSNHLVLQLLEQLPDHEKCSRHNFIYYQPEKISNWQQKRVIVNRTGCARSEGYNGIRSESDMFSFLMSQWRPDPPKPDPEALDMAVIQAECQGNSNTRHQPQNDVPVAMRYRACQRMYRENVGVFGSHIHGLGLFCLQEIDSGDMVIEYAGTVIRSTLTDYRERFYESRGIGCYMFRIDSDEVVDATMSGNMARFINHSCEPNCYSKVVAVDGQKKIMIFALRRIVPGEELTYDYKFPIEEAKIPCKCGSARCRKTLN*

>Aqu1_213539

GQATYQFNYQPCYHPGLPCDQECQCVSSNNFCEKYCNCNPDCTNRFPGCRCRSSCSTKHCPCFLAVRECDPDLCSTCGAGHNLEVKFTTCKNVSIQRGQKKHLLMAPSDVAGWGIFLKDGAEKNEFISEYCGEIISQDEADRRGKVYDKYMCSFLFNLNNX

>Aqu1_213782

MSTSSTFLPSFLELKETEEYGRGVYSVKELSPGTDVIVGDPLAHVISSHEREMYCHHCLKKERSLKKCGQCKFVWYCSRACQREDWLYHKVECAGISRISPSIPTDTVRLITSLPKGAQWDMDILTTHAPRYNEELKQSIGEQVFGAKLLMSDSKEYNPEVAFSIACKMISNSFSILDWEMNSIGSGVYILPALLNHSCDPNVIALFEGPKMRLRTTKKIFENEQASLNNTLFMIFLFFQLFVSYTDLLETRERRQRHLQKYYLFSCNCPRCSSEFSGSLDEKLIKTAADHKDGKVFFRLFGRLKRKLNWAALESLASEYLTKHSSRLGDTNILIVKMRECLMDSYIEQQKWREALQVAVTLKXXXXTKHSSRLGDTNILIVKMRECLMDSYIEQQKWREALQVAVALKGQYELYYSTYNPFLGLHCYKIAKLLQQVSEDTAALKESMKLLDKAIGILSVTHGSTHHCVVEGKRLLYDTGMELTGRKVHV*

>Aqu1_214303

MNYEIIDQCVPGPGVQLKLDHVLYESCDCHVTCQKEECPCIKKCLYDEYGRLSQELMSETTCPIFECNSKCTCTLGCINRVTQRKGTVFDKVMVKETSNKGDGLFAKEDIPIGTFIGEYVGEIIRVSTAKERLQSLSPQDSCYIVQFKENSEGSDVIMTTCIDARFYGNYTRFINHSCSPNLVMVAVRRDSIVPSLCFFTCAAVGAEEELCFSYCEGSETDEVSLGQKPCFCGSTNCRKYLPLQSS*

>Aqu1_218109

GWGLKATCDISRYSFVMEYCGEVCSLEEFERRRNIYEKESRRHYYFMSLKTDEVQCYLL*

>Aqu1_219530

METDNGFMIRCGCSDQCSLSHCSCRQLTEDEGQGLTRPNKSNITDGYDNSNRLMSKLVSGLYECNKYCQCSSSCGNRVIQNGIKHKLMVFKTKDIGWGVLTLEDIPQGSFVCSYVGLIMNDEIANRTGLDFGDNYLAELDYIEVLEYAKECDGGISGTSSSEGSLVLSSSSNSDSDTSNQISTTGSNMSCSPLLNEDNDLIDEDGHYLVSLSSNTPQSGLSSYSIPLTRSFFNESHSYVIDASSYGNVARFINHSCSPNLFVQNVFVDSHDIRFPSVAFFAQSLIPAYSQLFWDYNYIIGSVEGKAVKCMCGSSNCRGRLI*

>Aqu1_219539

MATNEKAVMKEEKDPSKLTIKKEPDQGENKKRNRTRGKAVKNDKTEMKPITQYFPLRRSTRRVKSLMEVIETDGKGRGVVATRTFHKDELICEYSGELISYREAVQREQEYSKDSGIGCYMYYFEFQSKKLCVDATTDNKRMGRLVNHSKTKPNVITKLIPVEDHPYLCLMAARTVSPGEELVYDYGERKKDIIKHHQWLTS*

>Aqu1_221177

MGKKSRKQQESEAKQKLCHALSDKGVVRLIDSLLERTSKFSPGSTANPNASTWEEYDAIVKLVHAIEQRQGPSPMPLPPRKDSVDEFVTWLTSNGAKFEKVEIMECPGAMGYGLRARTSIKEEDKVLEIPNSLFFSKEKVMSVEMEGLVRLEPLLSAMPNLVLALLLLNERYSLSSFWKPYINVLPNSFNLPLFFNKEDLLELQGSPVLMECISQVRNIARLYAILHKQLRAFSNFSLPICTVGFHLIDFKWAVSCVMSRQNKVPVSRSLSHLYKENQLVLIPLWDMANHTQGRILSDFDTDTQACISYSMSDLSPGDEFRIFYGPRGNGDMILNQGFAVEDNEYDYVKIWIGVSSASNDCIKRMVKRLRFTMLSTFSVAQGDSPFNEELIAFVRLLTTPEEEMKKWEEEKRTDEQIKEILNRKAWVQGDQRVMEFLEKRCQLLQLPYKTTIEEDKELLASDRVTGRTRNSIILRLMEKLILKNASEVAASLKAKLEGDGVEEIAWKGTNDAKKLKKDDDKLEETKDEGVTASTENGKLENGKMEEIMKIEETELPLKESKNTDDAEQEEADSK*

>Aqu1_222866

MFILINDYYIALGTATVTSKESPESTITTVAGGRGVKSTRSTAAVTSKEGPKSTVTTVAGGRGVKSTRSTATVTSKESPESTVTTVAGGRGVKSTRSTAGNKDNLYESFEKRSVRFDLLASDSITECPTCGAVYGEDESIWICCDNCDVWYNYECTGLQNEIPDEYFCSACQFSHVSVLRTEL*

>Aqu1_224220

MVKTKSLSSVEGSPHMSGCGLTTPTGTTPNSGISTKHAFDPILALKTCLLAKKNKVASIPPLPPKEKAPPLPSEAPPPPPEAPPPQIGLEEISSEDEGPKKVGKTFFEEISGEEGSPLVTRPQLIVEDISSCEEDGERGNGSVDMDISDTEQNSSQIIEINVRPLTAPAPHFIQPPLPPPPFPSQTFLNYQLSSEGPKTPPSEVRGDCLDNGGSAHPRTRKNKFSPAKQWKVPLAVTREEKRGQDVLYCALEQLSRILLRDVEKKLVESSAFPVLDEVWDKKESQRSKALGEEPMETEERVKPQEPYRRMLPGLVPNRSRPQEDDESTTPITPRKRLMNFKIPLVGSMYRRDNVMTATRRLHFNERGERDRRKRKNHYSDVLPRRKRRHRKEDKDERRSEYSQLSDISSEEWPSDEGRRSKDRDKKKKERKENAFGPRTPSATSEEEEEFNKLLQEEEEEEEGQEEEKVDSDSSLAFNEIFGSTSSSDFEADIASLRRPKKAMKLSEPAIKKPSDLIESELIATPPVKEDKEEMEIERIAEYKKRGEGEEAAILNSFLEEGFDKEDMDMMRSAYVKLKRIYSELISDVSWSYYPHNVVENNVTHSSGCARSEGYYKIDPSDKSRYLHHLRRSKASEDKDKNSAPPPTSSGRGNRLNHRRVASLFTSGDVSRELQQYNQLKARKKQLTFAKSTIHNWGLFALETIPADEMVVEYIGQVVRHGIADERERRYEAQGIGSSYLFRVDYDHVIDATKSGNFARFINHCCDPNCYAKIITVGNQKKIVIYSKRDIRAGEEITYDYKFPIEDEKIPCLCGAPQCRGTLN*

>Aqu1_224885

MEEENEEIEEEEEPLYVGQRDAEGNPNGRGTLKWLKSSRRFEGRFCSGMKQGRGCFYFKDGSSLSGTFRSDELHGEGTYTYPDGRLMIAQYVNGELDGPFTEYSETGDVMAKGEHREDRRFGFLQIFDEYGGILMGEVNRDTGELTGPGIAYVYPDKRSALLGRFSNSDMVCARPALLLTSVDQTPAKYALDENWSKTTLKLDESTSTCISCNPLTPDAYEQSKVYVSTCSDNPDKGEGLFAKTELAEGETVSFYNGLRLTHDEVDSRDWSLNANTITLDESMVLDVPLKYSSLDAYVASLGHKANHSSAPNCEYDHFVHPRFGHIKCIRTIKEVSKGNELTCDYGYSHKLPETDEEDLPEWYMTES*

>Aqu1_224967

MEPGDEVTCFYGKNFFGDNNAFCECVTCERREAGAYSKKEVSSDAAAAGSDKGGASDEGQASSEKKGTTSSAKEKYSLRETDKRLRRKRMAAELESGSDGETKPPVTKRSTVAAAGKGETQNGRRKTTKARPRRTMRPHRKRGRERSHRATSPQTASSQISPSKVQKTNYHTRMGTKSHSKSSALFSPPPVGSDGTKRYPTRRRQDGSTSSCSLNHKLDISIEYKTRHSSPLCNSSPYVSGSSVPSEYFKDNPREKDEVIASNNVVVALTINGSEQVLEGRDLSMDCSHLSRDKVAAITIEHGSESTAAFEINIRSRTSAVRMETPPPPYESVLSEMTC*

>Aqu1_225931

MLNDGDSSISSVEDTSMVDDGISPLEVASMVGSCSVEVCSEIVAIPNPSVSKNDVISRVEDELGNCVRVTNEEGTTEEDVMSKTDEETSVEEAKKLIKTETKFMSLSEEDKETSEDVSTTSLVVMTLEGDGDTIKSSLEDKRSSDETIADVKLKDSMSETDRMSEATPVSIDDVIKTIEEARLREDSIATSSEGDGDGIIIDPERDKEASLEDIAACSSEEDCVSTTFDVVKANGEELISSMFITDCVDEGNIDSAPDVLITRDSLDETRFKNEDERTDEMIIEESSASVSVAESTDSMVNKTSLLDLVASNE*

>Aqu1_226869

MKPPSVILKEAFDSCVTYPPSDFTFSELSKKVMLPVQEVKFWFNHLKTIQNNRISGAQKAAKTRRKKKTQPSIQLGSTLCDPQPSTLNSQPSTLNPQPSTLNSQLSKTPSYSTNEYQCGICHTLYQEFTQVEEDWIGCDTCDTWYHFICPGIDREAIPKTYICTECSG*

>Aqu1_228735

MEKCKRISKRKKLLLEKVKKRKQIVKGSKRYRSTAENVEFPEEPPAVTKFRHWCTTTASIAISDKVYIGVHGSSHGIGVFAKDRIRKGEVLAVIPRSSTLSAGNSTLSDLISANIITDNSWIPLIITIMNEYSMKDESYWCTYLNTVPNNTSVCSPPLLWSSEERERLLGAETGLYHLVDSDMKRLRNDYCTIIKPFMETHLPRVKISEEKVIEIASFIMSYSFTDPYSNDVLSETMMIPFVDLLNHNHCHHAELKFGNKVLKLIATRSINPGEEIMNTFGTSLPNYSLLHTHGFIDLNNPVDKVCIPLNSLKRAFFMKGANWKRKIWSELIDKDEFDEIYEVDSNGIPDPSLLKIIKLLCSRGTSLELRKRMQSKLLQKYIKRCLANIPVVFAKEPSLKEEYVMKLHQQLNEYLNKLLLTSFIM*

>Aqu1_228868

MKGRLSHAEEDYGDYTTRCICSFTHDDGYMICCDECLVWQHVECMECIFETVTYRWSVDESFVTVDIAVDPNNLPDKYLCEQCEPRPTDKKRAKMIQMKKREEMTGTDFDDDDSASASTDEGQRSAASSPTPSSSSSSSKRKKKQPKTAGMLRVQELKAEARRGKKEKGLEKEEGKRGSKRLKSKHHRKNTGGVSVGVASTQSPRSPPPSMLSGVQTLLTAASALEQLSPVATSPSPSLWSSKTAPWSDPYTKITSNTYSDSLKNFLSACLQQLKQEAVVYTKEEFQRMLPMLPICHTREVCPSRVGVVVDTNVPEDEMIIEYKGSITTRRALLHDIGINSIKLLWKNKPCPFVYQYDRMSDIHICIDARHSGNMARFIRRSCCPNAELRHFFVNNEVHFGVYALTHLSPEDEVTLPFDFYDKCDYNLSCACDHKTECGLLNSPRGGRGIGGGKGSHKVNNNNKEEDMEIEGAVVTRKSLKGRGHSAVQLTDEDSNSSWAGKRGRQHSQQTSREVTDSDSEDNLETDTPKRRKSREERKTEAVWKMFEKMEATRRKKQQHLPSETSSHDNEEIPTPFIGTPRGRERLSSSGRKVNLLPSSNNKPRLSSISSVDLQSPEPMDAFHSPPLTPSLSTTSLQDLTPSSNPVSSPPWGSLSKLSFEVTPYLHPKYLLAGFALPRKKAILHQWEMDHLDNTITNGFSLSSLKSLEDTNRPRLRGCLKHRWMKQYQEMEDSYQTTPSSFI*

>Aque_prdm7_9

MNICVICEDCQKIHHGDCPVHGPLATLDPSSGYNTASLQYTSVPVPRELTVKESKIPKAGLGVFATELIPNGVKFGPYKGQKVYYEDIDEDDDTSYMWEIKKPNESYYIDGQVESESNWMRYINCARNEEEQNLVAFQYHGEIYYRTFKDICPGTELFVWYGDQYAKDLGIETTVNNYGLFICKRCSNRFKSKSNFKTHLQSNKDCFKANPQIFTCGRCQESFYALHKLQDHIRKHEEVKTETKIRFVKIEENVTNNEERYRDGAKRRRKADCQRRFHCEYCNKSFTQRGSLNTHIRIHTGETYHCQYCDKSFTIRDNLDRHIRTHTGEKPYHCEYCDASFTTSGDLNRHIRIHTGEKPYHCEYCDASFTESGTLNTHIRTHTGEKPYHCKYCDASFTTSGHLNTHIRIHTGEKPYHCKYCDKSFTQSSILTRHIRAVHN

>Aque_prdm12

MSAVRMEDPASSSNTTRTAVIGTFSSQVIAFALFGRVSRLSCAAEESAPSFSDSINQVSKQFPDIPDIFRFGPSTVKFRSVGVFCRQFLNQGAVLGPFTGRKLSVEEVITLKLKSYWEIHQDGKVQYCVDGSDPHYRNWMYFIQYARHQDEQNLMAIQHEERVYFRAVRDIQLGEELLVWYDKYQYDLYMGIPQGFKDSLSAEENSQDDEIEDDSENEEIEVEETDDEDDGLEQEAGRQQHSPFQHTNCQQNTSSPIPIHSTLPGISAVTPFTMYHHTPHPTQPPSIPGIPNVQWLKPHGIGNAPPVLSMPPNWPNFLPLSPTNIGKPSNLDMFERWADGTRWRCQICQRIFTSQGSLRAHARIHTGEKPYQCQFCKRVFTQASTLRSHERLHTGEKPYKCDHCGKAFTQSAGLRSHLKTHSMH

>Aque_prdmX1

MISGSSSSSSCSAESSTDDEYYERIVTKRKIRKMKMKSSTSHIQQQTNVQSKKPRNTEGPQSVIVQQYTSPIAIPPEVTVTESGVYANSVIGSGVRYGPYKGLKIAEDELMTETDDISCLWEIKCCESTYYVDAHEESNSNWLKYVKLARNDNEQNLIAFQHRRNIYYLTIKPIGPGAELLVWYGDQYMTEIDGANNPYKCRVCCKTFTSSAGLRYHLNYKKKNNCTSVFAPPYFTCQSCSKGFPTLFQYHTHIRNNCKKTGELKKSGELQKCELCGKLFLNLKRHQVLMHHSDKPPFQCSILIGYLSEVMEYQSNLSSMSRHDTSTGNGLP

>Aque_prdmX2

MFYCYSGSSSDPSDDNNDDSDYTTGYYKPKKKRKTTKKGGRKPKTAMRENDSSTSESPPQLQEQQARAPQSATVPQLVTAPQLVTAPQLVTVPQLVMPTIQLCDPAQLPLLQSSIQSQAVLAIHRAPSMNQNSVVQGPCGLPPEMTIINSCGKQGVFAIARIESGVRYGPYKGLKIAEEDLMEETNNFNAMWEVNCGESTYYVDAHEADNSSWLKCVRFARNKEEQNLVAFQHRRNIYYMTIKPIHPSSELLVWYGDQYLQEINTGGESKTLYVLSFMFSPLIDSAESFVCKGCSTSFISFDVFELHRKTCSSTPYFSCRRCERKFPSLFQYHVHIRDHRESPCRKPEKFQCNICNKLYTSRVGLRNHQQRMHSEEKPEKCPHCDKRFAQKGELRQHMQIHNERHLKCCFCDRMFAYQTLLDCHLRYNHTEIKNYQCYICGNRYKTANHCKLHITRSHGSIQAAEQ

***Branchiostoma floridae* (Metazoa)**

>jgi|Brafl1|100071|fgenesh2_pg.scaffold_353000003

MLFSGFEDGVFEMSSMQPPDSQNLRYCLTRIRKNDDTRRLSYTSLYSRSLTNVRLPNEPEHFLYLLERCLQLQQGVFYEDNVSFVSVLERALDVCIEMGEWQNALEYAERLGRILRVYLQTDIGLGLLYKKKGLIQLELGRTAEAKESLSTAKRLLTVTHGWRHDLVQHIRNVLTDLQADEESTLNHENNFSEL*

>jgi|Brafl1|101392|fgenesh2_pg.scaffold_381000021

MNTPPIANDLPSKSIADLPLDGKSGANFADTFFDPNLNPLAANSVQDMAGVQTVLPPVDMLTEEDAAKSTLSPLKDEQSSVENELATSPSQPEGGAAVSMEDKSSPESDRPSGSPEIKLKIKRTFLRGREKLTSSLLSDGSSPEEAAGPSKKRKKKLMETTDNDDQTQEVTSKQSPPGKKGKRGRKKKATDPSTPVIDYPSSAIALTTPDVGTPVLPAASHPKGFLNLQALEERMPPKWLVGDLVWGKVSGHPWWPCMVAYDPVEGVYTKFKGGPIRASRLYHLQFFGDVAERGWVGERSTVKFEGKDQYEQVVEDSIKHITSTTQRNQARKKSAVKPSRKAAWEIALQAAEEAIPLSLLERKQQYTFKYDAAPSPTDEEDTAPQVSTPPGGKKRGRKRKSAVAASVCATSLLSDDTDNEEEHLVKKKRKVVKKAPTQGEDSPKKKNKAQKKEVERARFELFCQKQRDQVKVDHPDYAPEQIQEELQMQWNILPDKLKAKYTSKFTATSQNSASDSDFDQPGVKHGPTKQDKKVQPSSPKKRKTAGKSSEADLKSSVDQCINSILALSQSDSAESSYPSTPGQVDSPAAPYESLPVKKRIQRRAEEGGKPDKKAKKNRKPRRDDAGPNAVASEKEEGTQNTDVEELSSQVSDHESEESSAPSSSSGLGRGAWAASKENLCQVCEQVGELLLCEGSCCGAFHLDCIGLQQMPTGTFKCDECISGVHTCFVCRKSEATTKRCSIPICGKYYHEDCLRKFPNAVFEAKGFRCPLHVCGTCVAVAGGDVKKVKSRGRILARCVRCPTAYHVNDSCIAAGCIQLAQNNIVCSNHFQPVKNQAHHSHVNVSWCFMCSKGGGSLCSDPHQLVGGTAISDSDQLSHRGSYLLQQHLPAAGSGVAWGSLAVTRGGDLLCCEMCPAAFHPQCLGLEDLPEGTWFCRDCSLGKKPLYKEIVWVKLGTYRWWPAEIEHPSKIPQNIYNMPHQVGEFPVRFFGSNDYFWTHQARVFAFQEGDKGSKESATSKGIAKVFKKGVVEATERFKFLQTQKEQKEAQESQRIGKKPPPFRMIKTNKPVGSVQIHTADPSEIQRCECKVTDESPCGPESDCLNRNLMIECHPAGCPAGEKCQNQRFVKRQYPAVESFKTPDGRGWGLKTLVDVKKHDFVYEYVGELIDEEEVQRRIKKAHEDNVTNFYMLTLDKNRIIDAGPKANMSRFMNHSCQPNCETQKWMVNGDIRVGLFAMDDIPTGSELTFNYNLDCLGNEKTPCNCGAPICSGYIGVRPKTAAAAAAEERSKNAKKKQRKRKVAPKNVKKEHEDECFRCSEGGELVMCDRKTCPKAYHLTCLNLTKPPHGKWECPWHHCDVCGKLATVLCDICPNSFCKEHSTDDNVTKHPSAKMVCHEHTEEEVQDSIQALEEKQKKALAESEDENTAANADGATQKPNGQPKKKNQDKAPSEEVTVKKQKRKPKQKVENGTSGKGGKKQAGKAKGAVKKTARVNKKGKANVDSEQTVKLEAEEVPKVPDDDLDDFEGELMIDCDL*

>jgi|Brafl1|111620|fgenesh2_pg.scaffold_922000007

MCAIASKTITRGSIVAEYRGERISDSEAQRRLSSIQDGEPAKLLWVYTKSAAPMCVIDGDSSDYNPAALINHSRNNTNLKLTTVTGAEPDPAVLLIAKQDISQGMELLFDYGTRHGADFLRQSQTLQPCPASDNPSPEHIGSCHNSHLNEDSPLSSCSEHTTTSSPSMEGVERQKKLRVKEQITRCQCRLRNRSNVRINTVR*

>jgi|Brafl1|112391|fgenesh2_pg.scaffold_1220000001

MAYFQALFDRICGDLQRSTQEMSSDFLQLSTNQQRVSYALSIPSVHSYIKVKPMFKGKSAMEATKLREFGNKMFGQKDYDSALQMYSESVLKAPFDPNVNHPVAHGNMNGSSHGRAQCDKNDVTTNDGNEFSLALANRSAVLFSLGKYDLSMKDIDLALHHGYPKELTYKLHERKGRCLWLGRDEEALQSFLTAKEHVTKSSLNSKKRKSWKATVDKQIAALQRTPSSVESADPSSTTNIPSVSYGTNSTFPSLSTAVEIR*

>jgi|Brafl1|115033|estExt_fgenesh2_pm.C_1610006

MCSFLFNLNNDFVVDATRKGNKIRFANHSVNPNCYAKVMMVNGDHRIGIFAKRPIQPGEELFFDYRYSQNDAMKYVGIERELEVA*

>jgi|Brafl1|115742|estExt_fgenesh2_pm.C_3070002

MESDEEEEYTLQGPVDDEGFPHGHCTLAYTSGDKFEGHFDHGAKSGKGCFHFYDGSKLEGGFDGGLLQGKGLYTYEDGSVLKGVYIDGELNGPAEEFNSEGQLTFRGQYSDGVRCGFCWIYSADGGCICGDVNDEGEMSGKNIAYIYPDMKLALIGSFVDGEMVKGKLAEVVGISQDRPQFKMVSDGGIFKCDVSTGDGISSDPQLPDPYEQQMVYVGASTIQGASEGLFMKVPVKEDKVVAFYNGLRLTHAEVDARDWAKNGNTISLDEEIVIDVPPPFDDTKHYRASLGHKVNHSFCPNSKYDLFQHPRFGFIKCIRTIKPVSADEELTVEYGYDHHGCGADNPDAPEWYKEQMKSLKGKN*

>jgi|Brafl1|117164|estExt_fgenesh2_pg.C_20201

MLCGPPRPSLASPLPSHLPLALPSTPDTLTGSLPIQVSPAVTLMHDVTTPLSSAPRMSFMECSPNTCPYADQCANQRIQRHEWDPGLERIVTKDRGYGVRSKTPIPQGNFILEYVGEVVSEQEFRRRTVEIYHDHNHHYCLNLHSGAVIDGYKYGCEGRFVNHSCEPNCEMQKWSVNGVYRIGLFALRDIPAGEELTYDYNFHAFNMEKQQICKCGSAKCRGFIGGKNQRFNGALNNKPVAKNSGAKTRKSKTRLKKREQHSQQQSQGAQQQHPTPQHHLPRFGEPPQVIYKSCPHTIKPLSARERNLVQKHKMFLLRNWERVRNTNEAMKKKSSQETKSASTAVGGAYIPKTGREKPFQGNSSSPHTVQETCQETTDSVDYSPSPNGVKEIVRPEEVLFLRECKGSSSFAPENPTNSEYYSMIPDPLDLTTIENKIMTGKYKTMDDFEANMQQVFRNAEKFHGKKSPIGKDVCRLRKVYTTARSDASRQLEEILGEVSGEPDTTEMVEETVRDPEKDESDDDIIRCICGIFKDEGLMIQCEKCMVWQHCDCMRTTDDVEHYLCEECDPRQVDREVPMVPQPPYANRGCTYYLTLLRDDLLVRQGDCVYLMRDQSQRRSVDGKSVRTSYRLLSNISPDKLDVFRVEKLWKNEKNLAPHLITNKDSPRPEYWYPGCTKRQVHGSGWQSMVLTGVSRTGVIDGTRQSPPYCPRERIRADLPSASLLDSKAQKGNPR*

>jgi|Brafl1|117670|estExt_fgenesh2_pg.C_60150

MARIQGELFCEIGQGRGIRCNKKGSSGIEPGTLIVKEEPYSYTLTDGELLRTRCHYCLKRLENSVSCDACRTAKYCNEECKKAAKFHHTPECRGYSRLMNLPEHLRVMGRILYKMHARKTDMGALGPLSSLVSNVETLKNCEEGITSLDSKMECLSQHMEKDALPDRAFMEEIYGKIASNSFAILDENMCSIGIGVYPQASMINHSCKSNCIGMFYGPQIQIRANEFIRPGEQIFHGYIPPLLPTAKRQEKLLKTYHFLCQCADCRNTERSCLAQLDFTLHSDNVYVIRILKDLVEVCVELGLWEKAVVYGKRIEPGYKMFLKRYDLDQGLLYQKMALAYYRLGDQSAALPYLRQAKTTLTITSGEDSGLVQEVSDMLAECVAKEIDSLLVECGCPNMDH*

>jgi|Brafl1|118329|estExt_fgenesh2_pg.C_120093

MAGKRGRTWRRRRQRNKEHRPVSLCHEEHYIRFFRFLKKNGLDGFFLRPALFPDTGRGLMVPRKIKRGQTMIKMPQHMILSTKTVLDSVLGPYIESAEPQLTTIQAITTFLIYQKHIGETSFWKPYLDILPNEYTHPVYFGEEDFLYLPHSLRANIKAKKQECIKSYEELKPFFPSLEPLLPNWEGIFTFDAYRWAWSTVKTRSLYVDDKGSTVLRNLDKSGLGVTSLVPMVDLLNHSHSARTGLLIKKSCKNGDYFYTVTAEDDYKRGDQVLFCYRRADNQTLLLNYGFVLPDNHLDTIKFFLVKDIIGILELMNFEEEDPKFRRRKVLLIATKYTASDLTCDYRGVSKTLMTVMRILVCDSEDRPLFKRVLADDLPWDHPVSVQARSLAQELLERRLLSYGIMDDEAILAESDTPVRRLVVAMRNEEKKILQKGLKSLTRNNEMHK*

>jgi|Brafl1|120015|estExt_fgenesh2_pg.C_370216

MSSRTKGRYLVANGDLPMGHLVISEEPILTGPSYTTSALCLGCFDSVDGSYRCKGCNWPLCNRKCETAGSHQLECAMLKTADIDITDFVLTRLLYNIIMPCAVSCSSHVTRPGGNSSAGWSLTPRTKHENTGRCLSLACYDQWKKSQSYLIATIKLQRELYYFSRMEMGRNVVRVIRQTLGMDEFSEHEIMKVCGILTVNAFELFTTGPNKDRDSTPRAWAVYPTTYLMNHDCLANTLTSIDSKNKMHVRTRMPVKKGEALTAEYSECLWGTEIRRHQLHRYKYFWCSCQRCRDPTELGSFISSHRCTNCGGNVVATKPLDFNSTGFRCESCRVIIRGEVLKGTNHQVEEEVFELESKELETPIEEYEDLVERHESILHPNHYAMLRCKKFLAHAYGRQPGYSLAELSEDRLNKKMEMCRTLIDTLEIVSPRFNDDTAHFLYELHAALVEVARRRHDLGLISNQEMVVALTEAKHILHQALKHLYPWNDG

DLRDLEAESKAKKSLEALDGWIELVENSKPTKPVENTKPSKPVENSKPTKQVENTQPSKPTEQDKPKQEKQQHTAKKSKGKRKRKGGKGK*

>jgi|Brafl1|120285|estExt_fgenesh2_pg.C_420108

MEDGEPQKIGKPSPPQQDATKVEGNSSSESSSSSDDDDEDESTESAESESLSSSVDSSRGPRIKHVCRRAAVALGKPLAVFPTDDKIRLSALPPKEKRRLLHKQKAEERPGTPDSDEVPNRARTEEKAKRPLGLGSPPKSRRRRRCGTCDGCKRTEDCGKCPNCKDKTKFGGFNVKKQCCIHKKCRNPQPAGDESIRPRMGVQRPRPIRAGDGKQPLKVDFKEEYDVENAWEGGMVILASTPLITRIVCYLCASAGQHEICPKCVRCKSCGRTTPGQAYNAQWTHEFSLCQDCGKLFDMGNYCPICKKCYSDDDYESKMMQCGKCESWVHAKCEMLTDELYEVLSCIPDTVHYTCPSCTQTEPPEWRQAVQKELYTGFQQVLGTLISHKSGEHLLHTTQEEAAESDVTPVVTQEVQTASPEAMEVTAEPQDKDIKTDEELLAEGGDKKVETPEKKEDDPGDLVAVRKQVEAMEYTSVSVFCTNLVRIIQGALSAAPDNSIMKTNTNVIRVLFIKAAESDVTPVVTQEVQTASPEAMEVTAEPQDKDIKTDEELLAEGGDKKVETPEKKEDDPGDLVAVRKQVEAMEYSSVTAVPELGAAPGPPPGVPVKTHGKTAGKTAHQGTQAVEH*

>jgi|Brafl1|120570|estExt_fgenesh2_pg.C_470036

MESDEEEEYTLQGPVDDEGFPHGHCTLAYTSGDKFEGHFDHGAKSGKGCFHFYDGSKLEGGFDGGLLQGKGLYTYEDGSVLKGVYIDGELNGPAEEFNSEGQLTFRGQYSNGVRCGFCWIYSQDGGCICGDVNDEGEMSGKNIAYIYPDMKLALIGSFVDGEMVKGKLAEVVGISQDRPQFKMVSDGGIFKCDVSTSDGISSDPQLPDPYEQQMVYVGASTIQGASEGLFMKVPVKEDKVVAFYNGLRLTHAEVDARDWAKNGNTISLDEEIVIDVPPPFDDTKHYRASLGHKVNHSFCPNSKYDLFQHPRFGFIKCIRTIKPVSADEELTVEYGYDHHGCGADNPDAPDWYKEQMKSLKGKN*

>jgi|Brafl1|122719|estExt_fgenesh2_pg.C_970098

MVARRGRTWRRRRRRKDETRPVSLAHEESFVRFFQWLHRNGCRNVPLKPAVFPETGRGLMATKALKHEELILVIPKRLLITIDAIMDSYLAPYIERADSQLTPSQALAVFLMCEKCRREKSFWRPYIDILPEEYTCPAFFTEEDFRLLPNSLRGKAKAKKYECHKEFMELAPFFKMLADLFPDQEDAFNFKDFKWAWSAIKTRAFDVPLGGETCYRLRDSEDTSNPTMFPLVDSINHAAQAKIRHRYNEKRRCLESRTETVYRRHAEVMNSYGRADNDNLLLEFGFVVPGNPADTVTFHLVQDVLEYLQPENNELLERKIMFLARNNLISDLTCGVTGVSPQLLIVLMVFLCKQDDEDVWEDLRTGRFEEDGPLDVRARKVAGRLLSLRLRTYATTIEEDEGRLKEVTSPCGRLAVYCGMERKRLLQNGLKMLQHSSKVVIAKGVLRKLFSGR*

>jgi|Brafl1|123761|estExt_fgenesh2_pg.C_1280074

MVARRGRTWRRRRRRKDETRPVSLAHEESFVRFFRWLHRNGCRNLPLKPAVFPETGRGLMATKALKHEELILAIPKRLLITIDAIMDSYFAPYIERADPQLTPTQALAVFLMCEKYRREESFWSPYIDILPEEYTPAFFTEDDFRLLPNSLRGRAKAKKYECHKEYKELAPFFKMLADLFPDQEDAFNFKDFKWAWSAIKTRAFDVPLGGETCCHLRDSGDTPTPTMFPLVDSINHAAQAKIRHRYNEKRRCLESRTETVYRRHAEVMNSYGRADNDNLLLEFGFIVPGNPADTVTFHLGKVSSGNYVICCPRQIWRGTVGRLIVRMT*

>jgi|Brafl1|124382|estExt_fgenesh2_pg.C_1460060

MYSTFAAGKYLRQKRIDFQMPYDIWWQYKHNKLIPKIDERTKYKKIRSNIFVDVKPLSGCEPVVCTCVRPAQADKACQEDCLNRMSFMECSPNTCPYADQCANQRIQRHEWDPGLERIVTKDRGYGVRSKTPIPQGNFILEYVGEVVSEQEFRRRTVEIYHDHNHHYCLNLHSGAVIDGYKYGCEGRFVNHSCEPNCEMQKWSVNGVYRIGLFALRDIPAGEELTYDYNFHAFNMEKQQICKCGSAKCRGFIGGKNQRFNGALNNKPVAKNSGAKTRKSKTRLKKREQHSQQQSQGAQQQHPTPQHHLPRFGEPPQVIYKSCPHTIKPLSARERNLVQKHKMFLLRNWERVRNTNEAMKKKSSQETKSASTAVGGAYIPKTDVFMTQFTALKTSRSVRTRRLAAAEENSEVTRTARLAQVLKDIYNTVVNHKDTKGQALAAPLMILPNRKRNSEYYSMIPDPLDLTTIENKIMTGKYKTMDDFEANMQQVFRNAEKFHGKKSPIGKDVCRLRKVYTTARSDASRQLEEILGEVSGEPDTTEMVEETVRDPEKDESDDDIIRCICGIFKDEGLMIQCEKCMVWQHCDCMRTTDDVEHYLCEECDPRQVDREVPMVPQPPYANRGCTYYLTLLRDDLLVRQGDCVYLMRDQSQRRSVDGKSVRTSYRLLSNISPDKLDVFRVEKLWKNEKKFFPNELFRVPLYEIIPLEAVVGLCCVMDLKTFCKGRPKTVKEQDVYVCDYRLDRTAHLFYKTTKTKYPICTKPYAFDHFEKRLVPKRTYTPHHVPEHYKKTGGRPYWKSNRQDGSSETSSTMGDSIDQASTSGLEEPVISDNISPSSSAEEAPISSEPHKTKQRPKQKERLNNVLLKLLAKLPGKHKPVDVSYLLEEGAGKRQRKKTTLVDCW*

>jgi|Brafl1|124463|estExt_fgenesh2_pg.C_1470094

MADGVEIFSSAEKGRGLCATKVFKPGNLVRAADPYAYVLCNSERGKRCDFCFARKDDMSRCSGCKFARYCDGKCQKAAWTEHKSECKSIKTVKPETPTDSIRLIARIINKTKTDSPGVPGNSIDELQSNLREMPENVKEMFAQLAVVLRMYVGKDVMDDAREIFELFGRMTCNTFSICDPEMQYIGIGIYPKMSLFNHSCEPNCVAVFNGLRMEVRAIQNIQPGEELLISYVEMLAMSSVRKQQLLQQYYFTCKCPRCQDQTKDGMMMAVKCGNINCKKVIIQVDGAYETCKVCCHDNEKDPKFWKEVNKVTQFSEEMLAAITSADQRQEPKEGLRLAERLLERQQQVLHNNHLFVLKCLDKALDQAVVLRKWNRALRCALQTIEPYKVHFPAYHPSLGIQYMRIGKLLLYEKRLAALEALQMAEHILNVTHGKDHPINKELGDLLTQCMDEMRMYQAQKKK*

>jgi|Brafl1|125045|estExt_fgenesh2_pg.C_1670003

MKEDICAQSKTECSFKSTDLLHGPGYMEQRRDDLFHTSAISWCCGYRYSTQTDFQAGFLTNYGGTINMCVRHRQGDVNTDLKSCCTSTHLLVTMATDGDAEGIEMSDENFYENLDDIISGLVNEKLESSGWVEGLPPGMAAIERDLGSLEDQQARIQDVFRESERLLGVAGENLAWQQTWFNDWMEHNRTGSPEEMVDLTAEDDEVMVVSEGPATQQQQLQQPQQQAQQRQEQEHQSELPSTPQAATEEETVPTAPQPPQTESSVPTTNQESGTSSEQPIADQNEEQVQREVAPGTSDPVSVELAVGMRLMGKKKDDIWYQGTLIKIVPDEKGLIKYKVKYDGKGKGLLSGNHIAFSETVGQGVLHAGSRIVGQYVDEEGDSVQYLYAGIVAELPSKMNRSRYLIFFDDGFATYLKRDQIHQRAMVKVKVGQKIRTEWNGQWWDARVDSVDGSLVKMYYPTDKRSEWIYRGSTRLWPYEAFAHQEQAQTEGSKSIRSHTMGKTRGPHIEYTRAEEEAAKRKTTGAAQKKPASASASSASQSKPPSRSSTPRSSSTVDSSSLSFEKLLTEDIEKLQAGSKLVPSTSSELQNSGGGMGRPVANREAWFQIAGATQKKTVTSQQSNTTVKQTVTSQQSNTVVKHVVPTTVKQTVTTTQPPVTTTAQRINRPLKKQVARKSTGPRSKKPFPDQYVPPSVGRKDIASILQKRLMEEGDSNSRSSEDSVVDITVETDPMEVPTPKMVKFSPHQCTPACVSHVRPANPAQYRGRGAWPHINPLKVPILYQWERYIGKKRPGGTRDVSYRTPCARMVRNVRDVSWYLMQTKTDFLSIDQFCFDPYVMLDNMRPEKYFVKIPDISNGNEDVPISCVNEINHEHPDNVGYTKQRLPTPGVELNLDPDFLVSCDCTDNCQNKKTCACHQLTIQAYRSRPGGQEDPDAGYEYRRLTEQLPTGGWGIRCLDDIPQGAFICIYAGQLLNEDTANKGGNMFGDEYLAELDHIEIAEKFKEGYESDVPDSSGSDLNDSDSESDNSSSAEEPSITSSTSPEPDLDNSSDSDYKPDITDKPGDTSGAVKLILRRESRGSTSGRSVKPQKCDTDKKVESWIEKNLSEKKSGDTGSQSESRSSSGRNTPKQTHQSQDSKPGLPQPNKEDQSQSVDTNPAKKESSVHEQERHNIFDIMEFSESKDGTSKTPDREVQKKGDTNRSFQDALKDMNVSMELSPKKAPGQQGTVGAKKDSGKDDGMACTIVEEKEAAEGKAERREGDVQDKTGKTEGEEKDKKGKDGSSPMRHYGYRIDGDRIVGLKRKHRKKIVVKASSSSESEESKPKPASGDCELMIVGATSLAVDSGDETSDSEKGSSSDKGKKKKEKDQLEVPAPVLDDTQQPVAVLQPSPDRSTRKYFGEEHCYVMDAKVIGNCGRYLNHSCSPNLFVQNVFVDTHDLRFPWVAFFSSKRIRGGTELTWDYNYQVGSVAGKVLYCYCGSEECRGRLL*

>jgi|Brafl1|125138|estExt_fgenesh2_pg.C_1690059

MPPRVVLDDNNPFSEAFQEREKLHFPSTNNYTNIKETSTILSLNGRMFANAIQAQRMPPRVVLDDNNPFSEAFQEREKRERMERLREQQERQRMQLQQEIEQQRRLQQMERQDHDWHNQEVVQNNMANERRQGMMELPFFNTPEIPDIAGQLQQNPNPQQQQQQPPGQQQGPPFMNPNPQQQQQQPGQQQGPPFMGPGQGQPPMGQGPRFPPGGPNFPQGGPGSFPRGPLMHPRMPFPGPMGPIDPHQGFPVPPNYPMSGTPPETEKPKPKRKRNRKKKKSADSEEPPPPSMQQQQGMPPDIPQNPMNPQAPTMPHPGMVQGNPMMPPVRRDPSQPPLLDQSFTVPLRHIADGSQGQLDMNKHSLLLQHLTTEQKDALRMSATTGDNAPRIDSIDPDKLKAETLKLDPQKLETGNGEGEKGKEEDMEEGKGDGNGDGKKDSPEKPAANQLLKALLQGTPTQNLLAKAGLPPCKALQEAQDVTVEASTPEKVKKEEQEDSDDEEVINKLKLTPEQQKQLEMLEQMPETVPKGQNKWPTLNKKKEWDPKSLSSLPPAHSAPDVIVREQEEFERRRQEYQQQLKKRKETQTKRASKKRKKDEEDEITKMKQNRFPSTEMIMSTVKQLSLCEPEISVNFALFPPYGSGPLNGDNQLKGTFGHAFLDGVQDYYSNFLKSETLSNPPTPPASLPPTPPPPGMARPPMVNGHLHPDEILGTRRAKLIGDQRSHDQDELMKEMVSHQRTITRAENSTVLGLDGGKTMQDVNVPPSLPTPPPSNLAPGEAPRFSRDATTPDSIVPSSSPESVIDDEPPPQPPKLISLNRGPNRGPSPTIPLIAPTPRRDYSDDISKKPGLPSSCQLTFPKTLSKPKLLSTHPPSGGLPPGPTNPTLVIQSLHHHRAPKLTSQTTQATALPQCGMPRHKSADSAEPQRSKVQGIHPPSAIQTAQLAFQLSEPGKEAASASTSSTSFTVVVVNKTFLLQPNNAKLRGLPLVDSLPLPFAAKPPTEFGPPRDHPIPPTSGQSSFPSSQAGVFVRPSFSVPQSVIPPNTSTVDIRTSVSMPRATDAVQVTRTSESAFSVSNLERMRTLVGQAGGALPPQRPDGNMSVSLTLSATAAEDINGIVAAVADLVRVPVPTSYEISEGVDYPSSMAEHFRMQQQQRKGDGGVLPPQGVSLETLLQQGKPKFCRHCDVVVQGDGIRKASSDFPFLRDQEGRSDQVRTRFVAQDSSDSDSELTFCSSTCLMQFAISLQSRGRRETKEKAGSIVDHRSRDIVRPQHSEIPIHLSPTYVNNTSKPFSEGQGSEGKSERPRLKRRSDSNTSQTSQDLPPEPPKVLIKKWKGVRWRRWEVSILVPKSTYRPPSEKEIDELMSKLGTCLKPDDLPVDSRCCVLCGRAGDGDTEAAARLLNMDLDMWVHLNCALWSTEVYETLNGALINVEMAYKRGQTLACTACNKFGATITCHRYNCKRIYHLICAIKENCMFFKDKTVMCPVHAPNKHENELVSLSVFRRVYINRDDSKQIAKIMRNYGEKKYTLRVGSLIFHNVGQLLPHQLQAFHTRTAIYPIGFEVTRLYWSMRYANKRCRYVCRVEENQGRPQLVIRVIEQGHEDVVFKGSTPKLVWLNILEPIEKMRRGSDVLKLFPNFITGEDLFGLTEPAVLRIVESLPGTEMLQDYFFRYGRHPLIELPLAINPTGCARSEPKMRTYIRRPHTLTSSNTSRSSQTTLTGELLSPYHKQFAQSKSAQYRKLKQEWRNNVVLGRSRIQGLGLFAAKDIDKHVMVIEYIGVIIRNEVCNKREHIYEEQNRGVYMFRIDSDLVIDATLAGGPARYINHSCNPNCVAEVVNFEKEQKIIIISSRRLSKGEELTYDYKFDIEDDEQKIPCCCGAPNCRKWMN*

>jgi|Brafl1|125552|estExt_fgenesh2_pg.C_1870057

MLRRAVRRKDETSGTKREGTEDAGEDTEGPKKVLRTFQDKKTWKAARKAKEESALEELVLGRRQEEPTDRLQDAEHQSEDPSEEGSDDDVDSLGVVDTREAAWVDDDDDDDDRTRVEKTSDVPSWAQTSQGRKRQHESDDDSDEELLQHTGDLLAESSALPRGNINIKRCSDVNKTYPHQGPVRRVEFHPSAQVDGRNNPKIQTLHLEKFPVRTAHFSTCGREVILASTFKWFFVYDMIAGKVIKIPKIRGVDDTSLSRFEVSPDGRFLLFVLVCVCLRVSVLKVNMFVLMFMTGVDDTSLSRFEVSPDGRFLLFLGKNGFLHLLSAKTKECVSSLKMNSDVSAGTFSKDSRHLYSVGDDGEVYVWDVGTRDCIHKFVDEGCTHATTIALSHNSKYLATGSQSGVVNIYDTQRALTSSYPTPLKALLNLTTPVTQTTFNHTGEVMAMCSNEVFKAARLVHLPTLSVFSNFPEADSTVKRVQCLDFSPHSGYLAMGNNQGRAMLYRWDQTLQGLLIQPVPRPSCHQGLNGLPDRRGGHGDRKGGHAGHHGHWRAYKLLVDPALQKGHQQKLDKVYRYDGVVQGKPDLYPPIHVRDPRRLSRLWRRNEPADLPVPKFKVDDFYVGSPPRRQITFSNLNDNINSGFLQEMCKKFGTIEEMKIYHHPKTRKHLGLAKVVFATTTAAKDASNKLHRTSVMGNIISVQIDSKGKIREQLYDSIVHNKPSQEAPQVSKADPRRLSLDERPHAFMPPQRNLPIIPEAHHRPEAPLPPAPLPPARHDVVPPVYPPVPPEVHPAGVPPGVPPGVPPGVPPGAAGVPPGVYQQPPRPDGHKGYYSDPRRRLSSEGGAFAPYDNVSAHNSFPRPPAGMDDQGYYSNTPSSYSGQGYHGNSYHSNTPSLSESTPSYVPTPSSFHSSGQTPQSYPGGVQPPNFAVHTPGSYYSGTPVGFHGNTPQYSGNTTAPVTVVATTASTTVSVATPTQPPEDPAQNGGEKPADKGSLSLDSRIELLLKQQRTFPALGGMSPSYSEEEKGERSPTPPPLESFTPPHDASPRPPPQNSTPSSLQLLKTDDLLEEISPGSLADSDEDKNKGKSAGKEEEAVTMVTDRNAEVPTPVMDEEKGEGDMDISSGNDSDDDDKMSLSPLSSPEQSKLVVNPPPTPVYPPNVPPPNFPPPNIPPPMLPNPSVPPPNIPPPNIPPPGMEPSAPSIPPPGLPPPPLPPVSSLPMPPSSVPPYSSNFTNPMLPNQQQQHLARMGLWRPGMHNYGNHGNQQPKGPFPFPPPLSANQGPPRQPQMRGPRPFGQGATFRPWRPMFDPTVPPPGYVPPVEKVDPHKQTIDGVMERIVWELKEIMRKDLNRKMVESSAFKAFETWWDREEEKTKVPIKSEEKDEDKVVEPEVKVKPKPSKSLFEQPAALPSISNMDSFSPFGPSGLGGLGLGIRAAMPRMPSFKMKRKPPDSPAAEDGHPRKRSRLASPSMDDGSASDSDADKEDMLSQDSDLSRHGTPILQRAHSLDSEGSEVRVVPRPEEEQKSIIEDDDDEDGDEEEEDEDYDEDEDLAEADASDEEFPEEDELMVSDDEDEADDESEGSESGEDSDSYLSSSDEEETLADVTETEREFSEAEDSEQVPDSDATLPVSHQRSAELGQDQVGHRVMVSPGQEQAQSIQPPPTLAARKQGLATPEHAYSKQATSPSILSPRLPSPRLPGVLSPPLIPGSDSRTLPVDGATVAHGEDIRSVLDPHLQLRHLHRVPAGPAGPEDRVGASPVRPALLLPERSAFGFMSEATGAMLPSSPFPMEDLPRTPGGNIDLPRTPGRGMTLQLPTDPAAVPGFLAPTQAGATAIPSSPWRGIPPSPSTSLPVPSLPPLLAQFADIVTHEAMQRDVVAKDDSSEPPAAPPDTQTTPPQLLASQGKFSIASLLSPQQPARTEPLPPARLHPGLPITSEEHLRMLQANCQLPRFQSPPRPVQPPVQHLLPNQDQLLSRANNQAANVLANELLSPSKQTVADLLAASRTLERDVDVEGSDSEKTETSSEPEVSVDEVQERTSRQRVCPYMLEHNYAAPLPEHNYAARQPPSPAKPSLTHKQSKTRTEELPVVAAPPVCLPVNIKTEPPPVEDLPKDKENEAPIEVSIPSVDLVTTEKEEPKQHRKTAEKEKEQILREQITNKIKEEAVKPEPLRFDPRDNLMEMEVLYEFLKTGIDLEDVKYLQSSYDSLLQQDNSGVDWLNDTHWVYHPHILSQYSNTVTLVHRRKDDPTLGDHKTGCARSEGFYKISFKEKTKYLHRARLASVVEKPEDDLMESQNKAKQATQSSREARSNQRRLLASFGASCGDISDLLRFNQLKFRKKQLSFRKSRIHDWGLFALEPIAAEEMVIEYVGQCIRQTIADERERRYEEQGIGSSYLFRVDHDMIIDATKNGNLARFINHCCNPNCYAKIITVEGYKKIVIYSRRDIAVNEEITYDYKFPIEDEKIPCLCGAENCRGTLN*

>jgi|Brafl1|125839|estExt_fgenesh2_pg.C_1970013

MATDGDAEGIEMSDENFYENLDDIISGLVNEKLESSGWVEGLPPGMAAIERDLGSLEDQQARIQDVFRESERLLGVAGENLAWQQTWFNDWMEHNRTGSPEEMVDLTAEDDEVMVVSEGPATQQQQLQQPQQQAQQRQEQEHQSELPSTPQAATEEETVPTAPQPPQTESSVPTTNQESGTSSEQPIADQNEEQVQRETVGQGVLHAGSRIVGQYVDEEGDSVQYLYAGIVAELPSKMNRSRYLIFFDDGFATYLKRDQIHQRAMVKVKVGQKIRTEWNGQWWDARVDSVDGSLVKMYYPTDKRSEWIYRGSTRLWPLYEAFAHQEQAQTEGSKSIRSHTMGKTRGPHIEYTRAEEEAAKRKTTGAAQKKPASASASSASQSKPPSRSSTPRSSSTVDSSSLSFEKLLTEDIEKLQAGSKLVPSTSSELQNSGGGMGRPVANREAWFQIAGATQKKTVTSQQSNTTVKQTVTSQQSNTVVKHVVPTTVKQTVTTTQPPVTTTAQRINRPLKKQVARKSTGPRSKKPFPDQYVPPSVGRKDIASILQKRLMEEGDSNSRSSEDSVVDITVETDPMEVPTPKMVKFSPHQCTPACVSHVRPANPAQYRGRGAWPHINPLKVPILYQWERYIGKKRPGGTRDVSYRTPCARMVRNVRDVSWYLMQTKTDFLSIDQFCFDPYVMLDNMRPEKYFVKIPDISNGNEDVPISCVNEINHEHPDNVGYTKQRLPTPGVELNLDPDFLVSCDCTDNCQNKKTCACHQLTIQAYRSRPGGQEDPDAGYEYRRLTEQLPTGGWGIRCLDDIPQGAFICIYAGQLLNEDTANKGGNMFGDEYLAELDHIEIAEKFKEGYESDVPDSSGSDLNDSDSESDNSSSAEEPSITSSTSPEPDLDNSSDSDYKPDITDKPGDTSGAVKLILRRESRGSTSGRSVKPQKCDTDKKVESWIEKNLSEKKSGDTGSQSESRSSSGRNTPKQTHQSQDSKPGLPQPNKEDQSQSVDTNPAKKESSVHEQERHNIFDIMEFSESKDGTSKTPDREVQKKGDTNRSFQDALKDMNVSMELSPKKAPGQQGTVGAKKDSGKDDGMACPVVEEKEAIEGKAERREGDVQDKTGKTEGEEKDKKGKDGSSPMRHYGYRIDGDRIVGLKRKHRKKIVVKASSSSESEESKPKPASGGCELMIVGATSLAVDSGDETSDSEKGSSSDKGKKKKEKDQLEVPAPVLDDTQQPVAVLQPSPDRSTRKYFGEEHCYVMDAKVIGNCGRYLNHSCSPNLFVQNVFVDTHDLRFPWVAFFSSKRIRGGTELTWDYNYQVGSVAGKVLYCYCGSEECRGRLL*

>jgi|Brafl1|127996|estExt_fgenesh2_pg.C_2860038

MSDHYYNPESPTGDMDISSESGSENEADKDDIPPPPPPPVAQLPRIKALVNYDVANTSESSDLSEEDDTKRDNQTEAKGQSSKGHGGKPKSTKFYPLVENGEKHSKELDSGITDKAVKGPEKNTKFEPLLIFKDQNTIQIPRPITERTDTASVFTTQSEDTILGKTEKEEDDSKDGAGKWKPVGWSALQGNKTSKTDVKIPVKTNSFLSVKKTPVDFIPMKKTTIDDAYGLDIFEQSQKRKGGKANKSGAISFQLRKTTLGQKPNIRVFMPRQLKVRSSSESSLPRALSSEDPSPPPEETLPPLPKEESKEVVKPPLPTMEQEASTKPPLPIVEPERKEKPLIPISIGDASLKVHSSTPERPSSGQSGTLIERRDSKEKISRWSDASGERMQPNTVVTERRDSKETSRWGFEAGVQPNVQAGLPHVPTKFEALPASDSPRTLPVEQALDSIPLPCVDSTTVAASHAMFKTDEVELHGGANHKNSDQSDMDIASDQSDVERAKSAESSSSDTEDDSDSDADTRKRLRSVVTVVVTKQQTSSPSSTKESKEKKHESPSSSREPREKKRDRERRSGSRKGSSSKDDSKSERHERSRTRSRSESCSSSKTSPHNDHSRNREKSKTKSDSSEIKREPETSRRRESTSDKHRSISESKPHGATTQEELSNANILPVKDKPVDSMDTKIGKSLSCAVDKIDVKDSNILLVSPKKEQNVTQESNIKPVPTEAVHGETLVSSSLRHEPVEIPLSRLSDEQTFATNRKEFEVESLSGKQTSQPAIETFATKHSEDRFSPNKKDDSYPEHKHTVLETPMLDSEESSTQHKDGKEKVVYHALVNYSSQEEDDSSRATSPIVEEGISEEKDHSKTVSSSSGGVLEESVGFSLTSNAEINTVFHKEGTRPDDITSQFSQTASECKPPMEEKGVWPLAEEKSCDTKRTEPIPTREGGEEMAIPVVSGTGRRQKKATRWDSDTREKVEVNEHYVMDAPESHFEDRSQEPRILQPLHGDENDGGSTPLHDEYEEFESEPIVMGHSTNEERGRFLSTVHGRSEERVSIMAATSTSSHPQQDDVSYPSCTIRPMQPRVDLSNPWDDYEESGLYADFSGPSMLPRAPSISDSAAPCDSSASKDLPYVSNFVESTQSTEQPSDTWKEDEVRNLDVQNSSNVQDSTQFSEQDRQDLELTPIRRSARLKSQDSTDLSSSPSLSKDGTEKPQRRVSIEVPQEASSETTEDVLSEASAEKAVPEKEKKPEENLRPPFFEAITENLYLSERKKSKMRKDIRRMLCECMTSEEERDAGIAACGEDCLNRLLMIECGPRCLCGEYCTNKRFQRKECSRVEPFNCGDKGWGLRAAEDMISNQFVMEYVGEVLNFSEFKQRTKEYNRQKQHHFYFMALKNDEIIDATKKGNVSRFINHSCDPNCETQKWTVNGVLRVGFFTRRPISDGEELTFDYKFQRYGKEAQKCYCGAANCRGYLGGNKTTPVRQRTKKKVEDNLLDEEIDQMAEECEEGLTETEQVLYLSRLMVRSETVQQRFTLLKILLATDNQSCLKAFLRYHGLSLIWSWMVDMTDGSAPPDLQVKVLRCLSHLPITNRTILDESKVMAVVERWAKQLANQQPADADTESSSGEPLSRSATPLTLSLAYRNSPSELKSESEKNSESDNERTPAKRRRVQLLEDMENVKTAEDKDKNDVTCSTQSEETEEKKQEEEEKIERMMETRKEVADTSSDAKKGAKDSGDGEEDSNDTREADPTAESGSSSAKPAGNGEDDEESSDLESEQSQEPERPGGIAEMAAQLLESWSSLKEIYRIPKKEKEKRDSDIESEPDPERERERRDRERDRERDRRRDFKEEERREEHPWRMSAKKRPLEEPDSHSTPIKARWKELRKKPLDRPRSPLIKLSKEERRRLFEKKVAEEEETKQREQQELYMHQLQALQALGTFDPNMAQAFQQYSYDQEGAPLYPGGPQHAPDVSMDTSHIVPGQPVENLPQYPPQDFLDDSIGADPMDQMSRSLLDSSLNVSHLSGLEDPSGTPTSSLPTTPQPIPVLQSNVPVHPVTSSQPPPSVVVQSVPSPQVSHMQQPVQPQQQQLPVTPQPLPAAPVQQQQQQQQQWEQGQPVSFVVNPQQQQQPPYDIPQPPIVPPHQIPPQQQQQQQNIVYQQVQYIQPGPGDSQFYPQPQGVMIQPNVTYSQSGLVHTQVMQPIQQGKRKPTKADADTSSETARKCKDVFRKKMATFVVSILNPYRKPDCKHGRITSTEDFKHLARKNLAHCVTVPALSQKRTPYGQDLIGYRKSAEGKTKRYLKQNYNKKRRPKGWHRYSADEKRVAWGPARPRIIQGELFMKHFRKALY*

>jgi|Brafl1|129904|estExt_fgenesh2_pg.C_3960032

MAATLAKVPACVLRSGFLTSLKNNYWSRWTGTRAQNMATLPSLCSMFRGKASTSQRLVDRLVRDPTVVQGNWKIDARQQSNYAFRGATHNALEEQKHDNEEETTQETLRPDEDSVSVVPRADISGKCVVANTNIKAGTHLFTLYGDKLRQPTPYTIQVASHSHIHPEGKLIFVNHSCSPNTQFQYTPSWDLYAVRDILPGEELTFDYTTTEWQMAQPFQCNCCNTKCIHKVQGFYFLNPEQKAEREAVISPVIREQ

>jgi|Brafl1|131096|estExt_fgenesh2_pg.C_4920022

MNTPLEGVAPTNGTRDSMAAGSDPESVEAPVAPGDSNSCQQTTNQTTTTASSTAHHSYLGLPYQDHNYGAPPPPSPPPSPQAITRINGVVDENSSNTNVSSTVTADEDTCADDDAGITRCICNFDHDDGYMICCDKCSVWQHIDCMGISRDRIPETYLCERCEPRDVDRQRAVEIQTRKRIEMTDDDTSGTESGDEMLTYRAVSHTPTSVTITTKPTKKVKKRKREKSGEKDHEGRKKKVKVHRTPNGFNSLNNEDANEPWDYSPDDSDVYQEAISNQYTPDLASFLKTRQYTEEDVCITSSDQLQVESGEVASIGSAMKCVRALRDLHDNQPILEYRGKVMTRQEFQDPFFKRPHPHVLFYPVNGLEICVDARSFGNAARYIRRSCSPNAEVRHVLLEGMIHLCVYSLEEIMKGTEITIGFDYDFDTCPCEVECACQRESCPVAKSNARKQELLKQKRKRNKSGQQSGGDSESSTGRQRKVSPLRVSLNHNASTNHMTSDGEDESKDVDVETEEAAEERRRKMTREERKLEAVMRTFERMEKRAARSRAAHARIEKQKQTKVTPNENKEGEESMSKDMEDLPKQTATPHRQIRKRRRSQSRRNHAARGRQRLNSTCSSDMPMSPITETAANTEVVTTEIELKLSAPTTPVSALSPSIPSPSMAQELTVSTVSSVNVVPQPSPAALGKGFKFPKTKKFFVNEWLNEKANEQSAHKPLLIKTEPSDFSTGTTTCPSVSTYTRSTSMTARSPGPAYVLRPGHNVPASATKTRQRPSLDSSFGSAKKRWLRQAMSEGSSNGPSSGCNSPAPSGGSLSPGLTGMNGPDSPQCVSPTGSISSLSGKETGGELMTPLKKRRLRVSISGEGSMSPMPMTPPPSAGETDCAAKQGPRPKIVINDAMRNGFKPLYSPVTPVTPGTPQFENISSPENSPAHDHDLNNDSNPPQYYAFQVVLSKQASVDSLSSLASSVTSASGMDRLQKSGSETSLLRARMSSLDLKHEGRESCVHGDSSRDLMPREEEKEVEENSTAISEYSEPANIPMECDPSGRERTDLSSSLSESQVSMQTLQNSCSSLNDSHASSLSLSGSLYQRTLSERLDTRTDSQRDGDPRPRYSSPFKKEMASPEFHRSLSDGTLLQDERKPEYESRPGQRSSDLEATRSMNCYGEPKVVANEERTIPSGGDFLRQDSVSLSSSLPSLYAGRSEEPIGSAPTTPVSLGPGPVVHEDGGSKPIAKRKVSFKVSLLEYRKRKQQGQKDGNSSANSSAASTPTKLAPDSAPSTPIKNPMMSPLSTMPSYEHTVEAECKDKEKDRKAVDDLDKRWSTPTSVERIREEDPLQRFRRELKQSTERGMEKKDKDRKEKEPGHVYSRSVSVDSSHSPVRSHSPAPPPPPPSNRTPPPPPPPIKNGPDARYNHDKLSEGETKDPRSYVKSPVASSPVQAGASPSLSRYNPRQEFRSPSAQQQQQPQPIPTTYPAQPRSSSYNPPAAGTPGYQEQPPAPPQQQQQQQQQPQQQQSYYSRPPPQQYHATSAPSGTTYPPAPQQTYTSYQQQYPSQYQQPQTYPQYSAQAQSAQGSQYYTQQAYSGEYHQQQATPSGAYGHNPIGTAPTPPPPRPPYPQSYSASSSYYRQ*

>jgi|Brafl1|132192|estExt_fgenesh2_pg.C_6520014

MARGRSKKNKHVKGAASPQVSKTKQTEPTMQGEEKADTQVVITDFFPKSPENKALSSSPHRIAKELIDIAKATEDVVVTSLTLIIILACLLVLCISQLISTGLVTPETTPTKTKAKPEKVQISQLKPKSLESGLGDTTTAEPGVREALAAVSDQQDQTPDTKEMSAATSAEGRGRKNGKKVPSTSVARPASARKRRDRKPVKSHHIDDYFVRRSARKSQAELKQEKEEWIEQQILANAEEGMEVVDIEGKGRGVVATKAFGRGDFVVEYAGDLIDTKTAKDREAKYAEDPSTGCYMYYFKYRNKTYW*

j>jgi|Brafl1|174600|gw.442.67.1

HDYLTGRYLVANGDLPMGHLVISEEPILTGPSYTTSALCLGCFDSVDGSYRCKGCNWPLCNRKCETAGSHQLEVCTHDYSSLSCAMLKTTDIDITDFVGPNTSYQFITPLRCLLLKSRDPARWKLISGMESHTEDQTREYRWGMEMGRNVVRVIRQTLGMDEFSEHEIMKVCGILTVNAFELFTTGPNKDRDSTPRAWAVYPTTYLMNHDCLANTLTSIDSKNKMHVRTRMPVKKGEALTAEYSECLWGTEIRRHQLHRYKYFWCSCQRCRDPTELGSFISSHRCTNCGGNVVATKPLDFNSTGFRCESCRVIIRGEVLKGTNHQVEEEVFELESKELETPIEEYEDLVERHESILHPNHYAMLRCKKFLAHAYGRQPGYSLAELSEDRLNKKMEMCRTLIDTLEIVSPRFNDDTAHFLYELHAALVEVARRRHDLGLISNQEMVVALTEAKHILHVS

>jgi|Brafl1|210426|e_gw.42.232.1

DAGRMLYAGQDDWVHANCALWSAEVYEEADGSLQNVHTAISRGRQMRCERCNKLGATVGCCTRGCPANFHFMCARLEDCLFQEDKKVFCKEHKDKVDGELVKEDNFEVHRRVCVNMDNIKLNRKWMRGLESTIIQVLAGKVFMVFSLDIMQKER*

>jgi|Brafl1|218621|e_gw.75.16.1

PEPLRFNPRDNLMEMEVLYEFLKTGIDLEDVKYLQSSYDSLLQQDNSGVDWLNDTHWWVYHPHILSNYGSAHINWQEYHSSTVSCTKQKVVAMCKCASGVFLCPQESQNKAKQATQSSREARSNQRRLLASFGASCGDISDLLRFNQLKFRKKQLSFRKSRIHDWGLFALEPIAAEEMVIEYVGQCIRQTIADERERRYEEQGIGSSYLFRVDHDMIIDATKNGNLARFINHCCNPNCYAKIITVEGYKKIVIYSRRDIAVNEEITYDYKFPIEDEKIPCLCGAENCRGTLN*

>jgi|Brafl1|231117|e_gw.278.13.1

IEGKGRGVVATKAFGRGDFVVEYAGDLIDTKMAKDREAKYAEDPSTGCYMYYFKYRNKTYCVDATAESGRLGRLINHSAKHKNLMTRSISAGGLPRLILVAARDIQPGEELQYDYGDRSKASLESHPWLAC*

>jgi|Brafl1|231171|e_gw.279.30.1

MVVEEDSQHVPPPRSAPSTGMSSRELCENDDLATSIVLDPYLGFMTHKMNTSVQYNTVHLLVFFFYCSRHVVMFRPVRPGKSEDHKQILEKLRKSGNTEKAFTDLTQGEWIWKYLQHKTQHQKKIFKSHVVRYLHMFHPDAGFEVVPCDRYSLEKHGAKILATKAWTKNEKIPMLVGCIAEITAEEENQLLQPGVNDFSVMFSTRKNCAQLWLGPAAFINHDCRSNCKFVSTGRDTACVKVLRDIEAGEEITCYYGEGFFGDDNSYCECETCERRGTGAFASREGNKEKVHNTKYGLRETDKRIQRLRK

>jgi|Brafl1|235988|e_gw.331.26.1

MVVEEDSQHVPPPRSAPSTGMSSRELCENDDLATSIVLDPYLGFMTHKMNTSLYPSGCRHYLITVHVFRHTSATSLFRPVRPGKSEDHKQILEKLRKSGNTEKAFTDLTQGEWIWKYLQHKTQHQKKIFKSHVVRYLHMFHPDAGFEVVPCDRYSLEKHGAKILATKAWTKNEKIPMLVGCIAEITAEEENQLLQPGVNDFSVMFSTRKNCAQLWLGPAAFINHDCRSNCKFVSTGRDTACVKVLRDIEAGEEITCYYGEGFFGDDNSYCECETCERRGTGAFASREGNKEKVHNTKYGLRETDKRIQRLRK

>jgi|Brafl1|236789|e_gw.339.60.1

QFKATPLHCSASGGHVGVAELLLKAGARLDITDDVGDTPLHRAASRGHVGVAELLMKAGARVDSRISGKGSTPLHAAASGGHVGVAELLLEAGARVGSWDRFGATPLHKAASGGHVGVAELLLEAGARVDSTDQVGATPLHKAASGGHVGVAELLLKAGARVDIMNKVRVVAYYW*

>jgi|Brafl1|245427|e_gw.453.23.1

MYNVIFLCLICLVSLAHEESFVRFFQWLHRNGCRNVPLKPAVFPETGRGMMATKALKHEELMLVIPERLLITMDAIMDSYIAPYIERADPRLTPTQALAVFLMCEKYRREKSFWRPYIDILPEEYSCPTFFTEDDFRLLPNSLRGKAKAKKYECHKEYKELAPFFKMLADLFPDQEDAFNFKDFKWAWSAIKTRALDVPIGRESCRHLRDAEDTPTPIMFPLVDSINHAAQAKIRHRYNEKSRCLESRTETVYRRHAEVMNSYGRADNDNLLLEFGFVVPGNP

>jgi|Brafl1|250067|e_gw.555.14.1

WKKRVKSEYMRLRQLKRFRRADEVKMVWGANRGNVDMVLRRQAEQLEEKPLQHLSSPAMFDHSPITRQVYEPKQTTVLKVMNSVPSIPVMYTWVPLQQNYMVEDETVLHNIPYMGDEVLDQDGSFIEELIKNYDGKVHGEREGGFINDEIFVELVRNLSDQSTKDNEEASEDTPDDTTDSLKDASGTDSGSRRRFPCDQIFEAISSMFPDKGSAEELREKYKELIEQQDPTILPPECTPNVDGPNAQSVSREQSLHSFHTLFCRRCYKYDCFLHPYHPTPSQIKRKNADANKEVTEPCGAECFLHLVEKIGKPDPVPSPSRSKKKGRFGGGGKGKSSESDSGSKKEGEGDEGSMEDDSDDSSSADAATPIPPQPVPLPEGWNGADASLFRVLRAVYFNNYCSIAQLIGTKSCKQVYEFAQWEGSTDDLLAEKNTTPPRKKKRKHRLWAAHCRKIQLKKDSSSSHVYNYQPCDHPGQPCDSSCPCIMCQNFCEKFCQCSLDCQNRFPGCRCKAQCNTKQCPCYLAVRECDPDLCLTCGASDFSSNDKISCKNVSIQRGLRKHLLLAPSDVAGWGIFLKTSAQKNEFISEYCGEIISQDEADRRGKVYDKYMCSFLFNLNNDFVVDATRKGNKIRFANHSVNPNCYAKVMMVNGDHRIGIFAKRPIQPGEELFFDYRYSQNDAMKYVGIERELEVA*

>jgi|Brafl1|250354|e_gw.562.18.1

TEWLLEAIHKIKKQKQRPCEERICNAVQASHRVARDLILQHLETSVQEGAILKVINKGVCSYKDPQKSPGSKSRTSSSHTHKVDIVNLLKDAICELQEPAGSILKNIEKFLWKKHHAELRDKSEFSNQLRLAAKREVNAGRLVKDGRLFRLPAKKLDLGAGPSCFDYLSSSPSSPGSVSSIDIPRASPMPVCSFCLGTAECNRDGQAEELLSCADCGNSGHPSCLKYSPQLTAKVRSMRWQCIDCKTCTACENKNDLDNILFCDACDRGFHMKCCNPPLTKMPKGNWECTLCEDNPTNDQEELKQLANSIKKKYKKKSASTSLLPLMKNGAEESSCSEVRSKQPYFADIRDKTKGQGSPAHRKDSSKGRGGHDKPLSSDEGSAAAKPKGLVDGLTRFFTPGNKRKTSRSQSVHDDVFSLSCDEAVLTGSASHGTNGQEEPVTAAGLLDTTFRPQGHYQTPGRGQVKGLFDGLSHIFTTQGETRKRSLPLYAPPKKISRPDKHFEPFGFLSSEEGLQSSDLESCGQGPALDLLGETRDKVMMLAPKSRQDEKLVSSPAGAGSPYKGRGGGRDRKPLGKNCSCLANLVFSSGPGSDKAALPGVNDEDVKLFKKAQEIALTQMQPIAPELSIEAGTRSPAVIEFGRHEIHTWYSSPYPQEYARLPKLFLCEFCLKYMKSRSILKRHVIKCGWHHPPANEIYRKNNLSVFEVDGNVNKIYCQNLCLLAKLFLDHKTLYYDVEPFLFYVLTFNDKKGCHLVGYFSKEKHSQQKYNVSCIMTMPHFQRRGFGRFLIEFSKSSQQCLCKQCAVSVP*

>jgi|Brafl1|258045|e_gw.928.10.1

MATKKRGRTWRKRKRKTRERDKRPVSQGKDRELINLQQFMKTNNFKGCPLKPAIFPGTGRGTMAARNIRAGEVIIAVPRNILISCRTVLSSKLGTELKKWSGTSRFTCAQVLSLFLLLEKNKGNTSFWYPYIRSLPNSFTTPVYFTESELNALSPSLQEKARDLKKELLHAFNDLEPFVTSCLPELDSTFTFDAFRWAWSVLKTRTLYQEDCRSPYLSNKEPQTSTLVPMLDLINHSPSAKARFGYNVNTSCYEVRVLESYRKYDQVFISYGFEENTELMLKFGFFVPENP

>jgi|Brafl1|277814|estExt_gwp.C_390306

MAGPSKKKIKISEAVDKEDKKLDSFLQWCAKEDFQLNPKVHVGREGSCAQYGMVAQEELEEGECLFKVDKSAVLSTETTEIAHLLKEETSLHGDSLHGDSGWVPQILALMYEYTNPNSRWRPYLQLVPDFSQLDQPMFWTEDEIERDLCNTGIPEASSSDLTKMKLEYTSLALPFIRKHRHIFSEEVHSFELYKRMVAFIMAYSFFEPVNGREDEGGKSSLPLMVPMADILNHVAKNNAQLEWDADCLRMVTTRTVAAGEEVFNTFGQLANWQLLHMYGFAEAWPENIYDTVDIPMQVVLEEARRAAGPDDAQLLEEKWQFLEDQGAVSESGSFIAGMEGILTEEEVCESLKVLCMSEEEFREHQEKEGCCDCCASEEERSLSYDQLPNLPEAWRSLLAAAARLCMGKYRNTLQTNEQLLSKESWGKLSKRQQWSLLVCYGQQKLLHRIITSCLPEASQ*

>jgi|Brafl1|278042|estExt_gwp.C_420163

MPKEDKPKLVFEITSDDGFKTTADTIDAAWDQVVEKVQEARINARMKQLSFAAVRGCVMLGVTHESVMNLIEQLAGAKNCIHYKFKYHKYSPHDLAEDEDPPVNPNGCCRAEVLSRRSKFDMFSWLASQHRQLPVLDYNEEEEEVQHKSTRRPTSMDLPMAMRFRYLRQTSREAVGVYRSPIHGRGLFCKRNIDSGEMVIEYAGMVIRSVLTDKRENYYNSKGIGCYMFRIDDYEVVDATMHGNAARFINHSCDPNCYSRVIQVEGKKHIVIFAMRKIYKGEELTYDYKFPIEDQNSKIDCTCGSKRCRKYLN*

>jgi|Brafl1|278502|estExt_gwp.C_490178

MQQPVQPQQLPVTPQPLPAAPVQQQQQQQQQQWEQGQPVSFVVNQQQQQQPPPYDIPQPPIVPPHQIPPQQQQQQQNIVYQQPMAYTVQPTYVQPQPPPRAVLPQPPQVQYIQPGPGDSQFYPQPQGVMIQPNVTYSQSGLVHTQVMQPIQQQAVMVTPQPMVMQPPDIPSPPKPRMLRLPPNWRAARDQEGKVYYYHAVTRQTQWDPPSWDGIGNEPELGGDEADMDLGTPTHDDHHKGKRKPTKADADTSSETARKCKDVFRKKMATFVVSILNPYRKPDCKHGRITSTEDFKHLARKLTHGIMNKELKHCRHVEDLEVNENVKSKAREYVRKYMSKFDGLYQSSPREDL*

>jgi|Brafl1|284593|estExt_gwp.C_3470062

MGKKSKRDRGAGGSAGGGKGGGGGNTKPNGRNKKDVRDVMVLVNELLQKASSPGVPPGKELEEHMSIREVVERIRRKQEGWTLEPVSREQCFPVFLKWLEDHGVKSDAVTIEKFEVGGYGLKAVKDIKAEELFITIPRKLMLTTETARESSLGPLIKKDRILQVMANVSLALHVLCEKYSSNSFWAPYINIFPGTYTTPLYFEEGEMLHLQGSLNFSDVLNQYKSIARQYAYFYKLFQTQPEAAGLPLKECFTFDEYRWAVSTVMTRQNQVPTSDGRHLITALIPMWDMCNHSNGEVSTEFNLGSDSAECLAMREFPTDSQVYIFYGMRSNAEFLIHNGFVYPENVHDRVNVKLGVSKNDSLFAMKAEVLSRAGIHASTSFQVHCGKDPIPPELLVFLRVFTMVEGDLRDLLTSEHQSAYLSCLGRSDCMVTQEQETKAWAFLETRLSLLIRSYRTSIKDVETELQAPDMTYHSRAALQLKLAEMQILSNAAEYAKTQRE

>jgi|Brafl1|289518|estExt_gwp.C_7930004

MADGVEIFSSAEKGRGLCATKVFKPGNLVRAADPYAYVLCNSERGKRCDFCFARKDDMSRCSGCKFARYCDGKCQKAAWTEHKSECKSIKTVKPETPTDSIRLIARIINKTKTDGPGVPGNSIDELQSNLREMPENVKEMFAQLAVVLRMYVGKDVMDDAREIFELFGRMTCNTFSICDPEMQYIGIGIYPKMSLFNHSCKPNCVAVFNGLRMEVRAIQNIQPGEELLISYVEMLAMSSVRKQQLLQQYYFNCKCLRCQDQTKDGMMMAVKCGNINCKKVIIQVDGAYETCKACDHDNEKDPKFWKEVNKVTQFSEEMLAAITSADQRQEPKEGLRLAERLLERQQQVLHNNHLFVLKCLDKALDQAVVLRKWNRALRCALQTIEPYKVHFPAYHPSLGIQYMRIGKLLLYLEKRLAALEALQMAEHILNVTHGKDHPINKELGDLLTQCMDEMRMYQAQKKK*

>jgi|Brafl1|70261|fgenesh2_pg.scaffold_27000081

MFSSLQFQYTPTNVAGPGAPTDPSEIMYEGCDCQTPSCSTDCPCILRYGPTYDKTGCLLTEELEKTFRSKPILECNTSCQCGEPCSNRVAQKGVSLKLEVFRAPHKGWGVRAAERIPLGRFVCEYAGEVLGLEEAKKRTQNMKKEDMNYILTLREHVASGNIIETHIDPTYIGNVGRYINHSCSPNLLMLPVRVDSEVPKLALFAGKDIEVGEELSFDYSGEYGNVVNQGNLQKVTGQSKDSSKLKPCFCGSEMCTGFLPFDPSLYQVH*

>jgi|Brafl1|74375|fgenesh2_pg.scaffold_50000087

MSYMQGVAAGPDGQLVVVDRNERTVTIFPRPELLLTCPKCGIAKYCDEDCQSARKYEQGALGPLCDLCPHTKELKDSSELRIQLEKLSRYVNEDILPDRAQLESLYGKTTCNCFAIHNLDLREIGVGLYPQAAMINHSCKSNCVSTFRGPTLQIRALVDIQPGEEVCYSYTEKGNVTHERRDELRKYFFECQCPHCLDTDRDAIMKSVKCPSCQGQVKPTSSDRYEKCSSCGFTDFTTEFYEDLEIYIHVEFDLLFRENCLVELDKILHPDNIHVVRILVGAFAASVKLEEWTKAIDYGKRLDRAFGLYLPPNEPDTGLLYYKMGKAYYHLDDIENAVTSLRKAKTLLSIAYGRDSQLVDYAQDWLELCGDYGSSSEEEETDSETSCPSS*

>jgi|Brafl1|74594|fgenesh2_pg.scaffold_52000019

MEAKQKMSNGDVKKAGKLAEEAMTMSDMLEKFKSELESAGQDDNAEFNALRLCSQDPYPGDGEKAEGEEAWLQEHSYTDPAWIMRGRRHGYRSTATRTPHGSWRGRVLSSLGQYVLSEVWLRRASRLTEGNDYEALLLFQQTRIHRFYDPLTEGTSVQKMSNGDVKKAGKLAEEAMTMSDMLEKFKSELESAGQDDNAEFNALRLCSQDPYPGDGEKAEGEEAWLQEHSYTDPAWIMERERDPTAAAMEAEVAQVLRDTGSHEKALEHFTLALELDPGVLDYWALRAQANFTLKNYREAFRDCISVHKDIRPVAMWKIGGRVLSSLGQYVLSEVWLRRASRLTEGNDYEALLLFQQTRIHRFYDPLTEGTSVQVRFTKYGRAVFCTEDVAEGQELFRDTPLVSSQTDDSAKAHPACSHCAVSLLTAEDYFGMDTFRRMNKAQKAIIKKAWPKVTAYPCPHCKREKYCSLECRTHAWRQHHCHLCPSINPPAAKLYDFCAKGTTQEKGMWNSMFSPMIMARIWANILTRVKELGVKGEPTKDQWARAKEPYRRFLGFGVSGFVKQIPKMLKIMQAIFQNTEIKYKIDELEFERRYYQVACNVQSFGPPCVTWHEFVAEFHRTARPGENHRRVAQEMRGEPKDVTFGGLYALQSSLNHSCDKNVDVMDAVVDGKPGVVIRAKQPIKKGGELYTTYIDTSMQRPQRRAWLYRAYHFWCECQRCKYEGDDCSICTQCGKKARKDSPFSVCSRCHRAWYCSPQCQKVAWKAGHKKICKAWPTN*

>jgi|Brafl1|75068|fgenesh2_pg.scaffold_55000050

MATSERWKLFQESCKKELGRRLPEFSRLESDEERAKFCLALDCWDGDTLTKLIEKLQSLKGKVGKSTEKANHLRDKGNAAYKAQKFQEAFTLYTDSISYSPVTHVNDDNGTECLSLALANRSAVLFHLQEYKLCLQDIQASIDSGYPENLQYKLLHRKVQALFKLGKKDDAKKEVKGTLEAIEKADIPEDRKEAMRKELKLAGRNISKQEEGKEGEESAEEVKVPKLQYGESQQVAHASGGVEMREEEDKGRMLVAQKAFEPGSVLIVEQPYAAVLLQKHHSTHCHTCVTPVLVPHPCRGCQYVQYCSGTCEEQAWREYHRGCQYVQYCSRTCEDQAWKEYHSYECEHWHLLQMVETFAQLSLRLLLTAAARGEKHPSADMESPATASKPSDQAKLCTDKVSPTSDGAKTVQIDSETGSTSEQPGDLSVQTDVIEENPPSAGMESPTTADKPSDQNKLCTDDLSSTSEEAKTDSETESTLEQASNMSQAESTPEQASNLSVKTDSVQNSMQDVELHRGNYSSVYNLMTHTEHHSVEQLLTQMMVSCLMCKCLGVDMCVEVVKKLGLEGGNCTGATEGGGCGENKEGGDCAKSEEGVVCVEKMAALLCHHMQQLRCNAQAITTLQEQDSVSLLEDKQVRLATAVFPTEALLNHSCRPNVFVSFQGKTLIVRAVSHIKPGEELLHCYGPHAGRMVYGERQAALKEQYFFSCSCDACQEQVGNPNTVDMFSAYKCPVCNNAAKLQDKKLVCTSPNCDAQGDTDDIKTTSKKIQDLFVQSSAFLEEGQIQEAVSGLKQCLVLQWKILHPSNKDIARTHDALARCYATSGDCKKSVEHLKKSLSTVELQFGACSVELAHELHKLAQLQFNGQQVAECLDTIERALTIFGCHYGDHHPKSKELRAMKDCLIDVLV*

>jgi|Brafl1|76037|fgenesh2_pg.scaffold_61000131

MNTPLEGVAPTNGTRDSMAAGSDVWQHIDCMGISRDRIPETYLCERFLLKNMGLLLNFLEMIGKSGQTDSLVTNGLWNFMVHRTPNGFNSLNNEDANEPWDYSPEQKRKRNKSGQQSGGDSESSTGRQRKVSPLRVSLNHNASTTREERKLEAVMRTFERMEKRAARSRAAHARIEKQKQTKVTPNENKEGEESMSKDMEDLPKQTATPHRQIRKRRRSQSRRNHAARGRQRLNSTCSSDMPMSPITETAANTEVVTTEIELKLSAPTTPVSALSPSIPSPSMAQELTVSTVSSVNVVPQPSPAALGKGFKFPKTKKFFVNEWLNEKANEQSAHKPLMIKTEPSDFSTGTTTCPSVSTYTRSTSMTARSPGPAYVLRPGHNVPASATKTRQRPSLDSSFGSAKKRWLRQAMSEGSSNGPSSGCNSPAPSGGSLSPGLTGMNGPDSPQCVSPTGSISSLSGKETGGELMTPLKKRRLRVSISGEGSMSPMPMTPPPSAGETDCAAKQGPRPKIVINDAMRNGFKPLYSPVTPVTPGTPQFENISSPENSPAHDHDLNNDSNPPQYYAFQRIREEDPLQRFRRELKQSTERGMEKKDKDRKEKEPGHVYSRSVSVDSSHSPVRSHSPAPPPPPSSKHTPPPPPSKRRTPPPPPPPIKNGPDARYNHDKLSEGETKDPRSYVKSPVASSPVQAGASPSLSRYNPRQEFRSPSAQQQQQPQPIPTTCWEYKTHSGAMEQCYLSKGQEDEVLTTMLKGRTAGGAGWTEGQPKHRGSKISPKKRTVPQRARTSSAKQPRVGAVPEFPLVLVSATDAAIHVQWPTPGKLGVGSLRHIQVQWSSATCPKVKQTILPPRETGYVIGHCQAGVVYYVRVLLVGGLLEKAVVCKSRQLTVETVGPPDPPVVRVRSCMFYGITVEWSRPRTYGASELCGYDVYIDREFVAKFQPHQHWYMYRLGNPCQQYAFQVQAISKQGDQEFYSSRSAPVKAIWPGVKPPEVTRAPTDRTRAIGVRWDDPELTAVGVKVTGFKVFCEEDTPGENRQQLVQGPLPPSIRHTMFTELKAGASYTIYVEILVEGLPNPVRCKPIREVPAQVPPAPHLRLSVIGQRERQKIENIIFELVNRKNSLQKSVQYQQSLQSHKKPSKEPELERLQSSKKELEKMIYRCFKSLPQYTGVVNLTLEWEVTDTIDAVVRGYIVQVNGEQYGTVLHEGVTKLDIQLDLERRFHSVQLLTVTHYPEDTRQSSTLQVCTEDFLPFAVFCFHHVHKQLARWPDKGCCYYGDSLPEEQQSAPPVNQWLLGRCTPPPACTVYDVMTEGCAPLVTRRQIPRPDVILFWTSWCVASKRVMPFFVDYARENTLRCAFTACCCGTASGHDHMTKLQDLILDRCWRNDVTVRHCCCCQATRVQLERDTPVQRGAMLQRLSSNVPQLFGVPGVPTMVVISADGYLAWRGQICAIDYPSFKATMDEVVEKVLSRRPAPAGYSSVGHSLALGRPGRPRTMSAVSKTSSSQHSTMSEPTMSRLPQTGRAGNINRHSAPILRYRGTQGC*

>jgi|Brafl1|84846|fgenesh2_pg.scaffold_135000067

MFRSPYVPKSPSCDHCMRSMEPAEAMSRRLANSPSLVLPFPQCCAVKLEQHVTCPHCQKYTLPSRNGQYHDDCQNDSHGQAACDHCMRSMETAEAMSRRLANSHSLVLPFPQCCAVKLEQHVTCPHCQDQLELLRGLLTEALYEESLDQWFTPDGFRSIFAMIGRNGQGIGTSSLSVYVHNCDALELPSQDREKLDAFIDQLYVDMEHGNHSCEPTAEPSFDESNYVLSMRALRDITEGEELFICYLDECERTRSRHSRQKLLRENYLFSCTCEKCTREAEDPDVTSSEEEEDEGDGESDEQ*

>jgi|Brafl1|87433|fgenesh2_pg.scaffold_161000061

MSGTGDQPPSPVAGPSSDSAAPSTSTSVEPSPLAVFLEWKKRVKSEYMRLRQLKRFRRADEVKVVVEFGHADEPKQTTVLKVMNSVPSIPVMYTWVPLQQNYMVEDETVLHNIPYMGDEVLDQDGSFIEELIKNYDGKVHGEREGGFINDEIFVELVRNLSELEAPKSSSSSEAEKADSQSTKDNEEASEDTPDDTTDSLKDASGTDSGSRRRFPCDQIFEAISSMFPDKGSAEELREKYKELIEQQDPTILPPECTPNVDGPNAQSVSREQSLHSFHTLFCRRCYKYDCFLHPYHPTPSQIKRKNADANKEVTEPCGAECFLHLVEKIGKPDPVPSPSRSKKKGRFGGGGKGKSSESDSGSKKEGEGDEGSMEDDCSSMSQPESTSAQDAASPTQADDSSSADAATPIPPQPVPLPDGWNGADASLFRVLRAVYFNNYCSIAQLIGTKSCKQVYEFAQWEGSTDDLLAEKNTTPPRKKKRKHRLWAAHCRKIQLKKDSSSSHVYNYQPCDHPGQPCDSSCPCIMCQNFCEKFCQCSLDCQNRFPGCRCKAQCNTKQCPCYLAVRECDPDLCLTCGASDFSSNDKISCKNNRWKVDSLAQSAAFMILATLTPAAAAAEVDAPLVE*

>jgi|Brafl1|89216|fgenesh2_pg.scaffold_184000033

MATSERWKLFQESCKKELGRRLPEFSRLESDEERAKFCLALDCWDGDTLTKLIEKLQSLKGKVGKSTERANHLRDKGNAAYKAQKFQEAFTLYTDSISYSPVTHKNDDNGTECLSLALANRSAVLFHLQEYKLCLQDIQASIDAGYPENLQYKLLHRKVQALFKLGRKDDAKKEVKGALEAIEKADIPEDRKEVMRKELKLAGRNISKQEEGKEDDGSAEEVKVPKLQYGESQQVAHASGGVEMRQEADKGRMLVAQKDFEPGSVLIVEQPYAAVLLQKHHSTHCHTCVTPVLVPHPCRGCQYVQYCSRTCEEQAWREYHSYECEHWHLLQMVETFAQLSLRLLLTAAARGEKHPSADMESPATASKPSDQAKLCTDKVSSTFDGAKTVQTDSETESTSEQPGDLSVQTDVIEENPPSAGMESPTTADKPSDQNKLCTDDLSSTSKEAKTDSETESTLEQASYKSETESTPEQASNMSVKTDSLQNTMQNVELHRANYSSVYNLMTHTEHHSVEQLLTQMMVSCLMCKCLGVDMCVEVVKKLGLEGGNCTGATEGGGCGENKEGGDCAKSEEGVVCVEKMAALLCHHMQQLRCNAQAITTLQEQDSVSLLEDKQVRLATAVFPTEALLNHSCRPNVFVSFQGKTLIVRAVSHIKPGEELLHCYGPHAGRMGYGERQAALKEQYFFSCRCDACQEQVGNPNTVDMFSAYKCPVCSNAAKLQDNKLICTSPNCGAQGDTDDIKTTSKKIQDLFVQSSALLEEGQIQEAVSGLKQCLVLQWKILHPSNKDIARTHDALARCYATSSDCKKSVEHLNKSLSTVELQFGACSVELAHELHKLAQLQFNGQQVAECLDTIERALTIFGCHYGDHHPKSKELKAMKDCLIDVLV*

>jgi|Brafl1|90347|fgenesh2_pg.scaffold_197000014

MEREREKKREREREREYGTEMKEEICAQSKTECSFKSTDCSMDPDTWNKEETTCSTPLPSAGVVGTDLKSCCTSTHLLVTMATDGHAEGIEMSDENFYENLDDIISGLVNEKLESSGWVEGLPPGMAAIERDLGSLEAEDDEVMVVSEGPATQQQQLQQPQQQAQQRQEQEHQSELPSTPQAATEEETVPTAPQPPQTESSVPTTNQESGTSSEQPIADQNEEQVQREVAPGTSDPVSVELAVGMRLMGKKKDDIWYQGTLIKIVPDEKGLIKYKVKYDGKGKGLLSGNHIAFSETVGQGVLHAGSRIVGQYVDEEGDSVQYLYAGIVAELPSKMNRSRYLIFFDDGFATYLKRDQIHQRAMVKVKVGQKIRTEWNGQWWDARVDSVDGSLVKMYYPTDKRSEWIYRGSTRLWPLYEAFAHQEQAQTEGSKSIRSHTMGKTRGPHIEYTRAEEEAAKRKTTGAAQKKPASASASSASQSKPPSRSSTPRSSSTVDSSSLSFEKLLTEDIEKLQAGSKLVPSTSSELQNSGGGMGRPVANREAWFQIAGATQKRTVTSHQSNTTVKQTVTNQQSNTVVKQTVTSTVKQTVTTTQPPVTTTALRINRPLKKQVARKSTGPRSRKPFPDQYVPPSVGRKDIASILQKRLMEEGDSNSRSSEDSVVDITVETDPMEVPTPKMVKFSPHQCTPACVSHVRPTNPAQYRGRGTWPHINPLKVPILYQWERYIGKKRPGGTRDVSYRTPCARMVRNVRDVSWYLMQTKTDFLSIDQFCFDPYVMLDNMRPEKYFVKIPDISNGNEDVPISCVNEINHEHPDNVGYTKQRLPTPGVELNLDPDFLVSCDCTDNCQNANKR*

>jgi|Brafl1|90370|fgenesh2_pg.scaffold_197000037

MAYFQALFDRICGDLQRSTQEMSSDFLQLSTNQQRVSYALSIPSVHSYIKVKPMFKGKSAMEATKLREFGNKMFGQKDYDSALQMYSESVLKAPFDPNINHPVAHGNMNGSSHGRAQCDKNDVTADDGNEFSLALANRRVSAPLPCQKCSTARYCSHECADLAWTQYHGVECQYLGLIHSSGVGGMGHLALRLVNRTGFHFLQEFERTLEDKISRKVNEPQKDAQDRNSGNTDYKNFPGLNDEGVYTNDYNSVYNLENHSSERKPSDLFRRTVMAIFLLRILQNGGFFRVPAEEVAEEDQLLVASYLQSEQRGRHAVAAQDIRVGDVLIVEKPYGSVVLPEQGDTHCDYCCSCQPCAENWPLFYDMVQDLQRRNKLPKLKCRQCFSVLPGSEDKVLNKMKCAACGEEQELQVQRTQLDSIVDEVFTKVGVVVTGEVESSLPALERCLERMEKLVARPYGYYDSCQEAIKQCYGIVGNCYERTTS*

>jgi|Brafl1|91018|fgenesh2_pg.scaffold_204000062

MLNTYTPTNIAGPGAPTDPSEIMYEGCECQTPSCSSDCPCILRYGPTYEKTGCLLTEELEKTLRSKPILECNTSCQCWEQCSNRVAQKGVSLKLEVFCTAHKGWGVRAAERIPLGRFVCEYAGEVLGLEEAKKRTQNMKQDDMNYILTLREHVASGNIIETHIDPTYIGNVGRYINHSCSPNLLMLPVRVDSEVPKLALFAGKDIAVGEELSFDYSGEYGNVVNQGKLQKVTEHSTDLSKLKPCFCGSEMCTGFLPFDPSLYQVH*

>jgi|Brafl1|91548|fgenesh2_pg.scaffold_211000010

MDPRVAVPVGLLVLLAVAAGGTAQDPCSSYRVLNEALRNVRYNTPHGTYRCDSWFAGEWYRFMGAAGTQMPTQAPPDHRCGTASPMWMNGQHPSLADGEVSRQACAVGWNSNTCWYQACAHNELNTCWRQTTIHTRNPRALRGLEYLRSVCTWYFSCCHANGKTKCPKTPNYKAGGCSSAPCLCEVNGLLPFLADSVPYGVCLVLNYAGELSVLRAGGLRCWMTMRWSGTSRFTCAQVLSLFLLLEKNKGKDSFWYPYIRSLPNSFTTPVYFTESELNALSPSLQEKARDLKKELLHAFNDLEPFVTSCLPELDSTFTFDAFRWAWSVLKTRTLYQEDCRSPYLSNKEPQTSTLVPMLDLINHSPSAKARFGYNVNTSCYEVRVLEPYRKYDQVFISYGFEENTELMLKFGFFVPENPKDFMKINLSEMLESLPQINDEERKNKVDLLFDSGLLRVLEREIQNADLVEFVLRLLRPEDRNNAVIAFLYALATNEGLGNPSGWSAWVTLILEIIFLVACVLLLLGVKKGNLTFMKIWLVIAIILVIWEIVQIIVRSVQVATNEGFGEALAQALGVNWVLTLIFIGVKIYGILVVYSYTQTATVVG*

>jgi|Brafl1|94352|fgenesh2_pg.scaffold_252000001

MENMQSIRPPAINGYDVFRRLPHDGSKTHTGGSPMMEARQGFRRLPHDGSKTASGGSPMMEVLFMSEEEFREHQEKEGCCDCCASEEERSLSYGRLLSSKFTVHELCQVNPCGVMVNIDVSVITSGGDQLPDLPEAWRSLLAAAARLCMGKYRNTLQTNEQLLSKESWGKLSKRQQWSLLVCYGQQKLLHRIITSCLPEASQ*

>jgi|Brafl1|99284|fgenesh2_pg.scaffold_338000020

MNTPPIANDLPSKSIADLPLDGKSGANFADTFFDPNLNPLAANPVQDMAGVQTVLPPVNMLTEEDAAKSTLSPLKDEQSSVENELATSPSQPEGGAAISMEDKSSPESDRPSGSPEIKLKIKRTFLRGREKLTSSLLSDGSSPEEAAGPSKKRKKRLMETTDNDDQTQEVTSKQSPPGKKGKRGRKKKATDPSTPAIDHPSSAIALTTPDVGTPVLPAASHPKGFLNLQALEERMPPKWLVGDLVWGKVSGHPWWPCMVAYDPVEGVYTKFKGGPIRASRLYHLQFFGDVAERGWVGERSTVKFEGKDQYEQVVEDSIKHITSTTQRNQARKKSAVKPSRKAAWEIALQAAEEAIPLSLLERKQQYTFKYDAAPSPTDEEDTAPQVSTPPGGKKRGRKRKSAVAASGGATSLLSDDTDNEEEHLVKKKRKVVKKAPTQGEDSPKKKNKAQKKEVERARFELFCQKQRDQVKVDHPDYAPEQIQEELQMQWNILPDKLKAKYTSKFTATSQNSASDSDFEQPGVKHGPTKQDKKVQPPSPKKRKTAGKSSEADLKSSVDQCINSILALSQSDSAESSYPSTPGQVDSPAAPYESLPVKKRIQRRAEEGGKPDKKAKKNRKPRRDKGDDAGPNAVASEKEEGTQNTDVEELSSQVSDHESEESSAPSSSSGLGRGAWAASKENLCQVCEQVGELLLCEGSCCGAFHLDCIGLQQMPTGTFKCDECISGVHTCFVCRKSEVTTKRCSIPICGKYYHEDCLRKFPNTVFEAKGFRCPLHVCGTCVAVAGGDVKKVKSRGRILARCVRCPTAYHVNDSCIAAGCIQLAQNNIVCSNHFQPVKNQAHHSHVNVSWCFMCSKGGGSLCSDPHQLVGGTAISDSDQLSHRGSYLLQQHLPAAGSGVAWGSLAVTRGGDLLCCEMCPAAFHPQCLGLEDLPEGTWFCRDCSLGKKPLYKEIVWVKLGTYRWWPAEIEHPSKIPQNIYNMPHQVGEFPVRFFGSNDYFWTHQARVFAFQEGDKGSKESATSKGIAKVFKKGVVEATERFKFLQTQKEQKEAQESQRIGKKPPPFRMIKTNKPVGSVQIHTADPSEIQRCECKVTDESPCGPESDCLNRNLMIECHPAGCPAGEKCQNQRFVKRQYPAVESFKTPDGRGWGLKTLVDVKKHDFVYEYVGELIDEEEVQRRIKKAHEDNVTNFYMLTLDKNRIIDAGPKANMSRFMNHSCQPNCETQKWMVNGDIRVGLFAMDDIPTGSELTFNYNLDCLGNEKTPCNCGAPICSGYIGVRPKTAAAAAAEERSKNAKKKQRKRKVAPKNVKKEHEDECFRCSEGGELVMCDRKTCPKAYHLTCLNLTKPPHGKWECPWHHCDVCGKLATVLCDICPNSFCKEHSTDDNVTKHPSAKMVCLEHTEDEVQDSIQALEEKQKKALAVKVESEDENTAANADGAAQKPNGQPKKKNQDKAPSEEVTVKKQKRKPKHKVENGTSGKGGKKQAGKAKGAAKKTARVNKKGKANVDSEQTVKLEAEEVPKVPDDDLDDFEGELMIDCDL*

>Bflo_prdm1

MVIPYAKSCNSLWRACSTRRDKTMRGTTMTEDVLRWNEEDFVKNSTYLVVDQEWEEEGSQQPRAEGTLPRNLYLKFNDQNEVLGVCSKEYIPQGTRFGPLVGQVYCRDEVPKGANRKYFWRVYDGEEFQFYVDGYDITKSNWMRYVNPAHSGEEQNLVACQHDMKIYFFTIKPVLPDTELLVWYCREFAERLNYPPSGELMMQRIKQALLKEPSLPHANPSDQTANNNNDNIPIDFSKGRSTNPSPVSPTNGHAASPQSQVQVPVPNAASISLPITSERQDHKERNGLANGHPDETRYSLKPLPSSFFLPGAVANGTPSTTVVSPTEATKAHIFPSLPGLPKPPALLPNLPYLNGYTPPFPFVYHDAHARFGLLRPPFDNALLRNGHRLPPSDLRMLGTGPVLTPPLGRIGDMPHSMIKVKTEPSDISNNNRERQELSVAQSHYMGSPAGNGRSDANSPSKPRRQIQGYKSLPYPLIKKNGKISYECNVCHKVFGQLSNLKVHLRVHSGERPFKCQTCGKGFTQLAHLQKHHLVHTGEKPHECQICHKRFSSTSNLKTHLRLHSGEKPYQCKLCPAKFTQYVHLKLHKRLHGRDRPYQCQHCHHAYVYRCQLMLHQTKPCTPDDDGNAPAFPPEECDDHCGGCAGECDDDDQRSDVSDGERSAEKHDVGETTDQGYTLLEAAKKHPSMDTSDDGENYRSKGSPSNASDGSGSEESGFGTSNSVLSEGHEEEVPTKENFSKQGVEMPTV*

>Bflo_prdm2

MPPDSEGRELTSSCCFVINEGTAEYAVCITIASPVDNKRNCNERCVKMASSTLDFDAVPDSMVIPAELEVKRSGVSTKNGVWCKRSIPKSVKYGPFKGEKKKKSQVTNPDYMWEIRTGKGWFCVDASDPTKGNWMRYVNSARYFEEQNIVALQQHQRIYYKTIKEIKPGEELLCWFNSSDTAQQELAMEHAGKPKAKKKSKSSKIFGKVVKKRKGKQGKQGKAAATAIKSSSSQEDLTADTSVEETTFDESEAKTDDSQEADTGTEVSEETAEETSVDQESETPDEDDLPIASLRKQQQGTPLKKKEEEETSDDEKLVIDEEGSGDAKEGGKSSESAKQTVPKPFPCTQCGRNFSTKQGLERHIRTHSGEKPYKCSHCGKQFTTSTNMRRHERLHTGDRPHVCPECDHAFIQKGDLKKHMVSQHGKEEADSVEESEKDSQDNGSSPQSGICLKKSEEHPESIREPTPTKDGQSDVEKEVKDEPLGEMMSEIVGDADKQDIDEENEMEEGEGKTGKFVCEICNKRFTKHPNMTRHRKLAHERDQIIRKKKILVEELLEEGERPKKRKRVSQGDSPRSRSSTPGAHPGKGEDAAQYLDGRLEQKEIGKLSPKRIVDGSSSKKKSPIRSGHTVKIHVPRAVRPPTGTPNRGPQHMKSSGMVPISPYTQVHPGSSQKGAAAVQITSPPYGSPQYMSGKGRRMLEPPPAHSGPLVPPHQSSSNRRTDSPPLRSPTAKQSQGQRDQQLAHMHQRGRAPPNVVVGDQHLSPMHRPKQRARAPAPPNVVMAQKASFLARGAAGAGPRKMAPSAQARNVATAQQQQPKQSASRTPTIAQVAKHSPALQKMYAASFPDRMVSPSQNIRSQQLSYLASRVGTVHHGSNTQGPFGQSTPTLQQFQSSSPHQYSSTSQGAKVPSALWSKGKSLSRETQPYRMKPSPELYSHMVESDQPLDLSAPLKKAVTVQEELVFTDDGVLDLSGKKQVEVKPARQQQSSSGLDLSDANSVASCISRVPQYEKTSSYVAGSNSANAASGRSASPSFGLGNLFKCNVCHTPFKSMKDMNQHVAAHAKEWPFKCEFCIQLFKVSSELSQHRASLHGVSKTFMCSLCNREFGFLGNLQNHQRDVHPNMPCSYTVLQPGALRPQNFTDPSKSTPKKEELKRQTEFGSLIQSPKKDQTDGKGKISPVKPVDTSQDIKRNFAGYAMNIINPENPFNRPPQLQTSLYNSAIKCTKCGATFESMPNLHMHIIDCAEGKKTSPGPSPVKKGSPKKPAQDSLVKKLGGPRKLTDGEMAANKIKKKAKNLSKMNQKRKKAQQFQITYDPKKYTGRRRLATEMLDLHKCPGCQKNFSHFSNLQRHLLVCPGKDKIDKDVLDAHLGALKEDEKSRELKEQHMCSNCKRSFTYLASLRKHQQSCDKKFAAAAEGRADAATAATAVPALKKKRSKGRKLVPVDGESTEDSMLETTETEDTGMEEYGETSEEGNAPPTKKKRVIGWKGGRRKKRNFWWKKKRSQMKGEDGMEEGDPQGNGEGSQILNIMRSMSDELQSPTEQPEGAPFGNGGEPLIQGEQEAGTPGPGGDYNEEMKVFDAIEGIAKKVSGGKDSSTKGGAGKNNGANSNNNNNNARGNRVARTNVPSASAAKVETGQGDAAKPSTSQGPFICGDCGSEFRAKCHLVRHSSVHMNRPYKCKICHHSFAAQHNYDNHMRLRHKNAAVKVEKNTTVKVEKS*

>Bflo_prdm3_16

MKTGGFSQGYSSSSVIEMIYQIVDEFGKVKFIVDASEPGTGNWMKYIRSARNYNEQNMVALQINDQMYYKVVKDVEMGEELMVYMKDAMYPDGTMPPNFEEEKRFRCGECDDLFKSKVALRRHQKYACNNSLAIFNSINEDFKSRQTEELTSQLFECTDCDRIFPNMPSLERHVMTGHGSETREFKCDQCPKSFNWKSNLIRHQMSHDEQKRFPCENCDKVFTDPSNLQRHIRSQHVGARSHACPECGKTFATSSGLKQHQHIHSSIKPFTCEVCLKSYTQFSNLCRHKRMHADCRQQIKCRDCGQLFSTMASLNKHRRFCEGRNNFGISMPAMYRAASQSQSGTPLPPSSRMPLRNMPPASLMDYFGTGGLHPPTTVPQPASLSSVQSVPLSQTAAASLLSSRMQPPAVSYPSNTALRSHHPSSASTVSARSTFFPPTSTFSRPPFLNAVSMGPQKALLSSPMAHLLAQPPASAGSTKDTSTGQEEDMSRAKSGANTKERANDLSEGSDVSDVSTPTGSDLETSGGSELDSESEGDGADKKSSMHKEARMAKHQKNDPSVKEEPEDESAFLKPNDQSAAIPTSGADTIKAIASIADKYFGNDGEEMRSIRVQHQEGKPAGAYPTVAEAEKPFDLSVKKSERMAAQATSSGEDQPLDLSTHPRKGDVDQARNMAESRKSHAFGSLMATTYTSSQRGNPRLTYARPSPLAMDPIYRVEKRKFTDRIYSGIQEKYMRHANYPFSASEHLIQNGAAYEMGNRGGGMMPVSEFPPMASQLADPVVRGKGKDRYTCRYCGKLFPRSANLTRHLRTHTGEQPYRCKYCDRSFSISSNLQRHVRNIHNKEKPFKCPQCERCFGQQTNLDRHLRKHEQEDQDAASGKSPTSAEDLAELADKDESYFEQIQNFIESPTITPRKENAEGNGNLEKKFSPSNGHVAEQMAAGSPPALINGNKQSPPPDNTPCEEDDMMVDKEAETHKVNGHPKHAEEEEKKTAGVNGQHGPLGKLLNGDPLDYKPISETTMDDISVDVVDYYGGVMGDAESDGDRSNRQRRSKTQAYSMMLSLSDEEEMAMDDKHRGPRLRSMRRSLRVGDYSVWKALTNTRKGSRDCQSSSNPQNNQQTEEALRRNQDVSGTRLDRVPVVSGSHDVGNRIHQHAHNQASGMFIGEFSCLVVFKIWVLYHRCAKKSVDVGNQKFNPLVFLAPALCDMTGTSLMYIGLTLTYASSFQMLRGAVIIFTGLLSVAFLGRKLDWHHWCGIFFVLIGLILVGVSDFIFKTPETGVSTNGVITGDLLILMAQVIAAIQMVVEEKFVSGQNIPPMQGVGWEGFFGFTVLTTLLFPFYFIKVGPPFGANPRGVFEDALDGLWQISNNRNIALGVFGTIISIAFFNFAGLSVTKEMSATTRMVLDSVRTLVIWVFSLAVGWEDFQYLQAIGFVILLSGTAVYNDMLFVPLLRRYGILGPAHQVSLDEKRPLIQDNDVEPNYP*

>Bflo_prdm4

MYCSFQIFRDERSSHFIDARDENEANWMMFVKRARTSLEQNLVAHQCGGEIFFTSCKDIAEGEELLMWFAGNYAKLTGVTSKPEQSYKCCSCERQFADLGALGRHTKYAHPDMTGRKWKCDLCERAFTSSSKLQVHIMVHTKVKPHKCNYCEKTFTDPSNLRTHLTIHTGVKKHACSVCNKTFRQKAHLLSHMVTHTGEKKMKCQFCDKMFSRQSDVKQHMYMHTRDREVKCEECGKIFWRLQHLKKHMKSHTGERNFPCDRCSKAFFTKYHLNRHKKACKGRNAQQ

>Bflo_prdm6

MDVLPSEHQTLILTNNGTDIGFPYLTAQDMSIMLVQLPSNTNNPPSDGVWCVPRLSITVRPGVSDRRLTGEWPQPPRGQHCSCETTMMQVHQPDVVLLARTYSFTPEDVDFYLYGRNPVRTVQLPGLKRKPQDPQWCDLCKEDHQGECPVHGPLHSLRRMVSGAPTDQKQHYQQYPYAVTSLPDEVTLCHSSIPGEGYGICATRTIPVGTWIGPYEGVRMRSGEIPCAVKTTHFWEMYDNGMFNHYIDGTDVRRASWMRYIRCARHRAEQNMVATQYKGCIFYKIFREIKTGEELLVWYDPDSYIQFMGVPLGRRIKDEDTPPSVTMVLQKSGSQSSSEMETDKIETDSSDTASLNGHAASSQNRSSRYNSFRTNPPKHTGIFSARPSRQRRPNNTVTQSSPDQAEVLGQRDAQGTSQLSSSGRNSDFSDWNLWKCGQCFKTFTQRVLLQMHVCSRNPDRPYQCGHCTQAFSQPIDLRNHVVTHSSDRPFKCGYCGRAFAGATTLNNHIRTHTGERPFRCGKCKRRFSQATQLSRHQRTSGDCQQEPGNETETDEQSEASA*

>Bflo_prdmX

MIVKLQQRALKYAMQQAAIGVLPNTSYPQWDDTRRFAQVIADIIDNILRRDKYPAWEYSKPVGGEAVRLISIVRNFARLLSDSMKFHLKQGHLKIRDASVELTFKNFAVSVQVSATMTLNGGSSGIGTSVAAGHTSHYDIVPYYGTKTAVSVQVLQRDLSATMALECIEAAVSVQVLQRDVSATMTLNGRSRSSKDANINAPMVGTYVLEEQEMNNLTEPLLVTLPLSTYYNVSNPRCLTLQIGDKFHPYRWVTDGCKLVRRQYNTVLCECRKTGIVTVVTDMYDVNWRLSYQRHFEQEPIAVMGCIACMLLCIGAFCAFYYYKCQADTVEVHKNLAASITLLHLFFVIGIKRTESMLVCRGFAVLIHYFFLTMFTWLMNEAFNLYVVVSNAMHETAVQQRPMARYYVMGWGTYGEVLRHGMGYVWRGTTSWDGVRMARYYVMGWGTYGEVLRVMGWGIRMARYYVMGWVVPGMVVAAFVGINQDSYYDDGNLCWPSSNHVWMLLGPVFGILTITTLVLIFAMKDIVESSYSKDQQANKVVINHAKAVWTQLVLAMVTWTFSFLSLKMVGAILQYLFAIFSTLQVSRTYKAQQELWKQKKAQRRRYRHPMMSLKGKSRYRRMRRSNFSGRRSQERERQRERVVGRVAFFAICEGQRMGQTMDEGREIRGQTMTDERDSTELLTGVWTTADIPPRTVFGPYVSGTDDVDPAVLIGIRTKDKRMVSYAYKKKRGVKFAFQRKPPPTASQQDGGAKRERDREKEWWVGLLSSPYVRVTGSVQPIARRAQTTGAWPALPSPRRLLRGQRMGQTMDEGREIRGQTMTDERDSTELLTGVWTTTDIPPRTVFGPYVSGTDDVDPAVLIGIRTKDKRMVSYAYKVDPGCCREAGETLKWLRLIQPARDRREQNMEAFQRAGKVYFRSVCHIRRDEELLVWYSDDWAVNIGIPDILPSFIAGEKNYQCPHCCKMFQFPNPLRAHMRYKCEKRQSAAAATNCKAGTAIFPSSTAAIPPMTTKPVTVDIDLAVRTARMLKSAPAGVSKSGPGFSFHDLPKNMEEANGLAHGKTGGKTAPTSPESSSLTESLLTVRTSPPPRTSRSPETVSRHSPGGTSGSPQRHGNDSPSDGSRKRPNGDFPFQSSRHSKDMANVSSAKRPRSDSSDSSDSPFITNKSPERQTFQEETTSTGASKPYSPESRKTDTTSSSSHILLGRQVSNDGGSPLTPSSDTDDDGKSAFTEVTSSKSSSVAAKENLTPPLPEHRKSDSESRGTSNKLSSTSGRLLSSLGSIGQVLPASSGGTASAFSQPLRTAGGHHAVSPSYPPELNPFKVTNMDFPLEIKAPIANMAPINANLPPSMYLPRGDFDGPAFLSAAADKPPAPMLFPGMGKIEPRPFIPRPPVIPHVPVPPLISSPTAAVTFNLPTQNWCAKCNASFRMTSDLVYHMRSHHKAEVDPTKRKREEKLRCDVCGESFRERHHLSRHMTSHQ*

>Bflo_prdm7_9

MASVSSASSSGSDDENNDKQAASTSASSNPFANMIDTDFDMSKVPETEDIRIEQYFSKEELAELTKIEMTRYRNMKRNYDVMRMLGLPGKKPYFMEKNRRSLVQPKEPPTPPLTSSESEEDEEWTPELERKKNQRPKKPWFLLPPKKPRMPKPKPQTTGSHPKSKAKKKAEATVSKAPKESDDEDAKTFPGFQPEEVAEMQAATAASIAELEKKMEELELEIQQWEAAGSQDGQEEGSESDISTDLEEELLTTTLQPVKQQEPPKKEPVEDTEEPRKSRYPQRNIPRKDYKEVELPDDDHYLYCEDCNELYEGDCPVHGPLIVVKDKEVPKGVENRAVHTLPDYLSVCPSKIKGAGDGVWLDGKAMPKNFVFGPYDGKITGPEIGMTSGYAWQISKNDKVKYYIDATDITKSSWMRYVNCARNEEEQNLVAFQYYRNIYYRTYKPIPPGTELMVWYGNEYAKDLGIFEEAETKEEEPKQQNQRVHTGAVAGYRCSRCEKLFSTKDNLDRHVTRQVQLQATGAAGVQSGAVAGYRCSRCGKLFSTQDYLDRHVEVHVDIAQVLTVSSQVSQATGAAGEKPHKCHTCGKGFKQKADLKKHQRIHTGEKPYRCEVCGKAFNVKSNLTTHTLTHSGVQPCVCGECGKGFTHAGNLQKHKRIHTGEKPYKCQHCDMRFTMSGDLKRHVIRNHTKEFPHRCKVCNKGFVAPSHMRTHMQNQHRN*

>Bflo_prdm10_15

MENQEGTVWQQQVVTEESQEAGTQQAQYLQQLHTMEAGQTQQYTTADGSTIAVSFAPGVTYEQAQQAVESHVTNTQAAQGQATVTAVSVSDISDEGVEENGEAKTQQQQTITLAVAPDGQVQQVEQQQTIAIAVDAVAAQVQHVVQQQQAMEVAIDPSMTAGQEQQENVSADQWQAGPGAGDLGEGGQQGHVAMKDEVDEEAMDTTPFEDEPEDLWGGPVQRIPSKPVLSKARASLPNQLFLAKSGETTEHGVFTKRALPKRTQFGPVEAKLLKKEEIPEGTFLLKLQHKTEGEEQPDDQPELYYDLTNEDECDWMMFVKPAANHMEQNLVAYQHGNEIFYTTIKPVEPRQELKVWYSTSYASFMGVSVHEVTEEEKEAMREREATWPCFECPRKFMSSEQLQRHLAIHEKVITVRRRRWGKGRRGRRRKVPAGDRDNLECDTCHKVFLRAYSLQRHLVMHSGEKKFNCPVCKKMFSHDYNRTRHLRKHQDRGEGLEIVNQLLEGKSPSESTSEARDEPEEGEWSCRHCVLVFENAELLRLHVQSHPAEELDEDELESEDDWDSDEDEEDDVDENTVKTDANDSQDGSSEQPRESALGKNFPCDVCDKSLGSKQALKYHMRRHEERGEVEMQCPDPECEKSFQQKRDLVTHSTVHGKAKSAHGMVTRRALSLYKCDQCGKAFRDSEKLDKHLLLHVNEEDRPIVCEICNKRFLNNSAIACHMKTHSGKKYYACPFCNEGFDRTETLREHVPVHAVDGVYSCPTCSKTFPEFVQVRKHIRSFHSDKMYQCQVCEKAFPRPDKLKLHMLKHSDRRDFLCANCGKQFKRKDKLKEHMLRMHNPEREARNANRPNKPKAFKPKTPPSDYNSFTFKCRGCMLGFRRRGMLVNHLAKRHPETRPESIPELALPIIKPNRDYFCQYCEKVYKSSSKRKAHILKNHPGAELPPSIRKLRRNPQDISINTHTGTAGTIASAPVCCPHCPKQYSSKAKMTQHIRKKHAELVGTAIPMPAPTVPITPAAQQAIAEAAAADLTYKVIVRYDAPPNEPSEEAVLEHIPQHMVVQAPPQDANVARQTVQRFTVETTGQAIPVPSETMQAADLLTQAMSELSQTLSEYRPQPGEYQTIAAHRVVTTQPTGELVHHQVVPVSIQTADQSQGTVTVDVSQIRQSPTHFQQQALTIQQPISPAPTPPQQQVQQVQQVQQVQQVQQVQQVQQVQQVQQAVQAVQTVVSTTEQVAVTHPIAVSTQAQPQQFIARPWPGTFTGNVTYQ*

>Bflo_prdm12

MKPTLFDRQMYGHYRRNPPTVFPTVPLRKKKPETVLKSPAGELNSLALAQITPELLRTVLYGRWQGRSGETRAVSPRENRVPSPDHQRSPVSPDFLGGCARQSCDRHVNHPEINTVPRRVRFRRRPSEIDSADQSASVFLTRSFRPLSVRNGASDDRPWRQPSNQLVFLLTRSIVGNCQSGADQVRKTELPSLTGLVLPPQLEARSSLIPCRRWGVFARTWIKQGTEMGPYTGRRVNPTDVDPCVENEFMWEVFTPYGDLSHYVDASRQEDRSWMAYVNCARSEQEQNLELFQKGEHIYYRAMKAIPPDEELLVWYRFSPSTFLGIPGVPQPEEFTTKKEDDTVTTELPPAPVTHRLKCVVCNRGFNSRSNLRSHMRIHTLEKPFICKFCQRSFSQSSTLRNHIRLHTGEKPYRCDICHSAYSQLAGLRAHQKSSRHRLPTA

>Bflo_prdm13

KTSSNKTRKGHLCIYCGKLYSRKYGLKIHLRTHTGYKPLKCKVCLRPFGDPSNLNKHIRLHAEGETPYRCPHCGKVLVRRRDLERHVKSRHP

>Bflo_prdm14

GTRFGPFVGKIINISEIKTDDDNSHMWEIFQDGKLSHMIDGRGPTGNWMAYVNCARYAQEQNLIAIQCEGQIYYEACKEIPQGAELLVWYGDCYLQFMGIPVALKEMADSGAGAVAENGEEGYTCDRCGKVFAYKYYRDKHLKYTRCVDQGDRKFPCHLCTRSFEKRDRLRIHILHVHEKHRPHKCSVCGKCFSQSSSLNKHMRVHSGERPYKCVYCSKAFTASSILRTHIRQHSGEKPFKCKHCGKAFASHAAHDSHVRRTHTREKPCACSVCGKFFAQPYELKFHMNTHTGG*

***Capsaspora owczarzaki*  (Filasterea)**

>CAOG_00050T0 | CAOG_00050 | Capsaspora owczarzaki ATCC 30864 hypothetical protein (780 aa)

MIKTTSSRHKQRSYRVPGGGLSHEDANETGDDDSRLGAQPSSSSSHASAAGPADAKTESKKEPASAVDAQVQAKNTSPATEAHPLLPDNHELWTRAWFGSASKSGAAPVAAERVPVAIRNVPAAGNGLFATRAIAPGECIFTEPPIAVAYDADQLDLYCHRCGLMARNQSSALGVVSQSDEPYLSEFECAAVTPCERCKYAHYCSTDCQRTDATRHQPMCDALCRIAPRVPQRSVMLVCTMLWQHQREQQQQQQQQTSSAAAAPQLLAHSAPVSVNEALLRLQTHRDKLSVKEAEEIASGTFLVRHILSSASNQVVIDNLGAGKSSGASKSAISKPQEQYDDDDDDDDEWVDALRAQHDATHAEAAASFHLSSRLDDAPFLESSSGSTNSASAPTDSSDNAFAPEMAFNLACLARSNGFAMDGGVFVAGRASLLNHSCLSNCTVSLRANRVVVRAHSRIAEGQELLVSYRDLLQARVQWQAQVERGYHFACSCLRCTAPLDLTHPFGQFDASLVALEFRGKTPPSPEQLAAEVKRNPALLPSGKPRLLLDPAMHAPEVLDFQQRLASITSSMIEKGDHLAAVSALTQLLLECDPKATSSSKSPPAKTGLALSRTHHLMISAKRKLAMSAMRSQLPQRFDLAVQQFRHIFAPTSLVALLPAPAVVDPMEEYVHALFGALEWRAEHFQLSGAQIGNFDPSVAASRTSNPSCSNLDELYVIAREAYHFARRLGTLSAHTLGKQSSRLASAHLVERNAEIVLSHLLQSGFVPSAKSQDATLRY*

>CAOG_00472T0 | CAOG_00472 | Capsaspora owczarzaki ATCC 30864 hypothetical protein (477 aa)

MAEQQRVDDPVGDRFIAWLRANGATVSPKLTLQATAAFNADSRTQVLHRRVIASAEAGFDKEEELFSIPRKLLLSASTSSIAELLLENKKEACALVGWMPLVVAMMYEITNKDSFWRPYLDLLPETLDTPMFWNDDDLELLEGTSTLSHLGKEDAETIFTEQIVPFMKLHPTHFDLKVHNMALYHRVASVIMAYSFSEDDDEDDDDEDDDEEEDCCDGDANNECCSQKRQKRMEKIAMVPLADMLDHKTGCNNARLFYGKTTLAMSCIEPCAAGHELYNTYGDLSNSELLRKYGFIDDVNEHNSVDIPVEMLEERFESCSFMEEAMEALEEIGCWLPEFHIPADALPPQELEASIALLFQSPKQVRALRALDDEDEIRSFLATLVNKCRRKVSETLLAFGQKRAEEYTTTREEDEERLKESDLTHRQKMALRVRIGERTILHNYISHLKERLETTPPDQETKEPAPAHKNKKARKH*

>CAOG_00526T0 | CAOG_00526 | Capsaspora owczarzaki ATCC 30864 hypothetical protein (177 aa)

MSAEILQNVDTTTRATDLDLPVLRKTLPNGQFGVFTSRPVLAGEVLIVMPRGELAASPDRFTVQVGVNQHITGDQLGLAKFLNHHCDANAQMVVVADVDAPTACALQAVRDIPANHEVTFNYLTTEFDMDEKFTCNCSSLRCYREIRGFRHLSPDSQQLLVKSSQVAPHLLLLLQQ*

>CAOG_00643T0 | CAOG_00643 | Capsaspora owczarzaki ATCC 30864 hypothetical protein (1189 aa)

MSQRTARLSEEDMDTAATATTVSNDQEHQHEHEHQHELEELASPPAAGRHVRPRSMSSTRDEAQQEKEDGRPTTDHKRTRIRRPSSSSSDDEDVEQEQRQQLGEEQQPNQGETTMTQLPVEQPDTPETRMTNFLSWARDMAGGVFDKIELRTTGPEGDRGFFATCDLAPGDELASMPIATIISEQLASRSPVGMAMLSSPMLKRRGVTPIPGRTLICAYLIANRGKLDSPFYHYINILPQTYSDPLWWNDAELDHLDGTNIGGYIQERRNQVRNQFLNVFPVLSREQPALFPKDVFTYEAYLWAFSTCSSRAFPLRVTVNPTTGVESHAIGNPMKEPCVECLLPLLDMMNHQFGASITWFTDETSVRFFTGAKVRKGEQVYNNYGPKSNEELLMGYGFCLPNNEADHVKIQLTVGNDPDGEAKLAILRWHGLSLTHFLHNRSVPVELFSALRVLVMTPAEICMYSTPQQADLTDRVSLANETRMLRTLSTLLLTRLVQLGGESRDGFDRGLLLQPSLDYHVRLALTYRVGQRSILLACSNLIAWRTARNIESVSKAAETDGYLVRQDPLRTEVDAVAAMAVVQLRERTQPPFHYTLAIPQNGIGAIAQHGFSQLLLASSACITRASIRASRLLPGLDHVDGLEPDVEIALYLLAERMRNSESPFSRFILAVEFHSSELTPFLSCKPDSAILARASSGARRIFDPVLRPSRDYDELYDELFPALFQIDGMSDLESGFTRELFRWAMMVVGSLAIDLPLHPGSPAETILPPLLLKPRHSHLACTAFESRSFCVALNGPAATSSHELYLNYNDNDHLALLARHGLVLSSTIANPTDTVAVPISYLFDAAASEIAAARQRVASGKTAYPPTLFCDDWQDAPDSGWDATTEDDEDAEENSAAAEADEATPAGGAENLHSVLRRLPKYISDTLAAFGFDPAQTSLVLGREYGASGARFATSPVAIPLKLKQLAVLLCLSRIQCSYLREQFAEASTTYRQQRSSDAVCFELGSSANPLMLNGVPVGFRDHSPSDPGYGQAASLQTTACKNEVAQPESSTLAMSMQMDEDSFVLAPGASALSPSDLNGGAAASAEALPTTTISFTSVSSRDEENPPHLCPHAFLAAVPIPGYLKRLGARALQVALARIHTDLAGSLQLLDELAIEQGWSTVCKTTIQTILRGRLEIVRDAEAQLSA*

>CAOG_01287T0 | CAOG_01287 | Capsaspora owczarzaki ATCC 30864 hypothetical protein (513 aa)

MAFPPQPGRSVPALAAAILVLHWLLLPSVSFVQAQEGRLVGGTAQVQRIIDLVSWARRAEIEMARVDIRPSTDTSASAKFLFESRGLGLVLNAPARRGEAIVTLPPRARFRVPAFDSALRSLIDEFNEQHDNAIDPMTALALGLMYERSRADSPWRAWLRMLPDPIESMLEWNDVELWPVEQLYVKELREERIRNLEAVYESVITPFIDTYESDLVGVDFTIEAFVWAAVIAQTRGLHESEKNGLSLLPIVDMINHHREPNAVVVASGPNILVRTKTSLKAGEEITIDYEMSSHVLLLLYGFVEMSENLDFYPIRLSWESKDIDYPRRLRLLEGRGLSSPWYEFRLWPSIDEPIDDDLQFAMRVMVANEHELAILEQREKAASPDANSEISIAHERRMLRRLLQQIHEACPVQHAEMDALRTYGLQHHECYRCLSSFAYAVAAKSTIITVRDALTKRLDELEQPQASVAEDGNPHRDWMEPDTIAPADHAPFYDEDAAVQVQQVVYLPQHEL*

>CAOG_01362T0 | CAOG_01362 | Capsaspora owczarzaki ATCC 30864 mixed-lineage leukemia protein (1859 aa)

MLGGVEVEQMASAAAVDDTSPAAANAFPAKTEAVLPANFAPMPNHQAATEAPTAADSHVVVSIADATVDYLLGREASQQQEQQQQQPLVNQPVSSENGLPLQTAAAAAPAIKSPTKTTKSGHKKPSPRAKGHTQPQEAAFSRTPRAAAIVDQSIAATQPAGHRPCRATVNARAPPKYNFEADDAKANLEEDAWDRTSLRASAKPASATTVSRRGRPKVTKPPNASPVSATKLPVVGVNGGPAPTTAATNASPSPRPLQTLTLDQISASMRQAIPTAPVKQPSPFASPSGAPMKTAVRDSTAPRPILPLVSAIPLVASAPLPLEIPESVRLQSLLAQAQQARLPGIAQPTVSHSSSLQSSTLPQAALTQSPFPQSALPRGGMAVNSSASPSVATPTFQAAPPKPRAPQRLERKDRIQITLSDLIDAGLLKPGTVLSSGSAQCLLQADSSVVSQPEGKPYASAQAWLATVYTKEQRPSMWSRVSAKGMVLNIYREMYIKRAESAGPQGSRNKSRSNSSLRTTTSSTITQGNGGAASSSSQTVTPPSDQEQADAMRVRGIDQNGMWVTVTDLGEALLALPLCRGCGTRGTDEETSGMHWCNQCCQPYHDFCVKSSFGDAYESTLKEIAQGSWKCWDCIVCTTCNSSFPEETLVVCDNCAVGRHLGCMDIPLAEVPSGRWLCSQCVKCDSCGAQTPRGMGKTRLPSSFPSSQPCEWMFDYSLCQPCGLLKARGNYCRVCEKVYEDDDYDTPMISCEQCSMWLHTHCVGMDEETYEMYSNDENLAFTCPSCVHSLSGKGSTASTDADALAPRAAFISANDSTSMIFGLESNAEFEGFDKDSEIEEVYSRLRSLYAVITKTSAATPFLHLPSRSSLPDYYKSIKTPMSLEIVRLRIERYIYVISTEMLEDVTQIVINALRYYGPKSPEAGQALEIRRNFLGKFQKLFVASRPITVEELIRRSMEKQPIGAPSLASKAPKVTPSPKASSTTPKAPKRQPETATTPARVTRSSRSGQKSSQEVDAGSNAAVEPSSTGEEPSNDATPMDIDETPNTAAPTPADTVAPAPAEEAILDAESTMVQDAPNSSTAATVATTTHPMDEEAPLEFLVLDTFDPDVDTRIFITPAHPTDFNDSMPTAVEDPRHCSLCSVAGDSPPEESGRLLSVGDLGWAHLNCAIWSSEVSCLDDGHLDGVTAALSRSRAMKCHHCGKTGATIGCAKPRCQLNYHFPCARHARGCVLLTSKTLLCPNHCGTMPPNPSPGTHSSHAPLDMLAAGESGKQTDRPVTTFSIPLQCVSLLTDAVASRTLQKDVRAVVDMSTALAECHSDLSDKLEETLRDELLDRLSSPHSHEAADQFGQDLTEAVQVGRAESATWPSRRNFWNQIPASQRSVARSGTLNVRSFGTISVASMRFSPPTSLYCPGFVSTRLFFHPFEVSRALYLCEIVETVVVHREGLAHAHQQQPISSPNPVVSPSLVFRVTCITTRTIHEGRDPESAWGPYLCAIERMRPHFKIRPDTGHSLFGLTFGYVIERLEALPDAHLCGDAVSRLKSSTSGLAAYQLDWPCYSFRMSSPAPPPRTASLLNMIPERHLLPKNVERLCAHASRCAVYTEPIQRDRFHFMVPPVERKASATKPGSLLVRSMATLDPSLASVSVGAKPRAPTIRRSAVRTGGDSQTGSSVSAALNAAGVAAAAAAAAQEASLDPEATQSIQMPMAMQYRVLRDNYRTYSVVRRSPIHGCGLYAARRLEKDSMVVEYMGERIRDILTDYRERMYDARGIGCYMFRIDDDYIIDATMKANQARFMNHSCEPNCYTRIVNPDGVKRIIYFASRVVLEGEELTVDYKMPIEDVKIPCYCGTRSCRGSMN*

>CAOG_01403T0 | CAOG_01403 | Capsaspora owczarzaki ATCC 30864 hypothetical protein (480 aa)

MSEELDLAKPLRKLAEVDVAATKTDVETAERRWQARSERRHKAAAKANAAAGTSTTAQRAKATQKSSYPAPETTRTEAWARSMDEYFARHSMPVRTRYNPQSGYGLVATRPIRRGEVVVRECIFLGTVCENVKDRVCSTCFRHVLAQPKDRFRAARLPISPESQPPLEGNDSDPCALTVRCEKCVQVYFCSTACRDESKARDLHSEIECNALAFLGTDAIHPNDVISDLLRQAIRILSIRAKRLQLLPLPGEPQLRNSYENIAKNLAYSPTLADDNDHTLKFVVKFANELVPAAVRIPGPEFLNIFHRHQCNAFSMLGPGRMDTMYGAYTACWHLMNHSCAPVLMREHHAFHRPLVDGLPHFEARAVIDVAEGTEITWCYSNIRNPKAERREHLRQFYGFLCECPRCTGPDERRVLEGFPEHRRMLCRRHNCGFVMPGDPSAEDDGILGYCNQCGLVRRAGDDDVDEDDNVDAAQQGHC*

>CAOG_02237T0 | CAOG_02237 | Capsaspora owczarzaki ATCC 30864 hypothetical protein (1633 aa)

MAKSSTASVAGTDGGGGGTPPELPDSATAAATPAAAAALTPSALVQQLHAQQMLANNRQPPSTPVGAGAGKQGPPSSGSRSKPRNSASTSSSNTSKPKRPRSGASASKAPGAAAGKSGTAARKSGPAGTSAQPCAENSDDEQAEDSFQGEYVTRCICGFSHDDGFMICCDRCEVWQHLDCMGLKSGRLPETYYCEHCSPRDVNKSRAMLLQMRKRESMNSSDLSDDEGSAVPPPPPSSRSTPGNASRQRSLSTTVVPVVVAAPPPPPPPSDFASRLLRPLPNGHHSPTVNIEDDVDDSPSAADAPQPDLTSGADVTPLVNSSTTVPPPRLSSALASRLTPLVIPPSTTSKELSSLSTANSTDSVSPSVGELPRMEMCWEALPSNPFNALHQSIQQCIVENEDARAILADEVARLDAERHASRVNVTTDKTSQAPSTESMETDAQATAAAQGVSEPQVILPRDASDVETVLLDASQYNPVSSTLFCNGAPAPVLSIEPVGPNQQRKGVLVADNVSVGRFLCEFLGEITTPAAALHCHEAALAQLRSELVSFAPQHAELADSISPSSVPLPYALFHPRLGLCIDATRHGNDARFIRRSCTPTAQLKQLLVVGDFAPDSSTALPNRLHFGIFAASDMVKGAEVTLAFDFPVDDARFPVECACTSANCAMSDWRQRMLAERSFPGLISSQDALQRKRRASVSQDDQQVLKRMRMISETGTELDADANKSAALDAELVKSEPSDSAPMDTADHALVSSQDASSQHESSAPSSAVPVIETRSPLLDMDPKKMTREERKMYALLKQFEKVEAKQQAANKNAAGSTTPTHNGHAAAMRRTSTTASPAPQRKRGAEAPATPQQAPGASAPPMTPSDAELNRKRSHTAMALPSGTSAPPTPLATPRATSRSRLPSVDASSRPLVLTVAMLNADTSLFPVIETDSPPCTPTRARVLQLAHMSPAAVGVSSATSTSTPATLSGSISQSVFAFPASSTAAPTANAVEAGVTSSRESPAPLVAARFGKKAWAREFAATEQNGSVPSPLASPSVSGSGLSTPVATPLSLKSDMVQPLFEHHHFPLVPVHELLANPASPAGRGKKAWLRDFLDKDTAPPRVVPTSLSMTSGLPSLPFAMEVESSSSTSTLPLPLSSSFVVADASQSLGASSMNVVAPLESSSGSSILLQASSSVAAATPLVASQPSSLEPPSSVDLTNNLPQQGEVHSLAPTQSLPSSLPDTLAAASAPVVSLDGSFAADAALSAPRAAPNTPTKKKLNLGDYFKRKSAPGETPLETPSSESLPPVFPPSASLSASLPVVAEAAVPQSVAKQALPPLPAAVVAPTNSMVMVEDAAAGGAGASSLDRSTISLGQPTMPRPGASAALESNGLDHSSINILERMHQPLSAAPPSTLSATPSASAPSSATSASSTSTSAAPAGMSNVFAGSGFGFPSFGAFQSAPAQAPQQQPRPSGPSGPSTPGQQPAFGAPFMNAGMPTGFPPSSHPMAMNAFNQGPMPGQPPAFGMPMSAGAPGHHGWQGHPQQQQPHNMPPFDWNGSGRGGMDHREAGRDFNADRADSYRGGRPSFDRDFRGAPRGRPYHGNMPYSRDSVERDRDRDQRERERDRDRERDMRDRDRERELRERDRERV*

>CAOG_02820T0 | CAOG_02820 | Capsaspora owczarzaki ATCC 30864 hypothetical protein (615 aa)

MAPTRPTTRAQRGTVLALCAALALILLVCSSLPSSMLLTASASSDVGDVDVDEHGLDFDDLPLEPHVHHEPTDEHDDEKAALKRNVLAKLRRRTGGQIPESLMQGLQDFYTENSIELIKANIRYSPETDFGLYATADIDQGDEIVRAPVTLTIASQYLEDSPLTEEMQRLFGDQQPDELTAIALHILHEKVHKSQSFYSRWIHIGAHNCSMISNGFDCVAVEELNSTVMWDFNEVNELQISEEFVAMMQSLVDHMQEQYHRYFEPVSKARALAGFLSIMDGIIVKPEVFQWAYLTAIARGVPMKSKTGDVSYGIVPGIDWVNHAYDNNAHLDFSMQGRMLGSMTLRATRDIAAGEQIVRNYVPMPNNQLLLRFGFAIRDNPHDFVSVFLDQAVGATQMAARRKAILRRHQLDADFTEFSLLDTKKKYFHPDLLAAVRVVLANPQELRRIEQYQEKHGKLVCKTSKLSMRNEIDVLEFLGEKIIAARSYQWTTLEEDIATLDRSGSSLAPRLRHALIFRIGQKEIYARALEIILEQRHDLLIKYNEYKKNKKPSADDGAALDSDSDSSSPAVDERKPVENDSPAAATARLIDELQQKKKAAKKAKSKPAGDRTEL*

>CAOG_02948T0 | CAOG_02948 | Capsaspora owczarzaki ATCC 30864 hypothetical protein (981 aa)

MAEYTFRELSEYDDVSTDVVVDTLLGFQTHKMAPRYRPRHAVAQTVAAAVTALGDLTPPIVPGTQLSAQHEEIRARRQATAFAILCRDPWAAPYLATKTPEQQDAFKQHMLRYLGVFQPASGVLIVPTNRYSQDSRQGAKLCASRRWHKGEIIPLLCGCIAEITPQEEKSLLRAGQNDFSVMYSTRKEIAQLWLGPAAYINHDCSPSCKFIPTSKNTACVLVCKDIDIGEEITCYYGRHFFGEENEHCECHSCEIGQNGKFSRTDSQSSTDGETDSSESEASSSNNQPSNRNSYNFRRKHKASLTKHHEPLTHPVLALRAAAKVSPLKRAAVSESDTPTAKAKQPQLAEKATAASSAIVKKGAIQLTEEAKRGTKTSAGARASSSAASPKSQATPTTKPKLETPVKSSPASLEPQTAADLSKHQQRQLQSARSKDRELLTLGAVRKLVFVKPFDNPSSWWYPAMIVPTRELDVTMPVPQEGDCVVRYFEDNSFSAVAFKHVEEFAPQSRFYKRLASEFGSSFRTSKGVRLATECYNSENLVRVPKKFIWRHWGQSDLDEQRMAQLRSAGGNLPDPATFGPRSCFVCHTTPAVSRPLRPSDDIPSPLVNVRHESLYAAELVRLEVFSDEHGDLDAQSYSISSADASDSDVSSDTDGDSVSASVVGEDSDVPSEGDNQFDGDESQDVDVENDDDDPNDEAEVDDDDELIDFTDDEADNSGVVDAADVDEEDVHDEEEVNDDEDDETSDDDDDDDEVGSNESDSERAEETSSAMQLSNGTPRKRLSKKSLFVCGRHCEVRYISYPVTYRDWVPYSLVIQPKSRGRPPHVHPPERGVRNRGPLCFRPPEPEPTPSPLLSSKRPRGRPRKYPLGPDGKPIRVPLSSLPSGLAHKTAASKQPRTTTQSPKVQASAMRAPPAAATAPKPPATPKRRPTEQATTPVETMQQAVSTVLSRSGTPHRAKRRFPDEEYAVLTPRSKSPRVN*

>CAOG_03580T0 | CAOG_03580 | Capsaspora owczarzaki ATCC 30864 Setd1a protein (1292 aa)

MSGSAQDDAVARGSDALKDGAANEPSTKDFKVLVDPGLSHDRHAQLERRFDGIDPKTNTPVTVVDPRRGRPPRYTATLDLTIPRFKFDEHSVGDAPNRILFLSELNDNVESSFLLDELRKLGSVETAKVHHHPQTGRHLGIASVVFPSVRECTRLLPLIDGRTFMGKPVKAELDGSETKRRTEYARLTGTAAAPLTQAPAPTPATPTPPPTSRPPPLLSTQPQQPPPPPPPPTQLPELPPPPPPPTEPARLASRWGQTPADTAPLLAEPPKPAAPPSRWGQGPLQKKPSDGGAPGTGLLEWKPSTSGWDDSDVSTATPMATSHASSAGDAHVGNNRYASAQNPIAMAPDSIERPNIIVNVNPQLLSRKWLEDRFRSFYVVSIYQSPGDRIWIVEFFNPNDRDRALNRFDRTTVDGIYLSLSRGPMAPAPVPVPVPAAPLLGLPGTIPVGSMPGGSIPTHAPTGLLPTPGAAKPPLVAAQLIMPPLPPLLPPHLLPPQPAPVIPKLRPLLPEAAAPPPATSSSSSSASSSSKPSSSAVPRQSTTIPPLLPASAAKPTSKPSQPSPRNVPKESLEERVIKILQQDLIAAVARDLRAQHVEKPAFNKLLEWRAKQAATLKDPKRSGDASHSHTAAATDSNNSLTAQSNGEAHSTTAVPPARTVPLFQTNILGLGASGAAGRSAVWALPSFRKPGASERRQEHQPSSSSGSGLSSRLEHGRRGFDAGSQRKDSTRTKPSAKERGSKPARQSSSSGRSERHARVSDSEDDDESDESSQSSSESSLDSDSEDSRSDGKSAKQTPSKHLPHSSKPSKAEAAAEEQSVHANALANQGTQKRLKDEKSSASSARGAKRKAHELDDNDSEAAGLPAAGKPDSRPKSSSRKRAHEDDLDAEAAAASLEPPPVLHDLLLDDISKLSALPTSEGGLARLKLADEQMFASFNGMRMVVSDNPWHQPHLDVSHLDEEDSWYAAAAARYLEARGMDGGAQGFNAAEEVEATWPLNLSNLEQYLSNAAKLPVASDALTHQDRTLKRARLHATGSARSEGFYKPSHEDKKLVMHGAPAYLTGIPGLKFDSTLRNTMYAVTGASDASPQTPGTSSATVSTALASGSPSVLGMANASVNRANRLQLRQFAAVIEQATNSDTFSLDMLKSRKKLLKFQRSGIHAFGLFSQENISANDLVIEYVGEVIRQSISDIREHHYERRGIGSSYFFRIDEDHVVDATYKGNLARFMNHCCEPNCYAKIIMVDGHQRIVIYSKRDIKKGEEITYDYKFPYEENKIPCLCGAVNCKKFLN*

>CAOG_03801T0 | CAOG_03801 | Capsaspora owczarzaki ATCC 30864 hypothetical protein (645 aa)

MHPFERLLDGEFVHPGVVLTDLHHQGKGMALVTGSSASDGGAAAVVEDCRSEHPLHALFHSTRPEQPTASPVTLAFELLPDQATAATTNKQPCLRKGTILMRTVGVAPVGVYFDVKSTLSSYGADASDTADAMDDGDDDDNDDGSATSDTQDKYLLPGLVEYLVPLIQSRIGTRQFKLQSRTDADADDELSDSPDDIEHEQPDEMVDAEPQDSAESLENDFLFVPGSVLESRIAQLFPRTALDVPSALLDKLNEASQHIPMILETQHRLDLPSDADSQLCAAGSSSSSSSSSSSSSSSTAVPSNSTKQQPLFDENGLILSTLARIQLNSFDVGFFPLAAFFNHGCRPNCSGFVEITDQGTFFSVVLLEDVYLGDELVLSYLPPELLYKPREQRRAHLLQNFHFECGCSRCLADEPQLQRARVCPSCTITSRGLAVQCSLCADQQPAVDSILEEVESVLHDFRDVVPPSPDDLLTAEELAEQCDPLLALWQQIGSTVNMPTHWLVHELLAICSNYLWPTCLALARELVETDVDASSAQATRLRQLLALNLIACRIVSTNFARVLCAHDYLISNWVERMHETYVALSHWINLNDATHTDIDLSAYAFVDLAHAVIPATSTDVDRLAKLSMQCLQEYRRVSQWRTPV*

>CAOG_03906T0 | CAOG_03906 | Capsaspora owczarzaki ATCC 30864 hypothetical protein (891 aa)

MMMDVEPPSSSTSTSTSASTAVAPSTSTPMSTEPPSTSDASDASTNTSSSSASEPASEPMQIADATTAAAGAAASTAAGQSGSSAAAAAAAVQSGTKVQWGLKTHQNKAGDTFAVGHEPRDKPFQVITHSEYINLSAKKDPKFKFECECSFDPATDDPATACGKNCLNRMLMAECSPKRCPCGTYCTNQRFQNRQYPAMEVFRTEKKGNGLMVLEDLAPGQFLIEYVGDVVHNREFKKRTKSYHERQYDHFYFMTLSSDEVIDATVRGSISRFINHSCEPNCETQKWVVDRRIRVGIFAKKAIKAGTEITFDYKFERFSDEGQACYCGAPSCKGIIGGKKQSKDSDAAAQAQAADEEEEDEDDSDDAAMDTSSASTPSRKKPSKAKRKEFNSFLDDLNETLDVEGGLAKELDFSRLMLQVNEDEKRLHMLRALQKTKDKSILASFVAHFGLQQLRLWLVDIHATSTEPLLELLRTLQCLPLKTRNPIDNAKISKHVEPLLQHEQEEVRTAAESLLAEWQKLPVEYKIPKRKLPSPPATPSGPSGGSSHATTPAPLTPGTVPPLLPASSMDRRPRSTPQPPVREREREWSGARDDRGTSSDRDRDWSRDRERDRDYQDRDRDRDRDRDRDRERDRDRDRDYRDRDRDRDYRDRDRDRDYRGDRDTFDNRRNSKRDYSQVDGGADRDSKKAATDSSSQAQLGAMPFAMPQGMDAYATQLAQAYGMDVAQVQSQLQAQMQQMMMYAAYSAMQGGMPATAEATSSASAQPADAPASTRPSESNGQQDDDADSDQETGDGSHRVSKTREFKKKLAPIITQALSPYFKPACTQGRITSKEDFKHLAGKLVKLIIEKERKKLTDNEASLVLDEAVTKRAKLFVTKYMSAFDAEYTRV*

>CAOG_03954T0 | CAOG_03954 | Capsaspora owczarzaki ATCC 30864 hypothetical protein (557 aa)

MSLISPFHPPQRITPRAAKLLDRHSLALAYTPALGRHITAKRDFRAGELVLASKPYAAVADTDGPAAGRCSECFQAQDEDADVAAAAEMKRCAQCRRAQYCSVECQRAAWHGGHKAECAAWVRGLQPYTKDGVLDDDPVAINEVNLAARIIDARMSQVAGSSRTLPPPPPPSQDDLESPTFEDVALMLSNALPLARANAKRYASNAELAALLATRYSGGFGPDFDKLTPELLGEEGRLFSTRPEQFMLHLLCVMQCNNFAIHNDILFARGSGIYPVAALVNHACVANCVLTYDLKSKRQFIRAIRDIRAGEEITHAFTDAASPTVVRKAHLKSLYAFDCNCSRCNDSDAAKELDAELVATRPIDTIPAYFKRFRLERLAGLPSLLERATRDVPKVSSPADVEALLQASRSWLQEGMDPRLPTARAIVLLVTAWAVRQALLGDYNLELFESNVKIFGMALLWREEALSERQRDLALVSGLRNVLVDSARHVIRVYQRIYPANHPLLGLQWFSLGDIYSSDNDGARAKDAHARALAILTITHGPTNPLVNSLRRLVHE*

>CAOG_04094T0 | CAOG_04094 | Capsaspora owczarzaki ATCC 30864 hypothetical protein (974 aa)

MDQAASRRAASLLAWARQNGAYVSPRLTMFTDQGGAGAGVVAAAEIAQAELLIAVPLETLAVQGDTALLCHGAGAVVQTQSSESPLVSFVTVSEKGAGGYCAATARLEHLPHNSGNGNGAPPSVGGSPPVADQGGNELQPAGPTTQLARRPSRNASAASGRYSSSSSSSSHSNADSSSSSSRRPAASAVAIAPAASKKDPKFGFGALPAFLPQPAENLVGTPSIVSSTPPVWPPAYSARTRDAEAALAKDAASFNTQRGNNSQGVPPTSSERLTTFLAEHQPPLAPWLRLALVTMHELGLGASSWYHAYFATLPSEFTTPSYWNDEQWELARGTTVHHLYRRNMIEQQYEDYVRPLFAECASNPKLGALLDPAIHTLEVFRRASSIISSRAFNLQLNERGLPQTLPRPVLVPLADLLNHSTRDVNVVVDLVIGGPPVELASTTTTTTTSSTDADTTVAPPLGADAATATTTAAATTAAAGDAASAPFNPRASTYLALRALRPIAAGQEVLNNYGSLDNGDLVQRYGFVERHAAHRRVFIELSLVMTVGERLHLRCGSRRYLFDDKKKLLKHNNCLEDAYDLCDPGHGRKLLSAVLTVCLMNDRTFTALVNNVGYDAGQESILVLGRSRFPSNPPQVMAAMAFLCEAKLQSYTSSLVEDLEALEQPALDPVTRVVRQLCAEEKRVVAKLGVAIRRHIVDMLAKPCGEGSYGFASDDPTNGDSDESERLASRAHQRKVQYHHVRKLQAARRERRSTRQQIKSKHHPHAPDGQQVAVNGHGQEDDEDEDDDDDEDDDETNMTAISDVDPAAAHPRRASQAGYQSSGAKLARSPNSLTRQPSTLERAHSSSSSGVPAGPSSGSEAPFSDDAQRRLDKWLSDSEGSEYRYDDDGAASATDDDDDDDDESDYDEDDEYDFYEDDEDDDVVEVSSVVAPITIASDDSDPNPSAPIAATPAIKRQASEPAQALVTKRARTS*

>CAOG_04372T0 | CAOG_04372 | Capsaspora owczarzaki ATCC 30864 histone H3-K4 methyltransferase (535 aa)

MKMVEFAQSSVAYSRMERNNKRRRREAEASDAEHDERTTVNEFNNDDGDVNIEDENERDRVKRVRLMAASVAPDSWKAAAMLPDSSDDDEDDENDHDDKDDENENDESDAEEQDQDQDDDEDQSESESAADQEDHTELKADGTHVVHYKNGNTFTGTIRDGLKQGPGVMQYHDGVVLTGTYVDDQLEGASRYDYPDKTYVLCTHHRSRLTGLATEYDDQGRRLQLRHHNNSNDNSDDNDDDDDNDDVEILATTMYYVDGGRLDLGPNVTNNDEEAQSPDTSATAATEGVCGGEYDFPNSVVTLRGDWRRTTLISARAYDNNNRPFNPVAARREITCAAPSTCFEPAPESDTLSAKFTRTFATDAHICDAPLLPDPHESILVYVAPSTVPGAGEGLFARVDIAPRTVVSFYHGVRVSHAEVDARDWSANSNTISLDDTVVIDVPVPFDNVNSYCATLGHKANHHFQLKNAEYGLFTHPRFGLIKCVQALDRAIPAGSEIFVSYDYKPDQTTGDEPQAPQWYLDQYHEHMKIQAGR*

>CAOG_04380T0 | CAOG_04380 | Capsaspora owczarzaki ATCC 30864 hypothetical protein (501 aa)

MTRLTMKWASVAVLVLTVIAAAGMARAAETTTTTTNPGLGLGRKQSELPAFREFLARGGAEMHGVDIQEHVDADGRPGVAGRGVFALRDLAAGETVLRVPLSLLLNVEHASASPLGGILDDFRLSDAEAMAFWLIYELTRPERASPWLPYLESLPASIKQLTMFYDPFEMKRLQASPVAEFTSRRTVKMRNKFGKYREQISKHRPAHLAEIEFPVELITVDDFLWAMAVQFTRLITVQVKHPADGEWERTKCLVPLADLLNTAPADQINVECATNLDSTHFECATIRPVAEGQELLTPYGGAEQLSNGQLIMDYGVTFRNNPSDLVALPIPKLRETAVAYDSKMRLLMAMSLDRFDRLQLPVLDHFESIPKELLAFARVYVSTPSDLSDLEHVLELMKEHRAINPSNERRALELLLQLTNEMILKYITTIEEDETMLRELDAESVPNANAVNAVVLRLGEKRILSSLWQLLDSAIEALPESKQALEDVEDDQEQVVHAEL*

>CAOG_05002T0 | CAOG_05002 | Capsaspora owczarzaki ATCC 30864 hypothetical protein (641 aa)

MHCCSSILGLGRSRSRVLLLLAVALLAVILALQTTVANAADSGSGSDSGNAAADESMLLHSDAGDRPAIDGDGSDRGRNAAASNQCSDIADSQPTTVAQPAAAPPQRRQQAKQRVVLDSQNKDAGRRDRAQPQPQSQPRRRSANHAGSPPPVLSPAAAQLAAVSTPRGALARLTAWIDNAGLEINSNARPGLNDVDELYLFASNPIEAATLVATVPAPLVMFETYLRTLENPMILAIDRRFKTMSVPDPSYALAMALLYESYEPKSMWREWISSLPQTLDSTVFWSAEEQDALQSLPLKRKTQILERHLQQLYNATTPRLLAAFPHIFAGGNYSYEMFKWAYMIVDSRSLTFSTGPDTLPQIMLAPLVDLLHHDPVQTNIQLGVHPEEVLGFEISLKTTRAIKKGEPLVRHIGELPNHQLLLRFGLAMPRNPYEFYPILLGSSVLRALTRSNERVRVLSHAKLNVTTSEFQFYLEHPIIDPDLLFTLRVLFADSHELDLIKSSALIAEKVVSYTNERRVIAKIEELAVQAINLCDTTWETDAKELYSIKADVKSRKWKALVYRVSQKAILDAVLRWADEQKAAMAVLANEHLQQVLEQAAKQRAQQQQQQQQQQQQRSHSRSSTSEFGIRSDTIYARDDL*

>CAOG_05107T0 | CAOG_05107 | Capsaspora owczarzaki ATCC 30864 hypothetical protein (939 aa)

MHCSRLGLAVLVLIAAASGVAVGSEIELIMDSQEQTSMSRFLTWMRGNGAEFGHVDVSQDWHQGRRLIADNPLKPDDRIAAIPTLLTISLDTALQVGLPRAFTTIWHESGSQDDLLALFLLREKALGARSAWAPYIEILPKKLSNLLFFNDGELAQLQNEQLVEQVSQQKSELQGRFLALRQHEADIFGGKAELVLSDFLWARAIVLSRAFTIHARRYLIPFADLLNHRFHPTRGLDESGEFFYRHHDFQNGMFLLTCDRPVNENEEVEDDYGNLSNAQFLQLYGFVPESNPHECVEINLADLLHGEREALLLKSEYAFKLGIPHIVCIGATRPPSVTGALEAIAYINDLHALKLRACIDEFSPDRSPVESFSNCVQGRELNMADTVERIIVKLTSIAASFATTVAADELALQRTEDKPRLLHRRLALQYRIQRKRLVAELISTFANSFEPTAPAPSPASETVSSSVASSSSTSLPSSTPAPAPSPPSSSPSIPSVPLPSSSPAAAAATTPAPARLETSQPTAPVSSSKLQLFQAWLHEHKCRSKKIQLVDRSDEEDGQFPLAVIARSPLAEFETIARIPLGILINDETAKASLDVGELLTALDATSQQDLQAGKTADWDGSETNRLSLVLMHEYFVKRQESDYWPFLSTLPALSELNTLLSITREELGLFRQTPIFADMVQLISNIDRRFVDLSSLLGRVEKKSLFALNNTDGTTSSAWTRNNFMWANIMITTRAVWWNGRLHLMPLVDLMQRYENVDGRVGHFGLELSDDGGAVEVTSTEAIEGNGRAITASPSLANHQYLYYHGWTREPSPNDCIKLLLDVSVDPTSPRGGSSLSSADKKAKIDQLIRFGVPNGIKVFCLGLAPAIHVPASATSLKSLSTPTIDALEGYISTLEPALHFVRIQYSLGSHPRSVAVLSKFRDVVEAQLQIYRQMDL*

>CAOG_05217T0 | CAOG_05217 | Capsaspora owczarzaki ATCC 30864 hypothetical protein (265 aa)

MSSTTTTTTTTTADSSAPTKHKLCAVCQKLPTEVKRCGKCFKTYYCSRECQVKDWPRHKTECNSAIHINTSATGSASGSNSGEQAEETKKLQQTAAPAQLFTPQAVTGADIYTLFNLPVGDPKILQFVIYLVHLNKTNGAPITGQEQYHAPVPTIARFPGSDYHNYPVLGLSLCFEKNLLTAVHVYCEAVSGYTGYCGPLPHGISLKDNNVDIVKRLGEPTVKGGKVVNVWIAYEELGMQVDFSTKDWNDLDNKISSVALYAPR*

>CAOG_05634T0 | CAOG_05634 | Capsaspora owczarzaki ATCC 30864 hypothetical protein (951 aa)

MSRFVEVERQISAVLEASRSNAKHDNNESNDDDDDACQTAMASMVAAASALLGAKRNAISTARAAASTASAASAGISSTWNADLLAALAEDDELAFLEHVEAQQGQVAVRALRVCAWRARQTGPFKQDVDAHLALQIEASERRWSELEQPAVARVPAICAVAMQRQVYAQLVNTAREHLGLSASTLPSDREWLATLDNVGLLDDVPDVAATAQLGTLQPWLDTRIDRTLAWMDLNTEKLFVEDLPCVQWSIPDAVRVTLPRILTCHGSDALVAEQRLQLLMTGPCPRSFLYSKNADHTSSMTPVVLAILESLRLMRMTADEVYFENQLEHCRTTLTLQGGTFDGCAVSILNLSNERQAVTSLVQLCNTMMAATTAPASATFVQVPDHVAKYIELVHSTYEHLRNRAYEYWTAVIGTRPAALESRKIGDIVDKPHTDLLQWLHNAGMTSIAENHLSIADFEHTGRGVLANERIEAGVEVLHLPQHLLINIHVALDESHPIGRVLSDLRDEYDDDTLLLLYVLHEKLVAGSASRWAPFFETLPATYNSPLLFHVTELLELEGTRLIDETFEIKDGLRVLHESLGPLAEAYPALFPTDAFTYENLLWVRAMIDSRAMKLPVPAAAAAVAAAAPEDATETPFVANLIPFVDMINHEEHSHISVRRYDTSAKALVLTTLGACAAGTQLSLHYSTLPSWQQLLYYGMLSTELNPLTVTVDVYFTANNPDESSSDADTAPAAVVDDEEGGDDESDTAMDELDADLNDDEAQELYGSYFDQGPIAKWMDEHFLPRTHTLARGMHSSLLLPCVCATVLSAEEFEAVREETLTLLALQSESEHNEAANQLLRRQVALDALSNVSEDVRTRATESIASVLTELMEAFGTTAEEDSELLADSAQTDDSEMLSVNRTLAIRYRRGIKLVIADNLDKANRGDFGASAYTYAEEDGNVCDYGECN*

>CAOG_07304T0 | CAOG_07304 | Capsaspora owczarzaki ATCC 30864 hypothetical protein (214 aa)

MVHKVYGLGLSEKHLKGLRQAWKELQPVILKLNPEALEGVHELPLSEKQIQQIEMRKAKKKGYDLKLSKAQLKLIDDVKEGGSLMSGLTGIILKNTLLQGLKAVAKPLATGAITYAGGEAAKKIFGGAMLEISEKEVEELLELAMSDLLPEGAHTAIKEKIAQTQGRGLYGAQSGNGLYGAQSGNGLYGAQSGNGVYGAQSGGCMACRCEQQM*

>CAOG_07464T0 | CAOG_07464 | Capsaspora owczarzaki ATCC 30864 SMYD5 protein (448 aa)

MSSTAKANGKGKSKSVADSMPAAELVSARPLPLADEYFATLCAGKHVQVRVEDARKGRGLFATQAFKKGDIVFTEAPLVCAQFLWNEAYGYKACHQCMRSLESPGEMAARLATAPTTVVAAGSAVAKPRTPFELPFMDQCNLTIATQQDIVTCETCSLVFCNAACRDAAMESHHRILCTRNDADHPLQLLQSAWKSIHYPPETTSIMLLARIIAMLRQGLDKNSKDAFTAFQQFYRCYADAEGHFIHKFLDKKYDKQLAFIQELFKTALYDDRIPELFTTHGFRSLLALVGMNGQGVGTTALDMYLVAVERLNLSESDAKTRDQFVEKLLDDIDEHSGEFDACEGSALYCLQSCCNHNCQPNAVPTFTENNATLHMRAERDISAGDEICISYLTPEQRHMRRSRRMATLRENYLFMCACAKCEVQKAELDISSESSDDSESESAECC*

>CAOG_07681T0 | CAOG_07681 | Capsaspora owczarzaki ATCC 30864 SET domain-containing protein (378 aa)

MTKTSKKASAVQAKPEPQSGDEENVAASVSVSNTKPSPRSPSKAKARRSKPAVDDEADEDVVDVVEELPARRSPRRSPHNLEKPEPRTSSPAVAKSRQSKAADAEEQAQTQPMPSSPAKSRTARAAAAKPTARSLKGQIDAVAASAAAASTTAPAPATAPATASAPTAATSENSSSNTEKNTQAIAAPSGVPISRQATLPLPPVLTPTSSFERRSMRQCSSEVKRLLLANIQQAIRIGNEDHLEVYQTPTIGFGIKATRDYERSDYVCEYAGDLIDISEAKRRNEEYMKDESIGCYMYFFQHKETRWCVDATHSTRKGRLINHSKTDQNLLTRILTVDDKPRLVFFATKHISRGDALWYDYGERNAATIAAMPWLAQ*

>CAOG_08491T0 | CAOG_08491 | Capsaspora owczarzaki ATCC 30864 hypothetical protein (1445 aa)

MRLDQPRANAGLKRRFSFDDGANGHYSVSSWSATGPAAAAGRAVKLARWLADTKKDDDDEEEADNGGDPALLKNRLASTSAGSESSQREMRTDAAGTGTVAATSTCSSSGSALSTVQAKRHRHQSANGNRQNDDDNDPTEDEDATSDEETETAEDDEDSPDGDDAEEDNEAEPSSSTQQRPQRVRRMPVTLEGSLVMLHPVQRRRRASAPVAPPAAQQSANIRSPAAAPRAPAARFESPGAASRSRTNRYASSSPQNAERTAHIVGRRQPVYANPQDGASPRRSSRVGLPPKRIVLDHSNVNGVYPEVDQVIGQPTRRISVAANSKSKPTLENGNDDSADDSENEATSILDADEEDEESDEGSHVDEDETEGEEEDEEDEEEEDDSAPEMDYAALQALKEQYYVTKGLHWDVSRSVSTLFPLTDCQCVRGNHLLAFELDFQLPSELHRVFRPSEPEPLQLDQSDTPSPRTISKQKSSAASYASFTRIRKNAYIDVKRPKLIADNECNCKASAPCTESCLNRLMFVECVRGSCRMEDRCQNRNFQRHNWTKNLKVFQTPNAGYGLRCTDPIAPGQFVMEYVGEVVSDAERERRMWGPYAGNPNHYFLELEKGVLIDACSKGCDARFINHSCDPNCHVEKWNVNGEFRVGIFASRAIAPNEELSYDYRFETLGEIQQQCWCGAANCRKVIGKATPSEKRAARKAKPRIAIDFYNPSRTAPPSAQVLQAPELEQLQRTKLVLMYPLRRVLSQEEASGQSAARKAAEELLLPVNLSYNAVVAGHEMQQQRDLRLDNLLLKRCPNIAAGACAYPLPTSALARFVVSGTVLDRHQAQLLCRIATDLTAMDGLIQRLQTLASPEDNGVLFQARSAMLAPSEYLGRPAVQRRRPFLTLGGALKCWKKFVIDGVFETYDELESSIRGVLQRSIAFLEQAHESGMHGTMDASGSDDEASKLSPFASAQLVRSASDLIEEELAEIRQEFGRSFVEQFVPVPSTAPSIALPPARKPVIITPPKPSIALASPYEVRSPRTPSAGFAANGTGRSSSSASVPRPAHQDMTPPHSQDTRTAKSPQPLVTSGQDNLAQEDESSGSEVEDELDLEDSDAQQVTAMDDTPDLQVNPFDEDVNRCICGVTFDDGIMLECEKCHFWQHEECMNPLKPDGTRRRIRRDEEHLCHLCNPREELTAEVALAYDPKLRRTYFRSMECSTGRLLRMGDAAYVTEYPKPTNRPEGNRNDATHQKRPASKPVLTICRITEMFVDAKMRRFARGMAYVRPESVDADSPQTVHKQELYLTTRTLLFRIGRVRQRRVVVMDPVSYMLGRCRNMLGRKGLLAMEEDTFVCEFFVDSRSTRDRILPLASFDYPLSTNPLHFRAFPEPVPLVRDTNPSSLLLLISARKPLKTGGRHIDTVYKPCQGKSLSSILDQLMIETTGQVAPPLDLHKPFKHMP*

>CAOG_08575T0 | CAOG_08575 | Capsaspora owczarzaki ATCC 30864 hypothetical protein (534 aa)

MRGSRLQCIVIAAAVCSLTALAAAASAVVGLEYTEANNDLDTGDLTTTDALAQQQSVPPLMQDALDEFLQWASEEGIEVGESDAGAKTLELRLHPTMGLSIFASQAIEASTTTPLLSVPLSTFFARFTLLDSPMMAALAVRPVAREEAKLSLLFLYEYFDPDSFWQPWFQLFPRELDCAGFWDDLLLMELDNTSIRDAIRQLEALIEYEYDQLDLPALRLRFPDSFVADRFSYDDFKWAFMVLASRGLTMSVNNAPCTVMIPFVDFFNHNGAKSIAFSYTRRAGDASDVSSGNYDDSVENLNCAVISGNETFLPGEQMFLNYKAHSNEVLLLHYGFALPHNEHDTFLVRLHFDREKTNDPLMDLREHLLELRGIQENHPFLLRWHGDIIDPDILFALRVMIATKDQLDFLLEEGIASNSPFSVDSELVATVLLQRSLERNQAKFATTIESDEFAEQRVLRRLEETEPESPAMRGLIRELHAIRYRMGQKRLLAFALAQLAQLQAELDLNRITLDATYPFQRFQQSLHEQDSSS*

>CAOG_08663T0 | CAOG_08663 | Capsaspora owczarzaki ATCC 30864 hypothetical protein (501 aa)

MAEDSTVESAIAAAALADSHKKEGNALYHAQDYVGAVLKYQAAVDAGPPREALPMLYQNMSAAKYQLKQYAESIEDATKAIMIDPTFLKAHLRRGKALAALGNWHQARNAYAAAIAVTPNSTEAQIGRIEAEQALVAQYYKPYNETMTNAQVRYINPTRGKGVVTLKELQYGTEVFHEAPVVSHRFVGAEENSAIPACSHCLQTRLTPDMMGPFAVLHSEVYPSGTPSFLSCEKCFVPYEQYCSAKCRTQAWDDYHSVFCAKDRELAKIHPITELYALCRKHNRTNPLIIARAFAMSLTGVTSGRFSNMQDTFQRFGNFIASEEQTPHDAEEFALILNAFSPPHRKLMEIVLTIQNFRMLDGAIMRNAQRLNPVSDLHAMIDRLAQIDAHKLAAVLGKIGFKIPQLPGLRMSTPMRSLTVSGSGLFEIGNTMNHSCQPNVVSMTRATDFTLSVVAVATIPVNTEVCISYIDTDLPKAKRQAALEELYYFSCSCAKCQSED*

>CAOG_09018T0 | CAOG_09018 | Capsaspora owczarzaki ATCC 30864 hypothetical protein (351 aa)

MRPQGKTAENKQLQLVHRRQAVLNLRAQKVPVKQIATQTGLSLRGIYHIIDRYPDGIAAADARRPGRPNVLTDRDSRQLRHLVSSTPAISAKTAASILPARNGRHPGTSTIKRALHEMEFVNTLRALKPRLNKKNAAERLAWCTDHRSFTVADWRKVVFSSEAHMRLHASTPRGRVWRKKGAPLTRDMVKETVQHGGTEIYLWGAISSRGPLPLVPLDEALTGKQYAQMLDKNLLPGMNRLKMGGTFIFMQDNAAVHTSKPAIDYLEDADIKVLNWPPYSPDLNPIENLWAEIQKGVLSEFHPQNKQQLWEAIEKWTEKHVTPQYCRALIDSMPKRIEECLSAKGWYTKY*

>CAOG_09047T0 | CAOG_09047 | Capsaspora owczarzaki ATCC 30864 hypothetical protein (351 aa)

MRTPTGTGTPKQIERRERRQAVLDLLRQGATHPEIAAQSGLSLRSIRRIIQQCPTGVAREDAPRSGRPKLLDDRDRRRLARLVSSTPGITSGAAAAALTLKDGRHPSSSTVQNELHAMKYVNALRVTKLKLTRKDRQKRLKFCRDHASWTVAQWRTVIFSDEAFIKIHSSSHRGRVWRKRGERLAPGMVKETLQQGGSRIHIWSAVSRRGCFPLIPIEGNLGAHQYAKLLDTHFWKGLDKLGIGSNFIYQQDNAPARTSKVVEEWLELHQVNVLPWPPYSPDLNPIENMWSFIQRGVLAQYHPSNQAELWRDMQKWSEEHVTPAYCRKLIDSMPTRIQACIKAKGGHIDY*

***Dictyostelium discoidum*  (Amoeobozoa)**

>DDB0167139 pep:novel chromosome:dictybase.01:2:5934525:5936105:1 gene:DDB_G0275621 transcript:DDB0167139 description:"Predicted by Dictyostelium Genome Consortium."

MNNQVKPTLSNKAEIINKWLIDNGVRINNKLIKIVYLGKENNFEQTENTTATTTTSERINDSIVSGLGVISLKELKVDDIVAKIPKSIILSIHTSSISNILEKYKIENNIGTSIALIHEASLGEKSKWYGYISSLPRKVDVPILWDSESRKLLKGTAIEDVLNDDDILINQVYADVIESILSKNHPEIFGDKELYSIENFKIANSIISSRAFCVDSYHGDSLVPLADIFNHQTAREHVHIESNGDVCNKCGSIKTCKHRKVVTQHHTVNSTKGKRTHKVAGIPSSKKHIHKGNCCSTTTTKTNEEDKDTIIEEDDEHLYIKVVKGVEANKEVYNTYGDHDNAILLSKYGFLEMDNPCDRLSIDKQLVDKELELIGAENQITRMDLFNRISFYAEMFDIDSRNNHAIENDGRLDDALVCSVGIALAPQSIFQSWKSMSEHKREKYFEQLEAEDIVKQSQLVRNTLLSILNKLFSNYNNSNETTATTTIEQDQEKLSTLKNQREIIAQSLKICEKTLINKSINYYKNL

>DDB0186119 pep:novel chromosome:dictybase.01:4:2287653:2289871:-1 gene:DDB_G0284649 transcript:DDB0186119 description:"Predicted by Dictyostelium Genome Consortium."

MSTSPTIKKIERINYINKLITELETINKQNDLIMAQVPNQIQDSYNIITRFMEHVEDYKPQNLKNLSKSNIKIMELEKSINQLKTKIKDTQMRTNNVTSQKDLERELQFKLNQLEELRKKSLVETNNTVINNNKTTSVPSSSSSSSSSSSSSSSSSSLSSLITTTTPVSITSSSLSSAQIIVNNNLTNNDKLKAMKSDLKNLTVSIQKIYQCTTIFYQSFKSYFNTLGEVLNRIPTMRGGLDIGGVLQIGGDESTEILERMCESIYQSSKLAWTFLSELACLEKSWYNKNFKIYQSILEKTEKDSSFDDKSPSSSSSSSSSSSSSNNNDKKYGILNVLKRLKKKKTYRGSINLLEKITIMESESEIQQKLNNSPPMYSKQQQQQAKIGSTGLNNNDKKVTIVYEPINSSTTLLDAIILDLDKLLSFSDREITSHIDSLEKKSQMLIDILNLLKMRYQYFREKISRWVDNNSAILRVTSFEADPESFKIMEFKNIPDVQFKVKILYKVMLSYFTCCLVATPMVYLTPESWKLSNHSDFRAEKRKQIEKLLLVLKSNVAPIDLDVLRKLILEFTDILICALDIQIPLLIEVYSDDSSTAKRVNYKNEKKLLKETITKSINQLKYFQPQQSSLSSSFGISTSNNNNPVFQELLGNSVQMIQSVMLHFFKMKPIGRPHVYAKDGNWSLVSESKRRYEYRTSILI

>DDB0187856 pep:known chromosome:dictybase.01:5:1310716:1312160:-1 gene:DDB_G0288263 transcript:DDB0187856 description:"Predicted by Dictyostelium Genome Consortium."

MNNYWKKRIEKDFNINENEINDKDISFENQYIEKLGQDKEASKEFSDIYMMAAKLVFEDQLTSLTEIILEKDLGSIKYQLSSELRRNPNFFKDLLNQQQQIKNEEELESPSLNRMSLFFSIGTNDIEIFKYIFEQGGYNLVEFRDPVTGITIFHLAMMLGNKEILQYIIESPKIKDLKNFTFFDTIDSFRATGFDYAKLKGLLPSNTPPTPKSIKTYNNNNSSSFEDLSIEQLQLKLNIIYTSKVLSTNDYLVDLLFSSLQINPDLNFRNKYLNLINNSGGEENIILGFISESVGWGLFAGKDFKSGDFIVRYGGMITMNEKMETTNYNMMISNEDFGLDASKYRSMGGMINHSSKFKNAESECIFEYGCEQALITATKSIKKGEQIFIDYSKSFWGENQNDSNEMIELGGKNDYPSLIKF

>DDB0191793 pep:known chromosome:dictybase.01:6:2535539:2537077:1 gene:DDB_G0293118 transcript:DDB0191793 description:"Predicted by Dictyostelium Genome Consortium."

MNLQFSIPGFVSRRYINETKGRGLFAEKSFEKGELIFSERPLFSVQHVYNRATAWTCGNCFKFLGSLNKQMLHYRKVFGIKEASELPEFENLFNFHTKVYSCFAKCGEKYCSEECRNVAFYSHHQILCVGEQTPDSNPMYQFKKHSIETNELFLLAAQAIACLICRIANDPSSDQPTQIKIPLTTNNNNNNNNNTTTTTTTTTTTTTTTTTTTGNSSQDTIIQAPLVERICNEFFSEYQHREWWDVRIDGNFTLEEAKVWSNESLELLRKALIPLLQNNPKTNNQVFIGTVLSMEFYSHLLGLLEMNDNSIGFFNPLELFRRDINAIGKGPFTTPPIQKNLKDRSNAVLASTVDAFRQYHNEQDDQCDQEDCNDHEHSHGDSMDDDDHHGHGHSHGHGHGGSHGHGGSHDDHAMEDNQEEEEDDVVEEVFPSFDGFGIFGLQAMVNHSCEPNITVVFENHDNRAHIKATRRIEAGEELFHTYIEETNPYDVRQEDLITYGLKWECGKCLEKI

>DDB0202183 pep:known chromosome:dictybase.01:1:1104198:1106228:-1 gene:DDB_G0268386 transcript:DDB0202183 description:"Predicted by Dictyostelium Genome Consortium."

MDINSNHIKKINDFKDKGNKEFNKEKNKDKLNKRAITYFQLGIKYFNDEKHKIEINDQINKLISILYSNLSLMYLKGDEIILSLEASNKSIEFNNQNSKAFYYQSICKSYLCDYKGSMESIDKAISISKQDEKVVQQRNRLIEITNNKVKEDVYFENVLKNQKLENVAYENSIEKGRYLISKRLIKRGEEILRIAPFSFSLNGKYVSKFCGVCYQKKSIDNLLKCKDCDDFAICKECKAPNEPLSKYHPNDLCVLYKLNNEYYKMNNFEIANEDNKERGLTDERRLYINTIYNIFKNKTNNKAVVEVDNAISSSVIDVLNLSKKLLNHTNESQRAYSDSTKMKTDILELIFKSFYGANTLKKDEISNIIRIVDCNSHGYSDVISQKKKASGLYPFSCYTNHSCIPNSDYFIDKHGVMVLYASSDIQESEAITISYIYFLNRVEKRRKDLLNGHNFFCTCDQCSFQSSLTEEVCDKCNEIIPSTIGNSNLVYKEPKSLKETGFNYICPKGHTKSSLVYDIDNDQLYPTIAENYSKCKDLRNFRKINIFKEMLENRDFVKKLTPREFEVTPLAKYIANPKKSNSMLFEELINLVRGSVECGLTKSIEESNYSTYLVYDYYDLYKSLLLESKPQILKLNSIRDIIIKLLTPMVYNQNKPRIDKKLNKHLSNKINLKKNK

>DDB0206180 pep:novel chromosome:dictybase.01:3:1143468:1147597:-1 gene:DDB_G0278771 transcript:DDB0206180 description:"Predicted by Dictyostelium Genome Consortium."

MEQQQQQQHMIYNSQYDNNNNNGLSPQVYAMPDDINNYHQNHQLHQQQIHHQQMMMQQQQQQQQQQHQQSHYQQQYQQPYSPQQQQQPYSPQQPYSPQQQQQQPYSPQQQQQPYSPQQPYSPHQQQPFSPSQQPFSPPPTHTHIQQHPFSPQQHHHQQHHIQDVNVNEYLASPNISYNNNNINCCGDSNSHPMSPSQHHTNMPTVMYYNSNSNNNNNNNNNNNNNNNNNNNNNNNNNNNNNNNNNNNNNNNNNNSNSNSNSNNSGNNNNNCNNSNGNIQYQQQQQQQQQQQQQQQQQQQQQQQYYQINPRLTPPPQQPRSPIQQQQFIPITHSSSPSQQIPHISNSPPSPYQITSYNNNNNNNNNNNNNNNNNNNNNNNNNNNNNNNNNNNNNNNNNNNNNNSNSNNNSSMGNISEELNLIYANKSSTPTNMPVVINNLGNNNNNNNNNIGTQSISSSSTPLIQSRKRSICEPPSNDLLINVSMQNTSSTTSSVTIPSPLSSFEIPFEKQIESQVLSSFESFNNSNNNNNNNNNNNNNNNNNNNNNNNSNNTNTNNNNNSLRKDILLQISDTQEVLYRSLLKENIPKMNSGQLDQTKFLYDLYFMLEKCCNHPNLLNNQNNSIANSSSKFLATNYLIQNIIKQHQQQQQQRQVIILLSSSIQLFTLLEEFLKLKSLNYFKINDLLDSATSRNITSSPVIILSSFVEFINFDTKILTNLGGGNEWSSSSSSSSSSSSSSPSSSPTTSSTPPSSPPPIPTNLNSINQFNVIEFEYYWNLDLSKEININNSITLKLKIESLLSLSSSSSSSSPSTIRKFRLLTAGTVEEWLFIRNQKIDFNCISNTNSSNLMIESAEKTLFEKSKKDEMVEMMKFCIVKMSNYKKNILQTSTSSSSSSSTTTTTAAGGGAISPTTSSGSSSPLLFSQFQTKANLIQSMSILNSKSTSFDGSTNLLGNNNNNNSNNNLWKTSSLNGVKSFSLTSGGSDSSNPSPLSSSPSGSDPISMLKTAEDLKKKNRSPKKHKTLLDRNCFSCKKEEDSKGRTSMVQCRSCPKIYHRSCAGLAHTPRSWKCSRHACHQCKKTPNESGGSFFICKGCPSSFCITCLPNDVKILDKTEYAEFRHNQLPPPPPLPPQPQPSTTTTTTTTTTTENHLLLNPNNSHLVDDVTHRGSGSCLTISSSKPNGPTPISASSSSSSPALLETSHLLLQQQHQFPVKKQKIQRPTVFILCGVCVKLEQQNPGSTSSPHTLNTTSQHQITTTTNTTTTTTTTTNNSPNNNNNSSLSTNSTPNQIQGSNTTPTLEKLSSLNLALSPIQNTSPLDSAPSSNESPCAQSLPNTPSFYLSDTTSEYIN

>DDB0206224 pep:known chromosome:dictybase.01:3:1258942:1260744:-1 gene:DDB_G0278829 transcript:DDB0206224 description:"Predicted by Dictyostelium Genome Consortium."

MITDKCKLVNNNDERGNFVVATNEIKVGELLFPEESFASIPLEFHMNKDYCFLCCKQSEVEQVQQPSQQPQQTNNKSIQCKFGCNMWFCSEICSNDMTHQLECSFINKLIESSIKNECDVSTCLLALRIMIRNKIESDKYQETVGKLSNQLEKFIEINKSFIEKYDINFQQFINQLQQENSDPSLLAIFNKDEFLQIISSIYINSFSGLSNDFNRKPISNGYFYKPALLNHSCEPNIFFTIKDKNLEMRACKKIEKDEEIVDSYVDLLLPTIERQKILYNSKNFLCKCSRCSDSTEDERYLSSIYCFHCNDGVEFISPEIYFNKDTKKLQENWKCSNTSNTCEFNKKKQNKRNKDPELVLILAGINEFKEFINSEQLLFNDVNLSTADQEMPIIENILKRLLKFERDVLSRLNLNHQCWLIYHIKLSKTFEKLSLVYNNFDYLVQSIQHYKELTRHVESIFKSSTSGELVDCYYHLGRINEKYLLSLIKQRNQFNQLKLQNQNKQDLDIDKLNSDIKNLLKFINQCYTNSYNHSVLVYGNNHLKSIQLNEMILKSNLKSTTTN

>DDB0214933 pep:known chromosome:dictybase.01:1:3361680:3363770:-1 gene:DDB_G0270830 transcript:DDB0214933 description:"BOP Fragment "

MSSIPDHNNNNNNNNKNNNEILNFKQLFKKIEEFKQLGNQEFSKYDKTNDNLNKRAVTYYSLGIECFNDNFEKLKNNNNNNKDDEIELKKLVSIIYSNLSLCFINGYEIDKALEFANKGTEIDKDNSKCHYRKSLCYQYMCNFKESFKSINDALNSINNNNNNGKVIIDKSLKQIIENERKEIEKKLKKLEKEEKESQDRIKEDFEQFQQFPIELVWRDGFGRCVLASEDLPKGTMVLRVSPFASVLEDHKIEKNCGFCFKKINKSIRINQTCKNCKNHLLCPQCSVDEYSLNYHKDECDILNFLKQYYPSSQTRDFRFMFRVLLNVIKDKKNKSFSKENQSKQWLNHQNPFIFDSYKYLINLSRTLDKVQPEQMEAFKRSAQSVIAIFNKLRGPKFFDECGVTIDEIIEIYSIVLSNGHEMLHPLNCHTYGLGIFPTGSYLNHSCLPNAFWYNDDQGMMVFRTLRPIKKGEEILTSYTDITTECSERRKHLLKQYFFFCQCQQCKFQSKLTDQSCLSCKNQLSSSNTSFKIPSNDSIESGFYYHCLKCNSDNKQINCPDDSSSVFTSQHDQRTFKKINLLINIMQQRHQSIATNLLNTNNEYLMKGMPDHIVYKEITNALEQSMISNPSKVSADQVFTNSLIQYYFRLFQTLKIEKPNDTQQLQSVKTKILQLLTPIIHLPTSHHKSSIEKKLKF

>DDB0215922 pep:known chromosome:dictybase.01:5:667105:668784:-1 gene:DDB_G0287857 transcript:DDB0215922 description:"Predicted by Dictyostelium Genome Consortium."

MLEWGINNGIEWNEKLSVHDFEDIGRGVIANHEIKQDEVLISIPEKFLIHSKSKFSLEKLNPPIIKKIKSYIKTFVENNLSPSSIFYKPFHDSVNQFNSKQRISFHLIIEKLLKKNSIWYNYLNDLPTEYNITSTYDDDEIEHLGYPIYVEKVLELKNEMLESFDSFKEILMDNYKNDLNRIVIKLNDNSNDDDDDGGGGGGGGGGGGGGGGDDENITIKLKEIIDFNLYQWCWGTIQSRTYYYDRNMKELPKHLQLEDKDDCALVPLADLFNHSSDVNTETKFDEKKQCYQVITKTKFEKDSQVFISYGKHSNFTLMNYYGFIIENNSNDSIPLVQEDAIPDIILEKEMKQDLKSYERKMSILEQYGLSVYGENSKFLVSMDKELPFSWNYLSILKVLYMTKEELNNQLELNLFHYDLPISNSNYEKVLYFLKNLSIIQLTQFKNYLKFYNKNFNYNNNNNNNHNNDDDDGIISGDISGGGSGDGNIFNNNIYNGIGKRFEISYILEYNIKVWEGCTEWVDQQLHFSLNQR

>DDB0216408 pep:novel chromosome:dictybase.01:3:6179495:6183789:-1 gene:DDB_G0282711 transcript:DDB0216408 description:" contains a plant homeodomain (PHD) finger, a C4HC3 zinc-finger-like motif found in nuclear proteins thought to be involved in chromatin-mediated transcriptional regulation"

MKNCIVCSKVGQYPLNLVKCYECKSYYHKFCLQSTKIPRGEWYCSSCEIERKNKKNKKRKLDGAGKKNKKNNKDDDDDNDDDDDEGENDEDDEDHQDEDSGEEEDEEEDEEEEDDDDEEEEEGNEKNKIKKRKRIPINQSPSIKKRTKDIDSEEEEEEEEEEEENDSEEERRIQKRKNKIKQQQQLKMKEKKQREKLRKRRRRTRSSRKDDNDQDDDDDDDDNNSNNSDDNDQDENKNKQKRKRRKDKKKDDDDDDDQEEEEEEEEEIVTGKRKRRSLNKVQPNQHLNNENEEDNEEEEEEEEEEEEEEETDSSEEFLENLVCSSCNTGKDEDKILLCDTDNCSRGYHMYCLRYPITSVPKGDWICDFCRFGDISADLHSADDDEDGAINSQKEIEKQKQKQKEKEKEREREREREKEKEKERERERERREKEKKDALRSSFSNIPIPKINNINGSNGSNSNNNNNSNNNGSGVNRQSPPKVISKKSPQQSPKPLKSPTSSSSSTTSSPPPLHQQSKVTSKISPNSQQSIPTINSNINKSPPSQSKNSPPLKPLTPDLKKQIPITNITTSSTTTTATTTTTTTTTANTSNVATTTTLLKPSSQKKPTILSDKKQLNTSGTNLPMNGKKKEDLESSASHSILDMIPPNNKLFNNSIQSAPISWDLSGSQHKLSTSSSSLPNSNNNNNNNNNNNNNNNNNNNNNNNNNNNNNNNIKLQQQLQQQQQQLHQQLQQQQLQQQQQQLHQQLQQQKLKQKQIEEEQEKEREQKRLENEQKLLLQQQQQQQQQQQQQQQQQQQQQQQQQQQQQQEFIDQKELEDKLLSQFDNQNLKSPLSLTSTTSSTLSHLPQSTFESSTILSNKTIKQKWKSRILFETDQICQCEIIQIYGMDFKVNPFQNNITSKTLELSFNQVSFDNLPKPIRDEFITKSSTPSTCLDIGAIDLPNSALLIFLPEIPNQSSLYHLIIHFIENRVAGFIDLSNDLSLYIYPYQTSIISLDLIGILIDKKSLSPPSLILNQQQKQQKQQQQEQEQQNQSFDIKNICYIGINHISMNKILTSQGKNYFSFSNFNELTLSSDIKNFDLVVVDNKMVNNQTINKEINNLMQLQPTTNNNNNNNDINNNHDTLLLVIKDVEFSKWLVSQKKFPNSLEIQKIFQQQILFKQSRCIFLLDNSIIIENESSYFVEFISSFGKFKNNNNNNNNNNNNNNNNNNNNNNNNNNNNNNNNNNNNNNNNNNNNNNNNNNNSSNDWTIKISSTIFDELLPFGHQSNVKKKIDEIKRGINQQLIVSYDFSPMVPKQSSDFLLLALRIAHSKKYKQVFLITSNKIIQNQSKRYSNLNLNCLSLEGGIDYIKNINNKFSLKI

>DDB0216955 pep:novel chromosome:dictybase.01:2:1150556:1158821:1 gene:DDB_G0272064 transcript:DDB0216955 description:"Predicted by Dictyostelium Genome Consortium."

MDSIDNMSQEDISSNNSTRTTDTTTTTTTLITTIKDNNNKVEKGIIDKNISIKQQCQEELPFVAENQKQQPLISSIYIKKDGNDEEYEEDGVDKDVSESESSELKPAESESSEESKSEESESELEEEVLEEPEELSSINSTPSPSTSTKMVENENKNLSNSNNNNTNNNNNNTNNNNNNNNNNNNNNNNNNNNNNNNNNNNNSNNGSSSIIISSNGNSNGIVSEEEEESYQLYNFSINVTPKIHLPKNKPITIENLQAKTFQHNRLRYSGKKVKLLSHHYSKIKGGGSINYRNNEQTTFGGSKFFESLFSSRASCSKILRNSIRVERNTLNSDKQWLENNPNLYYKINNNNNNNNNNNNNNNNNNNNNNNNNNNNNNNIIIMSGGEDNDIDKDRLASIKIIEDEDLESSSDEEKSYSKSLRERKTLEELEKGINRQLRDKIDQINLNKQFFESSLGRKKVTLSKQQLEEQQFLETNLFQPNPQFTDNYNKDRPKRRREKVNRIINKDEDNDENYNSKNKKDLENGINNNNNEEEKEESSSDDDEGPYNILYYTIHPLRGLDKRIDSTLTPSGKRKMTLEDQLKETGVETPSFKLVTPYQPYNPDISRKYYSNYKSGTLLTHHSHNPNAQSLSNHHHHHHHNNQNNNSTVNRITSPTSPNSISSIINNSSMSTRRSNNNNSNNNSTNNSPFISPSNNNNKSYNSTPIPLSSPLVSSNSLSNNNGVPVISLWKKVYKILHPTHWTKEEIQIFDEMIYIYGFDWTEISRILCGIKSPIQMYKFYLKQQQQQQLLQISNSENVDNSNNGKNKNNNNNNNNNNNNNNNNNNNNNNNNNNNNSDLQLVTLDETQQILIKNQEKEFFNDWARICYVCFLENCCNKKVSSQKNLEKLRRDSNYESFVNCGTCESVFHLECADPPLHKIPPGTWYCSNECSSLSQLKCENCSKDNKIESMALCISCNRGYHIFCLETPLFEVPYYDWDCPTCETMKSQSADNNLSLLKELPILERLVSNSNNNDIPNSNSLNSSIDLQPFHNNNSNNNNNIINNTTNSCGDLSALNTPNLSISSSLPTIYEDEKEKQPIEQTQNLEKEIKEVVLLKSPVLNNDIISIATPVSSSSTAPTTTTTTSTNSTSQTSPSTPTQISNTIATTTATTSTSTTAQICTSNSALGSPSFFRRSINSPLYNSSSSIPLSLSSNNLLSASTSTSSLNTLLGSSFSSLPQFLGSTILHSEDCQFKNYNNMNNSNNNNNNPNNIDSDEIKSTNETNSEQQNSETNLSNARIIAKEGSKLSSQAVNKQFITLKRLGGVCLGCLVQAGLFEDSPNINTQEVYDYVDTIQQDITEEEFDVESLFTRRLDILLSSILYSTQQMIDQPILSNQQQVEDQQHQHILALEKLKKQLRQERKQQKRQLKKEKQQQRREKEKEKEQQQSQKSNNKSNNNNNNNSKSSSPASALSKNTQQPKQQIIDNIFPLEPASVVVIITPPQKSEQFLIEAPPIELIKLKEEKQEQHHQTEQKQEQHQQKLEESILSLQQELHSNVTTPTILSSQSAIPVDQHRLELPTLPHPLSQPHIPSSPPKQQEQKQQEQPQQEQSQQEQQQQLEQQQEQQQEHQQEHQQQGQQEQQQEQQPSTTSPISSSTTTIIKQEELEVSFTPQTSTVTPIESTTITTTLLEIPTSKITHITETPIMVQSMELATTVIVPQESIVLPIKQELDIPTLQETNIKEIPTTESLVTTEIPLTIEIPITTEINQHNLKGTSTESPTNQLDIVMEQKENSDDKITTENVLTPSKKRTFDQSEETNSIALPSPVSPIAPGFVPLPPPINEEDKLLANKEKIQISQFYDIVGEFIVAPLIEEKPLKRYKRSKKYNTKNNRLLQETQTQLLIMANNSITNGGVNLNGTVDQIAQHSLQFQTHLQLQPSQMDLVQSLQYPPPPPPYVQHPLQLTHSDLSLQQTLSPYSSFTSSNTFTTPTTFPTTIPATIPTQLPTTTIGVPQLPPKPTKATTKTISTPPPISLPIPSFLTMGSSTPLSSTSTSTTPTTTATTATTATTTTTTTTTTTTPIITPTHTPATPPLVNETTPSISLITPSKAKGRVKTKKASLALPQLTPHIPTIPLASMASISSLLPPSLTVSSTSVPIVTINNDSNNNNNNNNNNNNNNNNNNNNNNNNNNNNNNNNNNNNNNEVQTNSKELTTTTNPTSLTGNVIPSPSAIAAATALLMNPKCKTKDAIGVARGRRERMSTGDSINSIRWWIFSGNPKIKPELNSMTPGVEMDWVVRQHSDNIRKNDKVFIWSCGKNAGITATGFTLSDPERVMIENCSEPTIYQSSKPNQHHTIKIRIEKILPVVLPKRYILEQPHLQNMTIIRAPLATNFWLNDNESLVLCELFDSLEKGTLVLPTYENNITGSSSNTSTPPMSPTLTNSKDSKSSNSRRKQDQDDMKMAESSSSSSHSSDKDKSSSSRRRKKLKSEEDLSQPSPSSTLTPLLTNIENHSTSLAIVPTNTNNYEYDEGEEEEEQEEEEDNNDESPDENETKNKKIIKIVESKHRACSECNKMDVKSNMITCDTCCSYYHSKCLLDPIDKSGYAYWICFKCCMSDEEILNCKLVIGWLELKSKVKKLLFKKCDSFSFNLKKSLGNANKKKILSKFSSYSSQFNYYRNQLTKLVNQRYLNWYNGLMIQLNNNNNNNNKDSNIVIIPPQQPIIEEPKVTNTSKTRSRRSTIN

>DDB0218318 pep:known chromosome:dictybase.01:3:4629488:4633507:-1 gene:DDB_G0281543 transcript:DDB0218318 description:"Predicted by Dictyostelium Genome Consortium."

MNSSDDCLVRELEKEGMKLNSKRISLSNSSSLVNTPLDSSPPMQSYFEQHQTNILTTSNNNNNIFIPQISPFILGYAREDMLKDDELAIIPSYLMPLHPFASYQSLNILLQFFKVSTFYPDSLNNNNTTTTTTTINPIVTSLNDLIDHKLLITEQLTKVIIEFINQNRNTFSNLSLSIDDFVMLLFLVSEYYQTSIIEPSLTNTTRSSLKKSSNLNFSSSKVSTLSYFEIIQNLINISTPITNFNEFQNIVLNNNNNDDNNNNNSSTCNYLDLNFEFNKIKLDLDVVFKWIITFIGFKTSENEQSDKIYNKLESCLYRSYNYIRVASIVNVTLPNISFKSFLPLPHYIIQSNIYKQIKTLLNNKTSPISISTDSPTTTTTTTTTTTTSSLLSSPRNHSNLFLSSLENDDESFSELVAVLNTALFKDSPIQLENIEIITISEFNKRIKQNQQTEQQQQSSVLAKTKQNSSNSLLRYLSSNSFDDSQSLIDDYQFDLYSNEELLLFYGYTILDEEEMSSIKFEIQVFKPINQLEQLFTDKDELQREIKLMKVKKSILDNHLGKQQQQQLQKQQQQQQQQQKNEINSSTLIHNSTSSFKKSQTNNNNNNSNSNNNNNNKEGYYLDYLSQENILGINLLELTRLSKLRETEGYFYEDDQLSTMINLRNEMESLVYLSKEFTMILIKITKKLNQISNTLNINNNNNNNFKINNNLLNEIEKYYKFHLSIIERSLIQLSNLKNQFSKPTIKSVPFIKPSDEVYKRFENWLKAGGVQFPKLQIANFTDSTGRGVVTTKKVDENEAVVVVPKKYLINVDVAKAHPILGPIFEELHLNDDTILFLFVIYEKGNANSFWRPFYDTLPSYFTTSIHYSATELLELEGTNLFEETLHTKQQLNSFRDYLFPELSKQYPDIFPESQFSWENFLWARSLLDSRAIQLKIDGSIKSCLVPMADMINHHTNAQISERFFDHDSQSFKMISSCNIPANNQIFLHYGALQNWELALYYGFIIPNNIYDSLHIGYELEEDQEEEDEEKEKEEQEEEYDEIKQYDENGEEILSKREKKQLKDAKDRLLEKNNLVADSHFLRKDRIPSKNFACLRVILLTQKEFNPFIDIWNPINLRNESTVLQTLYQLGLSLLKNFSSTFIEDQHLLDSMNNNSSSNSNSNSNNNNNNNNNNNNNNNNNNEGGENNNENDIDNLSLDNNNNNTENNSNKDFNNTSNNEQKKKKQRNDSCSGNDNDNNKNNTNDSDDENDNSSNSEIDLQSSEQMKMVLQYRIEQKQILVSTIEKVVQLMSDVGIEPPQSPESTFTDYENDQDDFDNQPFEEQSSSSEEYFSDTNDDETN

>DDB0219841 pep:known chromosome:dictybase.01:6:2636918:2638502:1 gene:DDB_G0293294 transcript:DDB0219841 description:"Predicted by Dictyostelium Genome Consortium."

MTPSLKDLGNQFYTKGDYDKAIELYKQGIQEILDDNNIVNEDSNNQLSLITSNLSISYYQLKRYQESLEWALKSIEYNPKSAKPYLRAGDAYIELSNYKDAKEKYLLCIKNINTNDETAKNLLNQASNSLQNAKMKQFYQPILEGSPALYDRVEIKYLDSIREKALFAKVPIQKGEIIFSDLPFIHQLSVDSFKLHHNDICNHCIKFIDSSAAIIKCNNTNGCKYQYCSEKCKLESFSYHNQSCMNNSDLVLSSNHPISKYRAMVENVPTSTQLLLVESLISMISHLLKTKQAKNCNLALGTITHLKRGPLMAQQSSFNGKNLEDLQKQYQPLLSLLEDAYGLKLKEHLDDQLLKNEFKKYTNFKIIVFSVDFYDNLLGMINFNSTSTVVKSGKKIEIQVPVTTTGKGKKSNNQVKTTTKTIENSCWGVGLFPIFSCMNHSCFPNVEISNEIIDGVTSVRMVVKAKKNIPAGSEILHSYCDETLSNKERKDILFSQYGFKCTCNKCSK

>DDB0220075 pep:novel supercontig:dictybase.01:CH709175:4129:5337:-1 gene:DDB_G0294358 transcript:DDB0220075 description:"Predicted by Dictyostelium Genome Consortium."

MVHIVPCHKTIDAQHTAQLLLNHVFRLHGYPRTIVSDRDPRFLSEIWERWTKTMDSKLKMTVAHRAQADGQTERMNREIIRILTKASTEYGEICSDIIPLIEFAMNSSMSKSTKMSPFQIVYGFNPPTPVNHFNSLTKTRIPMSNIKKIVRDNILDAQINAQNYYNRGRGDVIFVVGEKVMVKRKFFQTNISKDLISHKLESKNCGPFIITAVHGNNVTLDLIGYPKKHNVFNKDQIVKLYEDSEWLREEISMPEPEEMDEASYEVESILNHDKVKKMYLVKFKGYPEPEWIKEVDTDCEELVREYWNNVQKKQLNSRRERENATTEAVEPIVSPPLSQEVQSSQTLQPITPRQQNSISNQRNQSKKRKSRNQNTSSDDDELDLSLQIKTTRSGRKVTPKSL

>DDB0220651 pep:novel chromosome:dictybase.01:3:5755702:5758268:1 gene:DDB_G0282427 transcript:DDB0220651 description:" shared a short region of similarity with histone-lysine N-methyltransferase, H3 lysine-4 specific MLL3\x3b contains a single HMG1/2 box"

MNTTITNNTMNIVPISQQQQQQQQQQQQQQQQQQQQQQQQQQQQQQQQQQQQQQQQQQQQQQQQQQQQMEVGDTPGTYLCSKCGQIKKGHLCPEKRGGGVTISGVAGSSIGIGGISNVGGVIGGSGASSSGVVGIDQQVMPMFGMQGAMATRRANIQLQQIFQQQQQQQKLQNDIQLQSFNEYQQLLIEQHAATSYFSYTLTPEQKKAREKNASDFNSILLLERQERYPHYFDPHTKTLQVISSWGIKVSDKWNDVPEFEKKLINYQNQQMINNRYVYLQQQRDIHEIQKEMLMNHQKQEKQIARYQELERDRKKKIEEEIEAIVRPRSNVSAYFHYMNINREDEKRMNPDVPLSDISKILGAKWKQLTPDDQKEYYEKAREDKIRYENEMVLYNQKCKEVENSFPQINFQLLINSPPPQPTQQQLQQLQQQRQIQQQLQQQQQQQQLQQQQQLQQQLLQQQQQQQLQQQLYQQQLLQQQLQLQQQQQQQQQQKPMQQPLQQPLQQQPMQQQQPMQQQQLQNQQQQQPLQNQQQQQQQQQQPLQNQQIINNITSSSTSTSGQQFTCDHCSQLDLNSNLITCSSCSKKYHAKCLNLHQKCIDKYREDPTQWKCTDCKSCELCDDSGHDEKMLFCDVCDKGYHTFCLTPPLSQTPEGGWRCNDCAFCIHCYSRVDKNSLNKIKWKENYTCCDSCFSKGFSEKSKYCPICSHSIKDEGEEEDSITTCQYCHKSVHDHCDQNIIDNLENEHFIYKCPNCISDKQIKPRGIIGVVEEKTPKKSLGKRKKKDDEDDVNIED

>DDB0220707 pep:known chromosome:dictybase.01:2:2883211:2884452:1 gene:DDB_G0273393 transcript:DDB0220707 description:"SET and MYND domain-containing protein DDB_G0273591 (EC 2.1.1.-)"

MFKSFDGLKLSNSELEGRYIIANRDIDIGESILKCKSYFAVTCEDFKKNSCYNCIKLIKSPSPQQVPRCFGCNEVWYCSEKCKQDNQAKHQHYECAFFNNIKSPKLIQNSKLDFDSYSEIRIILGLLSRYYQDKLLNNKFNSSIIINNQQDDEEDFIKDTLDGVLDLVENDINEETNSVAKEYIDNIIEYIINILKLTINNNSNDNNNNNNNNNNNNNNNNNNNNNNNNNNNNNIEELIKLIRPLIQKVRCNQFGIWTKNDKCIGMAVSPSSSYFNHSCIPNCESVRDGSDMTFKSLFPIKKGDQINISYLALDKSTKRRRDYLKFGYYFHCQCPRCNSTDIDPTGKLEDSLDNWISKFYCHQKKCTGLYYSKLKLSLQSLTNIDNHEIQLSCSTCNDQLIVNSNFYLNKPNY

>DDB0220708 pep:known chromosome:dictybase.01:2:2884698:2885858:1 gene:DDB_G0273253 transcript:DDB0220708 description:"SET and MYND domain-containing protein DDB_G0273589 (EC 2.1.1.-)"

MFKSFNGLELKSSENEGRYLIATRDIQIGEDLLKCKSYFAVTSETLKTTSCFNCIKQLPSVIKLSLKCNQCNEIWYCNEQCKNENINKHQHYECKFYKKLKSPKLKVYPNFDIETFTEIRMIVGLLSRYYQDILLNNKFIEQQLNNNNNNNNDNEQLTNTLDDVFDLVENQVTEESNPAAKERIDSIVEFISELFNLVLLGSTTTKSIINNDDKIEMIRKINEKSRSIIHKTRCNQFGIWTKNDKCIGVAVSPSSSYFNHSCIPNCTDVRDGSNMTFKSLYPIKKGDQLTISYIELDQPIQDRKDELKYGYYFDCICPRCNGDSNSIDSMDNWISKFYCSQKKCTGLYYSKPMIPILNTLTSNHEIQLSCSNCNNINIVTPSFFNK

>DDB0220710 pep:known chromosome:dictybase.01:2:7763036:7764910:1 gene:DDB_G0277331 transcript:DDB0220710 description:"SET and MYND domain-containing protein DDB_G0277331 (EC 2.1.1.-)"

MIPNNINNRKKLKLPELFKEKYGFNKKGIELRYCDGEKGMGIFSNRKFNKGEKIMKIEPYVWSVAKHAIVCDECLKNKLDLEEGKTLKRCSNCKLVYYCSTDCQTKAWKIHKQECKILSTIPSTTDKKNINTKSTTMLLRLFIKRNLELINNNNNNNNNNNNNNNNNDNHITGQYEIIDGLLNHKDIRSDNNEYKSFSSGFCSLLGEDPQLKAPIVLEYLLKLEPNCITIPRCEASSIGLYPLMLFFNHSCKPNISIINNRKELLIITNKIIEKDEELFINYSPAICYRNERLDNLKQCFFFNCKCTLCLGEEKIKSKDLYITCNINNCGGRINQEIDININNNNNNNNNNNNNSNNNNNNNNSNEEILKCYKCLKVYKGQEKDEILKKKLIIKNLQNKLSTNTDQININQEFKSLLELYCKEIHPTDPLFYEIVNKTQLFYLGNNNKFISDNELSTIYHPRYQIMIKYHLLQVQNLEYEYCRQMLDYVNTLATTSYFKDALNILTDLMSNHLSQIDFYGFNRDELLSLLYNLQHEYKNNIKTSRKIIN

>DDB0220711 pep:known chromosome:dictybase.01:6:1256247:1257987:-1 gene:DDB_G0292140 transcript:DDB0220711 description:"SET and MYND domain-containing protein DDB_G0292140 (EC 2.1.1.-)"

MDGVIESPSNNTIKISPSTSDSSTTTPIITTPPTQSTATVTTKAAATTTTTEASTTPPPPQPTPTPTQSTATVTKEVETTTETIPPIVTKGKIKKSKKSIKKPTIVKRPTTPIDYKQWHTEWPIHVYSHPINGRYLVATKDLDEQTVILRDLPYTWAVDHATCDSVCQHCFLEVPLNQQILPTDFYMCEGCQRVGYCSANCRCIDYSQHRFECQIFKELDTEEYSPFLMSEIKLLVRTLSRKWLEDSITQTAGIDINDETIKKQNTYNQYKNPQSLIPQDNGLRYNDYAELVSNVENYNESLKESLSYWICKYVVKLSAKLGKIEDEFDLLNILLRNRCNAFYIQGRPRDGSSGESRGCGVYVRNSFFNHSCDPNVNYWVVNNTLEVECTLLKNVKEGDELTISYIDTTSPLNKRREKLLEGYLFNCLCTKCVADESLPLDQTGTLEKDDDDNDDEKEKMDEDDDEKDDDINNKNDKKSKYKSDGSTDDEEDEDNNNNKNNNKNKNNNSNNQDHQNNDKSN

>DDB0220712 pep:known chromosome:dictybase.01:6:1666854:1667990:1 gene:DDB_G0292454 transcript:DDB0220712 description:"SET and MYND domain-containing protein DDB_G0292454 (EC 2.1.1.-)"

MNKILIRNFCTNIKNESTKPLIDNIYKNILKDNFKIVEIKDCKNNRGLFYNPIESPTQTLSVINPKTNKPNLIFKEEPFISYPSIIKSNENICNHCLKEIKKEEEEIKQECEECKVYKYCSIECKEKSSIEYHSVLCKSTGSGFNYLEKHASIEKRRFPLLAGKILARMIMGYHLEKSSKSTWLPLQMLSFAKKPPPLEWKDDYLIFSRSLLKGINNESMKKKFDYDWFVRVMQILYLNTIGIDIDPNQQSTKMSSPESGIGLYLLTSFINHDCDPNAFIHFPDDHTMHLSPLKPINPGDEITISYTDTTKDLVDRRSQLFENYGFNCECKKCLNDLLIKRNK

>DDB0229864 pep:known chromosome:dictybase.01:1:3520960:3522817:1 gene:DDB_G0269768 transcript:DDB0229864 description:" identified as a suppressor of smlA null mutant cnr9"

MIKALAAVKKQIEEINEYEKRIGPDCNLSQFQVTLENFNKLRQAQNAVILAKQSKANSGKIVEPTEAQLVANFIEWLKGKGFDESKCKVKIDRNTSEGTGLVATQDIKEGEDFVEIPSNLFITTAVAFQGLGKPPILENDRLIQSIPGILLSIFLVKELSNPTSEWGPYIKLLPKQYNTVYYWGLKEFTQFRGSPNLEYAMRYVRGAMRQYCYLYSMIDRTQSNIMPISSFTWDAFVWAISTVQSRQNPVYAGNGNGSIMALIPFWDFCNHSSTGSKITSFYHMDSNCMTSGAIKDFKKGEQVYMFYGPRDNTQLLMHAGFATKTNLHDSYPFELHLLEGNHEIRHDKVHLLEERGIRDGVVVNLNQNPTSNELPLELIPFYRIYALSEQETRAIAPPQVPGEHNHHHGHQLELKPLAFKIITQENEEKAYSNLVQALKGKLASYPTTLEEDEQELKKNPPANQRFILYTKINEKKILDRNIKYLESLIKKGVMNVTFKLPELVEHTEHANHDHEHGDNCNHEDHGSHGHSHGGDDSHGHSHGGNDNHNHSHGGHGHSHQHGANCNH

>DDB0233369 pep:known chromosome:dictybase.01:1:2983180:2987875:1 gene:DDB_G0269554 transcript:DDB0233369 description:" the D. melanogaster ortholog, su(var)3-9 has a central role in heterochromatin-induced gene silencing"

MDIKDIFFIDDSSEGSDDEISFICSTFTPNSSNKNNNNNNYNSNGSNNTNNNSNNNNSNSNNNNNSKSNNNNNNNNNNNNNKNKNKNNSSNDYIENRKKLAKQSTYEPYVNYDQKHNPYSITSPIVLDEIVEEVRQDLEKRYKPPISSSSLSSSSSSVANASVLNHNSNYDLPQVHISPSMVEIKDNDIVFTSLPTSSVSTPNSTPTSILTTNPMFLATTTTTTTTPTPIPTPIPTPIPTLTTTTTTPTTTTQTPSIFNRSNNIAKTNLNNINGNDSNKKSNSKPYTTLEKLQEERKRNIEITSGLTKKGTLELNKYNNNKNKETPVDIYPLEELESMYVDMYVKPVNNKPKGKIEEKKNTNQLASSSLSSPSPSPTSTSTLPSSISTSRLSSSASTAPPTSTLPSSISAPRSSSSSNSSTPSSLYSYLPYRYSSSSSSSSSYPSSSSYPSSSSSYSSYSSFSSPRKNLENNREFASPRKDDISFIERKELTEKQQKENERLEEQRISLLRLETERQNLAKQRLEKERILEKQKLNDERLERVERDRRNRLEKERLEKEKLEKERLEKERLEKERLGKEKEKEKEKVPSSSSSSSSSSSTTQNQIEQSSGTRTARKRVHVAPKDIRKIILGKLRSCLDAEDIIIKDLSDEVKMNVCEEIERFSFGSTQKKQDYVKKLAYQYTLIRGGLVPTYLIPLLPESFLSTLSPSQFITPAIATLMQNVYESTQHKPETNSSTNQINPSSELSSSYSSSTSSSSSSSSSSSSLLSSLLSIGTENTTTTTTTKTTTRSTSSHLQKDIPIKLETSMESSQNSTSTFRETQEIINNDTITELERDLKGMKFGDCIDAVTSEVIVKELIEDYSTSIINRNQLPSGMNLLRKYRNLIRSEYGQPIDNIVIILLSMVTDVSFIELKSKYLDLYKQDCKNGLIKSDILNIDMPYFEPLNEYIQRQKKSLVEIDETDLYNPNTNINQNNKSNKNNNGENKIQNDKSKISKKIFKANLMLENEFLYIFENSGKLVHVKKEIKRKTKIATSSSTKVSSSSSPSSSPSSSPPQLNGTNGKSRFSSQQKLPCLNCKRANIKQCLGNPCTRCLLKRSVCIQDRAYLDAENGSSDILLNNTSKKRKSIDEHNNGHHNHSRNNNDNNYNNDNDNNNNNDSYNDSDNDNNDNNYNNNNNNDNNNNNNNNNNNNNNNNNNNNDNNNNNNNNNNNNNNNNNNNNNNNNNNNNNNNNNNNNNNNNANNCHQQKKLKAQWIDPDIARGVYTYPLKAINEVDDIPLTNSLVNFKWIDKSFCDRETLNVKEFLSGCDCVGDCHNNPNCQCILEGGIYYSDQGTLTGKNIEGPIVECNPRCKCSHELCKNRAIQQGQQNSFPLELFKTSNKGWCARACIEIPKYTFVCEYVGEIISHDEAEERGLRYDTQGLSYLYDLNGDSNCLVVDATHYGNATRFINHSCSPNLISIFFYLDQRIEIDKPRIAFFSSRTIKEGEELTFDYRYNLPSGIQNKTNIPGGILCHCGSSKCRKWLWVPEVSKKRK

>DDB0233375 pep:known chromosome:dictybase.01:5:2551579:2556233:1 gene:DDB_G0289257 transcript:DDB0233375 description:"Histone-lysine N-methyltransferase set1 (EC 2.1.1.43)(Histone H3 lysine 4 methyltransferase)(SET domain-containing protein 1)"

MENETIVDNSLNNKSNVNNSNNDINNSKSNNNNTNTNYNNNHNNTTTTTTINKTEEKQNDSPKDSEFEFLDELKGVDDQHHVFSSEDESYTNGNKKRKQTDTPLSPNQDLKKRSITSPTTSPTTSTSTSTSTSTSTSTSTIINNNNNNLKDKTKEEIEFIKHIRSQLVKPKFLKDKPNFPLRSSGGNWIFVGKLPSLQSTTTDNTTLMSPNNATTTNGSSSNISTTTTTTTTTTPTTKILYRVNGFLSDNETIDSIEINFGDPRDRYEIERLHSSRINNPFELPCVSFKNPLFIKSNIAKDIGISNEYGGMNDSFEFSNQPPPPSPPPPPPPTLPPPPPPTLPPQHSLEQQSTKQQIFTQQQQQQQQQQQQQQQQQQQQQQQQQQQQQQQQQIPKINQQHYSTQPSVLIDDIYDPSNPTEPISPHQDHYPNFIFSKLQRYEHLPTRNPISQYDYRDRPRDWERDRDRDWERDRDWERDRDRERDRDRDRDWERDRDWERDRDWERDRDRDRDWERDRDRDWERDRDRDWERDRERDRDRYDRQTNFSPAPQSTTTSASTSSTTSSTDKNSNNTTSTSVSATTSTTKRKSKFSEPIEPSPFAIQIPRDNIKINGNLINNSSSSSSSGNNNNNNNNNNNNNNNNNNNNNNNNNNNNNSNNNNNNSDVKDIKDKLLKQFKIYDPVNVYMDESYWYIDFRSSESRERAIQVLNGSFIDTWKLNVDNKKTNTINEELQKQKQLENDSNNNKPNNFNLLENERSLKEICKLLVATELLSTSSKDISKNFIEAEILKTIKLLDSQRIDPLTQNSTIINNTTNTTTSNINNTSNNTTVTPIVTPKSIISAPTSRDSPRGGRSSSTTTKKPSKLDLNGSGVPPTLKKLDTIKQQQQPQPPLSPLKRPPKSHFYSDSEDDGNNNNDDDDDDDDDEDDDFDQELSPLHSSRDSKKNIKSIIKKKPIYSDDDDDHYHHHNHHHNHHHHHHHDRSEVELYNESDLQVDVLDSDNENQDESDYHKSSDNFGHVELSDDDNEFDSLDTDQDLYDTEENDNGKKSNKRPRKSKFNGKSKKPTTTTSTTTTATKSKGRSKKTTITTPTHNIPVLDEIQSNLDDEDASYVSMVMAADKDIKLLFSTKSEEGFEDSSQEILSTPTRTKPSRNRKERNLPFLDEEDDESFKQLPQPQQKQEKQEKHEHKLKNKELKQKNNEVIINKTEEHFSENLNGDNNNNNDKSENENENENENKNENENDNNNLNTSIDNINGVERRSITGCARSEGYTRSDIQKLFKRKQVAPTGKRGAASSASSGSNSSSSSTAESFETGGNLSKSARSSRFDNRGFGSDPITLASLKSRRKRIKFERSDIHDWGLFAMETISAKDMVIEYIGEVIRQKVADEREKRYVKKGIGSSYLFRVDDDTIIDATFKGNLARFINHCCDPNCIAKVLTIGNQKKIIIYAKRDINIGEEITYDYKFPIEDVKIPCLCKSPKCRQTLN

>DDB0233544 pep:novel chromosome:dictybase.01:4:1785353:1787081:1 gene:DDB_G0294595 transcript:DDB0233544 description:" multi-domain protein\x3b the TPR superhelical structures present a surface that is well suited to binding large substrates such as proteins and nucleic acids"

MTETTTSLREIGNKFFKEKRLNLAIENYNKAIEFDPSDYQSYTNRSLAYFQKKEYENSLEDSKEAIKINPQWDKAHYRYSMALKQFNRLDESLRSLYIIYNRFYNSTNNNNTTTSSNSNNIDINLITKEIDNIQKLLISNRYSKDIISQSETFSNSINANVKVDYINANLGKSVFLIKDITNYNQPLISENPLVSNLSSLYFDENYKSCFHCLKSIFVNESCYGEKMKKQKELFIEKPSFTCDKNCKFEVYCSENCKNEAWKQYHNLLCPNYDLILKLYGFCRIKNQIFPLVIMKMLAIVLNDLKNNKKSLEDSLSPFTSFQYDLSDSILSDDQLIAYAFIIDIFKSILKEKNDEIIFNQLINKERYYQFNSILKLNSSDVQPLSILEELIDNKVSKAGIKETNIKIGDEVIEIDSIGEFLEKNKLPTISIKGVGLYRVINSINHSCEPNVFCSFSKNDHSMTIYPTPKMQLKKGQEINISYINEDLPFSQRQKLLKENYSFNCNCKKCKNKE

>DDB0234108 pep:known chromosome:dictybase.01:6:1603469:1605457:-1 gene:DDB_G0294629 transcript:DDB0234108 description:"Predicted by Dictyostelium Genome Consortium."

MLNRLQSLKKNENLIKFYFNNNYNNLTRYFCTGGSGGGSDIFSTKNSKDAINSTIRSKEDIEEEIKGFQNAPQIKPKIPVDPASLHLSYSIQIAIQKAYDEFMNGKPEEALKRYYWVLSHTPADYAVRIQRATVLESLSRLDDAIIDCNTVIKNSTEGEILSEAFVIKGICLVRKEKYNEAIVAFEKSLLLIHNPKIVDLKREAEMMLYPDTVIVPNHEDDYEFFNGLSSTLMGNAMVKKSPIHGRGIFATRDIEEEELLFEAPSLLSISTNLAKHKYEKHDDEHCNNCHLSLQPVELQNDREISKSKEFPKIEDTLSRMTNLPLGSISGVCCPNCNEAIFCSSECEAQGMARHRLICSGTPSNVHTNFLNKFYHDISKLDDEERTEYLLMLQVFSLQYTTGGNQDEPLRSMQMDQFLKRLVHTEPSKNHTTSYLSRKDMKIYQSIKGIFSNREITQEIYHRVKSIIRLNAVAFPTSRIKILSEKNPMDELGYSFDFQEIPSQQLASILMQGSFFNHSCEPNVFIATPVVNDKSIRFCTRRPIKKGEELFISYLDGEKLTTEKRRTTLKETYSFICNCQACTSRKTIDFLSILD

>DDB0237830 pep:known chromosome:dictybase.01:1:1516724:1519586:1 gene:DDB_G0268132 transcript:DDB0237830 description:" contains a SET domain involved in histone methylation, an AWS (Associated With SET) domain, and a post-SET zinc-binding domain"

MNKKVFLNNYLNERKQLNGNEINNNNNNNNNNNYNNNNNNNNLNKDKDKDKDKERDKDRERIKERTKERGDKERDRDKERDRERKKEKVEKPQVAVLKQSAQHVKQQRLKEKEKGKEKEKDKEKDKEKDKEREREKEKEKEKVKDREKEKEKEKEKEKEKVKDREKVKDREKEKEKEKERDKLKPKDSKIKERDIEKEKVRDREKEREKIRDREKDKNSNNNIIKPKEKKDESIAKTQKNITIKENGNITSSSSISNSSSINNNNNNKIINKSVSTNGNGNSSNSTNNNNSNGSNGVDIKKKPILDSKKRALPSSSSKSQTTSTSTSTSTSKLSKPIVKKKKDSNLKRIKLSNGGVVTKYKRKKSNNDSGSDDSSYDSSDDSSGNDSSSSGSSSSSGSDDSDNSSDDDSDSSDGDSSSDNDDDNDKNSSGSDDDEDGDSSSFDSNYSGSYDSFSDSSECDDSDCSCGKKRKNKPDSSNSKLTNLKKPITNGNTTDNLDNGLTTSSIVVDDQQKITNEYIEKHLNSGKANPDYSDIAVLIQKSRNKKFGFISRNFFIERTEKILYEIDDIDICNCSKSSGSVCGDDCLNRESYVECNIEHCELGKKCTNQRFQRKQYSNIKPAFTGKKGWGLIANEDIEEKQFIMEYCGEVISKQTCLRRMKEAENEKFFYFLTLDSKECLDASKRGNLARFMNHSCDPNCETQKWTVGGEVKIGIFAIKPIPKGTELTFDYNYERFGAQKQECYCGSVNCRGYLGQKSKSSTSTTRPKQITRWKNSIVLNHNHHVKYFLGEPIIPHSNPSSSSSSRSVSTLYNSELSPEMELQLINSKRIFLHRNVRKLKSFYFNVFNYYSSGGGSGGNNKKLINITSTSTSNSSLNSSLNSNIKLKRKNKISDIFNI

>DDB0238220 pep:novel chromosome:dictybase.01:1:4811940:4818335:-1 gene:DDB_G0270410 transcript:DDB0238220 description:"Predicted by Dictyostelium Genome Consortium."

MSEENKKSSRKSVARRALPKNILESPVISSTSIDISSVKTEPPPSTLFKQLDFTNLFGSLKSETPTSTTSTTNTTVTASTTTTTTTTTTTSSSSSSSSSSSSINTTTVNNISSNNTSTSIPLNSPKQPTTTLTSKPNTPIHGGIIKDSQLPKISLEADSPTIQSSSMNGGENKNDSTLVPISILNNNNNNNSNNNNNSSNNNNNNTGSTNAASNTTSTSGINNKDHKDRNNNNNKLPTDRDMEDIPDKPPILSSKKNHSTPPTSSLTIATPNNNNNNNNNNNNNNNNNNNNNNNNNNNNNNNNNNNNNNNNNNNNNNNNNNNNNNNNNSNNSAPSVTTPRKGKSKTKTPIQTSQNHIEKEKEKEKEKEKEKEKEKEKEKDKDKDKEKEKDKEKEKDKEKDREKDKEKDNKEKDNKDQKDKHHHHHHHHHSEKEKEREQQKDREQQKEKEREQQKDRELQQQQQKEKDKEQKEREKEFKEKEKEKEQKEKEKEKEKEKEKEKEKEKEQKEKEKLEKELKEKEKEQKEKEQQQQKEKEIIHKTVSKKAAAAAADKLKAEKAKAAEKAKAAEKAKKEEDEEEEEDEDSDDDSKESVIRCICNNNIDQGLMVQCETCDKWQHSICYGIKGANNVPKHFYCEQCELKIMDCTCGKKECLVGKIIQCLSCHNWSHLSCVQSKNTRDIPDPFTCHTCEKLDNLESNLDIDESTTPSRLGPNTPNAKGGPGGKKRKSTSTGPNNRRKKQTTTTTTTTTTSNSTPASPIAVGAKKPAASTAQSSTPLLLEESILPPAPPPLTSSSSSSSSSSSSSSSSSSSSSSSSSSSSSSTTTNYQSPSISSTTLNTGSQTPKLKSETLMSGEVNMEIDTNNTPLLSSSSSLGENITTNPDSGKNTPMLDSDNGFPSLLKDFDVNSKNIGFTYIEDTMSINALKEKFNSIDIEQPSPYFQTGLDLFSFLKSNYIKSQFIDDYCKRKSIKQLKEVGKLCIYNLTVEDNILFQKYCLLFIEINEKDRNDFKKAISLLLEIDESKVNTYMIQYAHELSKQIESNYNQIQYYQHSSSSSDHHHHDEEDEDEMKIDQDEISKWKSIGLENSFDKDFIIPNDIPSNLIVESSKINRDFDKIIFGRLNSRPLLIKKELSNNNSNNNNTTSKPLIIKKESSNNNNNNNNNTTTTTTTTTTIEQQHQHQKPPQQQQSQPQQISSEQPQKIILSKLGQNENTFIGECTGIFKNQIIDIKNEWVDSNGFLLNTINPQILMFNSTYQSFNNTSYLKNTYNPKERDLCIDARSGSVCKFIRRSCLPNSKFQFSNINININNNNNNNNNNNNNNNNNNNNNNNKNNLKVSIFSLSQIEKNQEITIDFDYPYKCLKNRIYCACGTSTCSVSIWFNERASFGIKMLEMLGETVDPELKKRSLECTELYQQQIKQELQQQQKQQQLQLQQQQQQQQQQQQQQQQQQQQQQQQPPQQLQHQQQQESQQELQQQQQVKQEGQIIENDDNFDNNNENEENNVEKNIKEETSTTNMATPTQKKKRKGKMSSLEMDSIFFNTPSDKTSREDRKLQQIIQNFEQLEKKEKEKNNRKNSNNTSNSNSSSTDTSPSLLSTTTISTTQQSSPTTSTTSNSNGNVSPKSTKKKKPTDSSPSDENEDIEMNEKTPTTPTSAPISASLSSPPDDEGNKSKFGKKAWLAEFKQKEKDLPCIKEEERRADDSDGHQEEEGSLPNDESNNTNNQTNSPNNTNNSNNINNNSSGSINNKLNDQPPPPPPNQPPPPPPSQPPPPPSPHHHHHSDQQTLSKSSSGYPHERELRNSYTPSQSPNQSPSMKGGLSSSIGGFRDPIDQPYRLNRERSGDMGSLRENRDNRDLPLRGDPRDQVREPRDWDRRGDNWNSYNDRDQFSGDRGGGGGNRYGGRFKDDHWDSPHRKSGWDNSLRYNNDKNRIPYDRNVDKSGWDRRPNYPRNGFPSPPLSSYHNDDYDMDGNEPLPSQQSQSQQPSTSSTSSTSSSSSTSQTNTSGTNKSNPPTPSSNKSPQLLDSPSSFKYSNNNNNLNSNINNTNNTNNNNTNNNNNNNNNNFYGNQRGPNYNQNRSGNSPTLPDEYDQHNQWGRKPYFKRR

>DDB0238659 pep:known chromosome:dictybase.01:4:1478818:1482847:1 gene:DDB_G0284059 transcript:DDB0238659 description:"SET and MYND domain-containing protein DDB_G0284059 (EC 2.1.1.-)"

MTKKIKLSNSESNKVNNNNNNNHGNGHNHNHSHNHGEMCHGHGIIGIVDSVTGENIIQVNGENLRSVPNGHSLIHNINSIINSHSKHGKIKNILKDSTIISSSINLSDKPINKITEENSPPSSPTLSSSTNTTTDRQELPQQQQQPQQQQSQPSTPPPQQQTNQKQQPEFNRYQANDILFRNKLTRSLSRNIPPLSLLKQNFKDEMENSFHTAISNIKDLNEKVEMVNLIFQHNLSENNAMVNVDSIIHSSTSGLLSEYKQITETFDWEQTSKGYKNKGNELFQKKQYSDALLLYNESLRIYDMELAAAATSTNLNEKENGGGGNVGGSATATATTINTPDVSILSSIHSNRCLCLVNLERYKEGAIEATRGIDLVGSSSVLHKLYYRRGICYYHLRKHYKAKKDFLRAHTLIEKRDSSDLASIENYLFKIQKLSLPLQKDEEIEQELDNKNNNSNDDEKQQQQQQQQDIDVTISSILEKSESLIDSRVEFLYQSDLVGRISEASDFIPSNTVLYQEEPYVSCLDRNYHSQYCYNCFKEILSPIYCKECSNSQYCSNKCLNEDYVKQHGRECGKGFLIICSHESLLVIRLLARKGRDYREANKGKKEEEPQQQQQKQQKSRKLPNLTFIPKPNPTQIDQNLNKPKIVLPEPTTAAINTALSSASTPTTATATTTTTTTTATTPTTLAETLSSTSLTENKSDSTPPPTPLPPSSSSSSSSSSSTTTTTFSFNMSELQNLQISQDDELFDVPTDPALFGKSNTYSQSYELINSFNPHFEHHSNDSMANMIFDAFVIERFLLYYQKDLGILSEDIDVHVILRHLCQLTTYTFAIPGYIDNHDSLVLKTLQLQQQQQQQQQQQQNQQSNQQPNQQSNQQSNQQSNQQSQPNQSPIIQSQNPPHASPFSPLRYSVQKYSQDKIGYAVYPMASLMNHSCDNNTHLQYDGCSLTIKSLFNIEKGEEILGCYGPHAFLNPLKDRLINLYNEFFFVCRCKACSEKSGPDPIKCPGSYHDHLNSPESSPVECSGTLLESINMNQLVTMAKLQQQQQQQQQHQQQNDKKKFNSNPTNLGSNNNNNYLNNNFNNPLRNGNPFLLKQTYDRYDDHEHRYFCCSKCGIELNGFDSFSLTSQIIISDNLFEMGFKAMTLYGNLSKDIETMLLRALELRKSIFKPCSKKIGDIYDSLSRFSISREDGASAAKYLELLIENVISRLGHSNSADLGREYSKLGQIYLTLGEIEKSEDAIEKAESILMSWKSNDPTDEEVLFLLTNRRKLFTAAHLINK

>DDB0252650 pep:known chromosome:dictybase.01:5:2604715:2606297:-1 gene:DDB_G0289303 transcript:DDB0252650 description:"Predicted by Dictyostelium Genome Consortium."

MKKDEKQFQQFPNKGRTVQANTDLPKGSTVFRCAPFASSIEDSDKLATEKYCGFCLQRIPKSDSKECLCKNCKLYSVCKSCRSINESAVEPFQIIIKSTFHLGSDTRDMRLLLRIIANIANGKQGMQTPIDDYQDFMGLTSTLDKVDKEHMTKFKRGVTSISSLISSVRGVGYLKNTITIHEILECFSSVLTNAHQFSYATSKEIGRGVCPTGYFNHSCMPNTTWSLDDQGMLLFSTSSNVKKGDELSLGYLANEYPLKNRRRELLDGYYFFCQCPLCEFQSNLSGYLCEKCKEPLLNDSIVYHEPSLTNPLDTGIGLIKHLRKMIDFRQQSVEFKKMFSSEQLSNILQKGVPKHIQSVFNCDASDNEELQQIVEISERSIKSLPLINGEPIDFYPLSDRNYYEYFKQLKNSKISSPEELQRIKDKILSLINPTIYNSFKDKIDKRLLI

>DDB0304823 pep:known chromosome:dictybase.01:5:1280700:1282179:1 gene:DDB_G0288227 transcript:DDB0304823 description:" similar to the mammalian SET domain-containing protein 7 (SETD7), a histone methyltransferase that specifically monomethylates Lys-4 of histone H3"

MKKKIKNTNKKIVKKKPQPVNNNSNKNKKPISINNKINKEVVKIENKSEKGDDFLYVGEFINNEKNGKGIIYYEDGTLIEGNWKDGELNGQGIFKTTELIISGEFIDGELSGQVEEIDKETGKLVFKGEYSQGQRHGRGKLIMIEDGGELTGTWVDGKMTGYAEYTFPTCNGRFKIQGQWLNGDLVSGKYNINYQPLRNEIDGKRLPIDDREREYMRRLEHYQQKELSKECRNFILKLDESNQNTISTSPLHIDLYERFHCFVGKQSTIPNSGEGLFAKIFIPSGTIISFYNGIRLTHQLVNSRSWSENNNTISLNHETVIDVPPHLNDTVTQYSATISHKANHLVPANNSVYASFYHPRFGDIKCIKAIKDINENEEIFVDYGYSNNDTPSWYKVK

>DDB0304850 pep:known chromosome:dictybase.01:2:3147334:3148575:-1 gene:DDB_G0273591 transcript:DDB0304850 description:"SET and MYND domain-containing protein DDB_G0273591 (EC 2.1.1.-)"

MFKSFDGLKLSNSELEGRYIIANRDIDIGESILKCKSYFAVTCEDFKKNSCYNCIKLIKSPSPQQVPRCFGCNEVWYCSEKCKQDNQAKHQHYECAFFNNIKSPKLIQNSKLDFDSYSEIRIILGLLSRYYQDKLLNNKFNSSIIINNQQDDEEDFIKDTLDGVLDLVENDINEETNSVAKEYIDNIIEYIINILKLTINNNSNDNNNNNNNNNNNNNNNNNNNNNNNNNNNNNIEELIKLIRPLIQKVRCNQFGIWTKNDKCIGMAVSPSSSYFNHSCIPNCESVRDGSDMTFKSLFPIKKGDQINISYLALDKSTKRRRDYLKFGYYFHCQCPRCNSTDIDPTGKLEDSLDNWISKFYCHQKKCTGLYYSKLKLSLQSLTNIDNHEIQLSCSTCNDQLIVNSNFYLNKPNY

>DDB0304851 pep:known chromosome:dictybase.01:2:3145928:3147088:-1 gene:DDB_G0273589 transcript:DDB0304851 description:"SET and MYND domain-containing protein DDB_G0273589 (EC 2.1.1.-)"

MFKSFNGLELKSSENEGRYLIATRDIQIGEDLLKCKSYFAVTSETLKTTSCFNCIKQLPSVIKLSLKCNQCNEIWYCNEQCKNENINKHQHYECKFYKKLKSPKLKVYPNFDIETFTEIRMIVGLLSRYYQDILLNNKFIEQQLNNNNNNNNDNEQLTNTLDDVFDLVENQVTEESNPAAKERIDSIVEFISELFNLVLLGSTTTKSIINNDDKIEMIRKINEKSRSIIHKTRCNQFGIWTKNDKCIGVAVSPSSSYFNHSCIPNCTDVRDGSNMTFKSLYPIKKGDQLTISYIELDQPIQDRKDELKYGYYFDCICPRCNGDSNSIDSMDNWISKFYCSQKKCTGLYYSKPMIPILNTLTSNHEIQLSCSNCNNINIVTPSFFNK

>lcl|DDB0220662 pep:novel chromosome:dictybase.01:5:1329697:1332090:-1 gene:DDB_G0288277 transcript:DDB0220662 description:"Predicted by Dictyostelium Genome Consortium."

MSNTTTSSSSILFSFFKRDNSGGIEKNEIKKTLTKKRQPLTNKRKDPWGSDSEPDELSLPNKKHTINKITNALLMDDDGGENENDKDDENNENARIQIKDSSENKKKDVAINKNNKESINNNNNNNNNKTINYNNENERKNIKSLLSKTFESISSITTSPFRLKTLEKPPPKEQLQNSIINKQLQKPLPSDSKPKYTEQHLKSKTAHQKSTLNQSLEPNDQQTPNSIDQSLETNQPLYLESPKKTAQHQTGNIDPSKTKTTTSAKTPSTASLPIANDNGMKNSDNDSNKTSDYTTSDFNSSGDNSNDYNDFNHSNNRDDHNGYSGIEPINEKEKEKGNENENENENEKGNENENENENEKEKENENENGNERKKENERKKENERKKENERKKKTEKEIQNKENKIQNIKSKTPTKNSTLKMEATKECRHEICLKGANGSKFTNNSKDHVLRHENSSHYDCSTNCQMEKGHNPKPKFKCGLCNSYYSTKRYNLIRHIKAIHKDKKTDINGEEISKIENELKKKKECKHCNKIFSTQSNLTKHIRKFHKKSIDTITRFSCEICDSSFKRNDYLLKHIKSNHPKIQYNLDDNNNGDDNYDEDDYDDEDNYDDDDNEEEEEEDDDDDDDVDDGDDDNNNNNNYHNDHTLYNNNNNNNNNNNNNNNNNKECPAQNSVSGQNSDSDSDQNSDSESNQNSDSDSESSLSSSFESGIEPSSDESDLSEPDISSEPENKPSSKVSFKSKTNSTLESNYEYQDIDTCESQTNNDTNVSKDSIFISISEIDGSSSDSDEYFTDAEDTL

>lcl|DDB0304751 pep:known chromosome:dictybase.01:4:2306298:2308594:-1 gene:DDB_G0284659 transcript:DDB0304751 description:" contains a predicted signal peptide"

MKFKIIYILIFFSLFFIKINSQLPPNPTAVKFEVSKLLYIFNKTDSYFTKLVLYDYFYPRYEMAPNYFNCTISRNDDRVCIFHSDEPFSRLWGSLYSRVYFREVSSDNEYSTQLIQTKFPAPYDVSFSRYPPTSGGDIVINGTFLRLVGGPSRLLHSIENVEPFVVMGNFSDQSFNCNSIKVTIPPGSGNFNFYFDDERIYNVPFSYAPPIISSTINEFSRSIIVINGDNFFHDTSLIKIYFDNIPQPNPKITVNHTQIEASCYHITDPGPLSIHIKVNGVSSEKNSSVCIPAIVKSITSVSSKIGGVVTIEGSKLFSVSKPNLIPIIEIGGKSCVYLQSIENTLVCKLNPDKSGGKNLPIIVKFDGCNSIISNDVTFSYDIPTIINAKVSKGMVIVEGHNLASLKENSIIEVNSQFGDIKIEIKQFQVSPDESNLSFKLPLLKCKNFNVNITIDGTPSILPVQAPIMSSVIKRPSSVNGTLILELYNTKCIFFELMKIPKITYGNTPSMDCSVPSLQTSNSDFYITSCPAPYGTGIDKNYTLHFNSESFENKYSYAPPVIQYRTFSKGQEDLTVHGSNFGNSLSLIKVFFNGSDISSQIRSLNNNQFSFKKLTTYENGMINITVDSIDMESPFYITLPPIIFSINNNISCNGVITINGKNLLTKDKEFQVKVLANEQTTTKIYSSEKSLYVQINSKRSPINTTAYIGEYLIMSKNIEFYKKKSIFDFYFDITKDNC

>lcl|DDB0186030 pep:novel chromosome:dictybase.01:4:2036413:2037999:1 gene:DDB_G0284487 transcript:DDB0186030 description:"Predicted by Dictyostelium Genome Consortium."

MEDLINKKKYFDNILGCIYGQCLGDAYGLSTVCKMKHHVDRWYPKVNENSFIDFPNYKIDDYMEGGHTHDRGDWGIDSDNMILLLQSIIKVFQHTNDVNSNNSSSSSSNNNDELFFKVYRNIFLKNKIWDYIRIQEEEEEDDAILKIFIKKLKVYYTKKGFQELGDWDKNWFSFCRGRQTTQLVVRNDEFLKDPMMVSKYYYLIESTKYPNDTGALMRSSIIPCMNFLNEKQQLINTVNFSKVTHYDPRSVTCCIIQNNLITTILNYYYNNNNNNNNNNNNNNNNNNISNLLLKLIKIIYEDKKIEKLQNYYNEKNGMFENEIDKRRKIGISNYLIYSARDWGHLNLERSLQGGFSLSGLSSSIYSLKKFNQYFNQEKEKLINSGCSDGLINLSFLFRKVLDELIREGGDNNGASTGALLGSIIGYSNLPKDMLQSMPNKQWLDDQVIQFIKLLDERITNGKIIPFTISDDQEQNIYSSIGHNNGETQNYPFVNYENNNNKNKNKNNNDNNDNNNNNNNSNSSKCIIQ

***Drosophila melanogaster*  (Metazoa)**

>gi|24641164|ref|NP_727478.1| CG11160, isoform B [Drosophila melanogaster]

MPGRKKNHHKNNNNSRARHQRGGQQSKDKQEQQDKEQSPSPTEEKELPYRVEHSDIYGRYLVANRQLEAGETLIREEPLAIGPCVSGDPVCLGCYHPVSLKADQYRCPGCAWPLCGSTCAGLKHRHGHTETECQLYAERRAVAGELLTERAGPAEVRDLYELVMIVRILLLRQHDPEQFALIARMESHTEERRQNAVLWRHYEEKVVQRLRVTWQLEDLEAEQVHEVCGILDVNCFEIGQNGAKARTLYPSAFLLAHDCTPNTAHTDDPSSFEILLRTSRRVREREALTLSYAYTLQGTLKRRAFMHEGKLFWCCCRRCSDPRELGTDCSALVCATCRTGSVRAVDPLQQTGDWACDRCAHKMGATEVERQLDRINNDLEDIDVHDIPGLENFLLRCVCIHGS

>gi|24640767|ref|NP_572539.2| CG12119 [Drosophila melanogaster]

MICPKRTSELLDLHLAPFKDKDPAWEIGVSKIAGRGVVATRSLKRGEIIFRDSPLLIGLAAHEEDSLNACSVCLKMLPDTRFMCRQGCGLPVCSLCAKKKQHKSDCDLFKSWGPNEPDVANSVIIRLLCVARAINLSKEQRDLIYCLQANLDNNHRTEVRNAAKCFKNFPTDKKLIEIMNRTVAVLRTNGFDKTTDRTNDNQEFNYRALYPLFGVVNHDCIPNAYYTFEEKTNNMIVRAAVDIPEGFEVTTTYTKLFTGNIARHLFLKMKKSFTCKCSRCSDPTEKGAFISGLYCRDTNCTGLVVPEITGLPHPNWNCLVCKQKSTHAQMMKSQDFASGAINAKVNSNSLRTLVQYLNEKSDSFIPSSNYVVIDAKMSVLQRLQQGREDCSEELAHNTRLRYSRDITQLMDKLGLGDSLLRTHLEELLRREEERRAKRLADEAEKQRKLAELQAKAAEADKLLEVLQKEQSEQSEQAEPAGTADPGTAPAAPPVATAELA

>gi|21355139|ref|NP_648574.1| SET and MYND domain protein 4 [Drosophila melanogaster]

MEFDATYHEICSAQTVQSERRGFFNEFCVDVRDACGDKWLRNYFGKLKSNAARVLSIFSDREVCDPVLGVLEHVQPVFKQKDALFSAQRRAQADKLYLMSGSGDGEESRELLQQALMAANLAVMRAPDRNADPVLDEGLTLALAYRSRASILIRLGEGEAALNDLKLAINFGLELKSSVDYYLKMAKAYAVMGEPARAEISLKIAEKMPGCDATHIALCRKELSSVKPKPKEATSEQVPQLAHGESAELVGASKVVRLVETKDKGRFVVANEGLRTGDVLLFEEPVAACLEPSYFGTHCHHCFKRLHTPVSCLHCSGIAFCSAQCMGEACSSYHRFECEYMDLMIGSGMSILCFIALRIFTQAPSLEQGLATANLLFEHLCSHEEDRQPDDYLRRALMSGFLLRILQKSLYFGRRKTEGVNPTAVELQVATALLGLLQVLQYNAHQIYQTQVTEEHRFDGSKTVYLAAGLYGTGSYFNHECWPSTACHFVGKKLVLTATRPHRANELVAVNYGPIFIKNNLKERQRSLRGRYSFSCSCMACQENWPLLQKLDKQVRFWCTSANCSNLLKFPKDLAKDVRCPRCRKNISLKESVAKMIKIEELYREAARAMEAQKTVEAIELFKESLDMFFQVAALPHKDTIVAQQSLHKCLSDTGTTFKKEGK

>gi|28573973|ref|NP_610202.3| CG14590 [Drosophila melanogaster]

MVSSTECPVCGVAASQACTRCKMVRYCDREHQKQHWPQHKRRCRPFSEEQDAELGRYLKVTQNIAAGQIVFIEEPLVVGPKWYLSDADKEASNVPCVGCYTPCRLGKHQCRRCRWPVCSAGCKHESMECSVLSLGSGSPTRADARSLNDYFRGDALLVLKCLLLQRQSPTKWSALLEMQSHEEERKGTDLYEEAEKRVVTYLQKRFLCRLKQTNPNLLTDCGPEMLHRLCGIIETNFMVIELPSGVELSGLFRQACMMEHACQPNCDFQFDNKTQQVAVRAGCDLRKGDHLRITYTNILWGTQLRQHHLRLTKHFSCRCSRCLDPTEYGTYISALTCLGDVNQTCGGTHLPVDPLDENTQWKCDTCPMIVDGAYVAELQSHMTEQVEGLLAGCPSANQVELLLARLTHMLHPNHFHTFNLKHTLIQLYGNEAGLELGVLSNTQLERKLRLCGELYNVCRRLDPYSIKLAIYVTVILIEVAHTLQEQARRAPAEGTSLLGLAQSRLREAHMVLEKEQESVAGKKLNEKLQMEIFECEKLILALAYNQDHEANTK

>gi|19921122|ref|NP_609464.1| CG43129, isoform D [Drosophila melanogaster]

MTSPPTLSVNRTAVQWSPVCGRYLVAKGAIRGHGLLIEELPFAVGPKCNGPVVCLGCYEPNPDPEEELCSECGWPLCVECAQQADNAHFRLECSQLKDARARFFRLPSGSRHCPQLDCIMPLRVLLAKEANPERWDNEVAPMEHHKEERQRDADVWHADRVNIAQYLRGPCQLANRFSEELIMQVVGVLEVNAFEARSPKGYPLRCLFPYTGILAHNCVPNTSRSIYPSEGYKIRLRAMVDLEEGQPLHHSYTYTLDGTAQRQKHLKQGKFFTCQCERCLDPTELGTHFSSLKCGQCAEGFQVPRQPTEPDTSWNCANCGSDTSNADALAMLQSLQSEVNAVQALPMAAKRLEEIERLLRKYKSLLHPLHFIATGLRQLLIEMYGRVQGYEMVQLPDHQLERKAELCRQVLRVLNTFEPGLSRTRAMNLYELHVPLVLLAKSGFIAGKLKGGELRARLVDAIDLLKECVEILEFEDKSSQEGVLCVVAKQALKQLTLSVEGLGSELD

>gi|24641786|ref|NP_572888.2| Set2, isoform A [Drosophila melanogaster]

MEESGPPSNPSPVASRGRGRGRPPKVALSALGNTPPHINPSLKHADAEASPTAPEDQDSGQSECRRSSRKKIIKFDVRDLLNKNRKAHKIQIEARIDSNPSTGHSQSGTTAASTSMSTATASAASASSAATVSRLFSMFEMSHQSLPPPPPPPTALEIFAKPRPTQSLIVAQVTSEPSAVGGAHPVQTMAGLPPVTPRKRGRPRKSQLADAAIIPTVIVPSCSDSDTNSTSTTTSNMSSDSGELPGFPIQKPKSKLRVSLKRLKLGGRLESSDSGNSPSSSSPEVEPPALQDENAMDERPKQEQNLSRMVDAEENSDSDSQIIFIEIETESPKGEEEQEEGRPVEVEPQDLIDIDMELAKQEPTPDPEEDLDEIMVEVLSGPPSLWSADDEAEEEEDATVQRATPPGKEPAADSCSSAPRRSRRSAPLSGSSRQGKTLEETFAEIAAESSKQILEAEESQDQEEQHILIDLIEDTLSESEVTSSVSPTIEHMVVEEVVVEENQLVDEADEILDSKQEFVIKKVFSESDNIAASLNKDIFEPKVETKATCGEVVPRPEMVTEDVYITEGIAATLEKSAVVTKPTTEMIAETKLSDEVVIEPPLKDESDPKQTEVELPESKPAVNIPKSERILSAEVETTSSPLVPPECCTLESVSGPVLLETSLSTEEKSNENVETTPLKTEAAKEDSPPAAPEEEASNSSEEPNFLLEDYESNQEQVAEDEMMKCNNQKGQKQTPLPEMKEPEKPVAETVSKKEKAMENPARSSPAIVDKKVRAGEMEKKVVKSTKGTVPEKKMDSKKSCAAVTPAKQKESGKSAKEAILKKETEKEKSSAKLDSSSPNTLDKKGKDTAQWSPQLQTLPKSSTKPPQESAPSVISKTTSNQPAPKEEQHAAKKGLSDNSPPSVLKAKEKAVSGFVECDAMFKAMDLANAQLRLDEKNKKKLKKVPTKVEAPPKVEPPTAVPVPGQKKSLSGKTSLRRNTVYEDSPNLERNSSPSSDSAQANTSAGKLKPSKVKKKINPRRSTICEAAKDLRSSSSSSTPTREVAASSPVSTSSDSSSKRNGSKRTTSDLDGGSKLDQRRYTICEDRQPETAIPVPLTKRRFSMHPKASANPLHDTLLQTAGKKRGRKEGKESLSRQNSLDSSSSASQGAPKKKALKSAEILSAALLETESSESTSSGSKMSRWDVQTSPELEAANPFGDIAKFIEDGVNLLKRDKVDEDQRKEGQDEVKREADPEEDEFAQRVANMETPATTPTPSPTQSNPEDSASTTTVLKELETGGGVRRSHRIKQKPQGPRASQGRGVASVALAPISMDEQLAELANIEAINEQFLRSEGLNTFQLLKENFYRCARQVSQENAEMQCDCFLTGDEEAQGHLSCGAGCINRMLMIECGPLCSNGARCTNKRFQQHQCWPCRVFRTEKKGCGITAELLIPPGEFIMEYVGEVIDSEEFERRQHLYSKDRNRHYYFMALRGEAVIDATSKGNISRYINHSCDPNAETQKWTVNGELRIGFFSVKPIQPGEEITFDYQYLRYGRDAQRCYCEAANCRGWIGGEPDSDEGEQLDEESDSDAEMDEEELEAEPEEGQPRKSAKAKAKSKLKAKLPLATGRKRKEQTKPKDREYKAGRWLKPSATGSSSSAEKPPKKPKVNKFQAMLEDPDVVEELSLLRRGGLKNQQDTLRFSRCLVRAKLLKTRLALLRVLTHGELPCRRLFLDYHGLRLLHAWISENGNDDQLREALLDTLESLPIPNRTMLSDSRVYQSVQLWSNSLEQQLAVVPQEKQAALHKRMVALLQKWQALPEIFRIPKRERIEQMKEHEREADRQQKHVHASTALEDQRERESSNDRFRQDRFRRDTTSSRIGKPIRMSGNNTICTITTQQKGSNGAPDGMTRNDNRRRSDIGPPSEQRRTLSKELRRSLFERKVALDEAERRVCTEDRLEHELRCEFFGADINTDPKQLPFYQKTDTNEWFNSDDVPVPAPPRTELLTKALLSPDIDVGQGATDVEYKLPPGVDPLPPAWNWQVTSDGDIYYYNLRERISQWEPPSPEQRLQTLLEENTTQQPLHELQIDPAVLENELIQVDTDYVGSLSAKSLAQYIEAKVRERRDLRRSKLVSIRLISPRRDEDRLYNQLESRKYKENKEKIRRRKELYRRRKIEVLPDAVDEIPVPGKALPIQPYLFSSDEEETKVAAIEQPAAEEEQDSLNMAPSTSHAAMAALGKAVAQPTGLGTVGKRKLPMPPSVTVKKHRQEQRSKKVKSSQSPLTATSAREAHEKFRFEISGHVANFLRPYRKESCTLGRITSDEDYKFLVNRLSYHITTKEMRYCEVSGNPLSCTESVKHKSYDFINQYMRQKGPVYKKPAEND

>gi|24666583|ref|NP_649084.1| CG18136 [Drosophila melanogaster]

MASTSLTSCALCQAKASQLCAACRNVVYCSREHQKEHWKKGHRSECQCFEIATNEVLGRHLRATRDIKIGEQILKEAPLVLGPKVASAPLCLGCHRNLLAPGKPRGNYHKCSSCSWPLCGKECEDSVHHKAECQLMSGSNFQSKINYVPGEEERKESAYCVIMLLRCMHLKDKDPDAFLKLYNLEDHLKERLETPLYQVLRANLITFIKTVLGMKDWPEMDILRIAAILDTNTFEVRQPRERRKIRALYPGAAMISHDCVPNMRHRFDDDMNIVFLAKRKIAKGEILSISYTQPLRSTIQRRVHLRQAKCFDCSCARCQDPEELGSFAGAQTCLKCKAGKIISLNPLLNSAPWKCQLCNFKRSAKDVVTSDAELQQELESLDKTTPVALEEFIYRHRADLHETNTHILQAKYALTQLYGSAPGFAMEELSGESLNRKLQLCEELLKLADIFDGGWSIFRGNLLIDMEEALVTQALRSKDPLDCEEKLRNAAEMLREIRNIMKHEPEMQQLLLERQVILSSALERFEPVEN

>gi|24652121|ref|NP_724802.1| CG1868, isoform B [Drosophila melanogaster]

MERLARTFSQQNELITLNENYKDEFETFKSLASMPADILQIFQKLALDLLQDLEQPVDRKGCAERSKNLREEGNLMFKESASGSSKDSVLKACRLYSEAVFEAENAVEELSLAFANRGIALQEYGYYREAYDDCSNALECGYPERLRHKVIMRQAFCAWKLKKIASLEEHLDYLGQLQLKESFEQQLEDLKQKLELLKNQPREQETQGKVVDKSLGEILTDPGPRGRYMVAKEAISKGNVIFSERASCFVPLEQLLICQQCAATLMSAPIPCPNCHQRVVYCSRKCREAHSAIHKFECAAYRKDILRLLGISHLALRLLLTYIPYIRPHLQEMTSAKGMWEEIMNLSRKPEESENAPEYLRSLRMVSQLDQAIDEELNYHILCANLLQLYLKEHTDFYDQFHSLPASIEDWQLIISALILRFAGQLLANGHVGDALLGVGMEPKEFVMLQPELWQKPRHLKRGQLHNLSHSDPITAINLPYLSLCNHACEPSIRTKFDGCSVVNYAAKDILEGEEIFNCYTMDYRNSLKLQRSHPLKAIYKFECTCAKCTRTDPDQNYLSFHRYRCEKPNCRQEFLPDAKVQQNNLRWWLRCNAEKPITCTVCHELQHFAWYNEFLGLIGSSADSSKRQALFKAFDDLDKWLVDHHSLKRIMAEELVTACFYEIDGGTSLDEFEYEDLARIIRKQLEGIAAQCGDNSTEYIARMTYLWDIIAQKKCRTDRKELLEMKGKVDYLASEHREVFLNYYNDFIEQ

>gi|18543183|ref|NP_569834.1| G9a, isoform A [Drosophila melanogaster]

MTDFVELMNSMSSTFNSDCATSTAEGGTLLNLNLAEDKTLKWRNLANNQFASKEKKHKDKEEEERKEARNQEEIEDIKALLADVVDAAAVKLEEEEAQNAEKVEPHTKCEIEEEGRKEMEYDQDVAKQDSEMEKKQNGKATSITVKMESNERAEKHATEIATTSTERWENESFKTEQQNKKAAEKEEEPILAATQKLEANAEPLTTTRIEVAVASPLVVSSASVKLAADATNQMRAATSAGAATLADKNVQVSPGGTRRSRRTPRPIDTPTSVTDEHVQVENKKFGKSEQYTDCSSHLERFTLDDNTAIVRLQLKSEPDKPSLTALSPEENSAPAPKRGRGRARKIRPDAEVETSEVILPCEDSLGEKKPGRKRKLPDEPIDQQQLSDLVVVKTEQEELGDAPLGDVKRMRRSVRLGNRLHADGSPWEEVKTEALHPQPSAELSFAEVTSEILPLAVLDEKTPPKKRGRKAKTPCVKLESETSCGLPFANGNKKTNSSGGCELQLPKRSKRRIKPTPKILENDELRCEFETKHIERMTQWESAAAVDGDFETPTTGGNGSNSSTSRQKSDKSDGSNFEGGPGHPAGTSAIKKRLFSKSQRDIENYGAAMLAKSKLPPCPDVEQFLNDIKASRINANRSPEERKLNKKQQRKLAKQKEKHLKHLGLQKNHRDEPSDNDSSNTDNEFFPTTRVQVGKPSVTLRVRNSVTKELPTTATLKSRRNPVVQAAKLTRRIGARAAGEVTEAARASVPISTPDAEQLHSLDTSIQADVTPIRDLDMRPSTSRVSKFICLCQKPSQYYARNAPDSSYCCAIDHIDDQKIGCCNELSSEVHNLLRPSQRVSYMILCDEHKKRLQSHNCCAGCGIFCTQGKFVLCKQQHFFHPDCAQRFILSTSYEKELGDEEDQGVKFSSPVLVLKCPHCGLDTPERTSTVTMKCQSLPVFLRTQKYKIKPARLTTSSHLTQFGTVENANTPGATARNKGGLSTAVTLSAASSPASKTNGAQRGRAGTSNSNSRHALNSINFAQLIPESVMNVVLRGHVVSASGRVTAEFTPRDMYYAVQNDDLERVAEILAADFNVLTPIREYLNGTCLHLVAHSGTLQMAYLLLCKGASSPDFVNIVDYELRTALMCAVMNEKCDMLNLFLQCGADVAIKGPDGKTSLHIAAQLGNLEATQLIVDSYRTSRNITSFLSFIDAQDEGGWTAMVWAAELGHTDIVSFLLNQDADPNICDNDNNTVLHWSTLHNDGLDTITVLLQSGADCNVQNVEGDTPLHIACRHSVTRMCIALIANGADLMIKNKAEQLPFDCIPNEESECGRTVGFNMQMRSFRPLGLRTFVVCADASNGREARPIQVVRNELAMSENEDEADSLMWPDFRYVTQCIIQQNSVQIDRRVSQMRICSCLDSCSSDRCQCNGASSQNWYTAESRLNADFNYEDPAVIFECNDVCGCNQLSCKNRVVQNGTRTPLQIVECEDQAKGWGVRALANVPKGTFVGSYTGEILTAMEADRRTDDSYYFDLDNGHCIDANYYGNVTRFFNHSCEPNVLPVRVFYEHQDYRFPKIAFFSCRDIDAGEEICFDYGEKFWRVEHRSCVGCRCLTTTCKYASQSSSTNASPTNATTAPENETGTLSSTNTEKIGHA

>gi|24762738|ref|NP_726483.1| CG30426-PA [Drosophila melanogaster]

MHCALYNSCPVAHKHLPTLDIEPSDYVHEVPPPGEIVRPPIQLGETYYAVKNKAIASWVSIKVIEFTESTAINGNTMKSYKIRYLNTPYQMIKTVTAKHIAYFEPPPVRLTIGTRVIAYFDGTTLSRGKDKGVVQSAFYPGIIAEPLKQANRYRYLIFYDDGYTQYVPHRDVRLVCQASEKVWEDVHAASRDFIQKYVEKYSVDRPMVQCTRGQSMTTESNGTWLYARVIDIDCSLVLMQFEGDKNHTEWIYRGSLRLGPVFRETQNNMNSSSAQQLRVPRRTEPFIRYTKEMESSSKVNQQMRAFARKSSASAQNNALAAASSAATPAGGRTNAGGVSTSNSASAVRHLNNSTIYVDDENRPKGHVVYFTAKRNLPPKMYKCHECSPNCLFKIVHRLDSYSPLAKPLLSGWERLVMRQKTKKSVVYKGPCGKSLRSLAEVHRYLRATENVLNVDNFDFTPDLKCLAEYSIDPSIVKDTDISKGQEKMAIPLVNYYDNTLPPPCTYAKQRIPTEGVHLNLDEEFLLCCDCEDDCSDKSKCACWQLTVAGVRYCNPKKPIEEIGYQYKRLHEHVPTGIYECNSRCKCKKNCLNRVVQFSLEMKLQVFKTSNRGWGLRCVNDIPKGAFICIYAGHLLTETMANEGGQDAGDEYFADLDYIEVAEQLKEGYESEVDHSDPDAEEDNGGPDAEDDDDFRPNYHYQRKIKRSSRSGSTQNSSTQSSELDSQERAVINFNPNADLDETVRENSVRRLFGKDEAPYIMDAKTTGNLGRYFNHSCSPNLFVQNVFVDTHDLRFPWVAFFSAAHIRSGTELTWNYNYEVGVVPGKVLYCQCGAPNCRLRLL

>gi|24648668|ref|NP_650955.1| CG3353 [Drosophila melanogaster]

MNNFEIRELPGKGRAMIATKNFAKDEVIFEEEPFVSRQFSWNVAYGYAACDHCMRPLETVLENVRRLASDPKVEVPLLQHDPTAQWVAQFTQCPRCKVRYCSEDCLMEAQKRYHRVACMGAFHSDDTHPINVLNETWKKMHYPPETGSIMLIVRLMALYQQSTKKEEFLEQLQSFQSLIVNREQKIYHKMLGENFEQQMEQLYLAFCNAFTGEEFSIFKTPDAFKTLMAILGTNSQGIATSVLSQWVAKVSDLPLTDSEKEQLDTVIDGLYAKVGEFAGEFLNNEGSGLYLLQSKINHSCVPNACSTFPYSNDIVVLKALAPIQQGEEICISYLDECMLERSRHSRHKVLRENYVFICQCPKCRAQASDPDETSEDDDDDDEMDDYDDDDDMN

>gi|51951109|gb|EAL24598.1| Set1, isoform A [Drosophila melanogaster]

MQDVRNINLVNNSSNSHDSSLANSKMPRNFKLLSDPQLVKCGTRLYRYDGLMPGDPSYPTITPRDPRNPLIRIRARAVEPLMLLIPRFVIDSDYVGQPPAVEVTIVNLNDNIDKQFLASMLDKCGTSDEINIYHHPITNKHLGIARIVFDSTKGARQFVEKYNQKSVMGKILDVFCDPFGATLKKSLESLTNSVAGKQLIGPKVTPQWTFQQAALEDTEFIHGYPEKNGEHIKDIYTTQTNHEIPNRSRDRNWNRDKERERDRHFKERSRHSSERSYDRDRGMRENVGTSIRRRRTFYRRRSSDISPEDSRDILIMTRERSRDSDSRPRDYCRSRERESFRDRKRSHEKGRDQPREKREHYYNSSKDREYRGRDRDRSAEIDQRDRGSLKYCSRYSLHEYIETDVRRSSNTISSYYSASSLPIASHGFNSCSFPSIENIKTWSDRRAWTAFQPDFHPVQPPPPPPEEIDNWDEEEHDKNSIVPTHYGCMAKLQPPVPSNVNFATKLQSVTQPNSDPGTVDLDTRIALIFKGKTFGNAPPFLQMDSSDSETDQGKPEVFSDVNSDSNNSENKKRSCEKNNKVLHQPNEASDISSDEELIGKKDKSKLSLICEKEVNDDNMSLSSLSSQEDPIQTKEGAEYKSIMSSYMYSHSNQNPFYYHASGYGHYLSGIPSESASRLFSNGAYVHSEYLKAVASFNFDSFSKPYDYNKGALSDQNDGIRQKVKQVIGYIVEELKQILKRDVNKRMIEITAFKHFETWWDEHTSKARSKPLFEKADSTVNTPLNCIKDTSYNEKNPDINLLINAHREVADFQSYSSIGLRAAMPKLPSFRRIRKHPSPIPTKRNFLERDLSDQEEMVQRSDSDKEDSNVEISDTARSKIKGPVPIQESDSKSHTSGLNSKRKGSASSFFSSSSSSTSSEAEYEAIDCVEKARTSEEDSPRGYGQRNLNQRTTTIRNRNLVGTMDVINVRNLCSGSNEFKKENVTKRTKKNIYSDTDEDNDRTLFPALKEKNISTILSDLEEISKDSCIGLDENGIEPTILRKIPNTPKLNEECRRSLTPVPPPGYNEEEIKKKVDCKQKPSFEYDRIYSDSEEEKEYQERRKRNTEYMAQMEREFLEEQEKRIEKSLDKNLQSPNNIVKNNNSPRNKNDETRKTAISQTRSCFESASKVDTTLVNIISVENDINEFGPHEEGDVLTNGCNKMYTNSKGKTKRTQSPVYSEGGSSQASQASQVALEHCYSLPPHSVSLGDYPSGKVNETKNILKREAENIAIVSQMTRTGPGRPRKDPICIQKKKRDLAPRMSNVKSKMTPNGDEWPDLAHKNVHFVPCDMYKTRDQNEEMVILYTFLTKGIDAEDINFIKMSYLDHLHKEPYAMFLNNTHWVDHCTTDRAFWPPPSKKRRKDDELIRHKTGCARTEGFYKLDVREKAKHKYHYAKANTEDSFNEDRSDEPTALTNHHHNKLISKMQGISREARSNQRRLLTAFGSMGESELLKFNQLKFRKKQLKFAKSAIHDWGLFAMEPIAADEMVIEYVGQMIRPVVADLRETKYEAIGIGSSYLFRIDMETIIDATKCGNLARFINHSCNPNCYAKVITIESEKKIVIYSKQPIGINEEITYDYKFPLEDEKIPCLCGAQGCRGTLN

>gi|24645790|ref|NP_650024.1| CG4565-PA [Drosophila melanogaster]

MDESETAPNDDYEHPDGLDYILESVLMPSDGSKEFKFLADEYNSVLLNPCHCKGACENSEVCAHGGQYEFTEDGSELILRNSANPVIECNDMCKCCRNTCSNRLVYSGPRKHLEIFDSPVYGSKGLRTTAKITKGGYICEYAGELLTVPEARSRLHDNEKLGLMNYILVLNEYTSDKKQQVTIVDPSRRGNIGRYLNHSCEPNCHIAAVRIDCPIPKIGKFYC

>gi|19922072|ref|NP_610730.1| CG8378 [Drosophila melanogaster]

MDVYDVSDDLIKKLQDWKLIGIISGKFNELKENHRKVDFVMRALIDFKYIEKIFLNVTLREDKCNKRSVEFRMLGNEQFSLKNRNYFQALELYNKSICYAEPNSEHLSIGYANRSAVLFEWKRYRQCLDNIKLARQANYPARLSHKLDKRERDCQQLLDQQPPDVVPYEFKLSFEPHAQVPFIADCLELRETAAEGRFVVTNRDLAVGDLVSVEEPFCSTLLTPMRYIRCATCKRENYLTLIPCDSCCSTMFCSEECKSIAMQTYHRYECPIIDFLNRMFNKIHCIALRTTLVALNIFPSIEELIDFCEQEQNQDKCAFDLNYNELTPEEHYRAIHGLVTNQHLRSVSDLFQRSVVCAVLKHFIIEYTPVKEYLGGEEGVNFFTDLLFRHLQTSPSNMHGIDLVEQVNETKDDQTHSSGAYAFLSLINHSCAPNTVRIYEGTKAYMFVLRPIKAGNVLYDNYGAHFAICSKEQRLKRLSLQYRFDCKCEGCELNYPMFGMMPHKATVPSVTDDTELALSSYNYDFAVSNYRKYCDFLTQYGDDYPCEQISSAEECLKMALHIMADAVPLKAKM

>gi|19922236|ref|NP_610944.1| CG8503 [Drosophila melanogaster]

MNPCHVCEEPTKNKCSNCNQVSYCSVQHQKQDWKVHKPSCHPFKIAHNEQLGRHLVATRTIKPYEIVLKEAPLVRGPAQISAPVCLGCLNGIEAEDHIECEQCGWPLCGPECKSLDEHKAECGLTKDRGQKVNVQEFGGPHPLYTCLSTVRCLLIGETSTEKASKFQDLESLESTRRGSNQWKADLVSIGQFIPKFFKTQKFTEEEIMKAVGALQINGHEVPTTDPSHVAVFYTASFTENSCLPNLAKSFNKNGHCILWAPREIKKNAHLSICYSDAMWGTADRQRHLMQTKLFKCACERCVDVTELDTNYSAIKCEDRQCGGLMLPTKADEWNGSWRCRECHKQVQKHYVERILERAGKDIQSMEKIAENGLKYLKHYEKWLPPQHFHMSEIKILLVQLLAKDQKELMVIPDDRLLLKLNFARELVELYEKLTPCEVRTLGTLCFELHSAIAEQTRRVALETSLSPKDRLEESLFYVDKCVNYLKYESDIFIEGHVLKQAKINRDALRMVMS

>gi|24664023|ref|NP_648681.1| CG9007, isoform A [Drosophila melanogaster]

MPMSSHDSVFVEPAVSSSGGGSTSSINRIGVASGPQPPPPHIVIDGSSLSTQAQLRIMQRRMSISTRQILSASQSQSINQSQKYAVRTAAGTGALAAGAASSGSSGQELINPQIYKIVTTDGSTSNVIIDASAAAPPHNSKLLLSNLTVLSKQAPVQGAAGQQQQQLNYLGAKSLPYVSASPAKTQQQPQKFGSISAFKAQHQQQQQHPHATLQKVTLGSRVATRLQSYVPASPSQIMVQPAQPKYVNATATTIGGNAALLKKANQKITVKSVSNMSQQQQQQQQQQQQQLLQAQQLKTFITQQQQQQTYKAGAKAKFVKQTTALSIAALPQQQQQTTYAKQSMVTTSTPAKVTKLNSKYVQQQISLPQGTQLQHKASPNVTGGGYLLQQSQAAVGGTIKFVNSHGTVIQQHPQQQQATKRTHYNSSGSSDNEQHAVANSSLITDDVMIVNGTQMTDELSARILQSMAQKSFSQQQRFHQVPATGSGNMPPPTQIIYSNSTSNGAAASSPGGNASGNMLLAHYQAAGTKPVSSASFITVTGTPPVTVATTPSVSISSHGFASGSAAISSYMSSATAARRQSVSAPSSRAVSLERKQHHQQLQHDVIGGGRKAPTVIEYYNKHGVNSIVGSSNNLAQSNSMSNLAGPRSNSGSGFATTTPTPATPLHLTPVNVPVHVEAAPPSSPALVKGSSQPPAQPQQQQQQAHPLGPNQLNANDEELYIEEVRPVPVLTQDLRLQQLHAIMQDHTYASQQQQQQPQQAAGDTTNPGAAQQVQQPQQWSLGGIGVTVSGSQGTPTAVGGYCSYFGQQIARSQADDDAHSAISSSSRMGLASTDIDPGEETETAPEAEAEDDSVTRCICELTHDDGYMICCDKCSAWQHVDCMGIDRQNIPEEYMCELCQPRAVDKARARALQRQKRKEHMLLVATQAANGAAAVAAGTTLSGGLGSGLPMSEELQHRLASGLNGGFATGTGMSKKSKKTKENSGSTSTLKKTKKSAVGMGGEKNASGSGTPTGSSGKTSKKSSKRKSKSGGDGSSGGGSSPALTAAEKHAANLRQWIENYEYAVTNHYSPELRARLHAIQKQPSLLQSIQNTENKALRQIQQQLSTAGSAEQLEQRAQLIPYAGAKVLISSVDLSPHAPIHELRGKYMLTTQFRTQNPTVNMNTPPPSNYLNSFKAHKTPGQFVFFYQLPGVEAPMQTLRPDGSVPQVAQQPPSYLKGPEVCVDTRTYGNDARFVRRSCRPNAELQHYFEKGTLHLYIVALTHIRAQTEITIRHEPHDLTAVEQKKSHAAVIQPTSTRCACDMGSDCLFALPLAVQQQLQAPPTQPRSSHRNKAAAAAAAAAAANSAAAIQLTMGLGVGATVAAGASVLPNSRNRSTSSSGESSQMGLNSPQLGQLNLGFKTSVTATSLTAPVPGVHCNNSGGSSSSSNNSCSVSMSSVLHDSGICTSSSSPSVSIPSPTPTQMQSPTLQQHPQQIPQQQLSLLQRSPTQQHQQQILAALPTPMLTPMLSPQLPKPAQQQAHVVLPQSQQTSLLQQQQSQQSQEPLAVIAAAAAAQQPMATYFVRQPQQQQQQQSPKPQALVAQQQHVVGAQQQQHFLQQQQKQQQQQMADEARMAVSALQTLHAAPTSHIVSPIKVAAVQQQSQPQQQQQNTHQQPHNQQAVQQQSNQLQQQQSQQPNYPQSPQRQQKPQPVQHQPQIVISTGAQAIPATMPTKLSSPTKSAAPVISNNNITVSAQSSVVGGKKTPAKHPQQQQQQQQQPVTPVSAATAPAATPSSSESKEDDVSASSTTTPTTRTPAKDKPKQSREDRKLEAILRAIEKMEKQEARGKKDTRQSSGGKRQASNSPASPNKRNSSNSISEDVETPTSTNSAAAAAQRRNKKKRKVSRSLNNNTNGLGSGGGSNNKRRKSIVVESDGESHALTNSESEDQGQHPQSHHSGSEDQAAGLLLALAHNNSSPNEPFKSPLSQSHSLPATPASVSSACLLIEAAMGPLQQQPAPASASPSLAEFKYPPGGAKTKKSLMSSWFQQAEQQHASGLDSLVQAAMSEINGEREQLQRQPQGESLPAPALLKVEQFIHQAESTTAVPAREQLHLPLQNNSSVKKRWLRQAISEETTPVDELQQSQNQSVTATPSPQPVPTVSPLANGFSTPLKKRRLVVVSNGTNVESDETHIDVIGEPKDEAEENVAMTELKVEIENHHQEQDDDVDILRSPSPGTHQIVAEDNLVKIEPEDTSAAADDVKIDVEREESQACDKFEEMVKVKREEEEQREKEIKQLQERQEHEQPKVEPAPVEPKLENTVAKAEPKVEPSQEIVSKKEPTKVEPKPGESLLRSTATVTATPTAATIAATTLLDVSKVAFKTRPPLKLEDEPQKKKPKLESILPAPVATVPPVSVPPIPAASNATTSAVTNTAAASLTTTTAPSSTKNLTEHDIQERLLSFHAANISYLQSRNKKATAALTSASPSQKSNSSSGGSGTESKKSSKDKDEKRDKEKQLKKSKKEKKKSKDKEKQKAAVNVNSTSQIVDTKKKTTQPSKPDSKSSIAPVLVPPSLPVATANGKTKHTAYNNVDQQQQQQMRRRTMSMCITPVTPTPVVTPSPLHGTPPSTKKRQTNFEQELTKPNSQILSSSILLNSSKGLGLPLAAPTVVSVPTAVQQQQHRKENNHQEATPASGGPMSLAAAIASGKLNAISRRRESMCGSRQQQALIAAALKKEKKEKKKSKKKDREKQKHDKQKGKEKEREKDKEKDNKQKTNHIQKPAHPTTVPANSMPISAPAPVPVLVPTPVTTPKAAPIPVLITQPTPSPIHVTQPLVNNCSTKVASLPFYNTIYGKLQDPSTPTSSPIPVTNTMPSLAEYLESTKSKTTALSAVKPIGIAAVTPLSSAITPAKVAPSSLEMPPPATTPLKLYTRTASHDPRLNPMLTVPDPTPMPKRKLSISEYRMRHRPSVDTAPTTPTTPTTPTTPTTPPGSNKDRSFAKPQTINKCSLQSPERFQAAIRERRNSISAHHPQQNAHHNLNNNKGGISNKSGSSSGNHLQQALGVAGGRLQSKNAIVDPAATTLSTVNSILSTAQKLHMFDDKPKGGHFNAAPTLLEQQQEKMSERSRCLQRTISCDSRVIERLGTGAAAGALEKVTTASTRRDAV

>gi|24654325|ref|NP_611181.1| CG9640, isoform A [Drosophila melanogaster]

MQEFGENIQHAHDEKLGRHLVASIAIEPGDTILEERPLLVAPHWECHQLKCAQCLQESYVICRRCQVFPLCMDCNQHDEFECEFFTSGAGKALCKDILVKNFGICGLLKLLLLLENPRTKGDCQMLIDVPINLSDYRDGEGMWQEHEELVVRPLMESGLADVLPTQELTSDALHAHCIRIDSNSFEVTAKDGDTLKGIFVWGATLPHHCVPNTVVALDEQFNMKLYAAVPLQPGDIIYNSYTNPLMGTSQRQHQLRLSRRLECICSRCLDPTEMGTHMSSLKCKECPGFSVCEIDSNGKLGDWRCPDCRALLTAAEVHELQAAVGSALVDAMGDLQVYEALLTQYGPLLHPNHFMLLDIKQNIASILRAAALMNSMDQPCKKLLARRVELCSDLLPVCRAVVPGISKLYAIGLFEYLLALVELVELQFAESDLSKKEYVAHLRTASLVATEAMDLLRFEPENSAEGYLADRISMELERIESDLKKYGR

>gi|24654327|ref|NP_611182.1| CG9642 [Drosophila melanogaster]

MAAVTDDFARKCEIKQNDTLGRFAVALCNVRAGETLLLENPIVVLPLMGERRCSKCFNLTESFCRKCRLLALCEDCSDHDERDCKRLAEMNFSDDQVELLQKKEHTEIQPVLKCLLLREHEETLPLYEEMSQMDSQLMTRRGTEVWKNYQEHAFTPLDYGGVLAQLRGAADEDLVQGLLGILDINAYEIRAPEVGGAMRGLYRRAGLFAHSCTPNLVISIDDEQRIKVYANRFIAAGEILYNCYTNVLLGTEERRKILKVGKCFDCSCPRCQDPTELGTHMSSFICSQCSCVDGYIVRQPDTGIWQCLLNPEHTLKQEFVSNMLERAKEEIFHARDDIYRQELLLAKLSRLLHRNHFLMLDLKQNIASILRQILQNMGTRPNKKVYERKIRLCQEILLVLKVVTPGISRLKAIALYELANTQAELARKMYTEMEHSANDLLAELERVEVMLRESLRMLLFEPLATPEGQLTRSMLRELKELQDDIKNLRESDDDVVNQ

>gi|24662251|ref|NP_524021.2| enhancer of zeste, isoform A [Drosophila melanogaster]

MNSTKVPPEWKRRVKSEYIKIRQQKRYKRADEIKEAWIRNWDEHNHNVQDLYCESKVWQAKPYDPPHVDCVKRAEVTSYNGIPSGPQKVPICVINAVTPIPTMYTWAPTQQNFMVEDETVLHNIPYMGDEVLDKDGKFIEELIKNYDGKVHGDKDPSFMDDAIFVELVHALMRSYSKELEEAAPGTATAIKTETLAKSKQGEDDGVVDVDADGESPMKLEKTDSKGDLTEVEKKETEEPLETEDADVKPDVEEVKDKLPFPAPIIFQAISANFPDKGTAQELKEKYIELTEHQDPERPQECTPNIDGIKAESVSRERTMHSFHTLFCRRCFKYDCFLHRLQGHAGPNLQKRRYPELKPFAEPCSNSCYMLIDGMKEKLAADSKTPPIDSCNEASSEDSNDSNSQFSNKDFNHENSKDNGLTVNSAAVAEINSIMAGMMNITSTQCVWTGADQALYRVLHKVYLKNYCAIAHNMLTKTCRQVYEFAQKEDAEFSFEDLRQDFTPPRKKKKKQRLWSLHCRKIQLKKDSSSNHVYNYTPCDHPGHPCDMNCSCIQTQNFCEKFCNCSSDCQNRFPGCRCKAQCNTKQCPCYLAVRECDPDLCQACGADQFKLTKITCKNVCVQRGLHKHLLMAPSDIAGWGIFLKEGAQKNEFISEYCGEIISQDEADRRGKVYDKYMCSFLFNLNNDFVVDATRKGNKIRFANHSINPNCYAKVMMVTGDHRIGIFAKRAIQPGEELFFDYRYGPTEQLKFVGIEREMEIV

>gi|24650756|ref|NP_733239.1| Mes-4, isoform A [Drosophila melanogaster]

MKLSTDAHSEIEGDAAHGNVLCNSASDSLTATDEVAAGNDESVATEGDDVEIPRDTNNSTPVRLLDKPGQNPVQNGAQPAAEESELESQRQTPVQKQQQQRVSMVNRKRDLINLQSALSPKYIGYANANSPTPLSDSDDTIRTTRRRVNQAAALNNSSAGETLAHDNASPRTPGGGGGGGGDDSANQLLSKTYMSPIEKLLIKNGASSPNSTGFEAGSEDLGIRPIVRKHVKRKMKRVPKAKVTLELDEKNQQEVDEKSVKTEPIDEEVDRTDEAPTQEAQTTAISIKSETEAEHKAAVDVHIKQEDTIRLDIVNNPVESTSIVITEEPKDLEKSTEELAFALPLASSTEVDLKSPPDLSSTALATSIKSPSSVSIDSAKGLSIVTDPGWPTYQVGDLFWGKVFSYCFWPCMVCPDPLGQIVGNMPSHPQRSSLDNANVPIQVHVRFFADNGRRNWIKPENLLTFAGLKAFDDMREELRIKHGPKSAKYRQMVPKRTKVVIWRQAIEEAQAMTQIPYSDRLEKFYQTYENVVTLNRQKRKRTKYMMQDTSDVGSSLYDSTDNLHNKQGTQLLAVKRERSESPFSPAFSPVKSKNEKRAKRRKLSNGTEADTGSNSMAVTPSQTETTVDSSAYENPEFRQLLSAVMEYVMMNRSDEKVEKVLLSVVSNIWSLKQIQLRELERDLASGEIEEPLGSSVVGRGSGVGTIKRLSNRLMTMMVRRSMTPVVTPSTTPAPSEPDRRLSEPPKTKKPVNRPIEEVIEDILQLDSKYLFRGLSREPICKYCYQAGSDLVRCSRTCSSWLHADCLERKVTGAPMPKIGSRKALVIPPTSKSPSPDEDHVTADAKEVVAVGTSLVCHECNVGEPEGCVICHQVESPAVPSTPRKEDSSSHTPIEDKLLTCSQPMCGKRFHTSCCKYWPQASSSKHSARCPRHVCHTCVSDDPSGKFQQLGSSKLAKCVRCPATYHQLSKCIPAGTQMLNTTNIICPRHNIAKADAHVNVLWCYICVKGGELVCCETCPIAVHAHCRNIPIKTNESYICEECESGRLPLYGEIVWAKFNNFRWWPAIILPPTEVPSNILKKAHGENDFVVRFFGTHDHGWISRRRVYLYIEGDTGDGHKTKSQLFRNYTTGVEEASRFLPIIKARRQEQDMERQSGNKLHPPPYVKIKTNKAVPPLRFSQNLEDLSTCNCLPVDEHPCGPEAGCLNRMLFNECNPEYCKAGSLCENRMFEQRKSPRLEVVYMNERGFGLVNREPIAVGDFVIEYVGEVINHAEFQRRMEQKQRDRDENYYFLGVEKDFIIDAGPKGNLARFMNHSCEPNCETQKWTVNCIHRVGIFAIKDIPVNSELTFNYLWDDLMNNSKKACFCGAKRCSGEIGGKLKDDAVKAHAKLKQMRRAKASAVRIHVKPKKTPKVKHISADDEPMDAKDE

>gi|62473423|ref|NP_001014717.1| msta, isoform A [Drosophila melanogaster]

MSTAAQCPPPRPGGGGGDTTLAALSAHMAPCRDTTPEQLAQLIDVHLGDLRQEQPNWTISSSTVAGRGVFATRDIAAGELIFQERALVTGPTARKGQLSSCICCHETLPQTGFLCRHRCTLPVCETCSDSEEHQAECEHFRRWQPKDVDAEQEQVNPMSLRILTAVRVFHLGKEQRHLVDAMQANAERAYRREIIQAAQCFRNFPTTDRVFMDQLFRIVGVLNTNAFEAPCRSGGHETLLRGLFPLTAIMNHECTPNASHYFENGRLAVVRAARDIPKGGEITTTYTKILWGNLTRNIFLKMTKHFACDCVRCHDNTENGTYLSALFCREQGCRGLVIPVQTRTLQPDWRCITCENVFPHAKMAKYQDFALNTINNRINSCSVQDMIHFINELCPRFCPSSNYVLIEAKLNVIWRMTRFDHEEYTPEEMGHMDRYREEVLAILHKLGAGECTLKKLITGEIQ

>gi|62473432|ref|NP_001014718.1| msta, isoform B [Drosophila melanogaster]

MSTSTPVTEEPRSSSQMTISASTQELAELINIHLGDLRPEEPSWRVADSPISGRGIFATREIAAGEELFREHTLLVGPTAHRSMNLRTCTLCYRLIPGSTDSAALCPAGCGLPVCSECRDSTRHDLECKLFRKWKPLESQRIEPRALRILSVVRCFFLDEASRKLLYAMQANMDRYYMQEVQRAADCFEHFPREQDMLDYFYRTICAFNTNAFESRSNVDGHEVLVRALFPLAGLLNHQCTPNAAHHFENGETIVVCATERIPAGAEITMSYAKLLWSTLARKIFLGMTKHFICKCVRCQDPTENGTYLSALFCREQGCRGLVIPVQTRTLQPDWRCITCENVFPHAKMAKYQDFALNTINNRINSCSVQDMIHFINELCPRFCPSSNYVLIEAKLNVIWRMTRFDHEEYTPEEMGHMDRYREEVLAILHKLGAGECTLKKLITGEIQ

>gi|24646810|ref|NP_731901.1| pr-set7, isoform C [Drosophila melanogaster]

MIMVRRRQRPAKEAASSSSGGASSGSGIPVDQALPLNVAGNLLEDQYFASPKRKDCRLMKVTQNGQLPEATMMAHNKDNKAGRTIGVPLATRSQTRTIENFFKANAAAKDSQKTIHTEEQLNLGNQELKLDDEELNGQIKLDDEVLKLADKQINENLPFADEVDAKAEQKLMDEELQQVVEELLFDGSSRASSNSPFYQHDMDVMQEIQQTPEIPHIKKVTEPLEGLGSLADFQTHRSALRDSHSSTHSSSTDNIFLQEPVLTLDIDRTPTKASSIKINRSFELAGAVFSSPPSVLNACLNGRFNQIVSLNGQKEALDLPHFDLDQHDSSSCDSGVACGLTANTESPAGQPRRRKPATPHRILCPSPIKTALKVTGGICKVGSADPLSPRKSPRKLPTTTAAVAACKSRRRLNQPKPQAPYQPQLQKPPSQQQQQQQDDIVVVLDDDDDEGDDEDDVRALIKAAEERENQNKAPATANSNKAGMKTMLKPAPVKSKTKSKGPTKGQPPLPLAATNGNREMTDFFPVRRSVRKTKTAVKEEWMRGLEQAVLEERCDGLQVRHFMGKGRGVVADRPFKRNEFVVEYVGDLISIGEAAEREKRYALDENAGCYMYYFKHKSQQYCIDATVDTGKLGRLINHSRAGNLMTKVVLIKQRPHLVLLAKDDIEPGEELTYDYGDRSKESLLHHPWLAF

>gi|24647050|ref|NP_524357.2| suppressor of variegation 3-9 [Drosophila melanogaster]

MATAEAQIGVNRNLQKQDLSNLDVSKLTPLSPEVISRQATINIGTIGHVAHGKSTVVKAISGVQTVRFKNELERNITIKLERLSEKKIKNLLTSKQQRQQYEIKQRSMLRHLAELRRHSRFRRLCTKPASSSMPASTSSVDRRTTRRSTSQTSLSPSNSSGYGSVFGCEEHDVDKIPSLNGFAKLKRRRSSCVGAPTPNSKRSKNNMGVIAKRPPKGEYVVERIECVEMDQYQPVFFVKWLGYHDSENTWESLANVADCAEMEKFVERHQQLYEIYIAKITTELEKQLEALPLMENITVAEVDAYEPLNLQIDLILLAQYRAAGSRSQREPQKIGERALKSMQIKRAQFVRRKQLADLALFEKRMNHVEKPSPPIRVENNIDLDTIDSNFMYIHDNIIGKDVPKPEAGIVGCKCTEDTEECTASTKCCARFAGELFAYERSTRRLRLRPGSAIYECNSRCSCDSSCSNRLVQHGRQVPLVLFKTANGSGWGVRAATALRKGEFVCEYIGEIITSDEANERGKAYDDNGRTYLFDLDYNTAQDSEYTIDAANYGNISHFINHSCDPNLAVFPCWIEHLNVALPHLVFFTLRPIKAGEELSFDYIRADNEDVPYENLSTAVRVECRCGADNCRKVLF

>gi|20128811|ref|NP_569853.1| histone methyltransferase 4-20, isoform A [Drosophila melanogaster]

MVVGSNHTRRGETGSRFTNSSSSSSTSGGPTASASSTTSVTSSLATNSTSTSTAAALLSSMSHKSHGPPPSAPPSAHHQTNQQHHQVAHSQPHATHYQQTNQHPSHHHQSHQSSNGSGGGSAGSGSGSGSVVSGLNGCNGSAVSRLSQSTGMSPRELSENDDLATSLILDPHLGFQTHKMNIRFRPLKVDTQQLKAIVDDFIHTQNYDIAIQRIYEGPWIPRHLKNKNKIATKRLHDHIVRYLRVFDKDSGFAIEACYRYTLEEQRGAKISSTKRWSKNDKIECLVGCIAELTEAEEAALLHSGKNDFSVMYSCRKNCAQLWLGPAAYINHDCRANCKFLATGRDTACVKVLRDIEVGEEITCFYGEDFFGDSNRYCECETCERRGTGAFAGKDDGLMLGLSMGLGLASSGPGNNGGYRLRETDNRINRIKSRANSTNSTSNSNSNTNDSTGPSETSSTNGLVASGGAGGATGAAMLPTPSQQSTGGKEATAAVSLLEKKLPNVVVSPLTMKELRQKGMTKYDAEMIMANAAYQQQHHHQHHFHHHHHHHHHHHNHGQHASTGAEATAAVQQMAAMQKPGVGGTGAAGNAGATTVSSVAAGAGSEVNGGRSTSLRKSMRVNSTSSSISTASADEVIAPVVAASISLPSKAPVVLMPRCKPAQMAIAALHQSQQRQLRRSERQKEKLTDGESSDTSSEQQKKEQKQQDHQLPQKMFSLAEEPQPEKSEEKQQEQQKRVTRNSAGRVGLVARLATAHNNNIATTTNSSSSSNKATTITNCNNHNSNNSSRINHNSNLSSRLSVKSRKPAPSEASSIPSSTSSENQQQQATRRSCSPTPAYKKNLLASFDPDPPSTQGIKEQLKDESVTYSPVKQKRSRRAAALAAAQSIHCEALGGFPTGSTGSQRKRAQAGEPTTSCSSTTISNVEPLLKTPERRLKLTLRMKRSPILDEVIELGTSLSNGGAGRGAPGSHREGTAGEGSVRSALNLTGSSSNGIEYEILRMEGISEHGNDDDEDEEEDDEEPAAEEEEEPPPKEELQLVNKKQRKKQRSRSRSSQRRSPAPSSVYGTPQKKRLRLIFGNESHTIDIPPAAAEGSGSGLDDLNSSGGGGDESFNASYASSTSLTVNTSSSSTSSSSGVGGATSTSAEPVDSSTVGPPIAAQSPSSTTSSSFQSACTSTTNSNSYFPNGKQRAAGEDSYAMHYYQLGKFAGTPSPGQGQAIVSSSSGSSGGGGSGAGFLSMPKHTFGTCALLAPTSFACLQNQPQISQQKTSSGGGAGVVPTSTSTGAVTSHHHTNNHHGQK

>gi|28571451|ref|NP_726773.2| trithorax-related, isoform D [Drosophila melanogaster]

MNIPKVTTSLGAAEKAKPERVASVAAAAFNAVSLQKRSGDDTATPAEDPTRKKAKTELLLGTGTAAPSLPAKASSTAPQQLLYQRSGQQAKAQVKAASEPQDVETADGVWDARDQQIIVCNFGSGTEMGAIKAEDADKQSEYRISTPRNSQSNPLLHRNTAFTSFTKKEGASSSASSSSSTASVISIEPSGSGQDHAENSGKSEDLDYVLMPASGADSSTSVGNSTGTGTPAGTPIGATTSTIILNANNGTAGVSGAGTTTILTQKSGHTNYNIFNTTATGSQTPTTTLLNRVNLHPKMKTQLMVNAKKLSEVTQTTAKVSIGNKTISVPLLKPLMSASGAATAGGATIVESKQLLQPGGQVTTVMSAAQQSGGQQVHPHVHSHAHHNFTKLIKRGPKNSGTIVSFSGLQIKPANTKIVATKVVSKKMLQLQQHQQQIQQQQQLQQLQVTSGGGLAPPTGSIVTITTTNPSQTYAMVQDSATVGPAAHSEDDAPAPRKITAYSENLQKILNKSKSQESTGGPEEFTNINSVVIKPLDKNTLNCPPSFNIFKQQQHSQAAQSQSISAVGSGAGTPVTFTMASGNASDLATTSTVSVSAGTICINSPMMGTRPIISIQNKNISLVLSKTTMAQQKPKMITTTTLSSQAALQMHHALIQDSSADKAGSSANSGSATSGASMQLKLTTANTPTKLSVSLAPDVVKLEEVGSESKAKLLVKQEAVVKDSTGTPTSEERAEEIGTPEKRLNANATMTAINQVQNQSANQIQMATSTSTASNPSTPNPTVNATPMNNQRSAAEDNALLKQLLQNNSSSHSLNQISITSAHVGSASASAPLSARKVINVRAPSMGKVRSLEDQLARPVIPPVPTATQAAGSSSSSGSVATSTTTTTVASGGSSQQVATASATALPVSAVAITTPGVGGEAKLEQKSDQPAAIMQNQSQNQAPPPPPPPQQQQQQQLHQPQQLQPSPHQVKQTVQIVSKETSFISGPVAAKTLVTEATSKPAELLPPPPYEMATAPISNVTISISTKQAAPKELQMKPKAVAMSLPMEQGDESLPEQAEPPLHSEQGATAAGVAPHSGGPLVSAQWTNNHLEGGVATTKIPFKPGEPQKRKLPMHPQLDEKQIQQQAEIPISTSLPTTPTGQGTPDKVQLISAIATYVKKSGVPNEAQPIQNQSQGQVQMQAQMQATMQGHLSGQMSGQISGHAAGQIPAQMHLQVQHQLHMAVHPQQQQQQLHQNQPQNATIPLPVTGQGAVPIPVPTMESKAGDQRKRRKREVQKPRRTNLNAGQAGGALKDLTGPLPAGAMVQLAGMPPGTQYIQGAASGTGHVITSTGQGVTLGGVGASTGASSSPMLKKRVRKFSKVEEDHDAFTEKLLTHIRQMQPLQVLEPHLNRNFHFLIGSNETSGGGSPASMSSAASAGSSSAGGGKLKGGSRGWPLSRHLEGLEDCDGTVLGRYGRVNLPGIPSLYDSERFGGSRGLVGGSARTRSPSPAESPGAEKMLPMSSIQNDFYDQEFSTHMERNPRERLVRHIGAVKDCNLETVDLVESEGVAAWATLPRLTRYPGLILLNGNSRCHGRMSPVALPEDPLTMRFPVSPLLRSCGEELRKTQQMELGMGPLGNNNNNNYQQKNQNVILALPASASENIAGVLRDLANLLHLAPALTCKIIEDKIGNKLEDQFMNQDDEKHVDFKRPLSQVSHGHLRKILNGRRKLCRSCGNVVHATGLRVPRHSVPALEEQLPRLAQLMDMLPRKSVPPPFVYFCDRACFARFKWNGKDGQAEAASLLLQPAGGSAVKSSNGDSPGSFCASSTAPAEMVVKQEPEDEDEKTPSVPGNPTNIPAQRKCIVKCFSADCFTTDSAPSGLELDGTAGAGTGAGPVNNTVWETETSGLQLEDTRQCVFCNQRGDGQADGPSRLLNFDVDKWVHLNCALWSNGVYETVSGALMNFQTALQAGLSQACSACHQPGATIKCFKSRCNSLYHLPCAIREECVFYKNKSVHCSVHGHAHAGITMGAGAGATTGAGLGGSVADNELSSLVVHRRVFVDRDENRQVATVMHYSELSNLLRVGNMTFLNVGQLLPHQLEAFHTPHYIYPIGYKVSRYYWCVRRPNRRCRYICSIAEAGCKPEFRIQVQDAGDKEPEREFRGSSPSAVWQQILQPITRLRKVHKWLQLFPQHISGEDLFGLTEPAIVRILESLPGIETLTDYRFKYGRNPLLEFPLAINPSGAARTEPKQRQLLVWRKPHTQRTAGSCSTQRMANSAAIAGEVACPYSKQFVHSKSSQYKKMKQEWRNNVYLARSKIQGLGLYAARDIEKHTMIIEYIGEVIRTEVSEIREKQYESKNRGIYMFRLDEDRVVDATLSGGLARYINHSCNPNCVTEIVEVDRDVRIIIFAKRKIYRGEELSYDYKFDIEDESHKIPCACGAPNCRKWMN

>gi|19550181|ref|NP_599108.1| trithorax, isoform C [Drosophila melanogaster]

MPNEVARDPSPSSCTAAANGAASGKGSASNGPPAMASSGDGSSPKSGADTGPSTSSTTAKQKKTVTFRNVLETSDDKSVVKRFYNPDIRIPIVSIMKKDSLNRPLNYSRGGECIVRPSILSKILNKNSNIDKLNSLKFRSAGASSSSSNQESGSSSNVFGLSRAFGAPMDEDDEGGVTFRRNDSPEDQNNAEDDEMDDDDDDEEAEEDDENEDDNDEAVSEKSAETEKSAGADERDPDEKQLVMDSHFVLPKRSTRSSRIIKPNKRLLEEGAISTKKPLSLGDSKGKNVFGTSSSSAGSTASTFSASTNLKLGKETFFNFGTLKPNSSAAGNFVLRQPRLQFQADNQQATFTAPKACPTSPSAIPKPANSLATSSFGSLASTNSSTVTPTPSACSICSAVVSSKEVTQARKYGVVACDVCRKFFSKMTKKSISANSSTANTSSGSQQYLQCKGNEGSPCSIHSAKSQLKNFKKFYKDRCTACWLKKCMISFQLPAAHRSRLSAILPPGMRGEAAAREEKSAELLSPTGSLRFTSTASSSSPSVVASTSVKWKSSGDSTSALTSIKPNPLAENNVTFGSTPLLRPAILENPLFLKISNAADQKLAAAEAISPSLTKKNSKQEKEKVKESEQSEKLLSPTQAGTKKSGAAEAQVEEVQPQKEEAPQTSTTTQPSASNGASHGVPQAELAGETNATGDTLKRQRIDLKGPRVKHVCRSASIVLGQPLATFGEDQQPEDAADMQQEIAAPVPSAIMEPSPEKPTHIVTDENDNCASCKTSPVGDESKPSKSSGSAQAEVKKATALGKEGTASAAGGSSAKVTTRNAAVASNLIVAASKKQRNGDIATSSSVTQSSNQTQGRKTKEHRQQRTLISIDFWENYDPAEVCQTGFGLIVTETVAQRALCFLCGSTGLDPLIFCACCCEPYHQYCVQDEYNLKHGSFEDTTLMGSLLETTVNASTGPSSSLNQLTQRLNWLCPRCTVCYTCNMSSGSKVKCQKCQKNYHSTCLGTSKRLLGADRPLICVNCLKCKSCSTTKVSKFVGNLPMCTGCFKLRKKGNFCPICQRCYDDNDFDLKMMECGDCGQWVHSKCEGLSDEQYNLLSTLPESIEFICKKCARRNESSKIKAEEWRQAVMEEFKASLYSVLKLLSKSRQACALLKLSPRKKLRCTCGASSNQGKLQPKALQFSSGSDNGLGSDGESQNSDDVYEFKDQQQQQQQRNANMNKPRVKSLPCSCQQHISHSQSFSLVDIKQKIAGNSYVSLAEFNYDMSQVIQQSNCDELDIAYKELLSEQFPWFQNETKACTDALEEDMFESCSGGNYEDLQDTGGVSASVYNEHSTSQAESRSGVLDIPLEEVDDFGSCGIKMRLDTRMCLFCRKSGEGLSGEEARLLYCGHDCWVHTNCAMWSAEVFEEIDGSLQNVHSAVARGRMIKCTVCGNRGATVGCNVRSCGEHYHYPCARSIDCAFLTDKSMYCPAHAKNGNALKANGSPSVTYESNFEVSRPVYVELDRKRKKLIEPARVQFHIGSLEVRQLGAIVPRFSDSYEAVVPINFLCSRLYWSSKEPWKIVEYTVRTTIQNSSSTLTALDVGRNYTVDHTNPNSKEVQLGMAQIARWHTSLARSEFLENGGTDWSGEFPNPNSCVPPDENTEEEPQQQADLLPPELKDAIFEDLPHELLDGISMLDIFLYDDKTDLFAISEQSKDGTQAMTSNQAQNQNQQAGGANSVSICDEDTRNSNTSLGNGWPASNPVEDAMLSAARNSSQVQMLKTLAWPKLDGNSAMATAIKRRKLSKNLAEGVFLTLSSQQRNKKEMATVAGVSRRQSISETSVEGVATTSGSVRSKSFTWSAAKRYFEKSEGREEAAKMRIMQMDGVDDSITEFRIISGDGNLSTAQFSGQVKCDRCQCTYRNYDAFQRHLPSCSPTMSSNETESDVSGQGMTNNATQISAESLNELQKQLLANAGGLNYLQSATSFPQVQSLGSLGQFGLQGLQQLQLQPQSLGSGFFLSQPNPATQANTDDLQIYANSLQSLAANLGGGFTLAQPTVTAPAQPQLIAVSTNPDGTQQFIQIPQTMQATTTPTATYQTLQATNTDKKIMLPLTAAGKPLKTVATKAAQQAAVKQRQLKSGHQVKPIQAKLQPHPQQHQQQQQTQVQQPITVMGQNLLQPQLLFQSSTQTQAPQIILPQAQPQNIISFVTGDGSQGQPLQYISIPTAGEYKPQPQPTATPTFLTTAPGAGATYLQTDASGNLVLTTTPSNSGLQMLTAQSLQAQPQVIGTLIQPQTIQLGGGADGNQPGSNQQPLILGGTGGGSSGLEFATTSPQVILATQPMYYGLETIVQNTVMSSQQFVSTAMPGMLSQNASFSATTTQVFQASKIEPIVDLPAGYVVLNNTGDASSAGTFLNAASVLQQQTQDDTTTQILQNANFQFQSVPTSSGASTSMDYTSPVMVTAKIPPVTQIKRTNAQAKAAGISGVGKVPPQPQVVNKVLPTSIVTQQSQVQVKNSNLKQSQVKGKAASGTGTTCGAPPSIASKPLQKKTNMIRPIHKLEVKPKVMKPTPKVQNQNHSLLQQQQQQQPQLQQQIPAVVVNQVPKVTISQQRIPAQTQQQQLQQAQMIHIPQQQQPLQQQQVQVQPSMPIITLAEAPVVQSQFVMEPQALEQQELANRVQHFSTSSSSSSSNCSLPTNVVNPMQQQAPSTTSSSTTRPTNRVLPMQQRQEPAPLSNECPVVSSPTPPKPVEQPIIHQMTSASVSKCYAQKSTLPSPVYEAELKVSSVLESIVPDVTMDAILEEQPVTESIYTEGLYEKNSPGESKTEQLLLQQQQREQLNQQLVNNGYLLDKHTFQVEPMDTDVYREEDLEEEEDEDDDFSLKMATSACNDHEMSDSEEPAVKDKISKILDNLTNDDCADSIATATTMEVDASAGYQQMVEDVLATTAAQSAPTEEFEGALETAAVEAAATYINEMADAHVLDLKQLQNGVELELRRRKEEQRTVSQEQEQSKAAIVPTAAAPEPPQPIQEPKKMTGPHLLYEIQSEDGFTYKSSSITEIWEKVFEAVQVARRAHGLTPLPEGPLADMGGIQMIGLKTNALKYLIEQLPGVEKCSKYTPKYHKRNGNVSTAANGAHGGNLGGSSASAALSVSGGDSHGLLDYGSDQDELEENAYDCARCEPYSNRSEYDMFSWLASRHRKQPIQVFVQPSDNELVPRRGTGSNLPMAMKYRTLKETYKDYVGVFRSHIHGRGLYCTKDIEAGEMVIEYAGELIRSTLTDKRERYYDSRGIGCYMFKIDDNLVVDATMRGNAARFINHCCEPNCYSKVVDILGHKHIIIFALRRIVQGEELTYDYKFPFEDEKIPCSCGSKRCRKYL

***Homo sapiens*  (Metazoa)**

>gi|110349788|ref|NP_060959.2| histone-lysine N-methyltransferase ASH1L [Homo sapiens]

MDPRNTAMLGLGSDSEGFSRKSPSAISTGTLVSKREVELEKNTKEEEDLRKRNRERNIEAGKDDGLTDAQQQFSVKETNFSEGNLKLKIGLQAKRTKKPPKNLENYVCRPAIKTTIKHPRKALKSGKMTDEKNEHCPSKRDPSKLYKKADDVAAIECQSEEVIRLHSQGENNPLSKKLSPVHSEMADYINATPSTLLGSRDPDLKDRALLNGGTSVTEKLAQLIATCPPSKSSKTKPKKLGTGTTAGLVSKDLIRKAGVGSVAGIIHKDLIKKPTISTAVGLVTKDPGKKPVFNAAVGLVNKDSVKKLGTGTTAVFINKNLGKKPGTITTVGLLSKDSGKKLGIGIVPGLVHKESGKKLGLGTVVGLVNKDLGKKLGSTVGLVAKDCAKKIVASSAMGLVNKDIGKKLMSCPLAGLISKDAINLKAEALLPTQEPLKASCSTNINNQESQELSESLKDSATSKTFEKNVVRQNKESILEKFSVRKEIINLEKEMFNEGTCIQQDSFSSSEKGSYETSKHEKQPPVYCTSPDFKMGGASDVSTAKSPFSAVGESNLPSPSPTVSVNPLTRSPPETSSQLAPNPLLLSSTTELIEEISESVGKNQFTSESTHLNVGHRSVGHSISIECKGIDKEVNDSKTTHIDIPRISSSLGKKPSLTSESSIHTITPSVVNFTSLFSNKPFLKLGAVSASDKHCQVAESLSTSLQSKPLKKRKGRKPRWTKVVARSTCRSPKGLELERSELFKNVSCSSLSNSNSEPAKFMKNIGPPSFVDHDFLKRRLPKLSKSTAPSLALLADSEKPSHKSFATHKLSSSMCVSSDLLSDIYKPKRGRPKSKEMPQLEGPPKRTLKIPASKVFSLQSKEEQEPPILQPEIEIPSFKQGLSVSPFPKKRGRPKRQMRSPVKMKPPVLSVAPFVATESPSKLESESDNHRSSSDFFESEDQLQDPDDLDDSHRPSVCSMSDLEMEPDKKITKRNNGQLMKTIIRKINKMKTLKRKKLLNQILSSSVESSNKGKVQSKLHNTVSSLAATFGSKLGQQINVSKKGTIYIGKRRGRKPKTVLNGILSGSPTSLAVLEQTAQQAAGSALGQILPPLLPSSASSSEILPSPICSQSSGTSGGQSPVSSDAGFVEPSSVPYLHLHSRQGSMIQTLAMKKASKGRRRLSPPTLLPNSPSHLSELTSLKEATPSPISESHSDETIPSDSGIGTDNNSTSDRAEKFCGQKKRRHSFEHVSLIPPETSTVLSSLKEKHKHKCKRRNHDYLSYDKMKRQKRKRKKKYPQLRNRQDPDFIAELEELISRLSEIRITHRSHHFIPRDLLPTIFRINFNSFYTHPSFPLDPLHYIRKPDLKKKRGRPPKMREAMAEMPFMHSLSFPLSSTGFYPSYGMPYSPSPLTAAPIGLGYYGRYPPTLYPPPPSPSFTTPLPPPSYMHAGHLLLNPAKYHKKKHKLLRQEAFLTTSRTPLLSMSTYPSVPPEMAYGWMVEHKHRHRHKHREHRSSEQPQVSMDTGSSRSVLESLKRYRFGKDAVGERYKHKEKHRCHMSCPHLSPSKSLINREEQWVHREPSESSPLALGLQTPLQIDCSESSPSLSLGGFTPNSEPASSDEHTNLFTSAIGSCRVSNPNSSGRKKLTDSPGLFSAQDTSLNRLHRKESLPSNERAVQTLAGSQPTSDKPSQRPSESTNCSPTRKRSSSESTSSTVNGVPSRSPRLVASGDDSVDSLLQRMVQNEDQEPMEKSIDAVIATASAPPSSSPGRSHSKDRTLGKPDSLLVPAVTSDSCNNSISLLSEKLTSSCSPHHIKRSVVEAMQRQARKMCNYDKILATKKNLDHVNKILKAKKLQRQARTGNNFVKRRPGRPRKCPLQAVVSMQAFQAAQFVNPELNRDEEGAALHLSPDTVTDVIEAVVQSVNLNPEHKKGLKRKGWLLEEQTRKKQKPLPEEEEQENNKSFNEAPVEIPSPSETPAKPSEPESTLQPVLSLIPREKKPPRPPKKKYQKAGLYSDVYKTTDPKSRLIQLKKEKLEYTPGEHEYGLFPAPIHVGKYLRQKRIDFQLPYDILWQWKHNQLYKKPDVPLYKKIRSNVYVDVKPLSGYEATTCNCKKPDDDTRKGCVDDCLNRMIFAECSPNTCPCGEQCCNQRIQRHEWVQCLERFRAEEKGWGIRTKEPLKAGQFIIEYLGEVVSEQEFRNRMIEQYHNHSDHYCLNLDSGMVIDSYRMGNEARFINHSCDPNCEMQKWSVNGVYRIGLYALKDMPAGTELTYDYNFHSFNVEKQQLCKCGFEKCRGIIGGKSQRVNGLTSSKNSQPMATHKKSGRSKEKRKSKHKLKKRRGHLSEEPSENINTPTRLTPQLQMKPMSNRERNFVLKHHVFLVRNWEKIRQKQEEVKHTSDNIHSASLYTRWNGICRDDGNIKSDVFMTQFSALQTARSVRTRRLAAAEENIEVARAARLAQIFKEICDGIISYKDSSRQALAAPLLNLPPKKKNADYYEKISDPLDLITIEKQILTGYYKTVEAFDADMLKVFRNAEKYYGRKSPVGRDVCRLRKAYYNARHEASAQIDEIVGETASEADSSETSVSEKENGHEKDDDVIRCICGLYKDEGLMIQCDKCMVWQHCDCMGVNSDVEHYLCEQCDPRPVDREVPMIPRPHYAQPGCVYFICLLRDDLLLRQGDCVYLMRDSRRTPDGHPVRQSYRLLSHINRDKLDIFRIEKLWKNEKEERFAFGHHYFRPHETHHSPSRRFYHNELFRVPLYEIIPLEAVVGTCCVLDLYTYCKGRPKGVKEQDVYICDYRLDKSAHLFYKIHRNRYPVCTKPYAFDHFPKKLTPKKDFSPHYVPDNYKRNGGRSSWKSERSKPPLKDLGQEDDALPLIEEVLASQEQAANEIPSLEEPEREGATANVSEGEKKTEESSQEPQSTCTPEERRHNQRERLNQILLNLLEKIPGKNAIDVTYLLEEGSGRKLRRRTLFIPENSFRK

>gi|224465233|ref|NP_079033.4| histone-lysine N-methyltransferase EHMT1 isoform 1 [Homo sapiens]

MAAADAEAVPARGEPQQDCCVKTELLGEETPMAADEGSAEKQAGEAHMAADGETNGSCENSDASSHANAAKHTQDSARVNPQDGTNTLTRIAENGVSERDSEAAKQNHVTADDFVQTSVIGSNGYILNKPALQAQPLRTTSTLASSLPGHAAKTLPGGAGKGRTPSAFPQTPAAPPATLGEGSADTEDRKLPAPGADVKVHRARKTMPKSVVGLHAASKDPREVREARDHKEPKEEINKNISDFGRQQLLPPFPSLHQSLPQNQCYMATTKSQTACLPFVLAAAVSRKKKRRMGTYSLVPKKKTKVLKQRTVIEMFKSITHSTVGSKGEKDLGASSLHVNGESLEMDSDEDDSEELEEDDGHGAEQAAAFPTEDSRTSKESMSEADRAQKMDGESEEEQESVDTGEEEEGGDESDLSSESSIKKKFLKRKGKTDSPWIKPARKRRRRSRKKPSGALGSESYKSSAGSAEQTAPGDSTGYMEVSLDSLDLRVKGILSSQAEGLANGPDVLETDGLQEVPLCSCRMETPKSREITTLANNQCMATESVDHELGRCTNSVVKYELMRPSNKAPLLVLCEDHRGRMVKHQCCPGCGYFCTAGNFMECQPESSISHRFHKDCASRVNNASYCPHCGEESSKAKEVTIAKADTTSTVTPVPGQEKGSALEGRADTTTGSAAGPPLSEDDKLQGAASHVPEGFDPTGPAGLGRPTPGLSQGPGKETLESALIALDSEKPKKLRFHPKQLYFSARQGELQKVLLMLVDGIDPNFKMEHQNKRSPLHAAAEAGHVDICHMLVQAGANIDTCSEDQRTPLMEAAENNHLEAVKYLIKAGALVDPKDAEGSTCLHLAAKKGHYEVVQYLLSNGQMDVNCQDDGGWTPMIWATEYKHVDLVKLLLSKGSDINIRDNEENICLHWAAFSGCVDIAEILLAAKCDLHAVNIHGDSPLHIAARENRYDCVVLFLSRDSDVTLKNKEGETPLQCASLNSQVWSALQMSKALQDSAPDRPSPVERIVSRDIARGYERIPIPCVNAVDSEPCPSNYKYVSQNCVTSPMNIDRNITHLQYCVCIDDCSSSNCMCGQLSMRCWYDKDGRLLPEFNMAEPPLIFECNHACSCWRNCRNRVVQNGLRARLQLYRTRDMGWGVRSLQDIPPGTFVCEYVGELISDSEADVREEDSYLFDLDNKDGEVYCIDARFYGNVSRFINHHCEPNLVPVRVFMAHQDLRFPRIAFFSTRLIEAGEQLGFDYGERFWDIKGKLFSCRCGSPKCRHSSAALAQRQASAAQEAQEDGLPDTSSAAAADPL

>gi|156142197|ref|NP_006700.3| histone-lysine N-methyltransferase EHMT2 isoform a [Homo sapiens]

MAAAAGAAAAAAAEGEAPAEMGALLLEKETRGATERVHGSLGDTPRSEETLPKATPDSLEPAGPSSPASVTVTVGDEGADTPVGATPLIGDESENLEGDGDLRGGRILLGHATKSFPSSPSKGGSCPSRAKMSMTGAGKSPPSVQSLAMRLLSMPGAQGAAAAGSEPPPATTSPEGQPKVHRARKTMSKPGNGQPPVPEKRPPEIQHFRMSDDVHSLGKVTSDLAKRRKLNSGGGLSEELGSARRSGEVTLTKGDPGSLEEWETVVGDDFSLYYDSYSVDERVDSDSKSEVEALTEQLSEEEEEEEEEEEEEEEEEEEEEEEEDEESGNQSDRSGSSGRRKAKKKWRKDSPWVKPSRKRRKREPPRAKEPRGVNGVGSSGPSEYMEVPLGSLELPSEGTLSPNHAGVSNDTSSLETERGFEELPLCSCRMEAPKIDRISERAGHKCMATESVDGELSGCNAAILKRETMRPSSRVALMVLCETHRARMVKHHCCPGCGYFCTAGTFLECHPDFRVAHRFHKACVSQLNGMVFCPHCGEDASEAQEVTIPRGDGVTPPAGTAAPAPPPLSQDVPGRADTSQPSARMRGHGEPRRPPCDPLADTIDSSGPSLTLPNGGCLSAVGLPLGPGREALEKALVIQESERRKKLRFHPRQLYLSVKQGELQKVILMLLDNLDPNFQSDQQSKRTPLHAAAQKGSVEICHVLLQAGANINAVDKQQRTPLMEAVVNNHLEVARYMVQRGGCVYSKEEDGSTCLHHAAKIGNLEMVSLLLSTGQVDVNAQDSGGWTPIIWAAEHKHIEVIRMLLTRGADVTLTDNEENICLHWASFTGSAAIAEVLLNARCDLHAVNYHGDTPLHIAARESYHDCVLLFLSRGANPELRNKEGDTAWDLTPERSDVWFALQLNRKLRLGVGNRAIRTEKIICRDVARGYENVPIPCVNGVDGEPCPEDYKYISENCETSTMNIDRNITHLQHCTCVDDCSSSNCLCGQLSIRCWYDKDGRLLQEFNKIEPPLIFECNQACSCWRNCKNRVVQSGIKVRLQLYRTAKMGWGVRALQTIPQGTFICEYVGELISDAEADVREDDSYLFDLDNKDGEVYCIDARYYGNISRFINHLCDPNIIPVRVFMLHQDLRFPRIAFFSSRDIRTGEELGFDYGDRFWDIKSKYFTCQCGSEKCKHSAEAIALEQSRLARLDPHPELLPELGSLPPVNT

>gi|19923202|ref|NP_001982.2| histone-lysine N-methyltransferase EZH1 [Homo sapiens]

MEIPNPPTSKCITYWKRKVKSEYMRLRQLKRLQANMGAKALYVANFAKVQEKTQILNEEWKKLRVQPVQSMKPVSGHPFLKKCTIESIFPGFASQHMLMRSLNTVALVPIMYSWSPLQQNFMVEDETVLCNIPYMGDEVKEEDETFIEELINNYDGKVHGEEEMIPGSVLISDAVFLELVDALNQYSDEEEEGHNDTSDGKQDDSKEDLPVTRKRKRHAIEGNKKSSKKQFPNDMIFSAIASMFPENGVPDDMKERYRELTEMSDPNALPPQCTPNIDGPNAKSVQREQSLHSFHTLFCRRCFKYDCFLHPFHATPNVYKRKNKEIKIEPEPCGTDCFLLLEGAKEYAMLHNPRSKCSGRRRRRHHIVSASCSNASASAVAETKEGDSDRDTGNDWASSSSEANSRCQTPTKQKASPAPPQLCVVEAPSEPVEWTGAEESLFRVFHGTYFNNFCSIARLLGTKTCKQVFQFAVKESLILKLPTDELMNPSQKKKRKHRLWAAHCRKIQLKKDNSSTQVYNYQPCDHPDRPCDSTCPCIMTQNFCEKFCQCNPDCQNRFPGCRCKTQCNTKQCPCYLAVRECDPDLCLTCGASEHWDCKVVSCKNCSIQRGLKKHLLLAPSDVAGWGTFIKESVQKNEFISEYCGELISQDEADRRGKVYDKYMSSFLFNLNNDFVVDATRKGNKIRFANHSVNPNCYAKVVMVNGDHRIGIFAKRAIQAGEELFFDYRYSQADALKYVGIERETDVL

>gi|21361095|ref|NP_004447.2| histone-lysine N-methyltransferase EZH2 isoform a [Homo sapiens]

MGQTGKKSEKGPVCWRKRVKSEYMRLRQLKRFRRADEVKSMFSSNRQKILERTEILNQEWKQRRIQPVHILTSVSSLRGTRECSVTSDLDFPTQVIPLKTLNAVASVPIMYSWSPLQQNFMVEDETVLHNIPYMGDEVLDQDGTFIEELIKNYDGKVHGDRECGFINDEIFVELVNALGQYNDDDDDDDGDDPEEREEKQKDLEDHRDDKESRPPRKFPSDKIFEAISSMFPDKGTAEELKEKYKELTEQQLPGALPPECTPNIDGPNAKSVQREQSLHSFHTLFCRRCFKYDCFLHRKCNYSFHATPNTYKRKNTETALDNKPCGPQCYQHLEGAKEFAAALTAERIKTPPKRPGGRRRGRLPNNSSRPSTPTINVLESKDTDSDREAGTETGGENNDKEEEEKKDETSSSSEANSRCQTPIKMKPNIEPPENVEWSGAEASMFRVLIGTYYDNFCAIARLIGTKTCRQVYEFRVKESSIIAPAPAEDVDTPPRKKKRKHRLWAAHCRKIQLKKDGSSNHVYNYQPCDHPRQPCDSSCPCVIAQNFCEKFCQCSSECQNRFPGCRCKAQCNTKQCPCYLAVRECDPDLCLTCGAADHWDSKNVSCKNCSIQRGSKKHLLLAPSDVAGWGIFIKDPVQKNEFISEYCGEIISQDEADRRGKVYDKYMCSFLFNLNNDFVVDATRKGNKIRFANHSVNPNCYAKVMMVNGDHRIGIFAKRAIQTGEELFFDYRYSQADALKYVGIEREMEIP

>gi|56550039|ref|NP_005924.2| histone-lysine N-methyltransferase MLL isoform 2 precursor [Homo sapiens]

MAHSCRWRFPARPGTTGGGGGGGRRGLGGAPRQRVPALLLPPGPPVGGGGPGAPPSPPAVAAAAAAAGSSGAGVPGGAAAASAASSSSASSSSSSSSSASSGPALLRVGPGFDAALQVSAAIGTNLRRFRAVFGESGGGGGSGEDEQFLGFGSDEEVRVRSPTRSPSVKTSPRKPRGRPRSGSDRNSAILSDPSVFSPLNKSETKSGDKIKKKDSKSIEKKRGRPPTFPGVKIKITHGKDISELPKGNKEDSLKKIKRTPSATFQQATKIKKLRAGKLSPLKSKFKTGKLQIGRKGVQIVRRRGRPPSTERIKTPSGLLINSELEKPQKVRKDKEGTPPLTKEDKTVVRQSPRRIKPVRIIPSSKRTDATIAKQLLQRAKKGAQKKIEKEAAQLQGRKVKTQVKNIRQFIMPVVSAISSRIIKTPRRFIEDEDYDPPIKIARLESTPNSRFSAPSCGSSEKSSAASQHSSQMSSDSSRSSSPSVDTSTDSQASEEIQVLPEERSDTPEVHPPLPISQSPENESNDRRSRRYSVSERSFGSRTTKKLSTLQSAPQQQTSSSPPPPLLTPPPPLQPASSISDHTPWLMPPTIPLASPFLPASTAPMQGKRKSILREPTFRWTSLKHSRSEPQYFSSAKYAKEGLIRKPIFDNFRPPPLTPEDVGFASGFSASGTAASARLFSPLHSGTRFDMHKRSPLLRAPRFTPSEAHSRIFESVTLPSNRTSAGTSSSGVSNRKRKRKVFSPIRSEPRSPSHSMRTRSGRLSSSELSPLTPPSSVSSSLSISVSPLATSALNPTFTFPSHSLTQSGESAEKNQRPRKQTSAPAEPFSSSSPTPLFPWFTPGSQTERGRNKDKAPEELSKDRDADKSVEKDKSRERDREREKENKRESRKEKRKKGSEIQSSSALYPVGRVSKEKVVGEDVATSSSAKKATGRKKSSSHDSGTDITSVTLGDTTAVKTKILIKKGRGNLEKTNLDLGPTAPSLEKEKTLCLSTPSSSTVKHSTSSIGSMLAQADKLPMTDKRVASLLKKAKAQLCKIEKSKSLKQTDQPKAQGQESDSSETSVRGPRIKHVCRRAAVALGRKRAVFPDDMPTLSALPWEEREKILSSMGNDDKSSIAGSEDAEPLAPPIKPIKPVTRNKAPQEPPVKKGRRSRRCGQCPGCQVPEDCGVCTNCLDKPKFGGRNIKKQCCKMRKCQNLQWMPSKAYLQKQAKAVKKKEKKSKTSEKKDSKESSVVKNVVDSSQKPTPSAREDPAPKKSSSEPPPRKPVEEKSEEGNVSAPGPESKQATTPASRKSSKQVSQPALVIPPQPPTTGPPRKEVPKTTPSEPKKKQPPPPESGPEQSKQKKVAPRPSIPVKQKPKEKEKPPPVNKQENAGTLNILSTLSNGNSSKQKIPADGVHRIRVDFKEDCEAENVWEMGGLGILTSVPITPRVVCFLCASSGHVEFVYCQVCCEPFHKFCLEENERPLEDQLENWCCRRCKFCHVCGRQHQATKQLLECNKCRNSYHPECLGPNYPTKPTKKKKVWICTKCVRCKSCGSTTPGKGWDAQWSHDFSLCHDCAKLFAKGNFCPLCDKCYDDDDYESKMMQCGKCDRWVHSKCENLSDEMYEILSNLPESVAYTCVNCTERHPAEWRLALEKELQISLKQVLTALLNSRTTSHLLRYRQAAKPPDLNPETEESIPSRSSPEGPDPPVLTEVSKQDDQQPLDLEGVKRKMDQGNYTSVLEFSDDIVKIIQAAINSDGGQPEIKKANSMVKSFFIRQMERVFPWFSVKKSRFWEPNKVSSNSGMLPNAVLPPSLDHNYAQWQEREENSHTEQPPLMKKIIPAPKPKGPGEPDSPTPLHPPTPPILSTDRSREDSPELNPPPGIEDNRQCALCLTYGDDSANDAGRLLYIGQNEWTHVNCALWSAEVFEDDDGSLKNVHMAVIRGKQLRCEFCQKPGATVGCCLTSCTSNYHFMCSRAKNCVFLDDKKVYCQRHRDLIKGEVVPENGFEVFRRVFVDFEGISLRRKFLNGLEPENIHMMIGSMTIDCLGILNDLSDCEDKLFPIGYQCSRVYWSTTDARKRCVYTCKIVECRPPVVEPDINSTVEHDENRTIAHSPTSFTESSSKESQNTAEIISPPSPDRPPHSQTSGSCYYHVISKVPRIRTPSYSPTQRSPGCRPLPSAGSPTPTTHEIVTVGDPLLSSGLRSIGSRRHSTSSLSPQRSKLRIMSPMRTGNTYSRNNVSSVSTTGTATDLESSAKVVDHVLGPLNSSTSLGQNTSTSSNLQRTVVTVGNKNSHLDGSSSSEMKQSSASDLVSKSSSLKGEKTKVLSSKSSEGSAHNVAYPGIPKLAPQVHNTTSRELNVSKIGSFAEPSSVSFSSKEALSFPHLHLRGQRNDRDQHTDSTQSANSSPDEDTEVKTLKLSGMSNRSSIINEHMGSSSRDRRQKGKKSCKETFKEKHSSKSFLEPGQVTTGEEGNLKPEFMDEVLTPEYMGQRPCNNVSSDKIGDKGLSMPGVPKAPPMQVEGSAKELQAPRKRTVKVTLTPLKMENESQSKNALKESSPASPLQIESTSPTEPISASENPGDGPVAQPSPNNTSCQDSQSNNYQNLPVQDRNLMLPDGPKPQEDGSFKRRYPRRSARARSNMFFGLTPLYGVRSYGEEDIPFYSSSTGKKRGKRSAEGQVDGADDLSTSDEDDLYYYNFTRTVISSGGEERLASHNLFREEEQCDLPKISQLDGVDDGTESDTSVTATTRKSSQIPKRNGKENGTENLKIDRPEDAGEKEHVTKSSVGHKNEPKMDNCHSVSRVKTQGQDSLEAQLSSLESSRRVHTSTPSDKNLLDTYNTELLKSDSDNNNSDDCGNILPSDIMDFVLKNTPSMQALGESPESSSSELLNLGEGLGLDSNREKDMGLFEVFSQQLPTTEPVDSSVSSSISAEEQFELPLELPSDLSVLTTRSPTVPSQNPSRLAVISDSGEKRVTITEKSVASSESDPALLSPGVDPTPEGHMTPDHFIQGHMDADHISSPPCGSVEQGHGNNQDLTRNSSTPGLQVPVSPTVPIQNQKYVPNSTDSPGPSQISNAAVQTTPPHLKPATEKLIVVNQNMQPLYVLQTLPNGVTQKIQLTSSVSSTPSVMETNTSVLGPMGGGLTLTTGLNPSLPTSQSLFPSASKGLLPMSHHQHLHSFPAATQSSFPPNISNPPSGLLIGVQPPPDPQLLVSESSQRTDLSTTVATPSSGLKKRPISRLQTRKNKKLAPSSTPSNIAPSDVVSNMTLINFTPSQLPNHPSLLDLGSLNTSSHRTVPNIIKRSKSSIMYFEPAPLLPQSVGGTAATAAGTSTISQDTSHLTSGSVSGLASSSSVLNVVSMQTTTTPTSSASVPGHVTLTNPRLLGTPDIGSISNLLIKASQQSLGIQDQPVALPPSSGMFPQLGTSQTPSTAAITAASSICVLPSTQTTGITAASPSGEADEHYQLQHVNQLLASKTGIHSSQRDLDSASGPQVSNFTQTVDAPNSMGLEQNKALSSAVQASPTSPGGSPSSPSSGQRSASPSVPGPTKPKPKTKRFQLPLDKGNGKKHKVSHLRTSSSEAHIPDQETTSLTSGTGTPGAEAEQQDTASVEQSSQKECGQPAGQVAVLPEVQVTQNPANEQESAEPKTVEEEESNFSSPLMLWLQQEQKRKESITEKKPKKGLVFEISSDDGFQICAESIEDAWKSLTDKVQEARSNARLKQLSFAGVNGLRMLGILHDAVVFLIEQLSGAKHCRNYKFRFHKPEEANEPPLNPHGSARAEVHLRKSAFDMFNFLASKHRQPPEYNPNDEEEEEVQLKSARRATSMDLPMPMRFRHLKKTSKEAVGVYRSPIHGRGLFCKRNIDAGEMVIEYAGNVIRSIQTDKREKYYDSKGIGCYMFRIDDSEVVDATMHGNAARFINHSCEPNCYSRVINIDGQKHIVIFAMRKIYRGEELTYDYKFPIEDASNKLPCNCGAKKCRKFLN

>gi|148762969|ref|NP_003473.3| histone-lysine N-methyltransferase MLL2 [Homo sapiens]

MDSQKLAGEDKDSEPAADGPAASEDPSATESDLPNPHVGEVSVLSSGSPRLQETPQDCSGGPVRRCALCNCGEPSLHGQRELRRFELPFDWPRCPVVSPGGSPGPNEAVLPSEDLSQIGFPEGLTPAHLGEPGGSCWAHHWCAAWSAGVWGQEGPELCGVDKAIFSGISQRCSHCTRLGASIPCRSPGCPRLYHFPCATASGSFLSMKTLQLLCPEHSEGAAYLEEARCAVCEGPGELCDLFFCTSCGHHYHGACLDTALTARKRAGWQCPECKVCQACRKPGNDSKMLVCETCDKGYHTFCLKPPMEELPAHSWKCKACRVCRACGAGSAELNPNSEWFENYSLCHRCHKAQGGQTIRSVAEQHTPVCSRFSPPEPGDTPTDEPDALYVACQGQPKGGHVTSMQPKEPGPLQCEAKPLGKAGVQLEPQLEAPLNEEMPLLPPPEESPLSPPPEESPTSPPPEASRLSPPPEELPASPLPEALHLSRPLEESPLSPPPEESPLSPPPESSPFSPLEESPLSPPEESPPSPALETPLSPPPEASPLSPPFEESPLSPPPEELPTSPPPEASRLSPPPEESPMSPPPEESPMSPPPEASRLFPPFEESPLSPPPEESPLSPPPEASRLSPPPEDSPMSPPPEESPMSPPPEVSRLSPLPVVSRLSPPPEESPLSPPPEESPTSPPPEASRLSPPPEDSPTSPPPEDSPASPPPEDSLMSLPLEESPLLPLPEEPQLCPRSEGPHLSPRPEEPHLSPRPEEPHLSPQAEEPHLSPQPEEPCLCAVPEEPHLSPQAEGPHLSPQPEELHLSPQTEEPHLSPVPEEPCLSPQPEESHLSPQSEEPCLSPRPEESHLSPELEKPPLSPRPEKPPEEPGQCPAPEELPLFPPPGEPSLSPLLGEPALSEPGEPPLSPLPEELPLSPSGEPSLSPQLMPPDPLPPPLSPIITAAAPPALSPLGELEYPFGAKGDSDPESPLAAPILETPISPPPEANCTDPEPVPPMILPPSPGSPVGPASPILMEPLPPQCSPLLQHSLVPQNSPPSQCSPPALPLSVPSPLSPIGKVVGVSDEAELHEMETEKVSEPECPALEPSATSPLPSPMGDLSCPAPSPAPALDDFSGLGEDTAPLDGIDAPGSQPEPGQTPGSLASELKGSPVLLDPEELAPVTPMEVYPECKQTAGQGSPCEEQEEPRAPVAPTPPTLIKSDIVNEISNLSQGDASASFPGSEPLLGSPDPEGGGSLSMELGVSTDVSPARDEGSLRLCTDSLPETDDSLLCDAGTAISGGKAEGEKGRRRSSPARSRIKQGRSSSFPGRRRPRGGAHGGRGRGRARLKSTASSIETLVVADIDSSPSKEEEEEDDDTMQNTVVLFSNTDKFVLMQDMCVVCGSFGRGAEGHLLACSQCSQCYHPYCVNSKITKVMLLKGWRCVECIVCEVCGQASDPSRLLLCDDCDISYHTYCLDPPLLTVPKGGWKCKWCVSCMQCGAASPGFHCEWQNSYTHCGPCASLVTCPICHAPYVEEDLLIQCRHCERWMHAGCESLFTEDDVEQAADEGFDCVSCQPYVVKPVAPVAPPELVPMKVKEPEPQYFRFEGVWLTETGMALLRNLTMSPLHKRRQRRGRLGLPGEAGLEGSEPSDALGPDDKKDGDLDTDELLKGEGGVEHMECEIKLEGPVSPDVEPGKEETEESKKRKRKPYRPGIGGFMVRQRKSHTRTKKGPAAQAEVLSGDGQPDEVIPADLPAEGAVEQSLAEGDEKKKQQRRGRKKSKLEDMFPAYLQEAFFGKELLDLSRKALFAVGVGRPSFGLGTPKAKGDGGSERKELPTSQKGDDGPDIADEESRGLEGKADTPGPEDGGVKASPVPSDPEKPGTPGEGMLSSDLDRISTEELPKMESKDLQQLFKDVLGSEREQHLGCGTPGLEGSRTPLQRPFLQGGLPLGNLPSSSPMDSYPGLCQSPFLDSRERGGFFSPEPGEPDSPWTGSGGTTPSTPTTPTTEGEGDGLSYNQRSLQRWEKDEELGQLSTISPVLYANINFPNLKQDYPDWSSRCKQIMKLWRKVPAADKAPYLQKAKDNRAAHRINKVQKQAESQINKQTKVGDIARKTDRPALHLRIPPQPGALGSPPPAAAPTIFIGSPTTPAGLSTSADGFLKPPAGSVPGPDSPGELFLKLPPQVPAQVPSQDPFGLAPAYPLEPRFPTAPPTYPPYPSPTGAPAQPPMLGASSRPGAGQPGEFHTTPPGTPRHQPSTPDPFLKPRCPSLDNLAVPESPGVGGGKASEPLLSPPPFGESRKALEVKKEELGASSPSYGPPNLGFVDSPSSGTHLGGLELKTPDVFKAPLTPRASQVEPQSPGLGLRPQEPPPAQALAPSPPSHPDIFRPGSYTDPYAQPPLTPRPQPPPPESCCALPPRSLPSDPFSRVPASPQSQSSSQSPLTPRPLSAEAFCPSPVTPRFQSPDPYSRPPSRPQSRDPFAPLHKPPRPQPPEVAFKAGSLAHTSLGAGGFPAALPAGPAGELHAKVPSGQPPNFVRSPGTGAFVGTPSPMRFTFPQAVGEPSLKPPVPQPGLPPPHGINSHFGPGPTLGKPQSTNYTVATGNFHPSGSPLGPSSGSTGESYGLSPLRPPSVLPPPAPDGSLPYLSHGASQRSGITSPVEKREDPGTGMGSSLATAELPGTQDPGMSGLSQTELEKQRQRQRLRELLIRQQIQRNTLRQEKETAAAAAGAVGPPGSWGAEPSSPAFEQLSRGQTPFAGTQDKSSLVGLPPSKLSGPILGPGSFPSDDRLSRPPPPATPSSMDVNSRQLVGGSQAFYQRAPYPGSLPLQQQQQQLWQQQQATAATSMRFAMSARFPSTPGPELGRQALGSPLAGISTRLPGPGEPVPGPAGPAQFIELRHNVQKGLGPGGTPFPGQGPPQRPRFYPVSEDPHRLAPEGLRGLAVSGLPPQKPSAPPAPELNNSLHPTPHTKGPTLPTGLELVNRPPSSTELGRPNPLALEAGKLPCEDPELDDDFDAHKALEDDEELAHLGLGVDVAKGDDELGTLENLETNDPHLDDLLNGDEFDLLAYTDPELDTGDKKDIFNEHLRLVESANEKAEREALLRGVEPGPLGPEERPPPAADASEPRLASVLPEVKPKVEEGGRHPSPCQFTIATPKVEPAPAANSLGLGLKPGQSMMGSRDTRMGTGPFSSSGHTAEKASFGATGGPPAHLLTPSPLSGPGGSSLLEKFELESGALTLPGGPAASGDELDKMESSLVASELPLLIEDLLEHEKKELQKKQQLSAQLQPAQQQQQQQQQHSLLSAPGPAQAMSLPHEGSSPSLAGSQQQLSLGLAGARQPGLPQPLMPTQPPAHALQQRLAPSMAMVSNQGHMLSGQHGGQAGLVPQQSSQPVLSQKPMGTMPPSMCMKPQQLAMQQQLANSFFPDTDLDKFAAEDIIDPIAKAKMVALKGIKKVMAQGSIGVAPGMNRQQVSLLAQRLSGGPSSDLQNHVAAGSGQERSAGDPSQPRPNPPTFAQGVINEADQRQYEEWLFHTQQLLQMQLKVLEEQIGVHRKSRKALCAKQRTAKKAGREFPEADAEKLKLVTEQQSKIQKQLDQVRKQQKEHTNLMAEYRNKQQQQQQQQQQQQQQHSAVLALSPSQSPRLLTKLPGQLLPGHGLQPPQGPPGGQAGGLRLTPGGMALPGQPGGPFLNTALAQQQQQQHSGGAGSLAGPSGGFFPGNLALRSLGPDSRLLQERQLQLQQQRMQLAQKLQQQQQQQQQQQHLLGQVAIQQQQQQGPGVQTNQALGPKPQGLMPPSSHQGLLVQQLSPQPPQGPQGMLGPAQVAVLQQQHPGALGPQGPHRQVLMTQSRVLSSPQLAQQGQGLMGHRLVTAQQQQQQQQHQQQGSMAGLSHLQQSLMSHSGQPKLSAQPMGSLQQLQQQQQLQQQQQLQQQQQQQLQQQQQLQQQQLQQQQQQQQLQQQQQQQLQQQQQQLQQQQQQQQQQFQQQQQQQQMGLLNQSRTLLSPQQQQQQQVALGPGMPAKPLQHFSSPGALGPTLLLTGKEQNTVDPAVSSEATEGPSTHQGGPLAIGTTPESMATEPGEVKPSLSGDSQLLLVQPQPQPQPSSLQLQPPLRLPGQQQQQVSLLHTAGGGSHGQLGSGSSSEASSVPHLLAQPSVSLGDQPGSMTQNLLGPQQPMLERPMQNNTGPQPPKPGPVLQSGQGLPGVGIMPTVGQLRAQLQGVLAKNPQLRHLSPQQQQQLQALLMQRQLQQSQAVRQTPPYQEPGTQTSPLQGLLGCQPQLGGFPGPQTGPLQELGAGPRPQGPPRLPAPPGALSTGPVLGPVHPTPPPSSPQEPKRPSQLPSPSSQLPTEAQLPPTHPGTPKPQGPTLEPPPGRVSPAAAQLADTLFSKGLGPWDPPDNLAETQKPEQSSLVPGHLDQVNGQVVPEASQLSIKQEPREEPCALGAQSVKREANGEPIGAPGTSNHLLLAGPRSEAGHLLLQKLLRAKNVQLSTGRGSEGLRAEINGHIDSKLAGLEQKLQGTPSNKEDAAARKPLTPKPKRVQKASDRLVSSRKKLRKEDGVRASEALLKQLKQELSLLPLTEPAITANFSLFAPFGSGCPVNGQSQLRGAFGSGALPTGPDYYSQLLTKNNLSNPPTPPSSLPPTPPPSVQQKMVNGVTPSEELGEHPKDAASARDSERALRDTSEVKSLDLLAALPTPPHNQTEDVRMESDEDSDSPDSIVPASSPESILGEEAPRFPHLGSGRWEQEDRALSPVIPLIPRASIPVFPDTKPYGALGLEVPGKLPVTTWEKGKGSEVSVMLTVSAAAAKNLNGVMVAVAELLSMKIPNSYEVLFPESPARAGTEPKKGEAEGPGGKEKGLEGKSPDTGPDWLKQFDAVLPGYTLKSQLDILSLLKQESPAPEPPTQHSYTYNVSNLDVRQLSAPPPEEPSPPPSPLAPSPASPPTEPLVELPTEPLAEPPVPSPLPLASSPESARPKPRARPPEEGEDSRPPRLKKWKGVRWKRLRLLLTIQKGSGRQEDEREVAEFMEQLGTALRPDKVPRDMRRCCFCHEEGDGATDGPARLLNLDLDLWVHLNCALWSTEVYETQGGALMNVEVALHRGLLTKCSLCQRTGATSSCNRMRCPNVYHFACAIRAKCMFFKDKTMLCPMHKIKGPCEQELSSFAVFRRVYIERDEVKQIASIIQRGERLHMFRVGGLVFHAIGQLLPHQMADFHSATALYPVGYEATRIYWSLRTNNRRCCYRCSIGENNGRPEFVIKVIEQGLEDLVFTDASPQAVWNRIIEPVAAMRKEADMLRLFPEYLKGEELFGLTVHAVLRIAESLPGVESCQNYLFRYGRHPLMELPLMINPTGCARSEPKILTHYKRPHTLNSTSMSKAYQSTFTGETNTPYSKQFVHSKSSQYRRLRTEWKNNVYLARSRIQGLGLYAAKDLEKHTMVIEYIGTIIRNEVANRREKIYEEQNRGIYMFRINNEHVIDATLTGGPARYINHSCAPNCVAEVVTFDKEDKIIIISSRRIPKGEELTYDYQFDFEDDQHKIPCHCGAWNCRKWMN

>gi|10864041|ref|NP_067053.1| myeloid/lymphoid or mixed-lineage leukemia 3 isoform 1 [Homo sapiens]

MHNTVVLFSSSDKFTLNQDMCVVCGSFGQGAEGRLLACSQCGQCYHPYCVSIKITKVVLSKGWRCLECTVCEACGKATDPGRLLLCDDCDISYHTYCLDPPLQTVPKGGWKCKWCVWCRHCGATSAGLRCEWQNNYTQCAPCASLSSCPVCYRNYREEDLILQCRQCDRWMHAVCQNLNTEEEVENVADIGFDCSMCRPYMPASNVPSSDCCESSLVAQIVTKVKELDPPKTYTQDGVCLTESGMTQLQSLTVTVPRRKRSKPKLKLKIINQNSVAVLQTPPDIQSEHSRDGEMDDSREGELMDCDGKSESSPEREAVDDETKGVEGTDGVKKRKRKPYRPGIGGFMVRQRSRTGQGKTKRSVIRKDSSGSISEQLPCRDDGWSEQLPDTLVDESVSVTESTEKIKKRYRKRKNKLEETFPAYLQEAFFGKDLLDTSRQSKISLDNLSEDGAQLLYKTNMNTGFLDPSLDPLLSSSSAPTKSGTHGPADDPLADISEVLNTDDDILGIISDDLAKSVDHSDIGPVTDDPSSLPQPNVNQSSRPLSEEQLDGILSPELDKMVTDGAILGKLYKIPELGGKDVEDLFTAVLSPANTQPTPLPQPPPPTQLLPIHNQDAFSRMPLMNGLIGSSPHLPHNSLPPGSGLGTFSAIAQSSYPDARDKNSAFNPMASDPNNSWTSSAPTVEGENDTMSNAQRSTLKWEKEEALGEMATVAPVLYTNINFPNLKEEFPDWTTRVKQIAKLWRKASSQERAPYVQKARDNRAALRINKVQMSNDSMKRQQQQDSIDPSSRIDSELFKDPLKQRESEHEQEWKFRQQMRQKSKQQAKIEATQKLEQVKNEQQQQQQQQFGSQHLLVQSGSDTPSSGIQSPLTPQPGNGNMSPAQSFHKELFTKQPPSTPTSTSSDDVFVKPQAPPPPPAPSRIPIQDSLSQAQTSQPPSPQVFSPGSSNSRPPSPMDPYAKMVGTPRPPPVGHSFSRRNSAAPVENCTPLSSVSRPLQMNETTANRPSPVRDLCSSSTTNNDPYAKPPDTPRPVMTDQFPKSLGLSRSPVVSEQTAKGPIAAGTSDHFTKPSPRADVFQRQRIPDSYARPLLTPAPLDSGPGPFKTPMQPPPSSQDPYGSVSQASRRLSVDPYERPALTPRPIDNFSHNQSNDPYSQPPLTPHPAVNESFAHPSRAFSQPGTISRPTSQDPYSQPPGTPRPVVDSYSQSSGTARSNTDPYSQPPGTPRPTTVDPYSQQPQTPRPSTQTDLFVTPVTNQRHSDPYAHPPGTPRPGISVPYSQPPATPRPRISEGFTRSSMTRPVLMPNQDPFLQAAQNRGPALPGPLVRPPDTCSQTPRPPGPGLSDTFSRVSPSAARDPYDQSPMTPRSQSDSFGTSQTAHDVADQPRPGSEGSFCASSNSPMHSQGQQFSGVSQLPGPVPTSGVTDTQNTVNMAQADTEKLRQRQKLREIILQQQQQKKIAGRQEKGSQDSPAVPHPGPLQHWQPENVNQAFTRPPPPYPGNIRSPVAPPLGPRYAVFPKDQRGPYPPDVASMGMRPHGFRFGFPGGSHGTMPSQERFLVPPQQIQGSGVSPQLRRSVSVDMPRPLNNSQMNNPVGLPQHFSPQSLPVQQHNILGQAYIELRHRAPDGRQRLPFSAPPGSVVEASSNLRHGNFIPRPDFPGPRHTDPMRRPPQGLPNQLPVHPDLEQVPPSQQEQGHSVHSSSMVMRTLNHPLGGEFSEAPLSTSVPSETTSDNLQITTQPSDGLEEKLDSDDPSVKELDVKDLEGVEVKDLDDEDLENLNLDTEDGKVVELDTLDNLETNDPNLDDLLRSGEFDIIAYTDPELDMGDKKSMFNEELDLPIDDKLDNQCVSVEPKKKEQENKTLVLSDKHSPQKKSTVTNEVKTEVLSPNSKVESKCETEKNDENKDNVDTPCSQASAHSDLNDGEKTSLHPCDPDLFEKRTNRETAGPSANVIQASTQLPAQDVINSCGITGSTPVLSSLLANEKSDNSDIRPSGSPPPPTLPASPSNHVSSLPPFIAPPGRVLDNAMNSNVTVVSRVNHVFSQGVQVNPGLIPGQSTVNHSLGTGKPATQTGPQTSQSGTSSMSGPQQLMIPQTLAQQNRERPLLLEEQPLLLQDLLDQERQEQQQQRQMQAMIRQRSEPFFPNIDFDAITDPIMKAKMVALKGINKVMAQNNLGMPPMVMSRFPFMGQVVTGTQNSEGQNLGPQAIPQDGSITHQISRPNPPNFGPGFVNDSQRKQYEEWLQETQQLLQMQQKYLEEQIGAHRKSKKALSAKQRTAKKAGREFPEEDAEQLKHVTEQQSMVQKQLEQIRKQQKEHAELIEDYRIKQQQQCAMAPPTMMPSVQPQPPLIPGATPPTMSQPTFPMVPQQLQHQQHTTVISGHTSPVRMPSLPGWQPNSAPAHLPLNPPRIQPPIAQLPIKTCTPAPGTVSNANPQSGPPPRVEFDDNNPFSESFQERERKERLREQQERQRIQLMQEVDRQRALQQRMEMEQHGMVGSEISSSRTSVSQIPFYSSDLPCDFMQPLGPLQQSPQHQQQMGQVLQQQNIQQGSINSPSTQTFMQTNERRQVGPPSFVPDSPSIPVGSPNFSSVKQGHGNLSGTSFQQSPVRPSFTPALPAAPPVANSSLPCGQDSTITHGHSYPGSTQSLIQLYSDIIPEEKGKKKRTRKKKRDDDAESTKAPSTPHSDITAPPTPGISETTSTPAVSTPSELPQQADQESVEPVGPSTPNMAAGQLCTELENKLPNSDFSQATPNQQTYANSEVDKLSMETPAKTEEIKLEKAETESCPGQEEPKLEEQNGSKVEGNAVACPVSSAQSPPHSAGAPAAKGDSGNELLKHLLKNKKSSSLLNQKPEGSICSEDDCTKDNKLVEKQNPAEGLQTLGAQMQGGFGCGNQLPKTDGGSETKKQRSKRTQRTGEKAAPRSKKRKKDEEEKQAMYSSTDTFTHLKQVRQLSLLPLMEPIIGVNFAHFLPYGSGQFNSGNRLLGTFGSATLEGVSDYYSQLIYKQNNLSNPPTPPASLPPTPPPMACQKMANGFATTEELAGKAGVLVSHEVTKTLGPKPFQLPFRPQDDLLARALAQGPKTVDVPASLPTPPHNNQEELRIQDHCGDRDTPDSFVPSSSPESVVGVEVSRYPDLSLVKEEPPEPVPSPIIPILPSTAGKSSESRRNDIKTEPGTLYFASPFGPSPNGPRSGLISVAITLHPTAAENISSVVAAFSDLLHVRIPNSYEVSSAPDVPSMGLVSSHRINPGLEYRQHLLLRGPPPGSANPPRLVSSYRLKQPNVPFPPTSNGLSGYKDSSHGIAESAALRPQWCCHCKVVILGSGVRKSFKDLTLLNKDSRESTKRVEKDIVFCSNNCFILYSSTAQAKNSENKESIPSLPQSPMRETPSKAFHQYSNNISTLDVHCLPQLPEKASPPASPPIAFPPAFEAAQVEAKPDELKVTVKLKPRLRAVHGGFEDCRPLNKKWRGMKWKKWSIHIVIPKGTFKPPCEDEIDEFLKKLGTSLKPDPVPKDYRKCCFCHEEGDGLTDGPARLLNLDLDLWVHLNCALWSTEVYETQAGALINVELALRRGLQMKCVFCHKTGATSGCHRFRCTNIYHFTCAIKAQCMFFKDKTMLCPMHKPKGIHEQELSYFAVFRRVYVQRDEVRQIASIVQRGERDHTFRVGSLIFHTIGQLLPQQMQAFHSPKALFPVGYEASRLYWSTRYANRRCRYLCSIEEKDGRPVFVIRIVEQGHEDLVLSDISPKGVWDKILEPVACVRKKSEMLQLFPAYLKGEDLFGLTVSAVARIAESLPGVEACENYTFRYGRNPLMELPLAVNPTGCARSEPKMSAHVKRPHTLNSTSTSKSFQSTVTGELNAPYSKQFVHSKSSQYRKMKTEWKSNVYLARSRIQGLGLYAARDIEKHTMVIEYIGTIIRNEVANRKEKLYESQNRGVYMFRMDNDHVIDATLTGGPARYINHSCAPNCVAEVVTFERGHKIIISSSRRIQKGEELCYDYKFDFEDDQHKIPCHCGAVNCRKWMN

>gi|7662046|ref|NP_055542.1| histone-lysine N-methyltransferase MLL4 [Homo sapiens]

MAAAAGGGSCPGPGSARGRFPGRPRGAGGGGGRGGRGNGAERVRVALRRGGGATGPGGAEPGEDTALLRLLGLRRGLRRLRRLWAGPRVQRGRGRGRGRGWGPSRGCVPEEESSDGESDEEEFQGFHSDEDVAPSSLRSALRSQRGRAPRGRGRKHKTTPLPPPRLADVAPTPPKTPARKRGEEGTERMVQALTELLRRAQAPQAPRSRACEPSTPRRSRGRPPGRPAGPCRRKQQAVVVAEAAVTIPKPEPPPPVVPVKHQTGSWKCKEGPGPGPGTPRRGGQSSRGGRGGRGRGRGGGLPFVIKFVSRAKKVKMGQLSLGLESGQGQGQHEESWQDVPQRRVGSGQGGSPCWKKQEQKLDDEEEEKKEEEEKDKEGEEKEERAVAEEMMPAAEKEEAKLPPPPLTPPAPSPPPPLPPPSTSPPPPLCPPPPPPVSPPPLPSPPPPPAQEEQEESPPPVVPATCSRKRGRPPLTPSQRAEREAARAGPEGTSPPTPTPSTATGGPPEDSPTVAPKSTTFLKNIRQFIMPVVSARSSRVIKTPRRFMDEDPPKPPKVEVSPVLRPPITTSPPVPQEPAPVPSPPRAPTPPSTPVPLPEKRRSILREPTFRWTSLTRELPPPPPAPPPPPAPSPPPAPATSSRRPLLLRAPQFTPSEAHLKIYESVLTPPPLGAPEAPEPEPPPADDSPAEPEPRAVGRTNHLSLPRFAPVVTTPVKAEVSPHGAPALSNGPQTQAQLLQPLQALQTQLLPQALPPPQPQLQPPPSPQQMPPLEKARIAGVGSLPLSGVEEKMFSLLKRAKVQLFKIDQQQQQKVAASMPLSPGGQMEEVAGAVKQISDRGPVRSEDESVEAKRERPSGPESPVQGPRIKHVCRHAAVALGQARAMVPEDVPRLSALPLRDRQDLATEDTSSASETESVPSRSRRGKVEAAGPGGESEPTGSGGTLAHTPRRSLPSHHGKKMRMARCGHCRGCLRVQDCGSCVNCLDKPKFGGPNTKKQCCVYRKCDKIEARKMERLAKKGRTIVKTLLPWDSDESPEASPGPPGPRRGAGAGGPREEVVAHPGPEEQDSLLQRKSARRCVKQRPSYDIFEDSDDSEPGGPPAPRRRTPRENELPLPEPEEQSRPRKPTLQPVLQLKARRRLDKDALAPGPFASFPNGWTGKQKSPDGVHRVRVDFKEDCDLENVWLMGGLSVLTSVPGGPPMVCLLCASKGLHELVFCQVCCDPFHPFCLEEAERPLPQHHDTWCCRRCKFCHVCGRKGRGSKHLLECERCRHAYHPACLGPSYPTRATRKRRHWICSACVRCKSCGATPGKNWDVEWSGDYSLCPRCTQLYEKGNYCPICTRCYEDNDYESKMMQCAQCDHWVHAKCEGLSDEDYEILSGLPDSVLYTCGPCAGAAQPRWREALSGALQGGLRQVLQGLLSSKVVGPLLLCTQCGPDGKQLHPGPCGLQAVSQRFEDGHYKSVHSFMEDMVGILMRHSEEGETPDRRAGGQMKGLLLKLLESAFGWFDAHDPKYWRRSTRLPNGVLPNAVLPPSLDHVYAQWRQQEPETPESGQPPGDPSAAFQGKDPAAFSHLEDPRQCALCLKYGDADSKEAGRLLYIGQNEWTHVNCAIWSAEVFEENDGSLKNVHAAVARGRQMRCELCLKPGATVGCCLSSCLSNFHFMCARASYCIFQDDKKVFCQKHTDLLDGKEIVNPDGFDVLRRVYVDFEGINFKRKFLTGLEPDAINVLIGSIRIDSLGTLSDLSDCEGRLFPIGYQCSRLYWSTVDARRRCWYRCRILEYRPWGPREEPAHLEAAEENQTIVHSPAPSSEPPGGEDPPLDTDVLVPGAPERHSPIQNLDPPLRPDSGSAPPPAPRSFSGARIKVPNYSPSRRPLGGVSFGPLPSPGSPSSLTHHIPTVGDPDFPAPPRRSRRPSPLAPRPPPSRWASPPLKTSPQLRVPPPTSVVTALTPTSGELAPPGPAPSPPPPEDLGPDFEDMEVVSGLSAADLDFAASLLGTEPFQEEIVAAGAMGSSHGGPGDSSEEESSPTSRYIHFPVTVVSAPGLAPSATPGAPRIEQLDGVDDGTDSEAEAVQQPRGQGTPPSGPGVVRAGVLGAAGDRARPPEDLPSEIVDFVLKNLGGPGDGGAGPREESLPPAPPLANGSQPSQGLTASPADPTRTFAWLPGAPGVRVLSLGPAPEPPKPATSKIILVNKLGQVFVKMAGEGEPVPPPVKQPPLPPTISPTAPTSWTLPPGPLLGVLPVVGVVRPAPPPPPPPLTLVLSSGPASPPRQAIRVKRVSTFSGRSPPAPPPYKAPRLDEDGEASEDTPQVPGLGSGGFSRVRMKTPTVRGVLDLDRPGEPAGEESPGPLQERSPLLPLPEDGPPQVPDGPPDLLLESQWHHYSGEASSSEEEPPSPDDKENQAPKRTGPHLRFEISSEDGFSVEAESLEGAWRTLIEKVQEARGHARLRHLSFSGMSGARLLGIHHDAVIFLAEQLPGAQRCQHYKFRYHQQGEGQEEPPLNPHGAARAEVYLRKCTFDMFNFLASQHRVLPEGATCDEEEDEVQLRSTRRATSLELPMAMRFRHLKKTSKEAVGVYRSAIHGRGLFCKRNIDAGEMVIEYSGIVIRSVLTDKREKFYDGKGIGCYMFRMDDFDVVDATMHGNAARFINHSCEPNCFSRVIHVEGQKHIVIFALRRILRGEELTYDYKFPIEDASNKLPCNCGAKRCRRFLN

>gi|91199543|ref|NP_061152.3| histone-lysine N-methyltransferase MLL5 [Homo sapiens]

MSIVIPLGVDTAETSYLEMAAGSEPESVEASPVVVEKSNSYPHQLYTSSSHHSHSYIGLPYADHNYGARPPPTPPASPPPSVLISKNEVGIFTTPNFDETSSATTISTSEDGSYGTDVTRCICGFTHDDGYMICCDKCSVWQHIDCMGIDRQHIPDTYLCERCQPRNLDKERAVLLQRRKRENMSDGDTSATESGDEVPVELYTAFQHTPTSITLTASRVSKVNDKRRKKSGEKEQHISKCKKAFREGSRKSSRVKGSAPEIDPSSDGSNFGWETKIKAWMDRYEEANNNQYSEGVQREAQRIALRLGNGNDKKEMNKSDLNTNNLLFKPPVESHIQKNKKILKSAKDLPPDALIIEYRGKFMLREQFEANGYFFKRPYPFVLFYSKFHGLEMCVDARTFGNEARFIRRSCTPNAEVRHEIQDGTIHLYIYSIHSIPKGTEITIAFDFDYGNCKYKVDCACLKENPECPVLKRSSESMENINSGYETRRKKGKKDKDISKEKDTQNQNITLDCEGTTNKMKSPETKQRKLSPLRLSVSNNQEPDFIDDIEEKTPISNEVEMESEEQIAERKRKMTREERKMEAILQAFARLEKREKRREQALERISTAKTEVKTECKDTQIVSDAEVIQEQAKEENASKPTPAKVNRTKQRKSFSRSRTHIGQQRRRHRTVSMCSDIQPSSPDIEVTSQQNDIENTVLTIEPETETALAEIITETEVPALNKCPTKYPKTKKHLVNEWLSEKNEKTGKPSDGLSERPLRITTDPEVLATQLNSLPGLTYSPHVYSTPKHYIRFTSPFLSEKRRRKEPTENISGSCKKRWLKQALEEENSAILHRFNSPCQERSRSPAVNGENKSPLLLNDSCSLPDLTTPLKKRRFYQLLDSVYSETSTPTPSPYATPTHTDITPMDPSFATPPRIKSDDETCRNGYKPIYSPVTPVTPGTPGNTMHFENISSPESSPEIKRRTYSQEGYDRSSTMLTLGPFRNSNLTELGLQEIKTIGYTSPRSRTEVNRQCPGEKEPVSDLQLGLDAVEPTALHKTLETPAHDRAEPNSQLDSTHSGRGTMYSSWVKSPDRTGVNFSVNSNLRDLTPSHQLEVGGGFRISESKCLMQDDTRGMFMETTVFCTSEDGLVSGFGRTVNDNLIDGNCTPQNPPQKKKVSLLEYRKRQREARKSGSKTENFPLISVSPHASGSLSNNGDGCASSNDNGEQVDHTASLPLPTPATVYNATSEETSNNCPVKDATASEKNEPEVQWTASTSVEQVRERSYQRALLLSDHRKDKDSGGESPCVSCSPSHVQSSPSSHSNHIPQLQAKGPVPSFSELMEDPDPENPEPTTTNECPSPDTSQNTCKSPPKMSKPGSPGSVIPAQAHGKIFTKPDPQWDSTVSASEAENGVHLKTELQQKQLSNNNQALSKNHPPQTHVRNSSEQLSQKLPSVPTKLHCPPSPHLENPPKSSTPHTPVQHGYLSPKPPSQQLGSPYRPHHSQSPQVGTPQREPQRNFYPAAQNLPANTQQATSGTLFTQTPSGQSSATYSQFNQQSLNSTAPPPPPPPPPSSSYYQNQQPSANFQNYNQLKGSLSQQTVFTSGPNQALPGTTSQQTVPGHHVTPGHFLPSQNPTIHHQTAAAVVPPPPPPPPAPGPHLVQQPNSHQQHSVAHVVGPVHAVTPGSHIHSQTAGHHLPPPPPPPGPAPHHHPPPHPSTGLQGLQAQHQHVVNSAPPPPPPPPPSSVLASGHHTTSAQALHHPPHQGPPLFPSSAHPTVPPYPSQATHHTTLGPGPQHQPSGTGPHCPLPVTGPHLQPQGPNSIPTPTASGFCPHPGSVALPHGVQGPQQASPVPGQIPIHRAQVPPTFQNNYHGSGWH

>gi|27477095|ref|NP_758859.1| histone-lysine N-methyltransferase, H3 lysine-36 and H4 lysine-20 specific isoform a [Homo sapiens]

MPLKTRTALSDDPDSSTSTLGNMLELPGTSSSSTSQELPFCQPKKKSTPLKYEVGDLIWAKFKRRPWWPCRICSDPLINTHSKMKVSNRRPYRQYYVEAFGDPSERAWVAGKAIVMFEGRHQFEELPVLRRRGKQKEKGYRHKVPQKILSKWEASVGLAEQYDVPKGSKNRKCIPGSIKLDSEEDMPFEDCTNDPESEHDLLLNGCLKSLAFDSEHSADEKEKPCAKSRARKSSDNPKRTSVKKGHIQFEAHKDERRGKIPENLGLNFISGDISDTQASNELSRIANSLTGSNTAPGSFLFSSCGKNTAKKEFETSNGDSLLGLPEGALISKCSREKNKPQRSLVCGSKVKLCYIGAGDEEKRSDSISICTTSDDGSSDLDPIEHSSESDNSVLEIPDAFDRTENMLSMQKNEKIKYSRFAATNTRVKAKQKPLISNSHTDHLMGCTKSAEPGTETSQVNLSDLKASTLVHKPQSDFTNDALSPKFNLSSSISSENSLIKGGAANQALLHSKSKQPKFRSIKCKHKENPVMAEPPVINEECSLKCCSSDTKGSPLASISKSGKVDGLKLLNNMHEKTRDSSDIETAVVKHVLSELKELSYRSLGEDVSDSGTSKPSKPLLFSSASSQNHIPIEPDYKFSTLLMMLKDMHDSKTKEQRLMTAQNLVSYRSPGRGDCSTNSPVGVSKVLVSGGSTHNSEKKGDGTQNSANPSPSGGDSALSGELSASLPGLLSDKRDLPASGKSRSDCVTRRNCGRSKPSSKLRDAFSAQMVKNTVNRKALKTERKRKLNQLPSVTLDAVLQGDRERGGSLRGGAEDPSKEDPLQIMGHLTSEDGDHFSDVHFDSKVKQSDPGKISEKGLSFENGKGPELDSVMNSENDELNGVNQVVPKKRWQRLNQRRTKPRKRMNRFKEKENSECAFRVLLPSDPVQEGRDEFPEHRTPSASILEEPLTEQNHADCLDSAGPRLNVCDKSSASIGDMEKEPGIPSLTPQAELPEPAVRSEKKRLRKPSKWLLEYTEEYDQIFAPKKKQKKVQEQVHKVSSRCEEESLLARGRSSAQNKQVDENSLISTKEEPPVLEREAPFLEGPLAQSELGGGHAELPQLTLSVPVAPEVSPRPALESEELLVKTPGNYESKRQRKPTKKLLESNDLDPGFMPKKGDLGLSKKCYEAGHLENGITESCATSYSKDFGGGTTKIFDKPRKRKRQRHAAAKMQCKKVKNDDSSKEIPGSEGELMPHRTATSPKETVEEGVEHDPGMPASKKMQGERGGGAALKENVCQNCEKLGELLLCEAQCCGAFHLECLGLTEMPRGKFICNECRTGIHTCFVCKQSGEDVKRCLLPLCGKFYHEECVQKYPPTVMQNKGFRCSLHICITCHAANPANVSASKGRLMRCVRCPVAYHANDFCLAAGSKILASNSIICPNHFTPRRGCRNHEHVNVSWCFVCSEGGSLLCCDSCPAAFHRECLNIDIPEGNWYCNDCKAGKKPHYREIVWVKVGRYRWWPAEICHPRAVPSNIDKMRHDVGEFPVLFFGSNDYLWTHQARVFPYMEGDVSSKDKMGKGVDGTYKKALQEAAARFEELKAQKELRQLQEDRKNDKKPPPYKHIKVNRPIGRVQIFTADLSEIPRCNCKATDENPCGIDSECINRMLLYECHPTVCPAGGRCQNQCFSKRQYPEVEIFRTLQRGWGLRTKTDIKKGEFVNEYVGELIDEEECRARIRYAQEHDITNFYMLTLDKDRIIDAGPKGNYARFMNHCCQPNCETQKWSVNGDTRVGLFALSDIKAGTELTFNYNLECLGNGKTVCKCGAPNCSGFLGVRPKNQPIATEEKSKKFKKKQQGKRRTQGEITKEREDECFSCGDAGQLVSCKKPGCPKVYHADCLNLTKRPAGKWECPWHQCDICGKEAASFCEMCPSSFCKQHREGMLFISKLDGRLSCTEHDPCGPNPLEPGEIREYVPPPVPLPPGPSTHLAEQSTGMAAQAPKMSDKPPADTNQMLSLSKKALAGTCQRPLLPERPLERTDSRPQPLDKVRDLAGSGTKSQSLVSSQRPLDRPPAVAGPRPQLSDKPSPVTSPSSSPSVRSQPLERPLGTADPRLDKSIGAASPRPQSLEKTSVPTGLRLPPPDRLLITSSPKPQTSDRPTDKPHASLSQRLPPPEKVLSAVVQTLVAKEKALRPVDQNTQSKNRAALVMDLIDLTPRQKERAASPHQVTPQADEKMPVLESSSWPASKGLGHMPRAVEKGCVSDPLQTSGKAAAPSEDPWQAVKSLTQARLLSQPPAKAFLYEPTTQASGRASAGAEQTPGPLSQSPGLVKQAKQMVGGQQLPALAAKSGQSFRSLGKAPASLPTEEKKLVTTEQSPWALGKASSRAGLWPIVAGQTLAQSCWSAGSTQTLAQTCWSLGRGQDPKPEQNTLPALNQAPSSHKCAESEQK

>gi|20071601|gb|AAH27450.1| SETD1A protein [Homo sapiens]

LFLGEEAEPGTEVDLAVLADLALTPARRGLPALPAVEDSEATETSDEAERPRPLLSHILLEHNYALAVKPTPPAPALRPPEPVPAPAALFSSPADEVLEAPEVVVAEAEEPKPQQLQQQREEGEEEGEEEGEEEEEESSDSSSSSDGEGALRRRSLRSHARRRRPPPPPPPPPPRAYEPRSEFEQMTILYDIWNSGLDSEDMSYLRLTYERLLQQTSGADWLNDTHWVHHTITNLTTPKRKRRPQDGPREHQTGSARSEGYYPISKKEKDKYLDVCPVSARQLEGVDTQGTNRVLSERRSEQRRLLSAIGTSAIMDSDLLKLNQLKFRKKKLRFGRSRIHEWGLFAMEPIAADEMVIEYVGQNIRQMVADMREKRYVQEGIGSSYLFRVDHDTIIDATKCGNLARFINHCCTPNCYAKVITIESQKKIVIYSKQPIGVDEEITYDYKFPLEDNKIPCLCGTESCRGSLN

>gi|5689489|dbj|BAA83028.1| KIAA1076 protein [Homo sapiens]

EFESSSESSPSSSEDEEEVVAREEEEEEEEEEMVAEESMASAGPEDFEQDGEEAALAPGAPAVDSLGMEEEVDIETEAVAPEERPSMLDEPPLPVGVEEPADSREPPEEPGLSQEGAMLLSPEPPAKEVEARPPLSPERAPEHDLEVEPEPPMMLPLPLQPPLPPPRPPRPPSPPPEPETTDASHPSVPPEPLAEDHPPHTPGLCGSLAKSQSTETVPATPGGEPPLSGGSSGLSLSSPQVPGSPFSYPAPSPSLSSGGLPRTPGRDFSFTPTFSEPSGPLLLPVCPLPTGRRDERSGPLASPVLLETGLPLPLPLPLPLPLALPAVLRAQARAPTPLPPLLPAPLASCPPPMKRKPGRPRRSPPSMLSLDGPLVRPPAGAALGRELLLLPGQPQTPVFPSTHDPRTVTLDFRNAGIPAPPPPLPPQPPPPPPPPPVEPTKLPFKELDNQWPSEAIPPGPRGRDEVTEEYMELAKSRGPWRRPPKKRHEDLVPPAGSPELSPPQPLFRPRSEFEEMTILYDIWNGGIDEEDIRFLCVTYERLLQQDNGMDWLNDTLWVYHPSTSLSSAKKKKRDDGIREHVTGCARSEGFYTIDKKDKLRYLNSSRASTDEPPADTQGMSIPAQPHASTRAGSERRSEQRRLLSSFTGSCDSDLLKFNQLKFRKKKLKFCKSHIHDWGLFAMEPIAADEMVIEYVGQNIRQVIADMREKRYEDEGIGSSYMFRVDHDTIIDATKCGNFARFINHSCNPNCYAKVITVESQKKIVIYSKQHINVNEEITYDYKFPIEDVKIPCLCGSENCRGTLN

>gi|197313748|ref|NP_054878.5| histone-lysine N-methyltransferase SETD2 [Homo sapiens]

MKQLQPQPPPKMGDFYDPEHPTPEEEENEAKIENVQKTGFIKGPMFKGVASSRFLPKGTKTKVNLEEQGRQKVSFSFSLTKKTLQNRFLTALGNEKQSDTPNPPAVPLQVDSTPKMKMEIGDTLSTAEESSPPKSRVELGKIHFKKHLLHVTSRPLLATTTAVASPPTHAAPLPAVIAESTTVDSPPSSPPPPPPPAQATTLSSPAPVTEPVALPHTPITVLMAAPVPLPVDVAVRSLKEPPIIIVPESLEADTKQDTISNSLEEHVTQILNEQADISSKKEDSHIGKDEEIPDSSKISLSCKKTGSKKKSSQSEGIFLGSESDEDSVRTSSSQRSHDLKFSASIEKERDFKKSSAPLKSEDLGKPSRSKTDRDDKYFSYSKLERDTRYVSSRCRSERERRRSRSHSRSERGSRTNLSYSRSERSHYYDSDRRYHRSSPYRERTRYSRPYTDNRARESSDSEEEYKKTYSRRTSSHSSSYRDLRTSSYSKSDRDCKTETSYLEMERRGKYSSKLERESKRTSENEAIKRCCSPPNELGFRRGSSYSKHDSSASRYKSTLSKPIPKSDKFKNSFCCTELNEEIKQSHSFSLQTPCSKGSELRMINKNPEREKAGSPAPSNRLNDSPTLKKLDELPIFKSEFITHDSHDSIKELDSLSKVKNDQLRSFCPIELNINGSPGAESDLATFCTSKTDAVLMTSDDSVTGSELSPLVKACMLSSNGFQNISRCKEKDLDDTCMLHKKSESPFRETEPLVSPHQDKLMSMPVMTVDYSKTVVKEPVDTRVSCCKTKDSDIYCTLNDSNPSLCNSEAENIEPSVMKISSNSFMNVHLESKPVICDSRNLTDHSKFACEEYKQSIGSTSSASVNHFDDLYQPIGSSGIASSLQSLPPGIKVDSLTLLKCGENTSPVLDAVLKSKKSSEFLKHAGKETIVEVGSDLPDSGKGFASRENRRNNGLSGKCLQEAQEEGNSILPERRGRPEISLDERGEGGHVHTSDDSEVVFSSCDLNLTMEDSDGVTYALKCDSSGHAPEIVSTVHEDYSGSSESSNDESDSEDTDSDDSSIPRNRLQSVVVVPKNSTLPMEETSPCSSRSSQSYRHYSDHWEDERLESRRHLYEEKFESIASKACPQTDKFFLHKGTEKNPEISFTQSSRKQIDNRLPELSHPQSDGVDSTSHTDVKSDPLGHPNSEETVKAKIPSRQQEELPIYSSDFEDVPNKSWQQTTFQNRPDSRLGKTELSFSSSCEIPHVDGLHSSEELRNLGWDFSQEKPSTTYQQPDSSYGACGGHKYQQNAEQYGGTRDYWQGNGYWDPRSGRPPGTGVVYDRTQGQVPDSLTDDREEEENWDQQDGSHFSDQSDKFLLSLQKDKGSVQAPEISSNSIKDTLAVNEKKDFSKNLEKNDIKDRGPLKKRRQEIESDSESDGELQDRKKVRVEVEQGETSVPPGSALVGPSCVMDDFRDPQRWKECAKQGKMPCYFDLIEENVYLTERKKNKSHRDIKRMQCECTPLSKDERAQGEIACGEDCLNRLLMIECSSRCPNGDYCSNRRFQRKQHADVEVILTEKKGWGLRAAKDLPSNTFVLEYCGEVLDHKEFKARVKEYARNKNIHYYFMALKNDEIIDATQKGNCSRFMNHSCEPNCETQKWTVNGQLRVGFFTTKLVPSGSELTFDYQFQRYGKEAQKCFCGSANCRGYLGGENRVSIRAAGGKMKKERSRKKDSVDGELEALMENGEGLSDKNQVLSLSRLMVRIETLEQKLTCLELIQNTHSQSCLKSFLERHGLSLLWIWMAELGDGRESNQKLQEEIIKTLEHLPIPTKNMLEESKVLPIIQRWSQTKTAVPPLSEGDGYSSENTSRAHTPLNTPDPSTKLSTEADTDTPKKLMFRRLKIISENSMDSAISDATSELEGKDGKEDLDQLENVPVEEEEELQSQQLLPQQLPECKVDSETNIEASKLPTSEPEADAEIEPKESNGTKLEEPINEETPSQDEEEGVSDVESERSQEQPDKTVDISDLATKLLDSWKDLKEVYRIPKKSQTEKENTTTERGRDAVGFRDQTPAPKTPNRSRERDPDKQTQNKEKRKRRSSLSPPSSAYERGTKRPDDRYDTPTSKKKVRIKDRNKLSTEERRKLFEQEVAQREAQKQQQQMQNLGMTSPLPYDSLGYNAPHHPFAGYPPGYPMQAYVDPSNPNAGKVLLPTPSMDPVCSPAPYDHAQPLVGHSTEPLSAPPPVPVVPHVAAPVEVSSSQYVAQSDGVVHQDSSVAVLPVPAPGPVQGQNYSVWDSNQQSVSVQQQYSPAQSQATIYYQGQTCPTVYGVTSPYSQTTPPIVQSYAQPSLQYIQGQQIFTAHPQGVVVQPAAAVTTIVAPGQPQPLQPSEMVVTNNLLDLPPPSPPKPKTIVLPPNWKTARDPEGKIYYYHVITRQTQWDPPTWESPGDDASLEHEAEMDLGTPTYDENPMKASKKPKTAEADTSSELAKKSKEVFRKEMSQFIVQCLNPYRKPDCKVGRITTTEDFKHLARKLTHGVMNKELKYCKNPEDLECNENVKHKTKEYIKKYMQKFGAVYKPKEDTELE

>gi|12698059|dbj|BAB21848.1| KIAA1757 protein [Homo sapiens]

FKKSYFQDHNYGAPPPPTPPASPPVQTIIPRSDLNGLPSPVEERCGDSPNSEGETVPTWCPCGLSQDGFLLNCDKCRGMSRGKVIRLHRRKQDNISGGDSSATESWDEELSPSTVLYTATQHTPTSITLTVRRTKPKKRKKSPEKGRAAPKTKKIKNSPSEAQNLDENTTEGWENRIRLWTDQYEEAFTNQYSADVQNALEQHLHSSKEFVGKPTILDTINKTELACNNTVIGSQMQLQLGRVTRVQKHRKILRAARDLALDTLIIEYRGKVMLRQQFEVNGHFFKKPYPFVLFYSKFNGVEMCVDARTFGNDARFIRRSCTPNAEVRHMIADGMIHLCIYAVSAITKDAEVTIAFDYEYSNCNYKVDCACHKGNRNCPIQKRNPNATELPLLPPPPSLPTIGAETRRRKARRKELEMEQQNEASEENNDQQSQEVPEKVTVSSDHEEVDNPEEKPEEEKEEVIDDQENLAHSRRTREDRKVEAIMHAFENLEKRKKRRDQPLEQSNSDVEITTTTSETPVGEETKTEAPESEVSNSVSNVTIPSTPQSVGVNTRRSSQAGDIAAEKLVPKPPPAKPSRPRPKSRISRYRTSSAQRLKRQKQANAQQAELSQAALEEGGSNSLVTPTEAGSLDSSGENRPLTGSDPTVVSITGSHVNRAASKYPKTKKYLVTEWLNDKAEKQECPVECPLRITTDPTVLATTLNMLPGLIHSPLICTTPKHYIRFGSPFIPERRRRPLLPDGTFSSCKKRWIKQALEEGMTQTSSVPQETRTQHLYQSNENSSSSSICKDNADLLSPLKKWKSRYLMEQNVTKLLRPLSPVTPPPPNSGSKSPQLATPGSSHPGEEECRNGYSLMFSPVTSLTTASRCNTPLQFELCHRKDLDLAKVGYLDSNTNSCADRPSLLNSGHSDLAPHPSLGPTSETGFPSRSGDGHQTLVRNSDQAFRTEFNLMYAYSPLNAMPRADGLYRGSPLVGDRKPLHLDGGYCSPAEGFSSRYEHGLMKDLSRGSLSPGGERACEGVPSAPQNPPQRKKVSLLEYRKRKQEAKENSAGGGGDSAQSKSKSAGAGQGSSNSVSDTGAHGVQGSSARTPSSPHKKFSPSHSSMSHLEAVSPSDSRGTSSSHCRPQENISSRWMVPTSVERLREGGSIPKVLRSSVRVAQKGEPSPTWESNITEKDSDPADGEGPETLSSALSKGATVYSPSRYSYQLLQCDSPRTESQSLLQQSSSPFRGHPTQSPGYSYRTTALRPGNPPSHGSSESSLSSTSYSSPAHPVSTDSLAPFTGTPGYFSSQPHSGNSTGSNLPRRSCPSSAASPTLQGPSDSPTSDSVSQSSTGTLSSTSFPQNSRSSLPSDLRTISLPSAGQSAVYQASRVSAVSNSQHYPHRGSGGVHQYRLQPLQGSGVKTQTGLS

>gi|238550105|ref|NP_079136.2| N-lysine methyltransferase SETD6 isoform b [Homo sapiens]

MATQAKRPRVAGPVDGGDLDPVACFLSWCRRVGLELSPKVAVSRQGTVAGYGMVARESVQAGELLFVVPRAALLSQHTCSIGGLLERERVALQSQSGWVPLLLALLHELQAPASRWRPYFALWPELGRLEHPMFWPEEERRCLLQGTGVPEAVEKDLANIRSEYQSIVLPFMEAHPDLFSLRVRSLELYHQLVALVMAYSFQEPLEEEEDEKEPNSPVMVPAADILNHLANHNANLEYSANCLRMVATQPIPKGHEIFNTYGQMANWQLIHMYGFVEPYPDNTDDTADIQMVTVREAALQGTKTEAERHLVYERWDFLCKLEMVGEEGAFVIGREEVLTEEELTTTLKVLCMPAEEFRELKDQDGGGDDKREEGSLTITNIPKLKASWRQLLQNSVLLTLQTYATDLKTDQGLLSNKEVYAKLSWREQQALQVRYGQKMILHQLLELTS

>gi|18139549|ref|NP_085151.1| histone-lysine N-methyltransferase SETD7 [Homo sapiens]

MDSDDEMVEEAVEGHLDDDGLPHGFCTVTYSSTDRFEGNFVHGEKNGRGKFFFFDGSTLEGYYVDDALQGQGVYTYEDGGVLQGTYVDGELNGPAQEYDTDGRLIFKGQYKDNIRHGVCWIYYPDGGSLVGEVNEDGEMTGEKIAYVYPDERTALYGKFIDGEMIEGKLATLMSTEEGRPHFELMPGNSVYHFDKSTSSCISTNALLPDPYESERVYVAESLISSAGEGLFSKVAVGPNTVMSFYNGVRITHQEVDSRDWALNGNTLSLDEETVIDVPEPYNHVSKYCASLGHKANHSFTPNCIYDMFVHPRFGPIKCIRTLRAVEADEELTVAYGYDHSPPGKSGPEAPEWYQVELKAFQATQQK

>gi|45356743|ref|NP_065115.3| N-lysine methyltransferase SETD8 [Homo sapiens]

MARGRKMSKPRAVEAAAAAAAVAATAPGPEMVERRGPGRPRTDGENVFTGQSKIYSYMSPNKCSGMRFPLQEENSVTHHEVKCQGKPLAGIYRKREEKRNAGNAVRSAMKSEEQKIKDARKGPLVPFPNQKSEAAEPPKTPPSSCDSTNAAIAKQALKKPIKGKQAPRKKAQGKTQQNRKLTDFYPVRRSSRKSKAELQSEERKRIDELIESGKEEGMKIDLIDGKGRGVIATKQFSRGDFVVEYHGDLIEITDAKKREALYAQDPSTGCYMYYFQYLSKTYCVDATRETNRLGRLINHSKCGNCQTKLHDIDGVPHLILIASRDIAAGEELLYDYGDRSKASIEAHPWLKH

>gi|224177469|ref|NP_036564.3| histone-lysine N-methyltransferase SETDB1 isoform 2 [Homo sapiens]

MSSLPGCIGLDAATATVESEEIAELQQAVVEELGISMEELRHFIDEELEKMDCVQQRKKQLAELETWVIQKESEVAHVDQLFDDASRAVTNCESLVKDFYSKLGLQYRDSSSEDESSRPTEIIEIPDEDDDVLSIDSGDAGSRTPKDQKLREAMAALRKSAQDVQKFMDAVNKKSSSQDLHKGTLSQMSGELSKDGDLIVSMRILGKKRTKTWHKGTLIAIQTVGPGKKYKVKFDNKGKSLLSGNHIAYDYHPPADKLYVGSRVVAKYKDGNQVWLYAGIVAETPNVKNKLRFLIFFDDGYASYVTQSELYPICRPLKKTWEDIEDISCRDFIEEYVTAYPNRPMVLLKSGQLIKTEWEGTWWKSRVEEVDGSLVRILFLDDKRCEWIYRGSTRLEPMFSMKTSSASALEKKQGQLRTRPNMGAVRSKGPVVQYTQDLTGTGTQFKPVEPPQPTAPPAPPFPPAPPLSPQAGDSDLESQLAQSRKQVAKKSTSFRPGSVGSGHSSPTSPALSENVSGGKPGINQTYRSPLGSTASAPAPSALPAPPAPPVFHGMLERAPAEPSYRAPMEKLFYLPHVCSYTCLSRVRPMRNEQYRGKNPLLVPLLYDFRRMTARRRVNRKMGFHVIYKTPCGLCLRTMQEIERYLFETGCDFLFLEMFCLDPYVLVDRKFQPYKPFYYILDITYGKEDVPLSCVNEIDTTPPPQVAYSKERIPGKGVFINTGPEFLVGCDCKDGCRDKSKCACHQLTIQATACTPGGQINPNSGYQYKRLEECLPTGVYECNKRCKCDPNMCTNRLVQHGLQVRLQLFKTQNKGWGIRCLDDIAKGSFVCIYAGKILTDDFADKEGLEMGDEYFANLDHIESVENFKEGYESDAPCSSDSSGVDLKDQEDGNSGTEDPEESNDDSSDDNFCKDEDFSTSSVWRSYATRRQTRGQKENGLSETTSKDSHPPDLGPPHIPVPPSIPVGGCNPPSSEETPKNKVASWLSCNSVSEGGFADSDSHSSFKTNEGGEGRAGGSRMEAEKASTSGLGIKDEGDIKQAKKEDTDDRNKMSVVTESSRNYGYNPSPVKPEGLRRPPSKTSMHQSRRLMASAQSNPDDVLTLSSSTESEGESGTSRKPTAGQTSATAVDSDDIQTISSGSEGDDFEDKKNMTGPMKRQVAVKSTRGFALKSTHGIAIKSTNMASVDKGESAPVRKNTRQFYDGEESCYIIDAKLEGNLGRYLNHSCSPNLFVQNVFVDTHDLRFPWVAFFASKIRAGTELTWDYNYEVGSVEGKELLCCCGAIECRGRLL

>gi|238624095|ref|NP_114121.2| histone-lysine N-methyltransferase SETDB2 isoform a [Homo sapiens]

MGEKNGDAKTFWMELEDDGKVDFIFEQVQNVLQSLKQKIKDGSATNKEYIQAMILVNEATIINSSTSIKGASQKEVNAQSSDPMPVTQKEQENKSNAFPSTSCENSFPEDCTFLTTENKEILSLEDKVVDFREKDSSSNLSYQSHDCSGACLMKMPLNLKGENPLQLPIKCHFQRRHAKTNSHSSALHVSYKTPCGRSLRNVEEVFRYLLETECNFLFTDNFSFNTYVQLARNYPKQKEVVSDVDISNGVESVPISFCNEIDSRKLPQFKYRKTVWPRAYNLTNFSSMFTDSCDCSEGCIDITKCACLQLTARNAKTSPLSSDKITTGYKYKRLQRQIPTGIYECSLLCKCNRQLCQNRVVQHGPQVRLQVFKTEQKGWGVRCLDDIDRGTFVCIYSGRLLSRANTEKSYGIDENGRDENTMKNIFSKKRKLEVACSDCEVEVLPLGLETHPRTAKTEKCPPKFSNNPKELTVETKYDNISRIQYHSVIRDPESKTAIFQHNGKKMEFVSSESVTPEDNDGFKPPREHLNSKTKGAQKDSSSNHVDEFEDNLLIESDVIDITKYREETPPRSRCNQATTLDNQNIKKAIEVQIQKPQEGRSTACQRQQVFCDEELLSETKNTSSDSLTKFNKGNVFLLDATKEGNVGRFLNHSCCPNLLVQNVFVETHNRNFPLVAFFTNRYVKARTELTWDYGYEAGTVPEKEIFCQCGVNKCRKKIL

>gi|194306650|ref|NP_006506.3| histone-lysine N-methyltransferase SETMAR isoform 1 [Homo sapiens]

MFAEAAKTTRPCGMAEFKEKPEAPTEQLDVACGQENLPVGAWPPGAAPAPFQYTPDHVVGPGADIDPTQITFPGCICVKTPCLPGTCSCLRHGENYDDNSCLRDIGSGGKYAEPVFECNVLCRCSDHCRNRVVQKGLQFHFQVFKTHKKGWGLRTLEFIPKGRFVCEYAGEVLGFSEVQRRIHLQTKSDSNYIIAIREHVYNGQVMETFVDPTYIGNIGRFLNHSCEPNLLMIPVRIDSMVPKLALFAAKDIVPEEELSYDYSGRYLNLTVSEDKERLDHGKLRKPCYCGAKSCTAFLPFDSSLYCPVEKSNISCGNEKEPSMCGSAPSVFPSCKRLTLETMKMMLDKKQIRAIFLFEFKMGRKAAETTRNINNAFGPGTANERTVQWWFKKFCKGDESLEDEERSGRPSEVDNDQLRAIIEADPLTTTREVAEELNVNHSTVVRHLKQIGKVKKLDKWVPHELTENQKNRRFEVSSSLILRNHNEPFLDRIVTCDEKWILYDNRRRSAQWLDQEEAPKHFPKPILHPKKVMVTIWWSAAGLIHYSFLNPGETITSEKYAQEIDEMNQKLQRLQLALVNRKGPILLHDNARPHVAQPTLQKLNELGYEVLPHPPYSPDLLPTNYHVFKHLNNFLQGKRFHNQQDAENAFQEFVESQSTDFYATGINQLISRWQKCVDCNGSYFD

>gi|38093643|ref|NP_938015.1| histone-lysine N-methyltransferase SMYD1 [Homo sapiens]

MTIGRMENVEVFTAEGKGRGLKATKEFWAADIIFAERAYSAVVFDSLVNFVCHTCFKRQEKLHRCGQCKFAHYCDRTCQKDAWLNHKNECSAIKRYGKVPNENIRLAARIMWRVEREGTGLTEGCLVSVDDLQNHVEHFGEEEQKDLRVDVDTFLQYWPPQSQQFSMQYISHIFGVINCNGFTLSDQRGLQAVGVGIFPNLGLVNHDCWPNCTVIFNNGNHEAVKSMFHTQMRIELRALGKISEGEELTVSYIDFLNVSEERKRQLKKQYYFDCTCEHCQKKLKDDLFLGVKDNPKPSQEVVKEMIQFSKDTLEKIDKARSEGLYHEVVKLCRECLEKQEPVFADTNIYMLRMLSIVSEVLSYLQAFEEASFYARRMVDGYMKLYHPNNAQLGMAVMRAGLTNWHAGNIEVGHGMICKAYAILLVTHGPSHPITKDLEAMRVQTEMELRMFRQNEFMYYKMREAALNNQPMQVMAEPSNEPSPALFHKKQ

>gi|188035871|ref|NP_064582.2| N-lysine methyltransferase SMYD2 [Homo sapiens]

MRAEGLGGLERFCSPGKGRGLRALQPFQVGDLLFSCPAYAYVLTVNERGNHCEYCFTRKEGLSKCGRCKQAFYCNVECQKEDWPMHKLECSPMVVFGENWNPSETVRLTARILAKQKIHPERTPSEKLLAVKEFESHLDKLDNEKKDLIQSDIAALHHFYSKHLGFPDNDSLVVLFAQVNCNGFTIEDEELSHLGSAIFPDVALMNHSCCPNVIVTYKGTLAEVRAVQEIKPGEEVFTSYIDLLYPTEDRNDRLRDSYFFTCECQECTTKDKDKAKVEIRKLSDPPKAEAIRDMVRYARNVIEEFRRAKHYKSPSELLEICELSQEKMSSVFEDSNVYMLHMMYQAMGVCLYMQDWEGALQYGQKIIKPYSKHYPLYSLNVASMWLKLGRLYMGLEHKAAGEKALKKAIAIMEVAHGKDHPYISEIKQEIESH

>gi|12232401|ref|NP_073580.1| histone-lysine N-methyltransferase SMYD3 isoform 2 [Homo sapiens]

MRCSQCRVAKYCSAKCQKKAWPDHKRECKCLKSCKPRYPPDSVRLLGRVVFKLMDGAPSESEKLYSFYDLESNINKLTEDKKEGLRQLVMTFQHFMREEIQDASQLPPAFDLFEAFAKVICNSFTICNAEMQEVGVGLYPSISLLNHSCDPNCSIVFNGPHLLLRAVRDIEVGEELTICYLDMLMTSEERRKQLRDQYCFECDCFRCQTQDKDADMLTGDEQVWKEVQESLKKIEELKAHWKWEQVLAMCQAIISSNSERLPDINIYQLKVLDCAMDACINLGLLEEALFYGTRTMEPYRIFFPGSHPVRGVQVMKVGKLQLHQGMFPQAMKNLRLAFDIMRVTHGREHSLIEDLILLLEECDANIRAS

>gi|23272879|gb|AAH35077.1| SET and MYND domain containing 4 [Homo sapiens]

MDLPVDEWKSYLLQKWASLPTSVQVTISTAETLRDIFLHSSSLLQPEDELFLKRLSKGYLVGKDSDAPLFYREEGNKKFQEKDYTGAAVLYSKGVSHSRPNTEDMSLCHANRSAALFHLGQYETCLKDINIAQTHGYPERLQPKIMLRKAECLVALGRLQEASQTISDLERNFTATPALADVLPQTLQRNLHRLKMKMQEKDSLTESFPAALAKTLEDAALREENEQLSNASSSIGLCVDPLKGRCLVATKDILPGELLVQEDAFVSVLNPGELPPPHHGLDSKWDTRVTNGDLYCHRCLKHTLATVPCDGCSYAKYCSQECLQQVWELYHRTECPLGGLLLTLGVFCHIALRLTLLVGFEDVRKIITKLCDKISNKDICLPESNNQVKTLNYGLGESEKNGNIVETPIPGCDINGKYENNYNAVFNLLPHTENHSPEHKFLCALCVSALCRQLEAASLQAIPTERIVNSSQLKAAVTPELCPDVTIWGVAMLRHMLQLQCNAQAMTTIQHTGPKGSIVTDSRQVRLATGIFPVISLLNHSCSPNTSVSFISTVATIRASQRIRKGQEILHCYGPHKSRMGVAERQQKLRSQYFFDCACPACQTEAHRMAAGPRWEAFCCNSCGAPMQGDDVLRCGSRSCAESAVSRDHLVSRLQDLQQQVRVAQKLLRDGELERAVQRLSGCQRDAESFLWAEHAVVGEIADGLARACAALGDWQKSATHLQRSLCVVEVRHGPSSVEMGHELFKLAQIFFNGFAVPEALSTIQKAEEVLSLHCGPWDDEIQELQKMKSCLLDLPPTPVGPAL

>gi|1245372|gb|AAB38131.1| NN8-4AG, partial [Homo sapiens]

AVFSFCVGVAGRARVSVEVRFVSSAKGKGLFATQLIRKGETIFVERPLVAAQFLWNALYRYRACDHCLRALEKAEENAQRLTGKPGQVLPHPELCTVRKDLHQNCPHCQVMYCSAECRLAATEQYHQVLCPGPSQDDPLHPLNKLQEAWRSIHYPPETASIMLMARMVATVKQAKDKDRWIRLFSQFCNKTANEEEEIVHKLLGDKFKGQLELLRRLFTEALYEEAVSQWFTPDGFRSLFALVGTNGQGIGTSSLSQWVHACDTLELKPQDREHVDAFIDQLYKDIEAATGEFLNCEGSGLFVLQSCCNHSCVPNAETSFPENNFLLHVTALEDIKPGEEICISYLDCCQRERSRHSRHKILRENYLFVCSCPKCLAEADEPNVTSEEEEEEEEEEEGEPEDAELGDEMTDV

>gi|4507321|ref|NP_003164.1| histone-lysine N-methyltransferase SUV39H1 [Homo sapiens]

MAENLKGCSVCCKSSWNQLQDLCRLAKLSCPALGISKRNLYDFEVEYLCDYKKIREQEYYLVKWRGYPDSESTWEPRQNLKCVRILKQFHKDLERELLRRHHRSKTPRHLDPSLANYLVQKAKQRRALRRWEQELNAKRSHLGRITVENEVDLDGPPRAFVYINEYRVGEGITLNQVAVGCECQDCLWAPTGGCCPGASLHKFAYNDQGQVRLRAGLPIYECNSRCRCGYDCPNRVVQKGIRYDLCIFRTDDGRGWGVRTLEKIRKNSFVMEYVGEIITSEEAERRGQIYDRQGATYLFDLDYVEDVYTVDAAYYGNISHFVNHSCDPNLQVYNVFIDNLDERLPRIAFFATRTIRAGEELTFDYNMQVDPVDMESTRMDSNFGLAGLPGSPKKRVRIECKCGTESCRKYLF

>gi|13375930|ref|NP_078946.1| histone-lysine N-methyltransferase SUV39H2 isoform 2 [Homo sapiens]

MEYYLVKWKGWPDSTNTWEPLQNLKCPLLLQQFSNDKHNYLSQVKKGKAITPKDNNKTLKPAIAEYIVKKAKQRIALQRWQDELNRRKNHKGMIFVENTVDLEGPPSDFYYINEYKPAPGISLVNEATFGCSCTDCFFQKCCPAEAGVLLAYNKNQQIKIPPGTPIYECNSRCQCGPDCPNRIVQKGTQYSLCIFRTSNGRGWGVKTLVKIKRMSFVMEYVGEVITSEEAERRGQFYDNKGITYLFDLDYESDEFTVDAARYGNVSHFVNHSCDPNLQVFNVFIDNLDTRLPRIALFSTRTINAGEELTFDYQMKGSGDISSDSIDHSPAKKRVRTVCKCGAVTCRGYLN

>gi|50659084|ref|NP_057112.3| histone-lysine N-methyltransferase SUV420H1 isoform 2 [Homo sapiens]

MKWLGESKNMVVNGRRNGGKLSNDHQQNQSKLQHTGKDTLKAGKNAVERRSNRCNGNSGFEGQSRYVPSSGMSAKELCENDDLATSLVLDPYLGFQTHKMNTSAFPSRSSRHFSKSDSFSHNNPVRFRPIKGRQEELKEVIERFKKDEHLEKAFKCLTSGEWARHYFLNKNKMQEKLFKEHVFIYLRMFATDSGFEILPCNRYSSEQNGAKIVATKEWKRNDKIELLVGCIAELSEIEENMLLRHGENDFSVMYSTRKNCAQLWLGPAAFINHDCRPNCKFVSTGRDTACVKALRDIEPGEEISCYYGDGFFGENNEFCECYTCERRGTGAFKSRVGLPAPAPVINSKYGLRETDKRLNRLKKLGDSSKNSDSQSVSSNTDADTTQEKNNASK

>gi|31543169|ref|NP_116090.2| histone-lysine N-methyltransferase SUV420H2 [Homo sapiens]

MGPDRVTARELCENDDLATSLVLDPYLGFRTHKMNVSPVPPLRRQQHLRSALETFLRQRDLEAAYRALTLGGWTARYFQSRGPRQEAALKTHVYRYLRAFLPESGFTILPCTRYSMETNGAKIVSTRAWKKNEKLELLVGCIAELREADEGLLRAGENDFSIMYSTRKRSAQLWLGPAAFINHDCKPNCKFVPADGNAACVKVLRDIEPGDEVTCFYGEGFFGEKNEHCECHTCERKGEGAFRTRPREPALPPRPLDKYQLRETKRRLQQGLDSGSRQGLLGPRACVHPSPLRRDPFCAACQPLRLPACSARPDTSPLWLQWLPQPQPRVRPRKRRRPRPRRAPVLSTHHAARVSLHRWGGCGPHCRLRGEALVALGQPPHARWAPQQDWHWARRYGLPYVVRVDLRRLAPAPPATPAPAGTPGPILIPKQALAFAPFSPPKRLRLVVSHGSIDLDVGGEEL

>gi|19913348|ref|NP_579877.1| histone-lysine N-methyltransferase NSD2 isoform 1 [Homo sapiens]

MEFSIKQSPLSVQSVVKCIKMKQAPEILGSANGKTPSCEVNRECSVFLSKAQLSSSLQEGVMQKFNGHDALPFIPADKLKDLTSRVFNGEPGAHDAKLRFESQEMKGIGTPPNTTPIKNGSPEIKLKITKTYMNGKPLFESSICGDSAADVSQSEENGQKPENKARRNRKRSIKYDSLLEQGLVEAALVSKISSPSDKKIPAKKESCPNTGRDKDHLLKYNVGDLVWSKVSGYPWWPCMVSADPLLHSYTKLKGQKKSARQYHVQFFGDAPERAWIFEKSLVAFEGEGQFEKLCQESAKQAPTKAEKIKLLKPISGKLRAQWEMGIVQAEEAASMSVEERKAKFTFLYVGDQLHLNPQVAKEAGIAAESLGEMAESSGVSEEAAENPKSVREECIPMKRRRRAKLCSSAETLESHPDIGKSTPQKTAEADPRRGVGSPPGRKKTTVSMPRSRKGDAASQFLVFCQKHRDEVVAEHPDASGEEIEELLRSQWSLLSEKQRARYNTKFALVAPVQAEEDSGNVNGKKRNHTKRIQDPTEDAEAEDTPRKRLRTDKHSLRKRDTITDKTARTSSYKAMEAASSLKSQAATKNLSDACKPLKKRNRASTAASSALGFSKSSSPSASLTENEVSDSPGDEPSESPYESADETQTEVSVSSKKSERGVTAKKEYVCQLCEKPGSLLLCEGPCCGAFHLACLGLSRRPEGRFTCSECASGIHSCFVCKESKTDVKRCVVTQCGKFYHEACVKKYPLTVFESRGFRCPLHSCVSCHASNPSNPRPSKGKMMRCVRCPVAYHSGDACLAAGCSVIASNSIICTAHFTARKGKRHHAHVNVSWCFVCSKGGSLLCCESCPAAFHPDCLNIEMPDGSWFCNDCRAGKKLHFQDIIWVKLGNYRWWPAEVCHPKNVPPNIQKMKHEIGEFPVFFFGSKDYYWTHQARVFPYMEGDRGSRYQGVRGIGRVFKNALQEAEARFREIKLQREARETQESERKPPPYKHIKVNKPYGKVQIYTADISEIPKCNCKPTDENPCGFDSECLNRMLMFECHPQVCPAGEFCQNQCFTKRQYPETKIIKTDGKGWGLVAKRDIRKGEFVNEYVGELIDEEECMARIKHAHENDITHFYMLTIDKDRIIDAGPKGNYSRFMNHSCQPNCETLKWTVNGDTRVGLFAVCDIPAGTELTFNYNLDCLGNEKTVCRCGASNCSGFLGDRPKTSTTLSSEEKGKKTKKKTRRRRAKGEGKRQSEDECFRCGDGGQLVLCDRKFCTKAYHLSCLGLGKRPFGKWECPWHHCDVCGKPSTSFCHLCPNSFCKEHQDGTAFSCTPDGRSYCCEHDLGAASVRSTKTEKPPPEPGKPKGKRRRRRGWRRVTEGK

>gi|13699811|ref|NP_075447.1| histone-lysine N-methyltransferase NSD3 isoform long [Homo sapiens]

MDFSFSFMQGIMGNTIQQPPQLIDSANIRQEDAFDNNSDIAEDGGQTPYEATLQQGFQYPATTEDLPPLTNGYPSSISVYETQTKYQSYNQYPNGSANGFGAVRNFSPTDYYHSEIPNTRPHEILEKPSPPQPPPPPSVPQTVIPKKTGSPEIKLKITKTIQNGRELFESSLCGDLLNEVQASEHTKSKHESRKEKRKKSNKHDSSRSEERKSHKIPKLEPEEQNRPNERVDTVSEKPREEPVLKEEAPVQPILSSVPTTEVSTGVKFQVGDLVWSKVGTYPWWPCMVSSDPQLEVHTKINTRGAREYHVQFFSNQPERAWVHEKRVREYKGHKQYEELLAEATKQASNHSEKQKIRKPRPQRERAQWDIGIAHAEKALKMTREERIEQYTFIYIDKQPEEALSQAKKSVASKTEVKKTRRPRSVLNTQPEQTNAGEVASSLSSTEIRRHSQRRHTSAEEEEPPPVKIAWKTAAARKSLPASITMHKGSLDLQKCNMSPVVKIEQVFALQNATGDGKFIDQFVYSTKGIGNKTEISVRGQDRLIISTPNQRNEKPTQSVSSPEATSGSTGSVEKKQQRRSIRTRSESEKSTEVVPKKKIKKEQVETVPQATVKTGLQKGASEISDSCKPLKKRSRASTDVEMTSSAYRDTSDSDSRGLSDLQVGFGKQVDSPSATADADVSDVQSMDSSLSRRGTGMSKKDTVCQICESSGDSLIPCEGECCKHFHLECLGLASLPDSKFICMECKTGQHPCFSCKVSGKDVKRCSVGACGKFYHEACVRKFPTAIFESKGFRCPQHCCSACSMEKDIHKASKGRMMRCLRCPVAYHSGDACIAAGSMLVSSYILICSNHSKRSSNSSAVNVGFCFVCARGLIVQDHSDPMFSSYAYKSHYLLNESNRAELMKLPMIPSSSASKKKCEKGGRLLCCESCPASFHPECLSIEMPEGCWNCNDCKAGKKLHYKQIVWVKLGNYRWWPAEICNPRSVPLNIQGLKHDLGDFPVFFFGSHDYYWVHQGRVFPYVEGDKSFAEGQTSINKTFKKALEEAAKRFQELKAQRESKEALEIEKNSRKPPPYKHIKANKVIGKVQIQVADLSEIPRCNCKPADENPCGLESECLNRMLQYECHPQVCPAGDRCQNQCFTKRLYPDAEIIKTERRGWGLRTKRSIKKGEFVNEYVGELIDEEECRLRIKRAHENSVTNFYMLTVTKDRIIDAGPKGNYSRFMNHSCNPNCETQKWTVNGDVRVGLFALCDIPAGMELTFNYNLDCLGNGRTECHCGADNCSGFLGVRPKSACASTNEEKAKNAKLKQKRRKIKTEPKQMHEDYCFQCGDGGELVMCDKKDCPKAYHLLCLNLTQPPYGKWECPWHQCDECSSAAVSFCEFCPHSFCKDHEKGALVPSALEGRLCCSEHDPMAPVSPEYWSKIKCKWESQDHGEEVKE

>gi|172072684|ref|NP_001189.2| PR domain zinc finger protein 1 isoform 1 [Homo sapiens]

MLDICLEKRVGTTLAAPKCNSSTVRFQGLAEGTKGTMKMDMEDADMTLWTEAEFEEKCTYIVNDHPWDSGADGGTSVQAEASLPRNLLFKYATNSEEVIGVMSKEYIPKGTRFGPLIGEIYTNDTVPKNANRKYFWRIYSRGELHHFIDGFNEEKSNWMRYVNPAHSPREQNLAACQNGMNIYFYTIKPIPANQELLVWYCRDFAERLHYPYPGELTMMNLTQTQSSLKQPSTEKNELCPKNVPKREYSVKEILKLDSNPSKGKDLYRSNISPLTSEKDLDDFRRRGSPEMPFYPRVVYPIRAPLPEDFLKASLAYGIERPTYITRSPIPSSTTPSPSARSSPDQSLKSSSPHSSPGNTVSPVGPGSQEHRDSYAYLNASYGTEGLGSYPGYAPLPHLPPAFIPSYNAHYPKFLLPPYGMNCNGLSAVSSMNGINNFGLFPRLCPVYSNLLGGGSLPHPMLNPTSLPSSLPSDGARRLLQPEHPREVLVPAPHSAFSFTGAAASMKDKACSPTSGSPTAGTAATAEHVVQPKATSAAMAAPSSDEAMNLIKNKRNMTGYKTLPYPLKKQNGKIKYECNVCAKTFGQLSNLKVHLRVHSGERPFKCQTCNKGFTQLAHLQKHYLVHTGEKPHECQVCHKRFSSTSNLKTHLRLHSGEKPYQCKVCPAKFTQFVHLKLHKRLHTRERPHKCSQCHKNYIHLCSLKVHLKGNCAAAPAPGLPLEDLTRINEEIEKFDISDNADRLEDVEDDISVISVVEKEILAVVRKEKEETGLKVSLQRNMGNGLLSSGCSLYESSDLPLMKLPPSNPLPLVPVKVKQETVEPMDP

>gi|20336260|ref|NP_056950.2| PR domain zinc finger protein 2 isoform b [Homo sapiens]

MNQNTTEPVAATETLAEVPEHVLRGLPEEVRLFPSAVDKTRIGVWATKPILKGKKFGPFVGDKKKRSQVKNNVYMWEVYYPNLGWMCIDATDPEKGNWLRYVNWACSGEEQNLFPLEINRAIYYKTLKPIAPGEELLVWYNGEDNPEIAAAIEEERASARSKRSSPKSRKGKKKSQENKNKGNKIQDIQLKTSEPDFTSANMRDSAEGPKEDEEKPSASALEQPATLQEVASQEVPPELATPAPAWEPQPEPDERLEAAACEVNDLGEEEEEEEEEDEEEEEDDDDDELEDEGEEEASMPNENSVKEPEIRCDEKPEDLLEEPKTTSEETLEDCSEVTPAMQIPRTKEEANGDVFETFMFPCQHCERKFTTKQGLERHMHIHISTVNHAFKCKYCGKAFGTQINRRRHERRHEAGLKRKPSQTLQPSEDLADGKASGENVASKDDSSPPSLGPDCLIMNSEKASQDTINSSVVEENGEVKELHPCKYCKKVFGTHTNMRRHQRRVHERHLIPKGVRRKGGLEEPQPPAEQAQATQNVYVPSTEPEEEGEADDVYIMDISSNISENLNYYIDGKIQTNNNTSNCDVIEMESASADLYGINCLLTPVTVEITQNIKTTQVPVTEDLPKEPLGSTNSEAKKRRTASPPALPKIKAETDSDPMVPSCSLSLPLSISTTEAVSFHKEKSVYLSSKLKQLLQTQDKLTPAGISATEIAKLGPVCVSAPASMLPVTSSRFKRRTSSPPSSPQHSPALRDFGKPSDGKAAWTDAGLTSKKSKLESHSDSPAWSLSGRDERETVSPPCFDEYKMSKEWTASSAFSSVCNQQPLDLSSGVKQKAEGTGKTPVQWESVLDLSVHKKHCSDSEGKEFKESHSVQPTCSAVKKRKPTTCMLQKVLLNEYNGIDLPVENPADGTRSPSPCKSLEAQPDPDLGPGSGFPAPTVESTPDVCPSSPALQTPSLSSGQLPPLLIPTDPSSPPPCPPVLTVATPPPPLLPTVPLPAPSSSASPHPCPSPLSNATAQSPLPILSPTVSPSPSPIPPVEPLMSAASPGPPTLSSSSSSSSSSSSFSSSSSSSSPSPPPLSAISSVVSSGDNLEASLPMISFKQEELENEGLKPREEPQSAAEQDVVVQETFNKNFVCNVCESPFLSIKDLTKHLSIHAEEWPFKCEFCVQLFKDKTDLSEHRFLLHGVGNIFVCSVCKKEFAFLCNLQQHQRDLHPDKVCTHHEFESGTLRPQNFTDPSKAHVEHMQSLPEDPLETSKEEEELNDSSEELYTTIKIMASGIKTKDPDVRLGLNQHYPSFKPPPFQYHHRNPMGIGVTATNFTTHNIPQTFTTAIRCTKCGKGVDNMPELHKHILACASASDKKRYTPKKNPVPLKQTVQPKNGVVVLDNSGKNAFRRMGQPKRLNFSVELSKMSSNKLKLNALKKKNQLVQKAILQKNKSAKQKADLKNACESSSHICPYCNREFTYIGSLNKHAAFSCPKKPLSPPKKKVSHSSKKGGHSSPASSDKNSNSNHRRRTADAEIKMQSMQTPLGKTRARSSGPTQVPLPSSSFRSKQNVKFAASVKSKKPSSSSLRNSSPIRMAKITHVEGKKPKAVAKNHSAQLSSKTSRSLHVRVQKSKAVLQSKSTLASKKRTDRFNIKSRERSGGPVTRSLQLAAAADLSENKREDGSAKQELKDFRNFL

>gi|157364943|ref|NP_005232.2| MDS1 and EVI1 complex locus protein EVI1 isoform b prdm3 [Homo sapiens]

MKSEDYPHETMAPDIHEERQYRCEDCDQLFESKAELADHQKFPCSTPHSAFSMVEEDFQQKLESENDLQEIHTIQECKECDQVFPDLQSLEKHMLSHTEEREYKCDQCPKAFNWKSNLIRHQMSHDSGKHYECENCAKVFTDPSNLQRHIRSQHVGARAHACPECGKTFATSSGLKQHKHIHSSVKPFICEVCHKSYTQFSNLCRHKRMHADCRTQIKCKDCGQMFSTTSSLNKHRRFCEGKNHFAAGGFFGQGISLPGTPAMDKTSMVNMSHANPGLADYFGANRHPAGLTFPTAPGFSFSFPGLFPSGLYHRPPLIPASSPVKGLSSTEQTNKSQSPLMTHPQILPATQDILKALSKHPSVGDNKPVELQPERSSEERPFEKISDQSESSDLDDVSTPSGSDLETTSGSDLESDIESDKEKFKENGKMFKDKVSPLQNLASINNKKEYSNHSIFSPSLEEQTAVSGAVNDSIKAIASIAEKYFGSTGLVGLQDKKVGALPYPSMFPLPFFPAFSQSMYPFPDRDLRSLPLKMEPQSPGEVKKLQKGSSESPFDLTTKRKDEKPLTPVPSKPPVTPATSQDQPLDLSMGSRSRASGTKLTEPRKNHVFGGKKGSNVESRPASDGSLQHARPTPFFMDPIYRVEKRKLTDPLEALKEKYLRPSPGFLFHPQFQLPDQRTWMSAIENMAEKLESFSALKPEASELLQSVPSMFNFRAPPNALPENLLRKGKERYTCRYCGKIFPRSANLTRHLRTHTGEQPYRCKYCDRSFSISSNLQRHVRNIHNKEKPFKCHLCDRCFGQQTNLDRHLKKHENGNMSGTATSSPHSELESTGAILDDKEDAYFTEIRNFIGNSNHGSQSPRNVEERMNGSHFKDEKALVTSQNSDLLDDEEVEDEVLLDEEDEDNDITGKTGKEPVTSNLHEGNPEDDYEETSALEMSCKTSPVRYKEEEYKSGLSALDHIRHFTDSLKMRKMEDNQYSEAELSSFSTSHVPEELKQPLHRKSKSQAYAMMLSLSDKESLHSTSHSSSNVWHSMARAAAESSAIQSISHV

>gi|41349474|ref|NP_036538.3| PR domain zinc finger protein 4 [Homo sapiens]

MHHRMNEMNLSPVGMEQLTSSSVSNALPVSGSHLGLAASPTHSAIPAPGLPVAIPNLGPSLSSLPSALSLMLPMGIGDRGVMCGLPERNYTLPPPPYPHLESSYFRTILPGILSYLADRPPPQYIHPNSINVDGNTALSITNNPSALDPYQSNGNVGLEPGIVSIDSRSVNTHGAQSLHPSDGHEVALDTAITMENVSRVTSPISTDGMAEELTMDGVAGEHSQIPNGSRSHEPLSVDSVSNNLAADAVGHGGVIPMHGNGLELPVVMETDHIASRVNGMSDSALSDSIHTVAMSTNSVSVALSTSHNLASLESVSLHEVGLSLEPVAVSSITQEVAMGTGHVDVSSDSLSFVSPSLQMEDSNSNKENMATLFTIWCTLCDRAYPSDCPEHGPVTFVPDTPIESRARLSLPKQLVLRQSIVGAEVGVWTGETIPVRTCFGPLIGQQSHSMEVAEWTDKAVNHIWKIYHNGVLEFCIITTDENECNWMMFVRKARNREEQNLVAYPHDGKIFFCTSQDIPPENELLFYYSRDYAQQIGVPEHPDVHLCNCGKECNSYTEFKAHLTSHIHNHLPTQGHSGSHGPSHSKERKWKCSMCPQAFISPSKLHVHFMGHMGMKPHKCDFCSKAFSDPSNLRTHLKIHTGQKNYRCTLCDKSFTQKAHLESHMVIHTGEKNLKCDYCDKLFMRRQDLKQHVLIHTQERQIKCPKCDKLFLRTNHLKKHLNSHEGKRDYVCEKCTKAYLTKYHLTRHLKTCKGPTSSSSAPEEEEEDDSEEEDLADSVGTEDCRINSAVYSADESLSAHK

>gi|41349476|ref|NP_061169.2| PR domain zinc finger protein 5 [Homo sapiens]

MLGMYVPDRFSLKSSRVQDGMGLYTARRVRKGEKFGPFAGEKRMPEDLDENMDYRLMWEVRGSKGEVLYILDATNPRHSNWLRFVHEAPSQEQKNLAAIQEGENIFYLAVEDIETDTELLIGYLDSDMEAEEEEQQIMTVIKEGEVENSRRQSTAGRKDRLGCKEDYACPQCESSFTSEDILAEHLQTLHQKPTEEKEFKCKNCGKKFPVKQALQRHVLQCTAKSSLKESSRSFQCSVCNSSFSSASSFEQHQETCRGDARFVCKADSCGKRLKSKDALKRHQENVHTGDPKKKLICSVCNKKCSSASSLQEHRKIHEIFDCQECMKKFISANQLKRHMITHSEKRPYNCEICNKSFKRLDQVGAHKVIHSEDKPYKCKLCGKGFAHRNVYKNHKKTHSEERPFQCEECKALFRTPFSLQRHLLIHNSERTFKCHHCDATFKRKDTLNVHVQVVHERHKKYRCELCNKAFVTPSVLRSHKKTHTGEKEKICPYCGQKFASSGTLRVHIRSHTGERPYQCPYCEKGFSKNDGLKMHIRTHTREKPYKCSECSKAFSQKRGLDEHKRTHTGEKPFQCDVCDLAFSLKKMLIRHKMTHNPNRPLAECQFCHKKFTRNDYLKVHMDNIHGVADS

>gi|210031233|ref|NP_001129711.1| putative histone-lysine N-methyltransferase PRDM6 [Homo sapiens]

MLKPGDPGGSAFLKVDPAYLQHWQQLFPHGGAGPLKGSGAAGLLSAPQPLQPPPPPPPPERAEPPPDSLRPRPASLSSASSTPASSSTSASSASSCAAAAAAAALAGLSALPVSQLPVFAPLAAAAVAAEPLPPKELCLGATSGPGPVKCGGGGGGGGEGRGAPRFRCSAEELDYYLYGQQRMEIIPLNQHTSDPNNRCDMCADNRNGECPMHGPLHSLRRLVGTSSAAAAAPPPELPEWLRDLPREVCLCTSTVPGLAYGICAAQRIQQGTWIGPFQGVLLPPEKVQAGAVRNTQHLWEIYDQDGTLQHFIDGGEPSKSSWMRYIRCARHCGEQNLTVVQYRSNIFYRACIDIPRGTELLVWYNDSYTSFFGIPLQCIAQDENLNVPSTVMEAMCRQDALQPFNKSSKLAPTTQQRSVVFPQTPCSRNFSLLDKSGPIESGFNQINVKNQRVLASPTSTSQLHSEFSDWHLWKCGQCFKTFTQRILLQMHVCTQNPDRPYQCGHCSQSFSQPSELRNHVVTHSSDRPFKCGYCGRAFAGATTLNNHIRTHTGEKPFKCERCERSFTQATQLSRHQRMPNECKPITESPESIEVD

>gi|41349478|ref|NP_443722.2| probable histone-lysine N-methyltransferase PRDM7 isoform 2 [Homo sapiens]

MCQNFFIDSCAAHGPPTFVKDSAVDKGHPNRSALSLPPGLRIGPSGIPQAGLGVWNEASDLPLGLHFGPYEGRITEDEEAANSGYSWLITKGRNCYEYVDGKDKSSANWMRTKARDPSMSLMLSGLFKSKISQSTCGTQSLLSELPRTICKKTSPTRESLPRGSESGAAIF

>gi|150378439|ref|NP_064611.3| PR domain zinc finger protein 8 [Homo sapiens]

MEDTGIQRGIWDGDAKAVQQCLTDIFTSVYTTCDIPENAIFGPCVLSHTSLYDSIAFIALKSTDKRTVPYIFRVDTSAANGSSEGLMWLRLVQSARDKEEQNLEAYIKNGQLFYRSLRRIAKDEELLVWYGKELTELLLLCPSRSHNKMNGSSPYTCLECSQRFQFEFPYVAHLRFRCPKRLHSADISPQDEQGGGVGTKDHGGGGGGGKDQQQQQQEAPLGPGPKFCKAGPLHHYPSPSPESSNPSAAAGGSSAKPSTDFHNLARELENSRGGSSCSPAQSLSSGSGSGGGGGHQEAELSPDGIATGGGKGKRKFPEEAAEGGGGAGLVGGRGRFVERPLPASKEDLVCTPQQYRASGSYFGLEENGRLFAPPSPETGEAKRSAFVEVKKAARAASLQEEGTADGAGVASEDQDAGGGGGSSTPAAASPVGAEKLLAPRPGGPLPSRLEGGSPARGSAFTSVPQLGSAGSTSGGGGTGAGAAGGAGGGQGAASDERKSAFSQPARSFSQLSPLVLGQKLGALEPCHPADGVGPTRLYPAAADPLAVKLQGAADLNGGCGSLPSGGGGLPKQSPFLYATAFWPKSSAAAAAAAAAAAAGPLQLQLPSALTLLPPSFTSLCLPAQNWCAKCNASFRMTSDLVYHMRSHHKKEYAMEPLVKRRREEKLKCPICNESFRERHHLSRHMTSHN

>gi|147905620|ref|NP_064612.2| histone-lysine N-methyltransferase PRDM9 [Homo sapiens]

MSPEKSQEESPEEDTERTERKPMVKDAFKDISIYFTKEEWAEMGDWEKTRYRNVKRNYNALITIGLRATRPAFMCHRRQAIKLQVDDTEDSDEEWTPRQQVKPPWMALRVEQRKHQKGMPKASFSNESSLKELSRTANLLNASGSEQAQKPVSPSGEASTSGQHSRLKLELRKKETERKMYSLRERKGHAYKEVSEPQDDDYLYCEMCQNFFIDSCAAHGPPTFVKDSAVDKGHPNRSALSLPPGLRIGPSGIPQAGLGVWNEASDLPLGLHFGPYEGRITEDEEAANNGYSWLITKGRNCYEYVDGKDKSWANWMRYVNCARDDEEQNLVAFQYHRQIFYRTCRVIRPGCELLVWYGDEYGQELGIKWGSKWKKELMAGREPKPEIHPCPSCCLAFSSQKFLSQHVERNHSSQNFPGPSARKLLQPENPCPGDQNQEQQYPDPHSRNDKTKGQEIKERSKLLNKRTWQREISRAFSSPPKGQMGSCRVGKRIMEEESRTGQKVNPGNTGKLFVGVGISRIAKVKYGECGQGFSVKSDVITHQRTHTGEKLYVCRECGRGFSWKSHLLIHQRIHTGEKPYVCRECGRGFSWQSVLLTHQRTHTGEKPYVCRECGRGFSRQSVLLTHQRRHTGEKPYVCRECGRGFSRQSVLLTHQRRHTGEKPYVCRECGRGFSWQSVLLTHQRTHTGEKPYVCRECGRGFSWQSVLLTHQRTHTGEKPYVCRECGRGFSNKSHLLRHQRTHTGEKPYVCRECGRGFRDKSHLLRHQRTHTGEKPYVCRECGRGFRDKSNLLSHQRTHTGEKPYVCRECGRGFSNKSHLLRHQRTHTGEKPYVCRECGRGFRNKSHLLRHQRTHTGEKPYVCRECGRGFSDRSSLCYHQRTHTGEKPYVCREDE

>gi|41349462|ref|NP_955470.1| PR domain zinc finger protein 10 isoform 3 [Homo sapiens]

MSAYSVPSTFAQASLPVHNQVLPSIESVDGSDPLATLQTPLGRLEAKEEEDEDEDEDTEEDEEEDGEDTDLDDWEPDPPRPFDPHDLWCEECNNAHASVCPKHGPLHPIPNRPVLTRARASLPLVLYIDRFLGGVFSKRRIPKRTQFGPVEGPLVRGSELKDCYIHLKVSLDKGDRKERDLHEDLWFELSDETLCNWMMFVRPAQNHLEQNLVAYQYGHHVYYTTIKNVEPKQELKVWYAASYAEFVNQKIHDISEEERKVLREQEKNWPCYECNRRFISSEQLQQHLNSHDEKLDVFSRTRGRGRGRGKRRFGPGRRPGRPPKFIRLEITSENGEKSDDGTQDLLHFPTKEQFDEAEPATLNGLDQPEQTTIPIPQLPQETQSSLEHEPETHTLHLQPQHEESVVPTQSTLTADDMRRAKRIRLELQNAALQHLFIRKSFRPFKCLQCGKAFREKDKLDQHLRFHGREGNCPLTCDLCNKGFISSTSLESHMKLHSDQKTYSCIFCPESFDRLDLLKDHVAIHINDGYFTCPTCKKRFPDFIQVKKHVRSFHSEKIYQCTECDKAFCRPDKLRLHMLRHSDRKDFLCSTCGKQFKRKDKLREHMQRMHNPEREAKKADRISRSKTFKPRITSTDYDSFTFKCRLCMMGFRRRGMLVNHLSKRHPDMKIEEVPELTLPIIKPNRDYFCQYCDKVYKSASKRKAHILKNHPGAELPPSIRKLRPAGPGEPDPMLSTHTQLTGTIATPPVCCPHCSKQYSSKTKMVQHIRKKHPEFAQLSNTIHTPLTTAVISATPAVLTTDSATGETVVTTDLLTQAMTELSQTLTTDYRTPQGDYQRIQYIPVSQSASGLQQPQHIQLQVVQVASATSPHQSQQSTVDVGQLHDPQPYPQHAIQVQHIQVSGQPLSPSAQQAQQGLSPSHIQGSSSTQGQALQQQQQQQQNSSVQHTYLPSAWNSFRGYSSEIQMMTLPPGQFVITDSGVATPVTTGQVKAVTSGHYVLSESQSELEEKQTSALSGGVQVEPPAHSDSLDPQTNSQQQTTQYIITTTTNGNGSSEVHITKP

>gi|41349466|ref|NP_064614.2| PR domain-containing protein 11 [Homo sapiens]

MLKMAEPIASLMIVECRACLRCSPLFLYQREKDRMTENMKECLAQTNAAVGDMVTVVKTEVCSPLRDQEYGQPCSRRPDSSAMEVEPKKLKGKRDLIVPKSFQQVDFWFCESCQEYFVDECPNHGPPVFVSDTPVPVGIPDRAALTIPQGMEVVKDTSGESDVRCVNEVIPKGHIFGPYEGQISTQDKSAGFFSWLIVDKNNRYKSIDGSDETKANWMRYVVISREEREQNLLAFQHSERIYFRACRDIRPGEWLRVWYSEDYMKRLHSMSQETIHRNLARGEKRLQREKSEQVLDNPEDLRGPIHLSVLRQGKSPYKRGFDEGDVHPQAKKKKIDLIFKDVLEASLESAKVEAHQLALSTSLVIRKVPKYQDDAYSQCATTMTHGVQNIGQTQGEGDWKVPQGVSKEPGQLEDEEEEPSSFKADSPAEASLASDPHELPTTSFCPNCIRLKKKVRELQAELDMLKSGKLPEPPVLPPQVLELPEFSDPAGKLVWMRLLSEGRVRSGLCGG

>gi|15042949|ref|NP_067632.2| PR domain zinc finger protein 12 [Homo sapiens]

MMGSVLPAEALVLKTGLKAPGLALAEVITSDILHSFLYGRWRNVLGEQLFEDKSHHASPKTAFTAEVLAQSFSGEVQKLSSLVLPAEVIIAQSSIPGEGLGIFSKTWIKAGTEMGPFTGRVIAPEHVDICKNNNLMWEVFNEDGTVRYFIDASQEDHRSWMTYIKCARNEQEQNLEVVQIGTSIFYKAIEMIPPDQELLVWYGNSHNTFLGIPGVPGLEEDQKKNKHEDFHPADSAAGPAGRMRCVICHRGFNSRSNLRSHMRIHTLDKPFVCRFCNRRFSQSSTLRNHVRLHTGERPYKCQVCQSAYSQLAGLRAHQKSARHRPPSTALQAHSPALPAPHAHAPALAAAAAAAAAAAAHHLPAMVL

>gi|124107614|ref|NP_067633.2| PR domain zinc finger protein 13 [Homo sapiens]

MHGAARAPATSVSADCCIPAGLRLGPVPGTFKLGKYLSDRREPGPKKKVRMVRGELVDESGGSPLEWIGLIRAARNSQEQTLEAIADLPGGQIFYRALRDVQPGEELTVWYSNSLAQWFDIPTTATPTHDEKGEERYICWYCWRTFRYPNSLKAHLRFHCVFSGGGGGAFLHHEHAARQGAVPAADGLGLSPKPPAPDFAAPSQAGTLRPHPLGPPPVQACGAREGIKREASSAPSATSPTPGKWGQPKKGKEQLDRALDMSGAARGQGHFLGIVGGSSAGVGSLAFYPGVRSAFKPAGLARAAAAAHGDPYREESSSKQGAGLALGRLLGGGRACGRPGSGENSAAGGAGHHHHHHAHHHHHPKCLLAGDPPPPPPPGLPCSGALRGFPLLSVPPEEASAFKHVERAPPAAAALPGARYAQLPPAPGLPLERCALPPLDPGGLKAYPGGECSHLPAVMPAFTVYNGELLYGSPATTAYYPLKLHFGGLLKYPESISYFSGPAAAALSPAELGSLASIDREIAMHNQQLSEMAAGKGRGRLDSGTLPPAVAAAGGTGGGGSGGSGAGKPKTGHLCLYCGKLYSRKYGLKIHMRTHTGYKPLKCKVCLRPFGDPSNLNKHIRLHAEGNTPYRCEFCGKVLVRRRDLERHVKSRHPGQSLLAKAGDGPGAEPGYPPEPGDPKSDDSDVDVCFTDDQSDPEVGGGGERDL

>gi|13375636|ref|NP_078780.1| PR domain zinc finger protein 14 [Homo sapiens]

MALPRPSEAVPQDKVCYPPESSPQNLAAYYTPFPSYGHYRNSLATVEEDFQPFRQLEAAASAAPAMPPFPFRMAPPLLSPGLGLQREPLYDLPWYSKLPPWYPIPHVPREVPPFLSSSHEYAGASSEDLGHQIIGGDNESGPCCGPDTLIPPPPADASLLPEGLRTSQLLPCSPSKQSEDGPKPSNQEGKSPARFQFTEEDLHFVLYGVTPSLEHPASLHHAISGLLVPPDSSGSDSLPQTLDKDSLQLPEGLCLMQTVFGEVPHFGVFCSSFIAKGVRFGPFQGKVVNASEVKTYGDNSVMWEIFEDGHLSHFIDGKGGTGNWMSYVNCARFPKEQNLVAVQCQGHIFYESCKEIHQNQELLVWYGDCYEKFLDIPVSLQVTEPGKQPSGPSEESAEGYRCERCGKVFTYKYYRDKHLKYTPCVDKGDRKFPCSLCKRSFEKRDRLRIHILHVHEKHRPHKCSTCGKCFSQSSSLNKHMRVHSGDRPYQCVYCTKRFTASSILRTHIRQHSGEKPFKCKYCGKSFASHAAHDSHVRRSHKEDDGCSCSICGKIFSDQETFYSHMKFHEDY

>gi|93204879|ref|NP_071398.3| PR domain zinc finger protein 15 isoform 1 [Homo sapiens]

MPRRRPPASGAAQFPERIATRSPDPIPLCTFQRQPRAAPVQPPCRLFFVTFAGCGHRWRSESKPGWISRSRSGIALRAARPPGSSPPRPAAPRPPPPGGVVAEAPGDVVIPRPRVQPMRVARGGPWTPNPAFREAESWSQIGNQRVSEQLLETSLGNEVSDTEPLSPASAGLRRNPALPPGPFAQNFSWGNQENLPPALGKIANGGGTGAGKAECGYETESHLLEPHEIPLNVNTHKFSDCEFPYEFCTVCFSPFKLLGMSGVEGVWNQHSRSASMHTFLNHSATGIREAGCRKDMPVSEMAEDGSEEIMFIWCEDCSQYHDSECPELGPVVMVKDSFVLSRARSWPASGHVHTQAGQGMRGYEDRDRADPQQLPEAVPAGLVRRLSGQQLPCRSTLTWGRLCHLVAQGRSSLPPNLEIRRLEDGAEGVFAITQLVKRTQFGPFESRRVAKWEKESAFPLKVFQKDGHPVCFDTSNEDDCNWMMLVRPAAEAEHQNLTAYQHGSDVYFTTSRDIPPGTELRVWYAAFYAKKMDKPMLKQAGSGVHAAGTPENSAPVESEPSQWACKVCSATFLELQLLNEHLLGHLEQAKSLPPGSQSEAAAPEKEQDTPRGEPPAVPESENVATKEQKKKPRRGRKPKVSKAEQPLVIVEDKEPTEQVAEIITEVPPDEPVSATPDERIMELVLGKLATTTTDTSSVPKFTHHQNNTITLKRSLILSSRHGIRRKLIKQLGEHKRVYQCNICSKIFQNSSNLSRHVRSHGDKLFKCEECAKLFSRKESLKQHVSYKHSRNEVDGEYRYRCGTCEKTFRIESALEFHNCRTDDKTFQCEMCFRFFSTNSNLSKHKKKHGDKKFACEVCSKMFYRKDVMLDHQRRHLEGVRRVKREDLEAGGENLVRYKKEPSGCPVCGKVFSCRSNMNKHLLTHGDKKYTCEICGRKFFRVDVLRDHIHVHFKDIALMDDHQREEFIGKIGISSEENDDNSDESADSEPHKYSCKRCQLTFGRGKEYLKHIMEVHKEKGYGCSICNRRFALKATYHAHMVIHRENLPDPNVQKYIHPCEICGRIFNSIGNLERHKLIHTGVKSHACEQCGKSFARKDMLKEHMRVHDNVREYLCAECGKGMKTKHALRHHMKLHKGIKEYECKECHRRFAQKVNMLKHCKRHTGIKDFMCELCGKTFSERNTMETHKLIHTVGKQWTCSVCDKKYVTEYMLQKHVQLTHDKVEAQSCQLCGTKVSTRASMSRHMRRKHPEVLAVRIDDLDHLPETTTIDASSIGIVQPELTLEQEDLAEGKHGKAAKRSHKRKQKPEEEAGAPVPEDATFSEYSEKETEFTGSVGDETNSAVQSIQQVVVTLGDPNVTTPSSSVGLTNITVTPITTAAATQFTNLQPVAVGHLTTPERQLQLDNSILTVTFDTVSGSAMLHNRQNDVQIHPQPEASNPQSVAHFINLTTLVNSITPLGSQLSDQHPLTWRAVPQTDVLPPSQPQAPPQQAAQPQVQAEQQQQQMYSY

>gi|289547573|ref|NP_071397.3| PR domain zinc finger protein 16 isoform 1 [Homo sapiens]

MRSKARARKLAKSDGDVVNNMYEPNRDLLASHSAEDEAEDSAMSPIPVGPPSPFPTSEDFTPKEGSPYEAPVYIPEDIPIPADFELRESSIPGAGLGVWAKRKMEAGERLGPCVVVPRAAAKETDFGWEQILTDVEVSPQEGCITKISEDLGSEKFCVDANQAGAGSWLKYIRVACSCDDQNLTMCQISEQIYYKVIKDIEPGEELLVHVKEGVYPLGTVPPGLDEEPTFRCDECDELFQSKLDLRRHKKYTCGSVGAALYEGLAEELKPEGLGGGSGQAHECKDCERMFPNKYSLEQHMVIHTEEREYKCDQCPKAFNWKSNLIRHQMSHDSGKRFECENCVKVFTDPSNLQRHIRSQHVGARAHACPDCGKTFATSSGLKQHKHIHSTVKPFICEVCHKSYTQFSNLCRHKRMHADCRTQIKCKDCGQMFSTTSSLNKHRRFCEGKNHYTPGGIFAPGLPLTPSPMMDKAKPSPSLNHASLGFNEYFPSRPHPGSLPFSTAPPTFPALTPGFPGIFPPSLYPRPPLLPPTSLLKSPLNHTQDAKLPSPLGNPALPLVSAVSNSSQGTTAAAGPEEKFESRLEDSCVEKLKTRSSDMSDGSDFEDVNTTTGTDLDTTTGTGSDLDSDVDSDPDKDKGKGKSAEGQPKFGGGLAPPGAPNSVAEVPVFYSQHSFFPPPDEQLLTATGAAGDSIKAIASIAEKYFGPGFMGMQEKKLGSLPYHSAFPFQFLPNFPHSLYPFTDRALAHNLLVKAEPKSPRDALKVGGPSAECPFDLTTKPKDVKPILPMPKGPSAPASGEEQPLDLSIGSRARASQNGGGREPRKNHVYGERKLGAGEGLPQVCPARMPQQPPLHYAKPSPFFMDPIYSRVEKRKVTDPVGALKEKYLRPSPLLFHPQMSAIETMTEKLESFAAMKADSGSSLQPLPHHPFNFRSPPPTLSDPILRKGKERYTCRYCGKIFPRSANLTRHLRTHTGEQPYRCKYCDRSFSISSNLQRHVRNIHNKEKPFKCHLCNRCFGQQTNLDRHLKKHEHENAPVSQHPGVLTNHLGTSASSPTSESDNHALLDEKEDSYFSEIRNFIANSEMNQASTRTEKRADMQIVDGSAQCPGLASEKQEDVEEEDDDDLEEDDEDSLAGKSQDDTVSPAPEPQAAYEDEEDEEPAASLAVGFDHTRRCAEDHEGGLLALEPMPTFGKGLDLRRAAEEAFEVKDVLNSTLDSEALKHTLCRQAKNQAYAMMLSLSEDTPLHTPSQGSLDAWLKVTGATSESGAFHPINHL

***Lottia gigantea*  (Metazoa)**

>jgi|Lotgi1|106321|e_gw1.5.740.1

RGQTSLQSAVKLGCYEICKLLIDAGADVNMADAESNSLLIVACQNNHNDIAELLIQYNADRDFQNSDGNTALNICGCDGNVTITQSLLNMGCDVNICNYKIQSPVYTACYYKHIEIVKLLVNVKDCDINWGDVTHCTPLMLACEVGCLKIVQLLLQAGKDLIFIQIKQISPVRSKAHHIQNSVYGQQILIRFQRIKNGCK*

>jgi|Lotgi1|111681|e_gw1.14.355.1

YRFLIFFDDGYAQYTKASEIHKVVSQSDNVWEDVYPESQDFIRDYLSQYPERPMVRLQKGQGVQTEWNGRWWTAKVMEVDASLVKMYFQADKRTEWIYRGSTRLEPLFKALKTVPSHNSKFKKLAHNKKPILSFYLKMYISDLSHSKYSLANLEAHKGSSKGRRNDPNSKKPRIVYTRGEEEKGNILTIYFTQFLINIVFILVPHRCSSKCLKDVEEDPTKHRGRNPLLIPLLCSWERHVCKIKQSGKRHVIYRTPCARRLRTIEEVDRYLRLTNSNLTIDLFCLDPYLHTHTEFVPIKTFCDIKDLSYGKENVPISCVNGIDRQYPDYVEYSTVRMPAKGVKLNLDPDFLPGCDCTDGCRDSSKCACRQLSVEHTTALGEKDPTIGYKHRRLNEPIFTGIVECNVNCKCDKRCHNKVVQNGLNLRLQVFKTEKRGWGLRCLDDIPKGGFICIYAGQLLTDTGANEVNYIIEYFGIFQDGKQYGDEYLAELDFIEVVERQKEGYESDVEEPDSHIEEDIADEEDEEEEEEEEFEGTQHNSGSDSEFYFANPVAIYVVNFLADKRPNHPGTRSFFEDNQMCYIMDAKSMGNIGRYLNHSCEPNVFVQNIFVDTHDLRYPWVTFFAGMYIRAGTELTWDYNYEVGSVPDKVLYCYCGSSQCRGRLL*

>jgi|Lotgi1|116536|e_gw1.24.108.1

MISFVFQYIKTNIPGPNLDLELFEEEYAGCDCQKCDQIDFHLRLCSCITTTGLNYRDNKLLSEIFENVESKPILECNSNCSCSKTCHNRLVQKGIQIKLEVFDAGIKGLGLRTLEIIPRGTFVCEYAGEILTKSEAAKRCQNLLPSDMNYLMVLQEHCSSGLLQTFVDPKFKGNIGRWLNHSCQPNLTMFPVRINISVPRLALFANRDIKPLEELTFNYGVSAQLGARVSANRKKCFCGSKDCQELLPFDAGLCS*

>jgi|Lotgi1|116803|e_gw1.25.25.1

MSYSSRYTPSTGMTCKELSENDDLATSLVLDPYLGFQTHKMNVRHRPLKSKRELKGVVEEFIKTQSYEKCFKSLMSVECCRSSYIIKSKSQQQTFKEHVFRYFRMFDKRAGFRLMPCHRYSSEGQVGGKICTTKQWKRNDKIPMLVGCIAELTPAEEATYLRPGKNDFSVMFSCRKNCAQLWLGPAAFINHDCRPNCKFVSTGRDTACVKALRDIEEGEEITCLYGEDFFGDKNSYCECETCERRQMGAYEPKNKQQLIIDPEEDKGYKLRDTDDRLNRIKTQPELKQPNINGFITSANGRLLADQDYSFRNM*

>jgi|Lotgi1|117025|e_gw1.25.144.1

MVILLLYWYLFYRKVESDITTTNKTITDYFPVRRSGRRCKSELEKERMIEYEDKVLTYCEDGLEVVKMEDKGRGVVATKMFKKGDFVVEYAGDLIDLAESKDREMFYGMSTKFGCYMYYFNLKGKHYCIDATAESGKLGRLLNHSRISFNCCTKLIEIKNTPYLIIIASRDIVKGEELLYDYGDRSKESLEAHPWLKL*

>jgi|Lotgi1|118531|e_gw1.29.51.1

MLYCDGSIHRYIVLALHENQCQYFGCIFQDVCKSCGAFGRDEESKLISCTQCGQCYHPYCANVKVTKVILLKGWRCLDCTVCEGCGKPDDEGRLLLCDDCDISYHTYCLKPPLEHVPKGNWKCKWCVSCLNCGATTPGFGCNWKNNYTQCGPCASKVICPACRRSYQQEELILQCVQCDR*

>jgi|Lotgi1|118649|e_gw1.29.113.1

CGKPPVTGSKELDDYPEIHSCVEKIRKLQSGLSSGKKDRSDSFKDFFTWLTDNNVDISTVEITATGESGYGLKAKEHIKEASLFLTIPRKLMLTVDSAKKSVLGELVKEDKILSAMPNITLALHLLCEKYTPDSFWAPYINILPESYNTPLYFTSDDLHQLKGSPVLGEAVSQYRNIARQYAYFYRLLQKQEPYISKLPICNNFTFDEYRWAVSTVMTRQNQIPTEEGSKVTFGLIPLWDMCNHTNGHYTSDFNVEKNVSECYALRDFEAGEQVYIFYGARSNAEFMIHNGFVYPENEHDRVCIKLGISKTDTLFQTKSDILGQIGILPSRSFFLHKGQFPVDGELLAFLRISTADDALLREQFGGNVTMEMLENLKDEEKPICAENENKVWSFLQTRASLLLKTYPNTVQEDQEELNSGKDLTVPHQMALQLRIGEKQILTSTMEYCSQKCSEGEK*

>jgi|Lotgi1|123404|e_gw1.44.154.1

RVIDAGPKGNLSRFMNHSCQPNCETQKWMVNGDVRVGLFALNDISAGSELTFNYNLECLGNEKTVCACGAFNCSGFLGVRPKVSSIANANRVKDGNKKKKRKKKIDIKKEHDDDCFRCEEGGELVMCDHNTCPKVYHLQCLNLTKPPTGIDSLL*

>jgi|Lotgi1|134869|e_gw1.93.38.1

MNLNPKSKRYKIKKNAIAKELEEWEQKLNNISIDPAKIFVENQVDLEGPPQDFEFITDYKEGEGISIPQDPMIGCECTDCYDTRKQCCPSGCGTDFAYYKNKRVRVQKGTPIYECNKRCKCGPECSNRVVQNGCRYRLCIFRTANGRGWGVKTLDKIKKGNFVVEYVGEVITAEEAERRGQVYDAEGMTYLFDLDYNDDDCPFTVDAGYYGNVSHFINHSCDPNLEVFAVWINTLDPRLPRISLFAKKDIQKGEELTFDYMMTGSFISLFV*

>jgi|Lotgi1|138486|e_gw1.126.51.1

MDHSYCLPSAKYEYEATKSEEIPVDSDTSTWATSFSAKTDISESDLSDNKKKPKKPPVVKPKKKAEKLKDITNQKPKGGSRELASILPPPTPKPKPVYRPRSYQEERQIFFEIFSRGIDEEDIRYMKRTYDDLMQSDDPMFYWLSSILWVDHPHTNIPDPAPPKKKRRIDDPPLPRTNKTGCSRTEGYYKLTEEEKAPYMWNSRNVSKIPVYKAQDTAKKNIQSQRDARSENRRLQSSFAAITEMSDLLKFNQLKCRKKQLRFAKSPIHDWGLFALEPIAADEMVIEYVGQNIRHSLAELREKKYEEDGIGSSYLFRVDHDTIIDATRVGNLARFINHCCNPNCYAKIITMDSHKKIVIYSKRDIDVNEEITYDYKFPLEDEKIPCLCGAQGCRGTLN*

>jgi|Lotgi1|139900|e_gw1.141.88.1

KKQVIIEAKEPILPGLAVLEYKGIVLLRQHYVDTNLFFKIAHPHVLFYSKFEDIDLCIDSSSYGNKARYIRRSCSPNAEVRHLVEHGRIHFVIFSTKDIEKGEEITIPFDYNYQECMYCVECACLRNNCTVSKFWKKMTKNTQNK*

>jgi|Lotgi1|140849|e_gw1.152.14.1

LPPECTPNIDGPGAKSVPREQTMHSFHTLFCQGCFKYDCFLHCKSYLWFQSFVTRLPSV*

>jgi|Lotgi1|143433|e_gw1.203.31.1

MFKSKRHTKALELYSLCIINAPFPKGDDSPELALGFANRSAVLYHLSHYLLCVQDIKLALKNQYPEKMQYKLYDRLGRCYYFLRQKDEAITVKEFNHCHGIYTTKDVEAGELLFCEKPFASKNMHNSDLTHCQNCLNRVLSPLPCDQCSGVVFCSEECKAEAMKSFHFAECRVLETIHNIDFGLGHLALRMVLKAGLNHILQNNKKYPESFRSDILRIGFNKDGVYDSMDYDTVYSLVKHSEKRSLGDLFKRSVVAVFMVKCLEHTLSSQPLSTKAHLPEKCVIGGHILRHIQMLPCNAHEVSEFAYREYDLPNSQTMEIGSGIYATLSLINHSCDPNVVRHSYGDFCAVRAIRNIPKGTEVYDSYGALYPLTAKKDRQEKLLSQYFFKCSCKACLEKWPLYFQIPNEVPYFYCEKCSGYLSIPEDGKTQRAMCDRCRYVQDMTPKLDVWTRSDEIFAKKLKEIIGGVVTSDALSVLLGHLKTLDQLIIRPFADYNDCQEAIKQCLNLQANCKKVDLYA*

>jgi|Lotgi1|143999|e_gw1.221.12.1

MYNTIIIHSCISQIVSTKFYVVIISGDFILEYLGEVVSEQEFRRRMTEDYSKDCHHYCLSLDSGMVIDGYRMANIGRFVNHSCEPNCEMQKWTVNGLYRMVLFALRDIDPMEELTYDYNFDSFNMETQQVCKCGSENCRGVIGGKTQKNNQIKEKLSSTRPVGRPPKDKRKSKYKLKKFKDKVKPTSANNKLLNHSVIKPMSNRERTYCIKHGVFLIRNVERTKHKQLKQDGVEVKETCNISTTVNNTDNNNITTVIKPGYQLTQREDRSVKTRLVSRVEDNPELQKRCQLIKLFNKVYDTVANYKDEDDNILATPLMNLPNKKKCPDYYKIIDEPVDLTTIRNQIQTGEYETLDSLDKDVLQLFRNVERYCGRKSDMGRLVLKLRKVYCSAKNEVIPLIDDILSDGQIVSNTPPIAPPEIDTESIENRAIRDPPDEEEEIIRCLCNVFRDEGLMIQCEKCFIWQHCDCVGATGNEEYYVCEKCDGRTYDKEIKLVPQPTDGDSENDYYMTLLRDDLQVAIGECVYILRDYKRNSDGTPDIRPLSDYTNITPDKLDIFRIERLWKNKAGERFADGHPFVRPHETFHEPTRKFFPNELFRLPTLEIVPVEVMVGTCCVMDLYTYCKGRPKLVKEKDIYICEYRVDKTAHLFYKVVKNWYPINTKSYCFNTFKEKLNAKRTYSPHQVPEEYLRRTDKQSSTSKSSTDNSSKNTDKNKTEDKNSKKSNKTTKIKKRLSVSFSL*

>jgi|Lotgi1|144326|e_gw1.233.2.1

LPPNWKTAKDAEGKTYFYHSITRITQWDPPTWDQADIVADMDLGTPTFDEFGKKKKKTKTAEADTSSEVEKKLKDIFRKGMSTYIVSCLNPYRKNDCKLGKITCTDDFKHLARKLTHHVMAKELKHCRHVEDLEVNENVKSKAKDYVRKYMSKSGAVYKKPLDDDIY*

>jgi|Lotgi1|148071|e_gw1.5777.3.1

MVIEYIGDLIRNEVANRREKTYEEQNRGIYMFRIDDDNVIDATMSGGPARYINHSCAPNCVAEVVPFEKDSKIIIITNRRLSKGEELTYDYKFDFEDESHKIPCSCGAGCCRKWMN*

>jgi|Lotgi1|154500|fgenesh2_pg.C_sca_6000003

MDKYLIKKDKLLDGSDTTTIDTGIKTTAGNTTDAVPRCSEKTGVPGDIGKEDVPKQPRIKFPAKSGRHFSNSWGFLQRLRSIEGQSDVQLQTIKKSNHLRQSHTCTGKSECRIHQHSNFAQDLDFSTLNTTFMFK*

>jgi|Lotgi1|160845|fgenesh2_pg.C_sca_26000143

MSYNLGTRCFGKRYLNNSFNYVILLTSTNNFNIDFIKHDLSTILAYYRRFISYGSIHVCRRMPHMLPVFQRVFNHYGDKESFFINEEEFNGRYYQATCNLQAYSASINPYHFFMTNLSKTLDGFKLLKYLNKKAPDPLFTGMFPLHACLNHDCDHNIEVRDGFMDDLVGLPGVMMVACRDIKKGTELMTTYINPQMPRNLRRSWLYKSFNFWCQCNKCKFEGDDNTFCTNCQKESQTPNGFKACGKCKKAFYCSTKCQKISWQKGHKSICIKYDEAEASHILEMNNKLRKV*

>jgi|Lotgi1|169490|fgenesh2_pg.C_sca_86000023

MAANVGNWQQNLDDFLNHSLNSGCLESFGCLKTVRERIKFIFNQNLIQNVEWLKVYLDNCDLIHTKSSEKAAKCKNNGNIHFKKRNYGNAIEFYTESVLLSPVNNGNDLALAYGNRSAALYHLCKYEECLTDIERAVESGFPEESLYKLYTRRIQCRLQLNDFQHAGYELESTLTFINSQKDKIESKKFESVMKEIEGLQKTLHNKSESLTETKKTAQLKEHVVSYGTNDILTQASNCIDMRYTKEQGRYLTTNREIEVGDTLIVEKPFSSVLLPDHYKTHCHHCYHKLPLNLVGCIQCSVVRYCSSKCQEESWKLYHSVECPYLDLLHSVGIAHLSLRTVLTAGLQFLTDFIKERKDDESKKTANSRLPGLNERGKYERSYDTVYYLMTHDNDILTEDMYQYSGTAALLLIILVHSGWFNTNVTQIATHIDSTLQADLQSVEIDDKGGTIYANEGKDADIANDSKDNSLTNQKKQNNSISTNEKSMESNIDGLLTNGKTEACQSSSNKTSFCGVLTNEMLDIGGLLLRHIEQLVCNAHAITEVQCTDTINDSMILDTSQVRIATAIYPTASLMNHSCDPTIISSFHGDTLIVKSVKKVLEGEEIYNCYGPHHKRMVRKRRQEVLENQYFFHCKCPPCCEERAGDIKFNAAICNSCTGIIDTTSELPQCLDCGQTEDTASKEIRQQIEGLLHQGVTSLLQHSNTQAFKYTRLVMFLHQGVTSLLQHSNTQEALKFLLLCYKLCCKHLHEYNVIRASVDDSLARGYTVQGDHIKAVHHLQDSIKVTEAMYGSDSIEVGHELQKLADILFNAQRAEEALRVLHRAIYIFTAHYGEFHDTVKELIEMKDCLIDFIGAEKIKDIVL*

>jgi|Lotgi1|175490|fgenesh2_pg.C_sca_291000001

MTSDPARLRNAVTSNVWLDLFKKGPLPNRGNGVFAMQRHTKGQVVIDNGGHYLSGKEGDAKYYAGSDGGMGYMIKFRFGDKWHYRDASTDDGHMGRLINHSACCPNVKGNPIDIFGDGKPVIVYTATKVIFSGDEILLDYNDSQSSAEFLKKCPVCQGDPLRNNALVAEPDNQPPKKRRKQVI*

>jgi|Lotgi1|175732|fgenesh2_pg.C_sca_363000002

MAYVLIKNPGTKQCKLCPHPTFLVELTKHLVGVHGKDLAEAKELNKQKTANQNAVVRIIYSRHVLQHAFPSFGEDAIFTGKDVEKILHGHEDVMDQPWPRQTGTAAPLSAVFRSAPLHMPEMLPLAHSAVESRPSTSPGVDAPLPAHAFLNSLDVPGVSAPQESFTSDNVTPAPPPGTAASTSGTEASPSNLLRAPSPCSSLSSRASEDLTKEDCWLRLKKVALDMFPVAHDREIPKQHTVFATFKDLAEFKQLGGVDDKGVLAPEGKIIIKTKGPLPNRGNGVFAMRRHTKGQVVIDNGGQYLSGKEGDAKYYAGSDGGMGYMIKFRFGDKWHYRDASTDDGHMGRLINHSACCSNVKGNPIDIFGDGKPVIVYTATKVIFPGDEILLDYNDSQSSAEFLKKCPVCQGDPLRNNALVAEPDNQPPKKRRKRVI*

>jgi|Lotgi1|177746|fgenesh2_pm.C_sca_10000054

MSKKGELIAKAEPYVHVLAYKEIDKLCSFCFLPCEKLKKCAACGLVKYCGVVCQKADWPIHKTECPCFKESQPIIPTDSVRLFLRIIIRHMEWQMIDMMLSTVTELTKDHMTLPPAHTLFSFFGMMVINTFSICNDDLQPIGSGIYTSPSMLDHSCDPNAVAIFSGKTVFIRALKDIPDTTPNKMFISYIDQLKPSVERLAELEEQYYFSCECSRCLDTDLAEFTDILSQCERNLEKYSNLAEYNIYRVRLLDLAFDSCINLESWKKGLEYGLQTLAPYRKLYPTNTPNLSLQLLKVGKLQLFLEKTDKALKTLQEAGVGIKVSHGTEHNLYRTLSELLAQCHGELNGKT*

>jgi|Lotgi1|192045|estExt_Genewise1.C_sca_440173

MGDMLWGKVGGHPWWPCMVSECPYTFQFTKLSGSRMTRMYHVQYFGNEGERGWITESSTIPFKGLKEFLDHADEQIQTASKKLKPKVVQSFKIQPTRKCAWDIGVAQATEAFNFDIKERQEKYTFVYDLKQNFTTVKSKDTPGTPPKKRGRKRKVESDETSPSSVDEPNTPVEQNSKRQKLDSREEDKSDDSLVKALFTRPKTVKYEASFDTFYNKHEESVLDEHPDWSGDEVREHLSQQWSIMTDKQKARYKTKFTVDSDTPSSPADEDKKGQQNVNPKSTGTRKVEEPEKQQKQKKSEKKDTPKPPKESTKPPKSKSRRSESVSSTATPEDATEDTESSVPEGKEKKKSRKSKNLLSVPANHDSDSGSEKSEPRVILQSTIKSGDGEYELDIFKLHATSNAKRENICVICEQPGQLLECQGACQGHFHASCLKVSDYQEAEFKCNECETGNHTCFGCKEASPETRKCSVSSCGKFYHESCFKKYPQSRIEGKSYFCPLHTCATCAADNPKNPKATKGRLLRCVRCPVAYHAGDYCIAAGAINLAGNHIVCSKHFQPNKTQRHHAHVNVSWCFQCSKGGTLLCCESCPAAYHPECLKLSFPEGSWYCQDCSSGKKPLYGDLIWIKIGNYRWWPGEICHPRNVPLNIQEKTHQVGEFPVRFFGSHDFYWTHQGRVFLFQEGDKGSRDQSEAKGLAKIFGKEATEAFKIWQAAKANKEQLEKENNSKKPAPFKFVKTNIPIGNVQINKADLSEIPRCECKPDQDNPCSNDSDCLNRMLMYECHPQTCPAAEKCQNQRFTKREYPNSEALKTPSRGWGLYTKVDVKKGQFVNEYVGDLVDEDECRRRIKQAHEDNITNFYMLTLDKNRVIDAGPKGNLSRFMNHSCQPNCETQKWMVNGDVRVGLFALNDISAGSELTFNYNLECLGNEKTVCACGAFNCSGFLGVRPKVSSIANANRVKDGNKKKKRKKKIDIKKEHDDDCFRCEEGGELVMCDHNTCPKVYHLQCLNLTKPPTGKWLCPWHHCDDCGKAAVKRCSKCPNSYCSKHIKGNIFDVEGTLYCHDHD

>jgi|Lotgi1|198738|estExt_Genewise1.C_sca_1060043

MLLRAQMAGIEFYDAVLNNELQLLKRLVTQHKLDLNAKFVEVRKKNHSDLCPIHLASYRGYTYMLQYLIESKCDVNRTTTTLRRTALHFSVLRHKIACMLLLIAAGAKLDAKDTFGNSPCHYAADDGYCQILDVLIRRGVNVNSKDITSKTPLMKAVRNNKTDAVLRLLRANSDLNITDRNSDMALHYAARNGCVDIIDILLSAGSLIDVQNYWGRTPLMEA

>jgi|Lotgi1|200654|estExt_Genewise1.C_sca_1470059

MSTPMDNLRSSTQKAASASQAAMLIKEETNLVVDWKKCVRSEYMKLCHLRRNKRAEEVKTVWGRNRKHIDEQLENHTKYVESLPPNQSLRVRESFSSIPVTRKCEVINKMNFPDQTVPLRVMNALKMIPTMYCWAPLTQNFMVEDETVLHNIPYMGDDILDKDCSFIEELLRNYEGKVHGERPSNFMSDDIYIELVNNLHANYPDIDDNGFKIIAYMAKLNFLDDDDDKSFPKPTAVRDIIFKSISEVFPEKGNPEELREKYRELNEISDPNTLPPECTPNIDGPGAKSVPREQTMHSFHTLFCRRCFKYDCFLHRKSYFIIYAYHPTPSMLTRKIADRKQENEPCGTDCFLHVAGVQRTIKEEVKDDTSEEGDSKSSSGTSRGTGCRNKRGKINGNSASSGEDSERSNDALDKMDGVNSDRQVLKDSDLMYSCMIYNIPYYSYFVYQYLCIVDVEIRYPVRKEPTDPVWNGAEESLFRVVHDMYRNNYCAISKIIGSKNCKQVYEFAIKEEAHMPDLNEERIQTPPRKKKKKQKLWSMHCRKIQLKKDGSPNPVYNYTPCDHPGQRCDESCPCTMAQNFCEKFCQCSSDCQNRFPGCRCKAQCNTKQCPCFLAVRECDPDLCQTCGSDQFDTIKISCKNVSVQRSQGKHLLLAPSDVAGWGIFLKEPAEKNEFISEYCGEIISQDEADRRGKVYDKYMCSFLFNLNNDFVVDATRKGNKIRFANHSINPNCYAKVMMVNGDHRIGIFAKRSIQSGEELFFDYRYGPTEQLRFVGIERDADVP*

>jgi|Lotgi1|209271|estExt_Genewise1Plus.C_sca_30153

MLLHHQKQESMLQQVVVEEEVTDCPMLTLLEHEARVRQKHSSPSKSMSKLIFEITSDDGFSCQADSMEEAWKEVIEKVQDARAAVRLKPMACASVNGLSMFGISHKAVVYHVEQLYGAKHCRNYHFQYHSHTLTEEDEEPVINPSGCIRTEPYTSRKPSDMFSFLMSKHRQRPQVDDKPVEVCNEMIHKSSRRATSMDLPMAMRFRKLREHAKEAVGVYRSFIHGRGLYCKRNIDAGEMVIEYAGEVIRATLTDKREKYYDSKGIGCYMFRIDDFDVVDATMHGSAARFINHSCEPNCYSKVITVDNRKHIVIFASRQIKKGEELTYDYKFPIEDVKITCSCSSKRCRRYLN*

>jgi|Lotgi1|210687|estExt_Genewise1Plus.C_sca_60513

MKGRTWRKRQQKFVTKELVKPIDHTEIEENKHLGKWVKKMGTKKHRLQSRIFPETGRGMMALSNFSPGDVIISLPFNLLITADTVLNSTIGHYIKSYNPKLSLHQALALFLLFEKNKGEDSIWWNYLQTIPKSFTTVGYFSQEECVRLPERLQNSVQIHITKLRKDYRHCQKLWFMRGLYLNFEAFQWAWYAVNSRSVYYKTTCDHLEEEECNMALAPFLDLLNHSTEAKSEAKFNTINQCYEITTGNIFRKYDQVFISYGPHDNHKLFLEYGFILPNNPNSVYPITQEMIFELANQEKVKHFSQKIQILKDNNLLKESTCSNEGVDWKLDKVFKILSCNWTQLQNWKQIILGTVLYLEVVQESRRLSLILLNKELDKTLQQIQSQKPPINYHEEICNNLLLEDLKILQTSLKLQV*

>jgi|Lotgi1|215812|estExt_Genewise1Plus.C_sca_290355

MNCYMQFSITHQSAHAQAMETKESGNIVEHKSVESTPTTSPRNVMSALSITTTPPSGSSSANHTLPSTPTTPLLTPNFVAPKESPSPNTLSPQIKFKQEEKMSRKLKRMSSMTSDPPKVFKKWKDIRWKYWDIEISESLVKVKKHSKSEINELWAKLELYYKPNPMPEDTRVCSFCTLVGDGKPNGPGRLLNLDVDKWAHLNCSLWSSEVYETLNGALMSVDIAYKRGLTVECHRCKKVGATLSCFKVRCANSFHLPCAQDAGCMFFQDKTILCSNHLPKGPVENELSSLVVNRRVYINRDEDRQIASMIHQEEGNHILRIGSLTLHCIGQLLPHQIQTNNFNTREYIYPVGFRTSRFYWSTRELYKRCRYTCFIQDNDGKPEFAIKATETGYKDLVFKDSNPKDAWMQVFTPIEKIKRENDLVKLYPSFVTGEEMFGLTETNIVKVLESLPGTDLLKDYSFKFGRSPLIEMPLAINPTGAARTEPKLRTHFRRPHALQTSSSSRSLPSTVTGVTGDINSPYMKQFVHSKSQQYRRLKTEWKINVNLGRSRIQGLGLFAAKDLEKHTMVIEYIGDLIRNEVANRREKTYEEQNRGIYMFRIDDDNVIDATMSGGPARYINHSCAPNCVAEVVPFEKDSKIIIITNRRLSKGEELTYDYKFDFEDESHKIPCSCGAGCCRKWMN*

>jgi|Lotgi1|217400|estExt_Genewise1Plus.C_sca_390247

MAAPTKTKVNKDDDSSEIPNKISTTDIADQNLDAFLTWCKESHIKLSEKVRISKNGSCAQYGMIAQADIEEGECLFTIPRDKLLFENTTSISDILAKGSKELQSESGWVPLLISLMYEYNNPQSPWRPYLDLVPDFKELNLPMFWDREEREKLLTGTGVIEAVDRDLKSINTEFNKIVLPFINKHPDSFMPICKEKDFYKKMVAFVMAYSFTEDTVANETITSSPMMVPVADILNSIAKNNAHLEFDKDVLKMVAIKSISKDEEIFNSYGEPDNAHLLQMYGYAEKYPHNHNDTVEIPFKLIFDVARETNSENGGEEILDNKWEYLETMEIVDEDGSIVIGMTGVLSDYECEKVIKILLMRESEFDNFIQEEDQEDSDDEVEENIFTLENIPKLAQEWKTILQRWCYTRLKSYPTSLTQDEKKLAETEKLAPHYKYSFYTAHSQKTLLNNIIQACNKG*

>jgi|Lotgi1|227153|estExt_Genewise1Plus.C_sca_2330004

MITEPLEPTKIPYFEPIEENLYLTERRQSKEMRRMACDCTTSKEDREMGVEACGSDCLNRMLYIECGSRCACLDYCTNKRFQKRAYSNVEVFQTPWKGFGLRAVEELKTDDFVLEYVGEVLDFRGFKNKSRQYAKENQTHFYFMALNPDVMIDATVKGNISRFINHSCDPNCETQKWTVNGELRIGFFTKRTIPPGEELTFNYQFETYGQKAQKCFCGSENCRGTIGIEKPARFKSNKKKDKTKKKEEMIGVDEIEEQIERMNVEGLTNKNHVLNLCRLMVYVQQTDHRIAILKILQNTPEPACLRLFVEYHGLPLLWSWMADLNQEPASEFQFQVLSLLSELPITNRTILQDSKILSIIEKWTETSEAESYIESYTELPPPPPGMTPVGILSCTKDTKEAKDKLHKKRVTFAEEEPSSDSEMSDINCIMAEASSTISISAEGIEVSGTDNTMEVTGVSSEGNNSGQDVEQSDEVESSQDTVGDVPQHEADSSGDTREKKEGEYLIEGSATEEESQDDESSSDSQDKPTTSISTVASKLLEHWSNLKEVFKIPRKERVEERKRTEQELGTFFVLSQTCLLFAITDLNTNL*

>jgi|Lotgi1|228668|estExt_fgenesh2_pg.C_sca_30361

MATKVNMSKFLCDNCQKSLDKMKRCTRCRLVYYCSQECQINDWSKHKENCQSVKNLENKETNSYNESEFGGNADATTSLEATIKSCESDCSGIKYSRINFEKAANDLKSEPTVESFLEKIPSSNDENHKFYYLNNQSFYDVPMFKSDLPYNKVTVKSDKIKRKFDINLIWTGEEIYKFLSSELEIPLEKLKIINKGKVLTKETISDCVAALKPVFQILGEKSENEEGLDKNDIDVMMKQLGIERNSAVKTLRQKGDLIDAIIEAGNKK*

>jgi|Lotgi1|231752|estExt_fgenesh2_pg.C_sca_210212

MAASMPANVEVQFINESKGRGLFARQEIKEGEAILDEKPLVSTQFLWNELYKYTACEYCLRSLETAEAMARRLTNNPALSLPHPECCALDPSEFVVCPQCQVLYCSEECRKASWDRYHQILCLGSSHHDSDHPLLRLQEIWRNIHYPPETASIMLICKMIAMVKQAEDPGHVITIFNKFVNNTVNEEEQIAHKLLGDQFKCQLELLRSTTAEILFDESIPQWFTPEGFQSLFALIGTNGQGIGSCSISVWVKNCEDLELPEDKKTELDDFIDQMYEELEKESGSFLNCEGSGLYELTSSCNHSCDPNAGITFPHNNHVLTLVALKPIQPEEEIYISYISECEMSRSRHSRQKILRENYLFTCRCRKCDFEADEPDVTSEEEMDSGDEDGSEKMDNL*

>jgi|Lotgi1|232186|estExt_fgenesh2_pg.C_sca_260134

MGELDTKAEIELRKLENDGQILFENQNYEEAREKYSALIAATDVEDPHILLKRARCHMNLNDYQSAYADAKHAYKINPKSLEACILCGQASTQILLFEEALNYFNDGLKIDPKNKTITLNLKNLHSKILSDYNEKGKGAETTYSAVKFCTQDPYPGDSDLLNLEYEILAKKYKIPSEDKVNFDAVNKEEAAKHAALAFRLRNQDLSSAIHQCTLALSNEPPNLIYRQLRADMYLEKGDHVKALSDMWAIPKQNRGPDIWKAGGKVLMSLELPISAEFWFRRATKLSKEGDDEAAMLFQQVRVDRLYRPLTAGYPVKVEFKQFGRGVYATEDIKEGDIAFVDSPVVRAMISNPEHKIEACSHCARSLLTAAQYFGDALETMTEEEKELVNIHWPDVTPIYCDDCRRVKYCSDDCRLEAWDLYHQIICPKLNPASSELYDLLDNEGWGIRDDGTKGEIWGGHYSLMILANIWASIIMEAKRLMITDGATTATVTHWAKAKAPYRRFIAYGTTSAISRMPHMLPVFRRVFKNSGHEGVVFNVTEEEFNGRYYQATCNLQEFSARSTPYHAFMKKLSTDLRGFQMIKYLEKSPPYAGFCGMFPLHACLNHSCCNNVEIRDGDCNGTPGVNVVAKRFIKTGEELFTSYIDNKLSRNIRRAWLYKSFNFWCQCPQCTFEGEDKKECTNCNAKSESEKAFPCCSKCKRAWYCSVKCQKEAWRRGHKNICSKQKSGTA*

>jgi|Lotgi1|59489|gw1.3.101.1

RNVCYLCGSLGQEELLYCCICCEAYHTFCIDEEDRPSNDNKDNWCCDNCQFCNVCGYQNNLLSCDRCQSTYHPECLGPNYPTRPSSKKNIWVCTKCVKCKSCGVTT

>jgi|Lotgi1|86198|gw1.69.204.1

RKKDGKMHYECNVCYKTFGQLSNLKVHLRTHTGERPFVCQTCGKGFTQLAHLQKHNLVHTGEKPHECMVCYKRFSSTSNLKTHMRLHSGEKPFHCKLCPAKFTQFVHLKLHRRLHTNERPYECPQCNRKYISASGLKTHWKTGN

>jgi|Lotgi1|153726|fgenesh2_pg.C_sca_4000185

MEEVQNLEKPNEVEFRPSEVEIGKQGIWCIKKIKEGTLFGPYCGEIVLHDKQGLLDYRYAWEVWDLESDKLLYIINADKKNIGNWMKYVNCARYFEEQNIVSVQIDNEIYYKAIKDIEIGEELLTWFNLIDKNDDSLILPMEEEKKKHTKKMPSPKKSPKKSPMKAISTQTSPSKTKIASTSPAKRKSPSNMDASTSNSKKQKFVQTRMNFKISKKPRDKTKKNVENETVLNNVNSNSMNGETTEEVAKQLLSKDDKRIFRFHTQKKHHFVQDGKKMYKCELCSVVYKRPFSLKRHFLRSHINCMYLCETDVTNCCIDLDNQLMQIENAQNLQASGSCLFNGLIRSHTSSPMQDEKLIESDSKLNKKDSKTQEIDSSFPGLYKCNDCFFVFDDSKDLVAHFKCHDSKIGTKTPRCQSTDKKYNSKQKKSLTLPYSLKESFDSLYISEIPEGTKHLKSPKKTFEKSSMKTSPSKRFSALESKNLPQENETSPVSPSRLSKVKSPNTDSKEVVNGKGKLTVAATPTKKSYPNSIKQVKRSPSKSKSGSINNKESQSTNSPMKRLHSLKSPGRRETLAKSTSQKNTPSKTKFKSPAIEPSIKSSLRSRSGSNSRSPQKREDIGEKKQNFGTPKKSPLKSPGRRPNFSSPFSPKRRFGNKSPRRSSSRSPIKRKARGSKYVKTEFKKFSCTRCKVKFSRKANLDKHLKEHTENKTCPCYICGKQFMSETKMKLHIRYHFSQNVRCRHCDLKFPNVGAMRQHLTETHKDVWLINSKTTENTDESGKSKKKSGQRHKDKIKVVSDLDFRSKEALSELPKPVSIIPKSATKILSPPASHNNFKVLSSPVAVSKKKNAGATLDNSPAPNGYKSKLRYCCYTCKKRFISYYALVQHRRTNHRYNIFSAPSMLLHMKQKVSEEKNPLGNMPILSPPVTPDPYYETVPENVSNNWSTHIDGKSEALKNYRKHIQIKGYRSIHTVAQYKPEVFNWSCYNFPFHYEPYESVIGYNNYKPQPIIAEDLSLNNSIMDSLHNKVNFTPLKLDISSAEAAPILPCMTSPLGQNQQRRFSNDSDSKSMPSLSPTCQNTKFFSDVTESNGIRRDRYSDTCTDDSKSCPSSSGMVTDEHSAVKSNGDQSAFNKTSVKDIENESFVPTLQYLNLVKMSDKETIESAIQAASRTTPAQSYQRDFSDRLKTQSWEGYYSDIEFGKLGDIVNVCSVCYKYFDSEETLVRHQWTRHSSMNCRHLQMEKGHDIDNLFYPWPRNEGMVASSTPVPEDCHTLDSYKCSYCKSGFKNINRLHVHIISCDPKKLYYVEKMSVKERRKQEAKERKATTQNIDTTKKKSAASEMKMKLDKRRKKEKLKKLIREKQTAVFPANNKLPINVNKTQQGLSPRSLREVRRNEMDLLSNSDSRVTRQRKRKNYELLYVPKNHVRRRTATKVVETHQCSGCSSKFRTLFLLERHARKCSEKDKLLFDASIEDHDTNVNSKTQACEFCKKKFTYVKSLANHYNNFCPVKKQKLASEEIEEATVIESEFKSETTQDDVDDEGDADDLSEEPTKRRCGWPKGLKRKNRRKNHSWTITKRKKTSSDESDMDKNNHKEAHKGEDKIEKDSIQDDVEQDSNDSTNILPGDTSADDEPSPKEKRKEVTEDSSTNVQNGLTIKESAATMDIIKDKEMSANSCVQIDTKPDNESYIACSEVVPVSDDQTEISISPKLADNSMSPVSDNHCTEKNQGLHTKPDQNSTQHVQILLQSSNTKLDGKVEDSKSETGSYVDQNPADIADNGYHSDNSNSNSNSKSTKLEASLKNGHILLETKINSELGMSCLNENTEFSTGFKSSCENGHSCTSDLNGSSSIDNTLPVVLENQVPYCIKQSVESVCLISKRNQKVGLFENNKPSLNSTDSSVFYENGINSHESIENPRKELNNTLVPYQNGDENSEMSNESDLMNESDQNGFYSADTETDTDIVCYNGDTDSEESVLSKIINTEPECKLDGSNISPIS*

>jgi|Lotgi1|65785|gw1.31.124.1

IDVSEENNSNWLMFMKPARNKEEQNVIAYQAKDCIMFVTIKDIEPQEELLFWYSKEYAKHVGKSQPLSCILSFFRQEMSCNKKYCGILLMTLLKHQRQKHNEIPIKNHKCQYCDKPFGTMGKVKRHIITTHTNIRPFKCLQCGKEFADKSNLRGHEKIHSGSERQFKCTLCEKSFRQKAHLTSHMYIHTKIKGVPCPHCDKMFSRKADMMQHTIVHTQKKIYQCEQCSKIFYKQQTYKKHLRIHTNEKNYECESCQKKFHTKYHLQRHFKSCKNLKMNTMLDKLSERHKDKSEENSI>jgi|Lotgi1|69958|gw1.218.51.1SQNWCAKCNASFRMTSDLVYHMRSHHKREIDPVKRKRESYKLKCDVCNEVFKERHHLTRHMTSH

>jgi|Lotgi1|156439|fgenesh2_pg.C_sca_11000125

MGASKFGQKQLDFKSKQIPNEVDSFFTKKQLEEMSDYEKNRYKNLRENYEMMVLMGLPVKKPGFMTAKWKRKKSVEDSESEKESDEEWTPNKSSKKKAKNCKLVPRFHVPFKTTSSTATSTVPIKTEVKMPRKRRIISCNVNIPTDSGKKVKKDVKSKPEPRYPLRKRDTCNYMNIEVPDDDEFLYCEECNMEYGGDCPVHGSYNIIENKQVEESVSQKDKNRCYLTAPDCIEIKESSIPNAGLGTFASITIPNRSRFGPYGGDIIKDTETAHNSGYCWQIYQEGKHHHFVDAKNPATSNWMRFVNCARTESEQNVTAYQYCGEIYYRTFKEIPPGSEILVWYGNEYGADLGINRPLWSKSTFVAYHENMKKGNKFSTFPWRLNGAKPAIEDVYPCSFCQMSLAVPNLLLKHLYSRHYDTINIKKLRTLLNGDLKSIHHLSKIQSQQVLELIQNDDDLDSKISLNICKENILPLPTKHTGEKPYKCNVCSFSCNQACNLQTHMRTHTGEKPYKCDVCSYSCK

>jgi|Lotgi1|180523|fgenesh2_pm.C_sca_181000001

MQSLASKSIISSHLRTHTGEKPYICEDCNKSFRHKSDLTRHLRTHTGEKPFICEVCSKSFSQKSDLTTHLRTHTGVKPYTCEVCSRSFRQKSHLTSHLRTHTGEKPYICEVCSRSFSHKSDLTRHLRTHTGEKPFICEVCSKSFSQKSGLTTHLRTHTGEKPYTCEVCSKSFSQKSDLTTHLRTHTGEKPYTCEVCSRSFRQKSHLTSHLRTHTGEKPYICEVCNKSFSHKSDLTRHLRTHTGEKPYICEVCNKAFSDKSNLTRHLRTHTGEKLYM*

>jgi|Lotgi1|104847|e_gw1.3.24.1

CEECMTSYSPQCPVHRAIVVSDKIVLSRAWSSLPQMLQIYRFNKIGVFAKKPVSKMTQFGPFVADMVSSQDDLTNTRFLLMVLMNFFLYFQLEKNDGTLGYFETSDENKCNWMMFVRPARNFAEQNVVAYQYGQDIYFTVMKNIEARQELKVWYSAHYGARIGIPIHEITERDKEAMDEQEYKFPCYECNKKFRSAPALQRHLVVHEEAAARWFRNKGKQMKNGEGGESSGYQPDSEKIRRTIQSMYKRKGKETGGNEWVCMHCDLTFDNSSLLNLHTLTHAAEDVGLEEIQKLSSVAASITSENGEPSSDNGVVALNTILACPMCHAKFDDKNDLIAHATQHGTTKKKSLNALRPHKCTSCWKAFGTQERLNKHMLCHGSEESKPLECNVCHKRLMNNSAMACHMKIHSDKKYYSCPMCHEGFDQTHGLKEHCATHAVNGIYTCQVCQKQFPDFNQIRKHMRHLHSENEYPCLHCDKVFARPDKLKLHMLKHSTHREFMCENCGRQFKRKDKLKEHIKRMHIEQKETKITSSDERDKNLKKFVPKVLPTDYQRFIYKCHTCLLGFKRRGMLVNHLAKRHPDITPDLVPELNLPILKTQKDYYCQYCDKVYKSSSKRKSHIIKNHPGLDLPASSRKKSLTLDISGVPNPTYSQTVGSITTLPQQCVFCHKQYASKAKLMQHQRKKHPDLVPPSNDRRKSGCKEDQITTTTVQLVDPNQQQLEAYQEVTVTQVPQVHDLQAADLLTQAMSELTQTLQEYRPATGGDYHALAARIAQGVQGPAMVHVQPAQLQQLQHTTIELSHLGSTLAHAQITSSQPPQLIPAGTILTGTPQPPQSLSPQPQAPQAVPVSLVVTNPGTPNLATSVANGLPQNYVQGVRQNWQPYQGFR*

>jgi|Lotgi1|130157|e_gw1.68.116.1

ITNVTKPKQVEISRSTIPGCMLGVTSTTWISTGTQMGPFVGRIVKLEDIQNQPDITDNIWEIFDDKGVILYFIDGGTSFPRASWLSYVNCARNPQEQNLELVQIGHNIFYRAIKDIAPQKELLVWYGSSTKLYLGLPTPDLTIEKSSRNLCTDTTPFKFVCKLQCVLCRRGFNSKSNLRSHMRIHTLEKPFVCKFCDRRFSQSSTLRNHIRLHTGEKPYKCNICRSSYSQLAGLRAHQKSARHRPPKPQEEEELHVD*

>jgi|Lotgi1|130257|e_gw1.69.247.1

MDGDHSNQWMIYIQPARDRHEQNVEAFMDEKEEVCLRFIRGVKCGEQLLVWFHDTLARYRDIPILKPENIRGYQEYTCTVCQKVFKFPNSLKAHIRYWCRKEEVVTSSGMVHTTASQQPANLHQPIQIFNHIHTPAFPITMNSSPFSLYHPVLSQYSRSCCSQLPAVHPFMDRPFISSCTSRYTPPSSSSPVSSTATSRRGHLCIYCGKLYSRKYGLKIHLRTHTGYKPLKCKVCLRPFGDPSNLNKHVRLHAEGETPYKCQFCGKVLVRRRDLERHVKSRHPHEDVISSEVEADSSTLDSSLDDNDSDSTEIDSELIVV*

>jgi|Lotgi1|114994|e_gw1.20.93.1

GLNIIQTNLGGILHFGVFSKKSVITKGTRYGPFKGKVVNTSEIKTFDDNTHMWEIFKEGKLSHFIDGRGTNGSWMSYLNCARHIAEQNMVVLQEGDEVYYYATRDISPGTELLVWYGQDYMHFMGIPLTLPNKDSVEKVAAPAESNEGYQCERCGKVFAYQYYRDKHLKYTKCVDQGDRKFPCHLCSRSFEKRDRLRIHVLHVHERHRPHKCAVCAKSFSQSSSLNKHMRVHSGERPYKCVYCSKSFTASSILRTHIRQHSGERPFKCKFCGKAFASHAAHDSHVRRTHSRDKGHTCTLCNQEFVCQIDLKIHQRTHSSTGKFLQRFNNLHCSLKEYLGVGNTYKNAYM*

>jgi|Lotgi1|169169|fgenesh2_pg.C_sca_82000111

MEQIRPNMFPFFPGQVPPNFTGFPMERLRFTPQQQIVPFNSPPVFPGVPMTHHQMQQNMMSPRVPPLPPPFPGFGFSQDEVDMILYGYTKSKAGDQCLGHALSGLRIGDLTHGMEKILSGSPQTPSSTTSSSSIKSSPTSICESSSLDLPEGIVVHQTGFAGTLHHGVFCAKTVISKGVRYGPFKGRVVNTSEIKTNDDNSFMWEVFQEGKLSHFIDGRGSTGNWMSCVNCARYAQEQNLIAVQHEGEIYYEVCKDIPQGTELLVWYGDCYLQFMGVPVALKEMADGGAQDEADTSEGFSCERCGKCFAYKYYRDKHLKYTRCVDQGDRKYPCHLCNRSFEKRDRLRIHILHVHEKHRPHKCLVCGKSFSQSSSLNKHMRVHSGERPYKCVYCNKAFTASSILRTHIRQHSGEKPFKCKHCGKAFASHAAHDSHVRRTHAKDRPCTCNICGKSFTQPFELKYHMASHMPQ*

>jgi|Lotgi1|88101|gw1.186.69.1

FPCENCDKIFTDPSNLQRHIRSQHNGARSHACVDCGKTFATSSGLKQHQHIHSSVKPFICEVCLKSYTQFSNLCRHKRMHADCHRYCCKYCGKHFPRSANLTRHLRTHTGEQPYNCKYCERSFSISSNLQRH

***Mnemiopsis leidyi*  (Metazoa)**

>ML001115a

MFSQFERQYSDFLRAHLTHINIKENQTSERQLLAHINSHIQSCSSSSTPDSVFTPTLTKSSKSDDLARAHVRFAEELLEGESYVLALSELCEAVKNSTSAESLVETLVVRISVLSKLKLDHEMKADLRLLKSVCCQTCYNYIFNSKFDRKCNQYEPILRKFGPVERCTLNKSKREQIREIALNQTLESDENVVPHNPPRENILPCLSTETSLSEEYSFVCSKVQLAESLDRGRYVVAKEDIKIGEVLAVEEPRVFTLLPQRWSTHCQYCLNVCRAPLPCLNCPDVIFCSTECLSKDSLHQLECGYMPELQSLPKMVYLAFRTVLRYRHEIILHSRNKEISLEFQQLLDLITNFQLRSPIDLLRRASLTLFLSNIIDSLSVFTPCRKYSFINRPDKEFRSTGDDMEPDRELLCRKVRVGDVDICIGGSCLQGRLEKSRVLATAQIVLLKCLQSFPCNAHEISELQITQQFRRRTVLRSLRPIPAGHELLDNYGYHYATHSAGERRRALRSQYLFTCGCVACSERKSWPNFENVPSRVTIRCNRCDAPILTKRTICESCGRNASKSLQKIDRLANETSRFIQRFINDPNAVGSSVVFQFLEMGENHAVLPFRLHNECQEVAKQIWNLDSNFKTHLSRT

>ML003211a

MNSESIKRLETKLGGTPCIPYISDETGVGLRASKKIAAESILFKEEPIISGQYSWGIIYGYEACHHCLKSLETPLEMLRRLTKDKSLMLPLPDQEPSVDPSSFEFCPGCKVAFCSKQCLNNAVDQYHIAVCTNGDSDHPLTKLDELWRNMHYPPETTSLVFIVKIIGLVVVSGKIPEIFGEFYQTVEKSGNTHKVLQPEFIAQLDLLRDGLLQIFGHNSLVQQILTRENVISLIALIGMNGQGVGTSSFADYCKRLDNLPLTKDERDDLDSRMEKLYDKIDEEAGIFTNVEGTGLYELQSKLNHSCQPNVQIVFGSSNDELSVVALRDIDEGEELCISYLSCCQLDSSRHSRRKFLLQNYLFECECTKCQAQINDPDVTSDEDEDSEEEMECS

>ML01132a

MRNTGLNGMKRNAQKDIVRCICDFNPGIGDSICCDKCSVWQHMRCMGVHPNRVPKIYECDQCNPRPLDKEAAVQLQLLLREEESSSSDEEPERPQQAAPRKTHNPKRPRKASSNSIKSEESNADLDDRSDCRRTYKEVKENMYSPSIKHRLEARTEGLPSFGSSPPGLQRVQPQATGYTSPRIQYPAPLHLLNHGPSKIVITKHALKPGTFISDFMGHFKQLSEYYEEVGGKSTLYKRPMNYVLHYPLLDICVDTREYGNIARFMRRSCHPNAHAKSYLQEGKLHIGIYALELISPNSEITIGFNFPVDQYMYAQTCSCSSSSCLVNKNNESLTVRAKKPFSRTNSMDSINSEDDHKKHRMSREEVKMQSYIQVIEKIERDEERKRVLSENLKSPAREVADSTEDSNTPSRGSKSRRRKGSGKKGRRASGSSTAGNTTSNIATTGKSPEHFTFEGDRAGNSNNNSPVTSPKVFKPSVPAYTIMEPVLTKHHVQCNGYEADIQLRPISTAPTSRTRKNFQLRSSSFHITPIKVQKDGFIVPIHGSLINNKPKTNVAVYKTSKKRWLETFIQEGANKCLSPVPSPKHIYSPSRENITPEKEVTSPGSQVALKDTESLEAATRTLNLSDDTLTTSDVVTSTADQMDVTFSEPSVHPPAEMDSTALPHLSSSESCSASVSVVSGDAGSATPNPGTIEKKKRVTLADYRKRLQSRSSKTPSTSNEADCKVEPVSPKPRPVFEPISPIPQEKAVSPQSSLLEEFTRPSLSETISDESCHPNKIRKIDYSVSPTTDKVNNELQSCESTEKTSNGGPHSFDHPSHALSHDTFSKIKEILSQSNPGLMPSRNLNVSSLKTNLTMYDPNGSPVKQYHQYPGQYSDEMYSRDTKRLKPSVEYPAKYYKESDKR

>ML015713a

MPGNESYEDPYMYIANPRPKTVTRGRGRSNKSRNYNIYHDGSSRPDGSIFRDYSIKDEDCGPINRKRIKASHYENFVRKHPGGTKLCLLCKLEDNHLNCQGPLKWFPVKSKRGPGRPKDKGLPKTPRLMATFLSNTLEIAPAPKVITLKTEAGAHSEPIKIKIDMTKGGECLPNGNGFLDPDVIDSCGFYIHVECCRWTTSVAPEIEMFKAERYLLKGLGQSCSHCMMNGATVNCRFETCKKKFHVVCGLLAKCVPDMNSSSMICSEHLVGQSSGGQAACSKCSEDKFHNSMLHCMSCGSHTHKQCLSPDVQMKPLVVAGWQCGTCRTCQSCRKSVVNCTTAIIDCIICSKSYHLECIEPQQQLNIQPSQWRCKVSSLSMEKMNFSCTLQDIFITYEMRRFSRNFAIFSDRQNFGRRNRIRRIIFSLFQ

>ML015714a

CKDCQVCEQCSNPSDESRLLLCEDCDISFHTYCLTPPLEQVPTGPWRCSWCVKCVKCTKTTPGVHATWYQDYSLCGPCHSQTTCLVCNQEYVAGEFCVKCFSCEHLMHGRCENIASEETCPKKFVCSQCEEMVKKEGETPPKKLQSTTFSATKQALDSVIKHDARIVAYKDKDEKEFDAEREEKEKSRMLGYDYIKALNKQRHEHQLLQKSKKFSAQDKDPKMNELIQEGLTPPKDFSSSSAYQQIVRELTGNMPGNKSAVKRGRGRSVNKASDLMSPTKDPNAPKNRGRPRGSKNKTPSQRTRGKGIPHPEMLQQQLSSDDSINFSDFTGMGLPVRPPPNIKSPMKAPLKRSNSNKGRGKLIPQIMDPSQMMKQEISPAKMNPELAMNFFGDELMPAPGPDMMSPPQLMASQTMGPPMPMAPPVLSSPVNHPVNAMLPPQPGKPPYNVQIKPRNKLPPSPLLDPLQNKNSSVPPSPNNMNVLPSSKPQTPIKKEPEPFDLSKVNIDEINVCPESPFAPDHPDTLPEQEKIQAMEDQEMLRQSQRERRQRLYERHPTANRRQDGMITGNDPMMGGWQRFRTPEETSNSTVTVGPIPSKAGMPNQMPTIRDRPMTPAGPMTPGGPMTPGGPMTPGGPMTPGGPMTPGGPMTPGGPMTPGPIRSPMNPRAMATMNVQMMQSQMMGGPGYNMGRHFDHTTGQWVPHAVPGAHTPLRHLPPQAIIAQQNMQAQFNHMNQFGMQPTPEPVQPPPKTKKRTKKKKEKAPPVELPTPMDPQVNLENFPPHLRFGGPQTPAPPSSEFQGAGPGHPGFDYTEHFRQNIQMGPGMQNPMSKNFKRGNSPLLKSPQTSVDMERPKSLNINNSSPSATPKSHPNTPNKGAASKNSSPMIKNTNDDFSNDSHDSQQQMHQKHAIRIALEQRKQQQSHYMSQQMMMPSNLHFPMNVPQFVQFRLQQDWLKKFNQLKMTGGGIPQPPQHPNSMTNQQIQALYNRLMENIFNMTAGRMLGPGHPGMMEPMGHVIEEEKKPKTKKRKKKKAETPVKKDDKSKMLKIGPNLVMQTAMGPGPLGANSPQTAQDNFTGPHGINQNSLGFQQGMGNQMPQNIPGQGGGPPLGIINPKHLNMRSPGPAIMAGPHVSHIPGQIHQQQRLMAPHYAIALSRNSDPRHIRPTLTTQSPHFHDNYDIEPPRTPKPTPKSKSKSKTKESLPSSVLKPSDTPMQADSPRYQFETPRYQHQIKIEDNQDFSDVKSPQSQRSQTPSSAGTPTMKDPFDNPPLTPSTYNSLESAKRRKRRTKAEMDEHRAERERVGYNGPPIRAANKHIPPVQQPDIGKMVDIVVRGTGTSPHCKLVGSYGRAIIENQRDFYRDLVAQIKESRQKQAAADIAPGLILNDSKPQLTSYDKEAVKLHKKLSVQEQHMEKLRANIPVHHAPTVLTPQQEKMLCQRFGVTPAEAQRMYYYQQHQHMMALREQQIKAYQMQEELEQAKSGNNDSDKSKVLEHIRLLQESSREKNSISRGNSPAIAMEKIKQEENVSVSVKQQSDTIQLIFQRPMNEKTESKEQVEHEKPLLKPHKPMLELTKPTPPAHIKQEYMNKEVGSDNSMSPRELPFAITGPDFTPSIKPCNPPTDEKTTRVINLHKAFQMLSDMLGMKLKKDNLASLNELRVQNIIPGSLPHNLVCYNCIIVLHPNKLKHIAPGRPYYGIKDEHGATFCSDSCLAQYYCKKFSSLRPEDHVTPAPPPLRQPISSPLPSSPGQRVLTSPGKRRRQSESWKYWRLEDVVGMDNHRQNLADDNKRLFEMFKKWHEEHTTQTDAVDQRVCLLCGSVGDGSPNGSSRLLNYRKNTWIHLNCALWSNEVYETRDGGLVNIPQICKRAQDTTCCCCNRIGASIVCFKAKCNAAYHFLCARHSKCAFYTNKTVYCAAHKFQGAICDDFTVRRRVVIERDVQQLLANEGFKNPGDKIRIGPMIFHHPGHILNNTSKFHSRDIIYPFDYSATRIFWSTEDVGKRCSYDCSIDVKNEAPLFKMSYQKQGKTVDIENVCVDALWKEILNSIAELRSKTPDILSIHVKFHKGTFLHGLTERNVLRIIEGLPGSDKLEGYTFCYGRIETVTLEHLVPFNPSQSARTEKYTKHTKRVGGHRQISSSIQGSQAPSLWNREFGMFHDMLPYQKSFNRASQYRRLKTEWKLNVLLRRSGIQGLGLYARRFIDRNTMVIEYVGQLIRTSLCDQREIYYNSKNIGCYMFKIDDVVAVDATLTGGPARYINHSCEPNCVAEIVTFEREKKIIIISNRKIRQGEELTYDYKFDFEDDSEKIPCNCGSVICRKWMN

>ML01861a

VGREQVLHSYSSLFCRGCYKYDCFLHSWNKRPGPVKRRRSSLPAWPCSPDCYLHVYSRPPTPARRADTDASIATSTDDETKRKKDSQFMRERDEIASQWTCSEESLFNALSPVFQQNACSLATVLNSKTCRQGTSLSAHKKTFQKRFQKVLKKAQENHGNLVGSFVPCDHPGKPCDPCCSCVRGGHFCEKFCNCSRDCSNRFTGCKCKASCRTQMCPCFVAVRECDPDLCSTCGAGEDMTEKCSSCKNVCIQRRQGKHMLLAPSDVAGWGIFVKEACNKNDFLSEYCGEVISQEEADRRGKVYDKYKCSFLFNLTQDYVVDATRKGNKIRFANHSVSPNCFARVMMVNGDHRIGIFAKRNIEAGEELFFDYRYGPTEALKFVSIEREREFL

>ML021115a

MSYPHYPRPPPNVTSYPSSLSVNTNLGQTSATPPLTIIPSGTQVLAYYQDYAWYFGVVQSYNLTRSGSLNYQVLFRSGEIRSVSSSQVLSQQSNNQNIEIGSRVVVQHPTKSMFISSLVAELPGVVNFFRYLLFLDDGSACYKTKDKLWVINLNENPILMLPPPYKSFIQRYFTMTGSTVRLMNLNPNDSVNFTNYDNTNLQFSGKVVEVDCNIALIRYSIPSGGLAEEHIFRGSSRILSYGHNQYYSAADDLLKQNGAAKQIQHNNNFDANQVTQDQQTFIKSPLKGKPFMVPPSLDPFMFSEEENSMPSVSKSAKYNTFNSWPVSNNTSSRNTTYGNQVFSSKDGTLVSQPQSPILILDESPSKDPPPYSSPKPPISSFPSNPSPSKPCNENFMSPSSMSLSPHAYQPASVFSWSASEESGTKRRLDLSPELNTDHDVEFDFADTILNTPVSSTVTPPTFSIPLANKAPFHNHHDIKPKIKKEEVVIIDDDDNLPPLVIENESRNDEVLLLDATQIQVKESFKNEGATRNIPMILHQEACLDNYTAASAMETTSQNGNVIEVVVHSSNNGEAFISVTPSVEVVTAPAETVNTTEEVKEPVKQELSKPLTVEIKEESSTSPDATISRKRRIISPIKSPKVAIPKVKINLKAIAESAKDLAAQKCVKRARKQQSKDVKKDNKQAIADKKKKMAKMTEMNMKSLIMTKKEPIKLNSEAKIARWHAPWSCKTTGNIYHSKQKQDSATTFGEILEKVSVLKTDEELEEGEIGDSDGEESNEEKPTVSIVLPYQNPHTCSFLCNRFSCSDSSAASPFSTPIQLGWERISNNGKVTYISPCGIVCVDDGDVSNHLLATKCTSLYSTNFCFLPEISLDLSSLKPEFEPLYSDNDLSNGLESVPVSFLSNIGPIIKYSFHYVIDLIHDFTSNQFDPELTFCTCEGLCSRSCPCTADRSSKTECNSHCSCSVGCANRVVQRGLKHQLQIIQDDTSFKVKALHDIPKNSFICAVTGVVKKLAPSNDSFLYPLSQVPYAAKIDEVPASTEVVLKHENTKSNSYNSFYHDDPNLIMSSDLESVNRTSDRNSKSNQHNVYWIKAASRCNNLDSLLNPTLALSYPSLLVGRREPTQRSKRLSSNSRDGCIQSDKSELYLDCTEYGNVGRFIPIGENFNTEIQPVIIDSTSIPWLAIFSTSNIAKDSLLVLKSFRR

>ML02412a

MVVNAVPYYEQLACFDDYATALVVDPILGFTTHKMSSQWKSLRSKKANVEPIISEYRLNQDAELTFDRLMECHPHWYKYFTSLKTSQQFKVFKEHVIKYIHIQHPLSGFMVSICHRFSTNDRKECKIRVTKKWCAGEFIPKLCGVIAEMSPEEEANFITPGVNDFSVMFSTRKNISQLWLGPAAYINHDCKPNCKFVFVSRDVACVQVLRDMDVGDEVLVYYGDFFFGENNEHCGCKTCERSCKGLFSTGNGPQEIGVQIKGEDRSYSLRATIQRSNPQPKRYPHLSLYLGDEDEDSIYDLIPRPVIKTDVVIFSKPLSEDSDESSFVSTSRECRSLTRSDTRSRVSKSRENRDSKSVEPRGKRKRESKTKQTNSRDSKEGMVSRSGGQAARTKTESSSRPSSRCSRLSGRSDSSCRDDVPHNATPGKRKLTRSSCVLQLVDQRKSTKVVPEVRAITTRSSTKTSVNTRSRSLCQHTVPLPGLDSTFNSPKSRDIKSPRALRSREGVSRSNRSSPGSVSSNSDMILRKVSVPRSDRVLRGQRRKT

>ML024912a

MVVNKVPVTPENLKQQTLDSHVKKSKHGRHLAKKIDNADKTVVKEEKSEQINENLKTEPNSEKQSKDKQIRVQPKVEIKKEPKKPVVESTKRGPAKSAPPARASRTKKEVKTSPPADDRCQKLTDYFPVRRSKRQPLSKIKDLEYEQLKEKILNKCTDGLEIRQIEGKGRGVFSTRHFERKEFIVEYSGTLISYEEAKKREAEYVKEPEKYGCYMYYFVFKNKKYCVDGTAEDGSYGRLLNHSKNGNVESKLVNVNSKPVLVLVAKQDIQPQTELCYDYGERSKEIIDSHPWLNQ

>ML02591a

MSDKEKDQFLGFDNTIPCPKNYWKYAKSSSKQQKLILSKGKNPPRDFHQVEKRASIMIKPLPRYSRFWKISQILTLNASQGAILRAIHFKFLSVIVKTMNKISCNRHRYSLIPSLFMASQRLRMPKVKKMTHALLAGELPETLVQCLVDHGLNCPKYMNLSFQTLTAWAKHLDKRTEIKRPSTLPSPSSDVPGKTTSVPLSVEVKIKKEPIESSETKTVKVQKSLTESKSRTSTSHGDEQDQATLRTSTQLLCETGGMAGSQNIEENKDKSDTESEIVVSEELQSAVRAQDNQNSGEKDELCPPDENVSNLAEKKDEYHSKAEKEPEAIEIADVEMEETEEVKTKEEMETKPAEETERRVRRYKGSVGKRGKAQSKKQVKKEQTFEKLDGGLVSFLWREASLDQVWTEGYFIPTTQYGQSCPELCYLCGSAGLAGFIYCAACAEPFHKNCSLINFNNNLRYCVDIEDNEVISADWKCERCIMCHMCSKTTEELLLKCCECYKTYHGSCLSRDVSKAPSKNGIWKCPVCLFCRKCGTKGNSETKWLYDFSVCKPCGKLYSKGNYCPLCEAVYEDEDYDTPMVSCMSCEHWVHIECLAAMEGVVDQSKIRLQKYTTVRSIRTFENDVFQYIASTPGAVGTFRTVLNECFPWLAKVKKGIFDIKTPVKTPKKIHWFNKTSTQNKVPQKYESSTFVEKDCRTCVLCNQKGDGPEKGSGRLLVCGVDNWIHVNCALWSSEVFEESDGKLMNVEMAVNRGSSLRCDHCRIYNATVGCCDKQCPRSFHFTCGIKSGAVFLSDKRLYCKQHKEKHKKPHWQHMSQHDFTISRRIYIDPSKIGNTKRTFKQHGEVQFELTLTIQKPQLNTFDSDSFTGDKARIATPNKVKPVKTPVKLTAKIRQPREEPVMNLDGVKVKKKRGRKARENQEAVLDLPKANKSGDKSRVKRVHKKAELEKMLVVHRVSSANIAARQEQRNSELQRARKAALELQKQRIRQEQMFKSRRYAKLDHTYCMPYKNM

>ML033625a

MKQDDGSVKRNPLKNSQKFGTNKLDSSGTGLATAVLMKKRGEEFLPGDLVFAQFTGYSWPALVTQVGAKNLEVVTLVLEQDEMINKDSAVIFHGMDQLKQIMSVLTPGEQQDFSEASNSLLLLSLKTRQERLDIIKMGRSDFDEGESSKENKIFMSENESPVSECTKSPPKLQMSPKMESSMDSNGVKEIKKLSCDVDDISLPIVGSRLEKIIDQPRPRLTLEVGVPNFGPLDPLPVNSEGESEIEVKLNKSSESLLTPEKEASLSKRKKLQVKQQRDEDFIANAVKLTNKLLQTGGPSVELVPGHHLSYDSLKIARERAMAIAGCASPIISRTRRQSANKSTEASLTSPEDRAAKAHAEHLKATRAAQKKALKAIGKPLEIVQTTVKNPSKKPGRKSSSNVNSSTTKVAAEIAFIEAAEKLVANADIITSNATEPERALAAAAKESAQELVSINQQVNVSPLKSPARKRRLSKRQGSTENKVCEIIVNSKSVEEIQKTLSPQKIVSPSALQLSELQRSEKIKAVRCESLKRAREIKMQMIAEKKLAEQASPKAKSNRKSSKSLSVTSPISPKLIDEPKKHEKARIGGSTRRSRRCKSDIVEKSLTSAPEKEQIKSPSKNSVQTPSELTSEMGAVMEQEKVDDEFLSKPITRRKSRRAEASEQELTHAKNEKPSRSKCCRTPSENIAKTEELHSVNDEIPASTDKEKHSNSMEDSDKPTAIKSNPVDKPSSSKSEIINFLCPYYLNLFTNVIKEKIESQIKLDLEKLPQEERTLISNLVEGTNRSTRKQAYEIISKLVKNSPCQNLLTSNLPEVGPGTPPASPKTEVQRISNKVIPQTRKRNSNSNCHEVVHNPFPVETMELTEKARKKYVESGDLPKKHSVIPRTWSQYLKNNGRFATEQSKPVSVADKLKSPPSKSPSSALKPQNKRKKTDSPQSIKKEKNIELPTDFKMMALLFYVKDIQINLPEPFKLKQSEVKWTDLSSESQNKYIELCERLNVLSTDEKNLECTALDLFVRSASNISRLDWLSLSPTKHKKFLREAKKMTKKTAASLAETNEFSEKLVNKLINSESRKRKRESQPVDTDSEPSSKKEKSDDTLLDFFCMDCFTLDYNPKQYATCHGCHASFHRTCLESQHNSTKDKQDNKQRFYCRECQEGPVCLLCRKGNNLKSCSQCPYKYHESCVTPHMSTQVVSTTSFICPYHVCGSCEARPELNIKNFVRCVRCPQSFHHNHIPAGCMRISRSHILCFRHYQGFGKKLNPTVDAEQKLKRKRKSGSSLAQKKGKQNRKLAHLNVPFCFQCTFGGELICCEGCPASFHNECVPEENQIKEGEMSWLCPDCIVGKKPFIGDIVWVKFGAYRWWPCQVLDELDLPDNIFSRPHKPCQFPAICLGTKEFAWLDMSRVYSYEDGDKGSSSSNGMSASFKLAIIEAKDLHAAVKPMKEALLQETESLDRPSTKKPAPFKFIRMNTPYNSAMKEYEKQLKLERMPFPCDCTMADKCSAESSCINRVLLTECPTGCRWGDECENQRFQRRQYPSTKLVKTEGRGWGLVVNEDVTQGTLVHEYVGEIIDEQEVQNRLQNYQASGITDYYMLTVENSRIIDAKPKANNARFMNHSCDPNCETQKWNVLGLTCIGLFAKCDIKAGEELTFDYQFDTRGQEKKACLCGSANCSGFLGVPAKKIIEPTNKETKKKPKKKRRKKPAVAAKSSSSLHSEKSSTEDKSGETIAEPEHSTVKSKAPMKGGKSAKKTVGLPVRSNRKVPVKGVKSSTKSVLKNTILSKRRTKAEENDIEDDLQKINAIVRDVNDGESGSSQPEK

>ML03472a

MQVDLMKCVSDNVAEQFSSFKIDIMSRMDTIIEEKFSKIAASQHTTDPFNAINQDKVSDKSYSSLFLDSSKNPKSANDESVSQTMQVPLTPNSSIVPEFLVLSPNKNVSSSVNIEKMGCVKKLVEKKLKNCQVVSINCNEKSKMVSIGFPNCDVRDKAAALINFDKSLDPLGYQLQNASQGISTVAPVPPSVKQTMSVSFTFPMRYYTWVPVTQNVFVEDETVLHNIPYMGEEVLQQDTSFIEELILNYDGKIHDRCGFTDTCFDEVLEELVTNVLKFDGTLTIVDAIHIISPLYPQIGKKLSVFITPCAGGRTEVALSEVACRRDYLRHFIATSQTHFFDFKQIFDTGW

>ML056953a

MVMSSLSEQLWNLNSLDSLKNDAMKSADVEHAFLALSSNVQTAQLPEAEGKNWKLIIDPVIKGKGHTKVIRYGGTLDGPDEKPAVVKDPRFGSIMRQKEIDLKMPKFKYDKWSVGSPPMKSIFIYGLNDNVNQEFLTGLFQPLGSIDQLKVYYHPKTRKHLGIAFVSFSKSSVTKEVLAKFHGTSIMGNTVNIKPDPLCQITKKVLDKLIKAPGLVINIPEIIQSVELRSQHKTAAYAFRHNERSQNYYTARGKVRKPSDLSDQTKPDLLRRDSLTQEEPESHDSVPVQHNLKLEQVSPKIDTPPDTEISPIHPEEDIMAAQPPESLPSKPVKRERSLDRDSTVMTPELKYKVAKTEMMSPSDRHDERRRDARLYEMDRDRRKAHSERKWEEYKMRTEMQIRAEKTSRYVKEHCSGSSSRTGDKYVEEKYLADKYAEEKYGSEKYSKDKYSKDYHHMSERERYYAYYDKYYRDKDRASDHRYMDDKHRSKYDKYDKYAMDKYKYYDYHRHSDHKPRSSSKELEKYHKLSKSVKDYENVYHRESPRVDHVVALQELDKHRHLATSDPERSKSRSRPREEPHLVDRISKRKRSKSPIYHHSPISVPEPKKASPKIKMENSGSATSHDTGYHSISPLPISPPAPKPEIPQDISQLSENPYGCGRAVPRKNISKVKVIGMRSAGPSDDMSSSSMSLDEESGGEEEESPAVKEEMTLEDISPNTTPQQPAEQNTLPLQPPIPLLPVPTAALLPLPGVPPVQYQTGQWLHTTFPDMYQYGYESYNPEEAADILARREAEEKAAAKRIEQIRNRERKSKSLDMLLSLMESILKKDVKIILNEKTAFTKLEEVWNQEEDKKKKEEEELAAKRKAEMANNTDPLSSQAPDMPSLSEMRSMSDAKQSSHIRQANLSFKVPKLNRGMRPQVIVKKMQLPKKLDSAEEMSDDDVNDLDLVIKRRQVGNRYNKIYSSSSSSSESEEEEEEEEVEEVAEKKVEVELEPKDEVKLPSVAPPKYKSRVEEEEILVLEQIFREELTEEDKTFFKTAYDQILGDYPDLLDGITWFSAPEIPSSARRRQPSSVDMRHSTGCARTEGFYRMTKQEKMALKSSFNRTLSEPVTPGVQPPQPPAKKVDGRTTARSTRAMQRRQATHYLTVPEIQFMQLTTRRKQVKFMRSDIHDWGLFAQEPIIADEMVIEYVGEVVREIIANIRERGYEKEGIGSSYLFRIDQSSYIVDATHKGSISRFINHNCDPNCYARVISVGTKKKIVIYSKRDIDTNEEITYDYKFAIEEEANKIPCLCKSPLCRGFLN

>ML05765a

MQSITKFFSPKKNENESDFKAVKSNRKTNKSSKSSLLKIVDNEVEDITGLLFGTLNEELNQTKDIDEAETSEGSKKCTENSASCFDESFQIATENKVPENGASPVANRSRRFSFNIKSPVRTPQKKTTSLSRSKKDPQDTSFDEALSITVNNSWSNPEQDVDVELAHPKKRLKTETTASDVESIGSPSMDNTPRLKLVAKKLTIPCLQPPSAFSNPPKNKEFYNLRHSISNSRQKNYVVMKRACNKQNQAVYFEIEKILTHKISQSEKQYKFLVKWADFSDKYTSWEPEGHFDCSPHLLLDYINKLLHTVDKKSEISEELKVIKSRLKKRLQKIAAVKSQVHLWQLNAELSTQALKIQYKRLLKQYANKVQEELGPSFPTVEIHNTVDFNLPPLNFTFVRDRFAGKGVHISDDTLIGCVCSGEYGVKDCCLSPDVECCPHLGGAALPYNNKGKLVLKQGFPIYECNARCSCGPDCGNRIVQRGTQVKTAIYKTPDKGWGLKTLQNIPKGTFVVEYVGEVITFDEAEQRGKEYDAQGLTYLFDLDYQTTEGMEVDEGMFTIDAAFYGNTSHFINHSCDPNLAVFSCWISNLDLRLPHICLFAVKSIAAGEELTFNYQIDRIVEEDNRAAQRLSVECRCGTKKCVRYLHGMRQTTKTLPPFGWHSSTVHLLYFLYRKVLLCPKVCFVFSMANCTVCGKQTTKKCGKCSNAWYCRTECQRSDWKNHKKVCKYPFEIKESEGKGLGLFATRDLQIGDLVLCEDPILYFECGRTEVVLISHKKFKEAYGKLGREKRYQILVLAGANLEAAYSDVGLNLTRSGSSSEDEEWRQAMGAFSRNRISVNYEGKKVGSGLYITISRMNHSCSPNTECSYLKDKRKSKEVRAIRKINNGEELLGDYMTDETSFLTTSQRREQLMQTWKFHCECSLCSSDYEANDKMRQRVKKLYDVIPIFQAREDVEGAANAALERCKIIEKCEDMNRYLPGAYLELYNILVMAAMYKGDFGFNDALMANLVKQREEYREKAYYLNKRTKLTTRKDQHNKMIRQLAMMGCLGPGGSNKLMQ

>ML06271a

MSELPTNVVTPNISNNQPIKKPTAAVSPVVSFTQEKSDANSTVSVNEVAPNPPPAVATAAVSHPNQQQPIQQTPQFNINISLNTGQQTQQPSSSQVSQPQNPLPDNRRPMTGGYFKFPVPFRHQRMCTSCLKHKPPVIDVEQGSKDAPITLDSDSDEEKHVANTTCTSPHTSPLKSPPKALSTSGVFPLPKEPRPDPPKQVEEVESSSTHDESAKRRKLDSNSKQFNNQTLYYAALKGDTSKVSFLLEGNPDRMNFRVKDTGMTPLHAAVKSGSVATLKELTRHIGAGTLTLEAMDKELQTPLFYAIKHNKAQCLRFLLTQGVNVNARDTNARTALHYACELGNLLCIDEQNIALHWAAFSGNSDIVQLLLSVRSDIDAVNERGDTALHISARLSHSGSVRSLVNSGASLRLPNKQGKTCLQDAASPEISQLIQKLEKERDSRNFTSPKPQKNTVLHSDITCGKEKIPIPCINEVDDVAAPLDFVYVKESFESSYLCINRNMNVVKSCVCENHCRPGAPCSCFFLTTGGKCVYNKDGRLNTEISEQYNDIVIFECNQRCRCWVNQCRNRVIQKGLSNPLQLFRTNGKGWGVRSPKRLVKGSFICEYIGEIISDSDADRRLNDTYLFDLTVVSINSINNDNKTIGSIDACHYGNISRFINSSCEPNCVPVKVFVDHHDLRFPRIAFFTTKDVEPYEELTFYYGDSFWDIKKEVLKCCCGAPNCISLLSQKDASGEHESENSDIEIVE

>ML064936a

MVQYRKEYIDKSTGSGLFATSKIKCSSQVIKTNFLVTSLDQKFSKVLCNQCFKESESLKRCSKCKHMRYCSENCQREGWKIHKSECAGLCKVPPSSHIPSLIRLCALFLSSQELKNEYSIPSLMKHENIENEKIQEYYPYVLRGVTEIFKGSSSVDPVDVYEMYNKLVVNLFTLEDGQLRTIGSALIPEISKINHSCEPNCILSFKGRTAYITAIREIPKAIQFVLSDEFSHMRPEQRQQLLHEGSGPRVVNAYVEITFDNTDNRIPIEKEEVTIRRVIGFKKDQYFLDKKNVTKTDVMNLLESAGFSRSNPYYIVKQGRINQMAVAPDSQRLKLLREVAGTRVYDERKEESLGILKDTEGKIEKIDEFLQYIEERLKTLEEEKEELKEYQKWDRMHRSLEYTMYDKELREVRDKLEALDQQRLEEGDRTKDFTIHLSTTANEIKFLERDLKELEIKTSSTRTERDQLSEDRQLQIRQKAKLELDLKDLDEICNDDLATKGTSAEELAQVEEQIKTAQEALNQVIPQFEEQKELEQSCNSQLRMNEQRRTELYAKKGENLEFTDQKKRDEWIRSELKTYGSNLSDKEEQLKTVEKEVSDTKEKIEKITEEIAERSENLDERKKEIEETNKELNEAKIKRDEVQNKRKDLWRQDSNFEQSLNTSKEQLDEAERQLRSTVSKAFNNGIDAIKKIVREKNISGVYGPLIENFKCNEKFFTAVEVTAGNRLFYIIVDTDKTASQILSIMNKNKMSGEVNFLPLNRLSFKEIQYPTSNDVIPMVSKLEYNQIFKPAMETIFGKTLICRSLEIASQYARNEHMDCITLDGDQVSRRGALTGGYYDKRKSRLDSQRQVWYWQAKLQEEESNAGKIKGDMEEVDSELSKLVSAVQKLETKQIQLRETYERQKMDVQNLSVEKKQLEKHLDTKTRLKMMLEGDLTALRGTVASLQQELGTELLSQLTEDEQEEVKKLNREISSLQSQIRESLERRSKLEVEKNMYENLLHNNLFKRKDQLLSNIDEFGMTERKQEYDRKKAEFAQATEGMEATKKRLEWLENEMDVYTKKCKELATQIEHKRGKEREIQESIEEESKNMEKMANKRAVKLRKKEDVMRKIRELGSLPADAFEKYQNMSLKELWKLMNKCNTELKKYAHVNKKALDQFVSFSEQKEKLIERKKELDKGLDAIKNLMDVLEHRKHEAIQLTFKQVSHFFTDIFKKLVPGGTATLVMKRSEFDEEGSSVPLVEQFTGVGIKVSFSGKVSETKEMQQLSGGQKSLVALTLIFAIQKCDPAPFYLFDEIDQALDPQHRKAVAELISKQSDTCQFITTTFRPELVQAANKHYGVIYRSKVSHVQQVTMEEALDFIEDEAPDK

>ML08863a

MITTKPDYVYLPASLSGDQAQLDCVPCNCTGFCDSTCGHSIDIFDTDPIYICNIYCPCPASCPNRLSTAGLDSVTKYDEKKGWFLQSLSDISASTYIGDYTGFIIGAEEVRARHISSKVNYILSIREESDKGTVVTHIDATTHGSELRFMNHSCSPNVKVVPLRDNTVYPRATCWTMTHVTRGEELCISYGDGEHLGSVICHCASLQCLGYLPFQPIAETL

>ML09663a

MEGLDSYSPSPENDENSDLKLNRDGPVEDGPDSPKRSPSGEEEDDYQDEPVHYDDVASPEEQLESRPASSDEEEEHFREARNEHEFSNDGAAANGGEGTRDGPEVRSESEEEKEIGHEVEEEEEEKEVVGEGDDSMMSSTAVSDEGGGEEGGELEQGGEDENTGSEKQGEPLEESRADNTQSEEQNPGQAESGGESEVDMDTAIDHEEKDQPEHVEEVLEKKEEEKDNDDMEHGYHGDETMRSPEGAVNETMTSYSPQPCTPPHTDHTDVKSPDAEDVTQAPASPKMTIERPETPTELPMSPAERPLTPEPTEHLISPAECSNSPSEQERPKTPSERPTTPTDGPRTPTKLSVSPAHRPGTPADPPGTPTDTPGTPTDGPHTPTGPEPPDQPFSPRTPSPVTSPVKQMSHNPSSNTSADQSFESVTSEDSVPPFEMITENEYLSASKQRSRKDKKMVCSCEFSAEDPATHCSDNCLNRVLMIECNSRCPCGDLCKNKKFQKADYAKCEPFYTGVKGWGLKAAQDIIPGDFIMEYCGEVVNQNDFTKRMEAYGKKRNRHYYFMTISKDEIIDAQIKGNLSRFINHSCEPNCETQKWRVNGVLRIGFFALKSIKKGTELSFDYQYQRYGEDVQKCYCGSTKCRGSLGEKVSDLAPKPQESPLRDSTSSPRPKKKDSTGSLEQSLSRITRDDGGLSSPDNALQLSRLMVQTDSVEHRQLILKTIQSTQDAATLKRFLNYQGLNILWSWMVDVNEYPAPLRGECKLDIIRALQGLPISTRNAVEDSKVIKVLKKWTDNEQSSNSSQADSSEESAPNTEETGEKSPEEVESERQVVESGQALLDQWNSLKEVFKIPKKDKKEAKPSYSSTRSGPATFSLGAFLASVGEEVSAPPSNIPPPARTMSLDQADYELPMSFGSTKREYSSDSTRDSKRRRDDRQSKWSQPHTTPQSQPPSLPQTQPQPQPQTQSEMKHQGYMVHESRLNKPRGESRWDSNEDSKTKDRGKSEDSKSRDESRRESSRRDDRSRDDKRAGSRRDDRRDDRRDDRRDKRDYNRRDDRRDDRGRYNDRRDNKKDYDTSHDYSSRYSSRGSRDTSRSRDDDYKSKERSSSRERKGSKERRDKRDKSENNSDQDSKLPQDNKMDIDEKVSLPPAAVTQKPSTPAKIENLPDLKKPEIPSFTPPTSLAPPGGMPLLNNPPHRMPGYPPFNPRNAGSFPRFAGSMPMFKPRTDSPEPPRHDKPEQPRPHLMSTMSDMGLQHRPLMRMRYRPPTDMSRMQAPQYDYRGQQAMRSRFDQQWNNPALRFQHRFSSHQPELPPNSLSENQIPQHPPGARMPAVPPTSRMPPVSAGGRMPAHPGASSMPGVPQAQPLMQQHRMSQPGLGMQQYGLPGPGHRFPGVHAMPQDLFKQMTEQSLASGLVTPIAFPQQIQGIFQTPGAGTVPVNPPTTSIAPVLPANISHSQGPITTQPALPKEPEVEKKPQVKPAPTPVVKPEERKLPPHWRTAKDGEGKIYYYHAITRETQWDPPVLEDPTKKLETPASPVLLTKRQPVVKKVKREESPVKTKQASPVKKAEPQVDPVTKKALAEFKGQLAKHVVNVLGKYNKVDCKVGRIMDNNDFKYIARKLTHGLTEKELQRKGVGELAITDSLKQRVKQYITAYMSKFGPIYHRT

>ML09991a

GNFDNSMRKSSRFWVDKALISGKNFKSCSNYRPICPNLFCCCQVMNSKFTRFYVLTRFQAGQQPLQISNELKQSHGRSAPSQKTIYRWIDSIKSGTFQLEKNVSPGRPIDPVTKEMIASIKVMIRRDPRLSCRALALETSLSKSSVYRVLSDHLGLRNVNSVWVPYNLSNQNKADRVTCAKEILKLFATHPLEVLQSRYCVQDETWVTWAAQPSRRVWIAKKVKKPTTVKEKLTNRKNLVLVAYTCKPKRFSVKVLPRGQTIDAPVMTEYLTETNRRFRRRKNSSIQFSELIWQMDNARPHTAMMTQRYLTSTGVSMVKQSPYSPDLNLCDRYLFRILKNEVKKSSSSYDSAEEVETDVTRCFRHLSEKALCGQLVKLREHCKLVVESLGDYISTI

>ML124240a

MSQVIKCTRLLPNACRLLPRHSSTSTHVNKSELAATTEKAEQWNSGNSKYLFGAAAALGTFGFFGLTAFAKDIEEEDEDEDEDEDEDEDEEEMSAVLEAALAEDVELPADGNSQYDKWAATQQRYLLLSEAKRAQIELRDITAEELAECDELYDQDRMTDCEDKLIELNTVSPQNDEVLWRLARVKWVYGRGYQTNHQHKIFHVAHSMAWEAAQINPNNGNAHRWLAILGHYRAQKEGVKACVLNMEAMKYHLDRAIECNPDDNVAWTLLGQWYFELADMTRLQRRWSRTFAKVDPPAGSYKKALACFNKSDSIEPWIQNTMHIAKTQLRLRNKEEALEMFRRLRDDTVIATVEDYRMRKEARIYIHKLTDPTLFNKLMNEPLDCYQSSEWKEHTAAYTQYEKLLKQYEDGKIELEMSVGDDPAGGTSKLKNNRDKDSPVVNRNREPPKKNNKKTQKSKGKKSESSSEIKVEQIDRSEIVGVKTSFQTENTETEGPEVDDLEMFLQSIPGSVEALQDLLVHKNLASHVTDGPVDIVKLIAENPKFTHEILEANIDSILPDEDFLGYLKLEYPGGLKEFLEKNKDQALPDVRIEPPEGLFDSDDFNEETREDEEEEQYKFGRIWRDPTDKCYTRVKKLYDNFHHMTETEYCEKVRLELQAMNTIEVIDDEEKNTERTYLNYTEHSRAAVSVPDINTPSCSDVNFQTPSCFQITSSSTSPDKSGSDVLTSTSVTTHNAECTGSDSDEITSTSVTTHNAECTGSDSDVLTSTSVTTHNAECTGSDSDVITSTSVTTHNAECTECIDSDSDAITSTSVTTHNAECIGSDSDVLTSTSVTTHNAECTGSGVTDTEKLPTVLSNRELYDKFDKIPRPSYWLPETVPEKEKEQEIDDFEYLGNLDDGYLLSLFEEEEEEKIPSCADVETLSDVNNTLGENIAKRDAEIDQNAEGDLEKDAKKDAYEDAETESIYKEVEATALTVDNTVPSTSKVNSRRYNPPRRAKDGVEYCEDTDYLEEVLQSEYFCWQCKDNVRDVCALHGPLLPCTPNISVLPSKNKTKFSVPSFIEIKESTIPGAGQGAFTKQFIEPGRILGDYAGKMITEKEYKKLEREQKESGYAWKAVSQDGGVIYLDGADKRHSNWLRFVNCARGRCEENVLVRNIPGGVQYYTYKPIAIGTELLVFYGDGYFEELGYSLSTDSESTTHTGDKMYKCTQCDYSAAEKKSLTIHIYKHHTNKTYRCRYKKCGVKKPSVQELHEHIRTEHPVQQHRCDVCPMSFKGARNLTLHKLTHGERDSRYECKYCGKKFVRLADLKTHSVTHTVPAIESSSPNITLSDSQSIYLSFHRRMRPRHADARSCAGQASSISCIFISAVYYTV

>ML12425a

MFEEDKKEELGYELRSTPGKGVGVYATRNFPRNSVVVIGNPVKTAEKNTAWTTQVGVDRFILRGGLSSKVNHSCSPNVGYRDNANGCMDYVAFRDIRSGEEIVTDYAMGNHVIEHMPECLCRASRCRKTITGWKDLPDEVKREYAGFHAEYLTVMDANMDERLN

>ML13116a

MANCTVCGKQTTKQCGKCSNAWYCRTECQRSDWKNHKKACKYPFEIKESEGKGLGLFATRDLQIGDLVLCEDPIIYLSDGIKGVLKSPQTFKHTYENLGVEERNKILALVGADNDLTACNLVMESCLDSSSRKEDQEWKRALRIYGRNAIAVDDSGSALFSTISRINHSCSPNVDWSYLENHRTRKEVRAIRNIKVGEEVLVDYIPKSESFPVTSDRKEALRWGWHFECVCSLCSLDHDENDALRKIVQQLHDSVLNCGRREDVEGAANAALQKCKLLEKCEDLKIHLPVAYMELYETLIMLNLYRRAYGINDALMAKVADRREEYREKAYKHCRRTKLYIDKAMYNKKIKRLAMMGGSHILLR

>ML13535a

MNESESDVIVAHVSRSADIAFGILELISSLGGLFLNVCTVKFFFKKKSSPTYFLYLCIDRPSQHPPRFKHPEPSHLLPESSGRMLLGYRRPEFRTLRRRQGCLVQVPGLLLCHSPLLPSHLPSRRLLHHIRQDRPDEQTESEELPEQRPVQQQPHKLSISQPRAKSEDPGGGETCGHFLRAPLRSVPDECELQLSGGPTTTPAMCEEGDQWRGRSPTSHHNHVYRYSSVHHLQRALLELHSGCHVLPGRPHQVGHHQRDLHQHIPVPHKRGNERCLQSHRLLH

>ML16213a

MAICTVCSRLTSKKCAKCGLVWYCGTECQKSDRRNHKLICKYPIEIKESEGKGLGLFATRDLEIGDLIVCENPILHLKSGNERILQCPQEFKLIYEKLNREEKDQVLALTGIERESETSPMERFMDSFSGNEEWGKALRVYVRNSISVGISKEEGTALYLTISRINHDCSPNAEWSFLEQERTSKEVRAIRRIRKGEEVLASYINHYDQFPTTLRRRLVLEKEWNFRCSCSLCSSDHEENDFLRTRVQELHDEIPRLIQQGNVPDAARAAHQKCQILEKCEDMAGFLAVAYLELYELLIGVAKNRMIIRNLDPQIANLSDTREEYREKAYELCKNSRLIQRKEMYNKKIRKFATCGGADK

LI

>ML167015a

MPQKKRNKAAPAYRLQDTPDKGKVVVAGRNIKQGTVIFEESPILYTSDITDEDILEQFNKLNLKDKDKVLSLSTIPEEDPSKKVLSLFRRRNINKSKCKGGQALYFVLSRVKHSCGANIILSPVGGKSVGKIEIRACRDIKKGDELRADYLPGINLFEPKRERTKVFGERFSCDCPVCLLPEKTESETNETIRKNIKEHYRNTSIYMTLGDFLQALDHCKAMLAEMELLGDEVFPKRVSVSLECWQLAVLANLQRPEVDIDVEMFREKARQLCCVLGKAQLSKWERTDEIVQKGNFVEFDTVKLRSSGPWAVQIIENLTQAKV

>ML19101a

MSTSTSRVRKHRENLTLEQLELRRKKDRESKRRKRALMKEIRQVIPPDPNIAAPTSPALRRSHRDRASASAKASKIKSSVVNPCYECFDVRMSRGKGRGVYTLRSFRKDEFLLPYVGELISKREGVERERKMRESKGCYLYFFTHDGKSLCIDATAEDGSYGRLLNHSKSDQNCLPKRILINDRPYICFFAARNINPGEELLYDYGENRADVLNELPFLKS

>ML205611a

MFMLAVTVLAALALSSQSAAVLENSLLAGETADTEGSFFGLSVPMNCCETCGNETSKRCSRCYKVRYCRRDCQRKDWARHKSNCGPFEVRESPGKGLGLYAIRDLEIGDLIVQENPILYIEAGGIFRLSRNPEILGEAFQGLDESDRNNIMSLTAARSNNLIRLCGVKVPNDEGFDWETVFKIFCNNCIVANDYRDSALYGTISRINHSCSPNVEWSYVKKSSRSKEVRAFRKIKCGEEILADYIAESGKFAVAKVREALLRLKFGFICRCSLCSSDCTQNDAMRQRLQLLSRIIQKNCRVNPKYAVAAAYEKCQTLQTKFCEDLIHELPMAYLELYETLVWYKIKAPGSKARVSIDGLEKDHEFYREASWKLIQHTQLAHSKVAFLDRIGYLTQVQNQVASGRVQIPHPF

>ML221321a

MCNILILQIMTWSKKDEKDESNSDSDYELASESINHDHIDQVISETIGFEPIKCEDSDNIQSTSKCQEQSEIAESDSEETKYSEEELISIREKALDLKAKGNELYKSEDYQGAITVYSEAIQLCQKQLNDLRSILHANRAACHFSEANYQETEDDCCIAIDLNPKYLKAILRRAQAREKLEKLSPALEDYKTVLELDHHQRVALEATQRLPKQIEIQQEKMKEECISKLKDLGNLVLKPFGLSTDNFNMVQDPNSGGYNIQFSQNK

>ML22756a

MAKCTVCGEATSNKCGKCGQVWYCKRECQVTDWNEHKKVCKYPESYEVKQSEGKGFGLFAKRDLNIGDLIIREDPIIRVTSDNRSAGIAFERLAKFEDAFRKLNRATKLKLLALAGDRFGTMIEIENEEGDEFDRGSRDSALYEIISRINHSCRPNAMYSFMMSNREAKAEVRALTKIAKGQEICITYIPATDDERVFRISKERRQAALMSLRNFVCQCELCLHGSLEEEEMRKRIEELNDEVAKIIPNGKFEKAAGLILTRIDLMERCPGLVQEVTIAYRDLYELLLEARRRSIHINYKRIKLSSNIDVYREKIVESAKPFKLDIMKVIYNEIMQKFSTKYGVEPNLWI

>ML257621a

MDQNKECGFCTKSGVDLKKCTACKKIWYCNIQCQRADWQHHKENCAIVRVYPKILIPHKAVNFVTGEVITHNFEPDQYQGKTEDMIVKIQTTFARAMNSVGTEFLTRWMLVYDKSRKYQVKIVGAEGYDENYDAISDKILVDGLPCVQKHPLLKKLYFKARLYPDSSLDVYLDCTYNDQNW

>ML26851a

DISMGEMIIEYSGTLIRPGLCDIREKYYDSKGIGSYMFRIDRHEVVDATMSGSMARFINHSCDPNCYSRIINVEGKKKIIIFAERNIVRGEELTYDYKFPSEDVKIPCLCGTEKCRKWMN

>ML368810a

MLKNALHVKMARSKKKGRHLVATSDLTPGTVIIQEEAFAAVSNSPFYCSNCFCRLLSAALDRPYVCRDCRNESLTGFSRNDKSLVILCYKIVLSMLLQDQFSFEAFFREKSNSKSSEDDLYCNDFRAVAKLVTVDLDVSDSDIKTVCRMVQKKTSGHSKDQITSLVRHVFSTLPCNVQGIRCITETDGNFSDERIGSGLYLSLALMNHSCSPNTRIYFEKSTVVVVTTTKVKEGEEIYHNYGPNVLHFPKNERQQILARQYQFTCDCTACSVAGEEEPQYALRCKHCSASIWLQSDVPKCCSCRRFVDFAKISRRLYFLHQKLADVQDNPDSDVDFVNGIVDEAMQLYSSENVTFGNIMDTCASFYASKKVWEKAISCAKLSCSVVEKAIGTLNQTYAIELLKFCEIRMNSLEHDTLDEHLLEDIRFCVEILQQYKSHGLDRLHRVMSWSKLKA

>ML41301a

EPRKILEYEKHIEPIKVRPGYYIDCISANEGIKSLLQRVGGDDSLRCCGIPYINPKVVFMYEDENGKRCIKMAPPVTRRKSPDHEFEMENPSRGTTKLKSVLYKYNKKKKVPSSAGYLHRKVHRIDMKPAHQVSSLYSSNQFKNRVVQQIKAYHVSSNDPNSESPRKVIIPYTSDFPVFAASRTGDSRSRPAHSPHSTVSPTKQFQTRSPESGMRREDTTSPVMDIPSHSEDPQINLFSKDLLADVTKLHDDVIKSHNDLKQSSQITKDTDSNQPLLTTSKNELQPIEKSLVSVENGVNPCLGHDNIRSNSVPLTSSPTEVTSSTTTSPSPNFPPTSLRFTRKLTNTELPGARNISRKSNIPENSVFGKSNIPENVDFGKSNIPENYVFGKSNILDIPVGDIQHNPDYQYWELLINSQYSRTRGQSSSSSIMITNATPSHPTFSHHNPPLPVRNSSGKVMCVGSRDISPRTSEYSSTIVTGPVISSPINKKPRSGFDVFDFDSERTDDMYRMKCTAEHYTCIKREKKSLKKKTSEVERSTTSSSSLKLSSSSKPSWVVVEENKGRNYEKIDVDSWLTERTQAQKTQKLIEEKYSFDATIENEKLPHRVTEQPGFSYLGGSTRKSTKGSLSVEPEIQSPHGYSKPVQSFPQPVPRVSHSDQQMQRRPSVEFEHQPQRRHSSEMLPVQKKNSRDFLLSEFDAALKKFERTKVPDSLPLKDEGDDAKDKQDPRPMVISPTLLKSNKILEKTVQKAAPTKPLIISRALTSNTKEVKAPLPSSSEKTKAPFPWLGSKEPRSLFPQTKRVKHVDKVSLLGGGGNKPVVSGGQLNRSTPKTSNTTSKVDHSGGNQKSVVVHSGVTEQPGKEVFSPEMVANKKASEGKKTYRMSASLKELQPKVLRNAPPKPPSPVKEVLKDFIKEREIKKTLNFDVAAWKTKVGDATRAPQRSDPQQDNQNKKIIDSFSSYDPPRIPVQKPLSFTESLKSSTVDNYDITQPPSQGEGTSHSLLAELNSTVSPEPVPKSSLSIVQTGYQASPGKVSLEETASILSTSMELCQTYPDPAATRVSLMTSPGLDRVTTSNSGQVSQLNQRHTVTSPGYPQSTTPASASTPGRDPEVKMEAEETMSYRTHLPTVSQSSFLDSHTDIPKKCQNKHSLPANLPLKTEVQSQTESSKQTLTPTVPPRFCVSLPDKNVCSRHYLTEFLNFQFSRLKVKTEGVEQTTNKTVKMEVGEADEKKESPSTCRSKRKSAIIASQNLVEKVYKDEELDDEQVERKNKRKCDRPTIVQSPAKSLSSRHSPVKTPLRVKSVQSSAVKLKLQISQSNNRTYIRCHRKDRDPSEKTFPVFIVSTEDGVYVETDDADDAWTIISTLLQRIRNSKHQSVLHGLAMWTLAHDSVVHIIEQLPNTHRLEEYSFHFPEHGPQRAHMSRILPVPPGGCARTMPVVRKSYIDQFAFLNHPNRNIKPPSIDDGRLAVAMGDSVNLYNRRTTSLGDLPMAMRYRHIMRTVKEVLCVCPSIIHGRGLYCMQDISMGEMIIEYSGTLIRPGLCDIREKYYDSKGIGSYMFRIDRHEVVDATMSGSMARFINHSCDPNCYSRIINVEGKKKIIIFAERNIVRGEELTYDYKFPSEDVKIPCLCGTEKCRKWMN

>ML423310a

MVTYSDATYKMICSRIEKIEAIQQKIFKCLHESELQLRQIAQEYAIGYGEELHEVCTQLEQPTERVLAKHHGWQWLLGKTNTRKETLATEKRDLKISVQFDDILVEKCVSQNEIIYVHDYKRRDLEIGMKVLAEYNCYDAPGVFFTASVAEIPEQQNKYRYLLLFDDGTAGYVSEDKCFICPDQSFISSVNPTHRRFIQYYNKMYQEGGFKILDLSLQRTMLTEINGEWRLCPINFTDCNIANIQVSADQAEFVYCGSPRLAPIYHLLKTHLVA

***Monosiga brevicolis*  (Choanoflagellata)**

>jgi|Monbr1|10803|fgenesh1_pg.scaffold_24000017

MISDATSANFYAWVLRPLRSGFAVARNKFCLDSTLSGLTPSDPHSAAQIIEHPTFKRSLRLAVMQVEHPRQASMIVRRLHLQTLSGAPLVALLDYEDQLLDALSRRAVAECTHAVETRTQPGCLPDTLMPYLREFYPLGVRKPLRVTLHGDPPSAVVHFASAALAEAAYTQHRSPALAFLGDTAVARPASPPVNPRAVAEARAASFRRHLLAKVRKTLFSNVLRAVTRTLHREVVEPKIDDLIAMHVDWSRHRRESKRQRQEQADEAARSVEQKDLHSVGLLPAGYHPNPSPPRRRLVSSDKSDDESSEDEDAPQRRDYISSSSEDEQDMDVDEDDNDGDADSMPDNEDASEQLARLTQRRADAARRAQEAELESEADKAAREAEAAAQEALDKALDEPLRTPVSLDQPEALAQAQQEIETWLASATAEDVEFVIAALFAQTDRKAKDHAPAYATQYLPALLRALRAELASLDAESQLIATSVEHKPTAEPNELSEPPQVPKVIPHATGCARAEGYYFVSAETKRANVATGRQQVQKVARTATRDNRRQARHVTSALASTGNMNLAAEVGLIQQLKDIPANEVVIEYVGEVIRQSVADERERRYTQVKIGSSYLFRIDELNVIDATRRGHIARFMNHSCDVGLPRSIPNTPFHPKVFESHDEKHIVIYSKRDIREGQEITYDYQFPMEDEKIPCYCGAENCKGFLN*

>jgi|Monbr1|11753|fgenesh1_pg.scaffold_31000110

MVVVEVKAVPGKGQGLVVVGGRLGAGTAIRQALPVVAVVEDDERFRRCAGCGLSVDRALAYGHPGAQAAVEMTGDRPSWKRCSRCKNIAYCSPGCQKRDWKAHKRECASFNKLMPNTFGWCDTFDMSSFGAVVYAELSRANHSCQPNAAVVYNGAAAVLRSMRDIPEGEEVCISYVDPTLARDVRRRELVQSYGFACDCARCATEASQDPDAGVKDVAAWKQAQADIDAELSQGRVVKPLEVWATAKHLAPDSNTAKMAISRRLLDATLSLQMYREVGASDLAVHTSTCELLTLAQLQFELGQPEGPETLHHAKTMLELVFGMDHPIRAQINLGMPPL*

>jgi|Monbr1|14347|e_gw1.3.298.1

MADEAKLQKIELEDGSWMRGRLENGELQGRVEHFMADGTRRICTYVEGAMHGTQELLDGDGSLLSIMTFEHDQLQGRCELPLEDGGRIIAYIDASWLTCQIGAYIYPGQVCALIGRFHGGRLVCGHAGSVAEAEELINAAVVGRGHLPVYFGEAVSSTTIAERPLQPDPLEQAMVNVRPSLLPASGEGLFTSKPATAGTIVAFYSGVRVAQDEVDQRDWDETDYTLSVDDDCCVDVPPVYRSLGIYCATLGHKANHSFDQQNAQYEPCWHPRFGPIKCLRLLKDLAAGEEVYVHYDYEVGQILAEYGRAPPTTEPADAPQWFLEQLHRHLKATKVVGSATGPE*

>jgi|Monbr1|15842|e_gw1.5.440.1

MDAKGKGVLAGRAFRRGEYVCEYAGELIELEEAHRREERYRTEATAKGLDEMMCYMYFLRHKSRTYCVDATQTGRVGRLINHSRAKPNLKTKLFVLDDRPHLGFIVKRDIEKGEELLYDYGERDPATLRQMPWLKQ*

>jgi|Monbr1|16278|e_gw1.7.139.1

LAACLPKFAPGRVGLSVHPERTAEEEYDELVDWLKQCGATVDKVAVDHFNGMGQGLKATAEAAPGETLLRIPEACMLSEESARRSTLGAYMDSDTMLKLMPNVTLAFHLLLELHDLDSFWRPYIACLPVSYSVPLYWDLPDLMSLRGSSLFVEAIRLYKHVCRQYGYLHNKLSVRANPSCSCFPLTLGLSPEAFTFEDWRWAVATVMTRQNSIPQAGPDGQMKPTLALIPLWDMINHANHPMSTQFDSERECLEFVCPAPAKPGSQITMWYGDRNNGQFLLHQGFFFAGHANDYVNVPFSLDETDSLYKIKALLLRNLTVPPAGDFVLSTDDQLDPQLLAFARVASMSKGGPSRLEARCQSSAFLLTRTSSLRMPCLVVCMPGVLSVIWQ*

>jgi|Monbr1|26549|fgenesh2_pg.scaffold_14000215

MDQVVRGSEAGKGDDGNAVTQPMPTPAPYTSVDEVMLPARLQGRNNVYSGCSCTGPCAPETCSCLCNGRARYYECNDNCACDVATCRAGRTTQQPTALDIRLVWTSERGHGLLTGTRIPVGTYVGHYTGQLVNVATARARDSAADAVSPVHTYLLVLREHTQRGVLTTAVDAKEYGNLTRFINHSCAPNLELRPVRLGFVPRLAFFALTDIPAETELTFDYGGAPPSAKIATTLPADTDTGAPRHKRHVTAPELALSAKPCRCGAPTCRGFLPLTIW*

>jgi|Monbr1|26994|fgenesh2_pg.scaffold_16000182

MAAAEITEAAAARASEAGAGSDAVQDELQQLREQHKKAVHYTANDWIEYEQIEAPIYKAKMHKLEEDMECECQPNAADEDSYCGSNCLNRLLMVECNVARCPCGNKCRNRRLQKQQHARVEIFKTEKKGWGLRALEPIRKGDFIYEYCGEVFDQAVFRERQLEYAQEGRFHYYFMSLSADTVIDATRKGAVSRFINHSCDPNAETQKWTVGGVLRIGFFCIRDIAVNEEITFDYQYERYGRRDSGATEASSAKGRDRELNKLRDRLRPLLAPDGGLRNSFESTLSFMREMIHWRSDEVKGRDIFLDILTDTTDDDVLVGLMTKKILVVLHSWLSDMFDEHVNLQRKTLKILLRLPIKTKVAIRKVEPTLQCLTNSVDEVSQDLAKELLRAERSEVYIMNGQSAEQKNLREVVAVPTRHMTTFHSAIVRDIAYQHHVKVEQLPSTTVNGQDLIITGDDAMSVSSAAQQLRGLLEQAVRNLEELESQRRARAEIRNSPQAREAQSRLPGTPGASSLSMVAAASEPPKPSNLPVKFVDKHLPEFSSMSLHPDWDSVYSEQHNESYYFNRFTNETNWLRPVVQPLADRIQRRDNKRKLPGAAALGMDVDAKPTLSQVLAEGQQSKPKRVATEESKRKARLNKFKSEVSERVIKQLNAYREKKVKYGYIRTSEDFKYLARKLTHKIVEKELQRRGQDVNELVMTDGLKGKASTRMHIKNFITDTMKKCGKVFRQPAAPI*

>jgi|Monbr1|27206|fgenesh2_pg.scaffold_18000025

MAPNPKGNANTRTTSIRSARLAKKADDAKIIAEATAAILTAVAEAKTKQRPLAPPFERLPTRSQLPEYYQTIKDPIDIDTIRKKASRKEYPSVAALVEDFQRLFYNTQVFNSSRSLIYKDSRTLTNVVRRAAAAFVPEDELPQVVVFGGAAKPAPLPAPATPATRVPSATGPDATVSPTAAGGTASPTPAIDHVVVAALTELIPNINARVLVVDGNTGKPPLRASHNPCWRTLASYLNKHPSFRLAQEADQKIVDTIRADHGLDIKGAVDLLKAQLLPVSTAKTPPTQAPSATQPSKKVKNTPVRTPSLNQNKTAKSTKKTPKRAPATTPTVGSGSPLAAADAPTCPTSTPKEKLADLMAAADAYQASFPNNNSAAQETEAFNLTVRDLFARHVLSVRSTIRFRGVMGRLDAEGIFRLANGHPFPTPATMGLIMEDGGFASNRWEPVRLDQKTLAQLQASASRIAKAATLKAAKATAAARAHEDEAMDIDEQEDFLHLNLVDKWTSADQERYSHRLGLAVASDHECPLDLCFACGSNGHLIDQSRKGRKRSGEALKRASSSAPVTPVKSESGLDACPNAPLAGNGIPNASVEPLQATTEQSTTQPSKAQPEPSAHTAIEEQCQMAVSYSARLWQCKNCQTCDVCTRIEPTQHLLSCDVCGVHRHAACASAATPSYMLATQRWVCTDCVQCEHCGATDVRGHRPDPKLREEPTWQCDFRLCFDCGLNKLRGNFCPVCGKTYRGDDYDVKMVGCDRCDRWLHAECDDIDEARYHLLTFVPSSMSYFCPDCRRSDANASSVKELFEAAGSVMHERVGQALERATRSSSAWVLKVLPEGMTLDAENGKLPDFLPDGVSDFQSLYERVQSKGFASPQEFIRVFGDNDKRCRAITSFWGVLNTELGRSIDLSLDPLPERGQELSVLAAMAGTAKTISAAPLTTSPPASPRHSHQASDRTSPWPDSPVVTSTPTAARATQGLGPKDAPSSTPASPTTRENIDLQHQVKAASQRTIPIPSLQSPKLKDGQPSCPEQLQPASSSTSQTPLNSIPVPTLSGQFRSTLPIPSPPKPASETTLASDIPTAKPPSFLRRPSLSAIEEPLLDYVRFVNPALSVDMFRHVLALFHEYQTTCTPGHQGNATRVLQGFGLQPEHVQAGLEILARTWIHVNCLLWSPEVYEGPHGLCRARAATRRGRLIMYCDQHREYGCRQEKLQPSATALTPGKFRVERHIVVEHQSGSREGEGEQLEAVNRTLSLLPSPQQLALALLARFPPKRVVFPSELLQKLQATAQKSLRLPQICIGAFKLLWPGSMAEHCEGLYSDKLLLPNNYLACRRFWDINDVLNNRLNLTSYYMRVWACRRTDDPAPEDPDEAPMGAARCEAHDLQAARKLARSSLPRAGAESTDGDARHDRKVSSVSTEMNDKTLFRLSKQMLATTVRVSHSKIHGIGLFALRPLKPGEMIIEYAGEQIRPELTDKREAYYDSRGIGCYMFRVDANLVVDATLTGNPARFVNHSCDPNCASRIIQTDVGKHIVIFAERNIAVGEELTYDYKV*

>jgi|Monbr1|27776|fgenesh2_pg.scaffold_21000111

MFEVVGVPGRGRAVRATKALARGQTVLLNPPLAFVLRHEERVARRCEDCFVSEKPEHRLANCSLCHTAAYCSKPCQTRNWKRAHKHVCKLLQTLPENPQPPHIIDAAAMTVATLVALERRAKLEDKESEQASPDPGSAVRQPRCADFWAMAQHTPTLNSEELDDVLQLVAVTQCPGSTDKQRVMDVLQRADCNNFSIWDELLLPRGAGVYPWGAILNHSCEPNCVMTYRGPLHAQAVKALRDIAVGEELCHSYIDLYAPTGQRHSHLGDQYGFECDCALYLDGALAELDALPEVTAAEMRLPGKMLHELECEAEERQRLLSLYRAAETLKDYAWSTPEEELVCLLQGYNILRRLAHPANISLTSIMTRLQNVATECGRLEDIALPVGQHLALAYDHVYPEHNPLCGLQYYRLGDVANLAQKQALALLWHRRAYQVLRTTHDADHWLLTQLQETYGF*

>jgi|Monbr1|29283|fgenesh2_pg.scaffold_33000033

MAAAVQEERTAEQGRRLVATRHIEAGDIVWQESPLACAQFLWNRACGYRACQHCLRSLESPQETVNRLTQHDLPLPNPSHQLEPDARIPQFSCPGCPETYCSEACLEADQVFHAHLCPRAHPTVETLNEAWRDMHPPPESTSITLLLKLVILEHNQPGILSNFCHDFAREINGEMVAHRLLAPEFEASLSQLNELVWAWAEECQLDNAQQWLKPEGFRRLWSLIGTNGAGVASNTLAAYDRQLSALDLDDATQSEVDNTMNTIYEIAGEVVGEFLDAEGSAIYATHSACNHSCRPNAKVFFEGGNFELTIRAEQDIAPGEASCLFDTSEVTISYLDDHILDHGGDVRREVLREQYLFECSCVRCLNPTTTDDEDEESGDDGEQDQEMEEDDMVFVVSPEWFMLIETAFATLTVGAKLNGTKQQ

>jgi|Monbr1|29407|fgenesh2_pg.scaffold_34000047

MALGAGVALCETAELGRFLQAKTTLDAGAIVCASRGLCFHHDREATTDILDMLFQTTPAGTAKREALSHQITDLCPRASDDVDVLKAALGHATRALDLNDAVLERVHTKLGQNCFDQGFFPDASLLNHSCQPNCGVFVPDREGGQWRTMEVRATRAIATGEMLTISYLEGSRLMLPVDQRQRHLQAAYA*

>jgi|Monbr1|30554|fgenesh2_pg.scaffold_68000001

MDQVVRGSEAGKGDDGNAVTQPMPTPAPYTSVDEVMLPARLQGRNNVYSGCSCTGPCAPETCSCLCNGRARYYECNDNCACDVATCRAGRTTQQPTALDIRLVWTSERGHGLLTGTRIPVGTYVGHYTGQLVNVATARARDSAADAVSPVHTYLLVLREHTQRGVLTTAVDAKEYGNLTRFINHSCAPNLELRPVRLGFVPRLAFFALTDIPAETELTFDYGGAPPSAKIATTLPADTDTGAPRHKRHVTAPELALSAKPCRCGAPTCRGFLPLTIW*

>jgi|Monbr1|30718|estExt_fgenesh2_pg.C_20049

MSSNKQEPLEDLLRIGRDLLKTKSAEHHQHVQAKLQDNLELVRQRDRLLYGKRLRMRASGVDLKIAKTPPPSFSGSVRSRESHAQSLLTGQADSVPLLLMTKQTVLPRYCAWTPIRRNLMIEDDRILSHIPYVGDEETDQFLNALYEAYDDNLVDGRKEDCPAVINDVLMTTLQHWSAVHRNGAAMPPSAKAARVLGHLVGRSGEEIKERVLQIMDNCALSPTGQRAEFGMAPDADTPNLSVTAAELVDSYRNLFCRRCYTYDCRQHTDTELEFEPGLSPDAPTTPCRREPCGDHCYKHFFRSLTEHNDPAQLALAERTAFCHKLAQSHVLEKASAWRPPPVCSFGSEMKAVHVPSKATSSFMESLKQCRACQRQQIADGFECGCCQARIRGKLLQEAAPDLHGLAEDALAHACLERKECREPGSPPAKRTKSNTSLNIDNCQASTLDKLRALLQRESKVLQNPASLGKELAPILEQILTLLQPKSQAWTQRDMSLFEVGQSIYGYDYCELSRYIGGGKTCAQVFLLAAHKDASTALPANYGTEAGTGTPQHYTPCYHPGRPCDQDCPCVQSQNFCEKYCQCDASCPRRWPGCSCRGDCMTNRCACKCADRECDPDLCTVRLLGWGVFAKNSIAKGGFISEYRGEVRWAACCMLCVCVCVCVCVCVCVCVCVCVCVCVCVCVCVCVCVCVCVCVCVCVCVCVCVCVCVCVCVCVCIISQEEADRRGKVYDQLKCSFLFNLNQEYVVDATRKGNKIRFANHANDPNCCARVMMVAGEHRIGIFAERDIPAGRELFFNYRYGPTDALKYVSVERDQEEEPVDWLDIDWVQPLSDFTGVTMAVGTAGKPQARLTATILRHPLLKRRVQVRGQLHLAAGPVDPKLPEGTVHYILVLDTSGSMAGRPIREINTVLHELPKRLEGNARVHVVSYNSTASLYPDARLEDMELRGMGLTSFMSAFHGMIRLLEQLKPQAQDSVRICFMTDGENTRDSYDLAMIELREALNALNVRACVVDVVAFGVTRVHQLLETLRVMGQQEGAYLYAKGSDGPTILQEMMTLILDSAGFASRFEKRISIDCQPLGLSGLTVSAIQQEQAEASGSNQEPRAQAKHDGRETADDNDGNDSLYGFSCWAYEPKREGSDAGWEAMSHPVDVILPDGLDDGAGQPLRLSAEATIHTLTEAERLYYDILLDQVDAEIDRLCSIKGQALELDQRRDVEALQHKLNVCGRVYSRHDLGRVHRAELLTRRMEVQSRLDELHQLIMSAARAANSDEVLSQMSNLRFAGKLKARRQRVLARRVADNSSEQDLTAQLQQLYVRLPAKLDLDPAMDKYFSCLMSQATLSEILNDTADNVLGFGLSIRRPEAMIDAPSLVHVDEISLTFLSRESMLDALKYKLSIADQLAAHGGFEMRLSTPAAMTVGAAREPINAWLPLYIHPLHWERAKLLLKPSLGYFCCLDPLAFHEYQLDVPLVVLATMLVRLCTQPVQRGAELFVSFRRTCRALAEDFGTLATLREKLSLFCTQPEHRLKHHVTNPMTLLGAYLILDADEREHLEANHFSQKMGLELLRRGLHAAYEAAGPGTLTDMAARLVLGHKDQHFLSTEDDAVAQLVTLMSQTSAHDDDDDQADGDNHGDGQMAGDQAFSEAGRQAGDDTSSSSDACAATYRLDQTGFNAEVEAHPLVPMTLDNATRRALTVVAQSCYNAVPGPNIQQKTDAARARVEAWWAENQALARASLGSEEMQELLAQRDCFSDVKLEALARILRRGRAHQYPQPADILAFKNVAALLGDDDDDDAGDGNQASGVSQLEVQAVRVRAALENLARESTLRDELQLWSGTVPVTAHQLRGLALGILQVHTNKSARAAVAREGYRDVDAVDYAQRVIDDQLSRLNEREEANLEQRCRQACVPAVLGHMLRAEDMDIFLGLLVTLCSGRDQAYRELWALLLSDRTQPRALTAKVRLVLTGMYRDAAVFDRGNPNFPSGTADEQLRKRLGEYDYLDVRKACLARVRYHQYRESGIANRHGSSNEHPHLYWKCALCRANNHPFARLGLE*

>jgi|Monbr1|32426|estExt_fgenesh2_pg.C_100098

MKKSRLTPPATPSREALKRLEDELAKELEQLKQHADDYQDAQRSCHPEIPSSSHAHRMEREALLKQLRYVDQVRETQQQDALLGHIREAARQPALSNDNVLSFANQELLRRWTEHAPSLPSRVLATASQEVAMAEGRAVELTRKTADKSLLLEDLIPVLTEYQRECEARRHLDRFLVRIRWLALTDHDVIRDAMMGRTPVTQRYIPGSLADLMATRAGSASRGRHSSHGLSLTAANGDDNPLLRSTMPLNQPNYANHQLRLRFLGDFFALQFRLSEAPADQLQAFYQAQHLFQRRFTEQCNEAAHDRILIDADIGFHRGPQPQEEHAQEKRTAHLLSVGEALDELLKMELEFALRNDYDYATLRLRQRGHVTRSSGGSKSLQVSSFADGDVFTGGLWQDLYSAERGVNTAPTRVLAPVTETEEEEEDASGSGSSKDKTNRKDTLAAYLLLRHVAVRECRSQVAAYLNYFHSVQRTLHHEHAQLQATRSAESRLDLLSGGQEIARTTGADPSKPTGTDNETAPGSDSELNSTPDTDSAPGPEAKPKFVRDRYKWHGNCVAVLDSMGDPRHYAGAQAEMARLEHELMSLGTHYLQRLAKTTYASQGQRKRQQAATRRASRPRSASASSVSAAAAEEERRASAAVDLQLFGNGTADRAEVLFDLWLWELNFVQEKQRLLAVLHAGLDLTRDARQRASLVQQMINVLHERPKYDLEADYFVTSFHLSCVSYRLRRELYLGAVTGLLQRDSDASMAWSAAGHSVQMYSIDQRLALPMPVVDLFTVHASVGNVAHLAPAIAFALQEASRTLHPVSQNQFSRLEAALLSQALTVWEQEQAAPYFEKECSVDVASLARAPVGRDPKTLVDLVRRVARSELSQLDMEDQLVRGQGLAAVALETLRCRSMLLTQQYEGIVLAKLHRRLASQLNVGEHHAFVRPLTFMAAGHAKAEEEDPYVPSHHGLALFELDDAMVNNMALASEERVYELLTKAVQRSSRVGTTDQSGHSFQPLAGPLGDLQAAMQAQISHTALLSTVVDHMEAFGAMDAAQTQACRDKITSSVGDTEAAPAHAIASDMCGFVSIQLIKTRARAQMHTQYLQLLSTRDPYLLRREMVAKFSELVADTCEDLALRVQIARYLQSLRGLLNAFPSTRDNYFLDGSPELTARRQATTEALLHDKSAPSSSLQAKMSAAEVDEALERQKRTPLKLLSEDGQQLLNLWYLPHRAEVAHLYSNLPVTQRRHALQHLLALLAPLHDIIMFLCAHARLGSSHARLGTYDVAFQGVGADWGGAEGIGTELRKIKSEIAGLRAPNEPACVTEFLRLKRNMMFLEWDLTVRFYVRETFLGMRNQRAYDTVTINMATGLPALSMARQCASVLRNRFHGPAQLCAWFELEARQGPFLLGHPAYSEVANHMELCLAKLSDMERHNANGEVLGVSLLLEDIIEQELRESDHKDKAETYKVLQDFLIMSIRLEQLRFAWARLTLNLDVMSPETLAQFLPLYDAQVVAPVVTSLRALVLEPARPMLALDNSRAIDMAYTRLGPQAEQLRLEKRSRAQQVAELETQIRCALVSRSISKVARVRKLLQLSTSDTEQILPYQAWAKLQVQRAPERSRRQHERTLQVPQHVARLELINDFAARLLREARMDHDDGTVQLPARVLRDSLKQLGQDMHSRERERYMTFNELHKAIVAELISALDQSEAARREAVRASNRSDLEIAHAVECGIADRTYDMVHEITALRATVSRLRDEHRTQARDIRESVKRDYDDLVNNLFSTSFALKNQFEEYRLSLHEDVVEGLSEVRRTALQRMKLISVGKHDEKFEAGLKKADDLRDVQNANASLTALVLKMRTMNDWKRTGLRSHYGKKVHEAAMTANAMKQQLWDTQLIAQEAKAQRQHEVTALRAELLAAKKGLQSAQADLKHERAAREALEQWKASKVQLITAVEKQAKVYNKLDLDVEKVADQLEEQMDTIAQLHAEIDRLQRESQLRESKLRLDIKATQQQAEEDDASLASERRPSRPMSAPLRRRSSLRSPSPSGMSTTSERARPTRPQTTQAAISRPRPPVSPRSGTIRRNTIVGPIPEDREGSWETVDLRWNFQPMPLNDEHPNQHFCDKYYTLTVLTYKLNLSLSLSLSLSLSRSFVLHTVAQAGTPQGVGGHSIKWFMMTAFHGLELVAGLLSAVGAGAVVWCYYRFDRQHPAQIILTVAVLDFFLALKFMIRACAFLSDSNETNSHAESLHIFHDGCALSIAWSAICETATMAWNAIWCLNLMLQLASPAEDTRRLIRSYHLYAWSMVVLMLGLQLGATDRTVERSGDYHYCTMTPGGLKNHVRLIEYIFAMLQVMIALVSAGYAIWRLRQGGEESHRLLYHHVAYALVFAFLWAFEKSFIVNNRLGSTGHQSIGLLWQAQGFFVALTRLSELRVLSRLFSRKQSQYDYIDYTESSVQDSQTGSGNGGQQWGPSATVRSEATDFLTGTSHEKKNNKLSFTHGSASPSLSLSLSLSLSQTNSFSLSLSLSLSFSNRPNQFILSLSLSLSRSLSLSLSLSLALSHSAFAEGPSKSSDGDVGGSGPFRDPCHFNHLNGDTSDMAKPSDSGGNSASEEEGDWVTRCICTFTHNDDFMVCCDKCECWQHIDCLEFDADHVPDNYLCDQCRPRPLEVFKAISAQADKSRELARAERRSGRGKKFVFCEAAVLGDLQEADLEYKSDNQSHCTDEVKAVIDAAVPLEKLPDLLAWCQDASLHPALTSLEIAKNRPAIFVNQAVAKDMPLLEFCGNVVTTDSLELEAPVTEAPQPFIWFAKSMPVCIDARVLGNESRFLRRSCHPSAAVKLCRSGSRHCFVVTALRALGKGTEITIAFDVDPAKCSFFPVCACGRSDCQVNLRLRQHQRQRWKAERKQKEKEKQQHQLQIQQVVQKAPGEATPEARRPSGSSSAANSPLVQNPSSNSKMTREERKMAAIMARFAQMEQEQLGEPGSVELMRSPAPMMPLTPNATQKRRSSADSPSGSKKSKQPQQQHDLAVSGQHASQQQSQHSKRTRNAASPRPSSNKLRPSSIADILHAPLDSSPLNPYSSSNRRRSGNMTVPRYRLGQASQAPIAQHNQAAQLAIDEMLYSGIKGVLNKWQHPLRYVDPNRGQVVVAKFPEAEPAGAPGTEDAKQTSAKGTVSAASTSSPLPWHKQRLHEWCQSQAKPATAKETPADSSAEQPTQRDEAPPESSGESGMQPTSSAISTAPKINDTIARTAAEAATAEAGVEGNTAVLPGTASPAVPSLATSTPTDSMPGVGESRAVKNEPVAGPSAVADVPSQGPARTDSTEQDTAKGSEEESLASPSPAPKKKKMSLGEYLKRRRESSAPNKDG*

>jgi|Monbr1|32594|estExt_fgenesh2_pg.C_110223

MVNPTFHLLQATQMELDNLASTLTTASRYHDLLPCALPHRKPPPTTTRVCAQSIRGVGAQVKSRFVRLLKPNVTLLHTFLHALFTHTRSLSLSLSLSLFARAPTTGRWLPGSQVPSNLLKEFNQYSGSMRIVGRDEEHNLQWERLLGNQETKINEWCSLCPNLLAIAGPRRLVCSKCNTQAACHDCITNRLNYSEEKYRICTTEWNWECLECRSCYVCGKDSESVLLCERCDRGFDLACVYDPPLPTQADYCPTCLDVLAGKRTAINNPNPPLRLTLSLQQPRATKLLGTPTHKAPPSKTAASLPRAVSSATKSVLSRGRAMVAGRRAAGPVTIDSAEGDGPEEGEFEEDEAGSMADVGAEMDGEAGSVEDHHEASTVFVEDTSSSYDDRSEVETVDVNNNVQPWSAAEEDFLVSSPVKTEEKLSEPPHTAHRLDLHQKSRVRGVAESPFEREARFEQERPAALHHGKRPPVYLDSDSSHDLGDEEVNGGPHRTKRLRAPPRKRRHRAPQPVTQQTVATSPRPVEKSSVGTSTEAMFAGTDAGPLCMVPVALVRDLREAHDLLAHDRAGALKRFQIRSETDPERFQDHWHWLRHLFTGRIQNNPAWQSLAAIELPSRPATPPTPTPTSPQTAQES*

>jgi|Monbr1|33094|estExt_fgenesh2_pg.C_160027

MGRRRRCVLVLLLARAAQEVAAVARAGVLVWVQHASFPWWPGMLTYDPHSGEFTRESANHRAYHIQDGGLDPTLLKVGRLSPKLANERAAALQQAHAVAELSLRERILRWACDFKTERGDKAKPKLEESDKDQATAPGTVSAPPRKIGVPRSKTAYQFYLDHRRADVMHNLDDDGTSKFARNGKVTKLIAAEWQTLTADERLPFDKQAEKAKKQAISKAQEMRAELEKSEPVTPERTASLKPAVPRTKTAYMFYLDHRRADVMKGMDDDGTSKFARNGRVTKLIAAEWKALSEEARAPFVEAAEIAKKTAIARTLELAQADASCPDSPGDRPPPMVAAFRLFAQDFLAKAQDSGDELVHLDSADQGLRVHEKWMALTRTQQRAFVRRVQPDALPNKSGASTAAASPSASVSPDEDQASNEGQPAAKRSRSSRTTMTANDDTCRICGELGQLICCDGGCRGAFHLECLSILQPPTGEFRCDECSTGNHTCYTCDKVGADLIKCQFPHCNKLYHRGCAEKQFKADNFCLVCGTGGDLVVCDGCPGAYHAACIKSTFAFTGKPDEQGQWFCHDCLTGTKSMIHDVVWAKYGSYRWWPAVILSATEYPERCGQSPAEGNFLVKFLGSNDVAWVGHESIIPFSNGDQKNSYFTRNLKAAKKSFQEAVTLALDLFTERKQEREDQLAAIRGKIIERPPRFKLIKRNVYLPPAAKPPHRKEEQEICRCDAALNCADMHSCLNRMMYIECDAKCCNNGKNCRNQRFQRREYPKLIPFKTEHRGWGLRLGQDVEEGDLVIEYVGEVIDGAECRRRIDQYEERNTSSFYILSLGSDTFVDAREKANMARFINHSCDPNCVTQKWNVLGETRVGIFAKRALAKGTELTFDYMLDCLNSVKKTPCHCGAPNCSGFIGVKPNRSGTTYEDLDRTDDECFICKDGGDLLMCDKKNCDKVYHLACLGMNKVPAGKFICPHHACLKCGRKATIFSETGPEAYCSMKHIATPEVRALCEGKRPPVTFKSPVPVLPAVFANGTWAKRAKRAKADPTSTTQAQAASA*

>jgi|Monbr1|33144|estExt_fgenesh2_pg.C_160117

MSARRASRTSDKTNDVRLTVNNLVWCKLKKIPHWPCIVTSDKEGPQRFEVTFLNDKGAIEDVRVKDLFYWMDERREAWEKAGRQKHTMPNFPGAVEEAQKHLKQYPKIALDYYLGADKGSPPQSKAKKPARRSSTQDPAPMRRSRSTPRSGSGSAQTNETEPRRQSHSRSGSSPASEAVKDKNSHQRGQTSTPALDRKHSSSRATSAGSGLRRRSSTRLQDRNGNSDSDSDSDGDLPDVLTTASNSKGFPKGSIVWASLNGRTNFWPAKVKEDSTSNRAMVRFYNYKKGTQAATFLIDCKSEKIRKAIELATVALRARQRREKAEDDMDRLLNRKGDVLSSSSEASESEATSMPPQKRSKRPSTTAPHQQDGRRAHRVRARSRVVETDTQVATVSAVIKRHVRGKKLEQSLAQHMSYGPLATGYKNVDELPHRRRIFCDLVCLPEAIIQAAALHDNVPLSEERPAAMAQVDEPPKRYHIFISYRVQALAELAERLCDKLQQLDIDNEHGLRIRCFLDKQNLNHGASWRQQCTDAVRESCLFVPLYSEATLAKYLDTEEDQSDDTFLYEQALALELRRQGEIVFMPLMVGTVDDQERYTKFSGFGRRFSSALGPSLESGQTLPIKTLFKQAFQAQGIFVNPLELGEKVVQLADDLSRRVWPKFRNRWHDPAVLQPEAPVKCVQCEELYMPSQNTRGSCRYHQYPDWGSVRACCGSTTDPWRKKSQLGCQVGPHQSRHHNEYPYESKVILSQAFWNDVGNRKRMLVLESSDYETPALSVHAKLASATTQTRMTAFKGHLCLWLGYALRDYVAFIGPPELNHAEQTLSNDSGNTLIHRFERENGAYAEAHWVMEEGRVVGMRLVAKSATCEESSGIVLFDWDGKDEIKVREIKDVKDGQLAEYDPIDDVPLSCQPEALLSGGFWRQMLPGPGRGDLQPGLSPCLTLMTSGEMEANPVNIINADVECFNWTVSVINKSSEDNILLSVKTEFAVPGGEMQACQLQGDILTALPINLGSRQATRVTCRLIVPVPVQRGSAMWNRSGLARLHPVLFKLAVTDMHGETATLLHEFRNQVACLPEPRENSNELLRLPIDSGSCLDRSFLTMSVEGEDKLCIHGLSSRLEMTREVQRCLVYKAIRDKTDYFNIDMKLHADVPHQLGIWVDRSLRRCVALTLELQGELATVRAIVPMPFMARSVHEALDPAAPVSKASRQLTSLTLQQSFADWAVRPGADEPEWFSVEKVLQHQPFVAPDWQAYLPLAQRVGLTESQNDAPVGRERTETMPNLTASKASSYARRTNEHYHGTGKQQQGHADNSSRKPPIFNIRGVFVRYPHCNLIVITLMEEVENQADHAARPEPLKVNLKLHPPKVHEANHGEDEDEDRERERIQSITRSLSLSVFSLSSFSLSLLADTRSLGRNLVDGADSFDET

>jgi|Monbr1|33338|estExt_fgenesh2_pg.C_180090

MGQRRMAAVAVMVVLLAGLQPAVMDIVTTLTTTTINNLKCQCTNASLFSPNEQIAACRKAKLIETVDTLINATFETRLAALEAVAFGDGCEPDAYSGTSPIVLTTNPPPLNLTLCDAETDMFEYTLPESGVYEVYLSPIDDVVSCIMPFVKVSFLLNNMPIQFDNMTTDGVKVTFTSPDPVSGTIKFLIESNQTVPCACALPLCLLIHSSIPLSVSLSLSLSLSLSLSLYFSFSNRPNQFISLSLSLSLSLSLSLSLSLSLSLSLSLFCDRYNPRKPIARAWKCIRCKTCMRCHKKGNADQLLFCDGCDAAIHTYCCRPKLNGVPDSDFYCPNCQGAPSASTDKSEQAKPGRTSKRQSGAQESSQPTQARDKRGSTIHTWLSPKPTNPSTPSPPSTGRTPTRTSTRGRGRGRAQSRTRATDRGRGTPGHATAHDEPSSATASAENRRKKTKHKSRARLNPLRGLDVPKAEANLFQRAFSQAQAVLNRTPLSTMGRLSVEIGDHRIKAWYPGAYPHEYATLSDIYLCEYCLRYFPAREQVGRHMDKCECLGHPPGTEIYREPERYDARGQRLAQLQLWEVDGHAAKLYCQNVCLLAKLFLEHKTLYYDVEPFLFYILTVADERGCHFLGYFSKEKHCLKKYNVSCILTLPCFQRMGYGRYLIDMSYLLTRVEHTVGSPEKPLSWLGARTYEAYWLRVLQEYLVSHNEVSLDDVSRKTGMTMADITSTMAKHGYIKRIDDSVKLVVDRQALGAELSAKTARRPICKRTAPRVSDRQREDLKARDRGGSRMDSNQGSADVPISCALVVLT*

>jgi|Monbr1|33774|estExt_fgenesh2_pg.C_240060

MTASVPGEDKWSNVENRDLGGPGLVSGHAYSLIGVYKSKRGHRLIQLRNPWGELEWTGDWSDNSPLWTSDMLDEVKPTRNAKDGLFWMSLEDFLQYFSAINICHVCSAAEGLPWRESRAKNILNLQDQQRPTVPSFTVRSATDCLGWISLHQDDERVLHGQPYVDLALIVLLYTPDGQLSFVEAASGSWTRQTQLRLQLQQATYHVVPFTTGRTHDKVAYPLPSANVFDASHLLSNKYLSIVWEVFRRYDLDMNGFLTPEDFARLAMRAKLVASEQEAYDVAETLDQRAGAGITRDGLVTFISRHNESEIHEALLNLGYSQDLQTTQERSVVLSVHSTANAPIVQNAYDQELEHKALELWIRARGKSKEYDNGKLLVWELGRGDSGTTIAVETHYTREAEVTIDCSGSNNVAFSNDNPCLVKHVEPGKMTIFHHLSPLDPTKSWRWTYQLNLRTAAMEPEAKQAKLLARPAQARMDKFLSWDRGWRARMPGLTQRWGGRCTSMNITISPNVKVTADDVVHGFGMVAATSLAEGDVLFEIPRSALITVNNSQINQQLSEMAAAWAEEEDEPEDGDGDPRQWTQLVCAMMVENTDPASRFRPYLDFLPDHTTLAHPMLWTSAERDQLLAGLRLAQDVENDLEMINSHFQELALPFLRRHAFPALAELSDEDLRRNFMAFAAVVMAYSFTDDTTGEVCMVPVADILNHVTGKCNAKLYYAKDALQIFNTYGSLDNQQLLQKHGFVEPTGTPFDESILPVEELVAALRPSFEGVLDDAAVERKLDLLLERGFASGPPAYCALGRIVLEDMQDHNRNFLRAISILVARPEQMDALEEAAYTAEGYGSDEEDDDEEEGEGEGTQASAASGHLGRDATCFTNYLKVQEDPVLRKLPLEALGAVLEMMQEKIDANQARLAALASNPSSKVLGVSQLLRDDSAVLTSWRQIALE*

>jgi|Monbr1|34767|estExt_fgenesh2_pg.C_500025

MAETTGATAAAATAAATTMAGEKDGAAGVAKNMTTGHAAPATIRQKQEDDHDDGERDEQDGDGREPRTAAESSAGVPAAATSVAGGSMPEGSVHRTPLSGKRSMSSSEIRDPTQNPRLKTRLCTQFMTTGSCRYGDKCIFAHGPHELRGANATMYMTMMQTGAVNPMAMAAMMNNPNGEPAAKRKPILNMARAKTRLCKQAPIKTRCPPLRSCLSPPLSLSLSFVCVPFRRLQVFSRSFTTTCSVFTVYSMSPSPPRLSHSRAVTFFFLSLRSLSMPSNALDPVLTLSTRPSRLPPNSSLSSFPDAHPSPLSPLSSLLSRSLSVSMLLSTLYSLLPTLYSLIADLFSLLSTPYSLLSPLPPLLPTLYSLISDLYSLLSTLWSLISTPYSLLSTLYSLLSTLYSLLSTLYSLLSTLYSLLSTLYSLLSTLYSLLSTLYSLLSTLYSLLSTLYSLLSTLPTLLSLLYSLLSLLYSLLSSPPSPLSPLSSLLSTLYSLLSTLPTLLSLLYSLLSLLYSLLSSPPSPLSPLSSLLSPLYMHSLLSVAVDGEEEEEEDSSGGGEGILSVIPNAAPVVINAATNGKLTAIDQPHHVAGSFLGTGTGTMHVSPPFCSYPGPVTVSSFQRRSWWRQLKRIHFPLKGFGIITTADIPAGTFVMEYVGQVLSTEQFAERVATTYENRKHFHCLNLDGGLVIDAGKAGCDARYINHSCHWRVGIFAKRPIRAGEELTYDYNFESFKEDMACHCGASNCRGFIRPKARDDRLRRTSRLSALRLAIEEGTEPAQLWRPFFWHKGSMESTIEGQRNFLQAERIEAERLQQQAQLMQSTFQARFAAWRRSSRVNDVLDAHPEWLIDQRVAVATTLHDFLSLLSSHRSKTLGLVARPLMRALPRLARENGVLTWDDIAERVDDGQYKNIEDCLQDLLALCDAFFHKFARGSKEHRSILAVRHLILKNRGRVLQDQVRNTSEAMAVAVRSMHGDAVEHGADANRNVDDKTGVIAKTPWSDHIRCVCESLVDEGNMVQCCSCGTWQHVVCMGTTMAAAAEVQDYRCWLCAGIPRSREVPLDSDVERLADQARLAPQGRDLQAQLIDAEAEARAVEASRDALHSELSEAKASLQQLTNRRNKTRSFLGMVRAALEADIAMLPPNPARWSAVEVAGPVSTSESSSVSDLPLYELDGLSDLDNKNLGGGRGAGPLSTPLASSDMALPVATTPTSDSIGTLHPTRPPTPPTLSAEGMPILAAESASIISKGGLTEARTRMLLDLLFSLEHSDDANALTQAVDEMVHLRSTATATNTKSTELITTLSYRRKGHGSVTLSDVIDHSRRLLAQTMALQVYDNAAKQLKAKHAQMRSASRRLASRVATLEEQLDETLVHYRTLEVTNPWHDAKPALLRLGDCIYVVAENSEALEDHLMSSVRRSSLKPTKQRSWPKGQGTIPANHPQARQVYEVMRIEMLWRDANGQPWLTGRCFFFPEHSMREPRHQFHPQELAFSPEMRSIPAHLYRGHCCVLHRAQYISGRPTRSGHESHVYLVDRIYRPRPKHDWSNLDRPSMQILQVCEHPAEWQAFPKPLDVQRHPIQDEEEQRAALRERTAMQQAKRARLLECVYMFKTESHDLIEDASYLLSSRRREKKRLTF*

>jgi|Monbr1|36134|estExt_fgenesh1_pg.C_40136

MSIEALVHLVCANCGGTGERLKRCTACSMTAYCNAECQKQHWRHHKKECNPCVQCRQPCRRNARAVPGLVGLLEICSTCFCRQWGDDISDKDFFELIFVWQNARPHQKGLRGLFDIYSKRHTKASPCRQRVLTRWLMQLAQLAGLKLKADVVFDVNVLLCVPPSLAPFSPGKPMIVACMASHIADEDQLAKLSTCLTSATKQSIQVHELILSWSCAPKLRVACQAMLAQFKVTTLYRCTPQQPFEHYAGIATHLQQTLLQEVPEENVRLVFSRDDDLWDMYRIEQILDIVGRQPYFKLHIMPAAKPEDEAPILDGLDGFGDVCGNILKMELIYRRSIFAGPIVEGAVYEFWQAVVSPKLFLGFFEIATQPLLRDQYCDLGYAAYLSAYPSYRAAQAERDLAWCYFYRGYFHNFRCNVYEGCRRHMEKAALVSYCDPRRLPYLLCMLCDCLLPEITDESMDDHPGGLEVPASIMRLYPIEHVLTLFKTVAAKYQFGIRFDNMVSSLKHSLLNHMPRRPAGKEHTEQRQWCLETFVSTFSEFVQGESKNLN*

>jgi|Monbr1|36667|estExt_fgenesh1_pg.C_70095

MGLFARRDIPAGTLILREPSAGCLARPTGYYDDKVPICANADVAIRLLQAAPELADQAWRQLQPRQHEFNASDPIRTLHQADLELSLRYMRAKLKAQPPVQSSGELQRLVAICMYNCFLTDHERQPGFDVLGLWPQAAAINHSCRPNATHYLDASAPMKPRESGADLPPEGGTMIIRSVSDIARGEPITISYVELGDPWPVRQEALRTGYGFACTCIRCTEEAALDSEQSLPGSKPKLEVAAHANGVSLEQLGRALEAPAELQVRLLSALHAKAAEGPASPGAQCVRYQLALRLGQILARGTPSERATAVQLLSQAWDLGATFMPPGWPAQSTVARYLVRLTTELGSTAAHHAWVTRFESSRALCRGGRCTRCEAYGLCPGPANALCLACQPTPIQTHD*

>jgi|Monbr1|36878|estExt_fgenesh1_pg.C_80232

MREELAEQLKSRANAEVRAGRWRAAAALYGQALAGVQADDDRTGEDRKKTIAAVINLNLAHVCLKAAAESDTLPRDRRHRTRDAQQALDRIDPLLLTQTQQQKLDHRHSLTAQSLAANPASEHPDLTGTRRLPPGASEPRSPALHVRRLPHKGRALFTSTPLAAGDVLLRERAFASILAPTTTQENYLRCHECLDESWAPLPCPNCPRVFCSTECHAKANHWHLEACPATPPGSEVTPLKPSSLRQALAARVASCLAQSDPSCDATTLDTVRPRSDPDPTGILGLQHSSNQTEGDEDMKEDDDDDDDGDKASAVAVHQGSWAQSHAHLPSQPWWQSAKCTQCDLTTVTGLRQHLLRVLRHNQHGLAQVELQPSEQNGEVGVVHQVYGAGLFVHGSLFNHSCVPNVHLHFHGDELVATASKPIPANSELTISYGPLAVRDAWHAARQTQLRNTFNFACQCIACAPAQAANLFPSARCIACGQPLRVTGASFFSLKLLQVLRPRALVQATSCSVCGAAGMDAVDLPALVQDRQELLDLAGVPTISPASRTTWEHEIKTRCHALASQYEPLHPDLLELFDAVARRLAIELGDAAAAKPWVEQTLAIVEHAYGQHSREAAEEHAKLASLSARSK

*

>jgi|Monbr1|37907|estExt_fgenesh1_pg.C_170157

MAELVVPLTPLIPSPPPLTEPTSTSNNEVHANPTMTFLAAAQRVGCTVHPAVRFDADRKRPAAHLVALSGLSDPTHALCLSVDTQPLTACASFHLDLVAAPFHCHVDADVPKDTLMFALPLHLVLTPDPGTDLPNAGKHAGLVLTLAQRLHCQHDGHSHLSSLSHIRPEPEPEPPSKKTPESDFWRQYLAAAAVPDAALYWDEARVHALRGTNLAASLPTQQAELARQLAALHASDYAQRHQIPVTTLRHAHQLVLSRAFARDSLPPLPASFASHAAYLLPIFDALNHSHTLVNLRFVLEDQALGFRTTQPVPRGGRLYNTYGDRTNEELLCSYGFELAEPSCDAVTLALVLPDIPPASAAWLAQHLPRDWVTEPLAADKLRLRAVLRSDNWAWLILAILISEGLDLDGPLDSCQIQPLLQGLPHEFVKAGVAPLLQRLAALAQCTSAPATGAPGHLYVTGVQRICAHIQQQATALLAQCTDDSD*

>jgi|Monbr1|9299|fgenesh1_pg.scaffold_14000205

MAARTEVAGAAAYAAVGVAELLWDESRHPLGAGWRVVAKKELAPGTVVVREIGCAVAASESGAICPVCFDMHTSTQEESELAPCRALAAREQRILAPLMQAGLFDELQAYGVDRQLVLLWLRVINLAATTSDDLPAPAAARLQAWRDTLWGLAHQDVSAPSDTWLNGVTMAAVVLVQGLPPAVVAAATDLLLPLVQQSSHGESSGSLVEVLVLLAGMLNRNGYSVRAAREPNRETAWGHFPVIALVNHSCHPNCAVVSRPGGELEVRTLGTVRAGAELFVSYVDLTLPRAERQAHLLASKEFTCTCYRCQHPDAFPHEHEASMPSCPQCGLALRWTQTSASGKDDTETATCYSDAGGCGAVVTASAVQSRLTEAREALSQAGEPSAVADVVGSLLPEEHALVMQARLRLFARLNKDEAHREGAQEQLAKVAASLSRLVPTLWPELLEFEWLALRSAHALACEGDQSDASRLNSRLTALRSRAERIFGLGHPFVEQLQFST*

>jgi|Monbr1|31781|estExt_fgenesh2_pg.C_50338

MNVMDPGGLPPSGHSSHANAIPQMPFAGFNPMPGMAVTTTTAASLASTPTSTVPPTPFNWFAANMPLMMGQSLTPDAFALALQQQQQQQQQQQQQQQQQHNNQQYDQQQNQAHAQAQAAMMASFGTMPWMYMPFLMNSDGYAALTKQMQPNHIASGLPFSGSMPTSPQHGSPVLGQIGANALTKDQPAAKRAHKEHSSATDSHGSKAAAAALLAAAAAHGERKGLSRAQPQTATAVTSTIKPTVPPRKRRRRKPKACHICGRMFSNSSNRVRHMRIHTGEKPYKCEWCDRTFANSSNRRKHEASCAKATGSTASGEVAPDAASSATRRRKLQDQLTGRRDKEEQDKIAKEEDDDEFSSEASLENENAEEDEDKSGEEDEDDEPEAKAEARMQQPTKRTSSSPTP*

>jgi|Monbr1|32717|estExt_fgenesh2_pg.C_120214

MQEAQSCERETARERQIALSALSPSLSITSLCLSVSLSRVTASQPNKPSQTAMAKRKQPPCRPITKSPTNPTRRSPNLLSLLFGYLISRVPPSPTHTLTHTHTHTHTRFPVYLWPSCYPTLSLLSTLSLSLSCHSVPRIHTLSPKCPPLSRHFVLQVTTFTHTYDTHTSLSLSSLFLSLCLSTLSFSLFSLSFLSLSLFSLSLFSLFSLSDSLTLSDSLCLSLCLSVSLSLWRTGRGSTTPDCPGRLAANATPVNLCFLSTSPTYAKTRHTSFSRHRLPTQGTPSPKKMDVESMHIPVSQTISVSDGSPLLQPIPKDLHHTNMEKLLHLQTQHQVHQHQFQRQQQQQQQQQQPAPAHLLPAPQQQPSLQTGMSALDPHMFQEFRHALQQQQQQQQQQSLGLVAPSSSAPVSHVTHALPISSAYGSAGCSPATSSQASQQTVLPASSIHTSQPPHQLQQQQQQQQQQQQQQPPGPPGNVASVGGGQPQSQAQGLEQQFQQLTPQQLQHLHRLATQSQGAAAHNLLPPQSLGSVSSQPSSVAAALQQAVTQPGHLQDFQQLQQLQQQQQQWQSHHDRQQQEQHQQQQQQQLLLQHQLLLLLQQQQQQQQQQQQQQLGVHASQLTALQQVARDTRVSGASTYQGGLPNASMPSASSGNSASFSARLGRPAGRSRGHSTGSIVTNPEYLNPAAFAAQFASNGYLADQGAVSAGSTDFLSISGGAEISASLSPQDSIRSNNKSPAHDLEHYRDDSNNSTSSSRAASVASRRDSSIAPLRSASREQRRSISRRRTHSAGAALAQRPTAETLRNQQQQQQQFQQQQKRRSRGRSTGAIGAELPDDSTIQRAENISNESMSPASLRSLLLRNDLPADKYPCGPFCTKKEHTCVFCDKNFPSHSKLIRHVRIHTKEKPFLCTICNTRFTQNCSLRTHLRKHTAADHSRMLAMLGTDALASPVGLQAYQEHLSEDNATQAQFAYGLDGLAQGVQDLDFEQAESMQSLMYQQQSHQQQQQSHQQQQLQQFQQQQQQQQQQQQQQQQQHFVLSAQQQQPQLYSQQPDVNHVQQAQASMPGYAGEDAMRRLFQGLGTSFDDPLGHLEASLSFDEGQARFLPLCSNCVTHAK*

***Nematostella vectensis*  (Metazoa)**

>jgi|Nemve1|102160|e_gw.63.79.1

GTGIFPNAVCLNHSCAPNSVAVFNGTNIYIKALEEIPVGEELTISYIQQLHPRETRQEELQTQFCFYCQCHRC

>jgi|Nemve1|105099|e_gw.75.15.1

WFKANGGTAEHVEIHDFGDQGLGLRATADLQENQVFVAVPEKLLMSVVTAKKSSLGPLISREHGLRSMPHVVLALHVLCERLHEDSTWAPYLNILPRSYSTCLYFSPDDMMALQGSPSMGEALKQFRGIVKQYVYFFRLVQINPEASRLPLKNSFTFDDFRWAVSTVMTRQNDVKVSSNETVKALIPMWDMCNHCNGPFTTGFDDSTKEVKSLAFKPTRAGDQVFIFYGRRNNADRLFHNGFVYTEAEEDWVNIQLGVSKNDRLYAMKAQILAMVGLDASGRSYRVLRGPEPISPELRIFLRVFSMNTGELKPYLFNPEGLPVTPLAELCKAEFTLSEENELKLWSFFHTRLQLILGQYKTTKQEDEALLSRDDNTLHTRNCIRLRMSERDILVSALEHAKAR

>jgi|Nemve1|115756|e_gw.133.54.1

MADEKKEKSLELDSAISSFLLWCHDNDLKLNNKVSSMQKGSCHRYGMVAMEDISPDECLFKVPRGLLLEPKTCGISKILTGKVIQNMLSQHEGWVPLLLALMYEYTNPTSLWKPYMDIVPGIDILDQPMFWPDETRQSLLQGTGFEDDVEDDKQRIERQYFTVAVPIMKKFKKFFDLKRHSLSLYKHMAAFIMAYSFTEDSPSFHGNNVPVMVPMADILNHHSNNNARLEFGEEELSMVSTQHILKGGEVFNTYGQLANCHLLQSYGFVEGPDNPNDTVSL*

>jgi|Nemve1|116044|e_gw.135.38.1

ITEHFRVRRSQRKCKSAVEEEKQQNIEKAVLLGLEEGLEIRDVEGKGRGVFAVKYFSRGDFVCEYAGELIDYNTAKEREQKYSEKAEVGCYMYYFSFKNKKYCVDATKESGRLGRLLNHSVHGNCTTKLISIKGNPYLILVTSQDIKPGEELLYDYGERSKDIIESHPWLKA*

>jgi|Nemve1|116282|e_gw.137.8.1

QECCMCHKMGATVVCNRVNFYNKVRCSNTYHFGCAIRNNCMFFKDKTMLCHEHRPDGDREDRLPSYAVFRKVFIHRDEIEQIARMLRHNQESGGDKLQTLRIGSLVVKSLGQLLLHQLQTFHTRNAVYPVGFNSIRFYWSMREINKRCQYHCKVEECEGMPLFTLRVNEQGHEDTVFKGKSPRDVWKQVLEPIHKLRKEAVLVNLWPAYITGEDLFGLTEQFVLRIIESMPGVDYMQGYNMRYGPSSIMEMPLAINPTGCARAEALIKTHFRSTRSVSSISTAGSNAAGNSSREAEESTSLYMKQFVYSKASQYRKLKTEWRQNVFLGRSNIQGLGLFANRDMEPGCMVIEYIGSIIRNEVANKKESIYESQNRGIYMFRIDSDSVIDATIAGGPARYINHSCMPNCVAEVVTFEKEQKIIIISSRKIEKGEELTYDYKFDFEDDEHKISCLCGAPNCRKWMN*

>jgi|Nemve1|116372|e_gw.137.13.1

MCLCCGSFGKGPEGQLIVCSQCGQCFHPYCVGVKVNKMILSKGWRCLDCTLCEGCGKGSDEARLLLCDSCDISYHTYCLDPPLEKVPPGGWKCKWCVSCDDCGATSAGTQCEWQSNYTQCGPCASKTSCPVCNIKYNLNDLMIQCLHCDRWLHGSCDGLMTEEEVDRAADYGYQCLYCRPKTKCSLGALSGMSCSSSASSAGAASFSLFSSSASNPLADNILLTESGIKQMSRLKISPASRRRAKVKNKIK

>jgi|Nemve1|123168|e_gw.197.62.1

IYECNSNCACSSQCFNRVVQNGIQLRLQVFKTKSRGWGLRTLDDVPCGTFICTYSGQIMNEEMANKEGRDYGDEYLAELDHIERPTTRSLFGEEHCYVIDAKAYGNCGRYLNHSCSPNLFVQNVFIDTHDLRFPWVAFFAQHNIPAGSELTWDYMYEVGSVQDKELRCYCGSSECRGRLL*

>jgi|Nemve1|124123|e_gw.207.2.1

MDVTQSGFIVKETDSERGRALFASRDFKEGDTIFEEDPLVCSQFLWNAAYSYTACDHCMRSLETAQDMARRLSSNPTLELPYSAECCAVTKAGEPISYCPQCNVAYCSENCRIKALDQYHRILCLGTSTPDPNHPLVKLQETWKNIHYPPETANIMLIARIMATILQATNSDVKKGSFSHFCSNVVNKEQQIAHKLLGLHFQEQLDMIRILLSEAMYDDRLEQWFTPEGFSSLFALVGTNGQGIGTSSLSLYVHNIDSYPALSDDERQAIDIFLNQLYEEMERVSGQFLNCEGAGLYALQSSCNHSCAPNAEVTFPKNNSTLVLKALHPIKNGEEICISYLEECQRERSRHSRLKYLRENYIFDCTCTKCELQANEPDESSSDGDDEHDDE*

>jgi|Nemve1|135947|e_gw.365.41.1

MKTDEERVAFALRLPSVQENIIIKQTASTKSSSTAASLRNEGNTYFQKRQLGRALEVYTQSILVAPSTDLKVLSLAYANRSAVLFHMKEYELCLRDIEFAQKMKYPGEMVYKILDRKARSLKALCRIEESKKVFEEAIVSSKTSKLKRKDRESWVKQVEKQISECSNQTENLMKSVTHLEEARSGFSTPVQNYGLHEKFVSISKALDIKYTEEKGRHTIAARDINIGDVLLVEKPFASVLLQEQSKSHCHQCFVHILAPLPCSYCTTVRYCSEKCAKESWDAYHYAECMNLEHVYVAGKYGHLALRVVVKAGFQYLKASVKQFESEEKKCDPAELGCNPDGVYDPSDYRPIYHLVGHTHERTLNDLFVRTLNAIYLLRCLEGTEYYGDSTKLPSREDQAFIGGLLLRHLQSLPCNAHEISELQLSLKSVATSEAAEIGAGIYGTLSLFNHSCEPNVTRFFYGDKCVVRAFSSIPCRGEVVDNYGILSALTPRKQRQESLQSQYYFKCNCHACLEDSPLYSELIKQDVPQLKCANCRMALAGEILTDGKLVKCEKCGVPQSLEDKANLLRKSEVEYNEAMTKLLGEADVSSALPRLEGHLRVLEECVCMPWQGFNSTQELMKQGYNMLANCHLIE*

>jgi|Nemve1|137828|e_gw.414.17.1

GANIRFVITNDDGLRIEADSHKVAWRMIFDLVRDSRSSHLLKDLPFIGVSDRCMIGVTDEPVTYILEQLADAHLLRNYRFQYNPPFGTNGEDGLTPETYCVNAFCGYRKKMYDMFAFLASEYRTPPQLDDNYDELEAAEQAWGNMTTIKRPTTLDLPMAMRYRHLKQNNYNKGTIAVFRSGIHGRGLYCTRHIAAGEMVIEYSGMLIRSTLTDKREAYYESKGIGCYMFRIDGTYVVDATTSGNAARFINHSCEPNCYSRVVTIDGNKKILIFASKSISRGEELTYDYKFPLEDEKLPCHCKSKRCRKYLN*

>jgi|Nemve1|139248|e_gw.467.13.1

LLMCDKCQRGYHVDCLGPSYPVVPEGSEDTWICGRCAQCKLCGSKSAGEDPEAVWMHEFTHCYDCGTAWDNGNYCPICEKCYSDNDFDSKMMHCNDCQHWVHASCQNINPDEYECLSDLPDSIPFVCKLCCQ

>jgi|Nemve1|139250|e_gw.467.4.1

GANIRFVITNDDGLRIEADSHKVAWRMIFDLVRDSRSSHLLKDLPFIGVSDRCMIGVTDEPVTYILEQLADAHLLRNYRFQYNPPFGTNGEDGLELKENPTGCCRTEPHHRRLMYDMFAFLASEYRTPPQLDDNYDELEAAEQAWGNMTTIKRPTTLDLPMAMRYRHLKQNNYNKGTIAVFRSGIHGRGLYCTRHIAAGEMVIEYSGMLIRSTLTDKREAYYESKGIGCYMFRIDGTYVVDATTSGNAARFINHSCEPNCYSRVVTIDGNKKILIFASKSISRGEELTYDYKFPLEDEKLPCHCKSKRCRKYLN*

>jgi|Nemve1|155903|e_gw.9332.2.1

VDATKESGRLGRLLNHSVHGNCTTKLISIKGNPYLILVTSQDIKPGEELLYDYGERSKDIIESHPWLKA*

>jgi|Nemve1|169962|estExt_gwp.C_1370059

MVPSEPADMGKEAMDDVPLPQPMVHRRGPGRPPGRGRKKGAVVIGLPLRNSSQNMTPDKYTFGTVKEHEVHEAFESDGSVWAHHCCASWSEGVCQTDSYDLVNVDKAVYQAMTERCAHCSRFGASVVCQVPRCGRTYHYPCAASAGAFQEIHSMTMLCPDHLEDAGRLGGAEAQCYLCGEAKEIAEMLFCTSCGRHYHGRCLDPAVEITSLVRMGWQCPDCKVCQGCRQPGDDNKMLVCDVCDRGYHTFCLDPPMTTIPKTGWKC

>jgi|Nemve1|183768|estExt_GenewiseH_1.C_510132

MFCGDCNSAYHPDCIGLSTAPKGKFKCDECTSGVHSCFVCRQTGDVKACSQPLCSKFYHKECLQSYKCSKIDGDRIYCPLHFCSTCISNKTPVNRGRLTKCIRCPTAYHAGCLVAGCMPITSHLMVCAKHFLPNKSKAHHTHVNVNWCFVCSIGGTLICCESCPAAFHPECISYEGIPEGRFYCKDCVEGKSLLYGDIVWVKLGMYRWWPAMICNPRDVPTNIQSMRHQPGEFPVMFLGSHDFYWIHKGRVFSYQDGDKGTESGNNKYLAKVFKKALVEAKEKYDEWKKAREDKAEQDLQRFCKKPPQYKHIKTNKCTTAQRIILDPSEMPVCECTPDQACGQDANCLNLMLQFECVASRCPAGDKCQNQRFQKRQYVDCEPFRAHSRGWGLRSKQAIKKGTFVIEYVGELIDDATCRERVKKGDDDTNYYMLTIDKDCIIDAGPMGNLSRFMNHSCYPNCETQKWTVNGEVRVGLFTSRDVESQEELTFDYCLDCHGNEKKKCHCGSQNCSGFLGVRPKTQNAQMNEDKAKNAALKRRKRKPKKPAVKQVHEDDCFICGDGGQLIMCDRSGCLKCYHVDCLNLDKKPQGRWQCPWHFCDNCGKRATVLCSECPNSFCRSHAQGQINSTDKNTFLCTDHVDEVLPGMTDKFLATDQVLNALDLINGDLTGIRPTELDHLQQKPEYLELGS*

>jgi|Nemve1|194976|estExt_GenewiseH_1.C_4670002

MIFDLVRDSRSSHLLKDLPFIGVSDRCMIGVTDEPVTYILEQLADAHLLRNYRFQYNPPFGTNGEDGLELKENPTGCCRTEPHHRRLMYDMFAFLASEYRTPPQLDDNYDELEAAEQAWGNMTTIKRPTTLDLPMAMRYRHLKQNNYNKGTIAVFRSGIHGRGLYCTRHIAAGEMVIEYSGMLIRSTLTDKREAYYESKGIGCYMFRIDGTYVVDATTSGNAARFINHSCEPNCYSRVVTIDGNKKILIFASKSISRGEELTYDYKFPLEDEKLPCHCKSKRCRKYLN*

>jgi|Nemve1|194978|estExt_GenewiseH_1.C_4670007

MFFCKVCSEPFHGFCLDEEPIDEDSWCCDSCSTCVVCGQQDKLLMCDKCQRGYHVDCLGPSYPVVPEGSEDTWICGRCAQCKLCGSKSAGEDPEAVWMHEFTHCYDCGTAWDNGNYCPICEKCYSDNDFDSKMMHCNDCQHWVHASCQNINPDEYECLSDLPDSIPFVCKLCCQEENPKWLQEMQEEMQAGYQRVIDEVESSRGYQPLIGYFSQVEFENGTPKDLDAVRAKVKENAYRVVGDFCHDMLLILNKVCKITGNQPLVRNAAVLYEVFIKEVGAMFPWHKIKSVQDSTTTETVKPEEDIKQDVVPAPVTLKPCNGVVGEPIDMMGWDHLYSAAPLLEQLQKQQDSQEKAFEQEQEQKLEQNQEKQENGNKKTDNKGNNEAEMDVDQHKEDPCSEEKLNSTIQVQTSEPTEAKNESPSQQPINGKDSPKQGNIKSSGVTVARKSNKRNSKIIPKKPVQEITNGDQVTDTRKCQLCNRLGDDEPTRAGRLLYSALDEWVHINCGLWSAEVFEDDEGRLQNVQAAVTRGKMMKCELCGEAGATVGCCENRCPMNYHFMCGRDAEAVYQDDKKVYCAQHSFRAKEDLVLKDKQFHVERRVCVDMSRVRTKGAGLKGAEPHAIQLMKGMLILV

>jgi|Nemve1|195312|estExt_GenewiseH_1.C_5680002

METEMIKAVRLEYIRLREKRQMKRKESAKRDFIANRQKIREQLRKRKQKIENVTSCPIVGSALLTEQMQRPARKCTASSGFHHSTQHTLLRTMCAVTPIPNFFTWSPLQQNFVVEDETVLHNIPYMGEDVIEKDNSFIEELIKNYDGKVHNTRMLDDYTLTDDLLVELIDGVLATSSNVAKEPDPQETKGTETKDGQSGATDSQAVVPSDALFEAIIKLFPEKSWKLVDIKSRYNDAKTRIEGHAPPECTPNIDSPEAQSVSREQSLHSFHMLFCRRCYKYDCFLHGWRSFPSQAKRKSPVDLQESSPCGPDCWLHIKNVAVSPSGSPSKSESSSRKRSKGRKSSQHSQGFPSPDSASPTKKSADELLSQQSEWSGAEASLLRVLRTVYFNNYCTIAKLIETKTCKEVYFRAFGESEESLPVVDDTNTPPRKRKRKHRMWSLHCRKIQLKKDSTSTHVYNYIPCDHPGLPCDQSCLCVMTQNFCEKFCQCNSDCQNRFPGCRCKAQCNTKQCPCFLAVRECDPDLCGTCGADNFDQDSKTCKNVSLQRGQRKHMLLAPSDVAGWGIYIKQSVKKNEFISEYCGEVISQDEADRRGKVYDKYMCSFLFNLNNDFVVDATRKGNKIRFANHSISPNCYAKVMMVNGDHRIGIFAKRDIEAGEELFFDYRYSATDALKFVGIERDVDFALR*

>jgi|Nemve1|196738|fgenesh1_pg.scaffold_3000059

MRGYEEGKGSSCTLRVHGMPVLFFFLPRVLFVSTGRDTACVKVLRDLEVGDEITCFYGEDFFGDDNCNCECVTCERRGEGTFKSKQKENVKKQKYSLRETDKRLKRLNIMKGVPLLDVTGQATCPASMHHTEVYLVVRLQQNSLSKRARKSSVPQSGAMDLVTSRESTRHIEDPRIDDANNTVKPNTDNKV*

>jgi|Nemve1|198875|fgenesh1_pg.scaffold_12000098

MDRVYYGSLNCCGAKTKIESLDLQVDRLTLYGSLWRAYKYWESGSQEGEAVTRIVKDKGVAHMPSGLPPKIIEHNEPYLEGMAKSQRKPKQMRVQRKGNNEAEMDVDQYKEDPCSEEKLNSTIQVQTSEPTEAKNESPSQQPINGKDSPKQGNIKSSGVTVARKSNKRNSKIIPKKPVQEITNGDQVTDTRKCQLCNRLGDDEPTRAGRLLYSALDEWVHINCGLWSAEVFEDDEGRLQNVQAAVTRGKMMKSHRKTNFPSKSVSGLSGLLDAVGELFYPISCLADIFEKCELCGEAGATVGCCENRCPMNYHFMCGRDAEAVYQDDKKVYCAQHSFRAKEDLVLKDKQFHVERRVCVDMSRVRTKGAGLKGAEPHAIQLMKGCIACWKNMRKVNNQLYPFATALCSIRESQIDRNMRKQVKGPPNFTAT*

>jgi|Nemve1|204976|fgenesh1_pg.scaffold_55000083

MVLNTPEGLKPGISRARQLAESDDIASSLVVDSVLGFTTHKMAARFRPLKIDATVVKRALLRFLNDGDVDKAYDEIVFNAGDWGRCYFLNKSKNQIAAFKDHMLRYIGIFHRDAGFKIHKCNRYSGESNGAKVVSTGHWAKGDKLPNLCGCIAEMSEEEEKALLRAGENDFSIMFSTRKNLSQLWLGPAAYINHDCRPNCKFVSTGRDTACVKVLRDLEVGDEITCFYGEDFFGDDNCNCECVTCER*

>jgi|Nemve1|214071|fgenesh1_pg.scaffold_191000035

MAPKPSVFEVFETESKGRGLRAAKPLKSGDTILSEQPVVYMLSNMLRGQRCDFCLEKLSDLQRCSRCKFARYCGASCQRAAWRIHKSECERLKRVFPRVPTDLVLLMFRVWQLKSQNGWYDSLVSNVEKIDSDAKEDFVSVLMVLNEYLGSEISPPEGLELFSKISCNSFAICDGEMQAIGTGIFPNAVCLNHSCAPNSVAVFNGTNIYIKALEEIPVGEELTISYIQQLHPRETRQEELQTQFCFYCQCHRCLDASDNNKMLTSLICPNKSCEAIVYQTFDACVMEQDWTKALEYATRNLEVYTWFYPKYHPCLGVHLYKIGKLLAVTHQDLELAVKRLEQARRILEVTHGQSHPLVQELCEYLCQASEELRQGRI*

>jgi|Nemve1|214433|fgenesh1_pg.scaffold_200000015

MNIDTLSDTIAQGFIAKVHELLANKSSCKSKELSLRWCLAGDLAFTKSQFHRALSYYSQAIQYFPHQTGDQQSTLQICLPSILSKRAEVLFLVGEYEASLNDIAEASSLQYNHVIRDKLLKLQKSCLRKQARHSQNKNGSSGSACLSGGRHPKMANGSSLLKINYDQNQGRFLQASSEIRAGDTLIAEEPYSAVLLPENAKTHCECCYKSLVAPVPCNHCSSVLYCSAACRNKAWSQYHHVECEIFPVLEIVDTFTHLSLRILLTTSAKDIIDVLNGLSRDVATTSCSLPGCTVSGSYPGDYGSVFSLVTNSDLQPIKALMSFAMNSAFLVEFLENGTSSACIHCSQIKSDKTKVQTELDSDDDSDCSEVYNACEEQRTQNGNFEQDRTICSRNTPYSRQAYTSLGITTEEFCGKDGLSSDVVGALLVHHLQQMPCNVHAITAIVSTSSSDEEDEEMGSSHDQVVAREQRRIASAIYPTASLLNHACDPDVLVSFVDGVLVARATHNIAPGSGITHCYGPHVNHMPREERQKLLYKQYFFTCQCSACTSDEEMENTRLCFSAFACPRCKCPMKTSPLEPSLARCQNKKCTLEKSIEEELSHSRQAELLFFKAVRTMERIGVQEALGLFQECLRTRTQILHPHHKDLAETHDALARCYAMIGDFKLASQHCLQSSEAVEKAFGSTSVEYAHELHKLSQLLFNDRQAKKALPMIDKAASLLATYYGRNHPDVQELVEMKACLTSNGSVHINY*

>jgi|Nemve1|218071|fgenesh1_pg.scaffold_327000013

MAVVRELQRKHEQELRQVRDRTRRETITQFVRDHSDYMRCVEDDIAVQPDQREKTCVPARNDVTEDDEQKEPICHAQMSLVNRLHDFRLVMSLPVLEGQGSREQPREAECRGDNQSEDTAAGKTEDAKEQEEISQDVRALVNMNESEEPGDDIDQSVEGLNAISQLEEAEYEISQSEEAVAAIRQSEEAEKGISHSEDVDEWIGHSNEVEDEVSKTLMDNCTATENDRISNDVHVFIPVTSLSPDSAEVLLDAMQNFGYQPEITEEQSDGMRDPGNQQESTEEQSDGMRNPGNKPESAEEQSDGMRNPGNQPESNEEQSDGMRNPGNQPEITEAAERKQIADAPNGLVLDCSWCKAHGFGMMACSGCMSKFYCNEICQERDWSLGHQFDCQYYSVD*

>jgi|Nemve1|220581|fgenesh1_pg.scaffold_543000004

MDSPSLKVICNQASFKSLTVVKEVKKGEFLCDLWGVVSPVGMHTVQVDKEKHILPASNLSYTNHSCDPNAEFVFKPRNGITVPKGKELSWYLVARREIKQGEEVTFDYTLTEYDMNDPFECKCGADDCLGTVRGFKYLNNKQQQERLAHISPAIKKIVNN*

>jgi|Nemve1|22303|gw.44.14.1

CLFFLNGYSIILLSSRKKRINKEVRKMTCECYPEPDNPDFVGCGEDCLNRLLMIECNHRCPCGDLCTNRRFQEGCKIKVEVFKTEKKGWGVKTLEDLEQNQFVIEYCGEVMNYRDFQSRAQRYDRQKRRHYYFMTLRADEIIDATLKGSISRFINHSCEPNCVTQKWTVNGLLRIGFFTLRTIKAGEELTFDYQLQRYGKIAQTCYCESPSCRGIIGGEKHTPLKTTVEKIDELERLIGDHRGMTVSDQALKLSRLMVRAEDMPQRIMLLRVLQNTTDQSCLKAFLRYQGLSLLWSWMVDAGAKPSSKLQLELLATLKYLPVSSKNQLEDSKVMRVVNKWAIAVEEVP

>jgi|Nemve1|245129|estExt_fgenesh1_pg.C_1460039

MYKRDFSKLDKEALLSDIQSINWIDVLPSNDGSKNVRKKQLKFAKSSIHDWGLFALEPIAADEMVIEYVGKVIRQAIADYSELFTKTSENNPYELKLRDSGHNVEIPLIPKLNIAKPSAEQKQPPYDASSLLKLNNKPCPFTPSQSNLPRYLPSTVTPALKNNRLLFNTLTSLPSQKAISANVVFRDAIVWQQSHGVRDPRDSLQTPDANTVFRVASVSKVLTALFVLILLERGHVTSIDDPLEKYEPRFQVSNPFNAQKITLRNILSHTSGLPREAPCGPGWSRSPRYLCAVNTSYVLDWLRTTELKSPPGAEPSYRNILSHTSGLPREAPCGSGWSRSPRYLCAVNTSYVLDWLRTTELKSPPGAEPSYSNLGYALLARVLGQRFAADEYESWVTQNILRPLRMINTGFYLKDMSANRAVTYNNDVINSDIDWGWVAPALQLYSSAADLSKLMMALFGDLSPSLVRKDLLQQLLTPSFVYPDGQTMFGLGWEMKISGAYLVVSKAGIAPGYSAGFLLLPQLKLGATVLMSGHDAAPAAAQLLVKPLAQRLAVYMIDQMKVQVPVNPTKYTGSYRLISKWVSKVHFNITYKDNVLELHQDPGYPKDSFLSYVDHQVLELVYKPGYPCSVYGMGQNHERLVFRSPLGKDSKCSGFTFGPFVFERISVDSEQTNKNVKSRSLWEMV*

>jgi|Nemve1|245520|estExt_fgenesh1_pg.C_1640033

MALYRNQCWHCCVFTEGGPKCLRCNIAEYCSAQCRDDNQWRHKVDCDNVSRQLYCHKCSQKGGNMKQCSNCLSAWYCSKECQGEAWGSHKKRCTKMAKKTVQVWEQLKCLNIPGVSFTYSLSRLYYWGNSPAIDLLKLRLNEGEEVDGPLSLLLCGAGDGRHVFLTLAELPRTYKGQVTFVLNDIEPCVLARTVLMLYLTVKGGASKASLVTQIWYSLWLSASELEFLVSALQDLTRISCLEVISHGVLCIKNEHLKTLQSIWKVWLLLVGEHIDLENQRKGVVHEPSFYLADVLDKHKESLKAWFKHGVLCQDGSDAIKMHENVTLTGFKILEVSQMVKQDFKYAVPPQSYPFTGWDYKDAHQNFPEIDSVQQLYSRYISLVLQKSAQISQDAEKVRIKFVLGDFRDLPQVSQEGVKFDRIHMSNLWDYGDLSEVLSLAKHFLNPKNPSATVVTETFNWVKFFPRLMADAALNSFTKPKQNKEGGTDPEDDQLMMGATALHIKENEHYFLTGRLSDINFLSPRVLFSEHYNIMEHFLRYLRASLLSAEVMPPRFLPSLKKLAEDKGFVLRDPTRFENRVAPFRWGASCRLLSCTRGSELSLEWTVKQ*

>jgi|Nemve1|247208|estExt_fgenesh1_pg.C_2780032

MAGFVSYHEFITNNLRMGKPGRTQRKRLRQKRMNISGSNLKPQVLLEENYISLLKWAKRNGMVFKKIRPAIFSSTGRGMLAIERIHSSECVISVPERLLITASSVLESAIGNYVAERMKGGAKSSNDYLLVLFLMYEKYLEKGSFWAPYIRTLPDTFNTPCYFTRKELFLLPEQCREQAFEQVTQIKQSYKSFAKAYNDVLQDFDCNFWRTVDFESFKWAWCVVNTRSVYHDEPNRRAQPIDGNCALAPLLDLLNHCDKAEMCGRFNSSSKNYEINVITEYQKGTQVFINYGPHDNTRLFLEYGFVLPRNVHNSYRFTRSTILSHLGMSNSFLSNAKEELIERNSLTRDLSCVSSEGISWNLWTFLGVLALPEAIVSQWDMILSNKCPDKLKGKIEMWARLLLQRELSDLVTYNSGLQDIDPSDNSRLALHLRQDQIDILKESLLLL*

>jgi|Nemve1|248598|estExt_fgenesh1_pg.C_7280005

MDSDAIDIVTDNTSVPPSKSLKAVKKIKKGDFICDLWGNVNPIDRHTVQVSKDKHIMPEGKVKFTNHACSPTAEFEFKARDGVVAPEGKELSWHLVATRDINKGEEITFNYTLTEYEMDAPFECQCGAKQCLGRVQGFKHLTTEQQQEIMAKVSPAIKEIIKN*

LVTTEDLNKNQFLIKCKGRMMLGSNFEKENQFFKRYSPHVLFYDKLDHLCLCVDARTFGNDARFVRRSCSPNAEVRHFFSNGKLCLALFSLCPLPKGAEITIPFEHSLHEYKSHLTCACSQDTCAVVK

>jgi|Nemve1|33659|gw.32.106.1

QELDNYIDKMIEKNKNYKYKDRLSEETWEEEIENIPLFMTKPPEEGKSISDSIAALQAIKYEDENPVENALSYKEEGNYEYKRKNFKKAIDAYTEGIKLRCQDGHVNAILYTNRATVNFSLGNNRSAWNDAKTARKFEPKYMKAIARGAAATMEMKMYEETIKWLIQQNLCKFSLITTNIEPDNKTLLKLRTDAASEQKKIERDKRKAKAEKKKEAKEIDAVLKAIEERKINIEKQKSGKNKEDEDEDNVLINKFEALGLQSFHPSGARVQLDENKKLYWPVMFYYPEYKESDFIGAFYEEHCFLDHFKVMFTDEVASWDDEAKYEPYALEVYFDNPSNKHLCFVESSTALKDVLVDPRFLLRQGTPSFIILVKGSSFRDDFLKQYTVEK

>jgi|Nemve1|4240|gw.414.7.1

LLMCDKCQRGYHVDCLGPSYPVVPEGSEDTWICGRCAQCKLCGSKSAGEDPEAVWMHEFTHCYDCGTAWDNGNYCPICEKCYSDNDFDSKMMHCNDCQHWVHASCQNINPDEYECLSDLPDSIPFVCKLCCQ

>jgi|Nemve1|53017|gw.169.94.1

KLEEWEKWLNDCAVNEPYISVENKVDNTPPPTDFVYISQNKVPSFLDHLFDHNYLVGCNCQRCTPKSCECPKNSGGVFAYDRFGRVQFEPGKPIYECNSKCSCSESCRNRVVQRGRTVRVTIFRTYNGCGWGVKTMDPIMKNQFVTEYVGEVITNEEAEHRGRHYDAAGQTYLFDLDYNDGDCAYTIDAKKYGNISHFINHSCDPNLSVFGVWVDTLDPQMPRIAFFARRDIPAGEEITFDYLMT

>jgi|Nemve1|80162|e_gw.2.228.1

YITECIAGPGACIDIGKPFYPGCCCEECLVEECSCLVKYGSPYHKQDGKTLLTRTQHDGISQPIFECNSQCNCDLSCYTKLVQKLIQTRLEVFKSKHKLWGLRTLEHISQGQFICEYAGEVLSYKEAKKRTIEGKGRPNYIITVKEHISGGKILRTHVDPRIYGNAGRFINHSCDPNLVMVPVRVDSLIPKLALFASKDIFPNEELSFDYSGGRCGLPSSSCADDPALCLPCYCNSSNCTGFLPYEASLF*

>jgi|Nemve1|94595|e_gw.37.139.1

SARTEGYYKIDMKEKAKYAENIRRQVQRDEKLDASKGKQQSRENRAMQRRLQLAVCNEDFGDILKFNKLRVRKKQLKFAKSSIHDWGLFALEPIAADEMVIEYVGEVIRQAIADYRERCYEERGIGSSYMFRLDETTIIDATTMGNFARFINHCCDPNCYAKVIAVENMKKIVIYSKRDIQVDEEITYDYKFPIEDEKIPCLCGAPQCRGTLN*

>jgi|Nemve1|96894|e_gw.44.58.1

KPRALPPNWKTATDPQGKIYYYHTLTRRTQWDPPTPEIVVSPRKFTTDMEMDSGDEEPPLKKKKPKTPSTPPGAKQKIKDAFRVKLSNVVVNCLNPYHKVDCKQGRITNVEDFKYLARKLTHGVMMKELQHVKSAESLQCNDSVKVKTKDYIRSYMKKFGPVYQRT*

>jgi|Nemve1|104327|e_gw.72.27.1

MIPRGTRFGPYTGRIVFPDDLRTDLDNRHMWEVFAHEKVSYYIDGQDEQNNWMKFINCANSNTEQNMSVIQQGSDIYYEACADIYRGAELLVWYGSNYELYMGIPLSLKTEGKESVVKNKSNDENNGFACERCGKVFAYEYYRDKHLKYTRCIDNGDRKFPCTLCDRSFEKRDRLRIHVLHVHHKHRPHQCSVCSKRFSQSSSLNKHMRVHSGERPYKCPYCEKAFTASSILRTHVRQHSGEKPFKCKHCGKAFASHAAHDSHVRRTHTKEKPCVCHFCGKAFAQSYELKFHINMH

>jgi|Nemve1|193459|estExt_GenewiseH_1.C_2950019_jgi|Nemve1|197426|fgenesh1_pg.scaffold_5000232

MIATLLQLIFVLDADEEKVWPKMKEPKDAETQTDPEITGVDADDFEEGDYSSSGEESGSDSDSSDEEAAGTDKSKENASSSVKTSKGVGAGQVRRKNIELKYYFGEDMPDAEMQRLAEADVLVPPGLEVKESNIPNAGRGVFAKCIIPKHEFFGPYIGRIVTRKEAKTYKESPYVWEVFDDYGKLTHLIDGRDALQSNWLRYVNCSKGLDEQNLRAVQYDKNIYYMATKEIAIGEELLTYYGDKFAKKIGIKPKKSSVVPGEESFVCKNCGKMYTNPGALIGHLKFKCNTVRNYALRYPCLPFKDQMTEVYFFPLPCPLTYHNALDIAMRCATTAISQNVNLVQRGTKLYYETSRDVLQGEELLVWYGEGHVTMMGLPIGLAEKAKDATQNGPSFPCERCGKLFAYEYYRDKHLKYTRCLDNGDRRFPCPLCDRSFDKRDRLRIHVLHVHEKHRPHECHVCQKRFSQSSSLNKHMRVHSGERPYKCTFCDKAFTASSILRTHIRQHSGEKPFKCKYCGRAFASHAAHDSHVRRTHTRDQTWTCEVCGQVFTDMTQLHYHRNTH

>jgi|Nemve1|61034|gw.22.245.1

LPQGLRLISAAMGTASQYAVMCSQKVISRGMRFGPYRGRVVQLSQVNEREDNAFMWEVFQNGQFSHFIDGSTESENWMKFVNCARHRDEQNLRLVQDGGDLYYDACRDIIRGEELLVWYGPWYELYMGILVGMKTVQPSRKTGHKKLISEGAPGNYSCERCGKVFAYKYYRERHLKYTRCVDQGDRRFPCEQCDRSFDKRDRLRIHVLHVHEKHRPHECHVCKKRFSQSSSLNKHMRVHSGERPYKCTYCDKAFTASSILRTHIRQHSGEKPFKCKHCGRAFASHAAHDSHVRRTHTKEKPCICEYCGKAFAQSYELKFHINMHTG

>jgi|Nemve1|96522|e_gw.43.248.1

QIFQDGELVQYIDGSQDSTNWMKYVKCARHDGEQNLALAQDGNELYYDVTKDIYEGTELLVWYGDRYLKYMGIPITMKTKIAEVDSSINGVSNYTCDRCGKVFAYKYYRDKHLKYTRCVDQGDRKFPCHLCNRSFEKRDRLRIHILHVHEKHRPHQCNECGKRFSQSSSLNKHLRVHSGERPYKCPYCSKAFTASSILRTHIRQHSGEKPFKCRHCGKAFASHAAHDSHVRRTHTKEKPCVCEFCGKAFAQSYELKFHLNMHTG

>jgi|Nemve1|112691|e_gw.113.70.1_jgi|Nemve1|112757|e_gw.113.50.1

VPYALRRIPDEVRLCKSNIPGAGYGIQANTVIPAGAWIGPYEGNMVKAEDAPKEKNFYMWEIFKDGRLYGFIDGSDHNTASWMRFIRCARNKEEQNLFAFQYLGKIYYRTYRTIPMGEELLVWYDDKYTQYMEMPFAPTAELRAHAVVHQGRKPFKCGYCSRAFSGATTLNNHIRTHTGEKPFMCEACGKTFSQASQLSKHQKIPGDCVSP*

>jgi|Nemve1|200651|fgenesh1_pg.scaffold_23000016

MASSSSDTEFQDSPGRDQISWEDLYSYLYGHVTLKPLEPLDLKAIRCKRRRDSESSIDGTVPVKSPRSSPPLAAFVFPDEVRLCKSSIPGAKFGVCAAHPIPPGTWIGPFEGQIVTREEVIKRELDTSYMWEWYSLHSKPQWKLFEIYKEGRFSHYIDGKDEHLSSWMRFIQCARYQEEQNMTVFQYCGNIYYRAYKHIPKGRELRVWYDDKYSELIDIPALLKGQKDGGVYWKSPSNGHVRDNELFYHRENRPVSTTICQRIGTMATFTNRYNGHLYQSVQWAPLPIGTMGTFTNQYNGHLYQSVQWAPLPIGTMSTFTNRYNGHLYQSVQWAPLPIGTMGTFTNRYNGHLYQSVQWAPLPIGTMGTFTNRYNGHLYQSVQWPHLP*

>jgi|Nemve1|54602|gw.60.181.1_jgi|Nemve1|90179|e_gw.23.187.1

FPSEVQLCTSSIPGWGYGVCAMQPIPQGTWVGPFEGKRILPRDIPLDADTSMMWEIFHEDEVVCYLDASNENESSWMRFIRCARYRGEQNLDLMQYHGNVYYRAFKDIEPGEELLVWYSNESPQYMGLPIWRCGQCSQTFSQRVLLQMHICSQAPDKPYQCGHCPASFQEASDLRDHVVSHINEKPFKCGFCGRSFAGATTLNNHIRTHTGEKPFKCLKCNKQFTQSTQLSRHQKSPEECVGGKISS*

>jgi|Nemve1|84066|e_gw.9.119.1

MHGPLHSLRKLVTAGQSSSPLENGSVLKFPDEVCLCTSSIPCVRYGVCARKRIPAGTWIGPYEGKLMRPEDIGSDTDTEYMWEVFHDGQVSHYLDGREEANASWMRFVRCARHKKEQNMVVFQYHGCVYYRTVRDILAGQELLVWYDARYSQFMGVPVALSDSGTRAAYNGKYSGRTSRDMADDFTWRCNLCFKAFAVREQLEEHQCNGMGKNLVCHHCNQTFSHPVEFRNHVESHANERPFRCGFCSSAFASAALLNQH

>jgi|Nemve1|113856|e_gw.120.9.1

SYREAEVPDDDHYLFCEECQDLHYGECPIHGPLQAIADNATTSSTQTTARATLPHCLEIKTSSIPNAGLGVFSKSRIAKRVMFGPYKGTKVKLSDEQMENDTSYMWEISRDGNFNHFIDGHDEEQSNWMRFVNCSRCEKEQNLVTFQYRGQIFYRSYKDVHPGTELLVWYGDKYANDLGIALEDVEDSNIIASELCYTKDYTCKKCSRCFTSSNALLWHRKRCYIEPTGDELKCKLCQQQCATPKTLTGHIVSEHCENSSRVCPICEKDFCDYRYMIMHILTHTKYKPYKCTQCGKAFAHASSLTEHMRTHSGEKPHKCTQCGKAFARASVLTRHMRTHSGEKPHKCTQCGKVFAHAGNLTTHMRTHSGEKPHKCTQCGKVFAQAGILTTHMRTHSGEKPHKCTQCGKAFAQASNLTTHMRTHSGEKPHKCTQCGKVFAHAGNLTTHMRTHSGEKPHKCTQCGKVFAQAGNLTTHMRTHSGEKPHKCTQCGKAFAHASNLTKHMRIHSGEKPYKCTQCGKVFAHTSHLTKHMRTHSREKH*

>jgi|Nemve1|119550|e_gw.162.3.1

MAFVSLPEQVTIAPSSIPGVQLGVFSTCWIKEGTQMGPYTGRIVKPDQVNYEIDNNLMWEVLNEDGSVSHFLDAKEENPRNWMGFVNCARNEQEQNLEVFQYGGNIYYRAIKDVPPDQELLVWYGGTYMQFLGIPGIPPVNDYRRRRRGQNAEDTQSPIPRNVVPGRLKCTLCRRGFNSRSNLRSHMRIHTMEKPFQCKFCKKSFSQSSTLRNHTRLHTGEKPYKCTVCHFAYSQLAGLRAHQKSARHRP

>jgi|Nemve1|41788|gw.113.66.1

FPPGLQLCTSSLPGHVFGVRATTGIPAGFIMGPYEGNKVKPADVNTTADSSYMWEIFENGVLSFFVDASDEFTSNWMRFVRSARNSTEQNLSAIQFQQAVYYRTLRRIVPGEELLVWYDKSYPQYLGIPLATTTLNNHIRMHTRQKPYSCQKCGVTFTQAACHARHVRNSR

>jgi|Nemve1|40616|gw.114.81.1

CDFCGKVYCRKYVLKIHMRTHTGFKPLKCKFCDKSFSDPSNMKKHVKLHETENTVHKCKHCGRSFVRYRGLLNHIK

>jgi|Nemve1|66221|gw.19.270.1

YLCELCGKLYTRKYGLKIHMRIHTGYKPLKCKYCQKRFGDPSNMAKHIRLHAVGDTPYKCQYCGKVLVRRRDLDRHIKSRHP

***Oxytricha trifallax*  (Ciliata)**

>Contig10682.0.g70|TPR repeat

MQKLKQNSITLPQVQSSPINQNKSPQELLSGMLDAMIPTGMFGPMANKEMLMQRFTNLRTQMIENFGSANDPKIVAQNYMQKYMSFMYSGEEQLELQQVQAKQFTIPKLQIKPIELLQYSKITPQELTIEKVHYNYYLELDIKINCFRGQAYHFLAGEIEDPSDQNYEDINQSDQDERLKSVLACSVYKMENIYDIRYFQAGRKFVVLDPYFRFGNDCMAFVKVEDRDKLILLENNQTIDDIIEDALKTQTALDLKNSGNKEFGAGKFQGAINLYTYGITKAKSAKDQQLLGVLFGNRSQSFFNVRQYEKCLKDCEEALKLDPDNKKFKFRRAKVLGFLNREEEALQQLQLLDPTQQDKDIQEGIVQVQERLNQSKGMYNLSKLIEQSKKLKSITDIEVKEFIGPIEIGLIEGKNRGIIAQADIKKGQIILVEKAFSSNEQRQELRFELEQTFVYLNHHENIPLLRNTQCQMLNDSQSAKWVKYLFNGTNGELKVSIQELSKKHKLPKSQQRITYQEMDKMIKFNQCECMPLQSIFKKLKSLDKIVDKQAMGKLSALWPIFSFINHECNANTTRFSIGDALFIVALRNINQGEEITQLYMPLASAFEERENLMQKSWGFKCTCISCQRYISLPEDIKQLLKCAFIQPQNDKERKQMKNELLMSLPIVTSKLTELNLESTYLSDYYQMLVECLAIFGLQNDEKSFLKYWENFKQFGDLQEVSKLKQAALMSFGVQSQAYKAVSQLQKELFMIEFQNDQELFEKYG

>Contig10990.0.g25|SET domain

MQLDQLFQKFNQDNLEKQKLDKFVKIDADLQNLKNYYLNSNISCYLQQNFIDNLTNTQIQNEEFHRDVTLEPKLTSFQYNQIQATSQSTEIYNVKATPRNFCKPFYLNSDNDYIKFTDSGQMNWEYFGEQNAEKVYQKIKSKLLQSNIPINQSFSRFERQLKLYDHIQNQGLSNSEDLNFSQLPQRYQYLYEIDLSSSRNQIENDISVVQFDEALFQKDLVELRNKLKVQQDQNQSLRNYEAKFNAVKSLICKLCKNIACQKHILDLHNIPESICSSKFDQDMSIYEQVKEKLMRLELQKFLTSRDGDNRKQQCKSSECFYLTLDQTLQSCDQNYIDLKQFLRISLIHDFNPCKMFRYLQIFKQKTANFQQLDCCTKIGNSLLQNKDKLLAISSEIQNQPSIINLNLNHNLQQSSQIERQSQDGEDNEEMFKGCECEPGQCKESVNCSCLREGNFCDITTCKNCFDPKLQQDFKQSGQKFVDSLQYLRKFSQNSKEDTTLQQLCINTIDFFSIQNMKLGLSDSQIPNSGLGIFALAFIHKNQQIGIYTGEIIDQQNDNFQYDKRTFFKEFDAPSYIYDIKKDGFRDAINIGSLMRYMNHYDMKSKKVNVEVIIRKYQGASHIVVFRAKQDIQVGEELYINYGNDYFKKLEKK

>Contig144.1.g7|SET domain

MENDSDFYDEYSQETEINQHNSGMINAVNQVKRAHNSYSFPFNEAFELKREKLRQQELKNHPERFRLLAHSLSQFIPIKKNKYLERKRYVLNEKEASSLLVCNCQKATYKKNEKTNPLQSFNCGERCINRCVSTECCAQTCPSGAFCKNRRFQLHQNAYVFPAKTEKKGYGLFAGEFIPKGTFIMQYVGEVFSVDTDLGQQRVLEYRKSTCTYLMRTTNNEVIDPTYVGNVARFINHSCEPNCETQKWNVLGEVCVGIFSLRDIHENEELSFDYQFDFFKTPFTKCYCGTSKCKGYLGVLSKITDDEDEEFENPTCNICDDYINDKDDLLVCNGACRETFHLLCIIKKEGKTKTQAQALMQQSDRRNFVCRDCIVNSRKKKNRKNAKDLQMQDEQNINTSLLRNNQDAGFILENPQEDKDDVINLEVEVPPWESEDPQIKKFFEDNPQNRDLDIIIAREAEQIEQNLKSINEAAQTGGTGNNKFKRAKKKNSQFSEHYNFISELQKRLKMRIDGDKNNYKFRKFKDDIQEMKRMKQQLIQAKKQQRLLKQMALAQKQQQQALKNQQQLQIAQTINLEHKSQSVSQQFIGVIQQEEISGKKKRGRPRKIQSSLSFNKVNSHLQQQTSTSLLQITQSSQFRHQSTNLQHPQLHQSMSVQITDTQTTQLLLQSSSADPLNLQLSQPINEQQELHKEDESEELRELIDTQMDHQQVQRRQRLRKLEEEDVIMEESREVNELSQSKAEVQEEDMDDDCEDEENDEEDDNSNTLFEEVDENEEDFIDESVAAQALVDEDDDEEDDDDDDEDTFSNQGDEDNMDSNNRDDELDLDKLQGQYEDQEEQKVSFSANQLNSQRRLRLQSGNSLNEVYNNSRTPIKNRNLNDKNISPNKKDTLMQIGSTSQQDQHSLNANNHLSLVQREKQRILTLINTKINEFLKEQIMASSDTSLAPINPSELIVRESKFNIFEAVFNIVISENIDTLQCLPTKFQIFWEQAWGGMVTVRLQSTLKQYQFLTNLISEVTDLMYKAKLFAQSSSGLQGINGVQQSFGSSSGHNFSSKSELHFNKSDGKYQVPFEIVMMKVPKNFIRKLVGYQEKVLTEFRKNYQVDFYFDRDLMTDDIFQMNETTELRIFGRNQDVVQVNEMIQRELEQIKIKTVLLQMNDCKLLMDNVKEVKVLCDPCEIRVKRMMREWKDLRHPFYYLPNYFREMALIGKESEIKQAENRIDQFFTAKRDINYVNNQQISFLLPIYFKNLSSEMKVQLNTKCSKVQVFFYEPTYPRKHLTVLMIGPWSALLQAKIVLEELANVYMFRNNHSFENFQQFTYHQQIRFSFKSLKRFVLEKDIKYLNHWDLCSIFMENAGKPVPNFDWSTFLNQSQPSKGNQNQMLMNSSHQDALQPQISNQSFGQSSSQNLLFHHQQVANQQQIELLMKQREDERSKDYSALIDFLKNQDIETCINSIFVLNDSNNAEESLRELMTRLKVSKNELIQFMAIMLNKNIGDFEDSIRSYGARYEPIKIIQQEANDESETLIIQQSRDACRRPKGYYQTSSTFYKKRSKSRSESQVKYISGDHKYNTGFDQKSRGLSHDSSIENKKSKPKFSNFDNEQIMKQEKESSRSRSISRNRSKNMKSSDSNENSSDSSSHGRYKYFSKNYKRNADSPDFTRPIQRFYKRSPTREYSGYHKFRYHGSYKDIVNSTYRKSHHSRESPSKSRSRSYSKHKHHYSKSKFSRHYVKHQSRRRSSSSSSYHKKHSYHHDKTSGRKYYQSFYHGGTHHKYRDVDRASTRQEMNHDSNKRSDSSSSQNEHKSRNHYSHRSKSRETSHHRTSRSRHQNHHTYHKSSLSHHYSKSRRQDYHGFDFRHNFSSGGFDSQNYFSSNGSRDPYHSVRALQDPNRKKNRGGFDYNAANDSQQNKLVPVKSTSENMVQGQPGTSNEEGQI

>Contig14971.0.g38|LNS2 (Lipin/Ned1/Smp2)

MQSICTQIYPQEEFINPTSEETIMYSGMRSHCFVKLQSNYYPIKQSADDRQCYRSLMCFKNLDEVRRLYKDISSGSYESMDEKNVPKEEHMSELNVQLCLQTVKKCQPKSLKDALTYVGWYMKKIKITKQEFKDVMQTLLSTKMYIYRQTGKLQQSYTKDEAPLEQINEHLPLAFCPICMLFLCTKHLDANQETNTWIFQRINQTLKLNFDLNMLKREKSAKHLKEWIFNVENVCDARKNQVPKQSNKKQNTQAKLNQVNNQLEALSNIPYPEKLVNNFDFIIKTFQTLNPCFLKLLYNKFNNKKYKCNQISGIIEHIKGTYQNCVDAEDLLIQPKIKITMQDINRVCDMQFYEQKLKYQTKPQNDGIPHYSPCDHQGACTSDCSCIQSGLLCESWCCCKNYCLNAFPGCSCKGEKDCENKNKCLCLKYNRECDPNKCRGCRSYVTHNANANYQHTQAMLQGKLSVNKVGGQMQQQSLARLCKNVPLTLNFPCKRVFTAKSTLCDEIVGLFTMDRVKKDELIMEYTGKVITQEGQKETVDQLVNDLRGRSYGFTLDRLTTLDAVYVGNLMRFANHSSDKLSNCKINMIFAQGMQRVCLVASRNIDQGEELFFDYGFSKEFEWLKDYDRNGCIDVIVIKQPDGTLVSSPFHLRFGKLKVLKSSDKILSVKVNGQATDLVMKLGSAGEGYFLHETMDENYDDDLRASSPAMSSGEASPNRNYSSGDENDEQERLQQEAVKMNDLEQIEQKLKELDQQLVQKNDDDLIDAQASDQINTSTSTQNTQKPTPVLQDLDEEEKVDLRLLQQSPKAIQRSIEEQFKQELEKEKEKQNDGLSNKNDKKVERKSRWTWFWGELPEKQIKDQQIEEVKQTEETTGLQQDQQQDDQQKQPPQIVLEQDSSPQRIEQIPQPDQILISPQEDSSQVLSSPQQQRVVNKDQNNQQLENVQATAQLLPNQPNNIMDLTQPSNEQEKKGFLRRMFGYFKKSPSEQPLLIDSSQQQNVSSNNLNGYGQHQMTTEEEDEASEINLSLCAHLIMPIDGVQKSDEEIIMAFENHKITYEQFCQSPHLLLMDSRLVIRIEDQYYNWATAAPFIISMLAFKQPLSTDIIVTNDQEIDMNQQQDQQQQQQEQIQSYQTQSQQQQHQHQKITNAKDLPQKNFQDDSPIQQNSAKKQKVHYRKSLTPTSDQLKKLNLHDGSNDIIFKVTTGLLGEQQIQGRIFVWDHTYKIVISDVDGTVTKSDMLGHLLPRFGRDWTHQGIAKLYTSIAKNGYKILYLSSRPIGLADTTREYLKGIKQDENFNMPDGPVIMSPDRMVKSMTREVILKKPQMFKIAALKNIYNLFPEESNPFVGGFGNRDTDAISYRAVGISLDKIFIVNDDGEIYHFNSQNKKSYSLLNDIVDDMYPSVLEGLKQLAETVKDSVGNVVEKIEDKLLNNSGDKDSQDDSDKQNDQNNVESLKIYEIADSKDLFSDNTQNIEPNQNIAEPQLSHYDEEQSINNVLEKFEEGKNEPNQDPVNAQSSNTLDSQGSDNTNQNNNQHEETKDQEDTQEYSGQSHRNNGDKALTNTSNTLTMD

>Contig14985.0.g5|SET domain

MSARSTKNQTQQTKQEQPIQTTILQQQPINLSRKRSRAQFREQPKQDKIKNANELFEFDVPEPLIKQTKIVRSKRQVAKINETYSISKAIDQDNSIKQRAKSMGGEKKLKEQSNQPVTLTKQSNLQSFFKLGKKCKNLKCEKQTPSKFEDLIKCEVCSDNYHWQCSQQFQETPTQEDEFICTSCQMQLKETKRLKQNYQKEENKEVQCSQCKKQLKSEDNRQQCLKCQNNYHQKCFEKFEDEMFCLKCQKQLQQGKTQLIPEMKVPLITMISNHASKNTSKQTKNQSLTSFFNQTKKKLPETNYFADILKKDDTPQSQLINQFYSQDLMKKQNRKKSVSLTRKKNIFKLSKLIDDPLTEHEIRQSFERAFFAKDLTYANKQIYDDPLCLESKNNINLEPNVQKLVKENIESVQQLRRREELGILPPLILKHDQNQGFYVEAAQDLQDLTLLCEYAGEVRTLRQTIFDKNDSIMELLDTGDSDTSLVIAPQKYSNVGRFFNSINKSSKESKKKQNIRSIRCQIDGKATVMIYTQRNVKKGEQLLYDYNEAKNMYPTDDFV

>Contig15803.0.g97|SET domain

MDWCLKNGVKIPKLEYPAMFDHGLVGVRAREDIEHREAFLYVPFKLLITMELAHNHPIIGHVFKENKQIFTKEHEDFEQLTLTVFMLYEYQKGLESFWFPYLNLLPDVEFFCNWSKSDIEAIDDQELAYETKSYKRDIEIEWKEIELLLLHYPQHFSSALIDKHLFMRIFAQVCSRCFGWGLPTTAMIPMADNCNHSHITVVNETICRPLQIQGDSKSKYFTKDKFMNNYKDIFSKEEINEAKWNITGRFDRDKYVINSERVSLANLKKLMREGKHIWDLPYVDEEYEEDNDTEEEDEDDEGGHFDEGIMKTNSDIKTLGQSIGADRLAEDLINPKKGFKFFIEQEQRLLEKLEKKKKQLQDQHIQTQEQQTKQLEDAAQKFNSNVDDQSDDGSMQIDTSQSRASTAATKVVDLTDSNHNAQDQMMNTVDSDQVQINTATSEVASLAKNLEDIYLDPTALNIDDDHEDYNYEDEDFTWYDDKTMRDDTYFVLVNCERKTIKKGEQVYYCYGKRSNAFLLLNYGFCIPDNKYDSHIFKVKLNVNIAEDISNDYSELVPHPDNETDVLCSEEIRLKFDQINSVLLGYLRTIFRTYYQHKQISEHFNEYHLHSKEGGLLLTKPKEIDYEIFIFKKYLGLMEFLKYFKEKKSTLEQDLELLNNPDISYQMKFGVSYRLEKKKIIRSNIDLSKFILHILQTIKEAHLSGVMLTEQQYKDLYMSKTEMESNGCCKTWVMASDFNEEEQYYYKRLQLRNYLKDIGELAKICKY

>Contig15908.0.g94|SET domain

MRKSSQNPNQDKPQQDLSSQMCALILSSLDFKINHVLRDLQEYWSKQQQKKKGKEIQFFAEAHNLTKDSSYEEVLFVANDLKQNLKLPLDQAFYALYRVIGKADVNGKFHRHLFRYLWVNQEFKHQQQVLKFDSIDQLKKANSHMEKQQQQFNDIKQLKEEVLLKQEDVFKDLKNSQQINNTPMNVQSTTLFKDNSNLISTNEAVKKQTKKYKSKMQQKQSIEQQHDTLQEYDFEKKLEEIKYSSEALNNQSDNFIQSEQIHEQNGNQIKRKQKEQRIFAVHNGAQELLTNGIEKLKQKAIKEISSELLSELDNSSQNDITNNTTNYQSNQLIKQQKPKGLIYKKNMPTDDLDQVELLIGEQALNQQETNSRNESDQNSKNISKKVVKKRQKIVKHEQSQDEVQQLIFNSMLNQVEDGSGYRFIKIEDESQAAVPLTHLIWINQVDDALPYYINFGTSPNTQQYWKYVSQNFTDDLIVQKYLKNKRRITYCKCTDGCQVPKKCACYKYNRSLINPTYETMYHEDNTMIAIQTRAIQSNNRTLTFNECHPRCACNKDLCMNFLMESDNKHKWKTAIKRVKKVSVLRGQSFENVMWGLFTLEQIPAGAFVVEYLGEVLTAKEGDKRGKVYDKVGMSYLFDMSDPDDQDEYDMRVQHSNIEGFTYHKIGLFTTKVIQAGEELTIDYKWDKNFLNIPHNVACLCEKPKCRKYLMKSAYEKHRLEAGEDLLLAKNQLKEGSHTVKKEEIPQLLTPSQINFNNDFSDTLSEMSSSLKKRRIKKKVFIISKSEAQQMQGDLEQINQQFNNN

>Contig17606.0.g25|TPR repeat

MDQSMLQFMNDLFKSQAQTVIINENKSPEILLSEMIENLIPAGMFKKNAQKQEIVEKYSNRRFLMIKNFALAKDPKIIEENFLHLQQKHEKYFEEQKNFAAIQENKSFVKSKMKVKRIELLQYQKIHSQELSIEQIHYNRYLELEVITENCVKGKAFHFLASDIGHSPELIKKQSDMTDPQNSVVNCKVSKLHDICDTRYFSLGRKFVVLDPNFSFGNDSMAFVKIENKDKLILLDNNQTFENVIEEVLNNQKAIDLKNLGNKKYSSQKYYAAINLYSFGISKALLEEEETQLLSALYGNRSQSYFNTKQYEKCLQDCEEALKLDPENKKFIFRRAKVIGFLNREEEALQMLKLLDPNNQDKEIQVAVALINDRLNQSSGTYNLSRLIEQVKDLNPIFDIEVKEFIGPIEIGFIEGKNRGIIAQKDIKKGQLILVEKAFSTNEKNQNFHLQLKMSLVNLTEYANVPLLRNTHFKIIEDPLSSQRLNYLFNGSNGSLQVSIQDLEKNNQHPQSFSQRITFFEMQKIIKLNGCNILTLQNELDMLKTNDYSKLEFKDALWVISSFFNHDCYGNCSRYSIGDVLFVVANRDIQEGEEITQQYMPLMCTYEERVKTTELAWEFRCQCSSCQTYKSLSEEAQIVIRSAFFKPNNKIKEEEKNEILTMLTLQLSYDNLQGKFLTDYYAMLFECLLTFSRLKDEYSFLTFWTIFQNFADLKEVSIIKMMVLRNFGTQSLSYLIVSQLYKELFMIRYLNDEEFFNKYG

>Contig18196.0.g15|TPR repeat

MPKNNKKKQTKDTKDPQKLKELGNKAFINKNYEEAIEMFSKAIEEDPTDPVFFTNRAAVYLTIDKLDESVKDCDKAIEINKNFVKAYFRKAQALREKLDDLGAIEVLKAAIELDPENSDLSKLLDQTRQEYEEDNSIPIDHPERQRFETLLKWLEQGGSHFEKLKIRYYTADYRGVHAARDIKKGEIILYVPKHQIITLEMAMTSPVGKKMYEKGLRQRLISPKHSFLSTYIMQEKRKPESQWQIYIDILPKNFSNFPIFFTEEERIWLKGSPFLDQILEKIEDIKADYDLICKEVPEYVQFPIREYSEIRMMVSSRIFGIQIEGVKTDGFVAYADMLNHKRPRQTSWTYTDEKQGFIIEAMEDIQRGEQVYDSYGKKCNSRFFLNYGFINLNNDANEVPIKVYYHTDDQLKQVKQDMIVDHSEFKKFRVVENLEDRVMQEFFSWLRFVEYDENITLIYQYQGAAISKAQKYKRGDESDSDEADDPSKGFKAKDLPPLSIRNEKLVLERIINLAKEAYDKYERTYEEDLKILEREDLTFNHRNCLLYTSGEKKILLFLISACKKILPLLDMDFKTARKIVQSDIELDLCKEYISNPQAKPGDKKAGGAQQFIDDDYSDLPTLPPINNFVFATVPAFKYKKNLQNVYTYMLKHFQFQPEDPISQKIKVIQREDIVNYAKLKQYITEEESLDLIGKLGTEKITEVFAKTTNDLIMAFEVAAKRAKRDSQQLNQQESTDVETTLWLKDFPQTSNEFKAFYQYQQHANHIHSVIVMEEKFIPESEDEEILKFTSQDPTLERYYQEQERKRIEEKKKVEFLADERIQAFNTVLEVGRIFASSQGDSQFKLCSVSRIKFQGPDSSTILHEETLDPTQPASQHIHPENEQLYQKFAQEFKQNIDQQASDLQEYRQLKKNFLAQVQTKPLMPYPEEILKKMRIEEEKQKEIQRKLEEEKKAQEALIAQQQAQALAAQKKGGAAAKTQPTAQQNTQSRTALQSRGNDRAETQSKFEEQMAKFKAMPKSRDDEEKDWNFQQFEKVIAELSPENGTPGNILAAMVYQIANQNKKAKDRAITDIDKQFILKSEDQENDINDHFDDIFNKLAIEHNVIETTFKHHSDEQLNQNKNESVSPYQSIFNENDKVSKLAHTYTFLSGDTITSTENLALQNSKLPGINNRQDMPQQPPIQQPQRESDLNAILSFSPFSASQTKRLLILHQMEKMFKNKNAHPERDFFFLDRNFVEKYDSTTLRQILVQALRYGPDMVHHYEQREDALLLGIFYKNPPGRLLRRQWTHPLKVFPDYQVWKNFVKHDTNQALDFSQLYDIESSKVGLIRTNSKYCFPSDNSVIRIDKHQIGNRRYGASLVVKDNLVFGIKENLEKFKQKQGLFEDEILNREAFPTSDRRCTFWLEFENGVKLQVEMQERQTAHLEEIPLHEPLPPNLEEEAKKMNQTNTSMMHGGDVADSSPPNLQIESAPVGQSKPSGGLNKNESTKSIKNDGKQSQLKQNPPSAATGKNVPAQQLQKSDSQKSVGSDKSQPSGPSPQELALIQKQRDEQTRLMSAASKNQFFQDRYDPTVGAKLTFTFQQGLIVMIMPNGDVSQQIVENSSEKKKYGGTLHQDSQPANLHEKSRLITRNGQVIRYFLDGNMQIFYPDGTITTTDKRKGVWFTVNTKGIKRVRKLHDNMVYDEPKRLKITEKLDPETSAVVQVREDGVLTIKYIDESRLVMMPDGTQVLTKKNTETGSGTITIITKQGFAPVRLIFDPVKARAKTVIGLGGADSMMGIENIMERTNNGKVTEVLLPDKTTVQSYQERQELEGYNNFTTNLVHLIKRDDFSIIKVRQDGEMVLISSNQRAFLNEIGKNKEFGKNDYDHYFELFGVPNERRSGVFTVNLDQGRMITQDEEGNIFIVYANGDSVEKLSVSFNLDQMVEGIEKKEPDSPRIQDGEYIEEECKFLPPPKTIAHPRLFLIKNDVGYEFYNHEQLEYLFRCNNKDTQLVNSKKSVKIDNEEAISHMFLRLSQDFNPNQTFNGLVVTPKIPQSVELVNQTVSIPNIPFREHYEWRNIIEYKSLDVPKQVSEFKDSLKRYEQMKKRQQTESDRLKVTEIKTEEHKEKECKIWMRIAREKGIDISNLVGEEYKPLKDVVNEEKKQKFLDDLDNNFFSDEDQDNE

>Contig18340.0.g84|SET domain

MESAIQSQVSDKEIPQDDPGNVFNKIISDVSKEDFDRVVRQFRILENQFSELLDTFSEDFYKPPTSNANSQSLSTLLNTDNQYFKPTYDEQTVLSQDNVYKYCSNCFKTSSTLKQCSQCKFTHYCQQSCQKDHWATHRSECKSPKSAISLKSMPQSLRLLLSLLYLKTDSKNPKFMSNLQIFNEQVITMISNASKILMKSQEKREEVMNYAMVCFLKTAQSSNFEVNIDNFKLIVHLYFLSICNGFGIADNQMLRIGTGLYYPSNLLNHSCDPNCMVLFRGQTQFIVTCRPIEADEEITICYIDNGISERIIRQQYLQEQYHFNCMCARCLKQIGEGTELKEQKVQIQFSEEQQILNKQALHEESLGNFDKSLSQLSQLYKSVETNKSTQIFQNSMIKELLERLVYLSVMNKDYKFAVKFQQKLVDLCEIMYIKEVKREDELPHPLIALHYYQLGKLLSQRKKYQEAIDNLQKAMTLVSAYYGLKSSKDGKMIDEKLVHEVNENYLANCQKLQSSKN

>Contig1977.0.g21|SET domain

MENLKQFFDSQAQSETINSKASPDDILSGMLDVFISNGEFGPMPSKYYFTRKYSDRRHQMIENFSSVDDPKIVTQIYKQKFKLYKKSGEQQKDLIALQKKKKFAIPQMKINPIELLSYQKIATYELKIEKVHYYRYLVLNVQENCFKKNEAFYFLANEIEDSQNKQMAQSDNSERLSSVLKCKVSKMDDDFDIRHFQSGRKFVVLDPQFKFGNDCIPYVNIDNIKKLILLEDTQTFDNIIEKVLITKNALDLKNLGNKQFESKKYEAAINLYTFGITKAKSEDDQQLLNTLLGNRCQSYLNIRQYEQSLKDCQEALQIDPGNIKNKYRFAKAIGFLDREEEALNILHQLLEQTRDIKSLHEDVQKAILLIQDRLNQSSGIYNLSRLMEQAKNLKSISDIEVKEFIGPIEIGFVEGKNRGIIAKEDIKKGQLVLVEKAFSTNEMRQDSNFDPKQCLVFLTDFTNVPLFRNTHIQLLENPNAEKLVKYLYNGQNGKLQVDIQELAQKQNLSESQQSLTYQQMSKMLKYNFCGCPSLLRNLELLKAFERKEEEKIEYINAIQPILSFFNHDCYANTSRFSIGDAAFIVAKKDIKKGEELTQFYISLALPFDEREQLTQKAWGFECRCNSCIKYLQLPDDLQQLLKSVNMQPSNARKQDEMMKELIRNLPIVTSKLTQLDLQNSYLSDYYLMLQIGLAQYTMQRDEQNFLRYYEIFKHFGDLWQISKLKSMAQISFGPKSLIYKTLCQSYKEQFMIEFCNDQELFKKYG

>Contig20037.0.g88|SET domain

MYKNEAISYQGAYLTSCRQALLPYLQRGVQCLPDEDQETEIMKKLYEADLIEEYESIWDTEKEQLLKQLKYRVGHVLRIKDSSIDHHEAGRGVFLECPRRQPIVLPGTLLGIFPGVVCDPGCPMPPTPKRGLRPYLKRFDGYWIDYEKELPYPMPSPGSNLIDFAENFSSQIDKSGMHKSFIEVPPEYMNPFALGHLINHPPPDIPANVQLIDFDLPYTFFPSTVAVVAQQTISSGEELFCDYLTDERVSIEYLPDWLLEPPPSPNSVYLTKKEITANVPFTVKLLYSYQRAMGGKQIQEFEARTKHDQITDSQELKVKKLIQEKLNFGGPLLGSGNKKDAGQISSGSDEKPKQIGDSDNKDK

>Contig20227.0.g47|SET domain

MITSQDQASNMSALQCNPSTYPQCYICSQQLQRDPSQGVQMCQTCKTQAHLSCLQNSQKIKSNGEWRCDICIQQNQKMMTECFICCQPTSEQIQLAIFNEPHEISTGNLQQLEGLHSSVQNILTFGHEKCLIEKLPFTNQNLERRQPYQISRKDYINLKSAGRSQSQSPDRNSNKKSENGSLTSDQNTIIQAQSTFQPQLTNQIPSQNQYKPANLQSLLARHPKNFQSFNHPTQSQNKNILSASSEAQITINGSQTDNSQFQQKQSNLYNQVRSDNFEQVQLKHNQSVIAVEQNQNFSNVSIQSELVSQTDNQSETLTDYFQRRDFQAINQSASLQQHHSANFQSTNQGPLNQTAPPKLNQNGVKIKLDLHTEKMIGKRQRSAGDNEMVKNTISKEQKEELKHDLDLIKQENQSVEDLNEGNQNQSKERRSKNLENSPGQSSEMTKNKMLKALDADPSQFVVKEILTPILFQIDRLQFMRRDKQKFEINLLNKDLKLLSKERREEILNTTSYLDIIEKPGQQKEVDLVFIIDQNLPQRLIDSTRTSSNNQNDQQLNTVPLENTDINIQPNLDERQESDLLSAQDKNSELSPENEKMEVDNDEESQEMKIRDSKIFEEEGFNITGGSDEQSKASSDCLSKKKKKKKSYGHSAKSKNLSKALLNLPSNLSDFTWIENTQLNKQLLEEEREKQIQLKKQKELERKSKQQDSESDLSADFSIKVKQVTCEEDGLQLSETKLENQAKQDVEMIDTSTKIVEPPQIQKQVEQKPYDFKSKQGIQSIDDIWERTDDGYYHSLDELLKDIEILVYLSFMLQRVNVLIKTKKEEFKFRWRNYYEAKYVQNDLYYPRIKGSWWKTPGAKREYNLIDNYKIMTPAEPSNKYLQGSQVYKQEIIRKMQTTKASKCQGACCLKIEDLGPFKLGSDVWESKCECRLARVECSKDCGCDPTVCQNRQMSQNQGLKLGIDVIEKISWGIDMGTAVNLMTLLPKDMPMKAQSDFIEKRLVFAIQQQGDQGYDVREALKFIINDRENPRFRDIDRELAQIMLQGITMVKDNVERHFRVHSKGIGIFCKRNEGIKASNLIIEYFGEIYQPWNWYEKQDVLKQGQNKQTLSKDLPDFYNITFERHHDDPQGYDILMVDPILYGNYSSRLSHSCNPNCSTIIHVRDNQYSIGMFAIKDVSFGEELCFNYCSLTESEKEYESAICLCGTEVCQGKYLQLANDKKHMAIMKKYHTFVDRNYLLYKACKFPEITEEDEKRLNDFGIKESVLKDVPDWLKKWASLICEYIIFEEDIYPSFFKEIYPTFKEEDLRIEAKNQRDSKIWNIAITIDKVMHVLKSMGVFEPPIKDLSYKERCIRLWDSKDSLRESLIDVLNHIENYPEKLDALQKVISVPLVEAQFDEDENGRYYKEKYQEIQSVIALISGILRPIKSIKVMIPALCDSLWLYANTHTYFTPNENYKKCKGDEQKIRKCDVRIENQNQASLNPVEQEKQVYRGFKEYDPSYVWGQLVGWYKQTVDKPNASLSADRRGTLSMPDLESFIVSAKNQDNLKSGIKRKKTKKGSDDEQQEEILPTKQKGTKSRPPISTQASAWQLSNQDSISQLSDQGTSVKYASQKDPKIIYPHKKHQSRSAFLENWPENFSKQWDVVCHWSFKNKNRMYGSVQFESILQSDVDRTRRLYYFQTVLNYLRNQQ

>Contig20347.0.g91|SET domain

MAQNFMKDVMKQSAKTLSKTDSNLLGLQHSALDVSPKLYAFTQWLNSPEFIQTHKTSNMDIHGLPAIKINHPIVFENYNRINGMGYGLKTLHSIAKGEVIIQQKTALGLISNTLSSDPSFNPNKPAQISQTTQESQDSQLTPAEVQEEFESQNLLDQLISLTQRVSQHFFPNPLQENQRIRLFQHLMLTQKLILLERQTESVTVEENYMSSYLDLLPREDMTQLLFWNKNVQLTKNDPNSPYLGQNSLSIEEFMWAFSTVSSRHLVFNNQAVSTDQNPFLMMLPLVDMINHSPSFQPNVVVLPYEDKLNSESYIIIQAIQDIQENEQLYQSYGNLSNTHLIQKYGFTLEQNPNNMIQHSFPFGSFERYTYEEQQLKRKLSQQFKIPINEQRFMGSFYSNRFNQDMFKTLRLGFLASQNIIDRRGTETFINDVGDMSKPFDPSNEQLCYEYLVSALDQSYQKLQPQEYYEQQIDNIKHTILTKPSPDTQEIQQNAVKDNKFKKLAPLDQYNLMNAFRLQADESRILKSNIEFLKKNKSEAIKKVLESLH

>Contig20462.0.g58|PHD-finger

MEDFPEIDMSIGEYYKLLLEGRRPMRNRNARTTVGNDSSTRNRDALPTSIGDIAGSSRLRRNDHQLSANNSHNRVNQLRRRKGSDSSSQESEEEKKSPVRSGRDSQNKAGGLSFQVKRSQSSGQTLGNKKRLEKAKDKLGSKKGGTIGQQNQLRHHGKPTRSQMTLSNTISVKKSNLLRIQDDGGGIGSDVLRLQQQTNSMIQDQIQKEKALKEAQKPEKDLSQEREPQFEKHKEEKKIVEKFEYIDPHPLYCVDRYVINSTQNKSIQKIDKVDEATFELKTKSEESGRRQQLILLSKVFFVKIQMLESVPAQLQEQLIKGSSQTESNIVIIQKLTKGREDNQVDFVTPIQLQNGNYQAVDIKIKEQINYQKDLKDFEAEVLGPYLKSAYNKELTNQISEFADKVQKSFMSYFNDVKAIELNKNNLESLLFSIVSYEPISGDKSQQRQKLGIFLRAKYDPFYQDYVVFLRLLNCKVAQKERTDWFPLSKFSIKADLTRGFVSLDYLSSLENINNATQKLKMRMFEMVSFQESQLQQATLRSISNVQDHSEGERPAKTTIEARDERCSTCNYITNDIEHLKCSNCSKNYHLQCLKLPNIHSNLGLRETDWRCQDCIRCTNCLSLRNRDSMLICQKCNAGFHYDCLDQSVKQGVPSISNEKDLKLGIETRKTTYSQLNQFYKCEQCVECENCGSKEAGEKRNNKWSKDYKLCTTCNKKRSNKEFCLVCEKFWPDTKEKQDQLQTIQCVQCLMCVHQDCDRIFKNPNVLQQFSDGSLKYNCQKCRQNVRLRFISEIIEKLASEDKMYFYQEPVEGRVQNYTKIIKTPMCFKYVREKISEYVRNPEILKSDINLIFQNAITFNQPKHKVHKDAIRMKDLCNNILSKFWGRIEKNKLRTLDEDSHQQRIVEEINATKQGKNYKIQPDEEVFMLQAEDLLEPSEHIYNFPVGPSSSAKSNFASNQLSQRKDDQFANMDESENDQNYVARNARDKMFGAQNSSVNVNADVDMESDDQNASPGNNNDPHNLLEMRDANLGISKNLRVRKQINYNEDRLLAQGSGILGQSSNDQALSGNKRQFSNAFKSSSNIEVDQLIPPLSIAGNSIKNLKRQRQNQFKLDPDFIPPGTSTNFKPQSSAASLLTQALNQDNALISSEINNDRNETKAVDSVPVNHIQQQQQMMMSYYQEIDDEKCYYSNPARCSFDDPSLLFEEMCYLCGSFGNGEDFLSCTLCGESFHTYCLQLPEDQVSKYQQYWKCLNCKFCEICASATQEAFLLYCDVCDKAFHSFCLKPQLKSIPNCQWKCQECFKCQQCGTKEFFSAKDNEEKRNLEVTDFEFSQNFSFCYQCGKNEYKKSFCKICQKKTEDSDSESKQENPRKNKSIHSHSLALHQRQSKSKKKSLQDQMMKCNECGFYSHFRCSKLLPPALGEIRDQDIETNPEILKEYKCLECLLAGKTINHLIRETCEQYENIHQIISRQKLLASLCSDILSKYFPIQDKAPLFIKSFITQNYDFFRKDKQINDWLELLESNVCLESADQGTQDTEKMQDLDEDVQENSKLPDDDQIASAINQEERESPQITKVTVQDGQQERMNIETSIANQNDFSKQQKQFSDVHLEESLQKFDKNQIEKNKPQTKAMQVYDDMYGCKQTFLPEKRNLYKSLSQGDQHRFGQRDLFMQPSELSKLITVPTEENLAKINFSELYVQVEFKAVMRIKFHFFKWFEELIKQILRNNSKFIKTDKKISGENPKKSIEEQEDLEQIAFNYDQVQINTLSQKNHASEDELCTLEDTLVQNPQVKLQCVLCRLFGEYTVTGRLIPFQVNQFVHVNCAVWSTDVYEIGDGQLVNFFFVQNGKSRTTKCVVCNETGATVICFTRKCNQAFHFPCAYRENRVAFLRSKETYCDYCTKSREFQVGYLQDMTTKRRFQIARNQIPWNMSIMYQPPPGQQVNPFLMNTQLFALQQTQVALNNVSNPTDMEKPLQIEKWRPFYFDMFNRVGNLTVLSLKKEINFIIEETIYGTKKEINMDDLTDFAAIRVYWKFYKDYLMNDQEEESINPLNLRKSYFMFSNNTIREVNKNDLNSLVNYSSEKIREKIQIGIHYNQAKNLQIHVDEGAMTPINQTMFWQKIMQHFQRERPNFTLQNIIFSISEFGGKTKYPISVWNRAEVKNKGFFLKSGNQVLSQQNQTQDRDSQTQQSAKKFEGKTVDDIFKRMIKINQMGDNQNPMISMKRLTSSKHMKYENMLSTLGKKMFYSKSHQDEINENKNQQKSTVLAKKIRLTDTIDTNKEIDLPIAMKYRLSKAQPKRVEVGPSKIHRNGLFTLEDLMPGDIVIEYVGEKIRNKVADKREIYYEQKGIGDCYLFRLDKEYIIDATFFGNKARYLNHSCDANCSAKIINVQQQKHIIISTNRQIKSGEELTYNYNFDYETDKIACFCGAPTCSGRLN

>Contig3702.0.g12|SET domain

MRKSSQNPNQDKPQQDLSSQMCALILSSLDFKINHVLRDLQEYWSKQQQKKKGKEIQFFAEAHNLTKDSSYEEVLFVANDLKQNLKLPLDQAFYALYRVIGKADINGKFHRHLFRYLWVNQEFKHQQQVLKFDSIDQLKKANSHMEKQQQQFNDIKQLKEEVLLKQEDVFKDLKNSQQINNTPMNVQSTTLFKDNSNLISTNEAVKKQTKKYKSKMQQKQSIEQQHDTLQEYDLEKKLEEIKYSSEALNNQSDNFIQSEQIHEQNGNQIKRKQKEQRIFAVHNGAQELLTNGIEKLKQKAIKEISSELLSELDNSSQNDITNNTTNYQSNQLIKQQKPKGLIYKKNMPTDDLDQVELFIGEQALNQQETNSRNESDQNSKNISKKVVKKRQKIVKHEQSQDEVQQLIFNSKLNQVENGGGYRFIKIEDESQAAVSLTHLIWINQVDDALPYYINFGTSPNTQQYWKYVSQNFTDDLIVQKYLKNKRRITYCKCTDGCQVPKKCACYKYNRSLINPTYETMYHEDNTMIAIQTRAIQSNNRTLTFNECHPRCACNKDLCMNFLMESDNKHKWKTAIKRVKKVSVLRGQSFENVMWGLFTLEQIPAGAFVVEYLGEVLTAKEGDKRGKVYDKVGMSYLFDMSDPDDQDEYDMRVQHSNIEGFTYHKIGLFTTKVIQAGEELTIDYKWDKNFLNIPHNVACLCEKPKCRKYLMKSAYEKHRLEAGEDLLLAKNQLKEGSHTVKKEEIPQLLTPSQINFNNDFSDTLSEMSSSLKKRRIKKKVFIISKTEAQQMQGDLEQINQQFNNN

***Salpingoeca rosetta*  (Choanoflagellata)**

>PTSG_00179T0 | PTSG_00179 | Salpingoeca rosetta predicted protein (111 aa)

MPFDNRFAYQGQRDAERKPHGVGTLLHGGQLLYRGEFEHGSMTGRGYRVYMDGSSYSGAFHDGMRHGYGIWTSADGAVVRQGTFRADVFHGALRAWRLSRVNSLRGPPNA*

>PTSG_01368T0 | PTSG_01368 | Salpingoeca rosetta predicted protein (628 aa)

MGRRRAQLEKLRRKKRGEYKRKMKLLKQNAPVVGNSDQDAACQAFVQWCLHRGIEFSPNVAITRKRVVHGRGMVATANIKAGEVLFEIPRSAMFSEKTCKHADVLMANLDHGVHSVEEDSADCHEEADACDGACRTTAASNREDGGGDHGGTCCGSHAKEHKPTTDEQTGDIKLSGWSPLLLAMMLDMDAGEASEFAPYFNILPEDDELHHPHVWTDRERSTLLKDSRLQEDVARDLTLMKREYDTIAKPFMIRHPKIFPQPGKKAFSFRKYAQCAAIVMGYSFTDEEDGRVCLVPVADILNHVTGKNNARLFFSDKTLQMRSIKRIPAGAEIFNTYGDLDNLQLVQQHGFAEPSPTPWEEVSLHPRALKAILHLTDDRFAFLKDTVSGLLGEDARLLVGILGDAAMSYPNWLFAALVTLCVCQKDKCEPLREHIEQVHAAQAEADSDEDEAEEEKEEEDEAVEGQVEAARADDGAEKGEEAQEQEGQEVQEQEEQVSKRAKKGADDGNGNDDDDDDDDDDGNMEVDEDDDDDDGDDQHEGKTLAEQMEGFEYGGIELTEATQIALHRVMKHQSELYARRAEEVKAAGECVRKRRKLAAAVFEAGQAVCASWLARLGASDGGDAEKS*

>PTSG_01403T0 | PTSG_01403 | Salpingoeca rosetta predicted protein (432 aa)

MEDVVAAIAELKNVEVREIAGKRKGLVATMPIKAGTPILSEKPAVCFSAPANKKFKNAGEPNFVLAKSLLSRPDADSVGFGLLNAYMPGHDPDHPIFQDRKDILDEDAQRLFQFLHPEATAHPASDAPSQPQPTENADGVEPLSLDDLRQLLAVIHLNSFALSSERFPGMTTYGFYLRMAFCNHSCRPNACQYIDPNSTRARLNSPSIVLRAVSDIAEGEEVCISYIELMDTTPERREALQELYYFTCQCPRCERALPLHLPSSSPSAEKKTKADDATATATAGDGEEEGSLALETRVVEEFRATLQAANDKAKAKDMKGSMLAWGRCAELGERIYPSNWPTMATLYKHAHTAAKAAGANPFVLEAWKQRFNEARRKCRGPQCAACNCFLTGPLLCGRCRQVAYCSSECQRQHWKAAHKRECKPPAKVPDN*

>PTSG_02551T0 | PTSG_02551 | Salpingoeca rosetta hypothetical protein (817 aa)

MEGGDNSGDAGLHDSAEQGVQTQQSQQQQEGGERSRQGGGEEAKDVDTDDTSGEQVKVQGAEAGEEEEHVTQHDATTTTTAEQPRKRAKHGSNDDEAEEASGAPVQQEQQEQQEQVRGEEGVEQQAPQKQPGQNSTGEDSFGQTEKHGDSTATAATSGDAVDEVRHEKVMDEASDEAGEEVKQKGEDEREKKKGEEATDKGEGEEEGKTNEEKDEKDEEGKENDAKDEKEKEAASEEAQADPLMVVLEDPRTVVPTGIMDVEILKLIHPIMAHGEDMDSEANIQALKDYSKLPEDEVVAAALQEHLRYSSETAAEIQRFPGRRRSVVYFRDQPFLPSYMWPQGKGEWFTPLLFVKCLLGMLEDPSDAQLRAWGLRKGLTIRERDFLLKYEAFHPAVICLEDRLRMVRVDQVMSWCKEFRPAAYRRYDDARSIPFLGFSVSRRKAMLERFRANIRASAALAAGFNKAIARERAEERHEHHDLLTQVTLTPRDQQLHLVGCAPRSKPFAHDEGQLIEPVKNPLEDETVAPARPPSAKVVSVLHSQRSGERKEFDEHREHQKYLVRRAIKKFGGDTAVVMPKAPADTECGVCECTADDGPGGDGEPEELLTCLSCDRSCSSSIDARGLCIPGHVSATMKPEMGVAVKDIMTGTAWNCRVFEKCGSTKDEKDILFCDECDRGFHTYCTGLTSLPRGRWICSHCSVCDGCNFKPDDPSTYKWSHFTDRRDNQRRFLKTFCDACFKKWNTGDFCPICLELFEPSADLLECSLCSRLVHKDCEDLTPEDEETFKAQGWGRFACSICSGFKPELYDAFHRKYQS*

>PTSG_02583T0 | PTSG_02583 | Salpingoeca rosetta Mll1 protein (1191 aa)

MEKGWRLVTLDGKRMRISIQDGRRAKDPRDPNATSATPTLRSLPQNPEYATPEIRSVFIANLSTAVTEEQLKDTMAQFGPVATVEVFTRLFTREPTGFATVTFATTKAAKMCALQSGQLRLENQSVYVHMDHAIPNYKADGASATLSEVYMGKYHTDASRGHTPSFPALTQLYPDPLIRPEDMPASPTSSNMGMSPSPDAEYQFSSFGATTTTTNATNADAASTTTTTAAAASTSPASNARADHLPMQQNNSSTHTTPHHHHHRRHHDDERTRGRDRHHSDSDHHHHRDDREREPSSHRRRSGERSGRWSRSPSRTRTRTHAHGHSHAHGHNRDESRRYSSGSFDGESRHQHDDTRDHGYGDHASRRDHTHSHRRNHRGGGSRRGGRSGSRGGSRVGGWNSPSSSWNSGGRAGGDVHGHMRSPISGTSRSPYTPQPHGRYGDRGDHIASPLQSAAAATPPLPPPQLASSSPSTSTPTSHQPLVVLQIPGPFPRRVAPSDWLDFLSEYHPVQARFNTRHSVWRVSFRDKRDADAAARHCLHSQLLGYRLSRPRVLPQRSNSTSDNASHDSHNGTPHSKQQHQQQQQQQQRGGSWATTPMTTMTPTTGGGGGAGVYDGTTLTAVPKALAALLRNKLGPGGIVEGEGRGSDDEQHASGGAGGARESAHGHEHVRVMTWKEACKQRLSHLLQRSFVLRFSQSVVEAEVRRVVENHFAAIARRQQQLAQEEEDARAHLATPKAVSITSLSSLTIERRKDLPASTRSRLPSHTRARVTGGVRARARRTAVAPTSHVDGARRRRKPIAPQAGGKGDWWADMELASSSSSDEEEEQEQGDDMEEDEEEEEEDVMEDEDEEDESDVAHDHDVEFQHRAKRRHSRRFDSTDDEDDDGEGDDDKTLVIWQQETESGMWLDGARMGEVGGNTLGFFGAVFGPRAQHVLGHSFHGALHHWEAGSSSSSWTPRVVVSGHFKVYPDHSHEFHSTGCARTQGYYFIEASDKRKKITAGAMQRQLQVRQTTTSTTRTQRSAVRKEHRALQLGDLNELAADLVKANQFKRRRKLLRFARSMIHEWGLFAQEPIDKDELVIEYVGEIVRQTVAEDRERRYARIGIGSSYLFRIDEDYVIDATRMGSIARFINHSCDVVSVDGKKRIGIYSKRPIAANEEITYDYKFPREEGPNKIPCFCGARTCRGTLN*

>PTSG_04178T0 | PTSG_04178 | Salpingoeca rosetta predicted protein (1397 aa)

MTAGGRGSGASGSTAQPKQLKQAQQKQQQGSRRGRRGRQEQGGVATCADAGTPEAKDTAEVEEAAAKDQAAAKDQPISEQAMKRSKLDSAAVTAVTAVTASSAAGEASSSAIASSQAPSADQTTATAASNTSESEAGSHTPTTAKRRRSAPKRQRKQPRKTSSSTAASKEPNKELAGDGSKVGADSEAVGDSTNQAASTSEATQDGAKQGDQADQADEADEAAKAEEKLYLQAGVYSYTFKRANPPPLRPERTNFKGRDDYVCRGLPLPIYNGLNLLCQPEPFRLPASYVQASRQGTLAITEEACAPFRKIRVNVFVDKETEQFMAKHRQEVSRCECIPPPDGGPGCTHDCLNRLMYSECGPDCPCGRQCTNKRFQQRAWCTAIKRAPTPGKGYGVFATAYIPKGTFVIEYTGEIMTSSAFTQRANTLYRARKHFHCLNLDRGLVIDAGQAGSEARFINHSCDPNCHIEKWNVNGHWRAGVFASRDIKGDEELSYDYNFHNFNEKLVCRCGAANCRGEIRPRTSNETLAARTRAHTHAHLSRKQRIQKSTEGAELFNDFLWRRVKGEDMCAEFSPLLKKTQRIARDRRVFLLRNFNLLQQAHTMRSMEGDIKAAVSERRNEFISAFQAKFRQLRGASAGGSVRSTAARKTTGISEEWQLDIRAHMAVVLDSFLKEVMNHRDPKSGRKLAQPFVNLPAPHRYPRYYKVVSRPICLSSIQHDVRSGAYGSPAPFIADISLVWKNAKLVSQPGSERHTCALKMEEYSLLRWKQHYYDAVKAARRDAIGTNTAHYQTILARMLPDDEITRCVCRVHTEEGQMLQCSQCKTTFLTTITAQGEGTLHMFTREHRMCSGAPVDINIELETEVQKLQSLVQAPPSDTGVDADVKQQQAKIDSAQQRVQTLRQQELELKTRVRAAKQNRNKLSKELTALERASSSSSTPTRRRSRGRRRRSSAQNGGEEDGGGGQSCDGDTSSDVGEAGGSSRSQSVQQHHQVYQEQPQQTQPQQQLQRGQPQPQSQEQQQQEQVQQQQQEQERRRACESALTLAEVELSTLVLALSGARKDMRAAEAGERAARARLDALLFEKSTATAVFYRSLETSAGHLHLGAFVYVQRSALVGDEQPPISKPAIVQITMLTRDVRTASVSVFGQLFFYPAETFVHHTQRFYANEVAFTSTTVEFAPDDIAGNCFVMTPKEYVHMRPLCVAADDVYVCEYRYAPETRSWASIKTFDAFPLPLNRTFFRILPEKNDIQRSLTGEQQHFMLRRKSVKADGMSLGGDLYVTFITREEGDTRWPPFERELQLQRHGASASARAATPEPRPPLMVIVEDLREKLKDRAIKSVPRSSVPEVIDASYLLADTKRAQRFLARTTPTIATAATTPTTAKTRPALPPPASAP*

>PTSG_04361T0 | PTSG_04361 | Salpingoeca rosetta predicted protein (465 aa)

MLGVRKTVHGRGSFWVKKKTTKETKGGDEKSGEEALDGERLRKGTDVIKGKRPFAHVVEKAKMETVCQTCFVQCEQLRRPLQRCAGCKALRYCSAACQKADWKDHKPECAALKRISPVVPATFVMFLARILRKMERNTGEMDVLQLHMPGEPSDPQQQRGLFAILEHLRHFLPDAEKHLLKSAYPVLRITSANSFGISGVEGNNLGVGLYDTVSYINHSCAPNCSITFSGVYARVRSVHDLPPNQELTIAYIDPCDPRAKRRAHLKSQFMFDCECSRCERERDDDPLLTLCCPGSGCDQIIKYQLTAPPDDTAFEVTKKCERGHLDDPATFDSWSRKVKRVLGIIGEANKARRPVQGDMASFERLLPPTNYLMHIAYTGVLDQAIGTGQFQEALQWARKVILVYDALNSPMDPLRGIELLRVAKLQALARDGRGFLETIQKAVDVIRLTHGEDSVVYQQVLALH*

>PTSG_04400T0 | PTSG_04400 | Salpingoeca rosetta predicted protein (1050 aa)

MEPARQPAWADVLGCSLVKGLRLCRSASGDDEDKQEGEQVHVEAVEALVRRTGDGDDDDDDADEHGIVLVTVPQDAVMSLHNVLQTHLEPCALAASPTPPSQDIGAKQSAACTCPCAVIHALVEDLPRDLDTDNDSHDIGTIALATAIVFHASNPTSKWHGYLSSLPKHNLTTMTFDERALHLLRGTNLHHATIDRRNATARTAATICRWLQHKWPQHAAAFTLDAYVWAAETISSRALSGRVSQPDTVIHLLHLGIVDGDTPTSASDEQSSTRTSKSVVTVAPFPVLAHTPCLLPLLDLFDHDPQADVTWRNTGTHVRLITREAVAPGEPVFNNYGGKGNEELMLAYGFALPNNKHDDMHVMLGLPASLQQQLVAADDDVDVADGDADHQEDGVGVGDTNRDAMDASAPAHDAAAGIDSACAAAADLSSAFAALGTITPRGLRLSIHAASILADVTKLLQALSIVMHSTPFPLAPQVHARAPRSVARDPVATSVVDAACSVIAAAIGDDVVGGAEACVHVEGVAVTASDSSTRARAPLLPLDGVTVMRARAVCLQARTMLTSKLAHHTQLLRECNALLAMCDGNAGVDGDGHGDGDDGADGADGDGGGVGAVQTGKGVRALRRQLQMVQRFLQGQTRLMDAALVYMRTTLERELIVRGCAAMEQQQQQRQCQRVQLVQQQGLDGHMLFERVDDEPHAVHDGGVASNATGSSSGNDIGGGERVVATATVLTLDVLTSLSDGAALLLANIEGLEPDYAFALVLLAKLSSPDVAAVLDPFRPCIAQHVRRRGRTDDDDVDDDDDDDDDDDDDDDDDDDDDDDDEEEEEEEEEEHNNGGDGDDGGGDDCEQVLKKPKLKAGGVDVGDVLHQGGESGGGGKVVDAMVAQSRAEDQAVLGDLSPLIAHLSDMYPRGITGAAWAAALDCIRCRLLDVPPSPSSSATGPALLSRAAPVAMWAVGNVIARVHGSGSTGGVSGGGSDEGRGGVVKGLQHQQQHQHRQALPVVALVDDVGTLSCSDPIVLGIPEALLVQADAEMWPALRAVYCSHVHCQ*

>PTSG_06018T0 | PTSG_06018 | Salpingoeca rosetta predicted protein (589 aa)

MMHKDLRVAEAEHVGQHVVATNAIQTGETLVLAQGWRFNTENNDAIAFLRFIGAALASDDREQRTKSQQLVEEFQDVCPRREPAEDAAAPSDPLLAWVPDVDLYKKKLDQNCFDQGFFPAASKFNHSCTPNAEGMCLTAHDGVNFFEVKATRPIAAGEEVCISYLGVPQIMLPADQRRALLRTNYEFTCACARCTDEDNGTINATSVCNSLTKLSPCCGADLAAMQNWYTCVSCGARHDENKEADERLTCGFRAAAAAAWLNSIPDVIRVHNSLCEGLHRQHWICATLRVRLLHLLDRRREEMRLQDKREQELFTRHPHILQDRLLLPREERQRFREERCEFERERASLHILHAILLHRQMYACDAVLPRLSATAMGACAQRSEAWQAASTLLGELVWDLKADAKEQQQWEQEQQQQQEAEEEDEDEDEDVDEDEEVQLRQKERTAKQPQQQQQRLEQQPQPLKQQQQQPLKQQQQQPLKQQPKPQQQQEQQGQQGQKQRRRGLTTYEQWTALPPLPYPDDITRTFQASFDAADAVVRWLDYLNMASLSEAARRQSEIAWPASNHVPMLQEDSHSHYHYHDPRTTTIN*

>PTSG_06691T0 | PTSG_06691 | Salpingoeca rosetta predicted protein (599 aa)

MSSLLPLLRTALGVDAVRKAAAAAVQNTREWQAWHGCAGGDGCSLYHTATQWPKHGDDSDNADADDDNYDSDDVDDNDGDGDGDDRQQLLVLCHVIGVLRCNSFSVPTTTPTTTASTTATMVRKRSKSSSDKTVPVLPGFAHPLLSDKRLVVAQRGQHDKRGDDTHDTNNKDDRGGGEGGAGTEEVQHSSVARALFLRSSVFNHDCRPNAMQSFAGRSLRVIAARPIAPAHTAPTAATTPVGVCISYGPLACVSPVVERRRACLQQFGFECACATCKLEADVMALDTTPRPGATAAHAADSCHDNPDDDAADDDDDDDDDDGDDEGQPRQPPAPATTPSSSSTNTTTTSSTLRAPLPSLLECRPVALRCRRCATAYGWAEVAGAVNLTSLRSVLLRALLISSGDRSEGGGFKVLAVVPAADADQGGDGDDQGGDQGGDGKVVVRVVAASDDSGPLLPCGCDLRDELSTCLHHLSSALTGNLLSHDVRCAAIHHAECLASHLAPTCTIAAGVFDKLARVGVDVAHDFTGALALCLQSTRICQRAFGPVTMEHKHEVAKLKSLIDAAQYRQRSHTAEEVSIIRNAQQYVDSVAPALVVLS*

>PTSG_06889T0 | PTSG_06889 | Salpingoeca rosetta conserved hypothetical protein (837 aa)

MSSPSSPPSSSANVWRPHVFVDGSCKLVSCGDEVYVQSGRRVVARRDLEPGTTVILQRGVAVPTPYLPDPLTALLHGDDDDGDADDDGDDDHGDARESLAWAASMQMCCPICFAARGEPHAGGEKGSRTCATLAALLAPVEGVLYPRVQENKGKLKVDDTLLRLWLRVIAVLCIDRQPQDAEATAFIGDWHEALRQCSQLDTSAPDGEWILDVFSAANAFVELLDPEHVQALEAKLCPAIAISQTAERATSDGATGGTNDNSDGDGDGSAAATVSAAEALVVIAGQMNCNGYPLRLSTAPNTLVGMGIFPAVAMANHSCSPNCAVVTRPGGRLAVVTLQRIRKHQELTVSYVDLLRPRAHRRQYLLASKNFHCRCLRCQHPDAFTWDRALTLPLCSRCGCLPRWSLPWRKGEPVIVEDGDGGGDDGGQDNSSSKDNDSSSKGDATTTTSSPGTTTATTPSSNTTTTTTTTTTTASRQRTASVSGGRSGEDDASEYESWTKDGHVLVCWTEDGGCGHMLPPWLPSDVLPPLHAAIDQCQAALARLTRAQVLRAQKQKQREKEAEAKREKARAEEEARAKAAQQQEALDDSGTPRVVDVTEEMEKQEAAKKKQQQQQQQQQQQQQKAARNQEGTASKSAGNTNRPSTDNAGSAENTKHDSSGGDEDDSADVTRMCEEAIANAQHVLKTSVDVTVSCFHHSVIQLQCVLFALLIRTSPRANMPASIAVLHEAVHAMGRHLPSLWPELNEWRIRLLEANQVLARTRKDHGAKNAGSSSNRKDSGKDRKKTKKKAGKDEGEAGLSSKQRTNLRQDVLAAVRTVCGEDHPLYTRASTACANV*

>PTSG_07559T0 | PTSG_07559 | Salpingoeca rosetta mixed-lineage leukemia protein (1992 aa)

MMTASQPSRASSRKAAAAAQANDNANSNGHVAASRRQQRQQQRQQQRQQQQKPKAGEVGVGGDSSSNSKALAATNGIPRRRSGRVRKPVIREGDADEDEAHAQSKTRAQASASSATASPRTSRARNGNGNGKGLHTPTSAPGPARNRRRSQRIKQQEPVEPHSAPHKRRSSSSSTSGNGSSNGSKAATAAKMPKTKQVKQQAKQQAKQVQRQGKTAARNDKNAKRSKAGSAREVAQQHPKKKQPQRRRNQLKKPKLKKEPRHKRNGKGDDDDDDDDDEEDDGEEERKLNGINTAELQRTTKTPTEKPGALQCLINEANALEEEEEAQPSYERETETFCSAVRELHANGMLQTGDVCEFKGVVAELDGDGDLVLADGQVFPTPGMMACIMQWSGFSSNGWSCVKFYSSSPQLMDDGPTSSTKRGKRARKSSAAAKPAYSMLTIAQLRKQLLKKEQERRQLKREEEAASGQTAAAPLPGTTPTTAAAVKTEANATAADKGENGASCHDTMNIGKDDDDDDDGGGGGGDDDDDNDGSKAASHIVTKLEASAAPFWRHGVTDRISGRLGRCVAGTSPLLAHLELCVRCGSSGVFTDGNDSTNSGYNSSNNNSHNNSADGNATKSTPSPSSSLAFVHCRVCCQPYHRFCTGDSRLQSDERWRDAAEQWMCIDCQTCCAGDGVPQGLKRDDVYMCSACVVCTKCGSTSPGEFKGSTWHCQFELCEECHGQTLKGNICPMCDKLYSDDDFDTPMFCCEKCERWLHATCVDPAFKTNMELYYLISGVQTIDYHCQDCVRLSKGVLPDFAALWKATQAAFQERLVGLVEDMKQQEGYTALAAVSPAPVRDHTSDDTRTRASDDTRTRPSTGTDEDGNDGEAEGHNGSTFGLEQFLESVEQGLYRLPSRFADAFKGLVEGCLQAHLDTIMVTRWQVDAAVALKKSITHLENAAKLFDWCMDQLQKRTSVAEFKAPPLAMTYADIKSVLSPKLLLFDGTWCPATLDECVQYVQDEKAKKEAKRQAAREKTKARRANLKDNTPKKQSKSKNKRSRGKTRRARASNAQAPPAEPTTDAASTTAEEQATASTTTATEAAADTADAATATADAATATADAAATTADAATATVSGAADATTTTTILGAADAAAATAVSSSIASSAVTTPSTSAQPSQGSHVVSSASQQSEELHGSQSSATDNDGGDGGDGGDGGDGGDGGGTTGGPGTDHAQQQQELQDGELVAKRAKLQDVKQEQATATAEVAVLAGENTNGSAQDEAVQHEPPAEANEQQGHMYSCLDMSTPANDDGVTVRLFQQGEGGDCGDEDDMKELVGRLSEDTRMCCLCHQHGDLDPKDGGRLLPLEVDGWVHVNCALWSSEVFEDTDGTLQLLDSAIRRGKRMRCTTCDHTGATIGCCSSSCKGNFHFGCGLDSNTLFLSKEVFCKTHAQRLPGHKTDMGQFEVQRSVIAPPMHTSVEDVLECLKRPNPADRKLNALSSVGARTLDFQIDDLLVRCGGLAVICLGAIKHTSPAMHTRTRIYPDGFASVRTFFDLHDPSTTAVYLCRICTTPAQQQSQHQRKSQHDGSQGNQNHASCGDDHTVPSSQLSACASSQTKSESVPRFQVIYLPPRPSRDTAATSTSDAPTTTSTSDAPTTTSTTKTTAAGTSTATNTAQRRAVVFEGPTPEAVWSRVIATLRTNGWDKHDVARPPAGVHLFGLDVPYVVRLIEALPNAARCEKYEFVFARPPPPADEANTGRVNANGCARCEPLSVLRKKRQRRRPHATLAAANAGTSTTGTTQAPSSLAIAGGGDSSSSGGASSGGGSASGGDGATNGSGNGRRAAPASAMGHMQSHLSDAMQYRQCKERMRETVRVGYSKIHGFGLYAQRDISGGEMIVEYVGEVIRPELTDKREQFYDARNMGSYMFRIDDKQVVDATLTGGQARFVNHSCDPNCISRIISTDRGKKIVIVAKQHICKGDELTYDYQFPLDDDNKVRCSCGAANCRGFMN*

>PTSG_08195T0 | PTSG_08195 | Salpingoeca rosetta Ehmt2 protein (352 aa)

MSPTSKRSKKQPRENDEEKERVTAQRQHAASSYNHISQLKLPVGLKEEQLLVYEGCACSTAGTQSTCVAQCACALRSRPGLVRFECSDACSCDPETCAQRTCQRPSKAAPHLVVFDCGGSKGHGLKTTCAIPRDAFVLLYLGEVISLPEARRRAAKQDRDGRPTYLIRLQEHSTGSQARTIVTCVDAEMYGNEARFINHSCSPNLRLEVVRWHFIPHIALFASRDIADGEELTFDYGVADAAAGDSGDDDGDDDDDDDDDDDDDDDDDDDDDAAAGDSGDDDGDDDDDDDDDDDDDDDDDDDDDDVGGAVGGDVGIVGKEALGVGPSRTPCLCGASVCRGFLPRHDFGPTS*

>PTSG_08196T0 | PTSG_08196 | Salpingoeca rosetta Wbp7 protein (2384 aa)

MCISQDTQHCPVCKDEFEIKLSLQYILQCNACKGLVHDHCDPLIKSRRTRKKYIENGYECPTCVSKGAATQPPNERAAAAASSDHGTRAGTAMNHLHHYHHHQQQQQRDEEGNRREGREERNHDKGKKKESNTDDKALATNGTQEQQQQQTKNEKKNEGKATPISTRSNDDRTPQPEGVPAQANQHDHAEMQEGTVSSCKQRATTTAPTDTATPVAAVSSVAASSAATAATVAIASTATAAEAAPTVLDTAATLLNRAASPRATEATAAASKPAPALTSAGRKVTAPTEAPRAMSTSDTLTQPKPAVSTSDVQQQASKDTAPTCGSHVGLASSASVSPCARPGEQVVTNPGGQRRNDNKTKTQPKRKDKRKVKGKIKTKPQDKPRVKDKVTSTNKIRVQGKRLHPMAAGEDENCTAPARQKKPKMKSKTTAGRPHPSPHSFVQMPETHQQQQQQQQQQQQQQQQQQQQQREQQALFNGPDARLSKTFRLFAKFCEWQSNVQASPTPALPTAYLPLTSASSPQVSLPLPASTSAMSQASLMPGPHRLEPHPAFTLEQHHQQHQHHQQPLRGPFSLPVPAVSMSASALPVQQANSMTNGIRRLTQVGAASTLPPSAAQSPVSAVACFPPLSQRHAQQRQQQQQQQWQQQQQQWQQWQQQQMLMGTSTPAGNGGTFGHNSGTPQPPSVPLVSPPRPVVLPTPPHAFRQAGAASFSPPRTPSSGAAAVSVPSTLPPALMPSLQTQRALQQQQQQQQQQQQLLNCFGMTYQPPHEAAAVPREQPSVPRLTAHQAGRAAVALPHSMPVAHPLFKSTYANNSSSNNGNSGGNCGVSSVAHPTMAAAAAAEPFTSHEHLASVLLARQQQQQQQQQQDEEVKRQQDMAQHVPQLHHAQTAVQYRPPQQQRQPQGSALATQDPPTTAPSFLQGSLMHLLQRVNASMQNLARAPPAPPQPQPQHTNSNAGHYVRGAASCSSASDAVQHGGSNHSSSVWDNVSDLFKQPHQQQHSSPQPGHGAGSTEAGAVTSSMLQHQTSPTKHKHKHKHKHKHGHKRRRKHKHDHAQNTKQAHSLDEQCAHTDQRQQQQQHKQQQQQQQQQQQQQQQQQQQQQQQQQQQQQQQQHQHQQQHQQHQQQQQQQPGKRASQGMGSTRASSDGTDVGRTVSPDSGCAVSPLQPEDDATSSPPSNSDGGRGEENGEDQSKAGCTASLSAAEPKTKRKTKTKTKAKTKTEAKTETENGISSATTVPTPTTTSMPSTTTTIITTTNSSVPTTIGTCEGEGMTTGANVPANVDDDAVVASSTSASQPSASRAGDEAGTGRGNLRGAHGAAVDQSGTGADVASTAAAAATASSAPLTPGSSWEDTEQQRKQRPGTSREGAETGAVTTASAMTTTATSAPASPSAPADEATLATAAAAAATTAVATEPQPSVPVSPLRPVAVGPIVSVTRSTKRSTANKQQRDRETRAAAVETLRRTAASSPSLSSSSSSAASSLRPLATVALKGKGGAEGGAGGADDDDDDTSARAMRVKWLRSVAQAWKHALPTPRRLDAGTDVRPCLLCSGRGDAEPHQGGRLVNVGMDKWVHFNCIWWSAGLEREESSGRVQHVRRVSDVLSRGKQSRCTACRKKGATVGCHVPSCNANMHFPCAVAHNCEFLEGHLVYCAKHRTTSMDVTVDASSPPGSDAADAAATAVHAPATVGSVQCDDSGTAGTVDAAAATVDAAGATVTEHTDKQQRIMTEFGVLGCVVFERDEWAAYDKFFDCPTGHLRIGSLTVTDMGRIIAADGFHTRDELFPLGFTAVRVSSWRDVSSALSLPPSSSSSSSSSCGPSSSASTPAAFRHAAKRAKTASASRETKEGTSQSSTRGGVSRVLCHVSITKSTASRHRPEFKVTVSDGSATIVDFTCATPLKTAVAFLRIAGVRLSLPEKPCPPVQARETAAAPTTAGAGVSAAETETNQTGEKKTKQETRKEGTMQESTKELNEDTVSSRKRMLETVDAVIRVISTARNNNNNNNNNNNNNNNNSDADASSSVHASTLVEKKATNVAQHAQVHTHKPAQIAGTKAAEPPAIAAAEEEGTATAAATAAATTTATTTAAVNAGREHRLSDSASHGDNSANGNKDKDAVPVPFNGLWWFGLDTPIMRLLEFRPGAARCQAYRHHDELGRHNPIEALMPPPPPLDVPTRLQPVRSAAARNLERLLTYAAREVPHALHRIKPRRRPTRAYTVERLPSERIEARALAKMRKSKLRVGRSRISGHGLYAEQDFKAGECVAEYAGVRFRTTLADVREGEYRDRGLGCYMFSVGNNTVVIDATLQGNWTRFINHSCDPNCCSEEIDIEGQPYVVIFARRDIRAGEELTYDYKHQMDDDDKAPCLCGAIDCRAWVDMPPLTEC*

>PTSG_08197T0 | PTSG_08197 | Salpingoeca rosetta predicted protein (1152 aa)

MSTSVRAVRSQQQVSPTTTTTSIRLCAPFPSNGGTPLTSDTREAPRRDCTVTCRYERGTGASQFKDATTSPVELDEEEDGGDLQMTSAAIPHVLKALNIARKQRHLVVAHDGKAWFAATVVKVDEKKGRVRVHFKGFKKTADQWKPLSPSAVRLLSNENEESVQMVPVYRSIDVDRVHGRQTLVPKRYTTDSSHPSSSPSPSSSPLSPPPPPPPPPSSSSSSSSSSSSSSSSSSSSSSSSSSSSSSSSSSSSSSIATAATSPPPPPQVLAVCDRPRCKRAKSTAASHTRTRDEERQRPAASSRRGSKVSTATNTATTATTAATPTTQRAVPSPSPVAPTARRRRRSTFAKPTPTPTTQETPAPAAAAATTTPACTTVAAVPLLSRRASRSLVPVPLNPIPTPQRARPSNLRQAIRVPARMLDAICDAPGPQQPAQQRGLPAPEATATSLTGGKATSHRATTTSARNRRSSSSSSSSSSSSSSSAGGSAAKSTRHTTSTTSRRSSGAANKTTKGATGSSFRSSSSSTPSPSSSSRPPKRAVAQPGSKSSSSSSSSTSNSKSSGNSNRSSSKPKRDHASANAGGGSDERSLMVSNPFLANARARAKRARDQCAFCLLGCHGLLRPMFADAFGPYRLEGRMAHVHHPCLGWAPSVRPSTDADGNTVYKNARHAIVASLAVKCTYCGEPGANIPCACSSLYESGPKGQKRGSGASVDCMQVYHYPCITAAKAVMLPSTFHVFCRRHASVEDINAVLAVDTTNIPAGFDLARELQCASCSKMQEHKDMLLCMACARRFHPRCAGMQPSSPLFAANEESVRTCWLCADCTTCSRCDSTASARSRARMACRVCGKEFHPGCAGLPKRPPSYCCEDCRRCLDCHRTANEVSSWSATFDYCTPCSQLRKRGNVCPVCKVSYRDDTPDMVLCETCDTWVHAECEGLDDAGLRRLGATDDPYHCSTCLLNEQLAAVLPCLSGTMPARRVVKALPRPTPPRYSVEPDLKIVRPVTDPEQPAPARLLEALKLLAVFQPVYFSDPRLATIDMCTLCHSAGVGGDDDLMVFCDTCCEAFHLGCLLDTVPTTYRHAWHYDFSKFRCPRCLQCQCCARALAPDLVIGRIINMDTAEHVVCSRCQTICHTECIPPSHPSMDTKHWVCPE*

>PTSG_08216T0 | PTSG_08216 | Salpingoeca rosetta hypothetical protein (506 aa)

MTERRLVADKDYIVGDVVLKDTPFVCAQFSWNRKCGYVACSHCLRSLESAEAMATRLAGQDVKLPHPELCPNNGRERVTCTQCEEVYCNEACRDKAWQLHHKVLCPGDPQRAALLTALEEAWMQVHTPPETTSAMLIVRLLAQLVSGDTTTLKALDVLCHHTKAEIGGKMHTHKLLDPPHVDHVHAMQPFIIDIVNSWSDKHDIGLAMQWATERYLELWALLGLNSLGIGSSSVSQYDIALAQASISDDERQEASDFMDNVYEVMFEHSGEFLDAEGTGIFFHQSACNHSCEANAECQFKYGDCTVEVVATTAIAKGEEVFISYLDDQLLSASRNDRWDELRRGYLFECGCSKCVAQAANPDEPDSLSDIDSSLDGDDEDDGVGDDDGDDDGDGDGDGDGDGDENAKNTAEEKANSSEDTTPTQPEQQEEELQDEEQQQEEAKQQQEQQQQQEQEQVAPPPATPATNDEDDDDADGAESVDGFEDLDIGDEEDEEDCMEEEEMFQ*

>PTSG_08257T0 | PTSG_08257 | Salpingoeca rosetta predicted protein (1543 aa)

MQSDEDRIWDTPPRGFATFVEATLGLMNTDRQQVKHTFRNVSYRILRQIQINWKQRLSNEHRVAFKQREAKADEDIAARFAQVIRERILHGELSPFNISAEGGNRGSAHTTSATVATATTTASDNGGMGAVQFSSDDTPKEANDEEEEGKQAENEPSARHTNEQQQQQQQQKQDQKNKKKKKSQHLQVKTEPLPEMAAATGQDKQQQQQEKKGSDDQKSSHPPPSHQKPTAAATPPPPLLSLSSASSLLQSSMQLPSPSNLSVSPALIPVLQAIGYPEQHQRLISALLATNPGHLEELSLEQLHHILAAVGLATPPTKRDAIAVTVDFMHRISAMVTAHSPPFASLLSPTSILQQQHHRIAATTAASVPVSPAHAHPTHFTFAGAVPFPLVSTTHAHANANAHAHMRALFAPQSSSPSSSSPAAAAAAVSMAHMLHAPQVGLGGLLSLPTLGTMQAATAGATIPAHAFGDGESKDTREDTTTRQANTPHHHDNTTRTANAAAATTTSSSTTGTGTTLRQRQQQQHGTVSRQQPPPPEERVAHRFSLIERGRIAQKSTPPVTAAGYKQIMKAMATDGDVIRCRCGVHMEDGQMIKCDACDSWQHCVCMDVVDDDALEYTCEVCSPRTLPDTIVLKDVVSEAAPHRRFFKVLHQPALDVSAGSFVYWKTPDVEELRIGRVLEIYQDGAEAEPRVIVDEYVYANAIQHAAGRRVFQNEVFLLPGTKDISVRHILSLCCVVDVQFFRVGDPAGFDAQDVYVCEFMIDKEDGNFQPKKPDKWGATSDMFELVPLPKPRSVRRRCVINSTSAKHIPASLADLPTAHMCERILGTAHVWVCGVDKCGAQFLSHKSLISHLKTQHQQTKAEPRFVIRKTLSKQMLADAIVELQRLEGLPQRFITAAGCMDATDAHAAEAAAVSTAATTRATSPSPSPTSSSSSATPTTATSPLPSPRAAKKAKTRTSQEQHQQQRAKGRKDKHVSSHTSNSSSKKARQQAHMNGSRNSSSSSTNRSAANAPGNDTTTTTITAAAAAIKSQRTKQAGSAPAPDVSHRFGSVVSLMQDPKSAAFLLAGLDLNAPVPLPIPAAAGPTTLASHRITTATPITAMPVSNPPPLGAATVEVTSVLSEGSAVTARLYHDGNVFQGVLVQCEPTDSSRLPRPSPAPDAGIEQQVLFGRRRLAFHCPERGCDVWVPTPDELIEHMNDVHALQLLFTDATLPPTREPARVRCPFYRCKQEVTSANSLISHIMTCHCVFGAKSRHAKEAMRQRQRQQLRTLNSDMTTDDGGGGGREGSLFVKTRTTTAHTLTAAAKKKNSKGKVTKRDSSIKGGAAAGDGNNAKQGREAVFGVTSALDTASPSKGPRDSTTTSSPPSSGSTVLRRAKSDPTTGARQVVFGLDVCRRMSTLVGTGRSAVAARRRDGSVVVISSGGGSSGSGGGDRGGGPRTRPATSTSQRHEDEARAESNDTNGDGGGDGGGDGGGDGDGGDDSRVEGGRRRHKRGADELLMTTRVAAPAHHGRAHRHVFINATTIRRDEGAQGTAKRAKQL*

>PTSG_08872T0 | PTSG_08872 | Salpingoeca rosetta predicted protein (933 aa)

MGDVFVEADAAAMAAGSTNGHGHAPQHSGGANTTTTTTTTTTTTTTRSSSNINTDAAGSTATRSNTTNNGGVKVMHVSKAEMDKEWPFDVIKKNIYKSSSTSRSRNREKFVCECKYDPSDTDSACGEGCLNRMLMIECEKKSCPCGSKCQNQRMQRQQFSKVEVFQTERKGKGLRAVEDIHAGQLVYEYCGEVLDLLEFEQRMRWTVNGDTRIGFFAVRDIAAGEEITFDYKYERLGETAQPCYCGAANCRGFLGAKREDSSSSRRASSSSSGTFQSRARRMARQRELTELSDKLWEICGKEGVLTNYATVQSFIVLLKDTTKCPEYFQRVLSVLLDTENKTCLEGFVAKRGLTFLKNLLETANTTEAVDVLERCLLVLGRLPVYTKNVVDSCGIMPVLETLNTHDVYDVQVQSKQLAEKWSHLKEVWVISRDTAAVDHDAQRIQIPAIRRNDFRKSKLQSLEGELSVSLDLQRGARGGDFVLVVEGKPDRVSAAIQHINAYMDTLKLRDEQRQQAEVLSRTSSTSATPAPLSASSSSTALNTSNPHMSATPQRAFHPNHQQQQQQQQQPFFSPAGSAHVMHAAPAGFAAGMGPAQQQQQPLPLYAGGGGGAHMQQPMHPSMQPQQQQQQQPSQPHQPGLLGGMGPPLPGGAMLQQLQLHAHEPKPAPCGLPCHMIPTDKIDERALQEPVHPDWAQVKDAGSSKFYYYNRFTKETTWDRPTVQHGPAGRDEPTALEKALEEMREQERRKKEEEEEEEERRKRKKEEERRRKVNKANKAARNAKAGRGEGSSSSKRAKRSTDKHRSSSSSSSSRRRRDDDDDDGDMNDDPASARAKFKKDVSATVIKFLKRFRDEKVKFGRITNSDDFKHLARKLTHTMVEKIAQRRGGELAGAVVDKAMRTKIYDFVNASMKKCGPEYQRSVLHSSALTSTS*

>PTSG_09157T0 | PTSG_09157 | Salpingoeca rosetta hypothetical protein (485 aa)

MAAIAQLYAVVREPLEPNASDDELWEDHLKVRELVQRLRRKQGTGIARDAAGTSDFTTLEAWLRAEGAIIQKVEVGVSGAGMGNGLFATETTEVGETLLQIPSKCMLSEVTAASCSKIGSFVTSDPMLSAMPNLALAVHLLAELNDPASPYRAYIATLPSSFPLPMTWSERDIARLRGLPLFARVLRLYRNVARQYCYLYTRIVEGALRPAKQDTQTTAAKNKKKNNGKKGGKKGTKNASSTSVEATSSNSVEATGSNSTSGAALTPSSFVYDDFVWAQSTEFSVENDALVFKAPESCSKGAQVCMNYGQRSNEDLLLFQGFVDGDHPADTATLIFDVDADVEARPVRLMLLKNLGIQVPGPFHVDAFPHPLDPQLLAALRVAAMGKDELTARLRDPASCADLGEEKDGVVSADCARTANALLAAALARASAAGPSGDEDKQEGKEQDDSSGEAEATASPKALVNSLWRARKAVLWSRLQHMMM*

>PTSG_09362T0 | PTSG_09362 | Salpingoeca rosetta hypothetical protein (1280 aa)

MADALMSDSVPPRRQQQGSSAATDEEDAVPNKAAVVMDTRPDDAGKAGTLGPLDPPPPPTTSATISTPSTPVPPTAAHTDDPQPALNSPSTAASSSSASSTASAPSSDAKRAKRTPDTATASSTSSTRRRSRRLLMQGSSPSPQEPHAASSPSSTSGTTGTSGQGTSSSGNATPSSPQQDSQDQAMSSQEQQQQQQQTPSLLQPVAPAPPTPLLRDLADPVDRMASKHIPSHARVGLLVWVKQKGSPFWPSMLTYEPHTGNLIKRNPNGDIRMLHVQFFGDVITRAWSSYKSAMRLWDYQQENGGWELIKERVKPSLQKAFKISMEQARKAASMSLRERILEWTCFGEYDAPPAPAVEEQQRQDEEEQSKKQKKKKSKKDRAKEVDASAPPKPKRYTSAFFYFLKEQRNNDPAPLSVTESSRVFGQKWKELSDEAKAPYLELERRDRQRYARDMEKYKELLVAHAREHGATTPSMSKLISNKPKRGRSAYIFFRKEKEEELKKICATPQELLSKLGEMWQALSEDQKQVYKDRSEKDKLRYRRELLVFETNAVVELGRQKVEQLQSPEAADEMLQHIRLTPDLTLSGPVLFTLETSKRISADSDVADTAKHVLDQWNALTQEEQQQWHAKAATTRRKAAKRAAERESQEDADAAAADGDASTTDAGEDSSVDVTKHDDYCGICGEAGNLLCCEGGCLSSYHLFCVGLSCAPQGAFVCDACTTGNHLCFACEQPGGLEGLQTCSVRNCGKKYHRACISNNPRAALKDNSFKCPLHKCANCTYPQASTYPLVRCIRCPIAYHTCCVPAGCLHENAIYLLCPKHQPVEKHAKSNICLACGDGGRLFCCDTCPAAYHQECLKDVLALTGTPSEDSPWYCHECLGGVKATEQDIVWYKLGQHRFWPARILEHTQIPEALRSKRPPGICFALNFFGTNEVSWGRHDACIRWMRGDEKSRFSTSRGKPAFLHALQEAALAYDAVQADKSAMLEAIRGRMASKPPAFQRVRTNVYTIPRKRTPGQECCCSPATDTCDDSCLNRIVHCECDPKTCPVKDKCQNRRFQRRQYPKLIPFLTQSKGWGLKAGEDIAEGQFVIEYVGEIIDATECRRRLAASQAANDHSFYILSLSGSSFVDARNKANLARFINHSCGPNCETQKWNVLGETRVGIFAKEDIPKGTELTFDYQLDSLGSRGRTTCHCGASSCRGVIEKLGREAAQSRATPLVGADGHEDICHKCRKPGTLLLCDFEGCPRVYHPECAGVNMDDEEDDDDEPWFCPAHVTPTK*

>PTSG_09393T0 | PTSG_09393 | Salpingoeca rosetta predicted protein (368 aa)

MSYQTASDQTSGSVQTVPTSSSSVSQHAQQHQKQQHQEQHHGHALRNDPHQQHQQHQQQQPQEQETMAPTATVMRRRIDEGPGSLFSALAGASGSQDSNNSDNDFLPSSSATKATRDTAPAQRAESTSGGAKARRRQSQRTQPTQPSTAKRRAASTGTGRKGSKQQQAARATTKKGGKAKADPLKQTDIRQHFGVRLTRRISETAKKEQQLQEIQNMIREERDSPHLDIVDIEGKGKGVLARRTFEKGEYVCEYAGDLITKPEAAEREQRYLTEAREQHLDEMMCYMYFLRHRGKVWCVDATHSKRIGRLINHSRSSFNLRTKLFEVDGTPHLGLVATREISKGEELLYDYGERDPSTLRAMPWLKS*

>PTSG_09637T0 | PTSG_09637 | Salpingoeca rosetta conserved hypothetical protein (1002 aa)

MKAKKASHKVNRQPAAGMVAKKKDNKPKDKKKADSATTENKKHKQKQSTTKRATTSKTSSATKPRTQRLSTSNTARSQHQHQHQQRQQQQQQQQQQQQHTNGKHHAAQPNGDGMGNRAAAANSKGKQETTQVTSTTTTTSSSSSSSSSSGGGLNGDSAVGTQPASTASTTKSSGTTSGRSTPTEGTASQAAGQRRRQRQSRRTEVEYTAMNLGDDDDLLSDIFIDTALGFQTHKMNDNYRRADVDLALLEEAMLRLTWEQDTDKTFQYLFGGYKAGTAPDTGDQAQGLSQDEHGQAVSADQQTENQSMSQQGSTASTANTAKTAPPIEPTEATNDDHAHSSTTTTTTTTNTDTSTAKQKPKAYKGKDKGKKGKGKDSKQLRGSDNTSTSRRRRHSAPDGCADSTGEPPAKRHTTTASSATNNTDNDDHDDNGGVESQMDCDAAASDTSGDDVIDADSDESLLHRGMVVRPTKTALARLIDPMFKEHAMRYIQCFLPHAGFVIVPCYRYAIASRRGAKVLATRPWRRGDTIALLCGCIADMSDGEEKAYLRPGVNDFSVMFSNRKDCSQLWLGPAAFINHDCHPTCKFTAAGTTATVTVLRDIDVNMEITCYYGDDCSQLWLGPAAFINHDCHPTCKFTAAGTTATVTVLRDIDVNMEITCYYGDNFFGPNNSHCMCETCERLGRGAFATNPGALDAVRLKQPTPRVLQTAPASIPPEVCLSIPRYMAVFVRLEDSIDKFWAPALLVPRDERDERMPSCATDTEVVRYFRDAAFGTAPVARMFPFVVDAAPYSNFSTALGPMFLMDPAVRAATKFATTGVLDERMRWSKMQLPVTANNTSATKMGKGTSNALHKASVGELSLTTGRHALTNHRIMVLGEPPIRAPCIGRVDDERVFVSIAQRRQRLPCLTITWDAQLALNRKHPVATTSRASQSRRTSRSSSRASTGGTGTGEQQQQLVQDGGGREEGGGDGEGRQDSQQTIMRPVEGDPLFCLQRRGEGTN*

>PTSG_10474T0 | PTSG_10474 | Salpingoeca rosetta conserved hypothetical protein (2502 aa)

MSSGRASRSDSSGDVGETRCVCCYRHNDESMVCCDSCNVWQHIACFEDIDTDDIPEVYLCERCKPRQVDMLRAMRIQDKKARALARRRKRMPLPEPIQATPFQETNVLELSAPAKAAVAKNIEINQLDDITEFIKDQDHHVVQHHTIVSGKQALFVQEDVPKGAPITVVSGRLCLRSELAKQYKLEDTFLPYVIFFDTAGLSLAVDATTRGSIARYVRRACDASASLGVFQARGRLALALTASRNLPKGAEVTIPFDVDWASVSYLLDCACGSSLCQVAKAMKRRQRSLAAKGPRRPQPKQQRQPKQPKQPKQPKQQSKSVSSVPESPAGTPTARMSREERKLMMSLRRIDEACGEVAKQQQQQHTPAGGSEARASEGRRSSGGKGRRASSAKPASGGTKGRRPASLHLQMNPLADAAGRLPSSPSLISASASASALTPGYNYDARAMDAFPDPPHGRSGGGGGGVGRRSRRRRNSTPHKEELENYNTISSASQTPFAAYHNDACEILSDLLFQGLKGRISKAARPFEYSSPHVGRVVAVAFERAPQGGVYARKKYMSQWMPECVPSLDTDDLTPSTTPVASTAAATAPMTPTTTTTPSTTHPQQQSHGQQAKMASNPSTNAAPNAKTNAHSSTTANAGGDGEAARRVRFAIDGSRSSPNCGGVGPSRLGDDDNDNDNDDCVSNSSTSTRRNSAQDASATNTTTNNTTTLSSSTGTTGATTHTHTNNANASTTSSTAASALSWSWWWSKESATPGMTAEPAAQPVPFVSFKERLRRSLRTVQAAHPATTADGGRDDGQDGAVTGTMNAAPAAATTTAMDTAPDAAHSGVKAESESAAGTPRTQQQLQQHQQPLLPQQQQQQQQQQQRVVQVKQEPPMLGVKTVAAPLVTTPQTGATSASGITAGSSATTTTTTTATTNVVAPGAVVSQMPSRPAVHRNVHGLRVNASAPPSIVDGATSPGVSSPLSAQGSSVFLPDSPASSSPAAPSSPSSLPSPMLLQQQQQQQQQQQQQQQQQQQQQQQQQQQQQQQQPDHHPHQKHKSGRVAELKAEAIPTPSNAAAAAAAAKSATPSSPAAPASTAARGSPASGRKRKLSLADYLKRRGLSKPTPPSSPSPSSSSSSSLASATASATATKSPAAAPKTTSPAVATSHGSGAASNTKAAPASTEAAPASTKAAPASTPSATMPATTTKAPATPAKASSATTGVAAPSTATPVAASTTPTATTAATTTTTTPATAAAAAAAAAAAADRAHDSSQPPQAAAPAATATTAAKPEPSTKTPKQTPKPTAAKPKSMAKAAEAAAVPQSRAAKDDAKTASGAVVTPKEASSDAGPASSSNTTRATAPPPVSDEGNKEQQPVAAKSQEATQLPQTRKKVQAQQKQQPPYTQDDRAGVASKVQEHKQSTKRPQEEQPQQQQPEQQQQQPGASEKTQEADAKALSSSSAATAALVTDEAKQAAVKQTKKQDEFTASGTGTADTTTARKTKTLHTTAAAAAAAGGGADVVVEQTQSAAQQQEAQQEQQQQQKVQQECDEKAQESSPQQRKSGATPVHTAQPALATATSTHATLPAVKTTATAPAPTYDVDGDRDDRADRAGRDDAGEQPRKRAKQRAPSPSDDGATAVERNARAGAMTAAVAGGDQVKQSAAAPASSGADNAAATGAAGDDENADDEFKPAPPPPAKVTTPPATPAAKTSAAQPSPPSATEQPAPPAKAGRGDDDDDDDVAAAAADNPALAETAATATEAPHETPTATTSQPSAVRAALADSGKRDVPADTAGVDRQPSLDAGSEQPPKRAKRSAPPTDAQEHTSQREQQQQQRRQRRRRMGSTASSSSGRPSRESSRPRSRRPSRAHTPTEDRSATTTTMSATSAAAATTAATTATTTASTSRAGSSRASRASSRSGSVSRSGSRRRWRRNMSPPPPPPPPHDRSRDRDGDQVPLDRERDRGDRDRERSMRDLEPGRDRERERERGSRMPPPASASTSTTSASSAARPGGDRDWPRDRDRDKDRDRDRDRDRGYDRRLWSRLGDTNGPPSSSSSPPPPPHPAAGTWPRDRDRDRDRDRDRYGGRDHMWGRDSGPPPPPGPPPPGGPPLPYERDREREREREWDLRDRGRERDWGDRGGVGGWERMHDRPGWDRDRDRDRDRERGRDLMRDPRDWDRDRDRDSDRERDREWDRDRRDMRRYGRAPPPPPGLRDDGRRWEEGPPPPGLAGPPGPAPPAGPPPHHHPMYGPPPPSYRRPYPPHHPYPPPHHHPHPHHHHPRHPAGRGASPPATPPRAPSIPSSSASPQRDDGRQGIHARPPPAARAASLSPGRTPPPAATSGGSGGGGSEGGRGRGRGRFTSRFRRGGGWRGRDAELDRERERDWDRDRDRDPRDWDLREEWLERRRFSPPPYRYPTGDDGARPRKADDRHDAAPPPPPPPPPPPPGAHAYRRDYGSPLRLRRRSRSASPPPPPPPPPPLPSSSTSSVLQGEGRGGRYWSVPRDERRERSASPRRHPQRR*

***Schizosaccharomyces pombe*  (Fungi)**

>gi|19111978|ref|NP_595186.1| histone H3 methyltransferase Clr4 [Schizosaccharomyces pombe 972h-]

MSPKQEEYEVERIVDEKLDRNGAVKLYRIRWLNYSSRSDTWEPPENLSGCSAVLAEWKRRKRRLKGSNSDSDSPHHASNPHPNSRQKHQHQTSKSVPRSQRFSRELNVKKENKKVFSSQTTKRQSRKQSTALTTNDTSIILDDSLHTNSKKLGKTRNEVKEESQKRELVSNSIKEATSPKTSSILTKPRNPSKLDSYTHLSFYEKRELFRKKLREIEGPEVTLVNEVDDEPCPSLDFQFISQYRLTQGVIPPDPNFQSGCNCSSLGGCDLNNPSRCECLDDLDEPTHFAYDAQGRVRADTGAVIYECNSFCSCSMECPNRVVQRGRTLPLEIFKTKEKGWGVRSLRFAPAGTFITCYLGEVITSAEAAKRDKNYDDDGITYLFDLDMFDDASEYTVDAQNYGDVSRFFNHSCSPNIAIYSAVRNHGFRTIYDLAFFAIKDIQPLEELTFDYAGAKDFSPVQSQKSQQNRISKLRRQCKCGSANCRGWLFG

>gi|19115749|ref|NP_594837.1| histone lysine methyltransferase Set3 [Schizosaccharomyces pombe 972h-]

MWKIRCVCPFEDDDGFTIQCESCEVWQHAVCVNIDANNVPEKYFCEQCQPRPIDADKAHKIQLARLQREEEQSRILSRSRSSNNKRRTSFGKNGASPTHSASPRQGNNTGANGALFSQSTNSSNSGSYRNSVTGATLPNAHAPHSQNRRRRSNHLNNPPEAPITEASNEYVYSFHLEYVPLESNTFSASALEYSKNLDLKNLDESEVLMDGCQVVPISSSKFCCSRFGLVSTCEIPPNTPIMEVKGRVCTQNEYKSDPKNQYNILGAPKPHVFFDSNSQLVVDSRVAGSKARFARKGCQSNSVVSSVYMNGSNSVPRFILYSTTHIAPETEIIGDWTLDISHPFRQFAPGMSRPSFNMEELELLSEVLSTFLSFNECASQDKKNCVFSRVTKYIKAARRASTANRVSVAKDRLSLTPSSTPSTPSPAESLPQPSNPTSVYAKSLKEFWLDKYRLSILQKWPAVKSLPTESVGIDVVMEPKLQPSVKEKKPTKDLQSPLPSVEEDSSNRDKKTDIADLHTDSKVGIADVLSPISPDAALQSDGPLKKAKEPEESSITPTTPPSFNVGESLSRRSASPLQHPRTSPDMLDKTSPCKRGLGTITTVHKKHGSVDHLPSVKRRRSIANDFHGKPDYNKRSLSIERKPEAFKTKGDRPHKVHPSFHRNSDSKLKLEPSSKEKSGSMFFNTLRTVKDKSHVHDTQRSSDVNFSRQNGTRSHSPSVSPVGFSFDKSPVTTPPLPTAPAPVITSRHALVNNQFPTNNPNILDHKANNGDDISNALNTSRSENKPNSNLVQGSVVKPSNTSASALPTSAPKKLSLSEYRQRRQQNILHQQSKDNQAHGDTARPHTVPAATVSNPSFTR

>gi|19115892|ref|NP_594980.1| histone lysine methyltransferase Set2 [Schizosaccharomyces pombe 972h-]

MQTASSLSVLTPLNEENVDRKSSWSKDTIAVQAVGSSPSSSSSHDFESKEDAEGMNKDESAPSPSTSSPSSASSRSQSKYVRKEALPPQLFHHLDSAKDKALTTFEEIQECQYASANIGKPPENEAMICDCRPHWVDGVNVACGHGSNCINRMTSIECTDEDNVCGPSCQNQRFQRHEFAKVDVFLTEKKGFGLRADANLPKDTFVYEYIGEVIPEQKFRKRMRQYDSEGIKHFYFMMLQKGEYIDATKRGSLARFCNHSCRPNCYVDKWMVGDKLRMGIFCKRDIIRGEELTFDYNVDRYGAQAQPCYCGEPCCVGYIGGKTQTEAQSKLPENVREALGIEDEEDSWENITARRQRRKKGIDETSKIIEEVQPTPLTSESATKVIGVLLQTKDDLLTRKLMERIFLTSDPSVCRSIIALRGYNIFGLMLKKFSIDIEFILRSIKTMLSWPRLTRNKIQDSNIEPVVQEFCDHENEEVKDHAKTLLKEWESLEIAYRIPRRKPGQVAPQSTNAEPSNNQSNPPLRDQEPQRGDKGDIKSAINNSTEDLSKKHPALHSSRPSDSRSRSKFGNDYQSHSKHNLFRKNSFPKRRRLSNSDTPSETTTPNNEQEQVSNQANKVDLNKIISAAMESVNQKNVLKAQKEEEERIAQQKREEKRRLAYEESLKRHAKKLHEKKTKSSQDATIDHHLTSHSPESIAFKAVLAKFFANKTARYQEKLGKAEFKLRVKKMTEIILKKHIQLVLSKKEKALPDELSDSQQRKLRVWAFRYLDTVVSRSGTATTTPTDSPSIGESPKKAA

>gi|19114522|ref|NP_593610.1| lysine methyltransferase Set8 (predicted) [Schizosaccharomyces pombe 972h-]

MDIKYNELVNQFAPGAKQITIKKIRKKGNGIFSLNRYTSGTVLLEVPLENIICRKTVEQFRNSCDKFASIATLEEWNDMSFRTQAMLFLCYLWLGIQPRTNKWDKFLTVLPLSINTPAQWPEKEVYSLQGTSIFNPVCVKRKILQQEWLSLNQRYSDSWPSKITLPKWVHADALFHSRCLESPFKDPVLAPVIDLCNHSSKSNAKWSFSEDAMQLYLDKDIDENEEVTINYGSEKGSAEFLFSYGFLPEPEGDRITNVMKLLIPEDSNDSLDLAKRRSCKTPPMIEFVSDSSGELWWHAPFLFFSVLNVEDFTNFKMVCDESKAQTVDWEFEGQKCSVEDLPKLVQLSPKRDLYILRVFCLAEQLADAALNTNIENMYNPTERRSESVELLKRESFLLKKVLLYLRDVISKLLKSKVVVEFIHSQTIES

>gi|68043673|ref|NP_001019057.1| hypothetical protein SPBC16C6.01c [Schizosaccharomyces pombe 972h-]

MNNSKQFLKYQPLLEWLAKHEAYISPKLYIASSGVAGDGIFSTFDIDELEVLAKIPRRIILSPRNSRFGDSLYTHFNESNRSDDINFDNRDQVGLVMLVITVILENITDSPWNAYLNTLDETCMPDSPLLWKDKTCLEGTSMLDVINTNLRVYKNQYDQLVRPYFYKHADLKQLCPKWNQYLETCVLVQSRCFYVNSYYGLSLIPFFDIFNHKSGPAIASLHCQESNDHKGDIKIEFISFQYIRKMSEIFNSFGNFAADELFTQYGFIDTACKVWRVDMTMIAYETNRNFYMEWIHKKRIINTQELLVTPTTYANDKSETLRSVMIRQPMDLYIITDHGPSYGLYLYLFFCIYKIKFQKCDMNIVMLTKYFNEIWAVFIAHKEGEKEKVVAQLSGFSFYC

>gi|19113306|ref|NP_596514.1| histone lysine methyltransferase Set6 (predicted) [Schizosaccharomyces pombe 972h-]

MDAPLIASVILPEFGKGTVATDNIPIGKIIIRKRVDILSLDSANLTRTCSTCTEEKVKTQRCAACKIIHYCSKGCQKADWPFHKLECKALQASKQNGILPSVCRLLIRLYLLWQKNPAIIEPMEGHQNEFQAVSSSWSDAELIASAASHYTQIYQAELFQKLFCRLAVNAMNLVTSSFDSLGMCLDTILCRLNHSCDPNCQIIFDGAIVQLVSKRDIKKDEQLFISYIDIRLPKSIRQKQLLKKYFFSCYCPRCENDHTTKETDGSKWMGRLRNSKSLMKNLAMARDLWSCGWKQTAFPWSNLLHHIKLGMLDESNFNGAFAALYLKSSADEFLDALHVVDEYQLLLLGKQVAMEVKHLMFPNDKPLEMEFPSSSQPQQTVPTNNSLFLLKNIPSFGGLHHCILGRYSISLDDFVLWLLDRAQRLQQAVRISHPSTTFCSNVENDIKEIFEVCKDYCMLHVQNNLKAFEEKLWAACKDFLVTY

>gi|19075849|ref|NP_588349.1| ribosomal protein lysine methyltransferase Set11 [Schizosaccharomyces pombe 972h-]

MSNKQNIESEVSWVKSKGAFVHPSLEFSVIPDAGSCVLANNDINENTVLLKLPPNILINKRTCSRYSFRDKLTSFQFLSWLISEDVHSNLEISPYYTKALPQGFSFHPVTLTSDHPLWSILPDEVRNSLLERKNVMAFDYEQVKKFVSVDQPTFQWGWLCVNTRCLYYDTGSKNTEDHLTLAPIFEYFNHSPEAQTALINTRGTITIKSTRRIDKGEQIFLCYGPHGNDKLFTEYGFCLSNNPNISIQLDRFIEFDKWQQSFLQDHGYWNDYTCSLHGASFRTLVGVRTLLVSPSEKLNDASYDQTRRVLQYINGFSDGSRDRQDVEDYLKKVLQELLCEAEECKEKVKGISDGSYVFICAEQLWKDRIMCCQYLMEHSFE

>gi|19075913|ref|NP_588413.1| histone lysine methyltransferase Set5 (predicted) [Schizosaccharomyces pombe 972h-]

MNPYETEIYKVVPIPNKGMGMIAKVKIPVGTRIFAETPLIRTKSDAKEIEEALSTKTKEEQEAFHRLFNAHPDTMGPFLGPFYSNALTIDETKGGMFLLGSRMNHDCSPNVKHTWNPRLDQVTVHAVRDIEAGEEILTTYIDLHKSHTERQKILLEHFGFKCYCSVCSVEERKIRKISDLRRKQLAYYDRTMAKMCIVNPRGALRALRHRIHIAHEELLFGRLDIIALLDAFRLCVIHGDFERASIFAKKGTKAISLYEGTDSEKYLKISKYVENPRSHALAEGVPALPLFLEEDDELSDLEDNLWGCKLEEDVYSDTD

>gi|19075861|ref|NP_588361.1| histone lysine methyltransferase Set7 (predicted) [Schizosaccharomyces pombe 972h-]

MRIPVIRSPLEIRDTERKGRGVFALEPIPAQTCIEISPVLMFSKEEYEQHGQYTVLNEYTYVWSEGKQGLALGLGSMFNHDRHPNVYWKKDNRNNYISYYTLREIKTNEELCISYGDHLWFEDEASSASRISPNEENEDFPLQNISL

>gi|19075312|ref|NP_587812.1| histone lysine methyltransferase Set1 [Schizosaccharomyces pombe 972h-]

MDFNTSTRSKSQPVQRNNYKVLYDPELGIKENLGRKIIYRFNGVSKPPLVVRDPRLKNPIYARGIPKSGRPFLKSLQTINYDYNENSLGPEPPTQVFVSNISPLVTSEQLRYHFKSFGEVFDLDLKLNPYTGTSLGLCCISFDKRSSISVAAHSAKIAVQQANGLRFSGKPLSVVLDRDGSLCEEAFKKALNAVEKQFQEETLQKQRFEREDESSRQKLSAAMNEDIPPWRQPSKNSQTLSNGDLQHSKVQNVDQKSGFLTSSETDVPKNINDYIYLLIDDRFVPPDRVYYTDIKHHFRKFLYEKIYMNKDGFYITFNNYREASNCYRALDRTYVQNCRIKLKFHDIPSRTKEDGKKSAVRRVVLPPEEAYAEATSVVLRDLEAALLRDVKSKIIGPAIFKYLHSMPKPSVKEELQENLLVSSTSVPDVPLKIESTVGKLPSLPKFKKRVDSSKMNLSAGSKTKSKLQRRRRRRHEARPLHYQLNQMYNSSASEAESDQELLLSSGDERVERGKIGSIKSVKSDEATPVFSDTSDENDKFHRFRTKSKISKKKYEKMEVDYTSSSETESDASILSPSAAIPKSGSAIKDELISPKKEIDEVLALAPKWRINEFDETGSVYYGALPYNYPEDDVLLDLDGLQYLVKNDEDYSYLQEALKDEPLMDINDPNFWAYERKSCKFKNGDVKYGDTAILPEPKGYFRSNTSGSAKSEGYYIIPTTEKSLYLPLRNRSTIDTISHSTSRITSRMNRVNNRRLAAGVEKSQLPAEADLLRFNALKARKKQLHFGPSRIHTLGLFAMENIDKNDMVIEYIGEIIRQRVADNREKNYVREGIGDSYLFRIDEDVIVDATKKGNIARFINHSCAPNCIARIIRVEGKRKIVIYADRDIMHGEELTYDYKFPEEADKIPCLCGAPTCRGYLN

>gi|19075578|ref|NP_588078.1| histone lysine methyltransferase Set9 [Schizosaccharomyces pombe 972h-]

MRQTNTHLETFSLFDDVCTCLLVDKVFYWSQIHKVRKLVDRSIERMESCSIINIITKYIIEQTDLDQAAKNILQFRELDPLLRRLSSTSLLAFTRHLKYYLSLYLPSCKFEICSTNQYFSSSKPEACVIARESINAGEDITDLCGTIIKLSPKEERNIGIGKDFSILHSSRLDSMCLFLGPARFVNHDCNANCRFNTSGKRIWLRCVRDIKPGEEITTFYSSNYFGLENCECLCVSCERMGINGFKKLFHTSATSTSCSSKSSSDVSDLSSLPQSNRYVISEEDRSFLNIWDSGGELSDASSSDLDEEFSLFIPRHKKRVWSREKRLLSEMAITNHSPLLNVDDYRKFREDLWKKRHGKRKVYQCSNCSQTFINEDIQNSSAFCPKCIRHSKLFSLPWPCRHKVNRELKLEKEKEINTKRNLVTSSHSMSLRHKKAVDYQS

***Saccharomyces cerevisiae*  (Fungi)**

>gi|6321911|ref|NP_011987.1| Set1p [Saccharomyces cerevisiae S288c]

MSNYYRRAHASSGSYRQPQEQPQYSRSGHYQYSNGHSHQQYSSQYNQRRRYNHNDGTRRRYNDDRPHSSNNASTRQYYATNNSQSGPYVNKKSDISSRRGMSQSRYSNSNVHNTLASSSGSLPTESALLLQQRPPSVLRYNTDNLKSKFHYFDPIKGEFFNKDKMLSWKATDKEFSETGYYVVKELQDGQFKFKIKHRHPEIKASDPRNENGIMTSGKVATHRKCRNSLILLPRISYDRYSLGPPPSCEIVVYPAQDSTTTNIQDISIKNYFKKYGEISHFEAFNDPNSALPLHVYLIKYASSDGKINDAAKAAFSAVRKHESSGCFIMGFKFEVILNKHSILNNIISKFVEINVKKLQKLQENLKKAKEKEAENEKAKELQGKDITLPKEPKVDTLSHSSGSEKRIPYDLLGVVNNRPVLHVSKIFVAKHRFCVEDFKYKLRGYRCAKFIDHPTGIYIIFNDIAHAQTCSNAESGNLTIMSRSRRIPILIKFHLILPRFQNRTRFNKSSSSSNSTNVPIKYESKEEFIEATAKQILKDLEKTLHVDIKKRLIGPTVFDALDHANFPELLAKRELKEKEKRQQIASKIAEDELKRKEEAKRDFDLFGLYGGYAKSNKRNLKRHNSLALDHTSLKRKKLSNGIKPMAHLLNEETDSKETTPLNDEGITRVSKEHDEEDENMTSSSSEEEEEEAPDKKFKSESEPTTPESDHLHGIKPLVPDQNGSSDVLDASSMYKPTATEIPEPVYPPEEYDLKYSQTLSSMDLQNAIKDEEDMLILKQLLSTYTPTVTPETSAALEYKIWQSRRKVLEEEKASDWQIELNGTLFDSELQPGSSFKAEGFRKIADKLKINYLPHRRRVHQPLNTVNIHNERNEYTPELCQREESSNKEPSDSVPQEVSSSRDNRASNRRFQQDIEAQKAAIGTESELLSLNQLNKRKKPVMFARSAIHNWGLYALDSIAAKEMIIEYVGERIRQPVAEMREKRYLKNGIGSSYLFRVDENTVIDATKKGGIARFINHCCDPNCTAKIIKVGGRRRIVIYALRDIAASEELTYDYKFEREKDDEERLPCLCGAPNCKGFLN

>gi|330443605|ref|NP_012367.2| Set2p [Saccharomyces cerevisiae S288c]

MSKNQSVSASEDEKEILNNNAEGHKPQRLFDQEPDLTEEALTKFENLDDCIYANKRIGTFKNNDFMECDCYEEFSDGVNHACDEDSDCINRLTLIECVNDLCSSCGNDCQNQRFQKKQYAPIAIFKTKHKGYGVRAEQDIEANQFIYEYKGEVIEEMEFRDRLIDYDQRHFKHFYFMMLQNGEFIDATIKGSLARFCNHSCSPNAYVNKWVVKDKLRMGIFAQRKILKGEEITFDYNVDRYGAQAQKCYCEEPNCIGFLGGKTQTDAASLLPQNIADALGVTVSMEKKWLKLKKLSGEPIIKNENENINIEFLQSLEVQPIDSPVDVTKIMSVLLQQDNKIIASKLLKRLFTIDDDSLRHQAIKLHGYTCFSKMLKLFITEQPQVDGKGNETEEDDIKFIKGILDFLLELPKTTRNGIESSQIDNVVKTLPAKFPFLKPNCDELLEKWSKFETYKRITKKDINVAASKMIDLRRVRLPPGWEIIHENGRPLYYNAEQKTKLHYPPSGSSKVFSSRSNTQVNSPSSSGIPKTPGALDSKKHKLSDEEYERKKQKRLEYERIALERAKQEELESLKQKLKLENERKSVLEDIIAEANKQKELQKEEAKKLVEAKEAKRLKRKTVSQSQRLEHNWNKFFASFVPNLIKKNPQSKQFDHENIKQCAKDIVKILTTKELKKDSSRAPPDDLTKGKRHKVKEFINSYMDKIILKKKQKKALALSSASTRMSSPPPSTSS

>gi|398365041|ref|NP_012954.3| Set3p [Saccharomyces cerevisiae S288c]

MSVPNSKEQSLLDDASTLLLFSKGKKRAEEASKIGSKTDTIEHDESHEREKKGAIEMAAAALATASTVSLPLKKATEQSAAEAATSTAAKEETENQPQKQPQWPVPDSYIVDPDAGIITCICDLNDDDGFTIQCDHCNRWQHAICYGIKDIGMAPDDYLCNSCDPREVDINLARKIQQERINVKTVEPSSSNNSASNKNNGRDRASSTTISDVGDSFSTDQDNTNHRDKRRKRNPSNNSIDSKNESASVNSSDGLTSMPKKKEHFLSAKDAYGAIYLPLKDNVFKSDLIEPFLNKHMDDNWVIQYPHKTFKSVSIEVKPYADIAYSRTYPGFTKLGVYLKKDCIKGDFIQEILGELDFYKNYLTDPRNHYRIWGTAKRRVIFHSHWPIYIDARLSGNSTRYLRRSCQPNVELVTIKLQDTDNRNDKSSGRKSSRIKFVLRALRDISEDEELYIKWQWDSKHPILKLIKGMTIDSLDDLERYGLINSVETILSNGECGCGNNSKDCYLLKVKRYAQSLYKSVKSRGKMNNRYKLNEILNQYNCKKRREPPILHRLEEKAQNTIERAPILLNNFYRQKFLNRNNGPKIPQKNTIDSTNNPDDIAKPFKFALFAQHSSNISVPKKNETSEKPLIITKSTDYDESHITNIEELPIPVLLPINKTSRQTANDVEESQSKNEHKLSRTPSLSNFNKELSKEAQHSQAKTKEIMTEASVNSRRESTPESIMHLSDFSSSQLHSKKKLSFADYRKKLLK

>gi|6322356|ref|NP_012430.1| Set4p [Saccharomyces cerevisiae S288c]

MTSPESLSSRHIRQGRTYTTTDKVISRSSSYSSNSSMSKDYGDHTPLSVSSAASETLPSPQYMPIRTFNTMPTAGPTPLHLFQNDRGIFNHHSSSGSSKTASTNKRGIAAAVALATAATIPFPLKKQNQDDNSKVSVTHNESSKENKITPSMRAEDNKPKNGCICGSSDSKDELFIQCNKCKTWQHKLCYAFKKSDPIKRDFVCKRCDSDTKVQVNQVKPMIFPRKMGDERLFQFSSIVTTSASNTNQHQQSVNNIEEQPKKRQLHYTAPTTENSNSIRKKLRQEKLVVSSHFLKPLLNEVSSSNDTEFKAITISEYKDKYVKMFIDNHYDDDWVVCSNWESSRSADIEVRKSSNERDFGVFAADSCVKGELIQEYLGKIDFQKNYQTDPNNDYRLMGTTKPKVLFHPHWPLYIDSRETGGLTRYIRRSCEPNVELVTVRPLDEKPRGDNDCRVKFVLRAIRDIRKGEEISVEWQWDLRNPIWEIINASKDLDSLPDPDKFWLMGSIKTILTNCDCACGYLGHNCPITKIKNFSEEFMRNTKESLSNKSYFNTIMHNCKP

>gi|6322001|ref|NP_012077.1| Set5p [Saccharomyces cerevisiae S288c]

MTLTIKIGTLNDSDQSAVHNGTENGSDFRKITPTEEEICDDVVLLWKEEPGTEDATIQHLYDRITERNQSWKLSASRFRKILNEHHLYDTDLETVSLYKDKIHFPKALDSDAKVEVKFIDDEHGRGLFAKRDFSKGQIILKENKPIVYIPPLDKLFLISNGKACARCGKALYDLTQHKIMVHYLDCEVCKAIWCSEKCKKAHASLHELLYHSWRSNRIDILHAGNWKRFVNYCEKYCFTAAFSVGLIYGSMLLDTTGEVKEQWQKLASISQRERIKLRDASGIGSTFSLLNGTTVHTEEESDNGTKKGVEKNIDDETVWEKCYELFCGAFPKASEEIDFEKFLTMIGTFNINQYNGQVYHWISFINHDCEPNAYIEQVEEHEELRLHARKPIKKGEQIRITYVNPLHGVRLRRRELRVNWGFLCQCDRCQNELSTFERVPNLEKKNADANLGVEKIDSNDSSEDGSKKSTGNRKSSMREAQPDLKEILKNGKEFELDIPETVDTQGNVRKTSVRFDSNVSVAVDER

>gi|6325092|ref|NP_015160.1| Set6p [Saccharomyces cerevisiae S288c]

MTIDGDVHEISPFFQVRQTKWGGRACFSNGNIPKGTTVLQVSNFTGTSISYEFRKEVCHNCFAYANAKTMKYKLNYDYLRDLVCNAHYQINPKKFLGAGLWFCSEHCRTSYLQIPNIIELIECYEILLHHFPSMLKRYNYTSEQEEKLNSILISENVIQSSWDEIESKWIPRINNMKSAKRINQLPPTCEDEYCCIRFVCESLFNLKYMDPQCITYRAFNMLQSNELSKISKFPVLLHFQKLVFQTLYILLPSHLHRMLSIPLLRHILGTEYGNAFGLWQEGEASDSREYFGYWVFPEASYFNHSCNPNITKYRKGNSMLFTMNRDIKKDEQICIDYSGVLDLPTVKRRAFLADSWFFDCACERCKSELQSVH

>gi|6320463|ref|NP_010543.1| Rkm4p [Saccharomyces cerevisiae S288c]

MDDFSRDTENFVCWLKTTAEIEVSPKIEIKDLCCDNQGRAVVATQKIKKDETLFKIPRSSVLSVTTSQLIKDYPSLKDKFLNETGSWEGLIICILYEMEVLQERSRWAPYFKVWNKPSDMNALIFWDDNELQLLKPSLVLERIGKKEAKEMHERIIKSIKQIGGEFSRVATSFEFDNFAYIASIILSYSFDLEMQDSSVNENEEEETSEEELENERYLKSMIPLADMLNADTSKCNANLTYDSNCLKMVALRDIEKNEQVYNIYGEHPNSELLRRYGYVEWDGSKYDFGEVLLENIVEALKETFETNTEFLDRCIDILRNNANIQEFLEGEEIVLDSYDCYNNGELLPQLILLVQILTILCQIPGLCKLDIKAMERQVERIVKKCLQLIEGARATTNCSATWKRCIMKRLADYPIKKCVSIEKPSKGNSLTREELRDVMARRVLKSEIDSLQVCEETIDKNYKVIPDEKLLTNILKRKLTEEEKSSVKRPCVKK

***Sphaeroforma arctica*  (Ichthyosporea)**

>SARC_00017T0 | SARC_00017 | Sphaeroforma arctica JP610 hypothetical protein (736 aa)

MKSWKVDQDMMSFLQLVGNIAMRQRLESSDSVHTTTAPPKPVPIATGGDGTQDNDMRIIDEIGHTASIRPCYNDCSAKVWAAKSYRTVSDSAKSEPGIHAQNAIVEKDCEGAHAEYSAGQCDDSKRNVLSETGTDFGLLSTSPETLEGALEESSTDTKTETDVETYEPARCYCYDFMPYVPASDIISEQRLAVVSEEEKARQQTKLLARGLGAKSRTVSLLQTPRKDITPLSHTRKVSFVVAPDDKPHDTKAKVDQKRAVKIEGASSSGFGGLFAAIADMSVTTKSDDSVDEEDGSVEELDDSGIEDTSGDGLGGMIDRKDHQDNLKEGMGKDEDRVCLDAESIDREIRTDSRISHSLVDSGDRPGGESTAMQRVSNGTTSGSGMSMEARVEQHIREVQGHKAVPAISFSITDQDGDVQSIFRPSDGRGSQDKVMPSALRQKKGRSVSYSARSTTRSDGGERPSDSELRIAIPVRDDSKDYVNIHLGVVVAPEAKDFNLLESHHKTYDKDRVRDLRKPLQQLMSAMNTIFEAAPGAVVNAMPLTPAVASETVTTDSSAASANHSPARATATVEACPVSFDELLAIAGRIESNCFGYRNRKESVHTQLGRMVVPTASYFNHSCRPNVTAEFADSNLASFVAKRKIYDGQEVCISYIDENLPRSERQFRLKDSYLFHCVCAKCIEPDSQKGNPSWSKSVNAYAKPPSNQPKKNNKAINKATNKKKKGAKKGRQRQDE*

>SARC_00311T0 | SARC_00311 | Sphaeroforma arctica JP610 hypothetical protein (727 aa)

MDVECTEKCVVERDFTTTGRGLATERDVIAGEVLLSVPVSKCWTVAAAKASEELKHLSDAQLSNQSLVSLHLLVERNKGGDSSRAAHISCLPKDFDLPIFWDKDELATLEGSPWHDISISVRADTCKQFEELSAIPQVEKILKAYTIDLDAYLWARSVLWSRMAEMTKPDGERIEILAPWFDLFNHSPDVPSGDCFKIEGENVVVRATKDYKEGEQAFISYGDLPNGILLLTHGFTLEDNEYNSLPFYLNISNLSENKSAALMLCAPDITDKREDPRLAQFEYITVPDETGGDFCAKHLLSAQIPLPDALLNMVKIERLTPAQLASILEQDGQRGLLAKLEADQSLADGNDILAYITLHKTYTDMISALDMPQTEDKNTSDTTQRRRAHTLAVKQSERKILQNGVQLCEERLMKHWNRQVSNMDQINRCGCDGCMAKGSPYVHTLLANQPQMDANQEATVNVLQTLMSASVSDALHVQTAHLIFMFVHELWSHPLARSVVLLEDACPSKVSAGCDRGLRPTDLILTECPEADIYIEKMRLWSSYLLAQLITTATVSLSDMNTMRMEMYNLRSQGLIIPTGSALDTVVNLVRDDDECDGVVAVGAAKCHVWSTQLALKDIPIVTVETLNELATYPKHALLLVNPDDAGKGMAGWSCVNKDPKRRLLLTVGEWADSTLGAYATGMPVHGQSFSNMSQQAVEKNYDMVHSARLAVWPVYMDRLSVWRHK*

>SARC_00510T0 | SARC_00510 | Sphaeroforma arctica JP610 hypothetical protein (416 aa)

MMFVRDMGRGRGRAIFANRTILRGEVVLDAKPYVSVPSSTTSTCMECWQYVSDNNCTLKCKKCHQAVFCSDACAEKGSTRHALECAALKKLCCSSVVKDKGSRQMMHLALRAMLGVTALKFSAVHIDSRRRLSDENMIASTGPTPLMGPSTCSQIENNVMIDIGTAGAVNDRESVDRKNGKCLIEGIVSGQKGMESVEALEDNRMAFSTQQINQLTTAAKVLLKCAPSLSGEWSIRRVVSLLCKQECNVHGVWGGPDIGWRGVCTCIGMALIPEASYFNHSCTPTLVAIRSAARMKMRSDSLHNPVEVSDIDFCKINCQGTSSSADLSYVALHTIQAGEELTISYVSCKQTTEERQKALESYMFKCTCAACAGKARTHVMRNFLNRYACSKSTCPGLLVPFCGKDDSICAICGLR*

>SARC_00798T1 | SARC_00798 | Sphaeroforma arctica JP610 hypothetical protein (420 aa)

MSSNWEVSTTSHKGRYIRAKLPLTRGTLVLKSDPFAYTVTNKQKALRCSACLKASEGLRGCGRCKKLYFCNSQCQRRAWPRHKQECKAICASQKVALDAMSIVCHAVWRAAADKKTLFDSDFGVLQSNRRLQSTDDDVAQAPTAMLIRTYLESSGIEASQIPSIGDAMNALSVIKTNSFTILDDEMMECGVGIYPLASNFNHSCDPNVVITFNGFELQARLIRNVHTGDEMTISYIDNAKPREERRNQLRKQYYFNCDCSLCGDTQMDDTHLAFRCQQRVGGNQCDAMVPFDDGKQLVGDDALCSQGHVQSITGDRMNSTAQHLDDKITEAKSVSESDRAIQMCDEVMCTGRKILFKYNTQLTAISSATCDIAINHGRFDKAYDYGMDGTRGLYRILSDGFDPRLGIQEKGRGICERGG*

>SARC_00855T0 | SARC_00855 | Sphaeroforma arctica JP610 hypothetical protein (1755 aa)

MSSIGRTRSTGIRSSARRIREALLSDGQRTPETLESGAVVNVSTINEPVNTDTSEKEDTDINMSESIVDAENTVGERQVDTDDMEVDIDNQQDRRTKRGKPKSVRSQRSQDTQTLVHEDIDTIPGSVVDSVDQSVEEDGIKMVEINDISALTSMATENDIPTSDTIPQEHTRAQTMDTDDIADTTETQDVSAEKGERGVREGSLALHDDGNVAQEGMKYKSETQALFDDSSDESDEDVPLTLGSGSSKNKSTSAHNAADSDGDVNRDRFNRTDSEHNERGSDAQPGDGSEQESSGSAYTKAHDDTHTVVDGDSHTKGESDHAYASSPTGRPAKGSKARAEAKGKVVRKTSKPRPRKDKEGVDGVGSHKGGSRVGSSKDKKLSRPKKGMADKKALEDKIANTLNTIYAESKVKQAPRIVKISGHYTALNKVGRKDESVAEMKEIPLDWDGETVNTGMKRDDTGTKSSGAGTTHNSSTTNPKKRTKRERSSDTAVSGSSVRTKPAKVSVANEWGDTDCLSYIDSYKTVVGITSAFGRDMGSGEGSGKARRASSSDTSSPRGSRSKKEVRSRREGDGGGAAMGLGSDGVEGHVKKLTNVIPRKSRKNRAMLSSSEEEDFEMGISSIEPRNRGELSRKARQEPQRSNDANRRRSEIDTPQTKTEAPKVTGESAHEGYELLESSIRLVSSKEPMKRKEANMVCDCTYDNDEDELEDACGPGCLNRIMRLECRPELCPCGSRCQNQSFHKGLQVPLEVYETEKKGRGLRSPEKLTGGSFVMEYVGEIIGQEQFKERLVTYGEAGIKHFYFMTLGPDEFIDATKKGSPSRFFNHSCDPNCETQKWVVDGETRIGIYTTRDVEPNEELTFDYQMEREGELNQKCHCESKNCRNWLSSQKKRPTHPARGQSLVDVEPEKVNVAAAFKTLNKHNALAFIRTLLTVINDSDYLEDGMALMLCLDPDKLKPSAVQALLKFRLLKGLRLLLDKSVESLAQVSTLENRRALEGLVDKTVTAIADVATDASVAVAEGAAMDNDTTTEADQQLTQGAGEQESVRAIANGDGMSGAVKAEATADGTDTLGGGNEEIDQDPTDTVSSEDAKAAQTGADAVAIDTDNAGPKPSPIEAVNKQESQDLIVSTEDTDAPEPSVTEAEHTQDPEDQTAPAEVKGEVVDVVTEKAQPPQDFVEEDLYALATSVIRNDVPVVVDAAETAQLRQLQIIILRILSKIEFQFRNPIDDGNMWPVIKSLAKETNKDISDAAGRLITRWSSLRKEIKIPKKTREDKEKEKSDREKSDSRDRTRRASELNNDYSSAYAAAASRFGTHNRLRGSIGEVPLKPTHSTHIHTHTPKDLDLEQIYNEMMDQLPGQAGVQAHAQAHTVGRRGLYESVRGGNGLTYREMGEFSIDPGAHIAMSHTEAGVKPVHGMAEGGSLAYNSRIAPSYGMYSQQYHQSYTHAQGWQQSIAQQHNDIPDPRIQPLAPPEEALPAGWMSTMDPKKQRVYYYHVDTNQTQWTKPTADTVLHTIAPQPSLADEVPASKSPTSPSGSRDSLKRKSEAADKASESAHKRQKTSVEDTKTLEERKRNFRAMVSKSVIKVLGKYNKPGCKFGRITNKDDFKHLARKITHMIVERHTKKNDFSATEAVHEKLEKYIHKSMKKQGSVYTRKTMDSDIEYADTNTAAKRLTHLKERRRRKEAEARRKERRRSDSKYVSDAVSSPKEGLTWNESTARVDGHVDAAAGGGVEYMEEDSDMHISDDSNDLMD*

>SARC_01035T0 | SARC_01035 | Sphaeroforma arctica JP610 hypothetical protein (816 aa)

MRPQILQELWVSSLDRDSLEDIHDWVTFVVQVTPAEDVDRLQVPPALIKIIRSDRGYQKTVQKLKQINQTHGQKDDQSALDMCNRGNDEFRAKRFKESIWCYTQGLQLATFKSATLAKCYGNRSAAYLHSEAYIECLWDIESALRLDQCKTHVKLLKRFLRTADEGKLSIRSDLWTSLSRAPIDDDDVRAAVDAALSWIDSTLPKLVTSSAASAALDNLMCQNEPMNASSNPIRLTNVLADSIHVLTMEDGSGRGVYVQDAVSAGERIIRDEDPLVVTNPHTVAASSVNDMNRIRCVMCSKRCTKRRSVKGGKDQAPSANVHYSVGCLTCALCVYCSKRCQYQTQRRLISPELNAHTAVNPTSPRKQVATTNASDRLEAVADTDSEPVACVLSGLQHASILSTQARQALQMCSYPQQHAQPCGYLSSDQSLGSHTATRTGHLATGNLKAQALCTNATTLTYSFVSECLATAVLLNDAYGVGVEEVWHHMLRVKCNSVVFTDMTSTPSAKKINRNIGSDMRGGSAESRGLVLFATISMVNHSCDPNAIVQLNTDADPGVLASLYVTQPLAQGDQITICYGPQVGRHSLGERKVMLKDQYGFDCHCMACKREEIAISQRYSVFVCKKAVPYVEAYILRKLVPESVRDCLSHPLRLESATALVCDGCHSKYAAAEYENALASITSHMTTATDSIQPTVKEQFEATQRALCQNIAVLHPITLPIGSMYDDLARLAIANGDETAAGRMCALSTGIVGRVFGTSSIEYAHELHKLSSIYYRCGTNRKDLVWIRASAVAIFRAHGAQEYSEQTQELEAMVVR*

>SARC_01700T0 | SARC_01700 | Sphaeroforma arctica JP610 hypothetical protein (476 aa)

MTLPPASGFPVKSALLKGYDLQIREVKGRGRAIFATKRFQPGDIVLESNPYTWVAAAERQQSTCNRCLKDSNSLKRCSTCKSAWFCDRSCQLAAWKHHKRVCFKSETDWLQSETNTAKDEARILDIVLELADSGFGLHKENTIPRPSTADVEAMPHATRPTALDYVLAGVVIDRQRDRCIAKNVTPSHRTVDEVVKMIVRMKCNNFSIWNELLVPVGSGCYPAGALLNHSCEPDCIITYDLDTKVQTFRAIKTIEPGCEVTHSYLDIAEATSQRREYLLHHYGFECACPRCEAADQTLNDSLHAAISSEAAGDWTGCDSQRLDAAKQLIAKVANGELDLEETGREIEMLENALSMHTKYLHRHNLHRLKCLCTLHTSYLSYGLFAKDVAVLNEIVHVYTEVYPTNHPMIGLMQYTLGDVLMAVAQEQRGQQLRTTFEAALVSYRRALQTLTISHGAESELCRNLHGLIQQHSYSP*

>SARC_01720T0 | SARC_01720 | Sphaeroforma arctica JP610 hypothetical protein (472 aa)

MCGNHELDLSRGLERVPVKLTNRSSDELTEKPYIVGLVGKSGTSLAPLAYTQGCACPGSKCDVNTCACVRKWGGTQPGYSVLPDDKDALDSGEWMGLSDVHGDGSKFLLIYECGSACGCNENCGNRLVQHGVNIALEVYMTERKGWGVRALAPIRTGQFVCEYAGEVINTRESKRRQSEMYDPFGLNYLLSVTENIVERVGDRLSVSEAFVGGLDGQEGAERDGTSQNKLVDNSDYMPQSVPLLKRARVSEIEANVCVQEKKIRPDTDTSPALAQTNAPIAHLGITSSLNFVSGTNTVSGTNTGLCDGAPKSDGSDEHSEYSSDTEVKYTQHHPQEVHTSGEKGAQSLRRTASPQHSSYRTNIDATMMGNVGRYINHSCDPNLTKQIARHGLPLPSVALFANRDIQAGEELTFTYGDVIHEQLSKGTYVERETHSRAHSTTGKASDDGTRKVTQCKCGALVCTGFMPFQAD*

>SARC_01991T0 | SARC_01991 | Sphaeroforma arctica JP610 hypothetical protein (1311 aa)

METISYPPEVNMATSKSCAVCGAACSFTCARCKEVSYCGYAHQRADWDTHKVPCHAISAKSDTEIETETEAEHMEAYKGTHHTDNVRKIFETSVSDQYSTVEGVGMGGEELCAGSEEDMGLGDVYSDTDAHTHKLKISETNAQNRHAHTNGVATAQMRGCATTTKACDYEHEESNDHMDASHKKADEELKHAHPYVHAQDSGIYDVDQGIGFEDHVYLDPNVTIEKQLLNKMKEFVQDVEKRNGSTGSGHWNHLVEMFGTSLEHAPHPHQSGEVIEYEYEIGVGDLDGMHLDMNSDLGISMKEVQSSLGLSCVASSHISADKLSQITSKHDSGYAQNLGHDEYVHTYVVGHGMALNGTVADADILIGDEGEIEEIAEVLLKSALRKDTHPQGVSVNDTYANEHAFTSLDRESVADTAGVQPREQGDMELNTGDQSQVSPSSSRTSDSTSATTADAHDPVRNSSGRGARTYASAHRKGRGKALNGSTSKAGDSTQSIRDSVSASGGVGAGMAGRDHRPHTRAQPYTHSKSGAAHNQMTDSLADSRAYEDQDWELEPEPKESSVRPFTSTREPAKSCAVCGKGCSSTCSRCRAVSYCGLQHQRQHWLKHKDACISMTSDGGTNNNNNSSSSNSNSNNNNNISHNDRDISNGYGGNEFETTGRKNLPGKAMSARRPGGYGPVYATDDRTGYGYPKGTTAPKWEFDYDWESVLCVLFESLGYKGPVAISRTSVANKKPVTAVGANIGQGLSLGRGRSVGSSLRGGADGEFNTGEDTGTESSSEPRLTCEAVCVARVPGADDEVYVRSIVPKPKTATVAARDIPAGELCLVEDPLLVVSSKDFVQRHSGYMRLSPEQQKQFMSLFHPTPGTIEVQAYPTLKATETYIDARFAGLDRTDPRTDYDAVDDMIDKMKRVSFIIDMNGLRDAELASADGRHNAAHVYAHASRLKHSCAPNCCRAINSAGRVMVRALRPIPKGMELTVSYLDPTKRLCSGADRQELLRRLGFSCECNRCEAEFDDSFGFYCPNPKCGPPDGSSLGKLDYNHCNGLDVNPDFGKTSNTGTTTLGSKGKSNKKKKPGKKNGPNGGAKNGSASRATATGYGHVFLSHELDRLSPCAQCGTQPDQRYVERCASYQNQLATKLADLDTDCHKFEDDEPFVRTLQFCHTARMSQNRVVFVLHDMCVDWYTNMCEFGKAANYTLAQIRYLTRVCPLDPAVTAYALERLADCMAGCVRPQLYYDQARLRVCANHYPSSYLHANDLYPVRTPFLKAPESEQVNQMYRLAIELLSYAYGSPDRQCVRDVCDKLAWFLANQ*

>SARC_02365T0 | SARC_02365 | Sphaeroforma arctica JP610 hypothetical protein (123 aa)

RWVCDDCGVCASCGKTQPGEGASANMRWKHEYSKGTDTTDPVFLQTLCLACSKLFRSGNFCPICLKVYRNSENLTPMVCCDRCDQWIHIDCDNISEREYKLMSESERAYTCAVCRGDVPSRV*

>SARC_03282T0 | SARC_03282 | Sphaeroforma arctica JP610 hypothetical protein (490 aa)

MAYKGVAVAAATAVAVVGTIAYLDHVRTSDPEYRKKVKARKAAAREAKIAALAALKEKAKAEAEAAKAEAAEAAASSDKDAVGAFFVAQMQAGQEALNKGDLDGCATHFANAVTVSETPIDILVYLKQSIPEELFSLMVKKIDPEVLRDKYFDNFPGEDTGLRVEPTDIKYKQNCMFAVKDFAEGDVFHTEKPFLSALLPDMDPAGYCGLCAIVITDVVPCAQNCGQEFYCSTDCRDVAFGSHHAILCSGAKFSDPTDPMAMLVAHTKSTGRKEVLMVGKALAQVFNPKPVRDCTADIAHLSFDEPLPAPNEMVKKEFALLLPVLTAKVEQAEQILTLDSYTAMLSKIKRNAIPFTTHPNPKGLMVKSGHAVYLAGSFMNHSCDPNVKISFVKKTNQIQYTARKAIKAGDEVCFAYNGFSMKKTEERRAELKKAFAFDCMCGKCVPEVPQPKLSMEHLEKKLAEQKKATENMKNKSTPSPEQKAEDELE*

>SARC_03923T0 | SARC_03923 | Sphaeroforma arctica JP610 hypothetical protein (516 aa)

MAEKSRTDTMEIGRSRTSVECSVVSVSRQSSRGDDRRARLDATSYTVVREDDCERTSPQRYCTPMQDRESYHPRETNKSPTVHKDTYEHGTIERTESVQRRRATYRSHERDHLAERQADTRHDCSARVRESSTCNASGTPVAKPDRELSHRYSGHTPRDTAKTSRNADANSGSRGQERECTSIDSYCVSADKRRVSADRERVSGERDMPTPRTRAMTRSRQTDFHSSERAFERASVAKYTERDLRKSSGTGSMPTSRHRENKEHSYVHLDAPTPCRRGELSGAAGKSRTSIDKRTPESSTLSHNTLLRYFVDEDANTQDGVGVGTRADKKTPSPAVKDGSKHDRPKKVDNKKPAQRRKKKEAVVDPNQSSLLDYGYRRSRRKTQSEVKQKNWLLLEKRILEQDESHLEIYEDEVMGKGLRCKINLIRGDFVCEYSGDLIDPVEADSVDATVAGRKGRLINHNKKLANVHTKAFLVVDTPRLCFFASRDISAGEELQYDYGDRRKEVIRAMPWLAQ*

>SARC_04312T0 | SARC_04312 | Sphaeroforma arctica JP610 hypothetical protein (3325 aa)

MSVARTRAQDAPVCCLCERRSLKDARVFNAAAEGTAGNSGGGGITRGVCGSEVLGNCGTGLGTSTETDRERAQGTAMDVDGAYVAVTGSNMDRDTADPIDPEGAGTNAPESAQKRTLGAEQEAVPMGRDSSDEDVPLVPAASRRHNNTRHPSPHHSHTATPSPPPHHTPQSAPDPMFGDLMMYLDSSSRQKIEGGFDICGKTSALYAHVSCALFSAGVKVASDGSMCDFNQLTLLTAGVGAVVAGEKGPGGDSGCVSGWHSSSGTPGRDGGADGIRYKIPGQAHSHAERGACAEGDISAQPSVGVVRYANTWSRQDQLVEASTCSHCQQQNATVVCAEPGCDVRYHFRCLSGASAVCVDAGPVAVPRERRKVYCHAHTIRLRSAGDQTPCVQLECMVCHTDASGSTFRGSTATGAEGSVGVATVDGDRHVGLPMVQCHSCARRAHRKCVPGLGQTVLKLPRQDSPGARHVTYRERHVHTPRDAERNMLGVVVSGDDVSVDVVSVSDGVMSGTAAVLEKPGLSTPTSISGPVGTKLDITDTDMDMDTIADSTAHAAGYGTRSAEAAKETNGQASATTMATGEVQATAHAQQTDTPMDLRTTVDSEKAQVSVAAENMQVSVDTRATTDSVGLKSSDAHSRSRMDVCAKEGTDGASRADTAVYMQETLWFCDDCQRCAECMSVHDKSDETPTFHCTVCQAAYHIGCLNGVGARLYHVFPDVPFTCNDCVRCSECFFDTPGENKDDQCCEVCTVCLQASQSSSTHMILCTGCAQAFHDFCVADVPQQQEKRAHWKCADCKTCDGCGLEAADTTGENAKGMVTCIGCSLTTHLHCAYPKLKTHPEKPYRCLACALCRRCGTRQATAWHELHSLCDICFKVKQQEKESRKLGYVCPMCDSTYIESTNNMIACDNCEKWVHYVCLKITPEDGMEIEDDMMFYCGGCQQGNKALKKKNDKSRSDGLLTIKNEDFQQKSGPVAKPVKAKKAEAETRKRKLASSTKKAPSATSLSKKIRVQSRKDAPAPAPKIPDRRVTVVKRPNIEDMGEVQRRIQHHKELKRRVHLQKAAEAQALTQARTQTQTQTYTHPQTHTQTRTNEGDGSAHTQQPQQHAHTHTHAHAHAHSQSQPSETAISQVDGADSDTGNSRVMEPITNNMLLNNHHLPPNGYGGPGKGSAKRERPSGAKGGGGYRPHVYSGVDQEGQAQLRAQAMSDHRQPHAQTLADSSRQSYAHVGAETREAAPYRQSRVQGLSDIRPSRTQMVPDDRQPLPRAQEYALAAADQEQRKIETQDVLMTLSELSVSGRSVTAPNVNTYNNRYTGTQPDYSPHAHAHARTRSVDHKGHGSERTRAGEGVQGYGTHDADRYTRERDTPANRYNHAEQMNGPMGYAPRGSENSPYVGGYRMGAAGQNRTIQPRKAHSQNHNQQQQQQQQQQQHLYYVQQAHSQYTNQHENDHEASERRTQAEWPYKPLPHPYALPRRREETGQPHSGGGANMGQMGQLNGHSRMLSSEDVRLRGNGAQIYAPGSHLEGAGELSPGERRASVSSPNLREGVPGSRAYVPLNAEKTVYNQARPTQHRQVAPQAQAQAHAHTQAYTLASVSHAVADPRQAIPNASQGTPHPSASGRLLSAQYSHPDAPAPAGETKTVRLPHLPVRSHKNASTAKGMGARAPGERANQLLAKKYSSQSKQRKGSVRTPVDAREYDPPANTNYARMNSDYTPAPAGGLRDLHAHAHAHTADPHSMYAHTTSEHLRMSAYAPAYGSVGQPSGSGQGVFMDSFRGGGGDFLSAQDRDDLGAGDGLGMPVSWRQPGGTGIQQARNTGTHRSTPQALPRTDPPSPVALGRADREEVNVPNNLHGRGKGRSAPESTESGPGQNKRGTGYGTGAEGPGSGRDMDMDMDALRQQHTEHSPLGTRRKGDTHAELGTLQGTPSHTTLQAPTHERTQATGTSAAHLPTHTDSVAPGVLEVRQPAVAPTYQPSRTSSQPTGTSSQPTRKPSQPTRKTSQPTRKPSQPTRKTTQSTRKPSQPTRKPSQPTRKTTKPVSAEKRVSSPQIAQAHLQPNAPHTVVQPPPDTFFVKECKTTGQQLQQRTQQQPQQQPQQQPQHQHQHQHQRTSHTHTQPLAQALHLHDLPLTHPRPTKGTDAPQDGGGVAGEEPPRGRHGNQEVSELEGSSESEAEVMPWKKFDQRAQQLHQIQKQVSATHTPAHTLAQRDTDAAGDGVAGLAELRVAPNVRDVLFTEDGVGEIQVPKGVQVLESTTTKKAKARSKATPKTTQGQGQAKATEKESKKTVTDKKLPPAVKLTGESGTKAVHSKDGKGSEGVPMVSKKLKTGSTSGGVVKPGKQRKTQPSKKSHGNKLVTKVTDDVAKTPALVPSSAGEPVAMATTTGSNHSSYTQHKPAPTDTKPKHKSLSTDALVKSSKSAVHATARKQRKSVAKPKLTHAVRRGLEDSGGCGVDTEMATSADKLEPRVGDVTDRHESAMSVALSSEQREFATAVKNAMPAMRHTPSASSTTGKSLSCHTSTAPPDTPLLDLIKPLSTLVTTDNRLTHNTETRPVHVLHTAHPLTATCVDSSTRPNREACVQLSRLAMEVCQPYWGEDKLAPALKFWLSGPAISFVLEQRSWTNECDSHRSKWTACDVPVRYHNPFSELRRRTLKVVMPDIARVAFYQFQVVLRVACDVTRVRENESVDEYDENTNFATLLEAIAQKHAKAERGSYVRLAATHPTVVFSTGERQLKINHRCNLCDEKFAAVKCSLNRCNIVFHYPCARAAGCEFHTDGTVLCPQHSLSETVAVDGVDQITDASRDGIGEKPLAWNNPHTWYEEISKTIGLQDVCTSMLTRYEALGTITNRIHETTNAAAKANLPHVYIRDSTLMTVNMGRIVPHPAFHSTQYIYPVGYYAVRMFWSVLTPNKRIPYRCEISTTGAYPVFVITSPEGVRISRRSVLAAVRIVLRLVESARNFDLNQSEALDGAMPGAKGQSNPSPSIDPSTAMRSGATRTSRLSSTKPAARSRTISARNSVSRQPSYSHSVPVKDAVSKRVSFHPRTKHSVYAHWFFGLSATHTVRRIERLPGSWMCDAYRFKTFPTPSGEPARQDEELELAAAKAYDLTRHSVSGCARGDLYTKRRSKARCTTMRVEREDHTDSTPTKNGVTRQRGVLFNSHDAHKCERRRAGDTIETQYKQLRELVRKRTRVGRSGIQGQGLYATEPIEAGEMVIEYAGEAIRLHLTDQRERYYNSRKIGCYMFKMNDEIVVDATLIGNAARFINHSCEPCCKSEIITVCGEDHIIITAIRDIQVGEEFSYDYMFDFDDENRAPCLCGSKKCRGWMN*

>SARC_05171T0 | SARC_05171 | Sphaeroforma arctica JP610 hypothetical protein (573 aa)

MAERKSQALPLKEGFGVFGSESEGEAEEILIDSTAQHVLMRDGFDVFGSESEDEYEGNSVTTPHGKQGLDIGLEAQATTSVTSNKCLQNTPIYLADMDAWHDITSQPLQTGHCSFKRNIATIGGGRAFYAADDIAAGSLIMSERPILDWSVCKPYQRGGERMDAVAVRELLTGTYTPSVAVDDTNSLNEAIEKKLGQLALLCPVSLEACDVAPEVLAEFRASNGELLAGIQKMVRNTYEDQGVPLPNCVRNLIDDEYQSNEEGLNGTDALTRMLLALQFNGFASGLYLHLSIVNHSCNPNCVKTSGLQLPEDNASAWGYSEIRAARDIRKGEELVISYLQPQEQSTPRRQKQLRHAFHFECVCDWCLPCACPDDLDYHTLIHTQGDVSDSDMLQLEDSIEHLESEYREATSPAAKLDIAEQLLEIESSATYEHLPEYHIVRVRFENLRSEVLQQVLGSPGDTSPPESLMWSLVNKLTIQRLYLAEHDQRLADTLLALSSSANQLLVSHPEVATTAFRSLNIPDTQGMRSYIRAIDKEWGVINKANKANQRCDPIADKKVLAAQHVDRVGFDE*

>SARC_05279T0 | SARC_05279 | Sphaeroforma arctica JP610 hypothetical protein (347 aa)

MEQPGSIAESLNTTTTSDNTTTKSVNTTTKASGPTLQTKGKSKNTITGEVVWNTITKRSPAKDEPYPTEDGQNQKSEQREREGQSGPLAEFQRTVQQQLCMQHCAITPHDVALFSMACPGPGVVSLYEKQLAGEDATRPKMREHASGSARTEGYYTYSRKEKQANMDLIHIPKMREAARRANAAPGSVKGSQRAHRAKSRNHTTFGEEASTQQINDLGKRHKRLKFAKSLIHSQGLYALENIPANDFVIEYLGEVVRAQVAETREKEYERNGMGSSYLFRIDDQNVIDATTQGNMSRFINHRCDPNCVAKIIKVTGQQRIAIYAKDNIAVGEEITYNYNFPEEVYV*

>SARC_05902T0 | SARC_05902 | Sphaeroforma arctica JP610 hypothetical protein (194 aa)

MAVDTLSAVGVATEPSDHLWAVRMLMAINCNQGNGCVGTCALVLVQTSVHRLTTTWNSMRKGVFNLHFGMQRECDGQVFATAVYLVPSLFNHNCTPNVCYRSEGRQLILSTNQAVEKGAELQHSYLQRSNLRTYEVRSEALENSYFFACACDTCANELTLPTEHEGQGHSTPHSDSSGDEAGCYWNSDLSDSD*

>SARC_05940T0 | SARC_05940 | Sphaeroforma arctica JP610 hypothetical protein (474 aa)

MADATVVFEQVRSTIRDHGDKIQEIMYGVWTGAEPETDSPSNETAAIDNTEIASSENTENAATSTTVKDENKPGYILRNGTDTSVTDVIQCVCQLNVNEGCMLQCSGCLVWLHDTCVQLTPEQAADDSFDFVCAECLQVSPSEFLVVPNEYTDDDEERHNTYYRYLVRDGTHYRQGLCVYVPVNTHKRSSSRTAHQNIIRIAQIWTTPDGQAYVSGSRHYRPWETRHKINHRFGKQEVVKSNTTIVAKVDDLLGCCAVMSTKDYIEGKVYDIAERDTYYVEWRYDPDYAQWKKIRKHDFAEHHTDLCNYVYRATPADTTRYILNGSEFVEPPASTKSKGKSSPNRGNGDKANDALRPKKGPAKVSNANVNTHTSKRGASREGRSGSSVPETGTAPDTTTNEGVMANKIVGCTTAVGYTTAERGAAHMSHDDKKEAMESILDSLALEVDEDIRKVSVDVSFLLVGSRRRCGNRS*

>SARC_05941T0 | SARC_05941 | Sphaeroforma arctica JP610 hypothetical protein (480 aa)

MQANPEAHRHLDMYADIYADVLKPSLKERQAIVDDAICSCTVPSVPADGAGLTAELTGCGEDCVNRSTFTECSPMTCPVASICTNNRFQTNMVPCKLDVFDTNSKGYGVRCKSDIKRGALIAEYVGEIVSETECRRRMTERYKHMNNFYFLHHTGNNVIDGCQYGSAARFINHSCSPNAHIEKWTVKGLVRVGIFASTAIARGTEICYDYKYRVMGQGNVVCRCGSDNCSGFLGERPKKIVSMATGSHKKGRRKGNAAKSAKLAIAGEEPVLEVSLAQQRRNQARDFTYARKNRLFLVRNIPSHKLVPLDDLQPGRFGTANPDSRTNGLILGTTRNSAQNSTGRNSAMGRHGDVGRHSGTISSDSGVHLAVNTDYKQLVRIMRMIYTTVLKTVRGQCRLFLRLPPRSRYPDYYQLIDTPICLADIEQKINSGLYPTVEELQVRPISRTAQSQKRVHTATLHVTYLLYPTRWYCVHHVVV*

>SARC_05961T0 | SARC_05961 | Sphaeroforma arctica JP610 hypothetical protein (1114 aa)

MYVYINTLHLGVDIYNYTIIFLDTMLDARETVSKGVVRGQHTDTIPLCDGCRGREATLSCPCEQRIYCGDECHALDWKNHWKSCSTVDDEQKKAVAATQTKASEARLAEQKCDHCRVKVAILQCACGQRVFCGENCKDLDFKEHRWVCSAVPEAESSAGYRAAVERHEARPQFCDFCKQKPAIQACSCMQRNYCGDECVTLDWKLHRWLCPLESGGKRSATAGILVERQEARSLPRHCSRCLSKKATVSCPCTQRSFCDDQCFALDWKNHRWVCPTVPEKNKWSTLRTVVEEKERKVAPRPPLNNGRLPTSLDDDHRAPSTQASGTPDIPIVEKRSLEVKVSSDRDHSKENQREKESQPNKKLRVDRESSGEGPSRRGTRDMDRNSKGRQRESRESSGVRDSGRHKEDGRGRDRGRSGSVYRQSEKERESRKHVDRLRDERRSNERAREERRSRDRAREERRSRDQKVLRSRDQREHRSKDGGWSKDVHRDKNTDANMAKEVHSGENGSKNIESRVTGKTREGRGSRDEGIPRERSKFGHHNKNEQVNVTQHKEKVSNLNKKVSGIAEGPEDGRSHKDRTVDSDVGRDNPYHRSTKPTEELVYPKKPIRDDKDATEHQTIPEPVKSAGVPPEKKNLLRQWTEESMPIPKKGETFKIRVPKKGKARIAHVLELSAVQDVAEHSPLTKIHTVRPNQNNLSVHDDASAPKSGGGTASNAMKSLKKGSAHSLLRKQTSGTTDVSGSGDARTIFSRSSNESEKPRVNKTKIMDEQRRGLKKLRKEVKIESRRKRKLSRGMKEVSKADTVSQKNVKKDLKMAKMDYIDIRPKYVTSDSSDVSSGSEASAKYTDGNSSASELESEDGHGFATDPTKGKQPVCTICGDPDNSQGEGGMDYLFVCATPSCNNAGHGSCYGFTSVLVDNLFQSDRPWYCNYCKKCTKCGSTDEVNMYLCEYCDSGTHLACCDPPRKKRPSENEDWFCKLCTLYGNGAGIGLQPIPGEYRPKLFTSAKTGAVSSKPTSSGESSHEVATTNKGSATNKVAVQKLSAQTSASEGKTTSSAFPKSVHQNAHSDTGSSIAKAASIANNGGMRAQLGLTNSPFAIVKPNATVPLADPRL*

>SARC_06292T0 | SARC_06292 | Sphaeroforma arctica JP610 hypothetical protein (561 aa)

MHRQVCRSHSICKALPMTDRGLLFLFIPNRNAINRCYDERCENRMTMTECTKADCNIDTCNNMRLSKRQYANVNVFKTASGKAWGLGSKSELKAGQLVIEYVGEVIDTQTVQQRLAQAKETGCDDFYMLGMDNQLYIDAQFKGNLARFMNHSCDPNCETQKWRVNGEIRIGIFAMKDIRKDEELTFNYQFDTFGNEASKVCRCESANCSGFLGMKPKKKNSGAMSTQESGQKHRVKKSLKKRKVRVEKITEDECYHCGDDGSLTLCDMNFCPKAYHVGCLGLEKAPHGQWHCPWHQCSQCSKKSTRFCVGCPTSTCDACSVQTELMGHVQRKLTAGAKSTRTFGLCSECTQEAGVSPDSATEHVATIIDKVLLNTQLHRSSGSRHRKTKSTQAQAHTLTTHTVEPIKRQTKRSVQAMEPLVLSDMEDDVSSEQSGSGLKQRDESSAPPRLSTTTPSEDTQKSSSGEEPAQTVAGPNMAQPRAEMSVDKDERSDRDSQVLASGSSIESNGDLAMSDDSGNASVSSHTESAVSAKSNSVMASEKAGVVSIVEDSLGESSACA*

>SARC_06308T0 | SARC_06308 | Sphaeroforma arctica JP610 hypothetical protein (1396 aa)

MVVGASVGKKDTVNKENVEIVNGGDSGTIPTVAAESEPMNPVDVSIESTNNIAKEKGGSPVCSICVTGEESEWDTKIIPVIAVAKCIATTHTIEIDSKPKERVKTRADFIEIKPNRVPVVGVEHSSTTTCANTYSVERPAALVTMGSVADGASPDAAAVAQVDTNPTAASDGGGQVVNEEKGIMQQDKKIPTTSDDGDENVVVDGSGPQNNHEVSVDSDGNDMVVDEESNDSAGVNRDGKHSTGAGSPTSEVYDSTGNVDSTATGGAVEKRKSTDQAHAVVAELTITDISSDDDSTSPTAKAGLSASERAACTTSSNAAKLEDSYLKDQVTSESAKSSYESAAAGSETVEDACVAEVANTGSPRSVGLSENPATDKIDAEQEVDMIGVLADSKGNMASAVESSKEARHEGASDTDDAVDSHIQLGEVPTTIAGMNRNLTHAVLANVSKNCAAVAEVSTDIPDNISKKRSHGGVSKTSTGNALSPGDKQEGKGKKPYINECASTRMYTKPILAAQESSALSSAVKVAPDVIDLEMVDAGSGMDALKAPSPIDTLDHQKGLAKVKAVKTLGDYWRGNGDVHKPVMIGTGRKSVKTAHSWDNNSSGKHNVRGTKGQQNGVIDLSANDSLGRSDKRNPIDVESDPPGKGTKGNTNITIDGSQNTPGSGKPQIKKRGRGRPRKETYRRDPKEESIEDEEAEVIPVCSECRTSTTCSDVNPNPLDKLLVCTLSGCPNAGHARCLGFSEILARNLYYSTDSWTCMVCKRCVVCEEGDGDNEMLLCDLCDSGTHASCLDPPLKEMPDPNKEWHCVACREQAAGKMKSKSDARRGGKSKKRRKISDIDYSASNTSCDETDDYLAGPSTKAARTIFSRSSSSTAPDHAGSRPTTKHKKDTSLLVKLGGTLINKQAPTSSGANLYRLTGGSDPLGMYRGETKAASDTGKGMCPIAGCDGSGHSTGLYATHKTPSGCPILNPPDTDGQQKPPMKRRLSANEPSHQSNLHRRQGNNTHSLQMVRSSSAIDLSGPERNSTPAIPHPNNASAHKASYWEPFIYPSKLDTRTLITQETVIGDDSAIHPTEADIVNFESARIQAVDLLRSEYSGISYTKKLEAIIFGAYTLKTWYSAPYPEEYARLPRIFLCEFCLGYMKSAVILDRHRQTCDRFCPPGDEIYRKGKMSIFEVDGNRNKIYCQNLCLLSKLFLDHKTLYYDVESFMFYVLTAYDKFGYHLVGYFSKEKDSQMGYNLSCIMTLPHCQRKGYGRMLIDFSYLLSRKEKKLGSPERPLSKLGEISYKAYWSSVMLSYLYDHREASALSVKDLCVGTGFTEHDIVNTLLDLKLLRYQSGKHVLIRDVELLTKIAEKQERFDLENECIVSPQNLSDRWCPYKDSARRYSSSPRKIYS*

>SARC_06384T0 | SARC_06384 | Sphaeroforma arctica JP610 hypothetical protein (204 aa)

ALARDGDVKMAAMSVREMERFLELATATGGHENEVDALYCASMWAFEGIGRTDRRAYAHEGKRLFELAQRAERDSGEENTSEVKRICEVYMSQTNTPIPDRLHTPGHALNTTGPPLPSAIDLGVGVGAGVGAGAGVGVDGGGLSGTGRECHYCQTVTTDLKNCSKCKTPAYCGVECQRADWPRHRKICWPNQTHNITMDRSGA*

>SARC_06586T0 | SARC_06586 | Sphaeroforma arctica JP610 hypothetical protein (202 aa)

MLRILSSRTPAFSSSLRQGMLVRSLCASAAQQKPTGESSIQMRDSIELVYEPKVDGQAAHAAEDLNATQVIGRIDLTQNIVDKADRFSIQISETTHIKGSALQECVYLNHSCAPTCKVHFHNDTLDFVTLQPIKKGEALTFNYVTSEWDMATPFDCTCGAPGCVGHVAGFKHLKPKQQAALYRDGLLSPFVANKYDTQQQK*

>SARC_07401T0 | SARC_07401 | Sphaeroforma arctica JP610 hypothetical protein (106 aa)

MPTSVTMPATWTADETAWLQGTCAQRQTITAADRLQKEFSDTYTPLVRDNETLFNGKVRTFAAYQLAAGLVSSRAFRVDDESEDSEMLPLADMYVVGKCGTNQYL*

>SARC_07640T0 | SARC_07640 | Sphaeroforma arctica JP610 hypothetical protein (854 aa)

MSLLHRCARKGDDGAIADFLRIQGSVCVNVKDRHGLTPLHYALLHDHNSTAMLLVQSGGADVTVRDVCGRAPALMAFSIGLAGELCSDLSTEQAHTLIDDKGRGIVHYAASACDPVALKLALRLLDKPSDERKAIAIELPRDSQGQTPLHYVLGHPGANPRSTATDTVTKAYDNMTTPCDNARHGVTPPCDTTTNAYDNTSYLSDNATEAQAKVSQAYDIVPSACGTCLQNVSLLVDHCGTVEALFVPDYAGRTPVHIGGWRSKVHAMKQLKTLFVGEEKYRQLLNTPDFSGCTLLHMAYFRRDLDLATYLQIDMKLPFTLDGWRRSPADCRDTEAIDQNLEKWAELERLAREWTDANVESDSESKSAAPVPFQAPPFLTCNHGYSFLMNPQGDRQSRGIVPLSAKNKQLSTNSHSLELESSVAHAGPYGDIVNGLPIECSQQIIADDVQAFYLYRNRAARMGGCPDIISNLSHTLCPALTRLVQPLQTSPALQVRRIEDPTNPAYRPSRRARSRRSTCDGGTVRGVSKSEHAPMAELGGGFGLFAEAFVPKDTLIGEYCGEVVRLDMIESELAEDSDGSEGSEVRTAENTNENVPKDHSIGVAGKCIPSVDSVGYINKVIPKDYSIEIHVCPKSPTDDSYCMCNDNEELVVDGSNYRNALGFANDYRGTLNKAKDTSTATHTGSGTTADPDDVACNRASHRSICTNGDAEEHTIVNNSHSDLPRDRQCLAVSCVGCSRSQQKCKPESGAAGCGARPRCDDGLSQNTHASETVGIEPRLNSAASMAHETASLTDQRSKRKASDVLSKDRLQNTHFVQVMFRGWPRIVGVTIEDISAGDEILIDYGELFFQQHD*

>SARC_08990T0 | SARC_08990 | Sphaeroforma arctica JP610 hypothetical protein (434 aa)

MVALLKDANMDVDMDTHPDWETDLFQWGRCLGCRPLDVKVDTAPYGGRCLLCTTAKDVGDLIVSIPRQMVITAQSVTEKDERIGKLVQNLPDEIASGKTTLTLMLFLIAEKNSGADSLYQEYINTIPVTYTTPFYASDEHVQSMLAGTPLLFVYQSVREELVSNFKALCETITKFSDLSPITWEEFLWAYHVVESRSFTTMMDGQETNMLVPLVDLADHHPTEGRRVTKMFNSETQCFEVKAASKFAAGEQLYLQYGDVQNWQLVIYYGFALHDNVFDSFDINLELPSDDTPADSMRRSLLLTLGENSGMFNTENKLSIGADGAFVVTPTLLPTLRLLTADGVLLEKMTVQNLETYIKDASASKERELRMFESLSAMFKATLEAYPTPLDDDLARLSEDGVSSFETACLVYRIGQKRLLTAGLGWVDQQRRSL*

>SARC_09158T0 | SARC_09158 | Sphaeroforma arctica JP610 hypothetical protein (685 aa)

MLLKSRYIWCDNSCCTVLRFAVVNYLSQASDWKVHCLVCPTVPEESRLAAAREAKEANEASRLVRNGGTPISHTKHENMSVSSRLSEKTDIVQYYTANNQAATWNGGDGDGSQHTVHERDVSTDNRSKARRTRQRSDALFDVKDQKRRYTKYGTWQHGSARGVRDAQEQRRGGLQRRARGPGKMPDLQTGMKKGRSHGERKYVGHRKPSMKKDASLRTDGGSSRGTADSRGKRARAAEYQYKPNGKRVGGESRAKPDRSTAPRSKELDRWKGPVEGAQAMKRKSIGQPNQTGAASSETNRQARGSKKRARMDEPMEAAPNHELLAIDAFVAAHRKRKGAEGAASDRAGDGVTQTGGMGASSSSELEPGSEPEAEFTSGGGSEVSESEANAELEMQTLPVKDATPKCTLCGDPDNSHVEGGMEYLFVCATPSCKNAGHGACYDFSGQLVDNLYRSTAPWYCNFCKRCTKCGSMEERGMLLCECCDTGTHMACCNPPLTTPPSEFEDWICKLCREHGPGAGIGLYDNEGFRSTPTQPQPQPQPQSSHQVQAATGVSGWTGKSGRMGASLLKNRSTGDVRVSGVAVANRRVQRRTVPSGLRDMAAKLYTPQNISMAGNPHSQADTVTLSTPTAHASARGSMGETTTPIDAGRSKVVGINGQPIMPKGLTNSPFAILRPVVKQSSTTA*

>SARC_09709T0 | SARC_09709 | Sphaeroforma arctica JP610 hypothetical protein (2308 aa)

GDEPAAAGEEEDQDEGGVTACVCGFEHDDEHMIACDRCNNWQHTVCMGLKPSTVPSIHYCVNCEPRPVDVGRAKAIQSKKAEELKLVEESKKRKREKKIQERIQKDQRREAKRLQKEREKAEAGDNGSDFEDGNIDSDDLQGGVAGNDAMSERTRLLYESEYTQTRGCVLMPKELEDDLSDWTADGRLKGPQGHGGDYMSAGESDDISQSDSTSSEGNGRRIEGDRGPRNRVASNVMSQGGGVVKRLSACVRRYVRKTGRYTDDRAKDRGKRDGDISSQKGGTARTPDESQGQAGSGSGGSMALAASVHNQPVMHVHTIPKTANVCERPVEKSFQRGLFLSTQCSRVWSAGEDIHEYSGQLCASSAFVREWEKEEAFDITKRMCPHVVFLKGGVCVNARYYANLTRCVRRSCRANAEVVVCRVEDDAHKAVVGDLIRPESVTERHRISITLKDTRASGELTVVHVGARSGAQSLGVSADRAKQGTKERDGHVVGDTRKYGGGVSNKVEAQTADRDSGGNGTDGTHTGSESGRDTGLGNEHGSTVANNGSGVLAVSDKDGAGPEPEAGIEGRDRWNAPRVEVIPSTRTNTDTDMSTCTDADNPVNATMNTLTNAHAVLRTNADRTANSPVNVACKEEGTMEPGLVQSGLRVIVRAVATIKPGDEITIPFDYNYDVPAYVCTCACGAEDCVVLDSHRLKDLRRRVSLNTSSHAHRINSNNSYNNPHSGDGLTPQHSDSGVNSTYTSMPYAYINSPSAHIQAHVRMHRASDPYAHARHRDSMYSHDAALMRHDSAHTHAHAHEHSRNDDDMHASSGTPRGSVADILGPPRGRRLNMEEKKLLAILQTIKRMERQQQLQKINRDKREKEVRHLTTSAPGSVVTTPKVQSPSASRADLSSAPAPSVVVPKITRITVGKKGWMREIRKSSGNTSSSPVKNRSSTNAENVSISTAPMDTDEVSDTHRKSSATTAATTSTLANTNTSMNAGTHPRATADTTTTKPGSTKGAQIGAAAETTISANKRVGRKGSPGLVVSFRETLHRGPANREVSNKESAKDILLSDTNKIDTGKEREIVGSSTWLSVSSIAVDEGLSQEKLVAGTTAPHDSTICNDEQEAGDSRPGSGQNSTDKERGQIEPVKVQTSVTADDKTRAGNEEDGSSRPSSRREERHVVTVESGGNDEKMLAVGHGGQIGKSEAYNSAKDPSKELHIPVVAVLADTVKDSPKMKRRRDDRENSHSHSRSPTTRTAETGVSTNHSTITSHSTSSMHKKLKSSSGSRESNSAHRDTGSSGKLDGDQKKTLDEGRSSEPSKENGRGRSSSKSPAREGSGSRDKQGAINRNSGSEEARPGTGPSCTNKTSNANKTSTYVITTDMGGQTNISTHSLATPVSDNRTPDQISLTLAQGTSVGIGDLERQSKIPDRLTESRRDMHALGSSVSPEGTKRKAAPADLDQTASDTNKRSRSRYGTERQSSTEMSADANAGDAATTSDGSKNKSANSSNFENTGFQSDRRKGKNVQLSESPEKSAKQTSVDEKTDIGRDKDETLRKILCESGDASTKGAPGSGADSTSTDAVQMESVHKDLGSDTALGRATTQTAENNNASIQVGTKSSERTVQAQGEVAVANGQTSESRAGDSKRVSAAENPGRADTDRSSAAESKPATTGDLSDMVGQVLEPATTDGKTIQNTNTTDTSTKISTSKSTVADKKITPNSVTNVAPDAKTESESETEMHAHANASEICGTSALAGRSADAAVNKHTYTKPSDGGDEVPRKPTVISSNTDRSGGGTGAGVRLGNSSGLEIKRSLECSSGKGVGKPRRSISAPSRGSARQNSMYRSGSVSRESASAYSPKAARTDSVSKAHPVIKSKMISTTTIGLGSSSQQTKPSDRMSAVVGTEKRSSKLDTEQSDTIGGARAGGDKPRLSERSSSGAYSASAKNKVLRTSSGMVYKSFTDRERERSKLARSDSLPKARSGSASVPSRSSVASRLSSRTNSQSRRMLVGKTLDAPGSDLRAILLARKHTQEYSKPKLTHSLSSTSTGTPERSKMSSRYIGRDSTGRDSSGRDSGGASTNGSGRANTSRSIRRLSPSTNASTYTGTASTVGPHIASKADPTPGTAEGTYASTPVQPGAYGATTSQATTKSSVLPGKGAMTDSSSRIMRNSSLDRATSLRGQGKSSRRSRANSAMGQPGGSVAMATTVSTDLDSNLADVNSSSKTTSNTSQIVQASTHTVPSPTLLTKVANTSTRVTEEKVPASDASSEALARDLPTVVESKNTPAPSAPPASHPQVKKKLTLSELSARNRVLRESQAGKDKQ*

>SARC_10284T0 | SARC_10284 | Sphaeroforma arctica JP610 hypothetical protein (1325 aa)

MRPISPGDELLIFYGSHFFGLKNEFCQCATCEINTANAFDQPKQDRVQLPDHSQENTQSQPSATLHNISVNPPTNTHAQIQTQTVIDPQTRKQPSSQSSTPYDRSARESTASSTGGSVCSSESHIRHVELQARTSSNGKLAREDQCREEGFDESGVQVHITGRPEIQKPTQQSLRPRRSIRIDYRESSSTMTLSRSNSSNGSDTCVYDGVSVASHKRTDTLATSKSCRQPALARTRSMKGRASADNSDGGAEGESDASHASWIESDPDNIGTRSANTATGYAPTAKNPDDQRHNSRHGLRASANRSNSVGASSRIERLSASDRSSSADNRTDSNTNLSDEQRSSAHSKSRAAGRVQRSQVKATDQYERIVQQLMDANSLIYQPERPDSEIDTSTVPGAIKRVLSTQRKLATPPLPLQRIHWVNKWMKAQPTKHVAVYVQPKCRCRSRRPKRSPEYVDEATTNFSSENNSSHAAPSNTHPARSKSIISLKGARTRYNSPSASTSAESSRTVLGSSPAGPSSGGCHCVASPERLRKSIMLKHHSNLSASHTPQQHGGNAEGGTPLMITCHATDTHQSVHKRRQLSSTWRFALLCPPPATTEQDARTYTATRYRNRTLSELEFTAFDVTAQALLPQPKLVKVMYVDDDGLAAEIRAADSGEYDISLATSRYRLETDHVEIDRIVRFELDAWPHVEYVKEKPVQLGSRTSSRTRKMTIARDMSIDVGRASRTDRNDRGRTESSHVTDMTTTEGTSDNVFRHNVTQQTPGTHSQQPTHDVRQKASNDTINTNEETGMDMDAVVTQGRKRKHTETNAVRAGAEGQFTSGRGALQGKSKGKKGYSQAQVVVSCSESASESDGEPTLPPAALSLDDSRWTRIQNRGQTQAPQHTGYGAKPRTREQTRTHGERQGVTYTEQQSHTQLQLAPHTHAQLFSYKKPSDYHSAHGGEGGRYIASVVISSSSEGSECDEGNPSSIAVNANNSPGKSRNAHDKNIDLKHMKNRLETYIDTDDKVSEAKDCSATESLFERCQAGNESVQCMHVIDLTTSSPSSSPPPAIQLTDARRKSGKGFYAACKIRQAAETLSEPHACDSDTTPPIGEAGKNAAASATGRANVLAEVRIQDVHGGVVDFNAITRTTKIEADTETETATATETETEDNKVPHISTKDGEYFVDVDARVIQSVMDGAKTGDARVPTIIGKGYPEGVSVRASMGTHDSQPCVSAPTSKMKGTCIPTSTQDSLILPKGIATKGTEIHNRDKTMELCTALSGLTPSSETFASDEDLSIKQINCMHYPGKRMEEVQSEQMEKEDSKGSENRLSFTLMRGARAC*

>SARC_10542T0 | SARC_10542 | Sphaeroforma arctica JP610 hypothetical protein (341 aa)

MCIRFNHRSGGEHVHIEDDITGDGSNASGAANAEERLDIRTVQDVQKGEEVFNTFGDLSAADLLCKYGFVDLDAKGRARGEAETCRISLSLIVQVAKSHSKVGHKEIDKRVQELIRQRGDVFLGDDVMVVDIDEVASAEQTIQSGENTTEGTDKTIKLDQNRTDTTGKEGQNGTDKAFEPDQTDPDVANQDSKISAGKAGANESEIHGEVLYSVVGDGSFNYHLMLMLYVLCVKDKNLVRRWLSDANGLHMVEDMNAITMEEILELEGVSETLQNIVVARLKEYPDGKLEEEQAQIAGLVKNLASLSDSDRNKLYALVVRVEEKDALRKAYHSIFDTSET*

>SARC_10794T0 | SARC_10794 | Sphaeroforma arctica JP610 hypothetical protein (724 aa)

MSTIVRTQSGCELLLRCNGTAGRSSVVYSHSNPLILVDGRDLCSRNEDVLSVRSLKIPGRSSPSIAQMECANGVICCEKVHPVHEIIVGDSKISTKRKGLKVCSDSLCFSVPDVLHRCDVCELDRVVDRALVYGNLIDNNEKKQDLQLQGQQPEVEADSQNLQALEPKDEVKVTHVRCSNMQCTEISDTLILCKGCEREYYCGARCAALDRDRHAPLCGFKVAYESSMGRVLIALRDFKMGDVVLREPLLMWESHKEDEAPMNFLKAYLSADRETRRKIDDLYTPNSDTDNVNVLYAKNTAASLAKLQAQMIVPDHKSVCTCDCSKQGSKKSNSDMRYVEDCTGEGECVCECKCTPKDRIDREFKHISQNSLVRLMLKRNFNAWSMDPSGTKEGLGYYASMLNHSCDPNVKGGPVSGTLEYTATRAIRKGEELTVSYFDNSLIFQSLQERREALLASHAFQCCCNRCIGPDLVRPLHCPVNGCDSVCLRTSSIQYVDDDSSGGPDPGVWICQDPGCSFKGSDNEGDMPVQISAEKRLLRLANVTESREDFLYKLRKMEAVLPPAHHAYLKLFHCYGLALEDIRAPLADKQTTTFALLELILWRERFMAIHTAARCSQEHGMKWSIDSWCKNKWRLVKWSGREHASESTHDYLVDIWRTGQGRANETAIVTLQPNLLTKDPVIMSVNRLLLLNMTKLAIILLLRYSAVASAEEFTEGVVPDGDF*

>SARC_10855T0 | SARC_10855 | Sphaeroforma arctica JP610 hypothetical protein (434 aa)

MYSGEGEPHLTEKIVGCPLECGNVYCSKKCRDADQNEHGHSLLCVGQIDSLDHPLYQFKVHSLTTQEEFLLAAKALAVMMGVLWAADEDEQGGTLAQSLLKPYAHFQSGVWWDLAGGQEMAGVLQAQATEAFQLLQAAWSDLGHLPENTHGIMSMDTFNRLLSIFHLNNVGVRRVSPISRYMKMPPFYLYRNKSLVYYEQIASQLSDAATQDEIFDTLMSHVDPILMGDGCDDSDCDDSDCDDRESESESESEGEASSESGMESGTTSKPEIPDNSGTDSAIEPSACNDANDGDVEEEEEEQQQQQQQQQGEEGEEEDDDDEEEEEVTAAMTLGDWGDADIEAGESLFPMFDGCGLFPLISAINHSCEPNCKVTYGTTGRGKLALELRSLCSINSDTEITISYTATDLPAEERQEILGKYGFRCNCTKCQRGD*

>SARC_11016T0 | SARC_11016 | Sphaeroforma arctica JP610 hypothetical protein (1611 aa)

GITSPANTATTPTLGTNTETKVEMAALQIVNGNMNSNQDVKSDSNSQVIVSANANTGNDSASSEMTTDVVADAPIQSVFKMEGMLADTTLASTTAGKCTDTTPSDMAFTADVDSLDSTSSKMVKMDKDMTANVSAKGGMGTDSAALAVCKQEDIVRKVEGMDKDKAANVGTSADTSTDAAVSEIAEAAAVDLSRGIKIEEDVSIENGTDMTCALAGGAGFSPAVGTISPLCTPKLVKGTPNTTPIDTSAPTYISPHPHAHTPTFTHTASEAVDMLTQPLPSPEPPYVHTLLPPALSAVVGDSTRTILESVVVGDSVVPSLESQLPDPSLAHTLEPPVQIQTSSSYILHDKQKMLQTYGNETQLEGKALLEGDKVTSYNIIEYGTASAARSETLQNECIEVELELDVSEADSKNHGNEFETSTGACKAAQGESGSSQPESVEPLGNTRMPSAEELAALERSVVSQDGGTAAQAKTEAPEVDVQVSREEYLSSSEGREASLEASRASQTEMTALIAHPELSASPEDSKSLQGGSNVHSPQSDPTYARMRMDTTTTLPRGSATEPRKGRKRKRESVSMLKKLQTGLATHASKTEECKRSLQDLKAQLGEADSSKAVRGGSKTLHGGSKAPQVGSSALHGGSQPASPNRDGAVEELKKQIAKEETALKVNEHWVEYLTQEIAKLVADGGGINGSCKSKLVDSQALPIDGTRLQGQRRASPVRSKALQATCESHGILRAEISATADCKDNANKLRAVRWKRARNETLAEVSDDRGQLTSSASLLEVEDCRGESARNSQSTEAPAKTGQLTKSATRTESTESSVQLNAALTEAPDISEQLSRGVGGIHPKRSAVADQERGASGNNTLGRMRRKPSQIDCESKPGSTIVDGIDISAPRPKRQRASTTSPVVKQQKPPVNPTIQVTSKKLRTKLRLKDKTPKKVPGVETISKATTQLNRDGKRVCLGKEGISPTTTGRVLKAGSDVPKRKPGRPKNKTKAANDVKVELKDGLKIGTAAMGGVPAALPDGKAVEDNGWVSDVGVAEHLVTRSAGPSSGLVQSGVMTGVKPSVKTRIRTAGKMSPVSVSETAEKLTGDSDRMGKVEVKVENVDRSASPNTVYHANEVDAMEADPVVGMDVKKAVEKGVKNGIRKSLKRNAFKRGVNQDGKENGQTVVKKEVNRSVGSGIATSVGDTVVDGVRGLNVVVKAETASSKKPEPVPWYMNMPPEQELNPTVWDDEIGLFIDNILLRFGNNQDDRYCYDMAYVLKQIIASCDISSDARVIRFCFAVLKSIDTYGIEAFKIHPKGAGIVCNTEEGLRGNEFIVEFYGEVYSPWRWFEKQDIVKKMLRKGSLPEFYNMMLERHRDDAKGFDVLFVDPTNRGTYGSRLSHSCDPNCATHVMAVNGRFVLAVYTVRDIKYGEELCFDYNSVTESIDEYRKAVCLCGTSKCRQSFLYFANSATFQCILAKYHSTCDRTGAILMACSSPELTEDDRQRLSSHDFNASVLTGCPDWLQKWASLILKFIEYERMMMPHVLTADFKDDFGHPYTLESAGVEAAGVHGNREQNLAITLDKIKYILRQQPAEFADTMPLRPTSPQEISDYLWRGKESIVKRTYALCS

>SARC_13768T0 | SARC_13768 | Sphaeroforma arctica JP610 hypothetical protein (286 aa)

MSVLEESRLQYSAVEVRMTDGTGWGLFTSHAVRKGDMLLQELPLVVLDIKYDATPATQQKFQKTIQWASRWTAQGGPMNEYPKEFRKLMDDCVCECVQEIFNSQSGDVQRKWMALHDAKTPNDTVITASKLFYLNGLNSEKGRLLNGLECIILSTAPMSNGRWAARLTYDGKDLSVKKDSLKKKIPGNVASTNSFSVGGYDGVGQKLGLYEVLSRSNHSCEPGTSRSIDANGLAVLRAERAFSQGEQVYTRYIAGSLPYEARQSELRDKYGFDCGCQKCEAERRV*

>SARC_14258T0 | SARC_14258 | Sphaeroforma arctica JP610 hypothetical protein (538 aa)

MTKREEIKCKKEDNCNDLVVTKRAQRCLKYEALRKERLRAEALMHGVCIMTKREEIKCMKEEHCNELVVTKRVQRCLKYEALRKERLRAEALRKEKLRAEALRHDVYIMTKREEIKCKKEEICNELVVTKRAQRCLKYEALRKEKLSAEALRKEKLREKLRADGSKRVLRPRLRSNSSIKRTNGSIEHTNGSIEHTNGRIIRTICIETGCSAPRDNGARCTECLRNYNAKRTRLRRESDLKEHNSLFTRCQWALCEKNIYNAPISIYCKAHTRIRTQNNTSDRRRRAVNRAKTWEKLFRKPPKGADSTRTSGRYVKDDSRALQQCGVVCQDHSVCGNWTRNIIPIHCLHKANQTRYVPTSASAWPKYVRSWKHIKDDIYEGVCLHPWCKEVQRFPYCPTHMAITLAVEVKQSTIPSLRYGLFVLAAFVADEFIVELAGERLPTTGDVCEGDYLMSVEYNGERIIVNSDTSMHGVGRYANTGVNAFQNNARFQAYVVDGVLRVYIMATRLIKKGEEIFVAYGPDYDLRNSRTDTSRFR*

>SARC_14612T0 | SARC_14612 | Sphaeroforma arctica JP610 hypothetical protein (142 aa)

MAHSFRSGLSKLQQESVRVVALGVAGGKIGVREAVAIVNNMKYVAMQRESLRNAEKKVLFDKHLELFINMFSPETGVTLATALRYKTERAEAKVISTRHWNTDDIVTNFMGVIAEVPKDVEDDFFVCFMSTWWGQYQLRVE*

>SARC_14637T0 | SARC_14637 | Sphaeroforma arctica JP610 hypothetical protein (329 aa)

MSLFGISPSKHLGGRCINAAKTIPKATTLLRASPLLAYSDTITCDYTAKQTTPHTNPQSQKHTDVHENLDNESINSSQTNSKYVFCPHCYRRACQGECLDMSKKSTSIYYSLIGSNDVSQVLRDNGGSDMTGRYNGMALKTLIRLFTASVDPNNDDFTCAFQSLCFTKNFYNRSGQTSRDGLSLKDEHALLAKAMQDMLGCGNDGVEWVNLALVERVFGALRLNSFGFKHEVDDSAEHRISAVYDIASYFNHSCTPNADVGNDGAMIEIITNQEVDVGQEIYVCYDYGNRDMDVGERKRALWMNYGFECGCKTCRDVQIDRSIRDGEV*

>SARC_15066T0 | SARC_15066 | Sphaeroforma arctica JP610 hypothetical protein (95 aa)

MHRELNNNTITLNEDVVIDVPLPYDSLTHYRNTLGHKANHSTRPNAKYDVFFHPVFGDIKCIRSIQDVKEGEEVVVDYGYSHETVPDWYSALKT*

>SARC_16651T0 | SARC_16651 | Sphaeroforma arctica JP610 hypothetical protein (112 aa)
[truncated: 102,910 more chars]
